# Inhibition of the Clathrin Terminal Domain—Amphiphysin Protein–Protein Interaction. Probing the Pitstop 2 Aromatic Moiety

**DOI:** 10.1002/cmdc.202500321

**Published:** 2025-07-11

**Authors:** Kate Prichard, Mark J. Robertson, Ngoc Chau, Jing Xue, Martin Neuenschwander, Uwe Fink, Andre Horatscheck, Marc Nazare, Phillip J. Robinson, Volker Haucke, Adam McCluskey

**Affiliations:** ^1^ Chemistry, School of Environmental & Life Sciences The University of Newcastle University Drive Callaghan NSW 2308 Australia; ^2^ Cell Signalling Unit Children's Medical Research Institute The University of Sydney Hawkesbury Road, Westmead Sydney NSW 2145 Australia; ^3^ Marc Nazare ‐ Chemical Biology Leibniz Institute fur Molecular Pharmacologie Robert‐Roessle‐Strasse 10 13125 Berlin Germany

**Keywords:** aromatic moiety, clathrin‐mediated endocytosis, library synthesis, molecular docking, pitstop

## Abstract

Pitstop 2, (*Z*)‐*N*‐(5‐(4‐bromenzylidene)‐4‐oxo‐4,5‐dihydrothiazol‐2‐yl)naphthalene‐1‐sulfonamide (**1**) inhibits the clathrin terminal domain–amphiphysin interaction (NTD‐PPI) and has been widely used to investigate endocytosis. Herein, the synthesis of 56 novel Pitstop 2 analogues via four discrete focused libraries is reported. Specific modification to the 4‐bromonenzylidene moiety is explored, and their ability to inhibit the NTD‐PPI interaction is examined by ELISA. In cell endocytosis, effects are measured for selective analogues as is the inhibition of dynamin, another protein involved in the endocytosis process. The most NTD‐PPI active analogues retain 2‐ and 4‐disposed substituents on the aromatic head, with 2,3,4‐trihydroxy (**28**) the most active (IC_50_ 0.94 μm). Catechol‐free 2,3‐dihydroxybenzo[*b*][1,4]dioxone (**54**) is a more promising lead with an NTD‐PPI IC_50_ = 1.16 μm. The corresponding benzo[*d*][1,3]dioxole (**53**) is threefold less active suggesting ring size preference at this position. Nine analogues show improved or comparable NTD‐PPI activity to Pitstop 2 with IC_50_ values from 0.94 to 2.1 μM. Heterocyclic analogues are well tolerated and potent inhibitors of CME in U2OS cells, in particular, benzofuran **67** (NTD‐PPI IC_50_ 1.5 μm and CME IC_50_ 6.8 ± 2.7 μm). This positions **67** as one of the most cell active inhibitors of clathrin‐mediated endocytosis yet reported.

## Graphical abstract

Pitstop 2 binds to the clathrin terminal domain. This work explores the nature of the benzylidene moiety, R. The synthesis of 56 novel analogues reveals enhanced potency of a multiple analogues, with catechol‐free 2,3‐dihydroxybenzo[*b*][1,4]dioxone (**54**) returning a 1.2 μm IC_50_, ca 10‐fold more active than Pitstop 2.

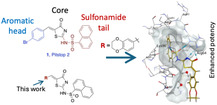

## 1 Introduction

Endocytosis is critical to cellular well‐being. It is the process whereby cellular material (or cargo) spanning hormones, nutrients, and receptors is internalized by cells. Among the key mechanisms involved in this process, clathrin‐mediated endocytosis (CME) is perhaps the best studied.^[^ ^1^, ^2^, ^3^, ^4^ ^]^ CME is a highly orchestrated process involving specific recruitment of proteins responsible for cargo selection, it requires the interplay of at least 30 additional proteins to enable initiation of membrane curvature, cargo selection, clathrin coat assembly, and ultimately scission and uncoating of the clathrin‐coated vesicle.^[^ ^1^, ^3^, ^5^, ^6^, ^7^, ^8^ ^]^ The hi‐jacking of CME has been implicated in the entry of viruses^[^ ^9^, ^10^ ^]^ such as Ebola,^[^ ^11^, ^12^ ^]^ Simian hemorrhagic Fever,^[^ ^13^ ^]^ HIV‐AIDS,^[^ ^14^, ^15^ ^]^ and SARS‐CoV‐2 into cells.^[^ ^16^ ^]^

In addition to its primary role in CME, clathrin has also been reported to have a “moonlighting role” in mitosis.^[^ ^17^, ^18^, ^19^, ^20^ ^]^ Clathrin plays an essential role in the assembly of the mitotic spindle interacting with the phosphorylated transforming acidic coil‐coiled containing protein 3 (TACC3) responsible for microtubule stabilisation.^[^ ^20^, ^21^, ^22^ ^]^

Clathrin itself is a protein complex composed of three 190 kDa heavy chain subunits and three 24–27 kDa light chain subunits. Assembly of these subunits generates the clathrin triskelion where each heavy chain contains one N‐terminal domain (NTD), a seven‐bladed β‐propeller which enables interaction with clathrin‐associated proteins.^[^ ^17^, ^23^, ^24^ ^]^ Within the clathrin β‐propeller, there are five protein binding sites.^[^ ^5^, ^25^, ^26^, ^27^ ^]^ These sites allow interaction with the adaptors proteins (AP‐1 and AP‐3),^[^ ^28^, ^29^, ^30^ ^]^ as well as TACC‐3.^[^ ^20^, ^21^, ^22^ ^]^ Clathrin ligands typically contain “clathrin‐box” motifs. The motif usually comprises of a LФXФ [DE] peptide sequence, where Ф is a bulky amino acid and X can be any amino acid.^[^ ^26^, ^31^, ^32^ ^]^ This enables binding at well‐defined sites in the clathrin seven‐bladed β‐propeller.^[^ ^23^, ^25^, ^27^ ^]^ Buried within the propeller are three hydrophobic binding sites responsible for the majority of clathrin protein–protein interactions. The majority of known interactions occur in “site‐1” between blades 1 and 2. A second site between blades 4 and 5 was discovered by co‐crystallization.^[^ ^5^, ^33^, ^34^ ^]^ The third site is speculated from molecular dynamics studies involving the marine natural product bolinaquinone and is proposed to exist between blades 5 and 6.^[^ ^35^, ^36^, ^37^ ^]^ From an inhibition of clathrin perspective, this presents a significant challenge for small molecules as most of the known clathrin ligands interact with the largest of the hydrophobic pockets (>1500 Å^2^), and this pocket contains exclusively hydrophobic residues.^[^ ^7^, ^38^, ^39^ ^]^ Most of the recognized hotspots are surface located, conspiring to increase the difficulty of developing small molecules capable of inhibiting key protein–protein interactions.^[^ ^40^, ^41^, ^42^ ^]^

**Figure 1:**
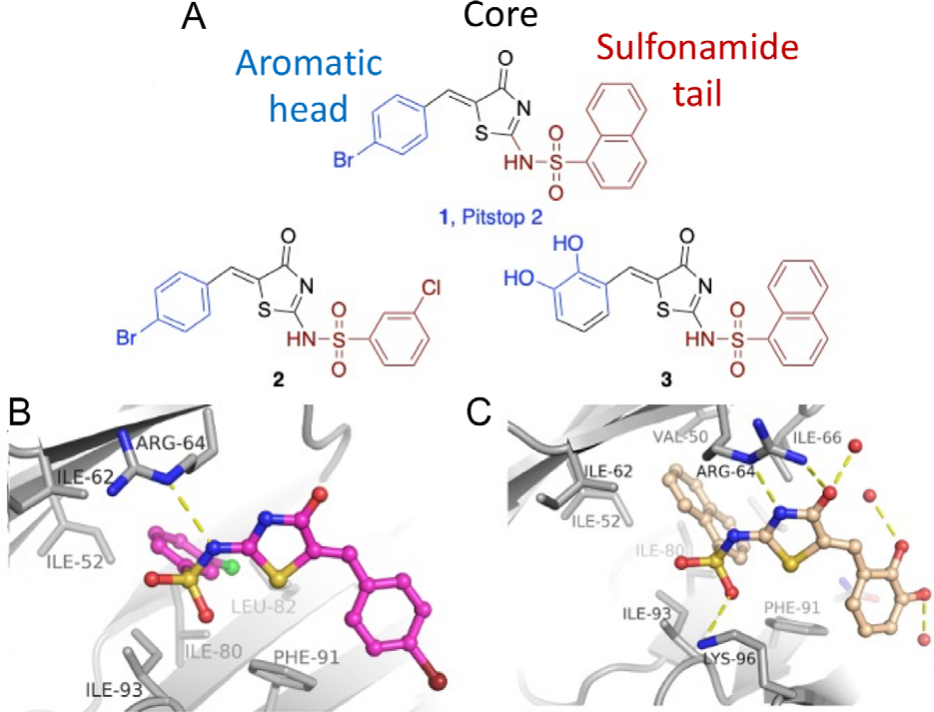
A) Chemical structures of Pitstop 2 (**1**) and analogs **2** and **3** with IC_50_ values of 1.9, 5.1, and 3.5 μm, respectively. B) Binding modes of each Pitstop compound in complex with the CTD are shown separately **2** (B), and **3** C). Water molecules in hydrogen bond distance to compounds are shown as red dots. Analogs **2** and **3** exhibit the same binding mode as Pitstop 2.

Recently, we reported on the first two inhibitors of clathrin, Pitstop 1 and Pitstop 2, which co‐crystallography revealed binding at site‐1.^[^ ^43^ ^]^ We have also reported on preliminary SAR development around the Pitstop 2 compound class of compounds including the co‐crystal structures of analogues 1 and 2 (**Figure** **1**). Previous SAR developments yielded two additional compounds (Pitstop 2c and Pitstop 2d), which displayed clathrin inhibition along with low cytotoxicity.^[^ ^44^ ^]^ These data suggested that additional modifications to the alkylidene and sulfonamide moiety were feasible and encourage the development of an extensive SAR exploration.^[^ ^45^ ^]^ Herein, we explore the nature of these moieties and their ability to inhibit the clathrin‐amphiphysin protein–protein interaction (NTD‐PPI).

## 2 Results and Discussion

**Scheme 1:**
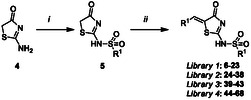
Reagents and conditions: (i) NaH, naphthalene sulfonylchloride, DMF, RT, 18 h; and (ii) R^1^CHO (see Table for detail), cat. piperidine/benzoic acid, EtOH, microwave irradiation, 120 °C, 20–90 min.

Our initial investigations explored the alkylidene requirements of the proposed Pitstop analogues. As with our previous reports, these analogues were readily accessed in a two‐step procedure: the introduction of the sulfonamide moiety followed by Knoevenagel condensation with an appropriate aldehyde. Thus, pseudothiohydantoin (**4**) was treated with NaH in DMF, followed by the addition of naphthalene sulfonyl chloride to give **5.** Piperidine/benzoic acid catalysed Knoevenagel condensation with a range of aldehydes (1.1 to 2.4 equiv.) under microwave conditions (120 °C, 200 W, 20–90 min) gave analogues **6**–**23** in modest (**8**, 35%) to excellent (**10**, **18**, 84%) yields (**Scheme** **1**).^[^ ^43^, ^45^, ^46^ ^]^

**Figure 2:**
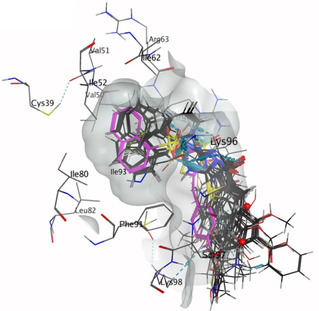
Docked structures of selected Pitstop 2 (**1**) analogs with modified substituents in the phenyl aromatic region.

In developing, *Library 1* (**6**–**23**) an *in silico* docking analysis was conducted with each analogue docked in the site‐1 binding pocket known to be occupied on Pitstop 2 inhibition. The predicted binding poses suggest that most *Library 1* analogues would adopt similar poses to Pitstop 2 (**Figure** **2**). The outcomes of ELISA evaluating these analogues are shown in **Table** **1**.

**Table 1:** Inhibition of the NTD‐PPI determined by ELISA and the inhibition of dynamin (IC_50_ μm) by *library* 1 Pitstop 2 analogs (**6**‐**23)**.

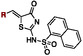								
R	NTD‐PPI IC_50_ [μM]a)	Dyn 1 IC_50_ [μM]c)	R	NTD‐PPI IC_50_ [μM]a)	Dyn 1 IC_50_ [μM]	R	NTD‐PPI IC_50_ [μM]a)	Dyn 1 IC_50_ [μM]
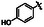 **6**	5.6	–b), d)	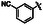 **12**	5.7	–b), d)	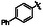 **18**	2.1	85d)
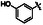 **7**	7.9	–b), d)	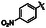 **13**	5.2	≈56d)	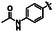 **19**	3.5	93 ± 2.5e)
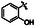 **8**	2.1	–b), d)	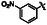 **14**	5.9	–b), d)	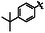 **20**	4.6	–b), d)
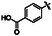 **9**	7.4	–b), d)	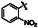 **15**	153	42.2d)	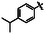 **21**	4.1	–b), d)
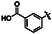 **10**	5.3	–b), d)	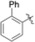 **16**	4.09	–	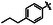 **22**	4	≈91.0
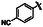 **11**	6.3	–b), d)	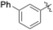 **17**	4.02	–	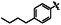 **23**	3.5	≈103.5

a) ELISA IC_50_ values represent the average of duplicate measurements (n=2);

b) ‘‐’ = not active at 150 μm compound concentration;

c) Dynamin IC_50_, *n* = 1 unless otherwise noted;

d) Dynamin IC_50_, *n* = 1;

e) Dynamin IC_50_, *n* = 2.

**Figure 3:**
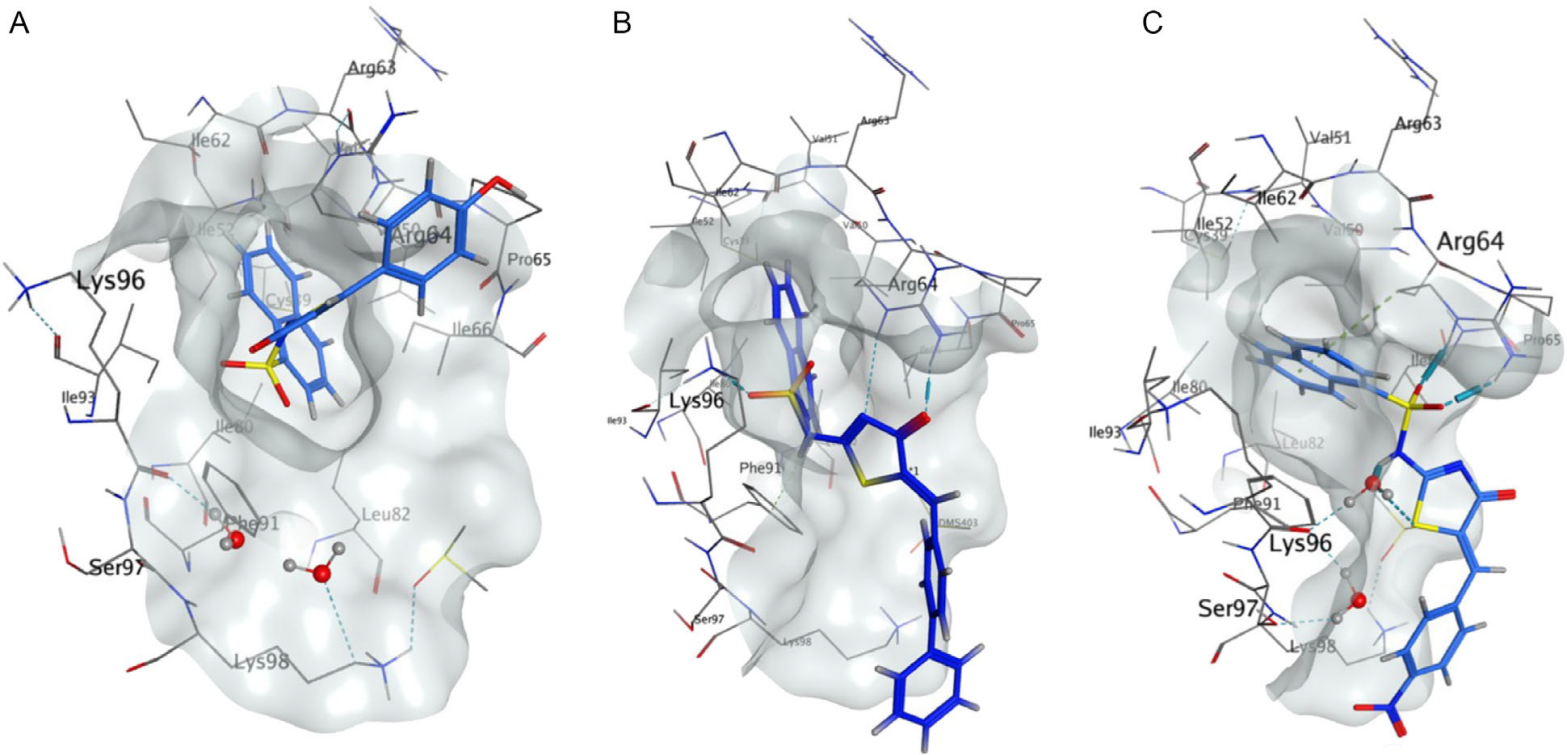
Predicted binding of selected 4‐position substituted analogs *from Library 1* docked into the NTD site‐1 (PDB: 4G55).^2^ A) Predicted binding pose of 4‐phenoxy **6**. B) Predicted binding pose of 4‐biphenyl **18**. C) Predicted binding pose of 4‐nitrophenyl **13**.

Modification of the 4‐Br moiety to 4‐OH (**6**) was well tolerated (IC_50_ 2.7 μm) with the position of the ‐OH moiety affecting the observed activity (3‐OH (**7**), IC_50_ 5.5 μm and 2‐OH, (**8**) IC_50_ 2.1 μm). Carboxylates **9** and **10** showed reduced potency as did the ‐CN **11** and **12**, most notable was the poor activity of the ‐NO_2_ analogues, a >100‐fold potency reduction was noted with 2‐NO_2_ **15**. The introduction of simple hydrophobic groups from 4‐Ph (**18**) to 4‐*tert* butyl (**20**) and 4‐butyl (**23**) retained modest activity with IC_50_ values of 1.4–4.6 μm (Figure S1, Supporting Information). Docking analysis supported retention of the Lys96 to 1‐naphthalene and Arg64 to pseudothiohydantoin H‐bonds; Phe91 and 1‐naphthalenesulfonamide *π–H* interactions (**Figure** **3**A,B). In the cases of ‐NO_2_ **13–15** with no major interactions with the naphthalene moiety, and a “side on” binding disposition in orientation leaving a large portion of the Site‐1 binding pocket unoccupied was observed (Figure 3C).

**Figure 4:**
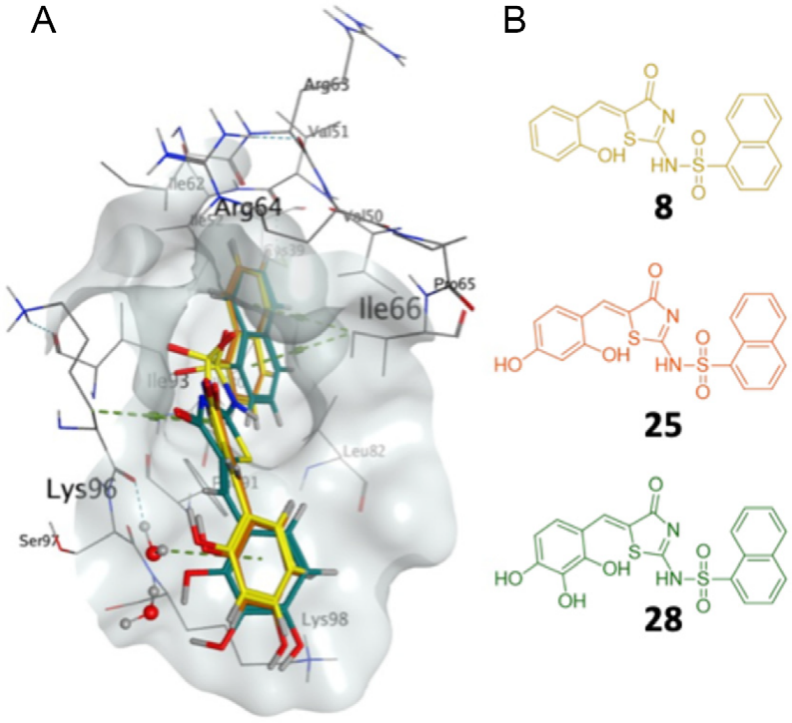
Predicted binding of selected phenoxy analogs from Library 3A docked into the CTD (Site 1). PDB: 4G55.^2^ A 2‐Phenoxy **8** (yellow), 2,4‐dihydroxyphenyl **25** (orange), and 2,3,4‐trihydroxyphenyl **28** (green) docked into the CTD. B Chemical structures of **8**, **25** and **28**.

The effect of increasing the number of substituents on the aromatic moiety was explored through the synthesis (Scheme 1) and ELISA evaluation of *Library 2* (**Table** **2**). Analogues retaining a 4‐disposed substituent were favored with 2,4‐dihydroxyphenyl **25** (IC_50_ 1.8 μm) and 3,4‐dihydroxyphenyl **27** (IC_50_ 1.6 μm). The 2,3‐dihydoxyphenyl **24** and 2,5‐dihydoxyphenyl **26** were 3‐ and 5‐fold less active. Introduction of a third ‐OH moiety with **28** saw sub micromolar potency. With the dihydroxy analogues (**24**, **25**, and **27**), these effects did not appear to be mediated by increased H‐bond donation as the equivalent methoxy analogues (**29–31**) were essentially equipotent. The docked poses of **25** and **27** were similar with H‐bonds to Lys96 and the aromatic ring forming a water‐mediated *π–H* interaction at Lys96. Analogue **24** showed interactions with Ile66, Arg64 and a water‐mediated interaction with Lys96 (at the hydroxy moiety). The binding poses of mono‐ (**8**), di‐ (**25** and tri‐ (**28**) hydroxy analogues are essentially identical (**Figure** **4**). Analogues with substituents in the 2,4‐position (**25**, **30** and **35**) bind in essentially the same orientation with equivalent activities (Figure S2, Supporting Information) Whereas, di‐Cl **36** presents with an altered 1‐naphthalene disposition, with a twist observed, which modifies the occupancy of the site‐1 binding pocket increasing the solvent exposure of the aromatic head (Figure S2, Supporting Information).

**Table 2:** Inhibition of the NTD‐PPI determined by ELISA and the inhibition of dynamin (IC_50_ μm) by *library* 2 Pitstop 2 analogs (**24**‐**38)**.

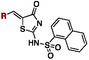								
R	NTD‐PPI IC_50_ [μM]a)	Dyn 1 IC_50_ (μM)c)	R	NTD‐PPI IC_50_ [μM]a)	Dyn 1 IC_50_ [μM]c)	R	NTD‐PPI IC_50_ [μM]a)	Dyn1 IC_50_ [μM]c)
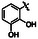 **24**	3.9	85	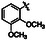 **29**	8.3	– b),, c)	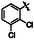 **34**	26.1	–b),, c)
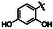 **25**	1.8	61.2 ± 7.5	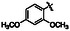 **30**	1.0	–b),, c)	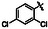 **35**	1.5	66.8
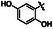 **26**	10.7	70.0 ± 5.3	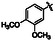 **31**	2.2	91	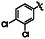 **36**	4.1	95.1 ± 7.4
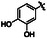 **27**	1.6	50.4 ± 8.4	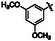 **32**	3.4	–b),, c)	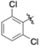 **37**	4.05	
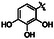 **28**	0.94	34.3 ± 3.4	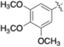 **33**	14.1	–b),, c)	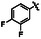 **38**	5.4	–

a) ELISA IC_50_ values represent the average of duplicate measurements (n=2);

b) ‘‐’ =not active at 100 μm compound concentration;

c) Dynamin IC_50_, *n* = 3, triplicate.

To further examine the requirements for an aromatic moiety, aliphatic substituted *Library 3*, **39–43** was synthesized (Scheme 1).

ELISA evaluation revealed an at least 2‐fold NTD‐PPI potency reduction with **39–43** (**Table** **3**). Docking studies were consistent with these analogues forming limited interactions within the site‐1 pocket, in keeping with the observed ELISA activities (Figure S3, Supporting Information).

**Table 3:** Inhibition of the NTD‐PPI determined by ELISA and the inhibition of dynamin (IC_50_ μm) by *library* 3 Pitstop 2 analogues (**39**‐**43)**.

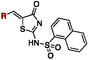		
R group	NTD‐PPI IC_50_ [μM]a)	Dynamin 1 IC_50_ [μM]**b**)
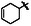 **39**	4.3	NDb),, c)
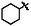 **40**	8.6	–b),, d)
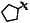 **41**	5.5	–d),, e)
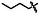 **42**	6.5	–d),, e)
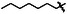 **43**	5.9	NDb),, c)

a) ELISA IC_50_ values represent the average of duplicate measurements (*n* = 2);

b) Dynamin IC_50_, *n* = 1, unless otherwise stated;

c) ND – not determined;

d) ‘‐’ =not active at 100 μm compound concentration;

e) Dynamin IC_50_, *n* = 2.

Next, the introduction of heterocyclic systems was investigated and NTD‐PPI inhibition examined by ELISA (**Table** **4**). Commencing with thiophene (**44**),^[^ ^47^ ^]^ a range of 5‐membered heterocycles were installed with pyrrole (**45**, **47**) and furans (**46**, **48–51**). The S to NH to O isosteres (**44–46**, respectively) show no heteroatom preference. There is a modest effect with C2 linked analogues, with pyrrole (**47**) 2‐fold less active than furan (**48**). The O and S analogues are equipotent with Pitstop 2. Halogenated furans (**49**, **50**) were well tolerated, but CH_2_OH (**51**) was threefold less active, consistent with the H‐bonding effects noted with pyrroles **45** and **47** (Table 3), as is the docked poses of these analogues (Figure S4, Supporting Information).

**Table 4:** Inhibition of the NTD‐PPI determined by ELISA and the inhibition of dynamin (IC_50_ μm) by *library* 4 Pitstop 2 analogs (**44**‐**68)**.

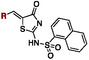								
R group	NTD‐PPI IC_50_ [μM]a)	Dynamin 1 IC_50_ [μM]	R group	NTD‐PPI IC_50_ [μM]a)	Dynamin 1 IC_50_ [μM]	R group	NTD‐PPI IC_50_ [μM]a)	Dynamin 1 IC_50_ [μM]
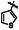 **44**	2.34	NDb)	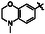 **53**	3.3	100.7 ± 1.0e)	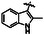 **62**	2.37	–c),, d)
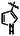 **45**	3	NDb)	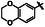 **54**	1.2	60.1 ± 8.6e)	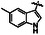 **63**	3.52	–c),, d)
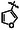 **46**	2.11	NDb)	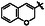 **55**	2.6	69.3 ± 7.1e)	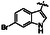 **64**	3.42	–c),, d)
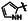 **47**	5.3	NDb)	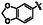 **56**	3.2	–c),, d)	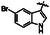 **65**	2.91	–c),, d)
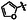 **48**	3.64	NDb)	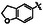 **57**	3.27	–c),, d)	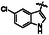 **66**	2.85	–c),, d)
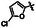 **49**	3.31	NDb)	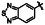 **58**	4.41	≈69.3d)	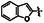 **67**	1.5	NDb)
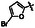 **50**	2.88	NDb)	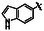 **59**	3.66	83.8 ± 10.7e)	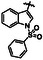 **68**	27	99.8 ± 3.1e)
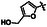 **51**	6.33	ND b)	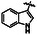 **60**	3.2	–b),, c)			
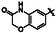 **52**	3.33	≈54.1d)	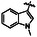 **61**	3.05	NA			

a) ELISA IC_50_ values represent the average of duplicate measurements (*n* = 2);

b) ND =not determined;

c) ‘‐’ =not active at 100 μm compound concentration;

d) Dynamin IC_50_, *n* = 1;

e) Dynamin IC_50_, *n* = 2.

More complex heterocycles were accessed as per Scheme 1 but under variable conditions. Most analogues were accessed in <90 min (up from 20 min) microwave irradiation. Increased aldehyde equivalence from 1.1 to 2.4 equivalents was required. Most analogues were isolated by filtration, some required chromatographic separation (benzofuran **67**) or addition of H_2_O (furan‐2‐ylmethanol **51**) to form a precipitate. These analogues were screened as before in the NTD‐PPI ELISA (Table 4)

In general, these heterocyclic substituents were well tolerated; benzoxazinone (**52**), dihydrobenzoxazine (**53**), chromene (**55**), methylenedioxy (**56**), dihydrobenzofuran (**57**), and benzothiadiazole (**58**) analogues returned IC_50_ values <5 μM and indole analogues (**59–66**) IC_50_ values <4 μm (Table 4).

**Figure 5:**
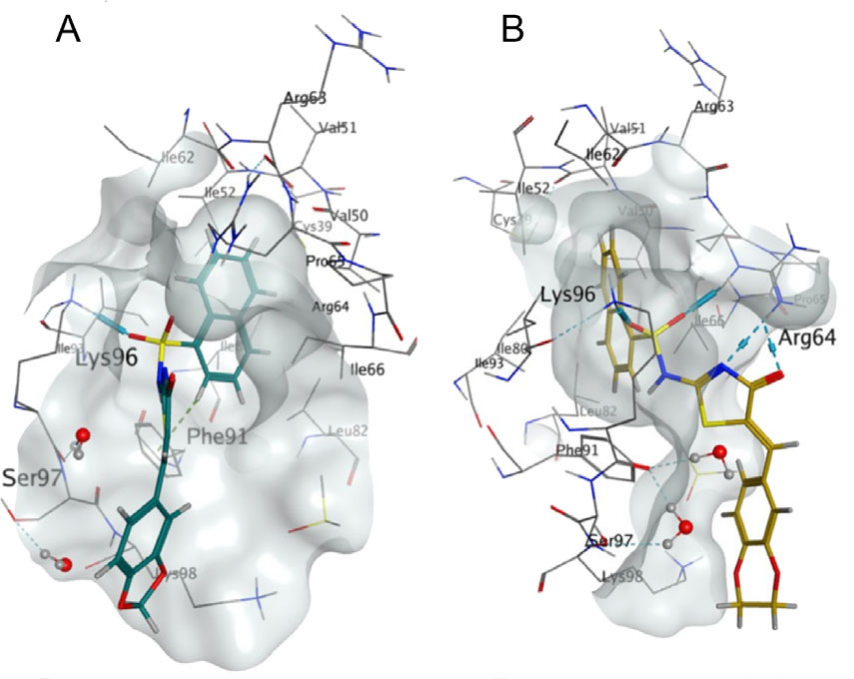
Predicted binding of selected heterocyclic analogs from *Library 4* docked into the CTD (Site 1). PDB: 4G55.^2^ **A**. Predicted binding pose of methylenedioxy **56** (IC_50_ 4 μm). **B**. Predicted binding pose of dihydrobenzo[*b*][1,4]dioxine **54** (IC_50_ 1.2 μm).

The oxygen‐containing heterocycles **53–57** and **67** were well tolerated (IC_50_'s 1.2–4 μm). Analogue **54** showed a >3‐fold activity increase relative to **56**, suggesting the 6‐membered bicyclic moiety is preferred. The predicted orientations of **54** and **56** are similar, although the aromatic head of **54** appears less solvent exposed in closer contact with the side of the site‐1 pocket. Methylenedioxy **56** forms a H‐bond with Lys96 along with a *π–H* interaction with Phe91. Dihydrobenzo[*b*][1,4]dioxine **54** also forms H‐bonding interactions with Lys96 and Arg64, at both the 1‐naphthalenesulfonamide and pseudothiohydantoin. The increased activity in the dihydrobenzo[*b*][1,4]dioxine **54** is likely a result of the more favorable pose, along with the interaction with Arg64 (**Figure** **5**).

Benzofuran **67** was twofold more active indoles **60** and **59** (Table 4). The docked poses of benzofuran **67** and indoles **59** and **60** were similar. In each case, specifically favorable interactions are observed with Arg64 (H‐bonding and *π–H*), Lys96 (water‐mediated H‐bonding) and Ser97 (water‐mediated H‐bonding). The more active benzofuran **67** showed an additional H‐bonding interaction with Arg64 and a less solvent exposed head region (Figure S4, Supporting Information). The methyl substituted 3‐indole analogues demonstrated a high methyl group moiety tolerance with IC_50_ values of 3, 2.4, and 3.5 μm for **61–63**, respectively. A similar substituent tolerance is noted for the unsubstituted and halogen substituted indoles, **60**, **64**, and **66** with IC_50_ values of 3.2, 3.4 and 2.8 μm, respectively (Figure S5, S6, Supporting Information).

### 2.1 In Cell Inhibition of Clathrin‐Mediated Endocytosis

Given the observed effects of most of these analogues inhibiting the NTD‐PPI by ELISA, selected examples were examined for their ability to block the uptake of a transferrin‐red dye, a measure of in‐cell endocytosis block. Using our previously reported CME assay in U2OS cells, the ability of these compounds to block endocytosis was examined (**Table** **5**).^[^ ^48^, ^49^ ^]^

**Table 5:** Inhibition of transferrin‐red uptake in U2OS cells, NTD‐PPI and Dynamin (IC_50_, μm) by 6, 7, 13‐15, 18, 4‐52, 54, 56‐58, 67, and 68 (IC_50_, μm), the ELISA inhibition of CTD‐Amp and the inhibition of dynamin GTPase (μm).

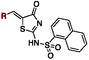							
R group	CME IC_50_ [μM]a)	NTD‐PPI IC_50_ [μM]b)	Dynamin 1 IC_50_ [μM]a)	R group	CME IC_50_ [μM]a)	NTD‐PPI IC_50_ [μM]b)	Dynamin 1 IC_50_ [μM]a)
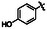 **6**	46.7 ± 13.1	5.6	NAb),, c)	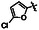 **49**	52.6 ± 9.1	3.31	ND
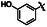 **7**	25.0 ± 14.6	7.9	NAb),, c)	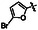 **50**	42.3 ± 25.7	2.88	ND
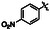 **13**	≈14.6	5.2	55.8c)	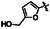 **51**	≈155.6	6.33	ND
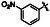 **14**	34.4 ± 13.7	5.9	NAb),, c)	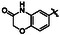 **52**	NAc)	3.33	≈54.1c)
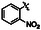 **15**	46.3 ± 13.7	153	≈42.4c)	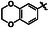 **54**	≈35.0	1.2	60.1 ± 8.6c),, e)
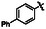 **18**	≈43.1	1.4	85.3	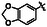 **56**	19.4 ± 10.5	3.2	NDd)
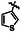 **44**	36.4 ± 13.7	2.34	NDd)	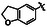 **57**	58.1 ± 12.6	3.29	NAc)
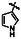 **45**	≈108.1	3	NDd)	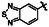 **58**	16.8 ± 7.9	4.41	≈69.3c)
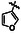 **46**	31.5 ± 9.2	2.11	NDd)	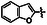 **67**	6.8 ± 2.7	1.5	NDd)
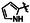 **47**	27.2 ± 11.7	5.3	NDd)	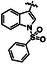 **68**	–e)	27	99.8 ± 3.1e)
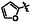 **48**	≈96.6	3.64	NDd)				

a) ELISA IC_50_ values represent the average of duplicate measurements (*n* = 2);

b) ‘‐’ = not active at 100 μm concentration;

c) dynamin IC_50_, *n* = 1;

d) ND = not determined;

e) Dynamin IC_50_, *n* = 2.

From analysis of the Table 5 data, it is clear that CME inhibition is low relative to the NTD‐PPI data (c.f. Table 1, 2, 3, 4, 5). We have observed similar outcomes on comparing dynamin and CME inhibition, in part attributed to variations in analogue cell penetration.^[^ ^50^, ^51^, ^52^ ^]^ The promising NTD‐PPI activities of oxygen heterocycles did not often translate to the whole cell assay, exemplified by the dihydrobenzo[*b*][1,4]dioxine **54** (CME IC_50_ ≈ 35 μM), a threefold decrease in CME activity compared with Pitstop 2. Despite the comparable NTD‐PPI activities of the methylenedioxy **56** and dihydrobenzofuran **57**, a threefold increase in CME inhibition was observed on inclusion of the second oxygen with **56** (Table 5). With CME IC_50_ values of >50 μm **45**, **48**, **49**, **51**, **52**, and **57** are essentially CME inactive. This may be a consequence of poor cell penetration or efficient efflux pumps. Of the remaining analogues, **13**, **56**, and **58** display activity comparable to Pitstop 2. Benzofuran **67** is 2‐3‐fold more active than Pitstop 2 and the most CME analogue examined herein. It is also amongst the most ELISA active analogues examined. The most active **28** (NTD‐PPI IC_50_ 0.94 μM) was not explored in the U2OS assay as catechols are presumed promiscuous.^[^ ^53^ ^]^ As our primary target is the NTD‐PPI, careful consideration has been given to the examination of potential non‐specific off‐target effects, e.g., against dynamin.

### 2.2 Dynamin Activity, an off‐Target Effect in CME

The large GTPase dynamin acts within the endocytic path downstream of clathrin and is involved in the final stage severing of the clathrin‐coated pit from the plasma membrane and the release of the vesicle into the cell. As such analogues that inhibit dynamin are also known to block CME in our U2OS assay. Screening selected analogues for dynamin activity showed that most were poor dynamin inhibitors, indole (**59**) 83.8 μm), acetamide (**19**) (IC_50_ 92.7 μm), propyl (**22**) (IC_50_ 91 μm), butyl (**23**) (IC_50_ 103 μm) dihydrobenzo[*b*][1,4]dioxine (**54**) (IC_50_ 60 μM), chromene (**55**) (IC_50_ 69 μM), and phenylsulfonylindole **68** (IC_50_ 99.8 ± 3.1 μM) (Table 1, 2, 3, 4).

### 2.3 Molecular Docking and Binding NTD‐PPI Inhibition

**Figure 6:**
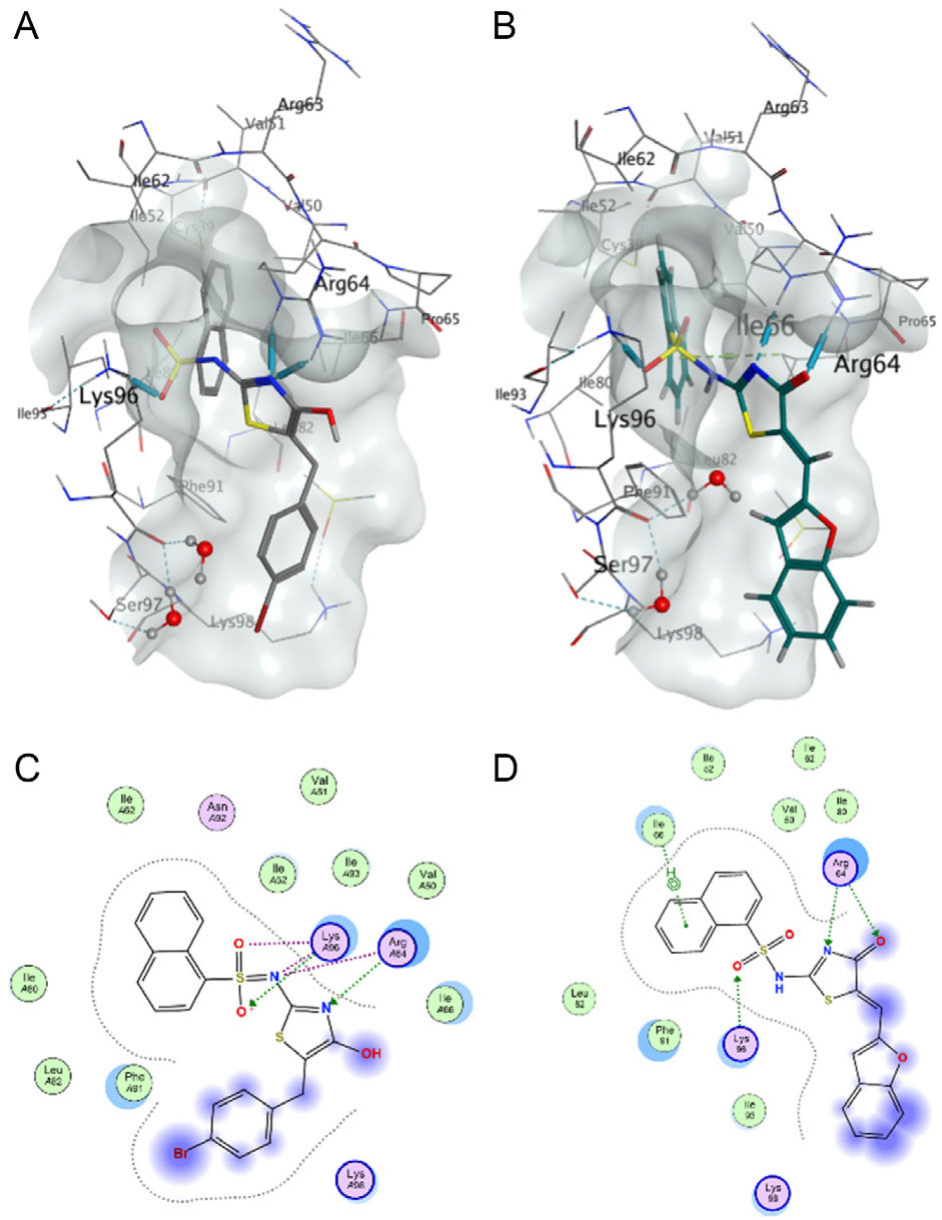
A comparison of Pitstop 2 (**1**) and an exemplar analog from *Library 4* displaying “typical” interactions in the CTD (Site 1). PDB: 4G55.^2^ A Binding pose of Pitstop 2, from the co‐crystal. B Predicted binding pose of benzofuran **67** from *Library 4*. C Molecular operating environment (MOE) generated ligand map of Pitstop 2, showing interactions with Arg64 and Lys96. D MOE generated ligand map of benzofuran **67** from *Library 4*, showing interactions with Lys96, Arg64 and Ile66.

The docked conformations of *Libraries 1‐4* were examined relative to the co‐crystal structure pose of Pitstop 2 (site‐1) in the clathrin terminal domain.^[^ ^43^ ^]^ Some interactions were consistent with all analogues docked. These included H‐bonding between the pseudothiohydantoin core and Arg64, and H‐bonding between the 1‐naphthalenesulfonamide and Lys96, which are also observed with Pitstop 2. Another common interaction observed was *π–H* between the 1‐naphthalenesulfonamide and Ile66 in the back of the pocket (**Figure** **6**).

## 3 Conclusions

A total of 56 compounds, in four discrete focused libraries were synthesized and ELISA screened for their ability to inhibit the NTD‐PPI. This was an interrogation of the key binding requirements of the aromatic head moiety of lead Pitstop 2. The data obtained are consistent with the predicted binding pose (based on the Pitstop 2 co‐crystal structure with the CTD).^[^ ^43^ ^]^ As anticipated wide substrate tolerance was observed, with the nature of the protein–protein interaction surface lending itself to multiple modifications to the aromatic head, with the naphthalene moiety buried within the binding pocket. The best NTD‐PPI affinities were noted with 2‐ and 4‐disposed substituents on the aromatic head, with 2,3,4‐trihydroxy (**28**) the most active (IC_50_ 0.94 μm). This catechol analogue was not explored further due to purported promiscuity.^[^ ^53^ ^]^ Further modifications gave 2,3‐dihydroxybenzo[*b*][1,4]dioxone (**54**) with a NTD‐PPI IC_50_ = 1.16 μm). The corresponding benzo[*d*][1,3]dioxole (**56**) was threefold less active suggesting a preference for a 6 (versus 5) membered ring. Nine analogues showed improved or comparable NTD‐PPI activity to Pitstop 2 with IC_50_ values ranging from 0.94–2.1 μM.

Heterocyclic analogues were well tolerated and potent inhibitors of CME in U2OS cells, in particular, benzofuran **67** (ELISA IC_50_ 1.5 μm and CME IC_50_ 6.8 ± 2.7 μm). This positions **67** as among the most active in cell inhibitor of clathrin‐mediated endocytosis yet reported.^[^ ^49^, ^52^ ^]^

## 4 Experimental Section

4.1

4.1.1

##### General Methods: Biology: ELISA‐Based Clathrin Inhibitor Binding Assay

The clathrin inhibitor assay was based inhibition of protein–protein interaction (PPI) between clathrin and amphiphysin as described in our previous report.^[^ ^45^, ^54^ ^]^ Bacterially expressed recombinant purified His_6_‐tagged amphiphysin 1 B/C domain (which comprises both the clathrin and the AP‐2 binding sites, amino acids 250‐578^[^ ^55^ ^]^) in screening buffer (20 mM HEPES, pH 7.4, 50 mM NaCl, 1 mM DTT, 1 mM PMSF) was added to a 384 well ELISA plate (Corning Maxisorp 3700) and bound to the plastic for 1 h at room temperature. Nonspecific binding was reduced by overnight incubation with 50 μL blocking buffer (20 mM HEPES, pH 7.4, 50 mM NaCl, 1 mM PMSF, 2% BSA, 2.5% skim milk) at 4 °C. Following extensive washes (with 20 mM HEPES, pH 7.4, 50 mM NaCl, 0.05% Tween 20), chemical compounds diluted in DMSO (10 μL) were added and incubated together with bacterially expressed recombinant GST‐tagged clathrin heavy chain NTD (amino acids 1–364) for 1 h at room temperature in screening buffer. After 3 washes, horseradish peroxidase‐coupled anti‐GST antibodies were added in screening buffer and the plate incubated for 40 min at room temperature. Following additional washes, 50 μL TMB (3,3′,5,5′‐tetramethylbenzidine) (Pierce Biotechnology) chromogenic substrate of horseradish peroxidase was added, and the plate was incubated for 10 min before the reaction was terminated by adding 50 μL 1N sulfuric acid to produce a yellow colour. The amount of bound protein was determined by spectrophotometric measurement in a plate reader. Relative binding was calculated as a percentage of DMSO control. From concentration–response curves, the IC_50_ value was calculated as the compound concentration at which PPI was 50% inhibited.

##### Dynamin IC_50_

Dynamin GTPase activity was determined in vitro using the Malachite Green phosphate detection absorbance‐based assay within a 96‐well plate format as described previously. The enzyme source was full length human Dynamin I expressed in Sf21 insect cells and affinity‐purified on GST‐Amph2‐SH3 (rat Amphiphysin 2 (494–588)).^[^ ^56^ ^]^

##### Clathrin‐Mediated Endocytosis Assay

CME was examined by a high‐throughput, quantitative assay for measuring transferrin (Tfn) uptake in U2OS osteosarcoma cells, based on methods described previously.^[^ ^48^ ^]^

Test compounds were made up as stock solutions of 30 mM in 100% DMSO and further diluted in assay buffer such that the final DMSO concentration was no greater than 3.3%. Compounds were made up fresh from powder stock just prior to commencing the assay, and stock solutions were stored at −20 °C for several months.

##### Molecular Docking of Compounds

The structures of all ligands to be docked were constructed in MOE and their conformations energy‐minimized using Molecular Mechanics in conjunction with the MMFF94x force field. The binding site for docking was defined by the position of co‐crystallized Pitstop 1 and Pitstop 2 in the clathrin terminal domain proteins, PDB: 4G55.^[^ ^43^ ^]^

Docking was performed using MOE's default settings, using the triangle matcher method in combination with the London dG scoring function for the initial placement of the ligand, followed by a refinement of the 30 top poses with rigid receptor setting and the GBVI/WSA scoring function. Analysis and visualization of the docking output, such as identification of hydrogen bonds, steric clashes, hydrophobic interactions, or *π*–*π* interactions were performed in MOE.^[^ ^57^ ^]^

##### Chemistry

All reactions were performed using standard laboratory equipment and glassware. Solvents and reagents were purchased from Sigma Aldrich, Alfa Aesar or AK Scientific and used as received. Organic solvents were of bulk quality and distilled from glass prior to use. Organic solvent extracts were dried over magnesium sulfate (MgSO_4_), and the solvent removed under reduced pressure with either Büchi or Heidolph rotary evaporators. Melting points were recorded in open capillaries on a Büchi 565 Melting Point Apparatus. Where available, literature values are provided and appropriately referenced. Electrospray mass spectra were recorded using 10% DMSO/H_2_O or HPLC‐grade methanol or acetonitrile as carrier solvents on an Agilent Technologies 1260 Infinity UPLC system with a 6120 Quadrupole LC/MS in electrospray ionization (ESI) positive and negative modes. TLC was performed on Merck silica gel 60 F254 precoated aluminium plates with a thickness of 0.2 mm. Nuclear magnetic resonance (NMR) spectroscopy was performed on a Brüker Avance III 400 MHz spectrometer, where proton NMR (^1^H NMR) spectra and carbon NMR (^13^C NMR) spectra were acquired at 400 and 100 MHz respectively, or a Brüker Avance III 600 MHz spectrometer, where proton NMR (^1^H NMR) spectra and carbon NMR (^13^C NMR) spectra were acquired at 600 and 150 MHz respectively. All spectra were recorded in deuterated dimethyl sulfoxide (DMSO‐*d*_6_) obtained from Cambridge Isotope Laboratories Inc. Chemical shifts (d) were measured in parts per million (ppm) and referenced against the internal reference peaks. Coupling constants (*J*) were measured in Hertz (Hz). NMR assignments were determined through the interpretation of one‐ and two‐dimensional spectra. Multiplicities are denoted as singlet (s), broad singlet (bs), doublet (d), doublet of doublets (dd), triplet (t), quartet (q), triplet of doublets (td), doublet of triplets (dt), and multiplet (m). Peaks are listed in decreasing chemical shift in the following format: chemical shift integration (^1^H), multiplicity (^1^H), coupling constant (^1^H). All compounds generated and screened were >95% pure by HPLC–MS and ^1^H NMR analysis.

##### General Procedure 1.

Pseudothiohydantoin (25.8 mmol, 3 eq.) was stirred in DMF (15 mL) prior to the portionwise addition of NaH (25.8 mmol, 60% in mineral oil, 3 eq.) slowly over 10 min. After complete addition, the reaction mixture was stirred at room temperature for 30 min. Desired sulfonyl chloride (8.6 mmol, 1 eq.) was added portionwise to the solution over 10 min. The reaction mixture was stirred at room temperature for 18 h. 1 m HCl (25 mL) was added slowly until a precipitate was observed. The precipitate was collected by vacuum filtration and washed with 1 m HCl (10 mL), H_2_O (2 × 10 mL), ethanol (2 × 10 mL), and diethyl ether (2 × 10 mL). If no precipitate formed the solvent was removed and the residue dissolved in H_2_O (15 mL) and extracted with ethyl acetate (3 × 20 mL).

##### General Procedure 2

To a 10 mL reaction vessel was added sulfonamide precursor (1 eq.), aldehyde (1.1 eq.), piperidine/benzoic acid mix (1/1.5eq, 10% in EtOH, 3 drops), and ethanol (3 mL). The resulting suspension was heated using microwave irradiation (200 W, 120 °C) for 20–90 min. After cooling overnight, the resulting precipitate was filtered, washed with small portions of cold ethanol (2 × 5 mL), and cold diethyl ether (2 × 5 mL) and air dried. If no precipitate was formed the solvent was removed and the resulting residue was purified using column chromatography using a gradient from dichloromethane to 10% methanol in dichloromethane.

##### N‐(4‐oxo‐4,5‐dihydrothiazol‐2‐yl)naphthalene‐1‐sulfonamide (**5**)

Synthesized using General Procedure 1 with pseudothiohydantoin (3.07 g, 25.8 mmol, 3 eq.), NaH (1.04 g, 25.8 mmol, 3 eq.), and 1‐naphthalenesulfonyl chloride (1.20 g, 8.6 mmol, 1 eq.) to give the desired product as a yellow solid (2.30 g, 87%).

^1^H NMR (400 MHz, DMSO‐*d*_6_) *δ* 12.53 (s, 1H, NH), 8.58 (d, *J* = 8.5 Hz, 1H, H_12_), 8.26 (dd, *J* = 15.1, 7.8 Hz, 2H, H_9_ and H_7_), 8.11 (d, *J* = 8.0 Hz, 1H, H_5_), 7.76 – 7.66 (m, 3H, H_11_, H_10_, and H_6_), 4.04 (s, 2H, H_1_) ppm.

NOTE: compound identified by NMR—compared to previous synthesis and used without further purification (small DMF impurity identified by ^1^H NMR).

##### Library 1: (Z)‐N‐(5‐(4‐hydroxybenzylidene)‐4‐oxo‐4,5‐dihydrothiazol‐2‐yl)naphthalene‐1‐sulfonamide 6

Synthesized using General Procedure 2 with **5** (155 mg, 0.51 mmol, 1 eq.) and 4‐hydroxybenzaldehyde (66 mg, 0.54 mmol, 1.1 eq.) for 30 min. Addition of hexane (≈2 mL) allowed precipitation to give the desired product as a bright yellow solid (159 mg, 77%). M.P: >255 °C (dec). *R*_f_ 0.15 (5% MeOH in CH_2_Cl_2_).

^1^H NMR (400 MHz, DMSO‐*d*_6_) *δ* 13.05 (s, 1H, NH), 10.45 (s, 1H, OH), 8.61 (d, *J* = 8.6 Hz, 1H), 8.31 (dd, *J* = 7.7, 3.8 Hz, 2H), 8.13 (d, *J* = 8.0 Hz, 1H), 7.77 (t, *J* = 7.2 Hz, 1H), 7.71 – 7.68 (m, 3H), 7.55 (d, *J* = 8.6 Hz, 2H), 6.99 (d, *J* = 8.6 Hz, 2H) ppm. ^13^C NMR (101 MHz, DMSO‐*d*_6_) *δ* 170.3 (br), 165.8 (br), 160.5, 135.4, 134.6, 134.4, 133.8, 132.9 (2C), 129.0, 128.3, 128.1, 127.6, 127.1, 124.9, 124.6, 123.7, 117.0 (br), 116.5 (2C) ppm. IR υ_max_ (cm^−1^): 3140, 3043, 2784, 1697, 1564, 1511, 1127. Mass spectral analysis: LRMS (ESI‐) *m/z*: 409 (M‐H, C_20_H_13_N_2_O_4_S_2_, 100%); (ESI+) *m/z*: 411 (M + H, C_20_H_15_N_2_O_4_S_2_, 100%).

##### N‐[(5Z)‐5‐(3‐hydroxybenzylidene)‐4‐oxo‐4,5‐dihydro‐1,3‐thiazol‐2‐yl]naphthalene‐1‐sulfonamide 7

Synthesized using general procedure 2 with **5** (150 mg, 0.49 mmol, 1 eq.) and 3‐hydroxybenzaldehyde (68 mg, 0.56 mmol, 1.1 eq.) for 30 min. Addition of hexane (≈2 mL) allowed precipitation to give the desired product as a yellow solid (154 mg, 77%). M.P: >243 °C (dec). *R*_f_ 0.16 (5% MeOH in CH_2_Cl_2_).

^1^H NMR (400 MHz, DMSO‐*d*_*6*_) *δ* 13.22 (s, 1H, NH), 9.99 (s, 1H, OH), 8.60 (d, *J* = 8.5 Hz, 1H), 8.30 (d, *J* = 7.7 Hz, 2H), 8.12 (d, *J* = 8.1 Hz, 1H), 7.77 (t, *J* = 7.3 Hz, 1H), 7.73–7.67 (m, 3H), 7.38 (t, *J* = 7.9 Hz, 1H), 7.11 (d, *J* = 7.8 Hz, 1H), 7.07 (s, 1H), 6.93 (d, *J* = 8.0 Hz, 1H) ppm. ^13^C NMR (151 MHz, DMSO‐*d*_6_) *δ* 166.7 (br), 165.8, 158.0, 135.3, 134.8, 134.01, 133.95, 133.8, 130.6, 129.1, 128.4, 128.1, 127.7, 127.2, 124.9, 124.7, 121.9, 121.6 (br), 118.3, 115.9 ppm. IR υ_max_ (cm^−1^): 3386, 3062, 2939, 1713, 1595, 1559, 1563, 1124. Mass spectral analysis: LRMS (ESI‐) *m/z*: 409 (M‐H, C_20_H_13_N_2_O_4_S_2_, 100%); (ESI+) *m/z*: 411 (M + H, C_20_H_15_N_2_O_4_S_2_, 100%).

##### (Z)‐N‐(5‐(2‐hydroxybenzylidene)‐4‐oxo‐4,5‐dihydrothiazol‐2‐yl)naphthalene‐1‐sulfonamide 8

Synthesized using general procedure 2 with **5** (148 mg, 0.49 mmol, 1 eq.) and 2‐hydroxybenzaldehyde (0.08 mL, 0.54 mmol, 1.1 eq.) for 1 h. Addition of H_2_O (≈2 mL) allowed precipitation to give the desired product as a yellow solid (69 mg, 35%). M.P: >218 °C (dec).

^1^H NMR (400 MHz, DMSO‐*d*_6_) *δ* 13.10 (br, s, 1H, NH), 10.69 (s, 1H, OH), 8.60 (d, *J* = 8.4 Hz, 1H), 8.31–8.23 (m, 2H), 8.12 (d, *J* = 8.0 Hz, 1H), 7.95 (s, 1H), 7.77 (t, *J* = 7.1 Hz, 1H), 7.72 – 7.67 (m, 2H), 7.44 (d, *J* = 7.8 Hz, 1H), 7.39–7.34 (m, 1H), 7.04 – 6.98 (m, 2H) ppm. ^13^C DEPTQ NMR (151 MHz, DMSO‐*d*_6_) *δ* 166.7, 166.0, 157.4, 135.4, 134.7, 133.8, 133.0, 129.5, 129.3, 129.0, 128.3, 128.1, 127.7, 127.1, 124.9, 124.7, 120.1, 119.9, 119.7, 116.3 ppm. IR υ_max_ (cm^−1^): 3373, 3210, 2961, 1708, 1555, 1355, 1126. Mass spectral analysis: LRMS (ESI‐) *m/z* (%): 409 (M‐H, C_20_H_13_N_2_O_4_S_2_, 100%). HRMS: Exact mass calculated for C_20_H_13_N_2_O_4_S_2_ [M‐H]^−^, 409.0300. Found 409.0321.

##### 4‐[(Z)‐{2‐[(naphthalen‐1‐ylsulfonyl)amino]‐4‐oxo‐1,3‐thiazol‐5(4H)‐ylidene}methyl]benzoic acid 9

Synthesized using general procedure 2 with **5** (150 mg, 0.49 mmol, 1 eq.) and 4‐carboxybenzaldehyde (82 mg, 0.55 mmol, 1.1 eq.) for 1 h to give the desired product as an off white solid (119 mg, 54%). M.P: >300 °C (dec). *R*_f_ 0.03 (10% MeOH in CH_2_Cl_2_).

^1^H NMR (400 MHz, DMSO‐*d*_6_) *δ* 13.26 (br, s, 1H, NH), 8.61 (d, *J* = 8.5 Hz, 1H), 8.31 (d, *J* = 7.2 Hz, 2H), 8.13–8.10 (m, 3H), 7.81–7.75 (m, 4H), 7.73 – 7.67 (m, 2H) ppm. OH exchanging—not observed. ^13^C NMR (151 MHz, DMSO‐*d*_6_) *δ* 166.6, 166.4, 165.4, 136.8, 135.2, 134.8, 133.8, 132.2, 132.1, 130.3 (2C), 130.2 (2C), 129.0, 128.4, 128.2, 127.6, 127.1, 124.8, 124.7, 124.2 ppm. IR υ_max_ (cm^−1^): 3289, 3023, 2519, 1726, 1561, 1309, 1280, 1116. Mass spectral analysis: LRMS (ESI‐) *m/z*: 437.0 (M‐H, C_21_H_14_N_2_O_5_S_2_, 100%).

##### (Z)‐3‐((2‐(naphthalene‐1‐sulfonamido)‐4‐oxothiazol‐5(4 H)‐ylidene)methyl)benzoic acid 10

Synthesized using general procedure 2 with **5** (151 mg, 0.49 mmol, 1 eq.) and 3‐carboxybenzaldehyde (87 mg, 0.58 mmol, 1.2 eq.) for 30 min. Addition of H_2_O (≈2 mL) allowed precipitation to give the desired product as a pale pink solid (179 mg, 82%). M.P: >162 °C (dec). *R*_f_ 0.08 (10% MeOH in CH_2_Cl_2_).

^1^H NMR (400 MHz, DMSO‐*d*_6_) *δ* 13.36 (broad s, 1H, NH), 8.60 (d, *J* = 8.6 Hz, 1H), 8.32 – 8.28 (m, 2H), 8.22 (s, 1H), 8.12 (d, *J* = 8.1 Hz, 1H), 8.06 (d, *J* = 7.8 Hz, 1H), 7.92 (d, *J* = 7.7 Hz, 1H), 7.85 (s, 1H), 7.79–7.67 (m, 4H) ppm. OH exchanging—not observed. ^13^C NMR (151 MHz, DMSO‐*d*_6_) *δ* 166.6 (2C), 165.4, 135.2, 134.8, 134.5, 133.8, 133.3, 132.6, 131.9, 131.2, 130.3, 129.9, 129.0, 128.4, 128.2, 127.6, 127.1, 124.8, 124.6, 123.2 ppm. IR υ_max_ (cm^−1^): 3289, 3023, 2519, 1726, 1679, 1561, 1116. Mass spectral analysis: LRMS (ESI) *m/z* (%): 437 (M‐H, C_21_H_14_N_2_O_2_S_2_).

##### (Z)‐N‐(5‐(4‐cyanobenzylidene)‐4‐oxo‐4,5‐dihydrothiazol‐2‐yl)naphthalene‐1‐sulfonamide 11

Synthesized using general procedure 2 with **5** (154 mg, 0.49 mmol, 1 eq.) and 4‐cyanobenzaldehyde (80 mg, 0.55 mmol, 1.1 eq.) 30 min to give the desired product as a pale orange solid (143 mg, 70%). M.P: >296 °C (dec).

^1^H NMR (400 MHz, DMSO‐*d*_6_) *δ* 8.61 (d, *J* = 8.6 Hz, 1H), 8.30 (d, *J* = 7.6 Hz, 2H), 8.12 (d, *J* = 7.9 Hz, 1H), 8.04 (d, *J* = 8.4 Hz, 2H), 7.85 – 7.82 (m, 3H), 7.77 (ddd, *J* = 8.5, 6.9, 1.4 Hz, 1H), 7.73 – 7.67 (m, 2H) ppm. NH exchanging—not observed. ^13^C NMR (101 MHz, DMSO‐*d*_6_) *δ* 166.5, 165.2, 137.3, 135.1, 134.8, 133.8, 133.2 (2C), 131.2, 130.6 (2C), 129.0, 128.4, 128.2, 127.6, 127.1, 125.6, 124.8, 124.7, 118.4, 112.3 ppm. IR υ_max_ (cm^−1^): 3166, 3049, 2965, 2215, 1711, 1553, 1348, 1131. Mass spectral analysis: LRMS (ESI‐) *m/z* (%): 418 (M‐H, C_21_H_13_N_3_O_3_S_2_, 100%). HRMS: Exact mass calculated for C_21_H_13_N_3_O_3_S_2_ [M‐H]^−^, 418.0300. Found 418.0325.

##### (Z)‐N‐(5‐(3‐cyanobenzylidene)‐4‐oxo‐4,5‐dihydrothiazol‐2‐yl)naphthalene‐1‐sulfonamide 12

Synthesized using general procedure 2 with **5** (152 mg, 0.49 mmol, 1 eq.) and 3‐cyanobenzaldehyde (77 mg, 0.55 mmol, 1.1 eq.) for 30 min to give the desired product as a peach colored solid (96 mg, 47%). M.P: >248 °C (dec).

^1^H NMR (400 MHz, DMSO‐*d*_6_) *δ* 8.60 (d, *J* = 8.6 Hz, 1H), 8.31 (d, *J* = 7.7 Hz, 2H), 8.14 – 8.11 (m, 2H), 7.97 (t, *J* = 7.4 Hz, 2H), 7.84 – 7.75 (m, 3H), 7.70 (td, *J* = 7.4, 5.0 Hz, 2H) ppm. NH exchanging—not observed. ^13^C NMR (101 MHz, DMSO‐*d*_6_) *δ* 166.6 (br), 165.3, 155.2 (br), 135.2, 134.8, 134.3, 134.2, 133.8, 133.7, 133.4, 131.1, 130.7, 129.0, 128.3, 128.2, 127.6, 127.1, 124.8, 124.7, 118.2, 112.6 ppm. IR υ_max_ (cm^−1^): 2965, 2816, 2234, 1708, 1564, 1121. Mass spectral analysis: LRMS (ESI‐) *m/z* (%): 418 (M‐H, C_21_H_12_N_3_O_3_S_2_, 100%); (ESI‐) *m/z* (%): 420 (M + H, C_21_H_14_N_3_O_3_S_2_, 100%).

##### (Z)‐N‐(5‐(4‐nitrobenzylidene)‐4‐oxo‐4,5‐dihydrothiazol‐2‐yl)naphthalene‐1‐sulfonamide 13

Synthesized using general procedure 2 with **5** (148 mg, 0.49 mmol, 1 eq.) and 4‐nitrobenzaldehyde (90 mg, 0.60 mmol, 1.2 eq.), for 20 min to give an off white solid (158 mg, 73%). M.P: >258.3 °C (dec). *R*_f_ 0.18 (10% MeOH in CH_2_Cl_2_).

^1^H NMR (400 MHz, DMSO‐*d*_6_) *δ* 8.62 (d, *J* = 8.6 Hz, 1H), 8.39 (d, *J* = 8.8 Hz, 2H), 8.28 (dd, *J* = 7.8, 3.3 Hz, 2H), 8.11 (d, *J* = 8.0 Hz, 1H), 7.90 (d, *J* = 8.8 Hz, 2H), 7.79 (s, 1H), 7.75 (ddd, *J* = 8.5, 7.0, 1.3 Hz, 1H), 7.71–7.67 (m, 2H) ppm. ^13^C NMR (101 MHz, DMSO‐*d*_6_) *δ* 147.5, 139.6, 135.8, 134.4, 133.8, 131.0 (2C), 129.5 (broad), 128.9, 128.1, 128.0, 127.7, 127.0, 125.0, 124.6, 124.4 (2C) ppm. Three carbons not observed. IR υ_max_ (cm^−1^): 3061, 2928, 2776, 1712, 1554, 1516, 1506, 1343, 1130. Mass spectral analysis: LRMS (ESI‐) m/z (%): 438 (M‐H, C_20_H_12_N_3_O_5_S_2_, 100%).

##### (Z)‐N‐(5‐(3‐nitrobenzylidene)‐4‐oxo‐4,5‐dihydrothiazol‐2‐yl)naphthalene‐1‐sulfonamide 14

Synthesized using general procedure 2 with **5** (148 mg, 0.49 mmol, 1 eq.) and 3‐nitrobenzaldehyde (86 mg, 0.55 mmol, 1.1 eq.) for 30 min. Addition of hexane (≈2 mL) allowed precipitation to give the desired product as an off white solid (87 mg, 40%).M.P: >243 °C (dec). *R*_f_ 0.21 (10% MeOH in CH_2_Cl_2_).

^1^H NMR (400 MHz, DMSO‐*d*_6_) *δ* 8.61 (d, *J* = 8.6 Hz, 1H), 8.53 (s, 1H), 8.31 – 8.29 (m, 3H), 8.11 (dd, *J* = 14.3, 7.9 Hz, 2H), 7.93 (s, 1 H), 7.89 (t, *J* = 8.0 Hz, 1H), 7.77 (t, *J* = 7.2 Hz, 1H), 7.73 – 7.68 (m, 2H) ppm. NH exchanging—not observed. ^13^C NMR (151 MHz, DMSO‐*d*_6_) *δ* 166.4, 165.1, 148.3, 135.5, 135.1, 134.9, 134.6, 133.8, 131.2, 131.1, 129.0, 128.4, 128.2, 127.6, 127.1, 124.9 (br), 124.8 (2C), 124.74, 124.66 ppm. IR υ_max_ (cm^−1^): 2979, 2763, 1728, 1569, 1528, 1354, 1126. Mass spectral analysis: LRMS (ESI+) *m/z*: 440 (M + H, C_20_H_14_N_3_O_5_S_2_, 100%); (ESI‐) *m/z*: 438 (M‐H, C_20_H_12_N_3_O_5_S_2_, 100%).

##### (Z)‐N‐(5‐(2‐nitrobenzylidene)‐4‐oxo‐4,5‐dihydrothiazol‐2‐yl)naphthalene‐1‐sulfonamide 15

Synthesized using general procedure 2 with **5** (146 mg, 0.48 mmol, 1 eq.) and 2‐nitrobenzaldehyde (83 mg, 0.54 mmol, 1.1 eq.) for 30 min. Addition of hexane (≈2 mL) allowed precipitation to give the desired product as a pale pink solid (100 mg, 48%). M.P: >245 °C (dec). *R*_f_ 0.26 (10% MeOH in CH_2_Cl_2_).

^1^H NMR (400 MHz, DMSO‐*d*_6_) *δ* 8.58 (d, *J* = 8.5 Hz, 1H), 8.29 (d, *J* = 8.2 Hz, 1H), 8.24 (dd, *J* = 7.7, 2.6 Hz, 2H), 8.12 (d, *J* = 8.0 Hz, 1H), 8.02 (s, 1H), 7.98 (t, *J* = 7.5 Hz, 1H), 7.78 (dd, *J* = 15.1, 7.9 Hz, 3H), 7.71–7.66 (m, 2H) ppm. NH exchanging—not observed. ^13^C NMR (101 MHz, DMSO‐*d*_6_) *δ*165.9, 165.6, 147.8, 135.1, 134.8 (2C), 133.8, 131.3, 130.5, 129.6, 129.0, 128.9, 128.3, 128.1, 127.6, 127.1, 126.5, 125.6, 124.8, 124.6 ppm. IR υ_max_ (cm^−1^): 3055, 2941, 2788, 1714, 1558, 1517, 1340, 1159, 1131. LRMS (ESI+) *m/z*: 440 (M + H, C_20_H_14_N_3_O_5_S_2_, 100%); (ESI+) *m/z*: 438 (M + H, C_20_H_12_N_3_O_5_S_2_, 100%).

##### N‐(5‐([1,1'‐biphenyl]‐2‐ylmethylene)‐4‐oxo‐4,5‐dihydrothiazol‐2‐yl)naphthalene‐1‐sulfonamide 16

Synthesized using general procedure 2 with **5** (150 mg, 0.49 mmol) and 2‐biphenylcarboxaldehyde (0.1 mL, 0.54 mmol, 1.1 eq) for 45 min to give the desired product as an off white solid (113 mg, 49%), MP 202–204 °C.

^1^H NMR (400 MHz, DMSO‐*d*_6_) *δ* 8.61 (d, *J* = 8.4 Hz, 1H), 8.29 (s, 2H), 8.12 (d, *J* = 7.8 Hz, 1H), 7.79 – 7.60 (m, 6H), 7.52 – 7.45 (m, 5H), 7.35 (d, *J* = 6.3 Hz, 2H), NH exchanging—not visible ethanol impurity at 1.06 ppm (4.84%); ^13^C NMR (101 MHz, DMSO‐*d*_6_) *δ* 166.2, 165.9, 143.3, 139.0, 135.2, 134.7, 133.8, 132.6, 130.8, 130.7 (2C), 129.7 (2C), 129.0, 128.5 (2C), 128.34, 128.31, 128.1, 128.0, 127.95, 127.6, 127.1, 124.9, 124.7, 123.4, 2C determined by 2D NMR. IR ν_max_ (cm^−1^) = 3455, 2980, 1711 (C=O), 1570, 1316, 1127 (C—N). LRMS (ESI^−^): 469 (M‐H, C_26_H_17_N_2_O_3_S_2_, 100).

##### N‐(5‐([1,1'‐biphenyl]‐3‐ylmethylene)‐4‐oxo‐4,5‐dihydrothiazol‐2‐yl)naphthalene‐1‐sulfonamide 17

Synthesized using general procedure 2 with **5** (148 mg, 0.49 mmol, 1 eq.) and 3‐biphenylcarboxaldehyde (0.09 mL, 0.54 mmol, 1.1 eq.) for 40 min to give the desired product as light pink/orange solid (156 mg, 40%). M.P: 201‐202 °C (dec).

^1^H NMR (400 MHz, DMSO‐*d*_6_) *δ* 8.61 (d, *J* = 8.5 Hz, 1H), 8.33 – 8.27 (m, 2H), 8.12 (d, *J* = 8.1 Hz, 1H), 7.96 (s, 1H), 7.87 (s, 1 H), 7.82 (d, *J* = 7.6 Hz, 1H), 7.79 – 7.64 (m, 7H), 7.53 (t, *J* = 7.6 Hz, 2H), 7.43 (t, *J* = 7.3 Hz, 1H) ppm, NH exchanging—not visible; ^13^C NMR (101 MHz, DMSO‐*d*_6_) *δ* 166.8 (br), 165.8 (br), 141.2, 139.1, 135.3, 134.7, 133.8, 133.64, 133.59, 130.1, 129.3, 129.2 (2C), 129.1, 129.0, 128.33, 128.27, 128.2, 128.1, 127.7, 127.1, 126.8 (2C), 124.9, 124.6, 122.5 ppm. IR υ_max_ (cm^−1^)= 3131, 2977, 2887, 1700, 1557, 1340, 1130. LRMS (ESI^+^) *m/z*: 469 (M‐H, C_26_H_17_N_2_O_3_S_2_, 100).

##### N‐(5‐([1,1'‐biphenyl]‐4‐ylmethylene)‐4‐oxo‐4,5‐dihydrothiazol‐2‐yl)naphthalene‐1‐sulfonamide 18

Synthesized using general procedure 2 with **5** (149 mg, 0.49 mmol, 1 eq.) and biphenyl‐4‐carboxaldehyde (98 mg, 0.54 mmol, 1.1 eq.) for 30 min to give the desired product as a yellow solid (189 mg, 82%). M.P: >300 °C (dec). *R*_f_ 0.25 (10% MeOH in CH_2_Cl_2_).

^1^H NMR (400 MHz, DMSO‐*d*_6_) *δ* 8.63 (d, *J* = 8.5 Hz, 1H), 8.31 (t, *J* = 8.8 Hz, 2H), 8.12 (d, *J* = 8.1 Hz, 1H), 7.89 (d, *J* = 8.2 Hz, 2H), 7.79 ‐ 7.67 (m, 8H), 7.51 (t, *J* = 7.5 Hz, 2H), 7.44 – 7.41 (m, 1H) ppm. NH exchanging—not observed. ^13^C NMR (151 MHz, DMSO‐*d*_6_) *δ* 142.1, 138.8, 135.5 (br), 134.6, 133.8, 132.7 (br), 132.0, 130.9 (2C), 129.1 (2C), 129.0, 128.32, 128.26, 128.1, 127.7, 127.6, 127.1 (2C), 126.9 (3C), 125.0, 124.7 ppm. Two quaternary carbons not observed. IR υ_max_ (cm^−1^): 3143, 2785, 2928, 1713, 1570, 1141. Mass spectral analysis: LRMS (ESI‐) *m/z*: 487 (M‐H+H_2_O, C_26_H_18_N_2_O_3_S_2_, 100%), 469 (M‐H, C_26_H_18_N_2_O_3_S_2_, 100%).

##### (Z)‐N‐(4‐((2‐(naphthalene‐1‐sulfonamido)‐4‐oxothiazol‐5(4H)‐ylidene)methyl)phenyl)acetamide 19

Synthesized using general procedure 2 with **5** (200 mg, 0.65 mmol, 1 eq.) and 4‐acetamidobenzaldehyde (118 mg, 0.72 mmol, 1.1 eq.) for 30 min to give the desired product as yellow solid (144 mg, 46%). M.P: >291 °C (dec).

^1^H NMR (400 MHz, DMSO‐*d*_6_) *δ*13.13 (br, s, 1H, NH), 10.31 (s, 1H, OH), 8.62 (d, *J* = 8.6 Hz, 1H), 8.30 (dd, *J* = 12.1, 7.8 Hz, 2H), 8.11 (d, *J* = 8.1 Hz, 1H), 7.79 (d, *J* = 8.6 Hz, 2H), 7.76 – 7.66 (m, 4H), 7.61 (d, *J* = 8.6 Hz, 2H), 2.09 (s, 3H) ppm. ^13^C NMR (151 MHz, DMSO‐*d*_6_) *δ* 168.9, 141.6, 135.6 (br), 134.5, 133.8, 133.0 (br), 131.5 (2C), 129.0, 128.2, 128.1, 127.7, 127.4, 127.0, 125.0, 124.6, 119.24 (2C), 24.2 ppm. Three quaternary carbons not observed. IR υ_max_ (cm^−1^): 2765, 1732, 1668, 1578, 1321, 1180. Mass spectral analysis: LRMS (ESI‐) *m/z* (%): 450 (M‐H, C_22_H_16_N_3_O_4_S_2_, 100%).

##### (Z)‐N‐(5‐(4‐(tert‐butyl)benzylidene)‐4‐oxo‐4,5‐dihydrothiazol‐2‐yl)naphthalene‐1‐sulfonamide 20

Synthesized using general procedure 2 with **5** (153 mg, 0.49 mmol, 1 eq.) and 4‐(*tert*‐butyl)benzaldehyde (0.1 mL, 0.54 mmol, 1.1 eq.) for 30 min to give the desired product as a pale yellow solid (106 mg, 48%). M.P: 261–262 °C.

^1^H NMR (400 MHz, DMSO‐*d*_6_) *δ* 8.61 (d, *J* = 8.6 Hz, 1H), 8.31 (dd, *J* = 7.8, 3.0 Hz, 2H), 8.12 (d, *J* = 8.0 Hz, 1H), 7.79 – 7.75 (m, 2H), 7.73 – 7.67 (m, 2H), 7.61 (s, 4H), 1.31 (s, 9H) ppm. NH exchanging—not observed. ^13^C NMR (101 MHz, DMSO‐*d*_6_) *δ* 166.5, 165.7, 154.1, 135.3, 134.7, 133.8, 133.7, 130.2 (2C), 130.1, 129.0, 128.3, 128.1, 127.6, 127.1, 126.4 (2C), 124.9, 124.6, 120.8, 34.8, 30.8 (3C) ppm. IR υ_max_ (cm^−1^): 3127, 3062, 2959, 1707, 1545, 1327, 1130. Mass spectral analysis: LRMS (ESI‐) *m/z* (%): 449 (M‐H, C_24_H_21_N_2_O_3_S_2_, 100%); (ESI+) *m/z* (%): 451 (*M*+H, C_24_H_23_N_2_O_3_S_2_, 100%). HRMS: Exact mass calculated for C_24_H_21_N_2_O_3_S_2_ [M‐H]^−^, 449.1000. Found 449.0998.

##### (Z)‐N‐(5‐(4‐isopropylbenzylidene)‐4‐oxo‐4,5‐dihydrothiazol‐2‐yl)naphthalene‐1‐sulfonamide 21

Synthesized using general procedure 2 with **5** (147 mg, 0.49 mmol, 1 eq.) and 4‐isopropylbenzaldehyde (0.08 mL, 0.54 mmol, 1.1 eq.) for 30 min to give the desired product as a pale yellow solid (107 mg, 50%). M.P: 227–228 °C.

^1^H NMR (400 MHz, DMSO‐*d*_6_) *δ* 8.61 (d, *J* = 8.7 Hz, 1 H), 8.32 – 8.29 (m, 2H), 8.12 (d, *J* = 8.0 Hz, 1H), 7.79–7.74 (m, 2H), 7.73–7.67 (m, 2H), 7.60 (d, *J* = 8.3 Hz, 2H), 7.47 (d, *J* = 8.3 Hz, 2H), 3.02 – 2.91 (m, 1H), 1.24 (s, 3H), 1.22 (s, 3H) ppm. NH exchanging—not observed.^13^C NMR (101 MHz, DMSO‐*d*_6_) *δ* 166.5, 165.7, 151.9, 135.3, 134.7, 133.83, 133.80, 130.51 (2C), 130.49, 129.0, 128.3, 128.1, 127.6, 127.5 (2C), 127.1, 124.9, 124.7, 120.6, 33.5, 23.5 (2C) ppm. IR υ_max_ (cm^−1^): 3153, 3056, 2972, 1703, 1539, 1327, 1129. Mass spectral analysis: LRMS (ESI‐) *m/z* (%): 435 (M‐H, C_23_H_19_N_2_O_3_S_2_, 100%); (ESI+) *m/z* (%): 437 (M + H, C_23_H_21_N_2_O_3_S_2_, 100%). HRMS: Exact mass calculated for C_23_H_19_N_2_O_3_S_2_ [M‐H]^−^, 435.0800. Found 435.0840.

##### (Z)‐N‐(4‐oxo‐5‐(4‐propylbenzylidene)‐4,5‐dihydrothiazol‐2‐yl)naphthalene‐1‐sulfonamide 22

Synthesized using General Procedure 2 with **5** (113 mg, 0.33 mmol, 1 eq.) and 4‐propylbenzaldehyde (0.05 mL, 0.36 mmol, 1.1 eq.) for 30 min to give the desired product as a pale orange solid (71 mg, 49%). M.P: 209–211 °C.

^1^H NMR (400 MHz, DMSO‐*d*_6_) *δ* 8.62 (d, *J* = 8.7 Hz, 1H), 8.31 (dt, *J* = 8.0, 4.0 Hz, 2H), 8.12 (d, *J* = 8.1 Hz, 1H), 7.77 (ddd, *J* = 8.5, 6.9, 1.4 Hz, 1H), 7.73 – 7.67 (m, 3H), 7.57 (d, *J* = 8.2 Hz, 2H), 7.39 (d, *J* = 8.2 Hz, 2H), 2.63 – 2.59 (m, 2H), 1.65 – 1.56 (m, 2H), 0.90 (t, *J* = 7.3 Hz, 3H) ppm. NH exchanging—not observed. ^13^C NMR (101 MHz, DMSO‐*d*_6_) *δ* 166.6, 165.7, 145.8, 135.3, 134.7, 133.8 (2C), 130.39 (2C), 130.35, 129.5 (2C), 129.0, 128.3, 128.1, 127.6, 127.1, 124.9, 124.6, 120.6, 37.1, 23.7, 13.6 ppm. 2C at 133.8 determined by DEPTQ. IR υ_max_ (cm^−1^): 2972, 2797, 1701, 1551, 1328, 1130. Mass spectral analysis: LRMS (ESI‐) *m/z* (%): 435 (M‐H, C_23_H_19_N_2_O_3_S_2_, 100%); (ESI+) *m/z* (%): 437 (M + H, C_23_H_21_N_2_O_3_S_2_, 100%). HRMS: Exact mass calculated for C_23_H_19_N_2_O_3_S_2_ [M‐H]^−^, 435.0800. Found 435.0840.

##### (Z)‐N‐(5‐(4‐butylbenzylidene)‐4‐oxo‐4,5‐dihydrothiazol‐2‐yl)naphthalene‐1‐sulfonamide 23

Synthesized using general procedure 2 with **5** (108 mg, 0.33 mmol, 1 eq.) and 4‐propylbenzaldehyde (0.06 mL, 0.36 mmol, 1.1 eq.) for 30 min to give the desired product as an off white solid (106 mg, 71%). M.P: 214–216 °C.

^1^H NMR (400 MHz, DMSO‐*d*_6_) *δ* 13.13 (s, 1H, NH), 8.62 (d, *J* = 8.6 Hz, 1H), 8.31 (t, *J* = 7.9 Hz, 2H), 8.12 (d, *J* = 8.1 Hz, 1H), 7.77 (t, *J* = 7.6 Hz, 1H), 7.70 (dd, *J* = 15.1, 7.3 Hz, 3H), 7.56 (d, *J* = 8.0 Hz, 2H), 7.38 (d, *J* = 8.0 Hz, 2H), 2.63 (t, *J* = 7.6 Hz, 2H), 1.60–1.52 (m, 2H), 1.35–1.26 (m, 2H), 0.90 (t, *J* = 7.3 Hz, 3H) ppm. ^13^C NMR (101 MHz, DMSO‐*d*_6_) *δ* 166.6, 165.7, 146.0, 135.3, 134.7, 133.84, 133.80, 130.4 (2C), 130.3, 129.4 (2C), 129.0, 128.3, 128.1, 127.6, 127.1, 124.8, 124.6, 120.5, 34.8, 32.7, 21.7, 13.7 ppm. IR υ_max_ (cm^−1^): 3120, 2933, 2784, 1729, 1554, 1324, 1123. Mass spectral analysis: LRMS (ESI‐) *m/z* (%): 449 (M‐H, C_24_H_2_N_2_O_3_S_2_, 100); LRMS (ESI+) *m/z* (%): 451 (M + H, C_24_H_23_N_2_O_3_S_2_, 100). HRMS: Exact mass calculated for C_24_H_20_N_2_O_3_S_2_ [M‐H]^−^, 449.1000. Found 449.0998.

##### Library 2: (Z)‐N‐(5‐(2,3‐dihydroxybenzylidene)‐4‐oxo‐4,5‐dihydrothiazol‐2‐yl)naphthalene‐1‐sulfonamide 24

Synthesized using general procedure 2 with **5** (146 mg, 0.49 mmol, 1 eq.) and 2,3‐dihydroxybenzaldehyde (96 mg, 0.54 mmol, 1.1 eq.) for 30 min. Addition of H_2_O (≈2 mL) allowed precipitation to give the desired product as a yellow solid (53 mg, 25%). M.P: >158 °C (dec).

^1^H NMR (600 MHz, DMSO ‐*d*_6_) *δ* 13.08 (br, s, 1H, NH), 9.90 (s, 1H, OH), 9.56 (s, 1H, OH), 8.60 (d, *J* = 8.6 Hz, 1H), 8.29 (d, *J* = 7.5 Hz, 2H), 8.12 (d, *J* = 8.1 Hz, 1 H), 8.00 (s, 1H), 7.76 (t, *J* = 7.7 Hz, 1H), 7.71 – 7.67 (m, 2H), 6.96–6.92 (m, 2H), 6.88–6.85 (m, 1H) ppm. ^13^C DMPTQ NMR (151 MHz, DMSO‐*d*_6_) *δ* 166.6, 166.0, 146.3, 146.0, 135.3, 134.7, 133.8, 129.7, 129.0, 128.3, 128.1, 127.6, 127.1, 124.9, 124.7, 120.4, 119.9 (br), 119.8, 118.9, 118.0 ppm. IR υ_max_ (cm^−1^): 3441, 2983, 2887, 2745, 1694, 1554, 1349, 1275, 1120. Mass spectral analysis: LRMS (ESI‐) *m/z*: 425 (M‐H, C_20_H_13_N_2_O_5_S_2_, 100). HRMS: Exact mass calculated for C_20_H_13_N_2_O_5_S_2_ [M‐H]^−^, 425.0300. Found 425.0274.

##### (Z)‐N‐(5‐(2,4‐dihydroxybenzylidene)‐4‐oxo‐4,5‐dihydrothiazol‐2‐yl)naphthalene‐1‐sulfonamide 25

Synthesized using general procedure 2 with **5** (152 mg, 0.49 mmol, 1 eq.) and 2,4‐dihydroxybenzaldehyde (161 mg, 1.17 mmol, 1.1 eq.) for 60 min. Addition of H_2_O (≈2 mL) allowed precipitation to give the desired product as a bright yellow solid (123 mg, 59%). M.P: >236 °C (dec).

^1^H NMR (600 MHz, DMSO‐*d*_6_) *δ* 12.92 (br, s, 1H, NH), 10.67 (s, 1H, OH), 10.33 (s, 1H, OH), 8.60 (d, *J* = 8.6 Hz, 1H), 8.29 (d, *J* = 7.4 Hz, 2H), 8.11 (d, *J* = 8.1 Hz, 1H), 7.92 (s, 1H), 7.76 (t, *J* = 7.6 Hz, 1H), 7.71 – 7.67 (m, 2H), 7.30 (d, *J* = 8.6 Hz, 1H), 6.49 (d, *J* = 8.6 Hz, 1H), 6.44 (s, 1H) ppm. ^13^C NMR (151 MHz, DMSO‐*d*_6_) *δ* 166.8, 166.1, 162.5, 159.7, 135.5, 134.6, 133.8, 131.1, 130.1, 129.0, 128.2, 128.0, 127.7, 127.1, 124.9, 124.6, 114.7, 111.5, 108.7, 102.6 ppm. IR υ_max_ (cm^−1^): 3352, 2983, 2893, 2786, 1685, 1542, 1211, 1126. Mass spectral analysis: LRMS (ESI‐) *m/z*: 425 (M‐H, C_20_H_13_N_2_O_5_S_2_, 100).

##### (Z)‐N‐(5‐(2,5‐dihydroxybenzylidene)‐4‐oxo‐4,5‐dihydrothiazol‐2‐yl)naphthalene‐1‐sulfonamide 26

Synthesized using general procedure 2 with **5** (152 mg, 0.49 mmol, 1 eq.) and 2,5‐dihydroxybenzaldehyde (75 mg, 0.54 mmol, 1.1 eq.) for 30 min. Addition of H_2_O (≈2 mL) allowed precipitation to give the desired product as a bright yellow solid (86 mg, 44%). M.P: >225 °C (dec).

^1^H NMR (600 MHz, DMSO‐*d*_6_) *δ* 13.09 (br, s, 1H, NH), 9.95 (s, 1H, OH), 9.34 (s, 1H, OH), 8.60 (d, *J* = 8.6 Hz, 1H), 8.31 – 8.29 (m, 2H), 8.12 (d, *J* =  8.1 Hz, 1H), 7.93 (s, 1H), 7.77 (t, *J* = 7.7 Hz, 1H), 7.72 – 7.68 (m, 2H), 6.89 (s, 1H), 6.82 (s, 2H) ppm. ^13^C NMR (151 MHz, DMSO‐*d*_6_) *δ* 166.8 (br), 166.0 (br), 150.7, 150.2, 135.4, 134.7, 133.8, 129.2, 129.0, 128.3, 128.0, 127.6, 127.1, 124.9, 124.6, 120.7, 119.6, 119.5 (br), 117.3, 113.5 ppm. IR υ_max_ (cm^−1^): 3518, 3381, 3167, 2977, 2887, 1706, 1561, 1306, 1158, 1121. Mass spectral analysis: LRMS (ESI‐) *m/z*: 425 (M‐H, C_20_H_13_N_2_O_5_S_2_, 100).

##### (Z)‐N‐(5‐(3,4‐dihydroxybenzylidene)‐4‐oxo‐4,5‐dihydrothiazol‐2‐yl)naphthalene‐1‐sulfonamide 27

Synthesized using general procedure 2 with **5** (150 mg, 0.49 mmol, 1 eq.) and 3,4‐dihydroxybenzaldehyde (102 mg, 0.734 mmol, 1.1 eq.) for 60 min to give the desired product as a yellow/orange solid (175 mg, 84%). M.P: 247–249 °C.

^1^H NMR (400 MHz, DMSO‐*d*_6_) *δ* 13.03 (s, 1H, NH), 9.97 (s, 1H, OH), 9.69 (s, 1H, OH), 8.61 (d, *J* = 8.6 Hz, 1H), 8.32–8.28 (m, 2H), 8.12 (d, *J* = 8.1 Hz, 1H), 7.77 (t, *J* = 7.3 Hz, 1H), 7.73–7.67 (m, 2H), 7.60 (s, 1H), 7.11 (d, *J* = 1.9 Hz, 1H), 7.05 (dd, *J* = 8.3, 1.9 Hz, 1H), 6.92 (d, *J* = 8.2 Hz, 1H) ppm. ^13^C NMR (101 MHz, DMSO‐*d*_6_) *δ* 166.7, 165.9, 149.4, 146.1, 135.4, 134.9, 134.7, 133.8, 129.0, 128.3, 128.0, 127.7, 127.1, 125.0, 124.9, 124.6, 124.1, 116.7, 116.4, 116.3 ppm. IR υ_max_ (cm^−1^): 3468, 3356, 2974, 2768, 1707, 1559, 1292, 1157. Mass spectral analysis: LRMS (ESI‐): 425 (M‐H, C_20_H_13_N_2_O_5_S_2_, 100).

##### (Z)‐N‐(4‐oxo‐5‐(2,3,4‐trihydroxybenzylidene)‐4,5‐dihydrothiazol‐2‐yl)naphthalene‐1‐sulfonamide 28

Synthesized using general procedure 2 with **5** (155 mg, 0.49 mmol, 1 eq.) and 2,3,4‐trihydroxybenzaldehyde (117 mg, 0.54 mmol, 1.1 eq.) for 60 min. Addition of H_2_O (≈2 mL) allowed precipitation to give the desired product as a dark yellow solid (100 mg, 46%). M.P: >199 °C (dec).

^1^H NMR (400 MHz, DMSO‐*d*_6_) *δ* 10.21 (s, 1H, OH), 9.58 (s, 1H, OH), 8.80 (s, 1H, OH), 8.60 (d, *J* = 8.5 Hz, 1H), 8.30 – 8.27 (m, 2H), 8.11 (d, *J* = 8.0 Hz, 1H), 7.96 (s, 1H), 7.76 (t, *J* = 7.3 Hz, 1H), 7.72 – 7.66 (m, 2H), 6.85 (d, *J* = 8.7 Hz, 1H), 6.58 (d, *J* = 8.7 Hz, 1H) ppm. NH exchanging—not observed. ^13^C NMR (101 MHz, DMSO‐*d*_6_) *δ* 167.0 (br), 166.4 (br), 150.2, 148.0, 135.6, 134.5, 133.8, 133.2, 130.3 (br), 129.0, 128.2, 128.0, 127.7, 127.1, 124.9, 124.6, 120.2, 115.4 (br), 112.7, 108.4 ppm. IR υ_max_ (cm^−1^): 3423, 2977, 2881, 2774, 1688, 1556, 1333, 1223, 1122. Mass spectral analysis: LRMS (ESI‐) *m/z*: 441 (M‐H, C_20_H_13_N_2_O_6_S_2_, 100), 459 (M‐H + H_2_O, C_20_H_15_N_2_O_7_S_2_, 100).

##### (Z)‐N‐(5‐(2,3‐dimethoxybenzylidene)‐4‐oxo‐4,5‐dihydrothiazol‐2‐yl)naphthalene‐1‐sulfonamide 29

Synthesized using general procedure 2 with **5** (156 mg, 0.49 mmol, 1 eq.) and 2,3‐dimethoxybenzaldehyde (101 mg, 0.54 mmol, 1.1 eq.) for 30 min to give the desired product as a yellow solid (188 mg, 84%). M.P: >263 °C (dec).

^1^H NMR (600 MHz, DMSO‐*d*_6_) *δ* 13.18 (br, s, 1H, NH), 8.62 (d, *J* = 8.6 Hz, 1H), 8.31 – 8.29 (m, 2H), 8.11 (d, *J* = 8.1 Hz, 1H), 7.86 (s, 1H), 7.77 (t, *J* = 7.7 Hz, 1H), 7.72 – 7.67 (m, 2H), 7.29 (t, *J* = 7.9 Hz, 1H), 7.24 (d, *J* = 8.1 Hz, 1H), 7.13 (d, *J* = 7.6 Hz, 1H), 3.86 (s, 3H), 3.79 (s, 3H) ppm. ^13^C DEPTQ (151 MHz, DMSO‐*d*_6_) *δ* 166.5, 165.8, 152.8, 148.2, 135.2, 134.7, 133.8, 129.0, 128.4, 128.3, 128.1, 127.6, 127.1, 126.4, 124.92, 124.86, 124.6, 122.9, 120.3, 115.9, 61.2, 56.0 ppm. IR υ_max_ (cm^−1^): 3161, 3060, 2977, 2882, 2792, 1700, 1563, 1300, 1268, 1124. Mass spectral analysis: LRMS (ESI‐) *m/z*: 453 (M‐H, C_22_H_17_N_2_O_5_S_2_, 100); (ESI+) *m/z*: 455 (M + H, C_22_H_19_N_2_O_5_S_2_, 100).

##### (Z)‐N‐(5‐(2,4‐dimethoxybenzylidene)‐4‐oxo‐4,5‐dihydrothiazol‐2‐yl)naphthalene‐1‐sulfonamide 30

Synthesized using general procedure 2 with **5** (152 mg, 0.49 mmol, 1 eq.) and 2,4‐dimethoxybenzaldehyde (93 mg, 0.54 mmol, 1.1 eq.) for 30 min to give the desired product as a bright orange solid (185 mg, 83%). M.P: >255 °C (dec).

^1^H NMR (600 MHz, DMSO‐*d*_6_) *δ* 13.02 (br, s, 1H), 8.61 (d, *J* = 8.5 Hz), 8.32 – 8.29 (m, 2H), 8.12 (d, *J* = 8.0 Hz, 1H), 7.85 (s, 1H), 7.77 (t, *J* = 7.6 Hz, 1H), 7.73 – 7.67 (m, 2H), 7.44 (d, *J* 2= 8.6 Hz, 1H), 6.73 (d, *J* = 8.6 Hz, 1H), 6.69 (s, 1H), 3.90 (s, 3H), 3.84 (s, 3H) ppm. ^13^C NMR (151 MHz, DMSO‐*d*_6_) *δ* 166.6, 166.0, 163.7, 160.0, 135.4, 134.6, 133.8, 131.1, 129.0, 128.9, 128.3, 128.1, 127.7, 127.1, 124.9, 124.6, 117.8, 114.0, 106.8, 98.7, 56.0, 55.7 ppm. IR υ_max_ (cm^−1^): 2989, 2887, 2786, 1682, 1557, 1268, 1126. Mass spectral analysis: LRMS (ESI‐) *m/z*: 453 (M‐H, C_22_H_17_N_2_O_5_S_2_, 100); (ESI+) *m/z*: 455 (M + H, C_22_H_19_N_2_O_5_S_2_, 100).

##### (Z)‐N‐(5‐(3,4‐dimethoxybenzylidene)‐4‐oxo‐4,5‐dihydrothiazol‐2‐yl)naphthalene‐1‐sulfonamide 31

Synthesized using General Procedure 2 with **5** (143 mg, 0.49 mmol, 1 eq.) and 3,4‐dimethoxybenzaldehyde (94 mg, 0.54 mmol, 1.1 eq.) for 30 min to give the desired product as a bright orange/yellow solid (163 mg, 73%). M.P: >255 °C (dec).

^1^H NMR (600 MHz, DMSO‐*d*_6_) *δ* 13.10 (br, s, 1H, NH), 8.62 (d, *J* = 8.6 Hz, 1H), 8.31 (dd, *J* = 15.7, 7.8 Hz, 2H), 8.12 (d, *J* = 8.2 Hz, 1H), 7.77 (t, *J* = 7.7 Hz, 1H), 7.72 – 7.68 (m, 3H), 7.26 (s, 1H), 7.24 – 7.22 (m, 1H), 7.14 (d, *J* = 8.4 Hz, 1H), 3.83 (s, 6 H) ppm. ^13^C NMR (151 MHz, DMSO‐*d*_6_) *δ* 166.4, 165.7, 151.3, 148.0, 135.3, 134.7, 134.3, 133.8, 129.0, 128.3, 128.2, 127.7, 127.1, 125.5, 124.9, 124.6, 123.6, 118.5, 114.2, 112.2, 55.7, 55.6 ppm. IR υ_max_ (cm^−1^): 2996, 2905, 2768, 1694, 1506, 1300, 1126. Mass spectral analysis: LRMS (ESI‐) *m/z*: 453 (M‐H, C_22_H_17_N_2_O_5_S_2_, 100); (ESI+) *m/z*: 455 (M + H, C_22_H_19_N_2_O_5_S_2_, 100).

##### (Z)‐N‐(5‐(3,5‐dimethoxybenzylidene)‐4‐oxo‐4,5‐dihydrothiazol‐2‐yl)naphthalene‐1‐sulfonamide 32

Synthesized using General Procedure 2 with **5** (148 mg, 0.49 mmol, 1 eq.) and 3,5‐dimethoxybenzaldehyde (66 mg, 0.54 mmol, 1.1 eq.) for 30 min to give the desired product as a pale pink/orange solid (127 mg, 57%). M.P: 222–225 °C.

^1^H NMR (400 MHz, DMSO‐*d*_6_) *δ* 8.61 (d, *J* = 8.6 Hz, 1H), 8.30 (d, *J* = 7.7 Hz, 2H), 8.12 (d, *J* = 8.0 Hz, 1H), 7.79 – 7.75 (m, 1H), 7.72 – 7.67 (m, 3H), 6.80 (d, *J* = 2.0 Hz, 2H), 6.69 (t, *J* = 2.0 Hz, 1H), 3.82 (s, 6H) ppm. NH exchanging—not observed. ^13^C NMR (101 MHz, DMSO‐*d*_6_) *δ* 166.3, 165.5, 160.9 (2C), 135.2, 134.8, 134.7, 133.8, 133.7, 129.0, 128.3, 128.2, 127.6, 127.1, 124.9, 124.6, 122.5, 108.2 (2C), 102.4, 55.5 (2C) ppm. IR υ_max_ (cm^−1^): 3179, 2965, 1723, 1594, 1299, 1124. Mass spectral analysis: LRMS (ESI‐) *m/z* (%): 453 (M‐H, C_22_H_17_N_2_O_5_S_2_, 100%); (ESI+) *m/z* (%): 455 (M + H, C_22_H_19_N_2_O_5_S_2_, 100%).

##### (Z)‐N‐(4‐oxo‐5‐(3,4,5‐trimethoxybenzylidene)‐4,5‐dihydrothiazol‐2‐yl)naphthalene‐1‐sulfonamide 33

Synthesized using General Procedure 2 with **5** (144 mg, 0.49 mmol, 1 eq.) and 3,4,5‐trimethoxybenzaldehyde (114 mg, 0.54 mmol, 1.1 eq.) for 30 min to give the desired product as a bright yellow/orange solid (137 mg, 58%). M.P: >231 °C (dec).

^1^H NMR (600 MHz, DMSO‐*d*_6_) *δ* 8.61 (d, *J* =  8.6 Hz, 1H), 8.30 (t, *J* = 7.9 Hz, 2H), 8.11 (d, *J* = 8.1 Hz, 1H), 7.77 (t, *J* = 7.6 Hz, 1H), 7.71 – 7.67 (m, 3H), 6.97 (s, 2H), 3.86 (s, 6 H), 3.76 (s, 3H) ppm. NH exchanging—not observed. ^13^C NMR (151 MHz, DMSO‐*d*_6_) *δ* 165.8 (br, 2C), 153.3 (2C), 139.9, 135.2 (br), 134.7, 134.0 (br), 133.8, 129.0, 128.4, 128.34, 128.31, 127.7, 127.1, 124.9, 124.6, 120.9 (br), 107.9 (2C), 60.3, 56.1 (2C) ppm. IR υ_max_ (cm^−1^): 3060, 2983, 2893, 2786, 1706, 1559, 1232, 1125. Mass spectral analysis: LRMS (ESI‐) *m/z*: 483 (M‐H, C_23_H_19_N_2_O_6_S_2_, 100); (ESI+) *m/z*: 485 (M + H, C_23_H_21_N_2_O_6_S_2_, 100).

##### (Z)‐N‐(5‐(2,3‐dichlorobenzylidene)‐4‐oxo‐4,5‐dihydrothiazol‐2‐yl)naphthalene‐1‐sulfonamide 34

Synthesized using General Procedure 2 with **5** (149 mg, 0.49 mmol) and 2,3‐dichlorobenzaldehyde (137 mg, 0.74 mmol, 1.1 eq) for 1.5 h to give the desired product as an off white solid (127 mg, 56%), M P 232–235 °C.

^1^H NMR (400 MHz, DMSO‐d_6_) *δ* 8.59 (d, *J* = 8.5 Hz, 1H), 8.31 – 8.26 (m, 2H), 8.12 (d, *J* = 8.1 Hz, 1H), 7.85 (s, 1H), 7.82–7.75 (m, 2H), 7.71 – 7.61 (m, 4H). NH exchanging—not observed; ^13^C NMR (101 MHz, DMSO‐d_6_) *δ* 166.2, 165.3, 135.1, 134.8, 133.8, 133.5, 133.1, 132.2, 132.1, 129.2, 129.0, 128.4 (2C), 128.1, 127.8, 127.6, 127.2, 127.1, 124.8, 124.6; IR υ_max_ (cm^−1^) =3125, 3055, 3025, 2962, 2787, 1716, 1565, 1346, 1156, 1129, 767, 714. Mass Spectral analysis: LRMS (ESI‐) m/z (%): 461 (M‐H, C_20_H_11_^35^Cl_2_N_2_O_3_S_2_, 100), 463 (M‐H, C_20_H_11_^35^Cl^37^ClN_2_O_3_S_2_, 65), 465 (M‐H, C_20_H_11_^37^Cl_2_N_2_O_3_S_2_, 15).

##### (Z)‐N‐(5‐(2,4‐dichlorobenzylidene)‐4‐oxo‐4,5‐dihydrothiazol‐2‐yl)naphthalene‐1‐sulfonamide 35

Synthesized using General Procedure 2 with **5** (150 mg, 0.49 mmol, 1 eq.) and 2,4‐dichlorobenzaldehyde (129 mg, 0.734 mmol, 1.1 eq.) for 60 min to give the desired product as a pale orange solid (110 mg, 49%). M.P: >202 °C (dec).

^1^H NMR (600 MHz, DMSO‐*d*_6_) *δ* 8.59 (d, *J* = 8.6 Hz, 1H), 8.29 (dd, *J* = 14.3, 7.8 Hz, 2H), 8.12 (d, *J* = 8.1 Hz, 1H), 7.85 (d, *J* = 1.6 Hz, 1H), 7.79 – 7.76 (m, 2H), 7.71 – 7.67 (m, 4H) ppm. NH exchanging—not observed. ^13^C NMR (151 MHz, DMSO‐*d*_6_) *δ* 166.2, 165.1, 135.8, 135.5, 135.1, 134.8, 133.8, 130.3, 130.0, 129.9, 129.0, 128.6, 128.4, 128.1, 127.6, 127.2, 127.1, 126.3, 124.8, 124.6 ppm. IR υ_max_ (cm^−1^): 3054, 2983, 2893, 2810, 1703, 1569, 1331, 1130, 764. Mass spectral analysis: LRMS (ESI‐) *m/z*: 461 (M‐H, C_20_H_11_^35^Cl_2_N_2_O_3_S_2_, 100), 463 (M‐H, C_20_H_11_^35/37^Cl_2_N_2_O_3_S_2_, 75).

##### (Z)‐N‐(5‐(3,4‐dichlorobenzylidene)‐4‐oxo‐4,5‐dihydrothiazol‐2‐yl)naphthalene‐1‐sulfonamide 36

Synthesized using General Procedure 2 with **5** (147 mg, 0.49 mmol, 1 eq.) and 3,4‐dichlorobenzaldehyde (111 mg, 0.54 mmol, 1.1 eq.) for 30 min to give the desired product as a pale orange solid (155 mg, 68%). M.P: >201 °C (dec).

^1^H NMR (600 MHz, DMSO‐*d*_6_) *δ* 13.29 (br, s, 1H, NH), 8.61 (d, *J* = 8.6 Hz, 1H), 8.30 (d, *J* = 7.5 Hz, 2H), 8.12 (d, *J* = 8.2 Hz, 1H), 7.94 (d, *J* = 1.8 Hz, 1H), 7.85 (d, *J* = 8.4 Hz, 1H), 7.77 (t, *J* = 7.8 Hz, 1H), 7.74 (s, 1H) 7.72 – 7.67 (m 2H), 7.61 (dd, *J* = 8.4, 1.9 Hz, 1H) ppm. ^13^C NMR (151 MHz, DMSO‐*d*_6_) *δ* 165.4 (br), 165.2 (br), 135.2, 134.8, 133.8, 133.6, 133.1, 132.5, 132.1, 131.6, 130.7, 129.01, 129.00, 128.3, 128.2, 127.6, 127.1, 124.8, 124.6, 124.3 (br) ppm. IR υ_max_ (cm^−1^): 3155, 3060, 2987, 2780, 1706, 1557, 1315, 1129, 766. Mass spectral analysis: LRMS (ESI‐) *m/z*: 461 (M‐H, C_20_H_11_^35^Cl_2_N_2_O_3_S_2_, 100), 463 (M‐H, C_20_H_11_^35/37^Cl_2_N_2_O_3_S_2_, 65), 465 (M‐H, C_20_H_11_^37^Cl_2_N_2_O_3_S_2_, 15).

##### (Z)‐N‐(5‐(2,6‐dichlorobenzylidene)‐4‐oxo‐4,5‐dihydrothiazol‐2‐yl)naphthalene‐1‐sulfonamide 37

Synthesized using General Procedure 2 with **5** (149 mg, 0.49 mmol, 1 eq.) and 2,6‐dichlorobenzaldehyde (128 mg, 0.735 mmol, 1.1 eq.) for 1.5 h to give the desired product as an off white solid (127 mg, 56%). M.P: 232–235 °C.

^1^H NMR (400 MHz, DMSO‐*d*_6_) *δ* 8.57 (d, *J* = 8.5 Hz, 1H), 8.30 (d, *J* = 8.2 Hz, 1H), 8.21 (d, *J* = 7.3 Hz, 1H), 8.12 (d, *J* = 8.0 Hz, 1H), 7.76 (t, *J* = 7.2 Hz, 1H), 7.72 – 7.61 (m, 5 H), 7.56–7.52 (m, 1H) ppm. NH exchanging—not observed. ^13^C NMR (101 MHz, DMSO‐*d*_6_) *δ* 165.3, 164.9, 135.0, 134.8, 133.8, 133.1, 132.1, 131.2, 130.5, 130.0, 129.04, 128.95, 128.89 (2C), 128.3, 128.1, 127.6, 127.1, 124.8, 124.6 ppm. IR υ_max_ (cm^−1^): 3237, 2980, 1736, 1569, 1335, 1126, 767. Mass spectral analysis: LRMS (ESI‐): 461 (M‐H, C_20_H_11_^35^Cl_2_N_2_O_3_S_2_, 100), 463 (M‐H, C_20_H_11_^35^Cl^37^ClN_2_O_3_S_2_, 65); (ESI+): 463 (M‐H, C_20_H_13_^35^Cl_2_N_2_O_3_S_2_, 100), 465 (M‐H, C_20_H_13_^35^Cl^37^ClN_2_O_3_S_2_, 85).

##### (Z)‐N‐(5‐(3,4‐difluorobenzylidene)‐4‐oxo‐4,5‐dihydrothiazol‐2‐yl)naphthalene‐1‐sulfonamide 38

Synthesized using General Procedure 2 with **5** (155 mg, 0.49 mmol, 1 eq.) and 3,4‐difluorobenzaldehyde (0.06 mL, 0.54 mmol, 1.1 eq.) for 30 min to give the desired product as an off white solid (92 mg, 44%). M.P: >125 °C (dec).

^1^H NMR (600 MHz, DMSO‐*d*_6_) *δ* 13.24 (br, s, 1H, NH), 8.62 (d, *J* = 8.3 Hz, 1H), 8.30 (t, *J* = 7.9 Hz, 2H), 8.11 (d, *J* = 7.8 Hz, 1H), 7.77 – 7.68 (m, 6H), 7.52 (s, 1H) ppm. ^13^C NMR (151 MHz, DMSO‐*d*_6_) *δ* 165.7, 150.4 (dd, ^1/2^ *J*_CF_ = 12.86, 252.58 Hz), 149.6 (dd, ^1/2^ *J*_CF_ = 13.18, 247.53 Hz), 135.3, 134.7, 133.8, 131.0, 130.8 (unresolved quartet), 129.0, 128.3, 128.1, 127.6, 127.1, 126.9 (unresolved quartet), 124.9, 124.6, 123.8 (br), 119.7 (d, ^2^ *J*_CF_ = 17.83 Hz), 118.7 (d, ^2^ *J*_CF_ = 17.89 Hz) ppm. One carbon not observed. ^19^F NMR (376 MHz, DMSO‐*d*_6_) *δ* −130.71, −133.36 ppm. IR υ_max_ (cm^−1^): 3280, 2983, 2887, 1709, 1587, 1318, 1125. Mass spectral analysis: LRMS (ESI‐) *m/z* (%): 429 (M‐H, C_20_H_11_F_2_N_2_O_3_S_2_, 100); (ESI+) *m/z* (%): 431 (M + H, C_20_H_13_F_2_N_2_O_3_S_2_, 100).

##### Library 3: (Z)‐N‐(5‐(cyclohex‐3‐en‐1‐ylmethylene)‐4‐oxo‐4,5‐dihydrothiazol‐2‐yl)naphthalene‐1‐sulfonamide 39

Synthesized using General Procedure 2 with **5** (150 mg, 0.49 mmol, 1 eq.) and 1,2,3,6‐tetrahydrobenzaldehyde (0.06 mL, 0.54 mmol, 1.1 eq.) for 20 min. Addition of hexane (≈2 mL) allowed precipitation to give the desired product as a pale pink solid (56 mg, 28%). M.P: >202 °C (dec). R_f_ 0.58 (10% MeOH in CH_2_Cl_2_).

^1^H NMR (400 MHz, DMSO‐*d*_6_) *δ* 12.93 (br, s, 1H, NH), 8.58 (d, *J* = 8.6 Hz, 1H), 8.30–8.25 (m, 2H), 8.11 (d, *J* = 8.0 Hz, 1H), 7.78–7.66 (m, 3H), 6.84 (d, *J* = 9.7 Hz, 1H), 5.74 – 5.66 (m, 2H), 2.50 – 2.43 (m, 1H, partially hidden under DMSO‐*d*_6_ solvent peak), 2.17 – 1.96 (m, 4H), 1.77–1.74 (m, 1H), 1.59–1.53 (m, 1H) ppm. ^13^C NMR (101 MHz, DMSO‐*d*_6_) *δ* 165.7, 165.5, 142.7, 135.2, 134.6, 133.8, 129.0, 128.2, 128.1, 127.6, 127.1, 126.8, 124.9, 124.7, 124.6, 124.0, 36.5, 29.0, 26.4, 23.3 ppm. IR υ_max_ (cm^−1^): 3137, 3025, 2901, 1711, 1635, 1543, 1342, 1132. Mass spectral analysis: LRMS (ESI+) *m/z*: 399 (M + H, C_20_H_19_N_2_O_3_S_2_, 100%); (ESI‐) *m/z*: 397 (M‐H, C_20_H_17_N_2_O_3_S_2_, 100%).

##### (Z)‐N‐(5‐(cyclohexylmethylene)‐4‐oxo‐4,5‐dihydrothiazol‐2‐yl)naphthalene‐1‐sulfonamide 40

Synthesized using General Procedure 2 with **5** (154 mg, 0.49 mmol, 1 eq.) and cyclohexanecarboxaldehyde (0.1 mL, 0.735 mmol, 1.1 eq.) for 60 min to give the desired product as a white solid (64 mg, 33%). M.P: >221 °C (dec).

^1^H NMR (600 MHz, DMSO‐*d*_6_) *δ* 12.96 (br, s, 1H, NH), 8.57 (d, *J* = 8.6 Hz, 1H), 8.31 – 8.25 (m, 2H), 8.12 (d, *J* = 8.1 Hz, 1H), 7.77 – 7.75 (m, 1H), 7.72–7.67 (m, 2H), 6.78 (d, *J* = 9.7 Hz, 1H), 2.23 (q, *J* = 10.2 Hz, 1H), 1.72 – 1.68 (m, 4H), 1.65–1.63 (m, 1H), 1.36–1.26 (m, 4H), 1.24 – 1.17 (m, 1H) ppm. ^13^C DEPTQ (151 MHz, DMSO‐*d*_6_) *δ* 165.6, 165.4, 143.5, 135.3, 134.7, 133.8, 129.0, 128.3, 128.0, 127.6, 127.1, 124.9, 124.6, 123.2, 40.6, 30.5 (2C), 25.1, 24.6 (2C) ppm. IR υ_max_ (cm^−1^): 3125 (N‐H), 3060, 2997, 2929, 2852, 1711, 1544, 1344, 1134. Mass spectral analysis: LRMS (ESI‐) *m/z*: 399 (M‐H, C_20_H_19_N_2_O_3_S_2_, 100); (ESI+) *m/z*: 401 (M + H, C_20_H_21_N_2_O_3_S_2_, 100).

##### (Z)‐N‐(5‐(cyclopentylmethylene)‐4‐oxo‐4,5‐dihydrothiazol‐2‐yl)naphthalene‐1‐sulfonamide 41

Synthesized using General Procedure 2 with **5** (108 mg, 0.35 mmol, 1 eq.) and cyclopentanecarbaldehyde (0.04 mL, 0.39 mmol, 1.1 eq.) to give the desired product as a white solid (85 mg, 63%). M.P: >218 °C (dec).

^1^H NMR (400 MHz, DMSO‐*d*_6_) *δ* 12.94 (s, 1H, NH), 8.58 (d, *J* = 8.6 Hz, 1H), 8.31–8.24 (m, 2H), 8.11 (d, *J* = 8.0 Hz, 1H), 7.78–7.74 (m, 1H), 7.72–7.66 (m, 2H), 6.88 (d, *J* = 9.8 Hz, 1H), 2.67–2.56 (m, 1H), 1.92–1.87 (m, 2H), 1.73–1.56 (m, 4H), 1.48–1.39 (m, 2H) ppm. ^13^C NMR (101 MHz, DMSO‐*d*_6_) *δ* 165.7, 165.2, 144.0, 135.3, 134.7, 133.8, 129.0, 128.3, 128.1, 127.6, 127.1, 124.9, 124.6, 123.5, 42.3, 32.1 (2C), 25.1 (2C) ppm. IR υ_max_ (cm^−1^): 3146, 3049, 2945, 1710, 1539, 1337, 1158. Mass spectral analysis: LRMS (ESI‐) m/z (%): 385.1 (M‐H, C_19_H_17_N_2_O_3_S_2_, 100%); (ESI+) m/z: 387.1 (M + H, C_19_H_19_N_2_O_3_S_2_, 100%).

##### (Z)‐N‐(4‐oxo‐5‐pentylidene‐4,5‐dihydrothiazol‐2‐yl)naphthalene‐1‐sulfonamide 42

Synthesized using General Procedure 2 with **5** (300 mg, 0.98 mmol, 1 eq.) and valaraldehyde (0.12 mL, 1.00 mmol, 1.1 eq.) to give the desired product as a chalk white solid (210 mg, 57%). M.P: >145 °C (dec).

^1^H NMR (400 MHz, DMSO‐*d*_*6*_) *δ* 12.95 (s, 1H, NH), 8.58 (d, *J* = 8.6 Hz, 1H), 8.31–8.25 (m, 2H), 8.12 (d, *J* = 8.1 Hz, 1H), 7.78–7.74 (m, 1H), 7.72 – 7.67 (m, 2H), 6.91 (t, *J* = 7.8 Hz, 1H), 2.27 (q, *J* = 7.4 Hz, 2H), 1.51 – 1.44 (m, 2H), 1.36–1.44 (m, 2H), 0.88 (t, *J* = 7.3 Hz, 3H) ppm. ^13^C NMR (101 MHz, DMSO‐*d*_6_) *δ* 165.6, 164.9, 139.7, 135.2, 134.7, 133.8, 129.0, 128.3, 128.1, 127.6, 127.1, 124.9, 124.8, 124.6, 30.9, 29.4, 21.8, 13.6 ppm. IR υ_max_ (cm^−1^): 3153, 3062, 2965, 2962, 1715, 1546, 1350, 1131. Mass Spectral analysis: LRMS (ESI‐) m/z (%): 373.1 (M‐H, C_18_H_17_N_2_O_3_S_2_, 100%); (ESI+) m/z: 375.1 (M + H, C_18_H_19_N_2_O_3_S_2_, 100%).

##### (Z)‐N‐(5‐octylidene‐4‐oxo‐4,5‐dihydrothiazol‐2‐yl)naphthalene‐1‐sulfonamide 43

Synthesized using General Procedure 2 with **5** (157 mg, 0.49 mmol, 1 eq.) and octanal (0.11 mL, 0.735 mmol, 1.1 eq.) for 45 min to give the desired product as a white solid (144 mg, 71%). M.P: >125 °C (dec).

^1^H NMR (600 MHz, DMSO‐*d*_6_) *δ* 12.95 (br, s, 1H, NH), 8.58 (d, *J* = 8.4 Hz, 1H), 8.27 (dd, *J* = 23.3, 7.4 Hz, 2H), 8.11 (d, *J* = 7.6 Hz, 1H), 7.75 (t, *J* = 7.4 Hz, 1H), 7.69–7.68 (m, 2H), 6.90 (t, *J* = 7.4 Hz, 1H), 2.26 (d, *J* = 6.3 Hz, 2H), 1.49 (s, 2H), 1.27 (s, 8H), 0.85 (s, 3H) ppm. ^13^C DEPTQ (151 MHz, DMSO‐*d*_6_) *δ* 165.7, 165.0, 139.7, 135.2, 134.7, 133.8, 129.0, 128.2, 128.1, 127.6, 127.1, 125.0, 124.9, 124.6, 31.1 (2C), 28.6, 28.4, 27.3, 22.0, 13.9 ppm. IR υ_max_ (cm^−1^): 3060, 2947, 2929, 2858, 1714, 1542, 1345, 1131. Mass spectral analysis: LRMS (ESI‐) *m/z*: 415 (M‐H, C_21_H_23_N_2_O_3_S_2_, 100); (ESI+) *m/z*: 417 (M + H, C_21_H_25_N_2_O_3_S_2_, 100).

##### Library 4: (Z)‐N‐(4‐oxo‐5‐(thiophen‐3‐ylmethylene)‐4,5‐dihydrothiazol‐2‐yl)naphthalene‐1‐sulfonamide 44

Synthesized using General Procedure 2 with **5** (147 mg, 0.48 mmol, 1 eq.) and 2‐thiophene carboxaldehyde (0.05 mL, 0.54 mmol, 1.1 eq.) for 30 min to give the desired product as a yellow solid (141 mg, 73%). M.P: >259 °C (dec). R_f_ 0.46 (10% MeOH in CH_2_Cl_2_).

^1^H NMR (400 MHz, DMSO‐*d*_6_) *δ* 13.15 (br, s, 1H, NH), 8.61 (d, *J* = 8.6 Hz, 1H), 8.30 (d, *J* = 7.6 Hz, 2H), 8.12 (d, *J* = 8.0 Hz, 1H), 8.08 (d, *J* = 5.0 Hz, 1H), 8.04 (s, 1H), 7.77–7.69 (m, 4H), 7.32 (dd, *J* = 5.0, 3.7 Hz, 1H) ppm. ^13^C NMR (101 MHz, DMSO‐*d*_6_) *δ* 166.3, 164.9, 137.0, 135.7, 135.3, 134.7, 134.0, 133.8, 129.2, 129.0, 128.3, 128.1, 127.6, 127.1, 126.8, 124.9, 124.6, 119.0 ppm. IR υ_max_ (cm^−1^): 3102, 3062, 3007, 1698, 1557, 1317, 1126. Mass Spectral analysis: LRMS (ESI‐) *m/z*: 399 (M‐H, C_18_H_11_N_2_O_3_S_3_, 100%).

##### (Z)‐N‐(5‐((1H‐pyrrol‐3‐yl)methylene)‐4‐oxo‐4,5‐dihydrothiazol‐2‐yl)naphthalene‐1‐sulfonamide 45

Synthesized using General Procedure 2 with **5** (150 mg, 0.49 mmol, 1 eq.) and pyrrole‐3‐carbonaldehyde (55 mg, 0.58 mmol, 1.1 eq.) for 30 min to give the desired product as a yellow/brown solid (53 mg, 28%). M.P: >264 °C (dec). R_f_ 0.42 (10% MeOH in CH_2_Cl_2_).

^1^H NMR (400 MHz, DMSO‐*d*_6_) *δ* 12.84 (s, 1H, NH), 11.70 (s, 1H, NH), 8.61 (d, *J* = 8.6 Hz, 1H), 8.32 – 8.27 (m, 2H), 8.11 (d, *J* = 8.0 Hz, 1H), 7.78–7.66 (m, 4 H), 7.48–7.47 (m, 1H), 7.04 (m, 1H), 6.37 (d, *J* = 2.3 Hz, 1H) ppm. ^13^C NMR (101 MHz, DMSO‐*d*_6_) *δ* 166.5, 165.9, 135.5, 134.5, 133.8, 130.4, 129.0, 128.2, 128.1, 127.7, 127.0, 126.6, 124.9, 124.6, 122.0, 118.0, 113.2, 107.9 ppm. IR υ_max_ (cm^−1^): 3639, 3498, 3177, 3042, 1695, 1591, 1538, 1355, 1123. Mass spectral analysis: LRMS (ESI+) *m/z*: 384 (M + H, C_18_H_14_N_3_O_3_S_2_, 40%); (ESI‐) *m/z*: 382 (M‐H, C_18_H_12_N_3_O_3_S_2_, 100%).

##### (Z)‐N‐(5‐(furan‐3‐ylmethylene)‐4‐oxo‐4,5‐dihydrothiazol‐2‐yl)naphthalene‐1‐sulfonamide 46

Synthesized using General Procedure 2 with **5** (148 mg, 0.48 mmol, 1 eq.) and 3‐furanaldehyde (0.05 mL, 0.54 mmol, 1.1 eq.) for 30 min to give the desired product as a light brown solid (145 mg, 78%). M.P: >245 °C (dec). R_f_ 0.42 (10% MeOH in CH_2_Cl_2_).

^1^H NMR (400 MHz, DMSO‐*d*_6_) *δ* 13.08 (br, s, 1H, NH), 8.61 (d, *J* = 8.5 Hz, 1H), 8.34 – 8.29 (m, 3H), 8.12 (d, *J* = 8.1 Hz, 1H), 7.93 (s, 1H), 7.79–7.75 (m, 1H), 7.73–7.67 (m, 3H), 6.80 (d, *J* = 1.3 Hz, 1H) ppm. ^13^C NMR (101 MHz, DMSO‐*d*_6_) *δ* 166.2, 165.2, 148.2, 146.1, 135.2, 134.7, 133.8, 129.0, 128.28, 128.25, 127.6, 127.1, 125.0, 124.9, 124.6, 120.49, 120.46, 108.9 ppm. IR υ_max_ (cm^−1^): 3138, 3026, 2958, 1705, 1611, 1543, 1338, 1558, 1130. Mass spectral analysis: LRMS (ESI+) *m/z*: 385 (M + H, C_18_H_13_N_2_O_4_S_2_, 100%); (ESI‐) *m/z*: 383 (M‐H, C_18_H_11_N_2_O_4_S_2_, 100%).

##### (Z)‐N‐(5‐((1H‐pyrrol‐2‐yl)methylene)‐4‐oxo‐4,5‐dihydrothiazol‐2‐yl)naphthalene‐1‐sulfonamide 47

Synthesized using General Procedure 2 with **5** (148 mg, 0.48 mmol, 1 eq.) and pyrrol‐2‐carboxaldehyde (52.3 mg, 0.55 mmol, 1.1 eq.) for 30 min to give the desired product as a deep orange solid (110 mg, 60%). M.P: >278 °C (dec). R_f_ 0.50 (10% MeOH in CH_2_Cl_2_).

^1^H NMR (400 MHz, DMSO‐*d*_6_) *δ* 12.91 (br, s, 1H, NH), 11.81 (s, 1H), 8.61 (d, *J* = 8.7 Hz, 1H), 8.33–8.28 (m, 2H), 8.11 (d, *J* = 8.0 Hz, 1H), 7.78–7.74 (m, 1H), 7.73–7.66 (m, 2H), 7.62 (s, 1H), 7.29–7.28 (m, 1H), 6.62 (s, 1H), 6.44–6.42 (m, 1H) ppm. ^13^C NMR (101 MHz, DMSO‐*d*_6_) *δ* 166.4, 165.6, 135.4, 134.6, 133.8, 129.0, 128.2, 128.1, 127.7, 127.1, 126.7, 125.6, 124.9, 124.6, 124.0, 114.7, 112.8, 112.5 ppm. IR υ_max_ (cm^−1^): 3266, 3112, 1705, 1557, 1337, 1125. Mass Spectral analysis: LRMS (ESI+) *m/z* (%): 384 (M + H, C_18_H_14_N_3_O_3_S_2_, 100); (ESI‐) *m/z* (%): 382 (M‐H, C_18_H_12_N_3_O_3_S_2_, 100).

##### (Z)‐N‐(5‐(furan‐2‐ylmethylene)‐4‐oxo‐4,5‐dihydrothiazol‐2‐yl)naphthalene‐1‐sulfonamide 48

Synthesized using General Procedure 2 with **5** (149 mg, 0.49 mmol, 1 eq.) and 2‐furaldehyde (0.05 mL, 0.54 mmol, 1.1 eq.) for 30 min to give the desired product as a pale brown solid (103 mg, 55%). M.P: >249 °C (dec). R_f_ 0.35 (10% MeOH in CH_2_Cl_2_).

^1^H NMR (400 MHz, DMSO‐*d*_6_) *δ* 13.03 (br, s, 1H, NH), 8.60 (d, *J* = 8.7 Hz, 1H), 8.31–8.27 (m, 2H), 8.19 (d, *J* = 1.7 Hz, 1H), 8.12 (d, *J* = 8.1 Hz, 1H), 7.79–7.74 (m, 1H), 7.73–7.67 (m, 2H), 7.61 (s, 1H), 7.18 (d, *J* = 3.5 Hz, 1H), 6.80 (dd, *J* = 3.5, 1.7 Hz, 1H) ppm. ^13^C NMR (101 MHz, DMSO‐*d*_6_) *δ* 166.6, 166.4, 149.2, 148.2, 135.4, 134.6, 133.8, 129.0, 128.3, 127.9, 127.6, 127.1, 124.9, 124.6, 120.0, 119.9, 118.4, 113.8 ppm. IR υ_max_ (cm^−1^): 3132, 3029, 1699, 1608, 1543, 1333, 1158, 1129. Mass Spectral analysis: LRMS (ESI+) *m/z* (%): 385 (M + H, C_18_H_13_N_2_O_4_S_2_, 90%); (ESI‐) *m/z* (%): 383 (M‐H, C_18_H_11_N_2_O_4_S_2_, 100%).

##### (Z)‐N‐(5‐((5‐chlorofuran‐2‐yl)methylene)‐4‐oxo‐4,5‐dihydrothiazol‐2‐yl)naphthalene‐1‐sulfonamide 49

Synthesized using General Procedure 2 with **5** (151 mg, 0.49 mmol, 1 eq.) and 5‐chloro‐2‐furaldehyde (73 mg, 0.56 mmol, 1.1 eq.) for 30 min to give the desired product as a deep red solid (139 mg, 68%). M.P: >240 °C (dec). R_f_ 0.48 (10% MeOH in CH_2_Cl_2_).

^1^H NMR (400 MHz, DMSO‐*d*_6_) *δ* 13.08 (br, s, 1H, NH), 8.60 (d, *J* = 8.5 Hz, 1H), 8.31–8.28 (m, 2H), 8.12 (d, *J* = 8.1 Hz, 1H), 7.79–7.75 (m, 1H), 7.73–7.67 (m, 2H), 7.53 (s, 1H), 7.23 (d, *J* = 3.6 Hz, 1H), 6.83 (d, *J* = 3.6 Hz, 1H) ppm. ^13^C NMR (101 MHz, DMSO‐*d*_6_) *δ* 166.5, 166.2, 149.2, 140.1, 135.4, 134.7, 133.8, 129.0, 128.3, 128.1, 127.6, 127.1, 124.9, 124.6, 121.6, 119.4, 118.6, 111.2 ppm. IR υ_max_ (cm^−1^): 3137, 3034, 2940, 1698, 1606, 1545, 1316, 1127, 874. Mass Spectral analysis: LRMS (ESI+) *m/z*: 419 (M + H, C_18_H_12_ClN_2_O_4_S_2_, 100%); (ESI‐) *m/z*: 417 (M‐H, C_18_H_10_ClN_2_O_4_S_2_, 100%).

##### (Z)‐N‐(5‐((5‐bromofuran‐2‐yl)methylene)‐4‐oxo‐4,5‐dihydrothiazol‐2‐yl)naphthalene‐1‐sulfonamide 50

Synthesized using General Procedure 2 with **5** (147 mg, 0.48 mmol, 1 eq.) and 5‐bromo‐2‐furaldehyde (105 mg, 0.60 mmol, 1.1 eq.) for 30 min to give the desired product as a bright red solid (108 mg, 82%). M.P: >243 °C (dec). R_f_ 0.46 (10% MeOH in CH_2_Cl_2_).

^1^H NMR (400 MHz, DMSO‐*d*_6_) *δ* 13.07 (br, s, 1H, NH), 8.59 (d, *J* = 8.6 Hz, 1H), 8.32–8.28 (m, 2H), 8.12 (d, *J* = 8.1 Hz, 1H), 7.79–7.75 (m, 1H), 7.74–7.67 (m, 2H), 7.54 (s, 1H), 7.19 (d, *J* = 3.6 Hz, 1H), 6.93 (d, *J* = 3.6 Hz, 1H) ppm. ^13^C NMR (101 MHz, DMSO‐*d*_6_) *δ* 166.3, 166.1, 151.3, 135.3, 134.7, 133.8, 129.0, 128.3, 128.1, 127.9, 127.6, 127.1, 124.8, 124.6, 121.8, 119.3, 118.6, 116.0 ppm. IR υ_max_ (cm^−1^): 3133, 3034, 1695, 1542, 1314, 1125, 594. Mass Spectral analysis: LRMS (ESI+) *m/z*: 463 (M + H, C_18_H_12_^79^BrN_2_O_4_S_2_, 90%), 465 (M + H, C_18_H_12_^81^BrN_2_O_4_S_2_, 100%); (ESI‐) *m/z*: 461 (M‐H, C_18_H_10_^79^BrN_2_O_4_S_2_, 100%), 463 (M‐H, C_18_H_10_^81^BrN_2_O_4_S_2_, 100%).

##### (Z)‐N‐(5‐((5‐(hydroxymethyl)furan‐2‐yl)methylene)‐4‐oxo‐4,5‐dihydrothiazol‐2‐yl)naphthalene‐1‐sulfonamide 51

Synthesized using General Procedure 2 with **5** (151 mg, 0.49 mmol, 1 eq.) and 5‐hydroxymethyl‐2‐furaldehyde (74.7 mg, 0.59 mmol, 1.1 eq.) for 30 min to give the desired product as an orange solid (65 mg, 32%). M.P: >267 °C (dec). R_f_ 0.36 (10% MeOH in CH_2_Cl_2_).

^1^H NMR (400 MHz, DMSO‐*d*_6_) *δ* 12.99 (br, s, 1H, NH), 8.59 (d, *J* = 8.6 Hz, 1H), 8.30 (d, *J* = 7.7 Hz, 2H), 8.12 (d, *J* = 7.9 Hz, 1H), 7.77 (ddd, *J* = 8.5, 6.9, 1.3 Hz, 1H), 7.73–7.67 (m, 2H), 7.57 (s, 1H), 7.13 (d, *J* = 3.5 Hz, 1H), 6.62 (d, *J* = 3.5 Hz, 1H), 5.54 (br, s, 1H, OH), 4.56 (s, 2H) ppm. ^13^C NMR (101 MHz, DMSO‐*d*_6_) *δ* 166.6, 166.3, 161.1, 148.5, 135.4, 134.6, 133.8, 129.0, 128.3, 128.1, 127.7, 127.1, 124.9, 124.6, 121.2, 120.0, 117.5, 111.0, 56.0 ppm. IR υ_max_ (cm^−1^): 3504, 3031, 2882, 2697, 1702, 1556, 1332, 1159, 1126. Mass spectral analysis: LRMS (ESI‐) *m/z*: 413 (M‐H, C_19_H_13_N_2_O_5_S_2_, 100%).

##### (Z)‐N‐(4‐oxo‐5‐((3‐oxo‐3,4‐dihydro‐2H‐benzo[b][1,4]oxazin‐6‐yl)methylene)‐4,5‐dihydrothiazol‐2‐yl)naphthalene‐1‐sulfonamide 52

Synthesized using General Procedure 2 with **5** (102 mg, 0.33 mmol, 1 eq.) and 3‐oxo‐3,4‐dihydro‐2*H*‐benzo[1,4]oxazine‐6‐carbaldehyde (69 mg, 0.39 mmol, 1.1 eq.) for 30 min to give the desired product as an orange/yellow solid (127 mg, 83%). M.P: >300 °C (dec). R_f_ 0.24 (10% MeOH in CH_2_Cl_2_).

^1^H NMR (400 MHz, DMSO‐*d*_6_) *δ* 11.12 (s, 1H, NH), 8.61 (d, *J* = 8.7 Hz, 1H), 8.32–8.29 (m, 2H), 8.12 (d, *J* = 8.0 Hz, 1H), 7.77 (ddd, *J* = 8.5, 6.9, 1.4 Hz, 1H), 7.73–7.67 (m, 2H), 7.66 (s, 1H), 7.29 (dd, *J* = 8.5, 2.1 Hz, 1H), 7.22 (d, *J* = 2.1 Hz, 1H), 7.13 (d, *J* = 8.4 Hz, 1H), 4.70 (s, 2H) ppm. NH exchanging—not observed. ^13^C NMR (151 MHz, DMSO‐*d*_6_) *δ* 165.9 (br), 164.3, 145.5, 135.4, 134.7, 133.8, 133.3 (br), 129.0, 128.3, 128.10, 128.08, 127.7, 127.2, 127.1, 127.0, 124.9, 124.6, 117.1, 116.1, 66.8 ppm. Two quaternary carbons not observed. IR υ_max_ (cm^−1^): 3178, 3036, 1726, 1669, 1553, 1324, 1126. Mass Spectral analysis: LRMS (ESI‐) *m/z*: 464 (*M*‐H, C_22_H_14_N_3_O_5_S_2_, 100%).

##### (Z)‐N‐(5‐((4‐methyl‐3,4‐dihydro‐2H‐benzo[b][1,4]oxazin‐7‐yl)methylene)‐4‐oxo‐4,5‐dihydrothiazol‐2‐yl)naphthalene‐1‐sulfonamide 53

Synthesized using General Procedure 2 with **5** (150 mg, 0.49 mmol, 1 eq.) and 4‐methyl‐3,4‐dihydro‐*2H*‐1,4‐benzoxazio‐7‐carboxaldehyde (100 mg, 0.54 mmol, 1.1 eq.) for 30 min to give the desired product as a red solid (77 mg, 34%). M.P: >242 °C (dec).

^1^H NMR (400 MHz, DMSO‐*d*_6_) *δ* 12.95 (s, 1H, NH), 8.61 (d, *J* = 8.5 Hz, 1H), 8.30 (dd, *J* = 7.8, 4.2 Hz, 2H), 8.11 (d, *J* = 8.1 Hz, 1H), 7.78 – 7.74 (m, 1H), 7.73 – 7.67 (m, 2H), 7.57 (s, 1H), 7.16 (dd, *J* = 8.5, 2.0 Hz, 1H), 6.97 (d, *J* = 2.1 Hz, 1H), 6.82 (d, *J* = 8.6 Hz, 1H), 4.25 – 4.22 (m, 2H), 3.43 – 3.41 (m, 2H), 2.97 (s, 3H) ppm. ^13^C NMR (101 MHz, DMSO‐*d*_6_) *δ* 165.8, 143.2, 139.6, 135.6, 134.8, 134.5, 133.8, 129.0, 128.2, 128.0, 127.7, 127.1, 126.5, 124.9, 124.6, 120.8, 116.5, 114.5, 111.8, 63.8, 48.0, 37.7 ppm. One quaternary carbon not observed. IR υ_max_ (cm^−1^): 2926, 2977, 1684, 1520, 1300, 1213, 1122. Mass spectral analysis: LRMS (ESI‐) *m/z* (%): 464 (*M*‐H, C_23_H_18_N_3_O_4_S_2_, 100%).

##### (Z)‐N‐(5‐((2,3‐dihydrobenzo[b][1,4]dioxin‐6‐yl)methylene)‐4‐oxo‐4,5‐dihydrothiazol‐2‐yl)naphthalene‐1‐sulfonamide 54

Synthesized using General Procedure 2 with **5** (197 mg, 0.65 mmol, 1 eq.) and 2,3‐dihydrobenzo[*b*][1,4]dioxine‐6‐carbaldehyde (125 mg, 0.76 mmol, 1.1 eq.) for 40 min to give the desired product as a red/orange solid (188 mg, 64%). M.P: >244 °C (dec). R_f_ 0.61 (10% MeOH in CH_2_Cl_2_).

^1^H NMR (400 MHz, DMSO‐*d*_6_) *δ* 8.62 – 8.60 (m, 1H), 8.30 (dt, *J* = 8.3, 2.5 Hz, 2H), 8.12 (d, *J* = 8.0 Hz, 1H), 7.77 (ddd, *J* = 8.5, 6.9, 1.4 Hz, 1H), 7.72 – 7.66 (m, 3H), 7.19 – 7.16 (m, 2H), 7.08–7.05 (m, 1H), 4.34–4.30 (m, 4H) ppm. NH exchanging—not observed. ^13^C NMR (101 MHz, DMSO‐*d*_6_) *δ* 166.6, 165.7, 146.1, 143.8, 135.3, 134.7, 133.8, 133.7, 129.0, 128.3, 128.1, 127.6 (2C), 127.1, 126.1, 124.9, 124.7, 124.2, 119.1, 118.2, 64.6, 64.0 ppm. IR υ_max_ (cm^−1^): 3056, 2926, 2771, 1694, 1553, 1289, 1123. Mass spectral analysis: LRMS (ESI‐) *m/z*: 451 (M‐H, C_22_H_15_N_2_O_5_S_2_, 100%).

##### (Z)‐N‐(5‐((2H‐chromen‐3‐yl)methylene)‐4‐oxo‐4,5‐dihydrothiazol‐2‐yl)naphthalene‐1‐sulfonamide 55

Synthesized using General Procedure 2 with **5** (149 mg, 0.49 mmol, 1 eq.) and 2*H*‐chromene‐3‐carbaldehyde (87 mg, 0.54 mmol, 1.1 eq.) for 110 min to give the desired product as an orange/red solid (58 mg, 26%). M.P: >237 °C (dec).

^1^H NMR (400 MHz, DMSO‐*d*_6_) *δ* 8.60 (d, *J* = 8.5 Hz, 1H), 8.31 (d, *J* = 7.5 Hz, 2H), 8.12 (d, *J* =  8.0 Hz, 1H), 7.79–7.75 (m, 1H), 7.73–7.67 (m, 2H), 7.32 (s, 1H), 7.28–7.24 (m, 2H), 7.17 (s, 1H), 6.97 (td, *J* = 7.5, 1.0 Hz, 1H), 6.87–6.85 (m, 1H), 5.12 (s, 2H) ppm. NH exchanging—not observed. ^13^C DEPTQ NMR (151 MHz, DMSO‐*d*_6_) *δ* 166.4, 165.0, 153.6, 135.2, 134.7, 133.8, 133.0, 131.8, 130.9, 129.0, 128.6, 128.32, 128.25, 127.9, 127.6, 127.1, 124.9, 124.7, 122.6, 121.9, 121.0, 115.7, 65.7 ppm. IR υ_max_ (cm^−1^): 3114, 2939, 2784, 1690, 1551, 1350, 1157, 1127. Mass spectral analysis: LRMS (ESI‐) *m/z* (%): 447 (M‐H, C_23_H_15_N_2_O_4_S_2_, 100%).

##### (Z)‐N‐(5‐(benzo[d][1,3]dioxol‐5‐ylmethylene)‐4‐oxo‐4,5‐dihydrothiazol‐2‐yl)naphthalene‐1‐sulfonamide 56

Synthesized using General Procedure 2 with **5** (152 mg, 0.49 mmol, 1 eq.) and benzo[*d*][1,3]dioxole‐5‐carbaldehyde (83 mg, 0.54 mmol, 1.1 eq.) for 30 min to give the desired product as a yellow solid (151 mg, 70%). M.P: >274 °C (dec). R_f_ 0.46 (10% MeOH in CH_2_Cl_2_).

^1^H NMR (400 MHz, DMSO‐*d*_6_) *δ* 13.16 (br, s, 1H, NH), 8.60 (d, *J* = 8.6 Hz, 1H), 8.31 (d, *J* = 7.7 Hz, 2H), 8.12 (d, *J* = 8.1 Hz, 1H), 7.79–7.75 (m, 1H), 7.73–7.67 (m, 2H), 7.61 (d, *J* = 1.2 Hz, 1H), 7.10 – 7.03 (m, 3H), 6.18 (d, *J* = 1.0 Hz, 2H) ppm. ^13^C NMR (101 MHz, DMSO‐*d*_6_) *δ* 166.4, 165.5, 147.8, 147.3, 135.2, 134.8, 133.8, 129.0, 128.3, 128.2, 127.6, 127.1, 125.6, 124.8, 124.6, 122.83, 122.78, 120.4, 114.8, 110.8, 102.0 ppm. IR υ_max_ (cm^−1^): 3066, 2948, 2791, 1708, 1562, 1300, 1158, 1123. Mass spectral analysis: LRMS (ESI‐) *m/z*: 437 (M‐H, C_21_H_13_N_2_O_5_S_2_, 100%).

##### (Z)‐N‐(5‐((2,3‐dihydrobenzofuran‐5‐yl)methylene)‐4‐oxo‐4,5‐dihydrothiazol‐2‐yl)naphthalene‐1‐sulfonamide 57

Synthesized using General Procedure 2 with **5** (100 mg, 0.33 mmol, 1 eq.) and 2,3‐dihydrobenzo[*b*]furan‐5‐carbaldehyde (66 mg, 0.44 mmol, 1.1 eq.) for 20 min to give the desired product as a light orange/pink solid (87 mg, 61%). M.P: >259 °C (dec). R_f_ 0.57 (10% MeOH in CH_2_Cl_2_).

^1^H NMR (400 MHz, DMSO‐*d*_6_) *δ* 8.60 (d, *J* = 8.7 Hz, 1H), 8.31–8.28 (m, 2H), 8.12 (d, *J* = 8.1 Hz, 1H), 7.75 (dd, *J* = 8.5, 1.4 Hz, 1H), 7.72–7.68 (m, 3H), 7.54 (s, 1H), 7.47 (dd, *J* = 8.4, 1.7 Hz, 1H), 6.99 (d, *J* = 8.4 Hz, 1H), 4.65 (t, *J* = 8.7 Hz, 2H), 3.00 (m, 2H) ppm. NH exchanging—not observed. ^13^C NMR (101 MHz, DMSO‐*d*_6_) *δ* 162.4, 135.5, 134.6, 134.2, 133.8, 132.2, 129.4, 129.0, 128.2, 128.0, 127.6 (2C), 127.3, 127.1, 125.4, 124.9, 124.6, 110.1, 72.1, 28.6 ppm. Two carbons not observed. IR υ_max_ (cm^−1^): 3114, 2972, 2778, 1684, 1549, 1489, 1157, 1127. Mass spectral analysis: LRMS (ESI‐) *m/z*: 435 (M‐H, C_22_H_15_N_2_O_4_S_2_, 100%); (ESI+) *m/z*: 437 (M + H, C_22_H_17_N_2_O_4_S_2_, 100%).

##### (Z)‐N‐(5‐(benzo[c][1,2,5]thiadiazol‐5‐ylmethylene)‐4‐oxo‐4,5‐dihydrothiazol‐2‐yl)naphthalene‐1‐sulfonamide 58

Synthesized using General Procedure 2 with **5** (147 mg, 0.49 mmol, 1 eq.) and benzothiadiazole‐5‐carbaldehyde (90 mg, 0.54 mmol, 1.1 eq.) for 1 h. The resulting solid was collected and purified by column chromatography using a gradient solvent up to 10% MeOH in CH_2_Cl_2_to give the desired product as a brown powder (68 mg, 31%). M.P: >221 °C (dec). R_f_ 0.34 (10% MeOH in CH_2_Cl_2_).

^1^H NMR (400 MHz, DMSO‐*d*_6_) *δ* 8.63–8.61 (m, 1H), 8.42 (s, 1H), 8.34–8.28 (m, 3H), 8.12 (d, *J* = 8.0 Hz, 1H), 7.99 (s, 1H), 7.97 (dd, *J* = 9.2, 1.7 Hz, 1H), 7.78–7.76 (m, 1H), 7.73–7.69 (m, 2H) ppm. NH exchanging—not observed. ^13^C NMR (101 MHz, DMSO‐*d*_6_) *δ* 166.7, 165.3, 154.3, 154.2, 135.2, 134.8, 134.6, 133.8, 132.2, 130.1, 129.0, 128.3, 128.2, 127.6, 127.1, 125.0, 124.8, 124.7, 123.5, 122.3 ppm. IR υ_max_ (cm^−1^): 3056, 2939, 2771, 1716, 1557, 1325, 1124. Mass spectral analysis: LRMS (ESI‐) *m/z*: 451 (M‐H, C_20_H_11_N_4_O_3_S_3_, 100%).

##### (Z)‐N‐(5‐((1H‐indol‐5‐yl)methylene)‐4‐oxo‐4,5‐dihydrothiazol‐2‐yl)naphthalene‐1‐sulfonamide 59

Synthesized using General Procedure 2 with **5** (146 mg, 0.49 mmol, 1 eq.) and indole‐5‐carboxaldehyde (84 mg, 0.54 mmol, 1.1 eq.) for 50 min to give the desired product as an orange solid (104 mg, 48%). M.P: >186 °C (dec).

^1^H NMR (400 MHz, DMSO‐*d*_6_) *δ* 11.56 (br, s, 1H, NH), 8.62 (d, *J* = 8.5 Hz, 1H), 8.33–8.29 (m, 2H), 8.12 (d, *J* = 8.0 Hz, 1H), 7.92 (d, *J* = 15.3 Hz, 1H), 7.88 (s, 1H), 7.77 (t, *J* = 7.27 Hz, 1H), 7.73–7.76 (m, 2H), 7.62 (d, *J* = 8.5 Hz, 1H), 7.51–7.50 (m, 1H), 7.43–7.41 (m, 1H), 6.64 (s, 1H) ppm. NH exchanging—not observed. ^13^C NMR (101 MHz, DMSO‐*d*_6_) *δ* 166.7, 166.0, 137.2, 136.6, 135.4, 134.6, 133.8, 129.0, 128.29, 128.25 (2C), 128.1, 127.7, 127.1, 124.9, 124.7, 124.5, 123.8, 123.3, 116.8, 112.7, 102.6 ppm. IR υ_max_ (cm^−1^): 3340, 2985, 2784, 1687, 1559, 1289, 1116. Mass spectral analysis: LRMS (ESI‐) *m/z* (%): 432 (M‐H, C_22_H_14_N_3_O_3_S_2_, 100%).

##### (Z)‐N‐(5‐((1H‐indol‐3‐yl)methylene)‐4‐oxo‐4,5‐dihydrothiazol‐2‐yl)naphthalene‐1‐sulfonamide 60

Synthesized using General Procedure 2 with **5** (150 mg, 0.49 mmol, 1 eq.) and indole‐3‐carboxaldehyde (78 mg, 0.54 mmol, 1.1 eq.) for 40 min to give the desired product as a yellow solid (63 mg, 30%). M.P: >298 °C (dec).

^1^H NMR (400 MHz, DMSO‐*d*_6_) *δ* 12.93 (br, s, 1H, NH), 12.23 (br, s, 1H, NH), 8.63 (d, *J* = 8.5 Hz, 1H), 8.31 (dd, *J* = 20.3, 7.7 Hz, 2H,), 8.11 (d, *J* = 8.0 Hz, 1H), 8.02 (s, 1H), 7.91–7.89 (m, 2H), 7.76 (t, *J* = 7.5 Hz, 1H), 7.72–7.66 (m, 2H), 7.55 (d, *J* = 8.0 Hz, 1H), 7.27 (t, *J* = 7.4 Hz, 1H), 7.21 (t, *J* = 7.3 Hz, 1H) ppm. ^13^C NMR (151 MHz, DMSO‐*d*_6_) *δ* 166.5, 165.7, 136.4, 135.7, 134.5, 133.8, 129.7, 129.0, 128.2, 128.1, 127.7, 127.0, 126.8, 126.4, 125.0, 124.6, 123.2, 121.3, 118.5, 112.6, 110.4 ppm. One quaternary carbon not observed. IR υ_max_ (cm^−1^): 3295, 3108, 2926, 2765, 1684, 1551, 1283, 1122. Mass spectral analysis: LRMS (ESI‐) *m/z* (%): 432 (M‐H, C_22_H_14_N_3_O_3_S_2_, 100%).

##### (Z)‐N‐(5‐((1‐methyl‐1H‐indol‐3‐yl)methylene)‐4‐oxo‐4,5‐dihydrothiazol‐2‐yl)naphthalene‐1‐sulfonamide 61

Synthesized using General Procedure 2 with **5** (156 mg, 0.49 mmol, 1 eq.) and 1‐methylindole‐3‐carboxaldehyde (97 mg, 0.55 mmol, 1.1 eq.) for 50 min to give the desired product as a yellow solid (213 mg, 97%). M.P: >289 °C (dec).

^1^H NMR (400 MHz, DMSO‐*d*_6_) *δ* 12.92 (br, s, 1H, NH), 8.63 (d, *J* = 8.6 Hz, 1H), 8.33 (dd, *J* = 22.2, 7.7 Hz, 2H), 8.12 (d, *J* = 8.0 Hz, 1H), 8.02 (s, 1H), 8.96–7.92 (m, 2H), 7.79–7.67 (m, 3H), 7.60 (d, *J* = 8.1 Hz, 1H), 7.34 (t, *J* = 7.5 Hz, 1H), 7.26 (t, *J* = 7.4 Hz, 1H), 4.00 (s, 3H) ppm. ^13^C NMR (101 MHz, DMSO‐*d*_6_) *δ* 166.3, 165.3, 137.0, 135.5, 134.6, 133.8, 133.1, 129.0, 128.2, 128.1, 127.7, 127.3, 127.1, 126.1, 124.9, 124.6, 123.3, 121.7, 118.6, 113.6, 111.0, 109.3, 33.4 ppm. IR υ_max_ (cm^−1^): 2913, 2758, 1684, 1521, 1299, 1122. Mass Spectral analysis: LRMS (ESI‐) *m/z* (%): 447 (M‐H, C23H16N3O3S2, 100%).

##### (Z)‐N‐(5‐((2‐methyl‐1H‐indol‐3‐yl)methylene)‐4‐oxo‐4,5‐dihydrothiazol‐2‐yl)naphthalene‐1‐sulfonamide 62

Synthesized using General Procedure 2 with **5** (147 mg, 0.49 mmol, 1 eq.) and 2‐methylindole‐3‐carboxaldehyde (88 mg, 0.54 mmol, 1.1 eq.) for 50 min to give the desired product as a bright yellow solid (56 mg, 25%). M.P: >301 °C (dec).

^1^H NMR (400 MHz, DMSO‐*d*_6_) *δ* 12.89 (br, s, 1H, NH), 12.16 (br, s, 1H, NH), 8.60 (d, *J* = 8.4 Hz, 1H), 8.30–8.23 (m, 2H), 8.11 (d, *J* = 7.7 Hz, 1H), 7.92 (s, 1H), 7.76–7.68 (m, 4H), 7.44 (d, *J* = 6.7 Hz, 1H), 7.24 (s, 2H), 2.53 (s, 3H) ppm. ^13^C NMR (101 MHz, DMSO‐*d*_6_) *δ* 166.5, 165.4, 144.4, 136.2, 135.4, 134.5, 133.8, 129.7, 129.0, 128.2 (2C), 127.7, 127.0, 125.0, 124.7, 124.5, 122.5, 121.0, 119.4, 113.0, 112.0, 107.3, 12.4 ppm. IR υ_max_ (cm^−1^): 3334, 2972, 2881, 2758, 1690, 1539, 1329, 1115. Mass Spectral analysis: LRMS (ESI‐) *m/z* (%): 446 (M‐H, C_23_H_16_N_3_O_3_S_2_, 100%).

##### (Z)‐N‐(5‐((5‐methyl‐1H‐indol‐3‐yl)methylene)‐4‐oxo‐4,5‐dihydrothiazol‐2‐yl)naphthalene‐1‐sulfonamide 63

Synthesized using General Procedure 2 with **5** (147 mg, 0.49 mmol, 1 eq.) and 5‐methylindole‐3‐carboxaldehyde (90 mg, 0.55 mmol, 1.1 eq.) for 50 min to give the desired product as a bright orange solid (103 mg, 46%). M.P: >278 °C (dec).

^1^H NMR (400 MHz, DMSO‐*d*_6_): *δ* 12.91 (br, s, 1H, NH), 12.15 (br, s, 1H, NH), 8.63 (d, *J* = 8.6 Hz, 1H), 8.34 (dd, *J* = 7.35, 1.06 Hz, 1H), 8.29 (d, *J* = 8.29 Hz, 1H), 8.12 (d, *J* = 8.1 Hz, 1H), 8.01 (s, 1H), 7.84 (d, *J* = 3.0 Hz, 1H), 7.79–7.75 (m, 1H), 7.72–7.66 (m, 3H), 7.43 (d, *J* = 8.3 Hz, 1H), 7.09 (dd, *J* = 8.3, 1.0 Hz, 1H), 2.42 (s, 3H) ppm. ^13^C NMR (101 MHz, DMSO‐*d*_6_) *δ* 166.8, 165.8, 136.0, 135.1, 135.0, 134.3, 130.8, 130.2, 129.5, 128.7, 128.5, 128.1, 127.6, 127.5, 127.2, 125.4, 125.3, 125.1, 118.5, 113.8, 112.8, 110.4, 21.7 ppm. IR υ_max_ (cm^−1^): 3321, 2907, 2752, 1697, 1558, 1322, 1126. Mass spectral analysis: LRMS (ESI‐) *m/z* (%): 446 (M‐H, C_23_H_16_N_3_O_3_S_2_, 100%).

##### (Z)‐N‐(5‐((6‐bromo‐1H‐indol‐3‐yl)methylene)‐4‐oxo‐4,5‐dihydrothiazol‐2‐yl)naphthalene‐1‐sulfonamide 64

Synthesized using General Procedure 2 with **5** (157 mg, 0.49 mmol, 1 eq.) and 6‐bromoindole‐3‐carboxaldehyde (120 mg, 0.54 mmol, 1.1 eq.) for 50 min to give the desired product as a yellow solid (114 mg, 45%). M.P: >297 °C (dec).

^1^H NMR (400 MHz, DMSO‐*d*_6_) *δ* 12.96 (br, s, 1H, NH), 12.27 (br, s, 1H, NH), 8.63 (d, *J* = 8.5 Hz, 1H), 8.32 (dd, *J* = 18.8, 7.7 Hz, 2H), 8.11 (d, *J* = 8.0 Hz, 1H), 8.02 (s, 1H), 7.91 (dd, *J* = 14.4, 5.3 Hz, 2H), 7.79 – 7.67 (m, 4H), 7.32 (d, *J* = 8.2 Hz, 1H) ppm. ^13^C NMR (101 MHz, DMSO‐*d*_6_) *δ* 166.2, 165.2, 137.2, 135.4, 134.6, 133.8, 130.0, 129.0, 128.3, 128.1, 127.7, 127.1, 126.1, 125.8, 124.9, 124.6, 124.1, 120.5, 115.8, 115.2, 114.9, 110.4 ppm. IR υ_max_ (cm^−1^): 3289, 2901, 2745, 1687, 1549, 1284, 1113, 709. Mass spectral analysis: LRMS (ESI‐) *m/z* (%): 512 (M‐H, C_22_H_13_^81^BrN_3_O_3_S_2_, 100), 510 (M‐H, C_22_H_13_^79^BrN_3_O_3_S_2_, 90); (ESI+) *m/z* (%): 514 (M + H, C_22_H_15_^81^BrN_3_O_3_S_2_, 40), 512 (M + H, C_22_H_15_^79^BrN_3_O_3_S_2_, 30). HRMS: Exact mass calculated for C_22_H_13_^79^BrN_3_O_3_S_2_ [M‐H]^−^, 509.9583. Found 509.9586. Exact mass calculated for C_22_H_13_^81^BrN_3_O_3_S_2_ [M‐H]^−^ 511.9563; Found 511.9564.

##### (Z)‐N‐(5‐((5‐bromo‐1H‐indol‐3‐yl)methylene)‐4‐oxo‐4,5‐dihydrothiazol‐2‐yl)naphthalene‐1‐sulfonamide 65

Synthesized using General Procedure 2 with **5** (149 mg, 0.49 mmol, 1 eq.) and 5‐bromoindole‐3‐carboxaldehyde (127 mg, 0.54 mmol, 1.1 eq.) for 50 min to give the desired product as a yellow solid (105 mg, 42%). M.P: >309 °C (dec).

^1^H NMR (400 MHz, DMSO‐*d*_6_) *δ* 12.94 (br, s, 1H, NH), 12.35 (br, s, 1H, NH), 8.63 (d, *J* = 8.5 Hz, 1H), 8.34 (d, *J* = 7.32 Hz, 1H), 8.29 (d, *J* = 8.21 Hz, 1H), 8.20 (s, 1H), 8.12 (d, *J* = 8.1 Hz, 1H), 8.07 (s, 1H), 7.94 (d, *J* = 2.7 Hz, 1H), 7.77 (t, *J* = 7.2 Hz, 1H), 7.70 (q, *J* = 7.5 Hz, 2H), 7.51 (d, *J* = 8.6 Hz, 1H), 7.38 (dd, *J* = 8.6, 1.7 Hz, 1H) ppm. ^13^C NMR (101 MHz, DMSO‐*d*_6_) *δ* 165.4 (br), 135.6, 135.1, 134.5, 133.8, 130.7, 129.0, 128.6, 128.2, 128.0, 127.7, 127.1, 126.3 (br), 125.8, 124.9, 124.6, 121.3, 114.6, 114.0, 110.1 ppm. Two quaternary carbons not observed. IR υ_max_ (cm^−1^): 3392, 3192, 1700, 1558, 1290, 1108, 565. Mass spectral analysis: LRMS (ESI‐) *m/z* (%): 510 (M‐H+H, C_22_H_14_^79^BrN_3_O_3_S_2_, 80%), 512 (M‐H+H, C_22_H_14_^81^BrN_3_O_3_S_2_, 100%).

##### (Z)‐N‐(5‐((5‐chloro‐1H‐indol‐3‐yl)methylene)‐4‐oxo‐4,5‐dihydrothiazol‐2‐yl)naphthalene‐1‐sulfonamide 66

Synthesized using General Procedure 2 with **5** (159 mg, 0.49 mmol, 1 eq.) and 5‐chloroindole‐3‐carboxaldehyde (109 mg, 0.54 mmol, 1.1 eq.) for 50 min to give the desired product as a yellow solid (66 mg, 29%). M.P: >306 °C (dec).

^1^H NMR (400 MHz, DMSO‐*d*_6_) *δ* 12.95 (br, s, 1H, NH), 12.35 (br, s, 1H, NH), 8.63 (d, *J* = 8.5 Hz, 1H), 8.35 – 8.29 (m, 2H), 8.12 (d, *J* = 8.1 Hz, 1H), 8.07–8.06 (m, 2H), 7.96 (d, *J* =  2.9 Hz, 1H), 7.79 – 7.75 (m, 1H), 7.73–7.67 (m, 2H), 7.56 (d, *J* = 8.7 Hz, 1H), 7.27 (dd, *J* = 8.7, 1.9 Hz, 1H) ppm. ^13^C NMR (101 MHz, DMSO‐*d*_6_) *δ* 166.2, 165.2, 135.5, 134.8, 134.6, 133.8, 130.9, 129.0, 128.3, 128.1, 128.0, 127.7, 127.1, 126.4, 126.1, 124.9, 124.6, 123.3, 118.3, 114.7, 114.2, 110.1 ppm. IR υ_max_ (cm^−1^): 3386, 3185, 1703, 1558, 1312, 1108, 702. Mass spectral analysis: LRMS (ESI‐) *m/z* (%): 466 (M‐H, C_22_H_13_^35^ClN_3_O_3_S_2_, 100%), 468 (M‐H, C_22_H_13_^37^ClN_3_O_3_S_2_, 35%).

##### (Z)‐N‐(5‐(benzofuran‐3‐ylmethylene)‐4‐oxo‐4,5‐dihydrothiazol‐2‐yl)naphthalene‐1‐sulfonamide 67

Synthesized using General Procedure 2 with **5** (200 mg, 0.65 mmol, 1 eq.) and 2‐benzofurancarboxaldehyde (0.1 mL, 0.72 mmol, 1.1 eq.) for 30 min to give the desired product as a bright yellow solid (213 mg, 75%). M.P: >279 °C (dec). R_f_ 0.25 (10% MeOH in CH_2_Cl_2_).

^1^H NMR (400 MHz, DMSO‐*d*_*6*_) *δ* 13.16 (broad, 1H, NH), 8.62 (d, *J* = 8.7 Hz, 1H), 8.34 (dd, *J* = 7.4, 1.1 Hz, 1H), 8.31 (d, *J* = 8.3 Hz, 1H), 8.12 (d, *J* = 8.0 Hz, 1H), 7.81–7.67 (m, 6H), 7.59 (s, 1H), 7.52–7.48 (m, 1H), 7.38–7.34 (m, 1H) ppm. ^13^C NMR (101 MHz, DMSO‐*d*_*6*_) *δ* 166.5, 166.3, 155.5, 150.8, 135.4, 134.7, 133.8, 129.0, 128.3, 128.1, 127.8, 127.7, 127.6, 127.1, 124.9, 124.7, 124.1, 122.7, 122.1, 120.0, 115.4, 111.6 ppm. IR υ_max_ (cm^−1^): 3117, 2943, 1690, 1557, 1335, 1125. Mass spectral analysis: LRMS (ESI‐) *m/z* (%): 433 (M‐H, C_22_H_13_N_2_O_4_S_2_, 100%).

##### (Z)‐N‐(4‐oxo‐5‐((1‐(phenylsulfonyl)‐1H‐indol‐3‐yl)methylene)‐4,5‐dihydrothiazol‐2‐yl)naphthalene‐1‐sulfonamide 68

Synthesized using General Procedure 2 with **5** (150 mg, 0.49 mmol, 1 eq.) and 1‐phenylsulfonyl‐3‐indole carboxaldehyde (156 mg, 0.54 mmol, 1.1 eq.) for 10 min to give the desired product as a bright yellow solid (210 mg, 75%). M.P: >300 °C (dec).

^1^H NMR (400 MHz, DMSO‐*d*_6_) *δ* 13.17 (br, s, 1H, NH), 8.63 (d, *J* = 8.5 Hz, 1H), 8.34 (t, *J* = 7.0 Hz, 2H), 8.15 (t, *J* = 7.9 Hz, 3H), 8.08 (s, 1H), 7.98 (dd, *J* = 14.7, 8.1 Hz, 2H), 7.92 (s, 1H), 7.80–7.70 (m, 4H), 7.68 – 7.63 (m, 2H), 7.48 (t, *J* = 7.5 Hz, 1H), 7.39 (t, *J* = 7.5 Hz, 1H) ppm. ^13^C NMR (101 MHz, DMSO‐*d*_6_) *δ* 166.0, 164.6, 136.2, 135.6, 135.2, 134.8, 133.81, 133.79, 130.1 (2C), 129.0, 128.7, 128.4, 128.2, 127.6, 127.2 (2C), 127.1 (2C), 126.4, 124.8, 124.7, 124.5, 122.9, 122.8, 120.3, 116.6, 113.3 ppm. IR υ_max_ (cm^−1^): 3120, 3049, 2965, 2784, 1603, 1551, 1363, 1306, 1124. Mass spectral analysis: LRMS (ESI‐) *m/z* (%): 572 (M‐H, C_28_H_18_N_3_O_5_S_2_, 100%).

## Conflict of Interest

The authors declare no conflict of interest.

## Author Contributions

**Kate Prichard**: compound synthesis, characterisation and modeling. **Mark J Robertson**, **Andre Horatscheck**: compound synthesis. **Ngoc Chau** and **Jing Xue**: in cell CME and dynamin assays. **Martin Neuenschwander** and **Volker Haucke** clathrin ELISA. **Phillip J Robinson**, **Volker Haucke** and **Adam McCluskey**: project initiation, funding, direction, manuscript preparation and editing.

## Supporting information

Supplementary Material

## Data Availability

The data that support the findings of this study are available in the supplementary material of this article.
