# Supplementary material for: Inhibition of the Clathrin Terminal Domain—Amphiphysin Protein–Protein Interaction. Probing the Pitstop 2 Aromatic Moiety
Source: ChemMedChem. 2025 Jul 11;20(16):e202500321. doi: 10.1002/cmdc.202500321 (PMC12368479; doi:10.1002/cmdc.202500321)

Electronic Supporting Information

Supplementary Figures

Figure S1. Docked poses of *Library 1* analogues, **20** – **23** in the CTD, Site 1.

Figure S2. Docked poses, and ligand interaction maps, of *Library 1* selected analogues, **6**, **13** and **18** in the CTD, Site 1.

Figure S3. Docked poses of *Library 2* analogues, **25**, **30**, **35** and **36** in the CTD, Site 1.

Figure S4. Docked poses, and ligand interaction maps, of *Library 2* dihydroxy analogues, **24**, **25** and **27** in the CTD, Site 1.

Figure S5. Docked poses of *Library 3* analogues, **39** – **43** in the CTD, Site 1.

Figure S6. Docked poses of *Library 4* analogues, **44** – **51** in the CTD, Site 1.

Figure S7. Docked poses of *Library 4* analogues, **61** – **63** in the CTD, Site 1.

Figure S8. Docked poses of *Library 4* halogen substituted indoles analogues, **60**, **64** and **65** in the CTD, Site 1.

Figure S9. Docked poses, and ligand interaction maps, of *Library 4* selected heterocyclic analogues, **54** and **56** in the CTD, Site 1.

Table S1. 2-point CME data for selected analogues from *Library 3* and *Library 4*, along with corresponding ELISA and Dynamin 1 IC_{50} values. 2-point data shows the percentage inhibition of transferrin uptake in U2OS cells at 5 and 50 μM.

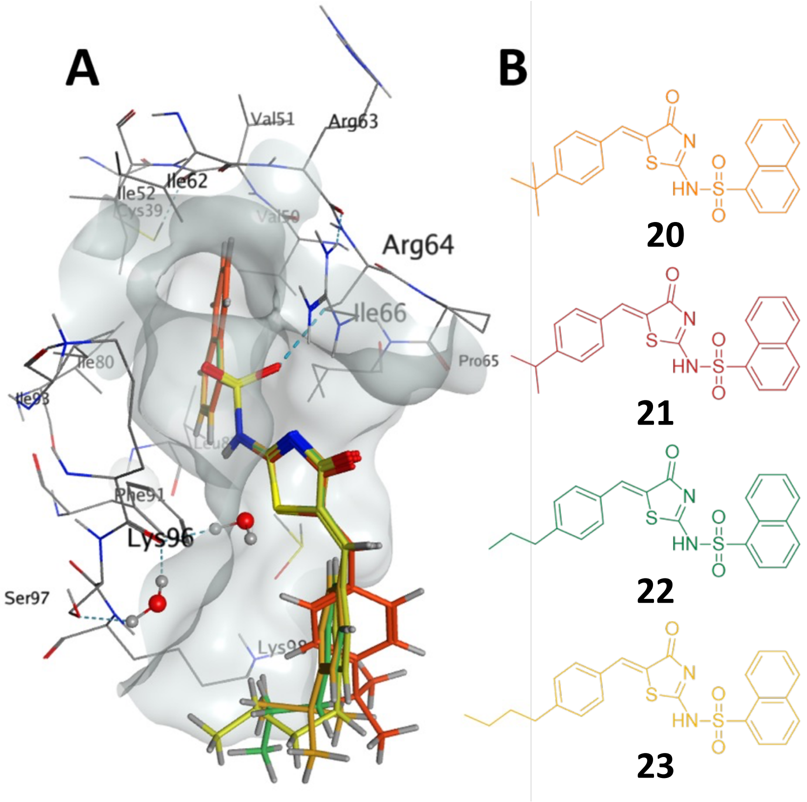

Figure S1. Predicted binding of aliphatic substituted analogues from *Library 1* docked into the CTD (Site 1). PDB: 4G55.[2] A. 4-*tert*-butylphenyl **20** (Orange; NTD-PPI IC_{50} 4.6 μM), 4-isopropylphenyl **21** (Red; NTD-PPI IC_{50} 4.1 μM), 4-propylphenyl **22** (Green; IC_{50} 4 μM) and 4-butylphenyl **23** (Yellow NTD-PPI IC_{50} 3.5 μM) docked into the CTD. **B**. Chemical structures of **20-23**.

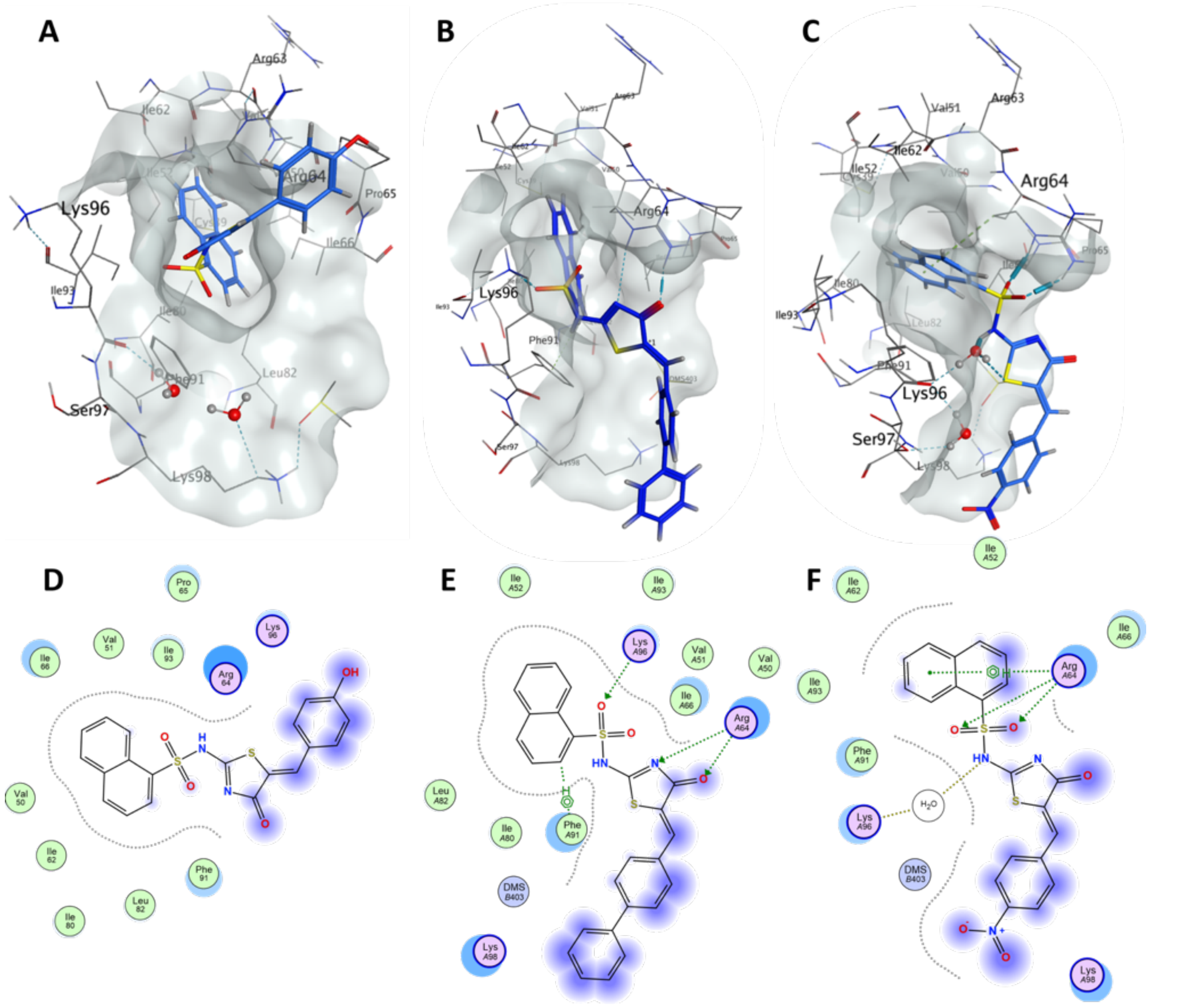

Figure S2. Predicted binding of selected substituted analogues from *Library 1* docked into the CTD (Site 1). PDB: 4G55.[2] A. Predicted binding pose of 4-OH **6** (NTD-PPI IC_{50} 2.7 μM). B. Predicted binding pose 4-Ph **18** (NTD-PPI IC_{50} 1.4 μM). C. Predicted binding pose of 4-NO_2 **13** (NTD-PPI IC_{50} 5.2 μM). **D**. MOE generated ligand map of 4-phenoxy **6**. **E**. MOE generated ligand map of 4-biphenyl **18**, showing interactions with Arg64, Lys96 and Phe91. **F**. MOE generated ligand map of 4-nitrophenyl **13**, showing interactions with Lys96 (water-mediated) and Arg64.

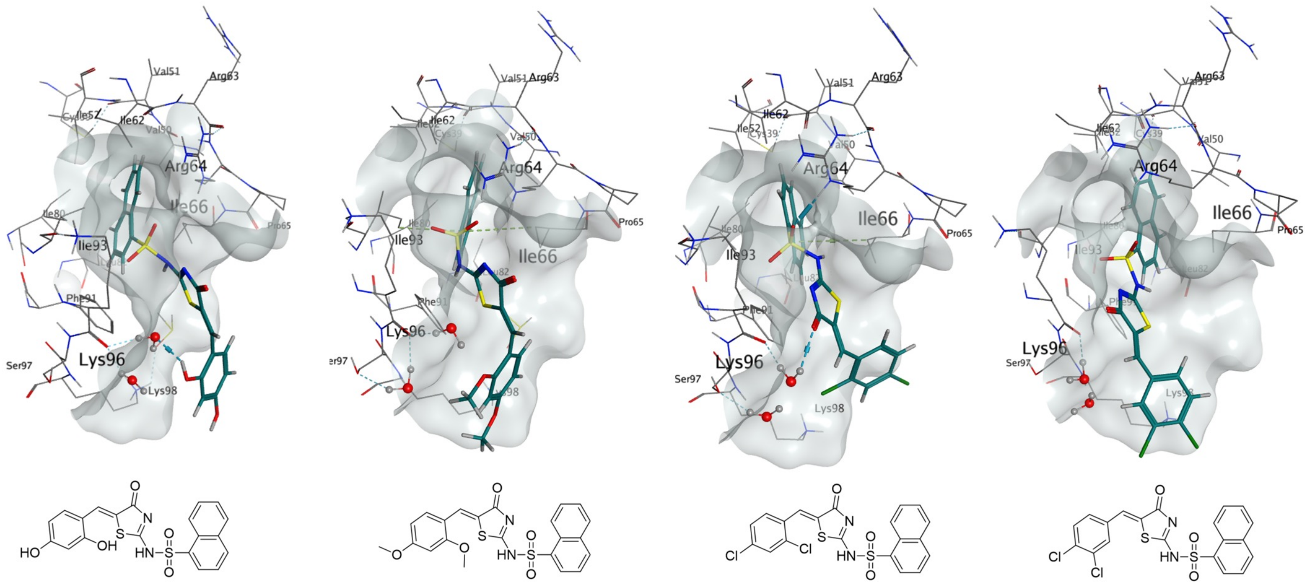

Figure S3. Predicted binding of 2,4-disubstituted analogues from *Library 2* docked into the CTD (Site 1). PDB: 4G55.[2] **A**. 2, 4-dihydroxyphenyl **25** (NTD-PPI IC_{50} 1.8 μM); **B**. 2,4-dimethoxyphenyl **30** (NTD-PPI IC_{50} 1.0 μM); **C**. 2,4-dichlorophenyl **35** (Green; NTD-PPI IC_{50} 1.5 μM) docked into the CTD; and D. 3,4-dichlorophenyl **36** (NTD-PPI IC_{50} 4.1 μM).

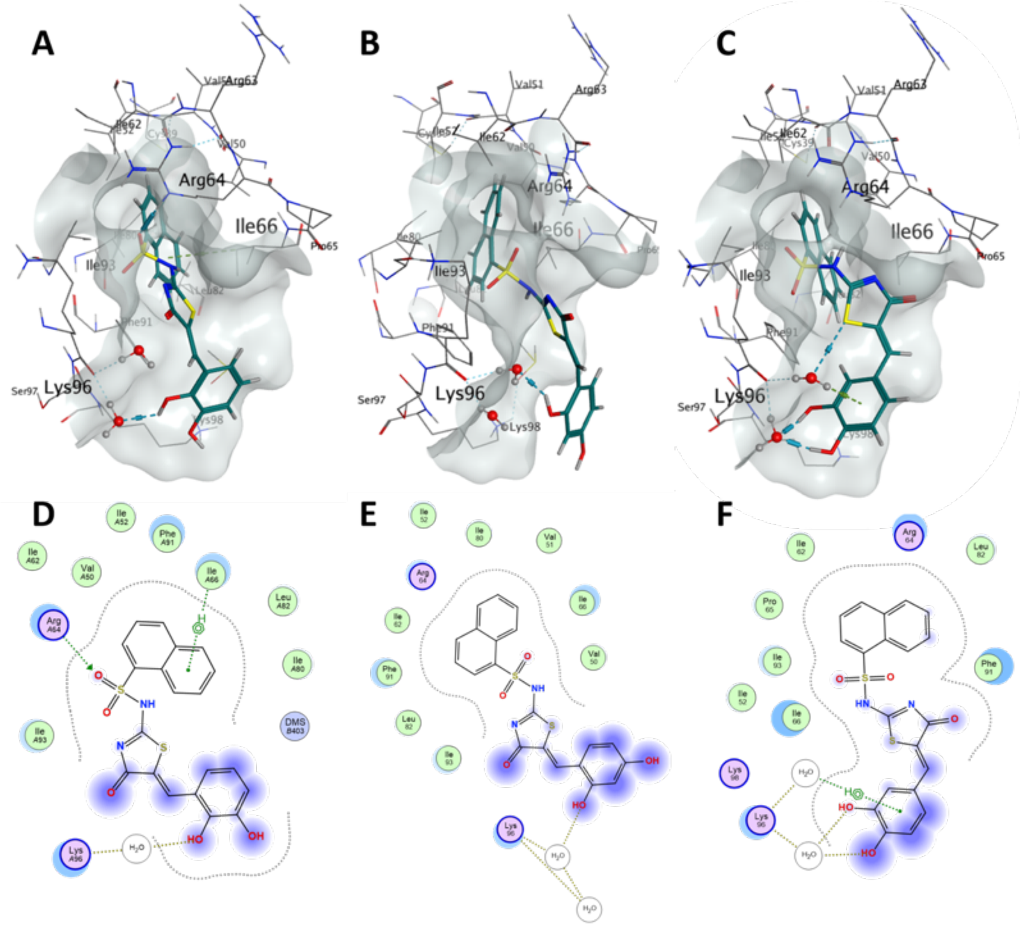

Figure S4. Predicted binding of selected di- hydroxy substituted analogues from *Library 2* docked into the CTD (Site 1). PDB: 4G55.[2] **A.** Predicted binding pose of 3,4-dihydroxyphenyl **27** (NTD-PPI IC_{50} 1.6 μM). **B.** Predicted binding pose of 2,4-dihydroxyphenyl **25** (NTD-PPI IC_{50} 1.8 μM). **C.** Predicted binding pose of 2,3-dihydroxyphenyl **28** (NTD-PPI IC_{50} 6.3 μM). **D.** MOE generated ligand map of 3,4-dihydroxyphenyl **27** showing interactions with Ile66, Arg64 and Lys96 (water-mediated). **E.** MOE generated ligand map of 2,4-dihydroxyphenyl **25**, showing an interaction with Lys96 (water-mediated). **F.** MOE generated ligand map of 2,3-dihydroxyphenyl **24**, showing interactions with Lys96 (water-mediated).

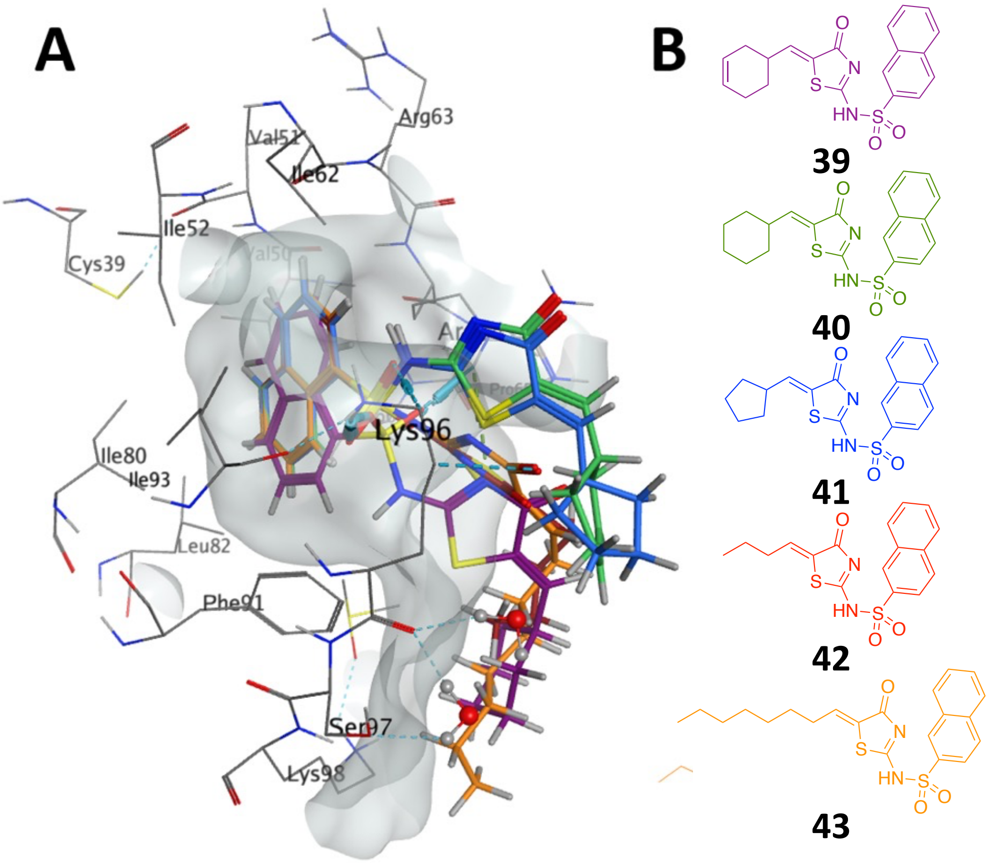

Figure S5. Predicted binding of aliphatic analogues from *Library 3* docked into the CTD (Site 1). PDB: 4G55.[2] **A**. Cyclohexene **39** (Purple; NTD-PPI IC_{50} 4.3 μM), cyclohexane **40** (Green; NTD-PPI IC_{50} 8.6 μM), cyclopentane **41** (Blue; NTD-PPI IC_{50} 5.5 μM), butane **42** (Red; NTD-PPI IC_{50} 6.5 μM) and heptane **43** (Orange; NTD-PPI IC_{50} 5.9 μM) docked into the CTD. **B**. Chemical structures of **39**-**43**.

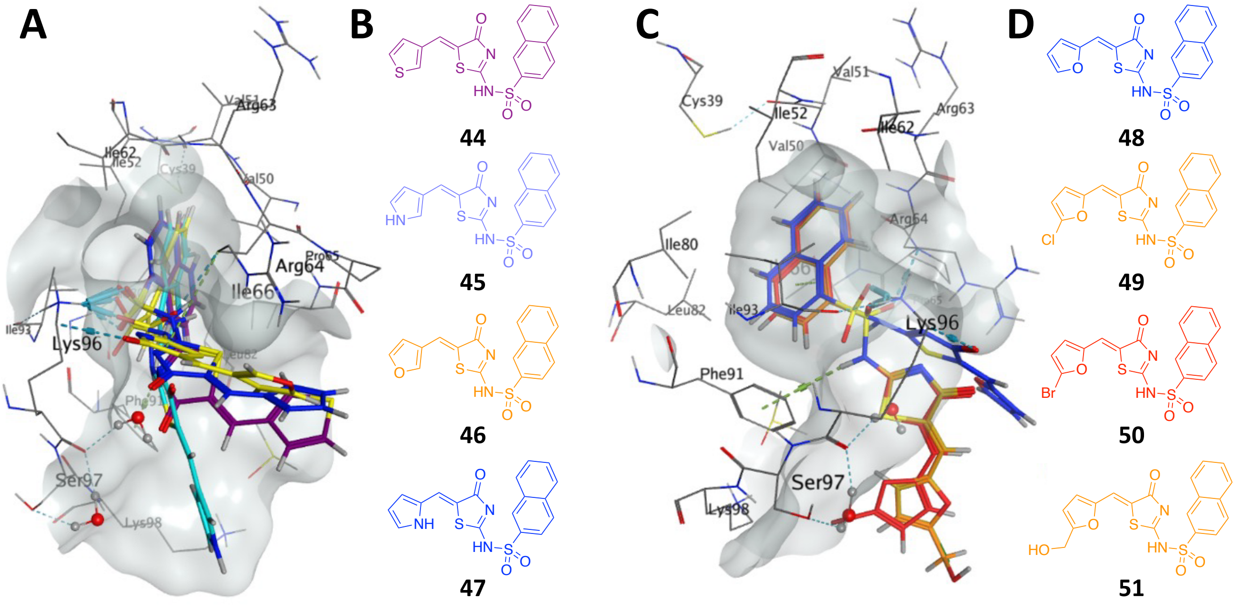

Figure S6. Predicted binding of 5-ring heterocycle analogues from *Library 4* docked into the CTD (Site 1). PDB: 4G55.[2] **A**. Thiophene **44** (NTD-PPI IC_{50} 2.3 μM), 3-pyrrole **45** (NTD-PPI IC_{50} 33.0 μM), 3-furan **46** (NTD-PPI IC_{50} 2.1 μM) and 2-pyrrole **47** (NTD-PPI IC_{50} 2.1 μM) docked into the CTD. **B**. Structures of thiophene **44** (purple), 3-pyrrole **45** (aqua), 3-furan **46** (yellow) and 2-pyrrole **47** (blue) in corresponding colours. **C**. 2-Furan **48** (NTD-PPI IC_{50} 3.6 μM), halogenated furans (chloro **49** and bromo **50**, NTD-PPI IC_{50} 3.3 and 2.9 μM respectively) and CH_2OH furan **51** (NTD-PPI IC_{50} 6.3 μM) docked into the CTD. **D**. Structures of 2-furan **48** (blue), halogenated furans (chloro **49** and bromo **50**, yellow and red respectively) and CH_2OH furan **51** (orange)) in corresponding colours.

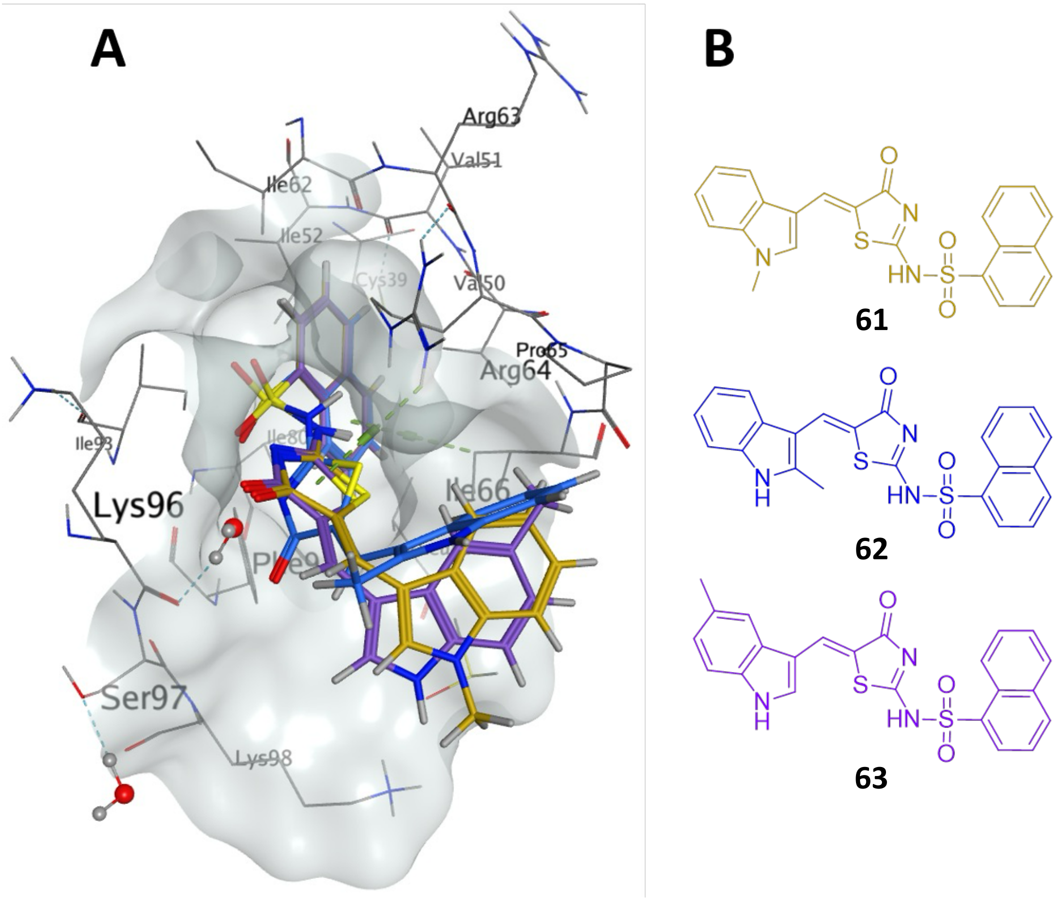

Figure S7. Predicted binding of methyl substituted indole analogues from *Library 4* docked into the CTD (Site 1). PDB: 4G55.[2] A. 1-methylindole **61** (Yellow; NTD-PPI IC_{50} 3 μM), 2-methylindole **62** (Blue; NTD-PPI IC_{50} 2.4 μM) and 5-methylindole **63** (Purple; NTD-PPI IC_{50} 3.5 μM) docked into the CTD. **B**. Chemical structures **61**-**63**.

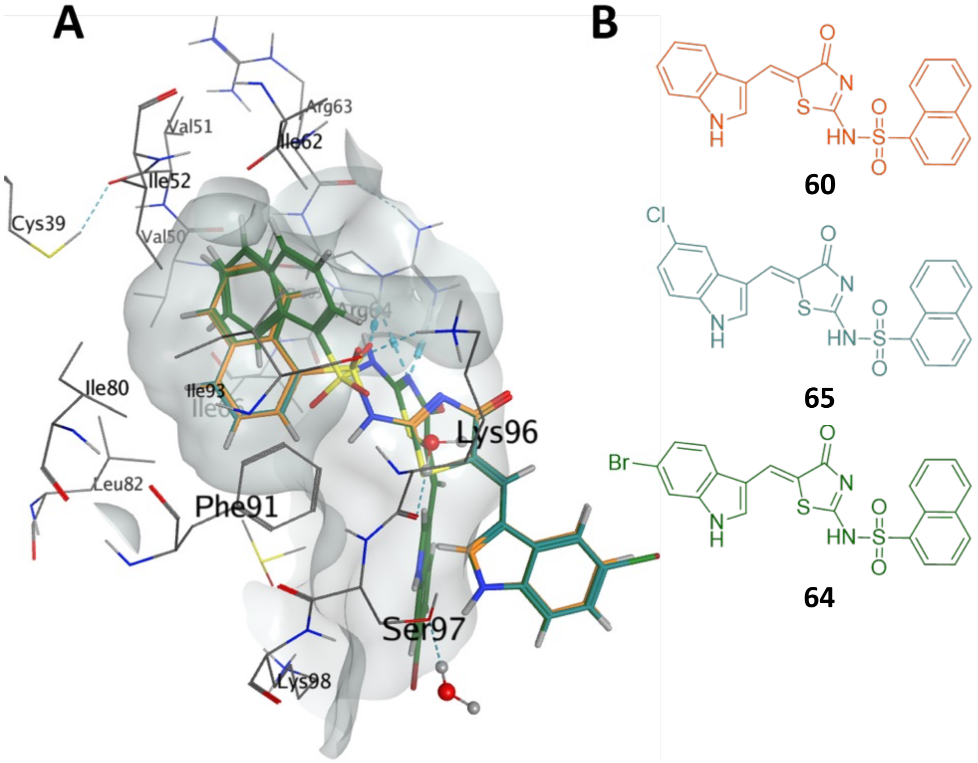

Figure S8. Predicted binding of halogen substituted indole analogues from *Library 4* docked into the CTD (Site 1). PDB: 4G55.[2] **A**. indole **60** (Orange; NTD-PPI IC_{50} 3.2 μM), 5-chloroindole **66** (Blue; NTD-PPI IC_{50} 2.8 μM) and 3-bromoindole **64** (Green; NTD-PPI IC_{50} 3.4 μM) docked into the CTD. **B**. Chemical structures of indoles **60**, **64** and **66**.

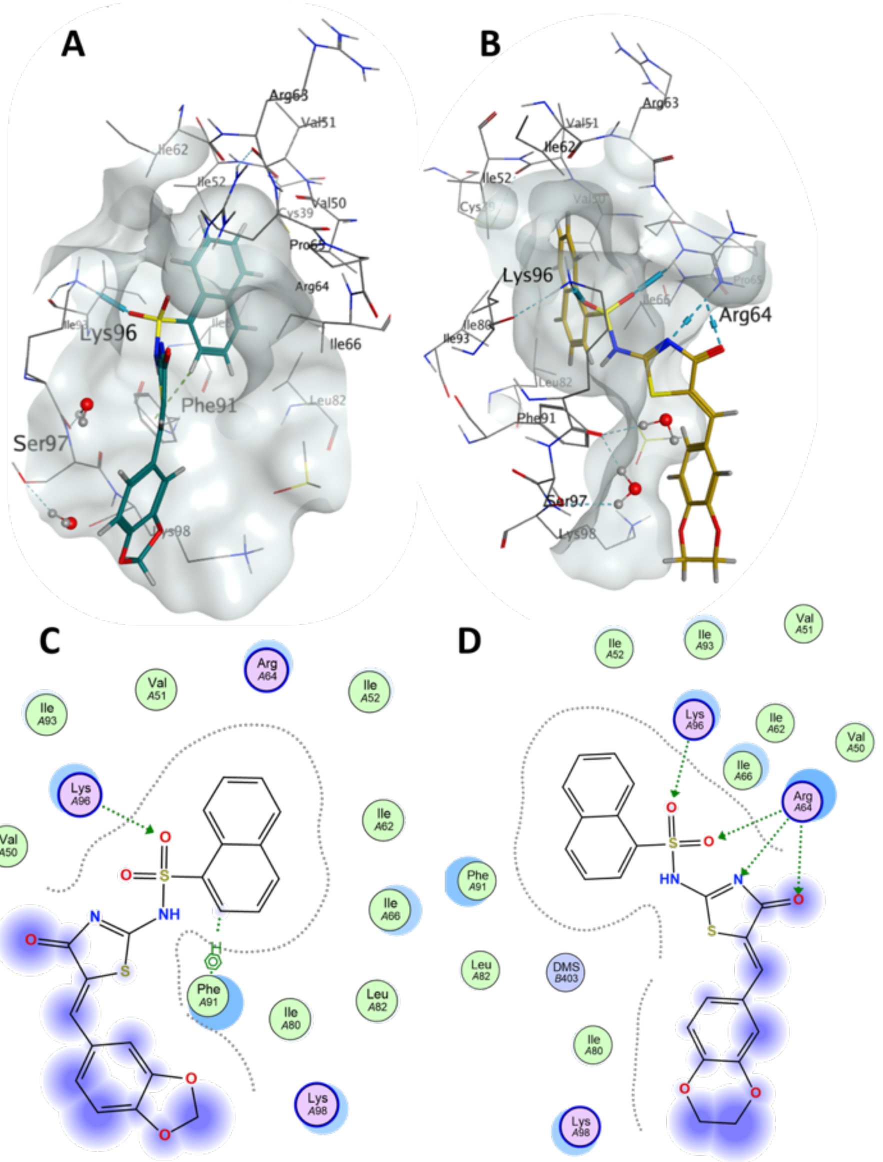

Figure S9. Predicted binding of selected heterocyclic analogues from *Library 4* docked into the CTD (Site 1). PDB: 4G55.[2] **A**. Predicted binding pose of methylenedioxy **56** (NTD-PPI IC_{50} 4 μM). **B**. Predicted binding pose of dihydrobenzo[*b*][1,4]dioxine **54** (NTD-PPI IC_{50} 1.2 μM). **C**. MOE generated ligand map of methylenedioxy **56,** showing interactions with Phe91 and Lys96. **D**. MOE generated ligand map of dihydrobenzo[*b*][1,4]dioxine **54**, showing interactions with Lys96 and Arg64.

Table S1. 2-point CME data for selected analogues from *Library 3* and *Library 4*, along with corresponding ELISA and Dynamin 1 IC_{50} values. 2-point data shows the percentage inhibition of transferrin uptake in U2OS cells at 5 and 50 μM.

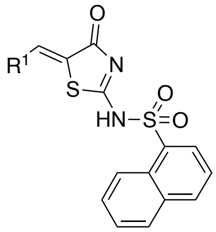

R	2-point CME (% Inhibition) 5 μM	2-point CME (% Inhibition) 50 μM	NTD-PPI IC_{50} (μM)[a]	Dynamin 1 IC_{50} (μM)[b]	R	2-point CME (% Inhibition) 5 μM	2-point CME (% Inhibition) 50 μM	NTD-PPI IC_{50} (μM)[a]	Dynamin 1 IC_{50} (μM)[b]
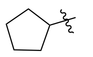 **41**	11	100[d]	5.5	NA [c,e]	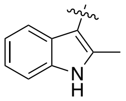 **62**	6	71	2.4	NA [c,e]
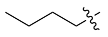 **42**	0	31	6.5	NA [c,e]	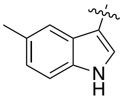 **63**	6	68	3.5	NA [c,e]
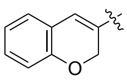 **55**	0	40	2.5	69.3 ± 7.1[e]	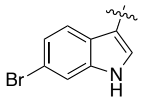 **64**	17	70	3.4	NA [c]
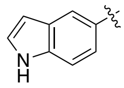 **59**	0	83	3.7	83.8 ± 10.7[e]	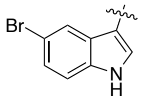 **65**	0	64	2.9	NA [c,e]
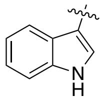 **60**	0	38	3.2	NA [c,e]	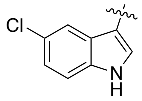 **66**	2	100[d]	2.8	NA [c]
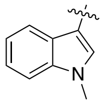 **61**	9	69	3	NA [c]	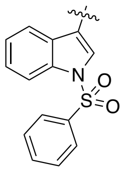 **68**	8	59	27	99.8 ± 3.1[e]

[a] NTD-PPI IC_{50} values represent the average of duplicate measurements (n=2); [b] Dynamin IC_{50} values are preliminary (n=1) unless otherwise noted; [c] Not active includes IC_{50} > 150 μM; [d] 100 indicates analogues that displayed an apparent 100% inhibition at 50 μM, but these cells were noted to be damaged, with no observable transferrin-signal. Cells membrane of these were also noted to be overstained and consequently over counted by the program; [e] Dynamin IC_{50} values represent the average of duplicate measurements (n=2).

Compound Name: (Z)-*N*-(5-(4-hydroxybenzylidene)-4-oxo-4,5-dihydrothiazol-2-yl)naphthalene-1-sulfonamide

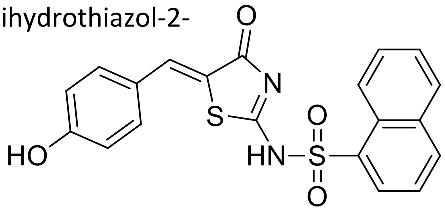

Chemical Formula: $C_{20}H_{14}N_2O_4S_2$
Exact Mass: 410.04
Molecular Weight: 410.47

Compound Code: 6 (KP5123)

Obtained Weight & Yield: 159 mg (77%)

Purity (by LCMS and ^{1}H NMR): > 98% by LCMS and NMR

Appearance: Bright yellow solid

Solubility: DMSO, slightly soluble in ethanol, acetone, methanol and ethyl acetate

Melting Point: > 255 °C (dec.)

TLC Rf (and conditions): Rf 0.15 (5% MeOH in DCM)

IR Analysis (including assignment): IR (neat): ν_{max} = 3140 (broad, OH), 3043, 2784 (aromatic C-H), 1697 (C=O), 1564 (N-H), 1511 (C-C aromatic), 1127 (C-N) cm^{-1}

^{1}H NMR Analysis: ^{1}H NMR (400 MHz, DMSO) δ 13.05 (s, 1H), 10.45 (s, 1H), 8.61 (d, *J* = 8.6 Hz, 1H), 8.31 (dd, *J* = 7.7, 3.8 Hz, 2H), 8.13 (d, *J* = 8.0 Hz, 1H), 7.77 (t, *J* = 7.2 Hz, 1H), 7.71 – 7.68 (m, 3H), 7.55 (d, *J* = 8.6 Hz, 2H), 6.99 (d, *J* = 8.6 Hz, 2H) ppm.
Methanol impurity at 3.17 ppm (1.56%)

^{13}C NMR Analysis: ^{13}C NMR (101 MHz, DMSO) δ 170.3 (br), 165.8 (br), 160.5 (C-OH), 135.4, 134.6, 134.4, 133.8, 132.9 (2C), 129.0, 128.3, 128.1, 127.6, 127.1, 124.9, 124.6, 123.7, 117.0 (br), 116.5 (2C) ppm.
2C peaks assigned by 2D NMR

MS Analysis (low res): LRMS (ESI-) *m/z*: 409 (M-H, $C_{20}H_{13}N_2O_4S_2$, 100%); (ESI+) *m/z*: 411 (M+H, $C_{20}H_{15}N_2O_4S_2$, 100%)

HPLC method details: Column: Zorbax SB-C18 Rapid Resolution HT 2.1x50mm 1.8-Micron; Method: LCMS ISOCRATIC 50% B.M_REDUCED FLOW.M filename: KP5123_N; Peak retention time: 1.18 mins; Area (%): 99.

Procedure: To a 10 mL microwave vial was added the *N*-(4-oxo-4,5-dihydrothiazol-2-yl)naphthalene-1-sulfonamide (KP5122, 155 mg, 0.51 mmol), 4-hydroxybenzaldehyde (66 mg, 0.54 mmol), ethanol (3 mL) and a catalytic amount of the benzoic acid/piperidine catalyst (approximately 10 drops, 1:1 solution 10% in ethanol). The suspension was heated using microwave irradiation (200 W, 120 °C) for 30 minutes then placed in the freezer overnight. No precipitate was observed and after failed column chromatography the reaction mixture was partitioned between ethyl acetate and water. The organic layer was dried with magnesium sulfate and filtered. Hexane was added until a precipitate formed and the solution was left in the freezer overnight. The yellow solid was collected by vacuum filtration and washed with cold hexane to give the desired product (159 mg, 77%).

PerkinElmer Spectrum Version 10.4.00
Friday, 8 March 2019 10:42 AM

Analyst	research
Date	Friday, 8 March 2019 10:42 AM

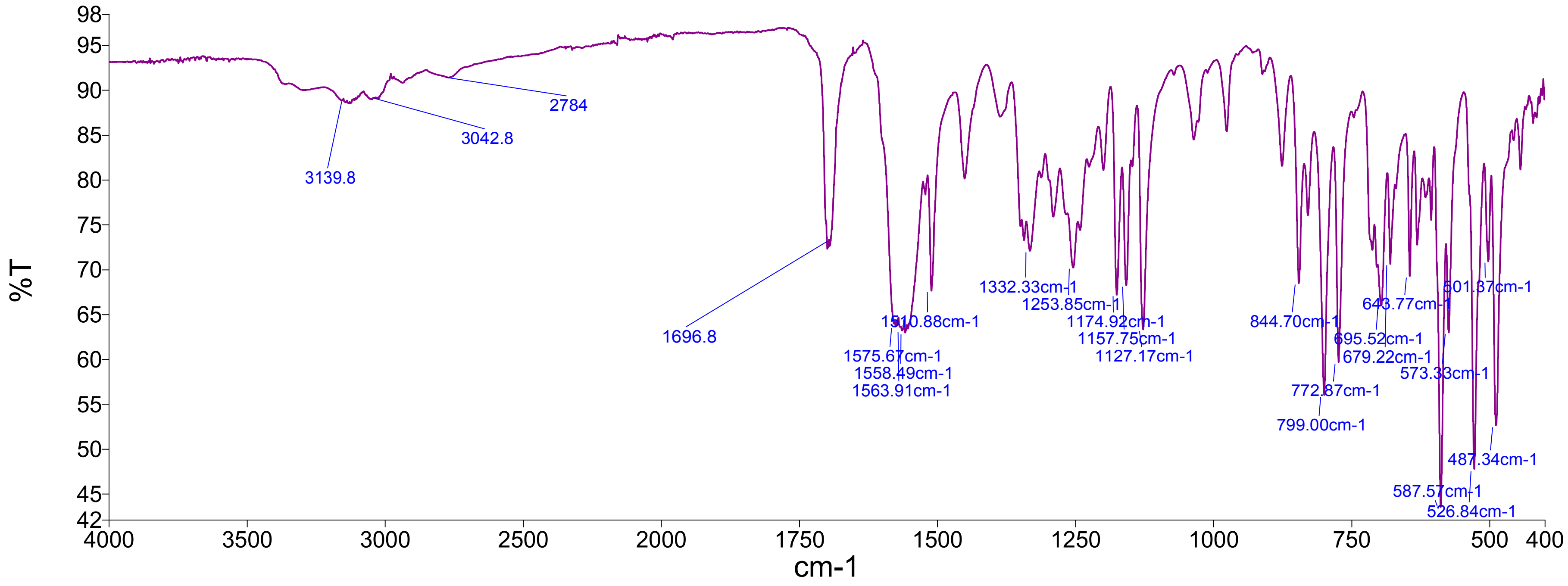

Sample Name	Description	Quality Checks
kp5123	Sample 175 By research Date Friday, March 08 2019	The Quality Checks do not report any warnings for the sample.

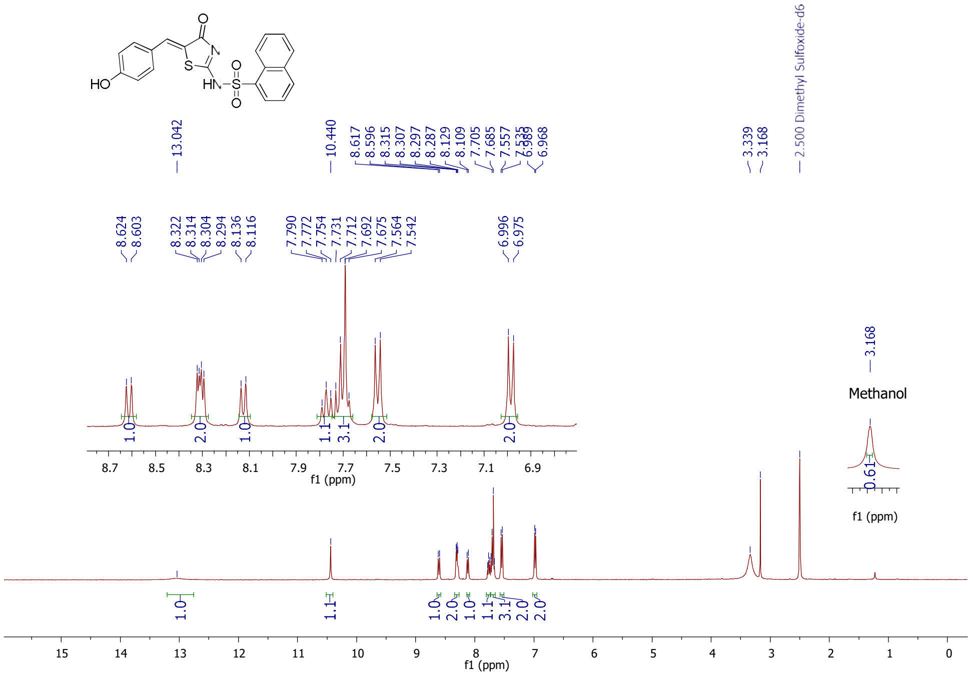
HO
S
N
O
HN
S
O
O
2.500 Dimethyl Sulfoxide-d6
13.042
10.440
3.339
3.168
Methanol
f1 (ppm)

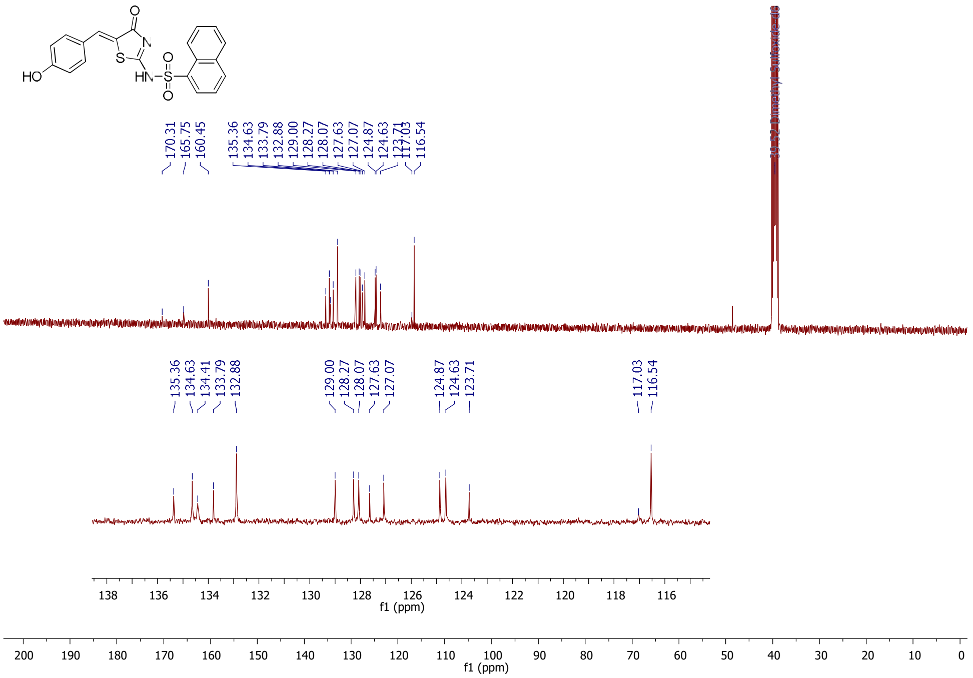
HO
S
N
O
HN
S
O
O
170.31
165.75
160.45
135.36
134.63
133.79
132.88
129.00
128.27
128.07
127.63
127.07
124.87
124.63
123.71
117.03
116.54
134.41
f1 (ppm)

LCMS Report

**Data file:**	D:\Chem32\1\Data\KP\PRE 11-6-19\KP_5123-6003 2019-05-09 13-17-58\002-71-KP5123_N.D		
**Sample name:**	KP5123_N		
**Description:**			
**Sample amount:**	0.000	**Sample type:**	Sample
**Instrument:**	LCMS	**Location:**	71
**Injection date:**	5/9/2019 1:26:01 PM	**Injection:**	1 of 1
**Acq. method:**	LCMS ISOCRATIC 50% B.M_REDUCED FLOW.M	**Injection volume:**	2.000
**Analysis method:**	LCMS ISOCRATIC 50% B.M_REDUCED FLOW.M	**Acq. operator:**	SYSTEM
**Last changed:**	10/21/2016 12:01:19 PM		

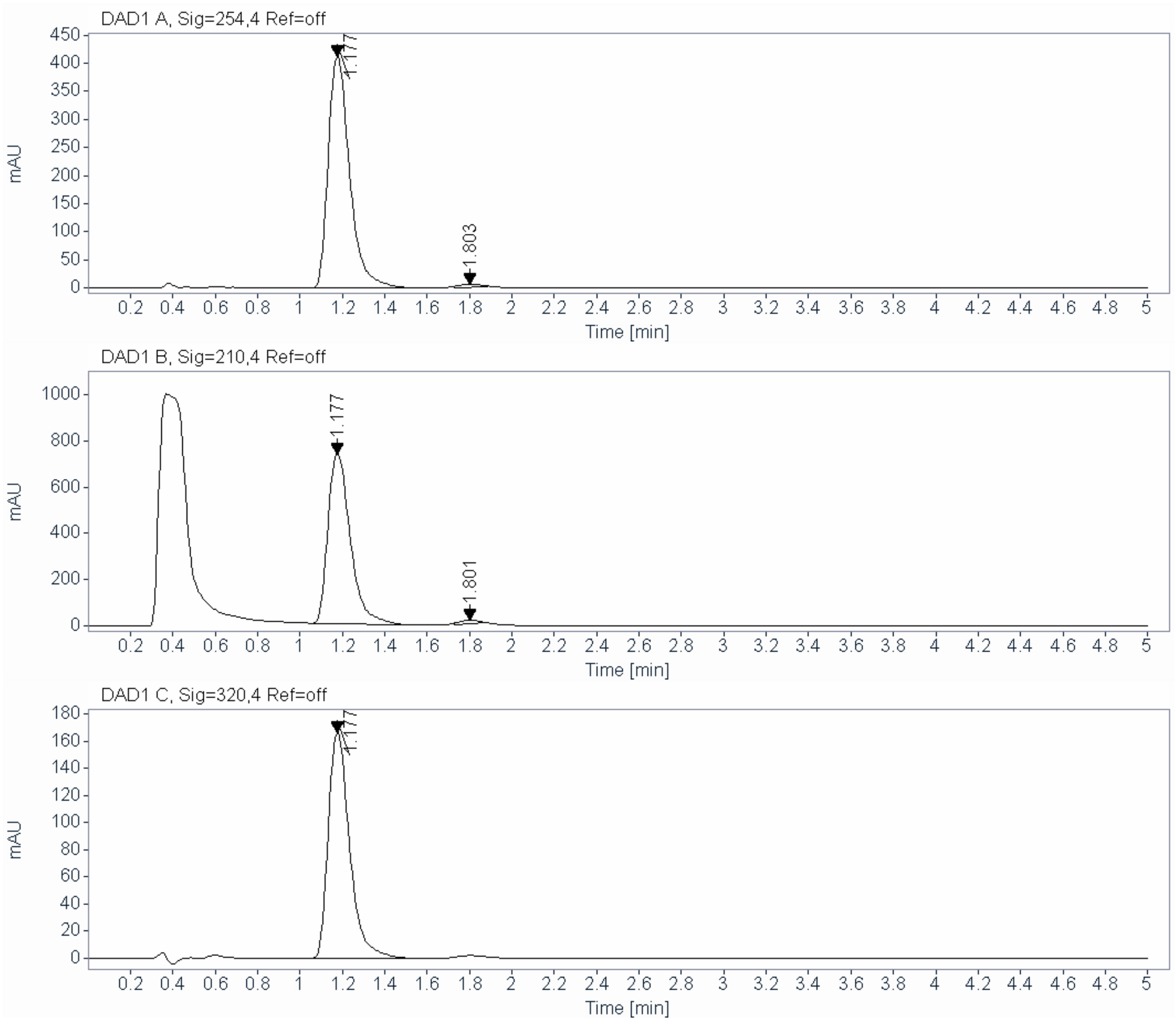

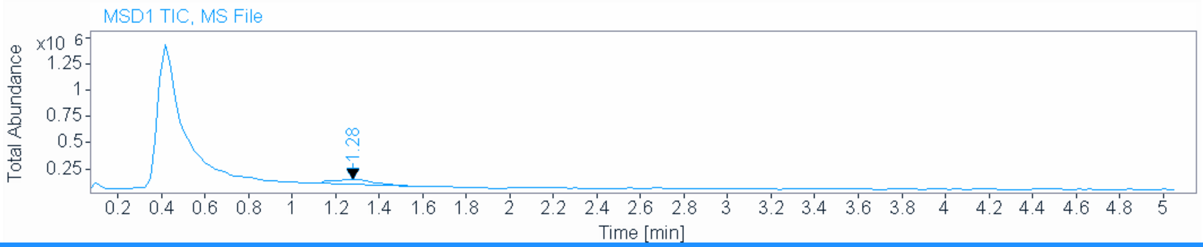

Agilent Technologies

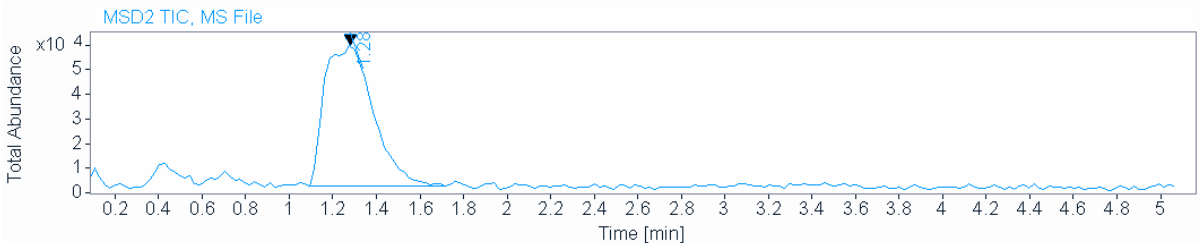

Signal: DAD1 A, Sig=254,4 Ref=off

RT [min]	Type	Width [min]	Area	Height	Area%	Name
1.177	BB	0.1075	2890.6182	414.0179	98.8091	
1.803	MM	0.1118	34.8405	5.1957	1.1909	
		Sum	2925.4587			

Signal: DAD1 B, Sig=210,4 Ref=off

RT [min]	Type	Width [min]	Area	Height	Area%	Name
1.177	BB	0.1134	5386.4839	736.0042	98.3968	
1.801	MM	0.1000	87.7646	14.6208	1.6032	
		Sum	5474.2485			

Signal: DAD1 C, Sig=320,4 Ref=off

RT [min]	Type	Width [min]	Area	Height	Area%	Name
1.177	BB	0.1075	1167.4493	167.1922	100.0000	
		Sum	1167.4493			

Signal: MSD1 TIC, MS File

RT [min]	Type	Width [min]	Area	Height	Area%	Name
1.280	BB	0.1887	675648.3125	48910.4219	100.0000	
		Sum	675648.3125			

Signal: MSD2 TIC, MS File

RT [min]	Type	Width [min]	Area	Height	Area%	Name
1.280	BB	0.2427	880386.3125	56822.4727	100.0000	
		Sum	880386.3125			

Agilent Technologies

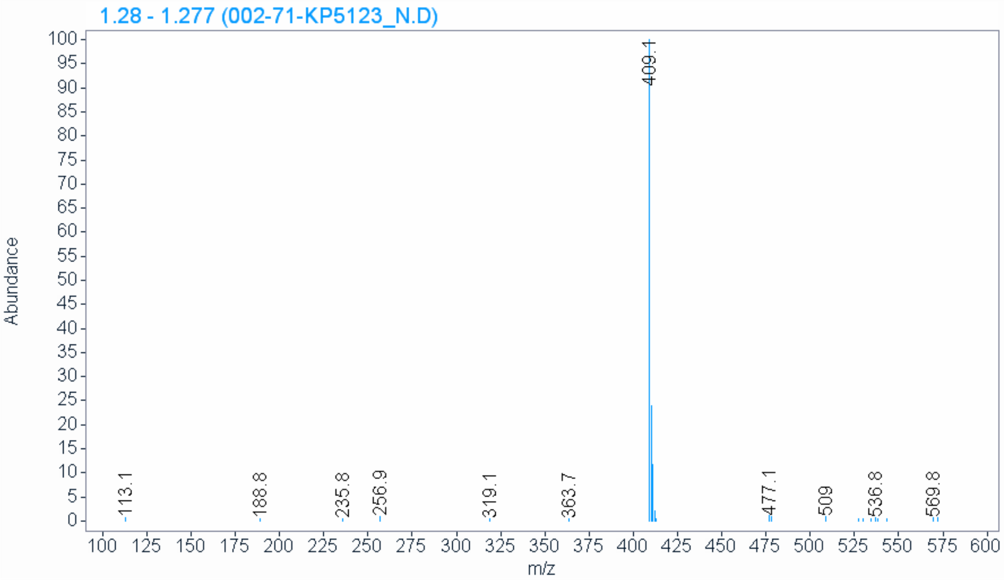

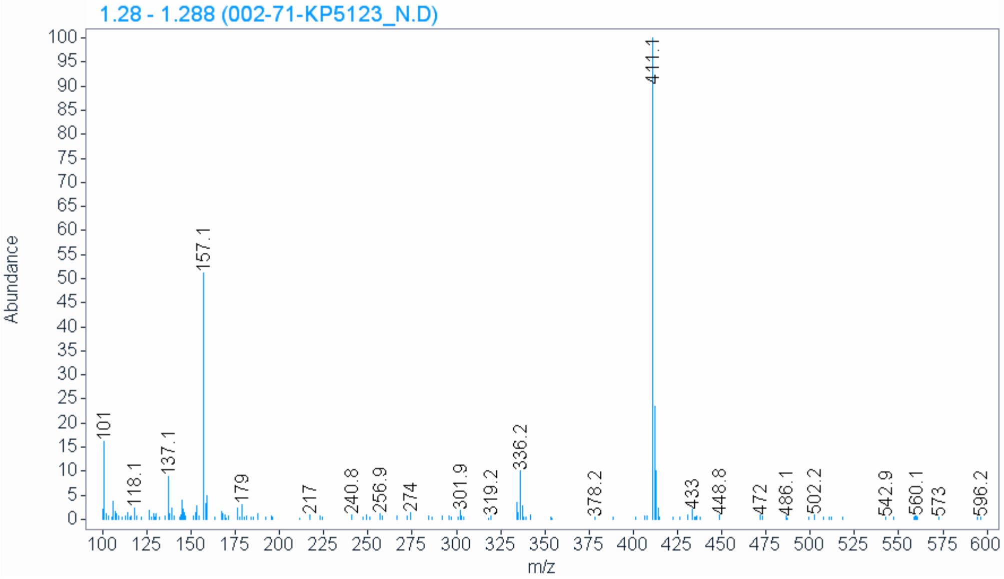

Compound Name: *N*-[(5*Z*)-5-(3-hydroxybenzylidene)-4-oxo-4,5-dihydro-1,3-thiazol-2-yl]naphthalene-1-sulfonamide

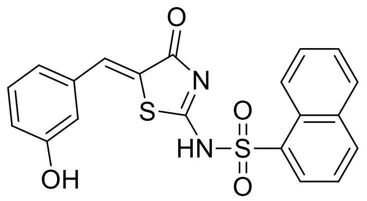

Chemical Formula: $C_{20}H_{14}N_2O_4S_2$
Exact Mass: 410.04
Molecular Weight: 410.47

Compound Code: 7 (KP5125)

Obtained Weight & Yield: 154 mg (77%)

Purity (by LCMS and ^{1}H NMR): 98% (LCMS), >99% (^{1}H NMR)

Appearance: Yellow solid

Solubility: DMSO, slightly soluble in acetone, ethyl acetate, methanol and ethanol

Melting Point: > 243 °C (dec.)

TLC Rf (and conditions): Rf 0.16 (5% MeOH in DCM)

IR Analysis (including assignment): IR (neat): ν_{max} = 3386 (O-H), 3062, 2939 (C-H aromatic), 1713 (C=O), 1595 (N-H), 1559, 1563 (C-C aromatic), 1124 (C-N) cm^{-1}

^{1}H NMR Analysis: ^{1}H NMR (400 MHz, DMSO) δ 13.22 (s, 1H, NH), 9.99 (s, 1H, H), 8.60 (d, *J* = 8.5 Hz, 1H), 8.30 (d, *J* = 7.7 Hz, 2H), 8.12 (d, *J* = 8.1 Hz, 1H), 7.77 (t, *J* = 7.3 Hz, 1H), 7.73 – 7.67 (m, 3H), 7.38 (t, *J* = 7.9 Hz, 1H), 7.11 (d, *J* = 7.8 Hz, 1H), 7.07 (s, 1H), 6.93 (d, *J* = 8.0 Hz, 1H) ppm.

^{13}C NMR Analysis: ^{13}C NMR (151 MHz, DMSO) δ 166.7 (br), 165.8, 158.0, 135.3, 134.8, 134.01, 133.95, 133.8, 130.6, 129.1, 128.4, 128.1, 127.7, 127.2, 124.9, 124.7, 121.9, 121.6 (br), 118.3, 115.9 ppm.

MS Analysis (low res): LRMS (ESI-) *m/z*: 409 (M-H, $C_{20}H_{13}N_2O_4S_2$, 100%); (ESI+) *m/z*: 411 (M+H, $C_{20}H_{15}N_2O_4S_2$, 100%)

HPLC method details: Column: Zorbax SB-C18 Rapid Resolution HT 2.1x50mm 1.8-Micron; Method LCMS ISOCRATIC 50% B.M_REDUCED FLOW.M filename: KP5125A; Peak retention time: 1.47 mins; Area (%): 100

Procedure: To a 10mL microwave reaction vessel were added *N*-(4-oxo-4,5-dihydrothiazol-2-yl)naphthalene-1-sulfonamide (KP5122, 150 mg, 0.49 mmol, 1 eq), 3-hydroxybenzaldehyde (68 mg, 0.56 mmol, 1.1 eq), benzoic acid/piperidine mix (1:1 mixture 10% in ethanol, catalytic amount 10 drops) and ethanol (3 mL). The resulting suspension was heated using microwave irradiation (200 W, 120 °C) for 30 minutes. After cooling overnight no precipitate observed. After failed column chromatography the crude product was dissolved in ethyl acetate and hexane was added dropwise until a precipitate was observed. After cooling a yellow solid was collected by vacuum filtration (154 mg, 77%).

Other analyses, reference papers, previously obtained data, comments, etc:

PerkinElmer Spectrum Version 10.4.00
Friday, 8 March 2019 10:36 AM

Analyst	research
Date	Friday, 8 March 2019 10:36 AM

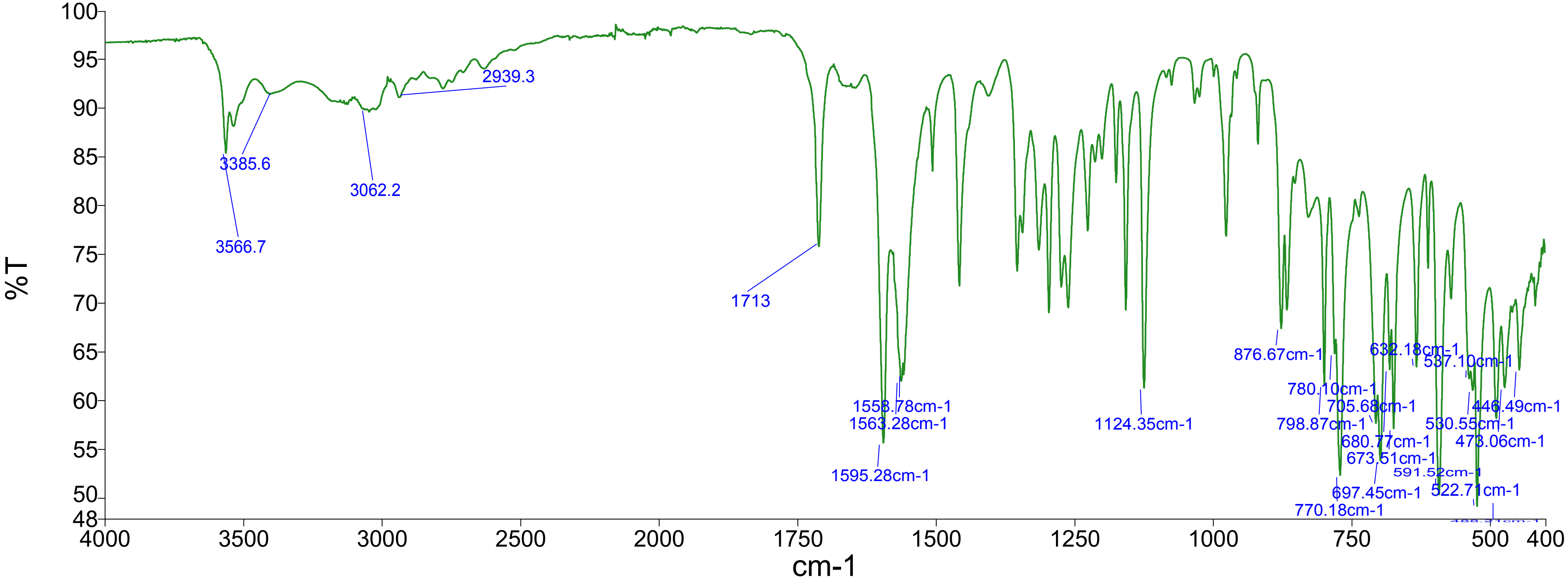

Sample Name	Description	Quality Checks
kp5125	Sample 173 By research Date Friday, March 08 2019	The Quality Checks do not report any warnings for the sample.

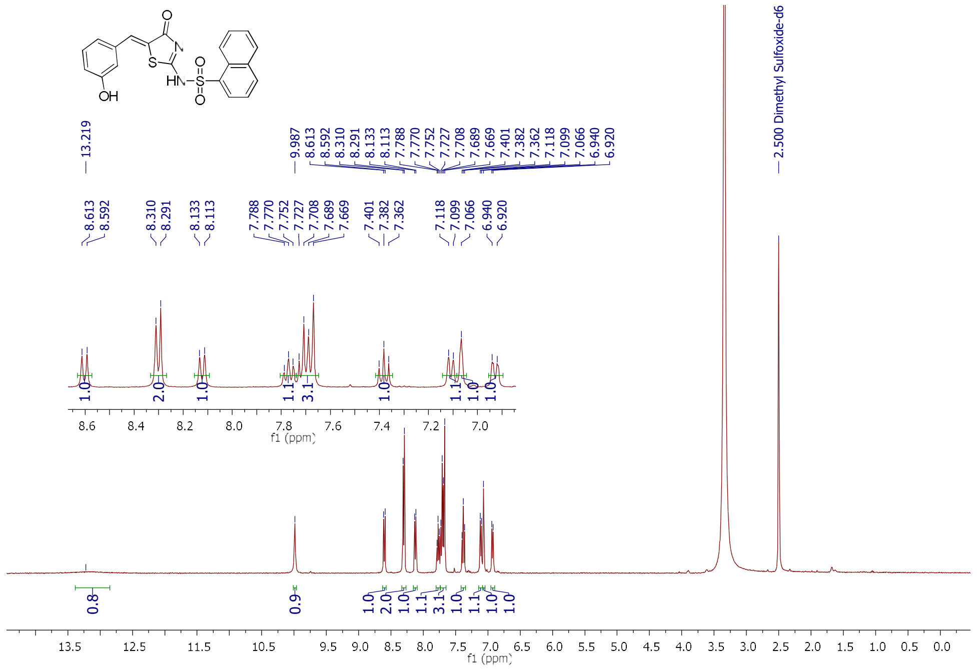
13.219
9.987
8.613
8.592
8.310
8.291
8.133
8.113
7.788
7.770
7.752
7.727
7.708
7.689
7.669
7.401
7.382
7.362
7.118
7.099
7.066
6.940
6.920
2.500 Dimethyl Sulfoxide-d6
0.8
0.9
1.0
2.0
1.0
1.1
3.1
1.0
1.1
1.0
1.0
f1 (ppm)

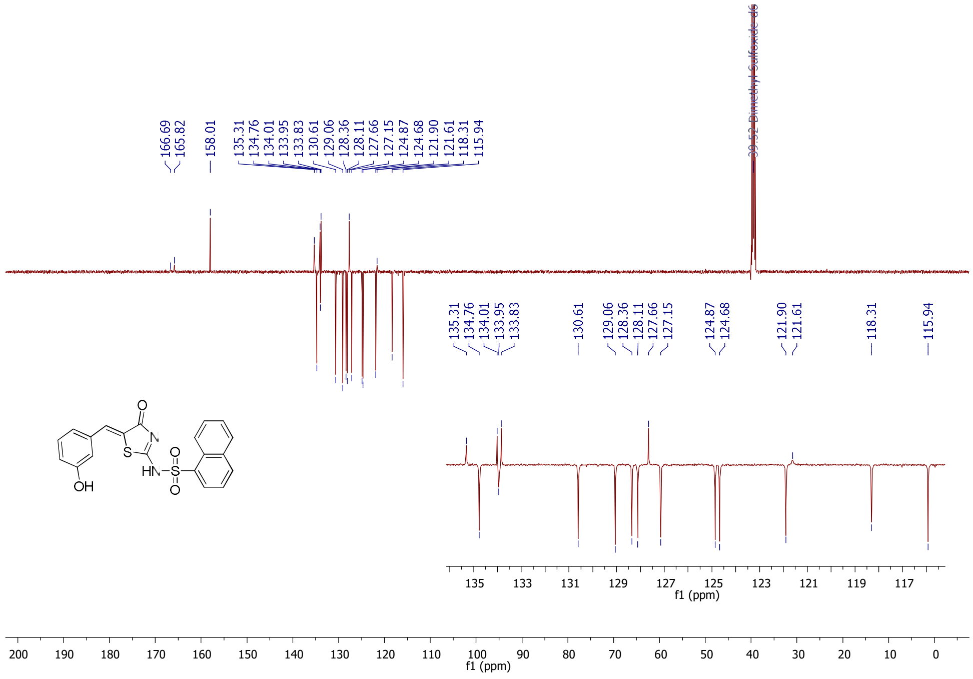
166.69
165.82
158.01
135.31
134.76
134.01
133.95
133.83
130.61
129.06
128.36
128.11
127.66
127.15
124.87
124.68
121.90
121.61
118.31
115.94
f1 (ppm)

LCMS Report

**Data file:**	D:\Chem32\1\Data\KP\PRE 11-6-19\KP5125-5133 2019-02-28 11-01-07\003-42-KP5125A.D		
**Sample name:**	KP5125A		
**Description:**			
**Sample amount:**	0.000	**Sample type:**	Sample
**Instrument:**	LCMS	**Location:**	42
**Injection date:**	2/28/2019 11:15:49 AM	**Injection:**	1 of 1
**Acq. method:**	LCMS ISOCRATIC 50% B.M_REDUCED FLOW.M	**Injection volume:**	2.000
**Analysis method:**	LCMS ISOCRATIC 50% B.M_REDUCED FLOW.M	**Acq. operator:**	SYSTEM
**Last changed:**	10/21/2016 12:01:19 PM		

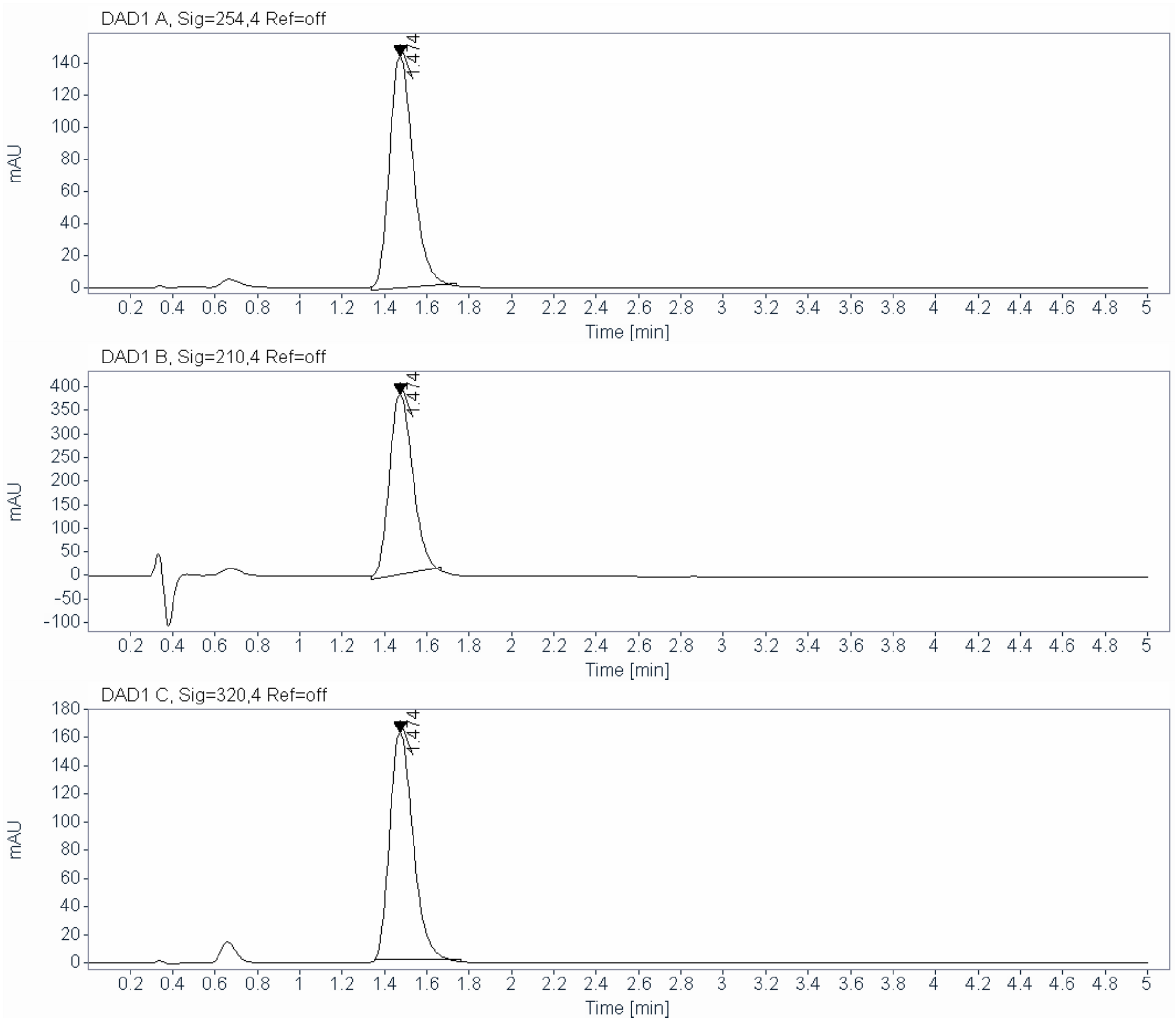

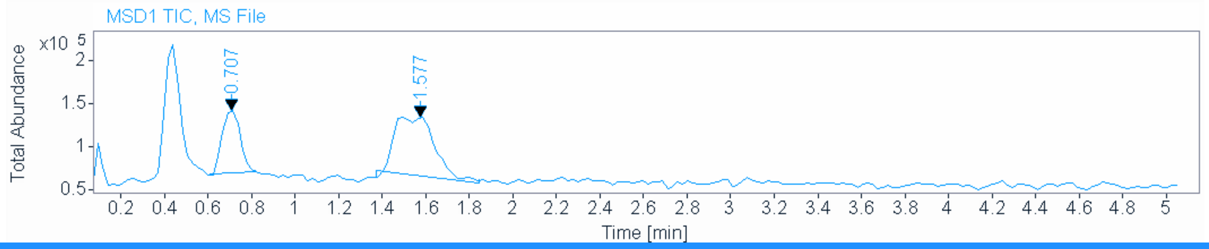

Agilent Technologies

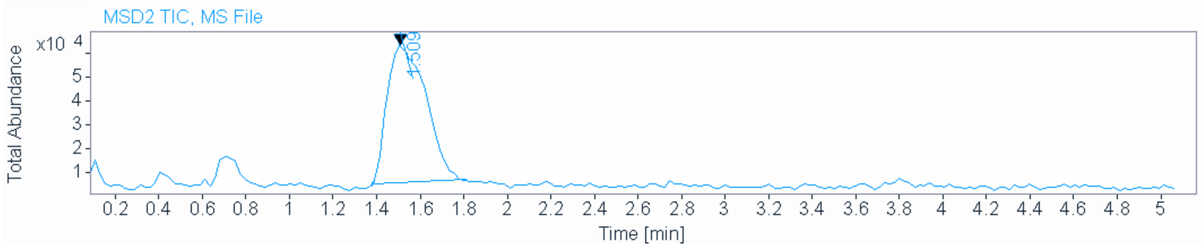

Signal: DAD1 A, Sig=254,4 Ref=off

RT [min]	Type	Width [min]	Area	Height	Area%	Name
1.474	MM	0.1295	1128.5656	145.2694	100.0000	
		Sum	1128.5656			

Signal: DAD1 B, Sig=210,4 Ref=off

RT [min]	Type	Width [min]	Area	Height	Area%	Name
1.474	MM	0.1271	2916.8247	382.5350	100.0000	
		Sum	2916.8247			

Signal: DAD1 C, Sig=320,4 Ref=off

RT [min]	Type	Width [min]	Area	Height	Area%	Name
1.474	MM	0.1273	1233.6141	161.5628	100.0000	
		Sum	1233.6141			

Signal: MSD1 TIC, MS File

RT [min]	Type	Width [min]	Area	Height	Area%	Name
0.707	MM	0.0933	419244.8125	74888.5234	32.5390	
1.577	MM	0.2089	869193.7500	69356.0391	67.4610	
		Sum	1288438.562			

Signal: MSD2 TIC, MS File

RT [min]	Type	Width [min]	Area	Height	Area%	Name
1.509	MM	0.2051	715930.2500	58170.2148	100.0000	
		Sum	715930.2500			

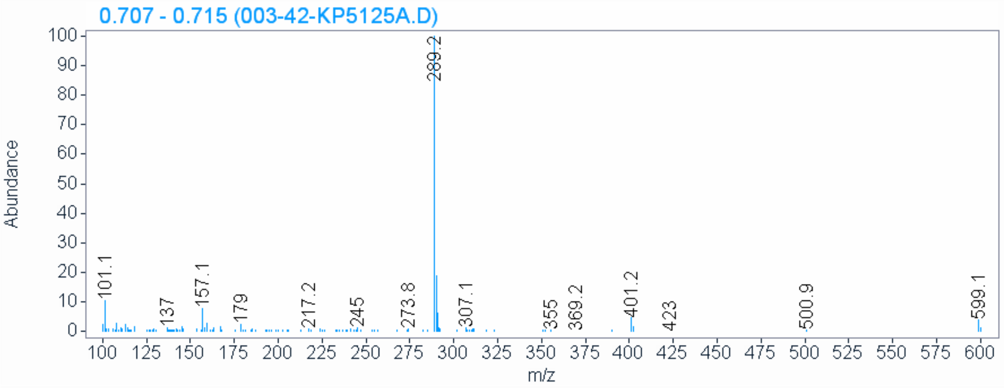
0.707 - 0.715 (003-42-KP5125A.D)
Abundance
m/z
289.2
101.1
137
157.1
179
217.2
245
273.8
307.1
355
369.2
401.2
423
500.9
599.1

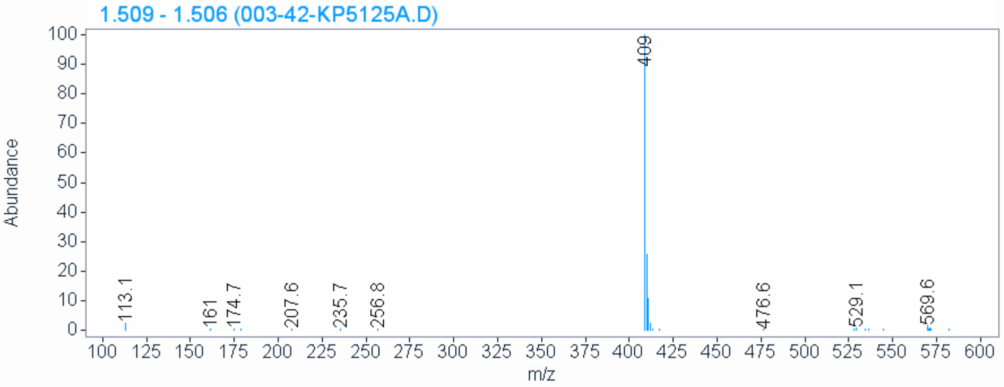
1.509 - 1.506 (003-42-KP5125A.D)
Abundance
m/z
409
113.1
161
174.7
207.6
235.7
256.8
476.6
529.1
569.6

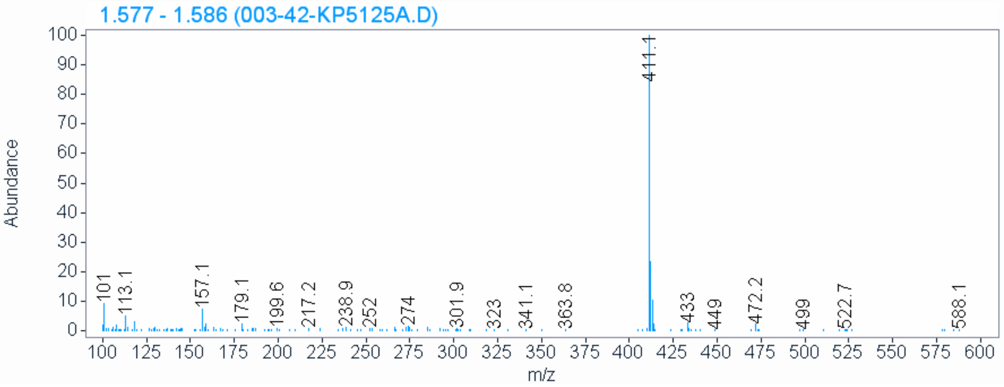
1.577 - 1.586 (003-42-KP5125A.D)
Abundance
m/z
411.1
101
113.1
157.1
179.1
199.6
217.2
238.9
252
274
301.9
323
341.1
363.8
433
449
472.2
499
522.7
588.1

Compound Name: (*Z*)-*N*-(5-(2-hydroxybenzylidene)-4-oxo-4,5-dihydrothiazol-2-yl)naphthalene-1-sulfonamide

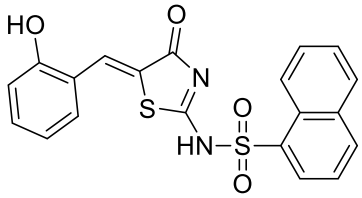

Chemical Formula: $C_{20}H_{14}N_2O_4S_2$
Exact Mass: 410.04
Molecular Weight: 410.47

Compound Code: 8 (KP7196)

Obtained Weight & Yield: 69 mg (35%)

Purity (by LCMS and ^{1}H NMR): >99% ^{1}H-NMR, >99% LCMS

Appearance: yellow solid

Solubility: DMSO, slightly soluble in MeOH and EtOH.

Melting Point: > 218 °C (dec.)

TLC Rf (and conditions): N/A

IR Analysis (including assignment): IR (neat): ν_{max} = 3373 (O-H), 3210 (N-H), 2961 (C-H aromatic), 1708 (C=O), 1555 (aromatic C-C), 1355 (sulfonamide), 1126 (C-N) cm^{-1}

^{1}H NMR Analysis: ^{1}H NMR (400 MHz, DMSO) δ 13.10 (br, s, 1H), 10.69 (s, 1H, OH), 8.60 (d, *J* = 8.4 Hz, 1H), 8.31 – 8.23 (m, 2H), 8.12 (d, *J* = 8.0 Hz, 1H), 7.95 (s, 1H), 7.77 (t, *J* = 7.1 Hz, 1H), 7.72 – 7.67 (m, 2H), 7.44 (d, *J* = 7.8 Hz, 1H), 7.39 – 7.34 (m, 1H), 7.04 – 6.98 (m, 2H) ppm.
Ethanol at 1.06 ppm (0.71%)

^{13}C NMR Analysis: ^{13}C DEPTQ NMR (151 MHz, DMSO) δ 166.7, 166.0, 157.4, 135.4, 134.7, 133.8, 133.0, 129.5, 129.3, 129.0, 128.3, 128.1, 127.7, 127.1, 124.9, 124.7, 120.1, 119.9, 119.7, 116.3 ppm.

MS Analysis (low res): LRMS (ESI-) *m/z* (%): 409 (*M*-H, $C_{20}H_{13}N_2O_4S_2$, 100%);

MS Analysis (high res): Exact mass calculated for $C_{20}H_{13}N_2O_4S_2$ [*M*-H]$^-$, 409.0300. Found 409.0321.

HPLC method details: Column: Zorbax SB-C18 Rapid Resolution HT 2.1x50mm 1.8-Micron; Method: LCMS ISOCRATIC 60%B_3MINS.M filename: KP7196 Peak retention time: 0.821 mins; Area (%): 100.

Procedure: To a 10 mL microwave vial was added the *N*-(4-oxo-4,5-dihydrothiazol-2-yl)naphthalene-1-sulfonamide (148 mg, 0.49 mmol), 2-hydroxy benzaldehyde (0.08 mL, 0.54 mmol, 1.1 eq), ethanol (3 mL) and a catalytic amount of the benzoic acid/piperidine catalyst (approximately 5 drops). The suspension was heated using microwave irradiation (200 W, 120 °C) for 1 h. A precipitate formed on the addition of H_2O and the mixture was allowed to cool overnight. The resulting precipitate was collected by vacuum filtration and washed with cold ethanol and cold ether to give the desired product (69 mg, 35%)

Other analyses, reference papers, previously obtained data, comments, etc:

PerkinElmer Spectrum Version 10.4.00
Thursday, 25 June 2020 12:29 PM

Analyst undergrad
Date Thursday, 25 June 2020 12:29 PM

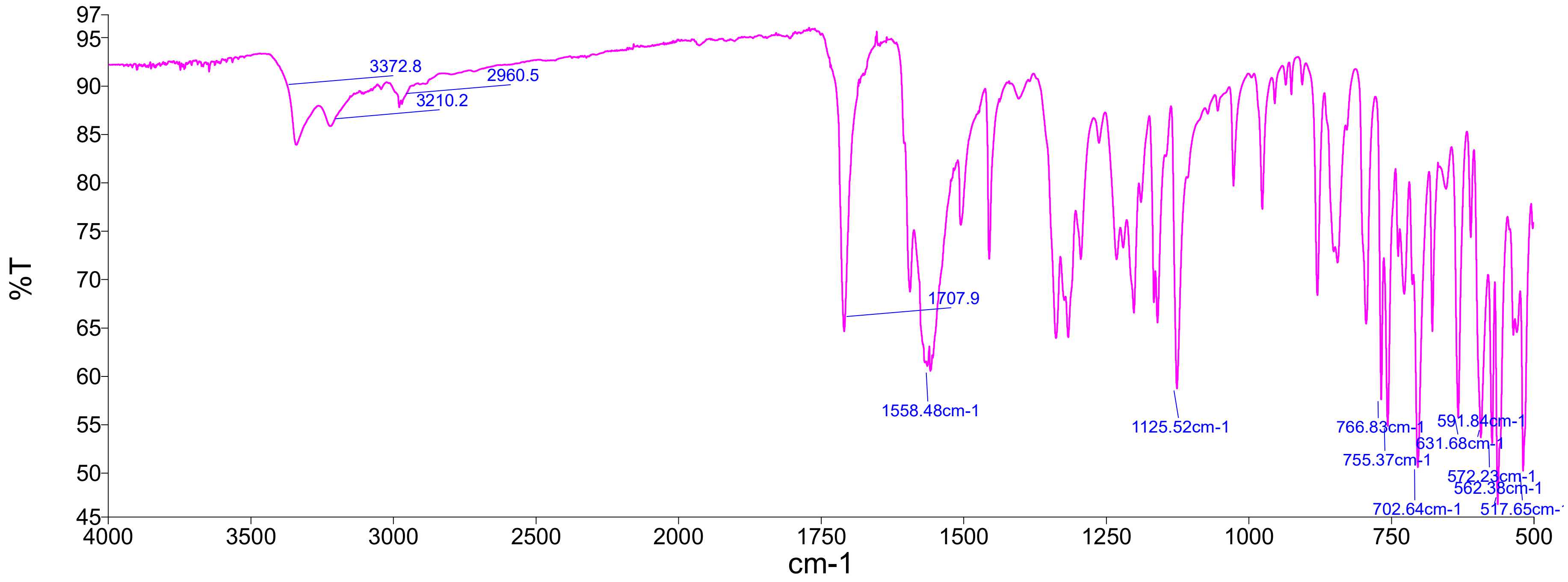

Sample Name	Description	Quality Checks
kp7196	Sample 060 By class Date Thursday, June 25 2020	The Quality Checks do not report any warnings for the sample.

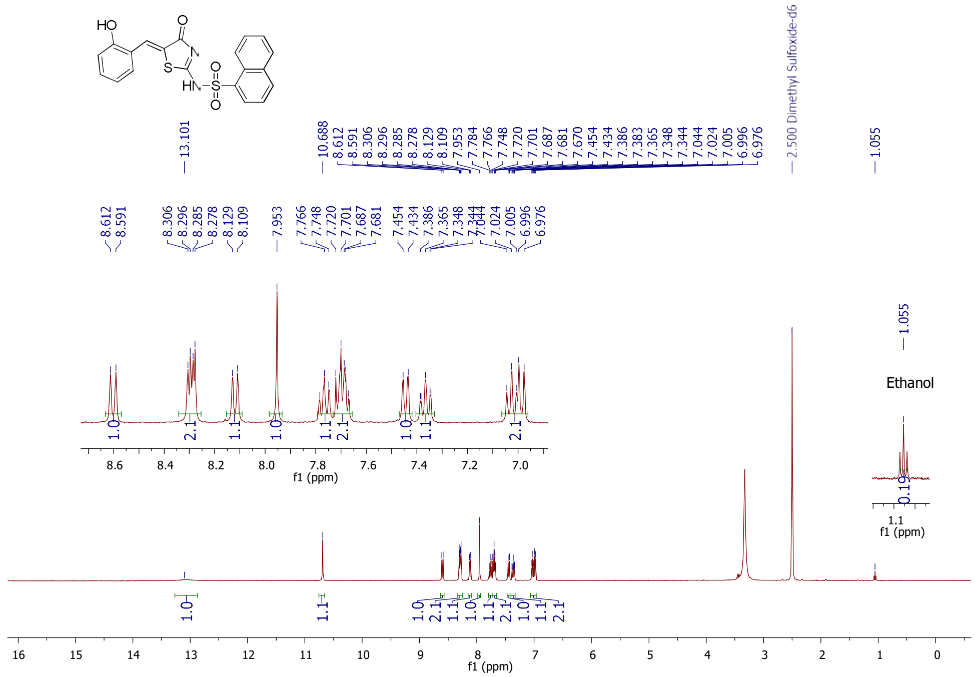
HO
O
S
N
HN
S
O
O
13.101
10.688
8.612
8.591
8.306
8.296
8.285
8.278
8.129
8.109
7.953
7.784
7.766
7.748
7.720
7.701
7.687
7.681
7.670
7.454
7.434
7.386
7.383
7.365
7.348
7.344
7.044
7.024
7.005
6.996
6.976
2.500 Dimethyl Sulfoxide-d6
1.055
Ethanol
f1 (ppm)

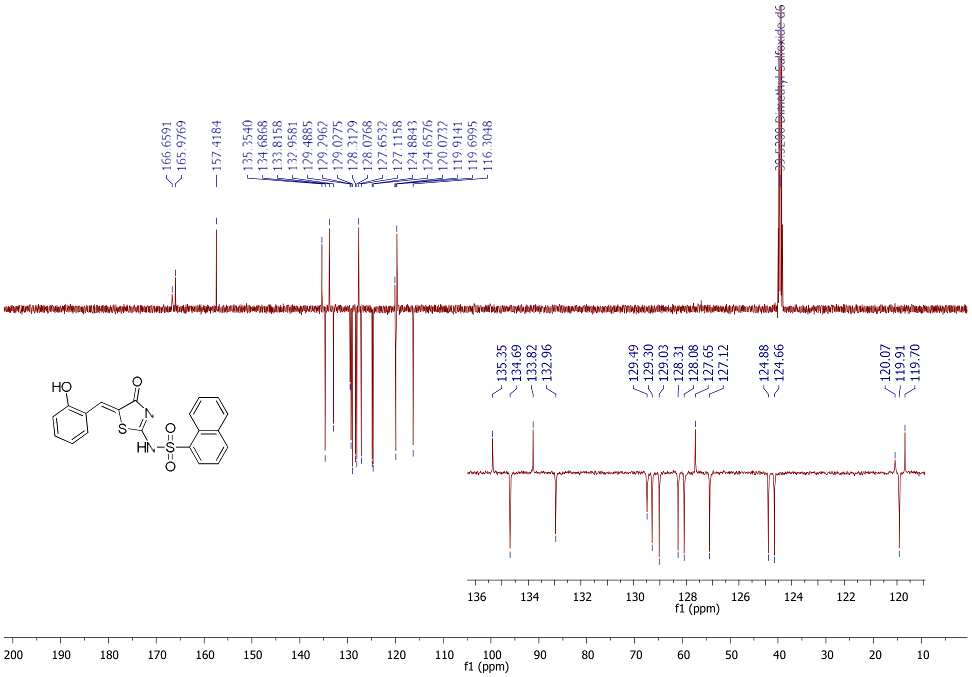
166.6591
165.9769
157.4184
135.3540
134.6868
133.8158
132.9581
129.4885
129.2962
129.0275
128.3129
128.0768
127.6532
127.1158
124.8843
124.6576
120.0732
119.9141
119.6995
116.3048
Dimethyl Sulfoxide-d6
135.35
134.69
133.82
132.96
129.49
129.30
129.03
128.31
128.08
127.65
127.12
124.88
124.66
120.07
119.91
119.70
HO
O
S
N
HN
S
O
O
f1 (ppm)

LCMS Report

Agilent Technologies

**Data file:**	D:\Chem32\1\Data\KP\KP_DS3_19MAR20 2020-03-19 11-59-31\003-29-KP7196.D		
**Sample name:**	KP7196		
**Description:**			
**Sample amount:**	0.000	**Sample type:**	Sample
**Instrument:**	LCMS	**Location:**	29
**Injection date:**	3/19/2020 12:10:21 PM	**Injection:**	1 of 1
**Acq. method:**	LCMS ISOCRATIC 60% B_3MINS.M	**Injection volume:**	2.000
**Analysis method:**	LCMS ISOCRATIC 60%B_3MINS.M	**Acq. operator:**	SYSTEM
**Last changed:**	5/19/2016 3:52:53 PM		

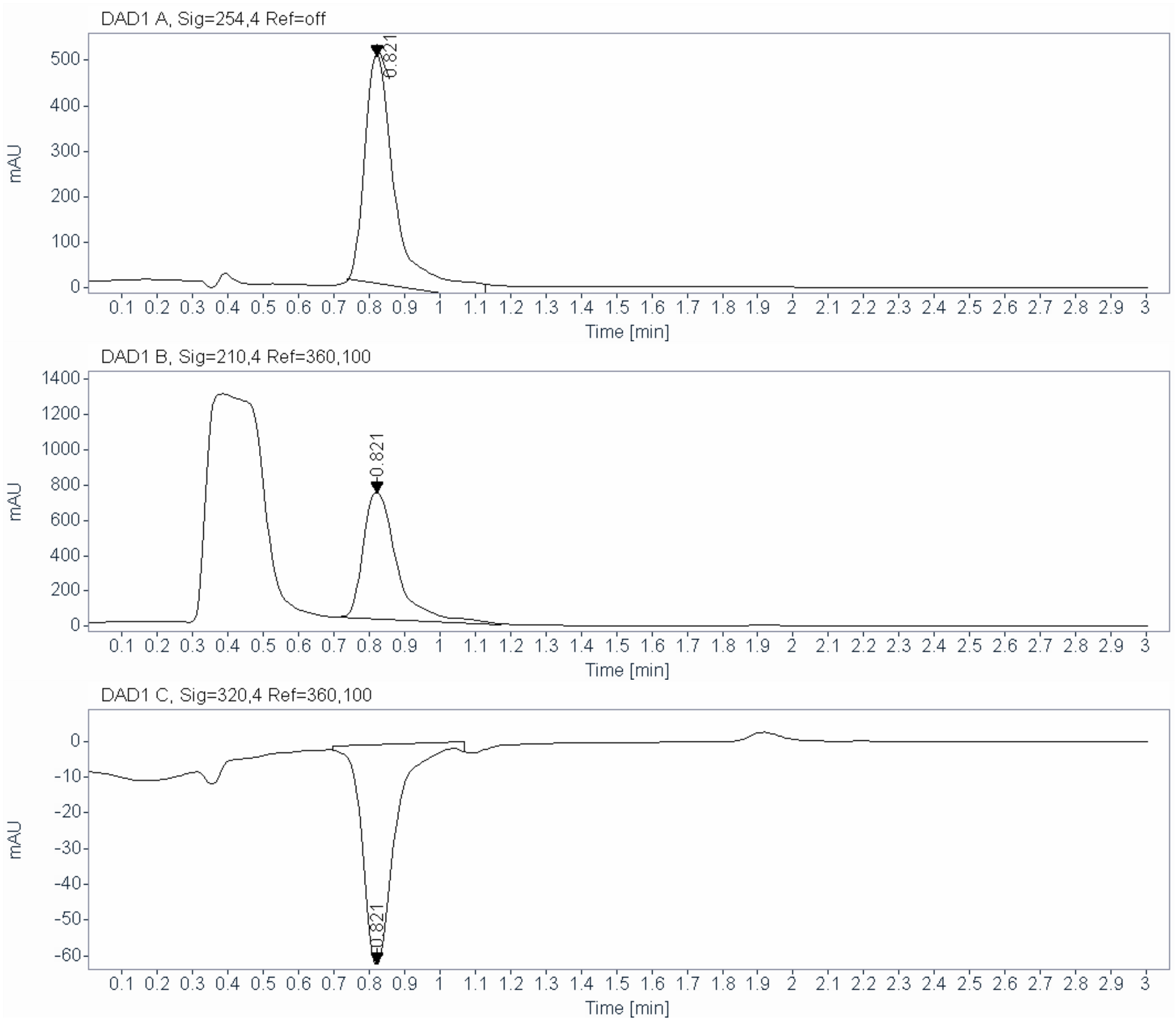

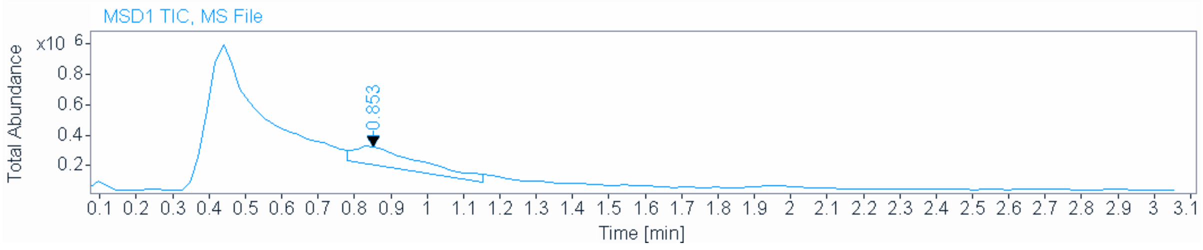

LCMS Report

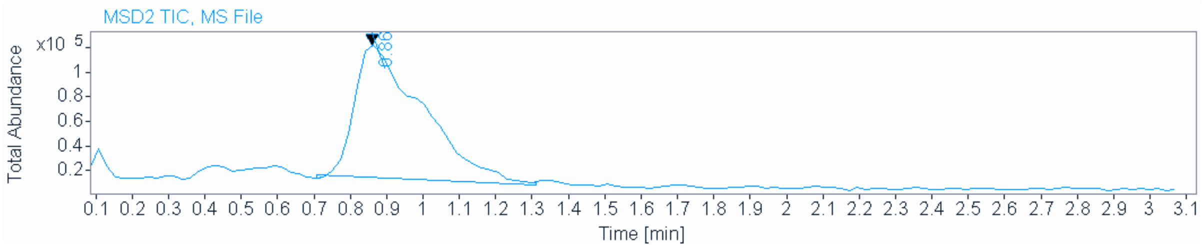

Signal: DAD1 A, Sig=254,4 Ref=off

RT [min]	Type	Width [min]	Area	Height	Area%	Name
0.821	MM	0.1028	3100.3018	502.4877	100.0000	
		Sum	3100.3018			

Signal: DAD1 B, Sig=210,4 Ref=360,100

RT [min]	Type	Width [min]	Area	Height	Area%	Name
0.821	MM	0.1122	4812.5488	715.1274	100.0000	
		Sum	4812.5488			

Signal: DAD1 C, Sig=320,4 Ref=360,100

RT [min]	Type	Width [min]	Area	Height	Area%	Name
0.821	MM N	0.1012	375.6044	61.8472	100.0000	
		Sum	375.6044			

Signal: MSD1 TIC, MS File

RT [min]	Type	Width [min]	Area	Height	Area%	Name
0.853	MM	0.2258	1597317.875<br>0	117904.7344	100.0000	
		Sum	1597317.875			

Signal: MSD2 TIC, MS File

RT [min]	Type	Width [min]	Area	Height	Area%	Name
0.860	MM	0.2161	1398340.375<br>0	107848.7813	100.0000	
		Sum	1398340.375			

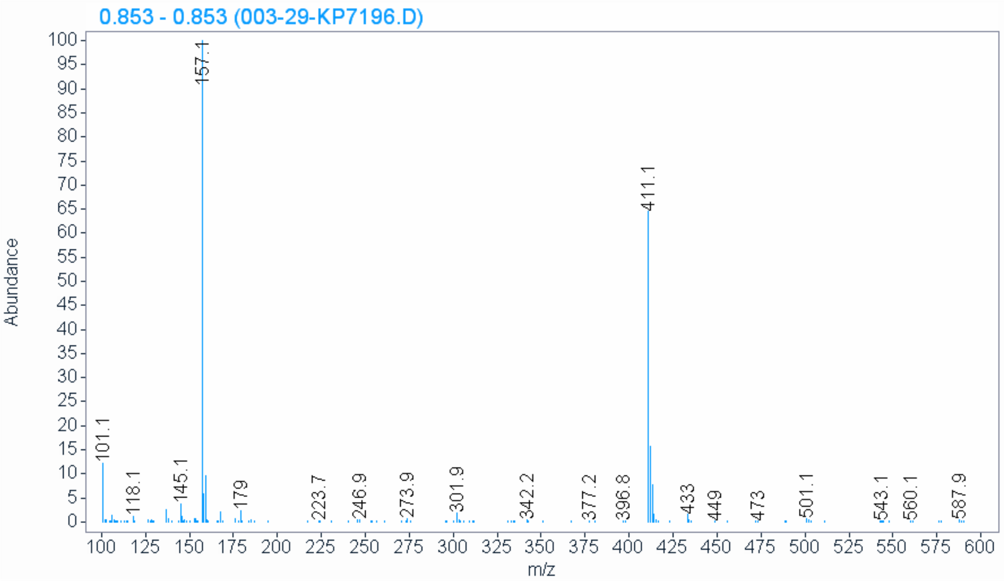

0.853 - 0.853 (003-29-KP7196.D)
Abundance
m/z
157.1
411.1
101.1
118.1
145.1
179
223.7
246.9
273.9
301.9
342.2
377.2
396.8
433
449
473
501.1
543.1
560.1
587.9

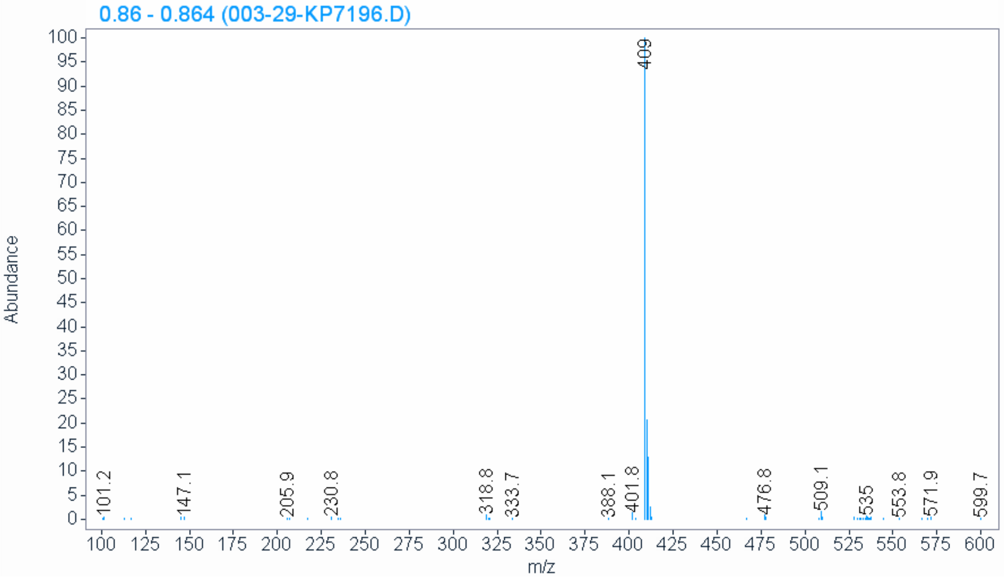

0.86 - 0.864 (003-29-KP7196.D)
Abundance
m/z
409
101.2
147.1
205.9
230.8
318.8
333.7
388.1
401.8
476.8
509.1
535
553.8
571.9
599.7

Compound Name: 4-[(*Z*)-{2-[(naphthalen-1-ylsulfonyl)amino]-4-oxo-1,3-thiazol-5(4*H*)-ylidene}methyl]benzoic acid

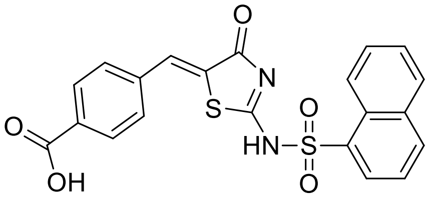

Chemical Formula: $C_{21}H_{14}N_2O_5S_2$
Exact Mass: 438.03
Molecular Weight: 438.48

Compound Code: 9 (KP5139)

Obtained Weight & Yield: 119 mg (54%)

Purity (by LCMS and ^{1}H NMR): > 99 % by ^{1}H-NMR

Appearance: Off white solid

Solubility: DMSO, slightly soluble in acetone, ethyl acetate, ethanol and methanol

Melting Point: > 300 °C (dec.)

TLC Rf (and conditions): Rf 0.03 (10% MeOH in DCM)

IR Analysis (including assignment): IR (neat): ν_{max} = 3289 (O-H), 3023 (C-H aromatic), 2519 (O-H carboxylic acid), 1726 (C=O), 1561 (C-C aromatic), 1309, 1280 (C-O carboxylic acid), 1116 (C-N) cm^{-1}

^{1}H NMR Analysis: ^{1}H NMR (400 MHz, DMSO) δ 13.26 (br, s, 1H, NH), 8.61 (d, *J* = 8.5 Hz, 1H), 8.31 (d, *J* = 7.2 Hz, 2H), 8.13 – 8.10 (m, 3H), 7.81 – 7.75 (m, 4H), 7.73 – 7.67 (m, 2H) ppm.
OH not observed

^{13}C NMR Analysis: ^{13}C NMR (151 MHz, DMSO) δ 166.6, 166.4, 165.4, 136.8, 135.2, 134.8, 133.8, 132.2, 132.1, 130.3 (2C), 130.2 (2C), 129.0, 128.4, 128.2, 127.6, 127.1, 124.8, 124.7, 124.2 ppm.
2C assigned by 2D NMR

MS Analysis (low res): LRMS (ESI-) *m/z*: 437.0 (M-H, $C_{21}H_{14}N_2O_5S_2$, 100%)

HPLC method details: Column: Zorbax SB-C18 Rapid Resolution HT 2.1x50mm 1.8-Micron; Method LCMS ISOCRATIC 50% B.M_REDUCED FLOW.M filename: KP5139; Peak retention time: 0.577 mins; Area (%): 99.

Procedure: To a 10 mL microwave reaction vessel were added *N*-(4-oxo-4,5-dihydrothiazol-2-yl)naphthalene-1-sulfonamide (KP5122, 150 mg, 0.49 mmol, 1 eq), 4-carboxybenzaldehyde (82 mg, 0.55 mmol, 1.1 eq), benzoic acid/piperidine mix (1:1 mixture 10% in ethanol, catalytic amount 10 drops) and ethanol (3 mL). The resulting suspension was heated using microwave irradiation (200 W, 120 °C) for 1 hr. After cooling overnight in freezer the precipitate was collected by vacuum filtration and washed with cold ethanol and cold diethyl ether to give the desired product as an off white solid (119 mg, 54%).

Other analyses, reference papers, previously obtained data, comments, etc:

PerkinElmer Spectrum Version 10.4.00
Friday, 8 March 2019 10:47 AM

Analyst	research
Date	Friday, 8 March 2019 10:47 AM

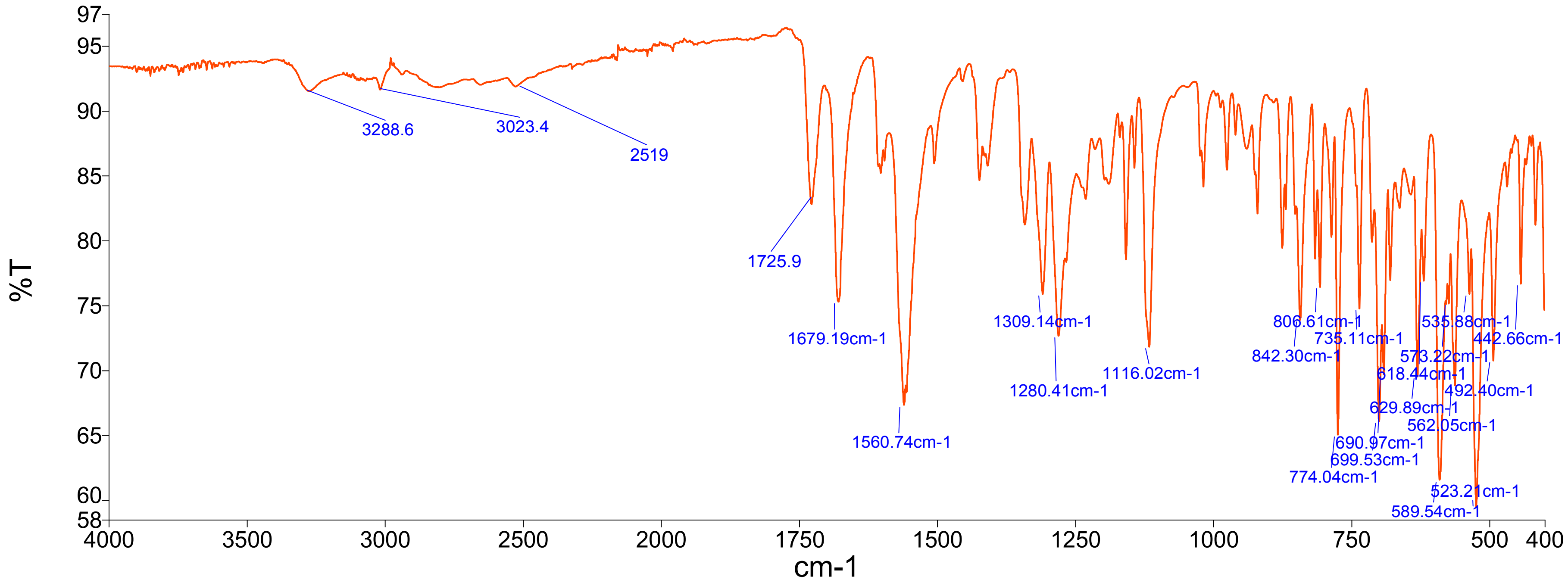

Sample Name	Description	Quality Checks
kp5139	Sample 177 By research Date Friday, March 08 2019	The Quality Checks give rise to a Weak Bands warning for the sample.

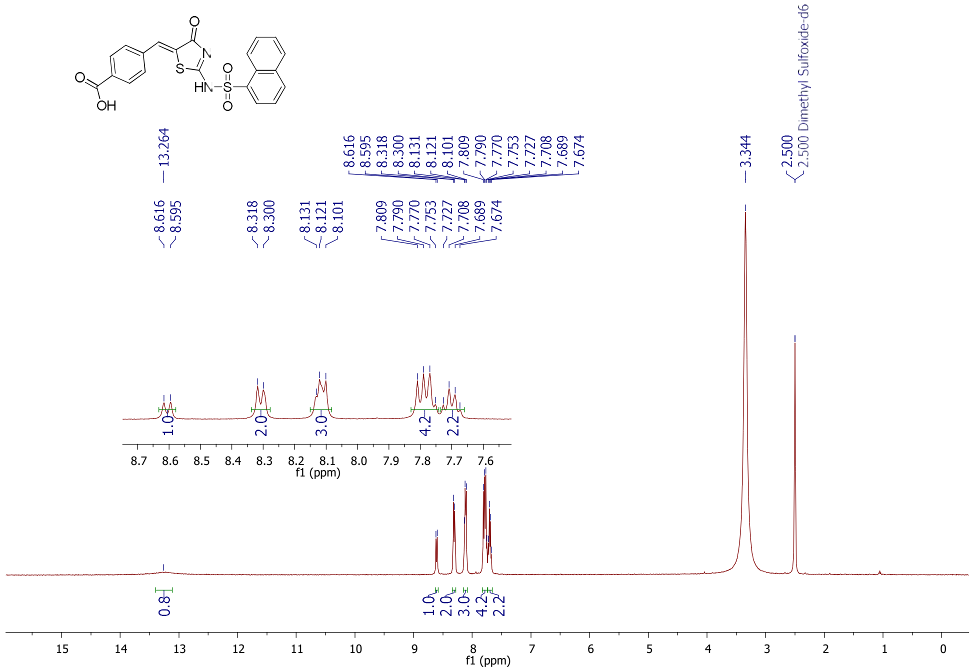
13.264
8.616
8.595
8.318
8.300
8.131
8.121
8.101
7.809
7.790
7.770
7.753
7.727
7.708
7.689
7.674
3.344
2.500
2.500 Dimethyl Sulfoxide-d6
1.0
2.0
3.0
4.2
2.2
0.8
f1 (ppm)
HN
S
O
OH

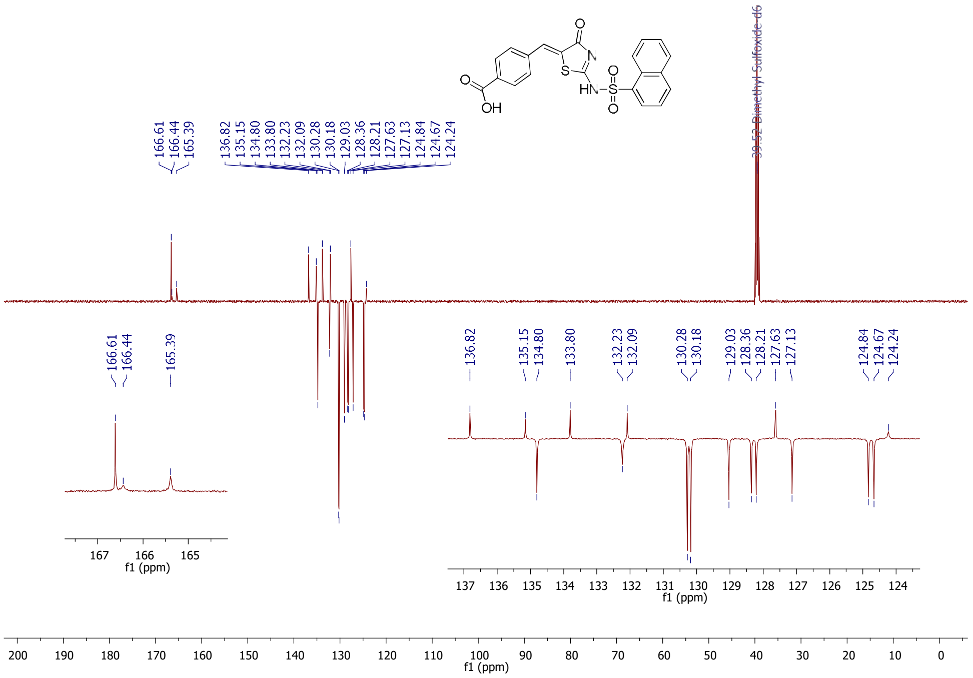
166.61
166.44
165.39
136.82
135.15
134.80
133.80
132.23
132.09
130.28
130.18
129.03
128.36
128.21
127.63
127.13
124.84
124.67
124.24
Dimethyl Sulfoxide-d6
f1 (ppm)
HN
S
O
OH

LCMS Report

Agilent Technologies

**Data file:**	D:\Chem32\1\Data\KP\KP5139 2019-03-01 10-21-36\002-45-KP5139.D		
**Sample name:**	KP5139		
**Description:**			
**Sample amount:**	0.000	**Sample type:**	Sample
**Instrument:**	LCMS	**Location:**	45
**Injection date:**	3/1/2019 10:29:56 AM	**Injection:**	1 of 1
**Acq. method:**	LCMS ISOCRATIC 50% B.M_REDUCED FLOW.M	**Injection volume:**	2.000
**Analysis method:**	LCMS ISOCRATIC 50% B.M_REDUCED FLOW.M	**Acq. operator:**	SYSTEM
**Last changed:**	10/21/2016 12:01:19 PM		

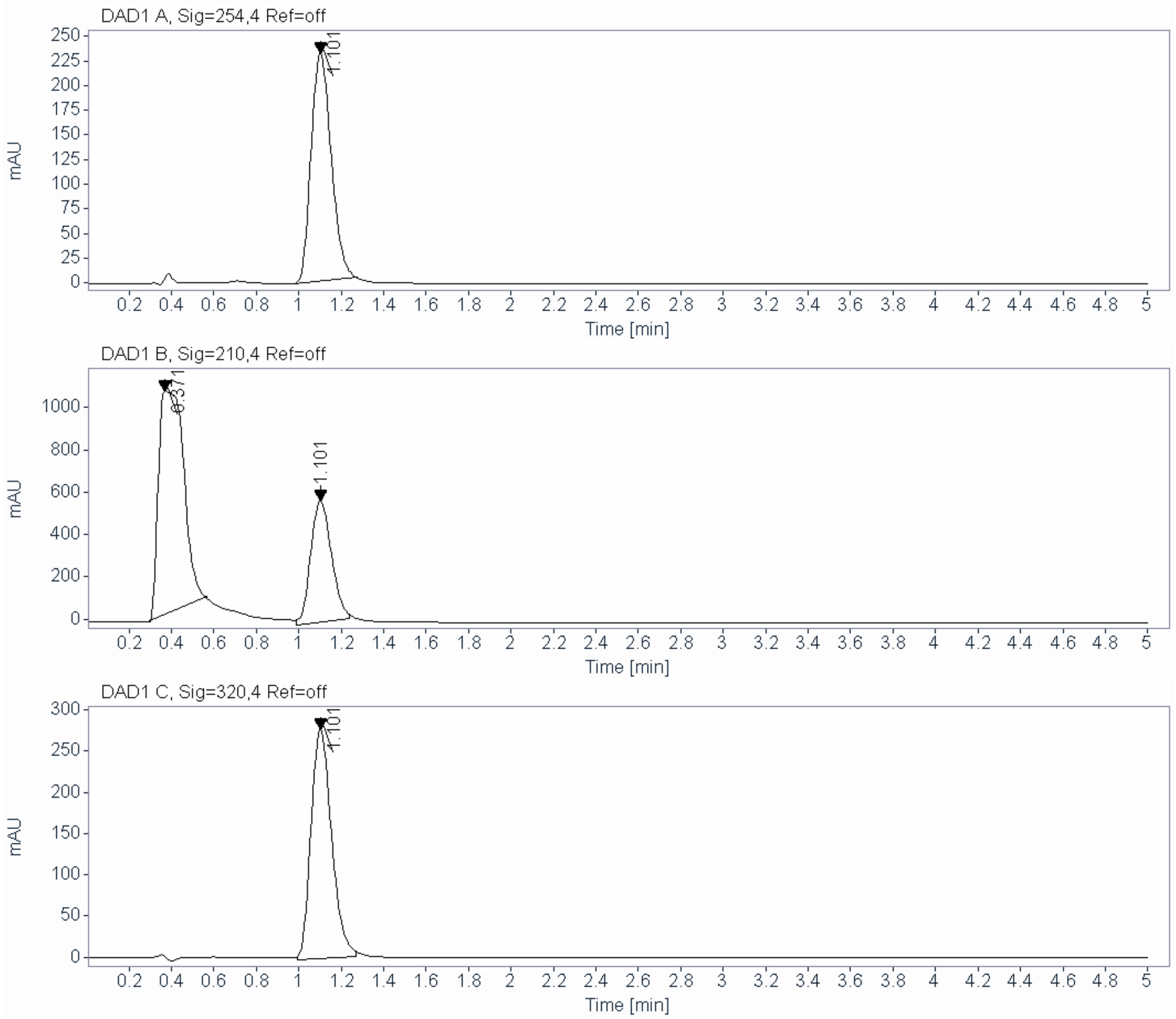

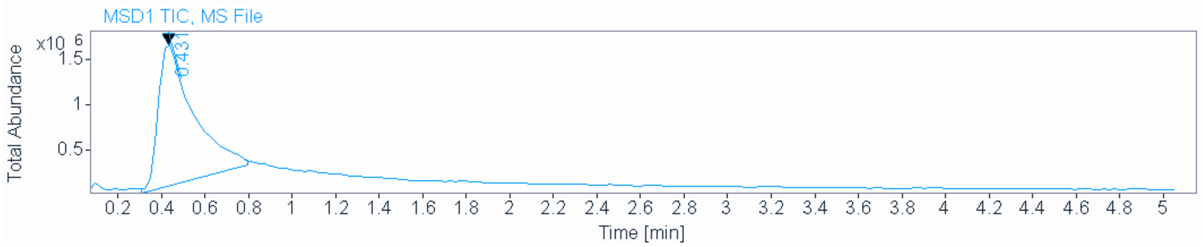

Agilent Technologies

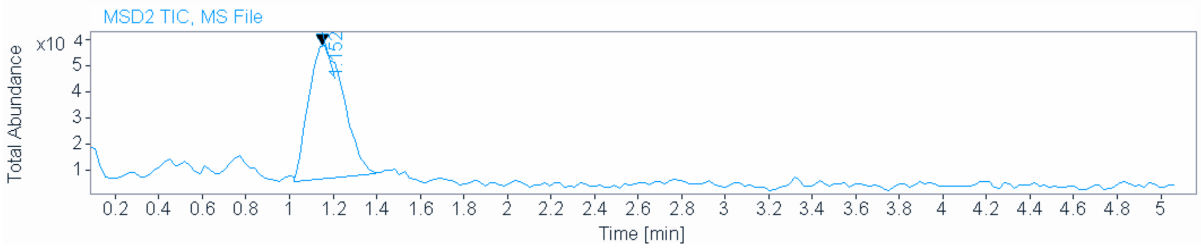

Signal: DAD1 A, Sig=254,4 Ref=off

RT [min]	Type	Width [min]	Area	Height	Area%	Name
1.101	MM	0.1094	1522.5050	231.8956	100.0000	
		Sum	1522.5050			

Signal: DAD1 B, Sig=210,4 Ref=off

RT [min]	Type	Width [min]	Area	Height	Area%	Name
0.371	MM	0.1361	8620.7959	1055.6846	68.8027	
1.101	MM	0.1135	3908.9382	574.0224	31.1973	
		Sum	12529.7341			

Signal: DAD1 C, Sig=320,4 Ref=off

RT [min]	Type	Width [min]	Area	Height	Area%	Name
1.101	MM	0.1122	1876.9137	278.6975	100.0000	
		Sum	1876.9137			

Signal: MSD1 TIC, MS File

RT [min]	Type	Width [min]	Area	Height	Area%	Name
0.431	MM	0.1846	17550388.0000	1584458.1250	100.0000	
		Sum	17550388.00			

Signal: MSD2 TIC, MS File

RT [min]	Type	Width [min]	Area	Height	Area%	Name
1.152	MM	0.1813	565009.1875	51948.8594	100.0000	
		Sum	565009.1875			

Agilent Technologies

0.431 - 0.439 (002-45-KP5139.D)

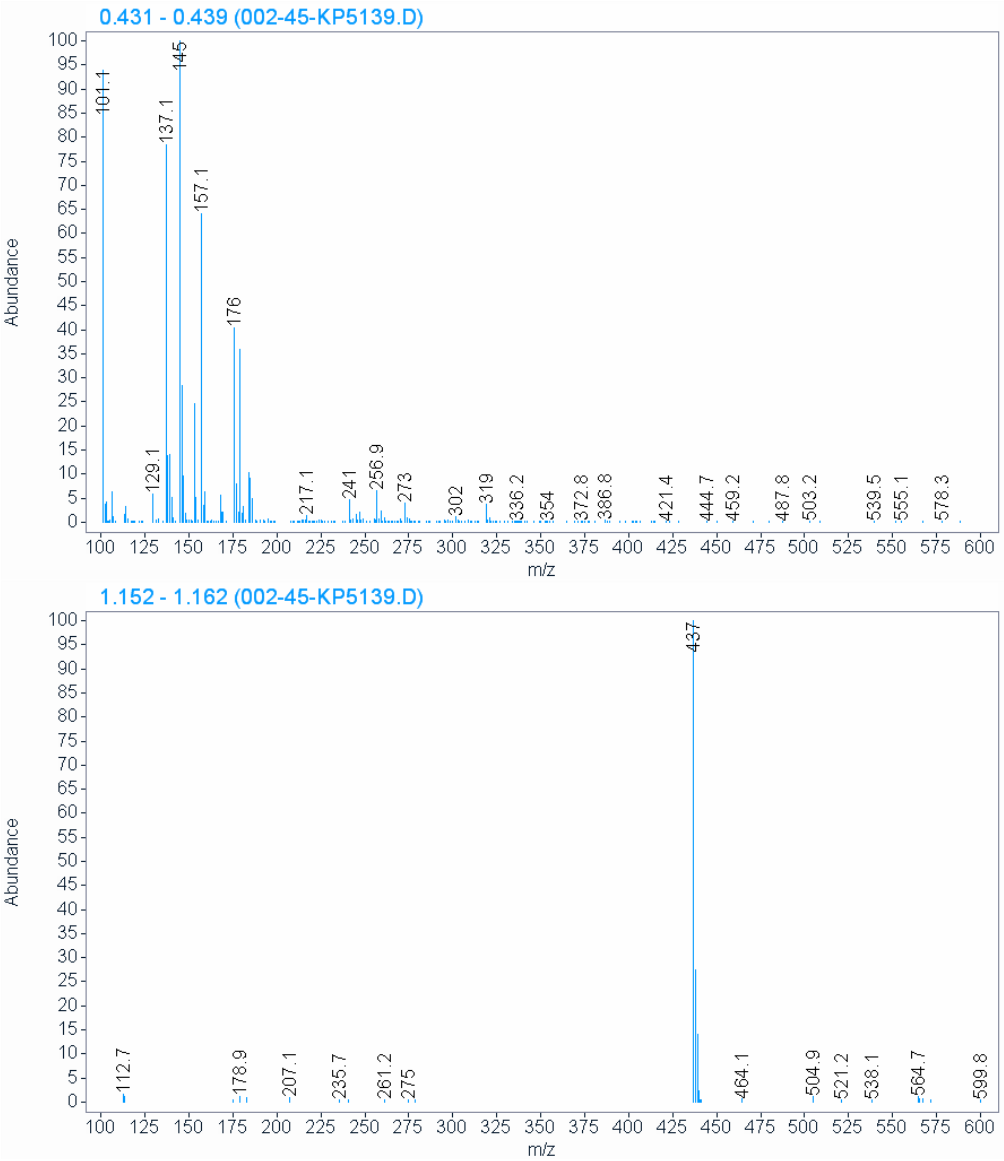

Compound Name: (*Z*)-3-((2-(naphthalene-1-sulfonamido)-4-oxothiazol-5(4*H*)-ylidene)methyl)benzoic acid

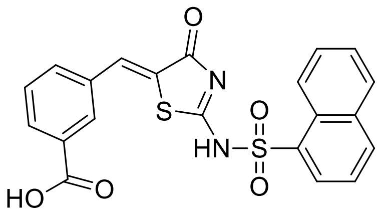

Chemical Formula: $C_{21}H_{14}N_2O_5S_2$
Exact Mass: 438.03
Molecular Weight: 438.48

Compound Code: 10 (KP6015)

Obtained Weight & Yield: 179 mg (82%)

Purity (by LCMS and ^{1}H NMR): > 97% by ^{1}H-NMR

Appearance: Pale pink powder

Solubility: DMSO

Melting Point: > 162 °C (dec.)

TLC Rf (and conditions): 0.08 (10% MeOH in DCM)

IR Analysis (including assignment): IR (neat): ν_{max} = 3289 (O-H), 3023, 2519 (C-H aromatic), 1726 (C=O), 1679 (carboxylic acid), 1561 (C-C aromatic), 1116 (C-N) cm^{-1}

^{1}H NMR Analysis: ^{1}H NMR (400 MHz, DMSO) δ 13.36 (broad s, 1H, NH), 8.60 (d, *J* = 8.6 Hz, 1H), 8.32 – 8.28 (m, 2H), 8.22 (s, 1H), 8.12 (d, *J* = 8.1 Hz, 1H), 8.06 (d, *J* = 7.8 Hz, 1H), 7.92 (d, *J* = 7.7 Hz, 1H), 7.85 (s, 1H), 7.79 – 7.67 (m, 4H) ppm.
OH exchanging – not visible
Methanol 3.17 ppm (2.33%)

^{13}C NMR Analysis: ^{13}C NMR (151 MHz, DMSO) δ 166.6 (2C), 165.4, 135.2, 134.8, 134.5, 133.8, 133.3, 132.6, 131.9, 131.2, 130.3, 129.9, 129.0, 128.4, 128.2, 127.6, 127.1, 124.8, 124.6, 123.2 ppm.
2C assignment by 2D NMR

MS Analysis (low res): LRMS (ESI) *m/z* (%): 437 (M-H, $C_{21}H_{14}N_2O_2S_2$)

HPLC method details: Column: Zorbax SB-C18 Rapid Resolution HT 2.1x50mm 1.8-Micron; Method: LCMS ISOCRATIC 50% B.M_REDUCED FLOW.M filename: KP5139; Peak retention time: 1.13 mins; Area (%): 100.

Procedure: To a microwave vial was added the *N*-(4-oxo-4,5-dihydrothiazol-2-yl)naphthalene-1-sulfonamide (151 mg, 0.49 mmol), 3-carboxybenzaldehyde (87 mg, 0.58 mmol, 1.2 eq), the benzoic acid/piperidine catalyst (approximately 10 drops) and ethanol (3 mL). The suspension was heated by microwave irradiation (200W, 120 °C) for 30 min after which water was added dropwise until a precipitate formed. The solution was filtered and washed with diethyl ether to give the desired product (179 mg, 82%).

Other analyses, reference papers, previously obtained data, comments, etc:

PerkinElmer Spectrum Version 10.4.00
Monday, 27 May 2019 1:36 PM

Analyst research
Date Monday, 27 May 2019 1:36 PM

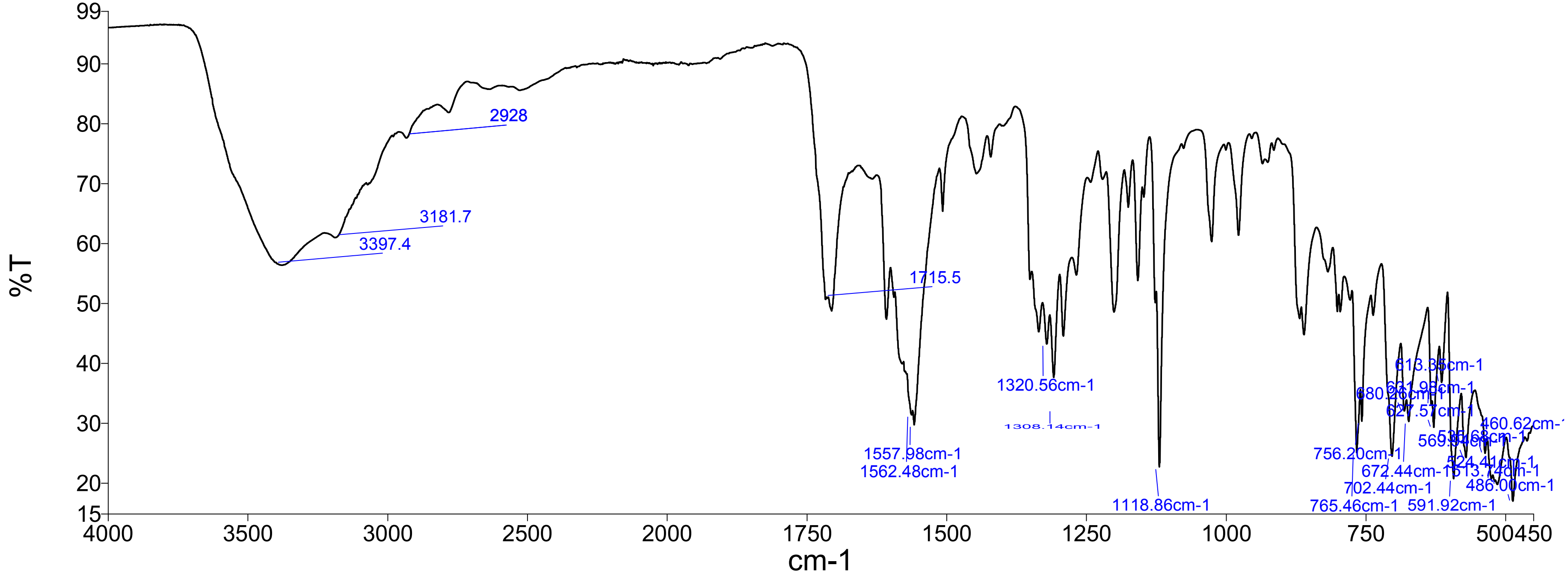

Sample Name	Description	Quality Checks
KP6015	Sample 070 By class Date Wednesday, May 08 2019	The Quality Checks do not report any warnings for the sample.

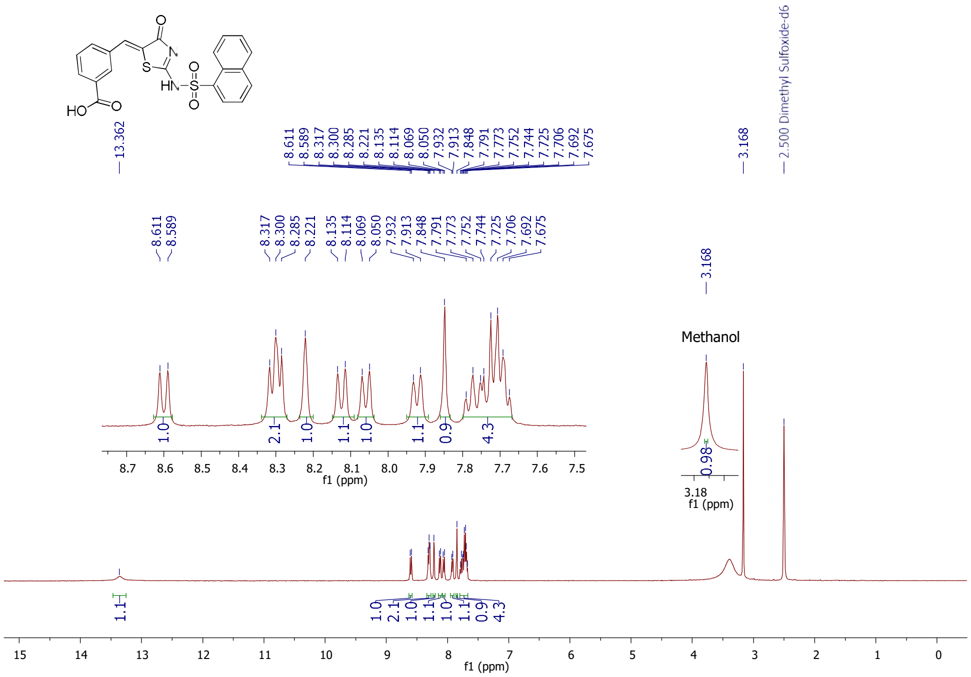

HO
O
S
N
HN
S
O
O
13.362
8.611
8.589
8.317
8.300
8.285
8.221
8.135
8.114
8.069
8.050
7.932
7.913
7.848
7.791
7.773
7.752
7.744
7.725
7.706
7.692
7.675
3.168
2.500 Dimethyl Sulfoxide-d6
Methanol
0.98
3.18
f1 (ppm)
1.0
2.1
1.0
1.1
1.0
1.1
0.9
4.3
1.1
f1 (ppm)

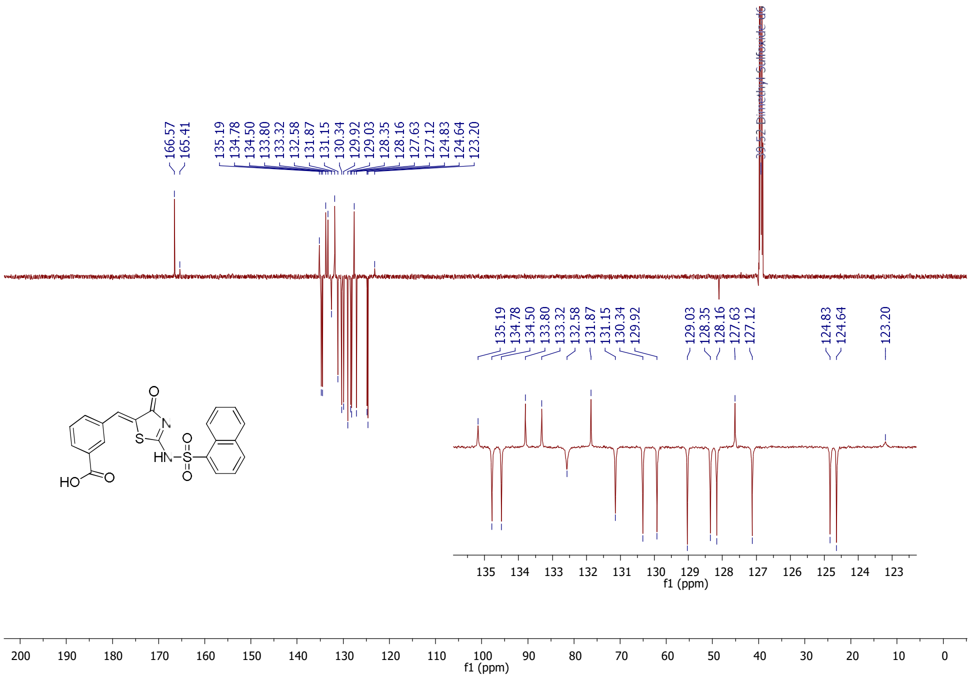

166.57
165.41
135.19
134.78
134.50
133.80
133.32
132.58
131.87
131.15
130.34
129.92
129.03
128.35
128.16
127.63
127.12
124.83
124.64
123.20
39.52 Dimethyl Sulfoxide-d6
HO
O
S
N
HN
S
O
O
f1 (ppm)
f1 (ppm)

Agilent Technologies

Data file: D:\Chem32\1\Data\KP\PRE 11-6-19\KP5189-6020 2019-05-10 13-00-54\004-75-KP6015.D
Sample name: KP6015
Description:
Sample amount: 0.000
Sample type: Sample

Instrument: LCMS
Location: 75
Injection date: 5/10/2019 1:22:34 PM
Injection: 1 of 1
Acq. method: LCMS ISOCRATIC 50% B.M_REDUCED FLOW.M
Injection volume: 2.000
Analysis method: LCMS ISOCRATIC 50% B.M_REDUCED FLOW.M
Acq. operator: SYSTEM
Last changed: 10/21/2016 12:01:19 PM

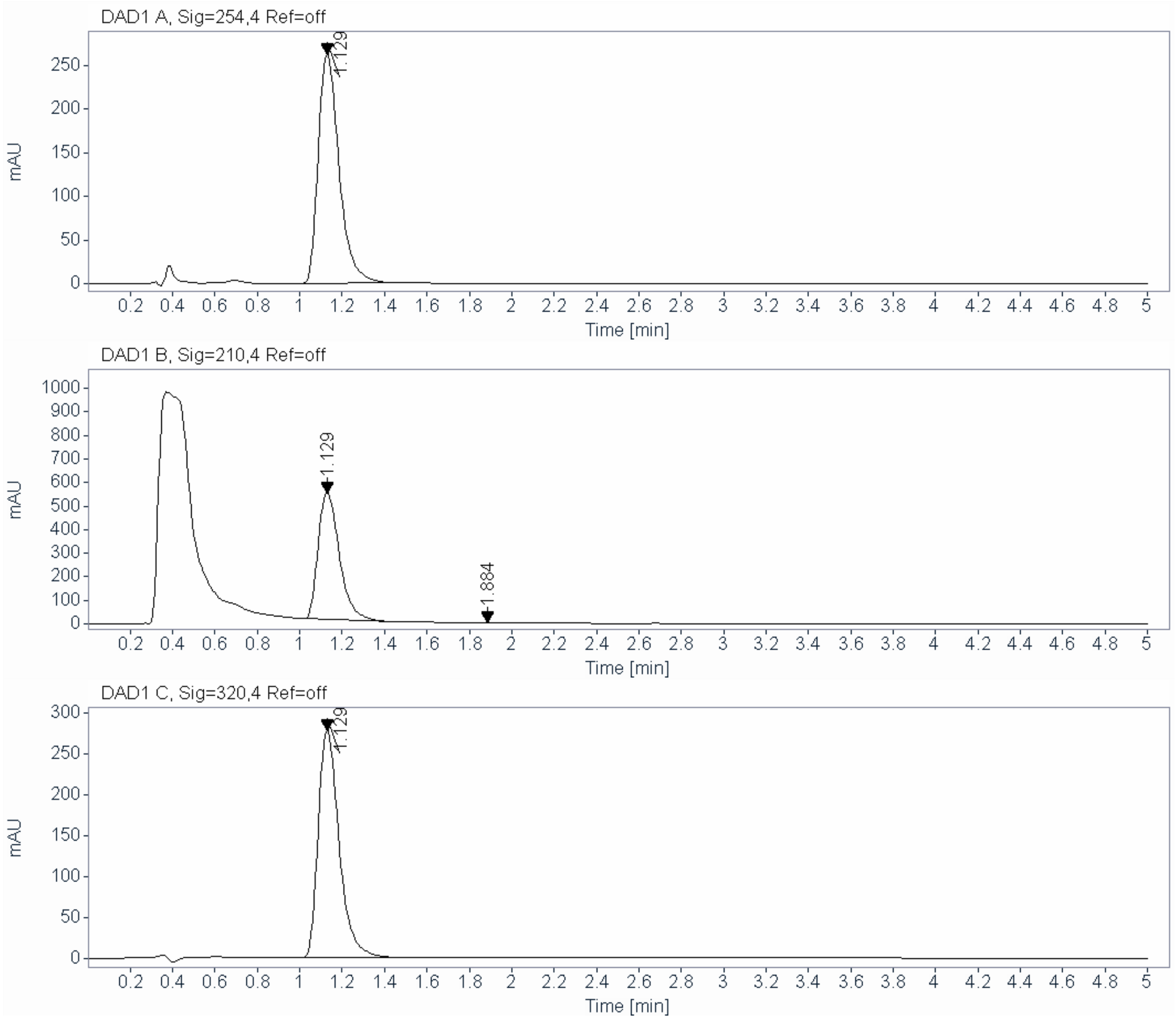

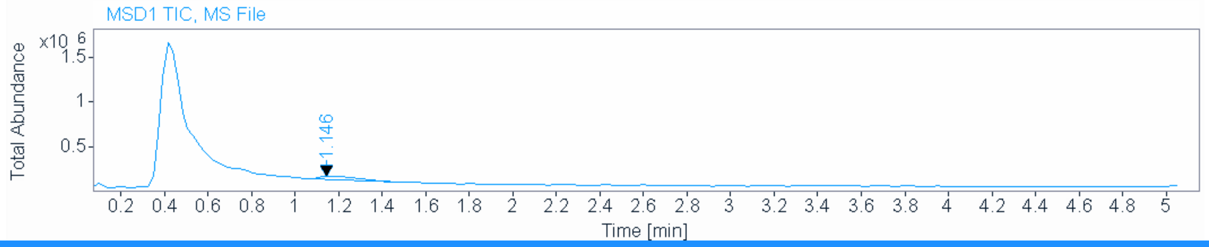

Agilent Technologies

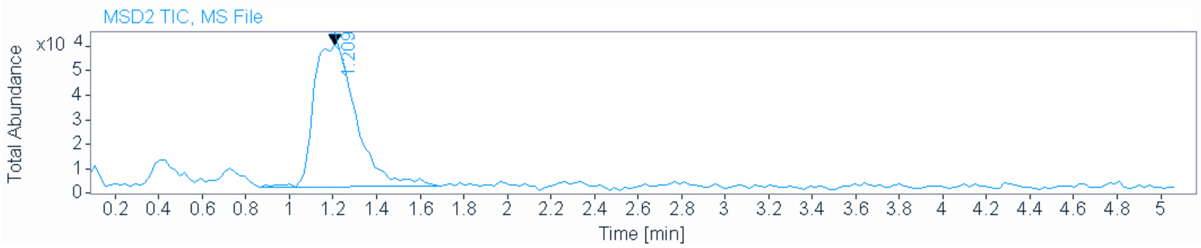

Signal: DAD1 A, Sig=254,4 Ref=off

RT [min]	Type	Width [min]	Area	Height	Area%	Name
1.129	BB	0.1057	1797.6929	263.0273	100.0000	
		Sum	1797.6929			

Signal: DAD1 B, Sig=210,4 Ref=off

RT [min]	Type	Width [min]	Area	Height	Area%	Name
1.129	BB	0.1073	3753.1118	538.4868	99.6049	
1.884	BB	0.1198	14.8864	1.7067	0.3951	
		Sum	3767.9982			

Signal: DAD1 C, Sig=320,4 Ref=off

RT [min]	Type	Width [min]	Area	Height	Area%	Name
1.129	BB	0.1062	1923.0734	279.6395	100.0000	
		Sum	1923.0734			

Signal: MSD1 TIC, MS File

RT [min]	Type	Width [min]	Area	Height	Area%	Name
1.146	BB	0.1648	520314.5625	40197.4883	100.0000	
		Sum	520314.5625			

Signal: MSD2 TIC, MS File

RT [min]	Type	Width [min]	Area	Height	Area%	Name
1.209	BB	0.2071	785847.9375	57671.1445	100.0000	
		Sum	785847.9375			

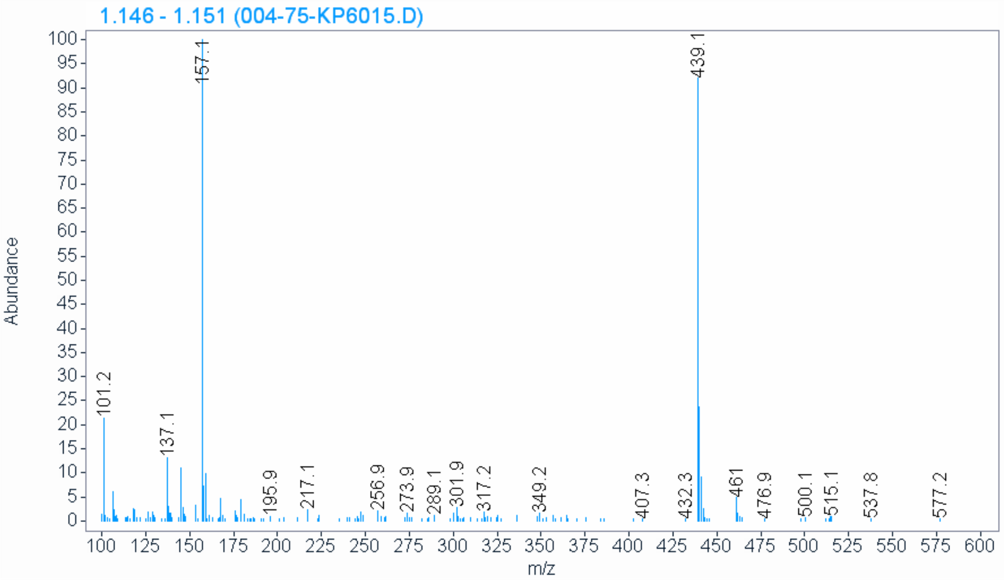
1.146 - 1.151 (004-75-KP6015.D)
Abundance
m/z
101.2
137.1
157.1
195.9
217.1
256.9
273.9
289.1
301.9
317.2
349.2
407.3
432.3
439.1
461
476.9
500.1
515.1
537.8
577.2

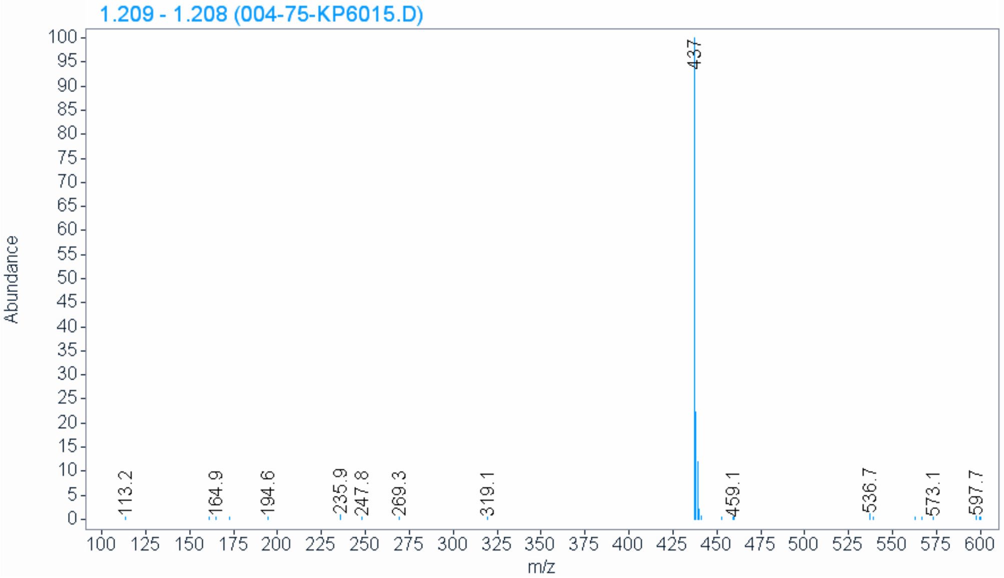
1.209 - 1.208 (004-75-KP6015.D)
Abundance
m/z
113.2
164.9
194.6
235.9
247.8
269.3
319.1
437
459.1
536.7
573.1
597.7

Compound Name: (*Z*)-*N*-(5-(4-cyanobenzylidene)-4-oxo-4,5-dihydrothiazol-2-yl)naphthalene-1-sulfonamide

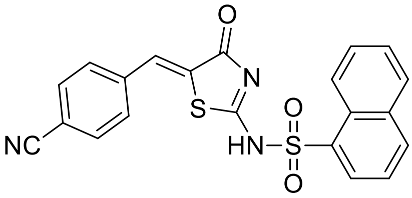

Chemical Formula: $C_{21}H_{13}N_3O_3S_2$
Exact Mass: 419.04
Molecular Weight: 419.48

Compound Code: 11 (KP7114)

Obtained Weight & Yield: 143 mg, 70%

Purity (by LCMS and ^{1}H NMR): >98% by ^{1}H-NMR, >99% by LCMS

Appearance: pale orange

Solubility: DMSO, slightly soluble in acetone and methanol

Melting Point: > 296 °C (dec.)

TLC Rf (and conditions): N/A

IR Analysis (including assignment): IR (neat): 3166 (N-H), 3049, 2965 (C-H aromatic), 2215 (CN nitrile), 1711 (C=O), 1553 (aromatic C-C), 1348 (sulfonamide), 1131 (C-N)

^{1}H NMR Analysis: ^{1}H NMR (400 MHz, DMSO) δ 8.61 (d, *J* = 8.6 Hz, 1H), 8.30 (d, *J* = 7.6 Hz, 2H), 8.12 (d, *J* = 7.9 Hz, 1H), 8.04 (d, *J* = 8.4 Hz, 2H), 7.85 – 7.82 (m, 3H), 7.77 (ddd, *J* = 8.5, 6.9, 1.4 Hz, 1H), 7.73 – 7.67 (m, 2H) ppm.
NH exchanging – not visible
Ethanol impurity at 1.05 ppm (1.44%)

^{13}C NMR Analysis: ^{13}C NMR (101 MHz, DMSO) δ 166.5, 165.2, 137.3, 135.1, 134.8, 133.8, 133.2 (2C), 131.2, 130.6 (2C), 129.0, 128.4, 128.2, 127.6, 127.1, 125.6, 124.8, 124.7, 118.4, 112.3 ppm.
2C determined by 2D NMR.

MS Analysis (low res): LRMS (ESI-) *m/z* (%): 418 (*M*-H, $C_{21}H_{13}N_3O_3S_2$, 100%);

MS Analysis (high res): Exact mass calculated for $C_{21}H_{13}N_3O_3S_2$ [*M*-H]$^-$, 418.0300. Found 418.0325.

HPLC method details: Column: Zorbax SB-C18 Rapid Resolution HT 2.1x50mm 1.8-Micron; Method: LCMS ISOCRATIC 60%B 0.4MLMIN-1.M filename: KP7114; Peak retention time: 1.118 mins; Area (%): 100

Procedure: To a 10mL microwave vial was added the *N*-(4-oxo-4,5-dihydrothiazol-2-yl)naphthalene-1-sulfonamide (154 mg, 0.49 mmol), 4-cyanobenzaldehyde (80 mg, 0.55 mmol, 1.1 eq), ethanol (3 mL) and a catalytic amount of the benzoic acid/piperidine catalyst (approximately 5 drops). The suspension was heated using microwave irradiation (200 W, 120 °C) for 30 min then allowed to precipitate at in the freezer. The resulting precipitate was collected by vacuum filtration and washed with cold ethanol and cold ether to give the desired product (143 mg, 70%)

Other analyses, reference papers, previously obtained data, comments, etc:

PerkinElmer Spectrum Version 10.4.00
Thursday, 21 November 2019 11:38 AM

Analyst research
Date Thursday, 21 November 2019 11:38 AM

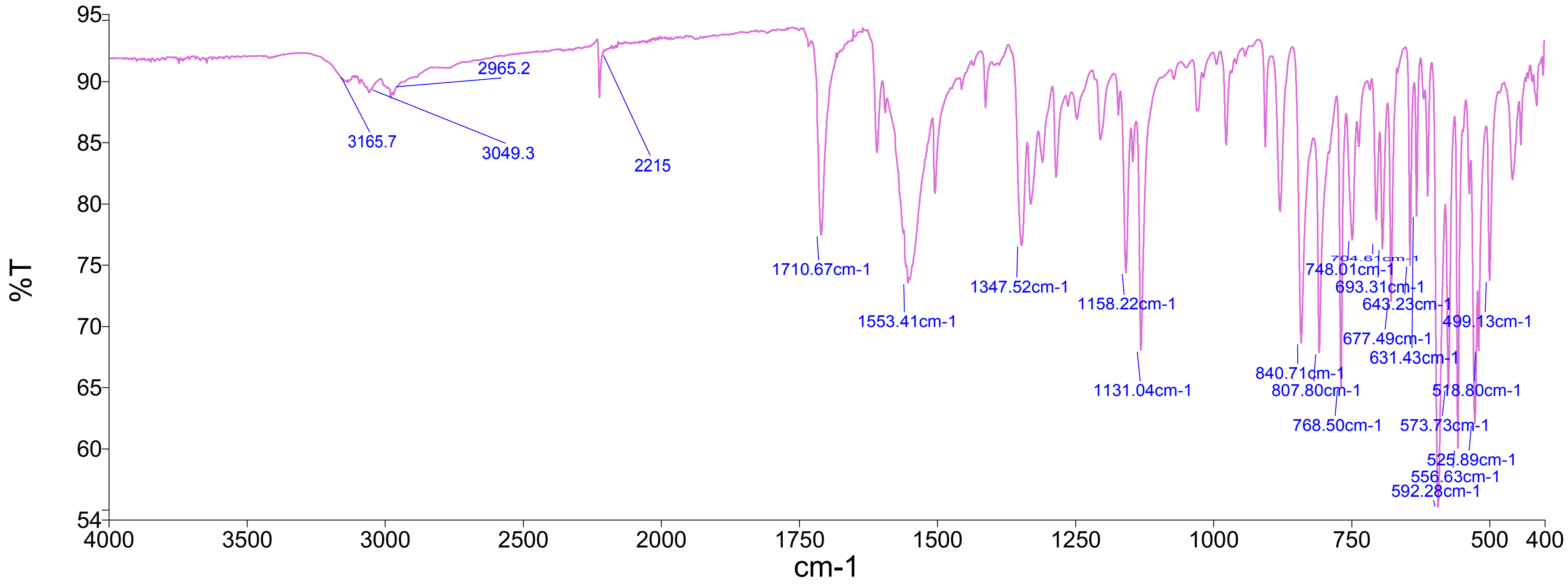

Sample Name	Description	Quality Checks
KP7114	Sample 249 By research Date Thursday, November 21 2019	The Quality Checks do not report any warnings for the sample.

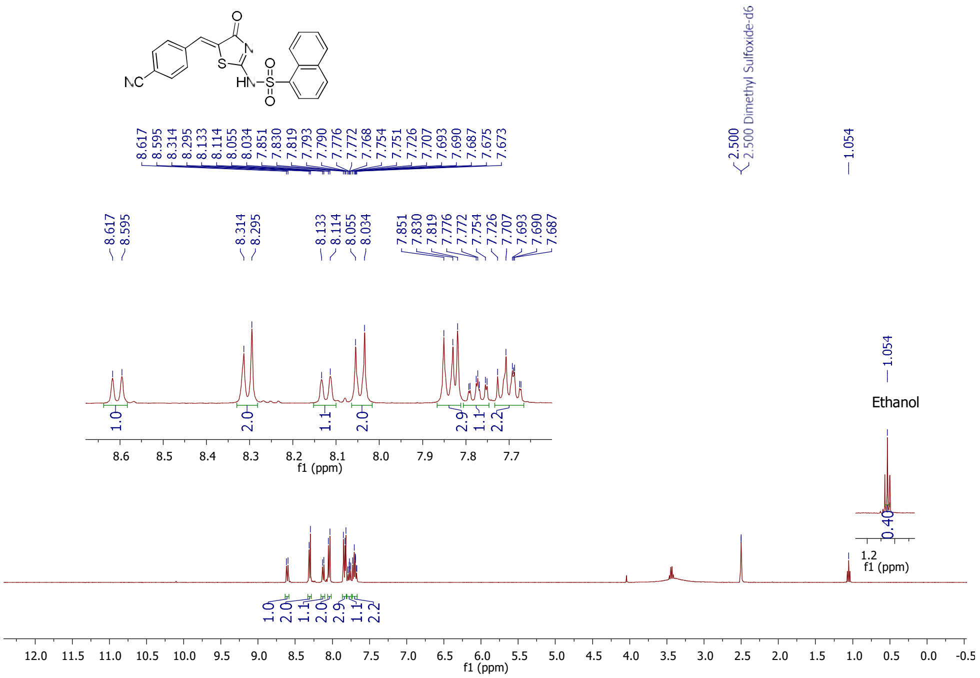
2.500
2.500 Dimethyl Sulfoxide-d6
1.054
8.617
8.595
8.314
8.295
8.133
8.114
8.055
8.034
7.851
7.830
7.819
7.793
7.790
7.776
7.772
7.768
7.754
7.751
7.726
7.707
7.693
7.690
7.687
7.675
7.673
Ethanol
1.0
2.0
1.1
2.0
2.9
1.1
2.2
0.40
f1 (ppm)

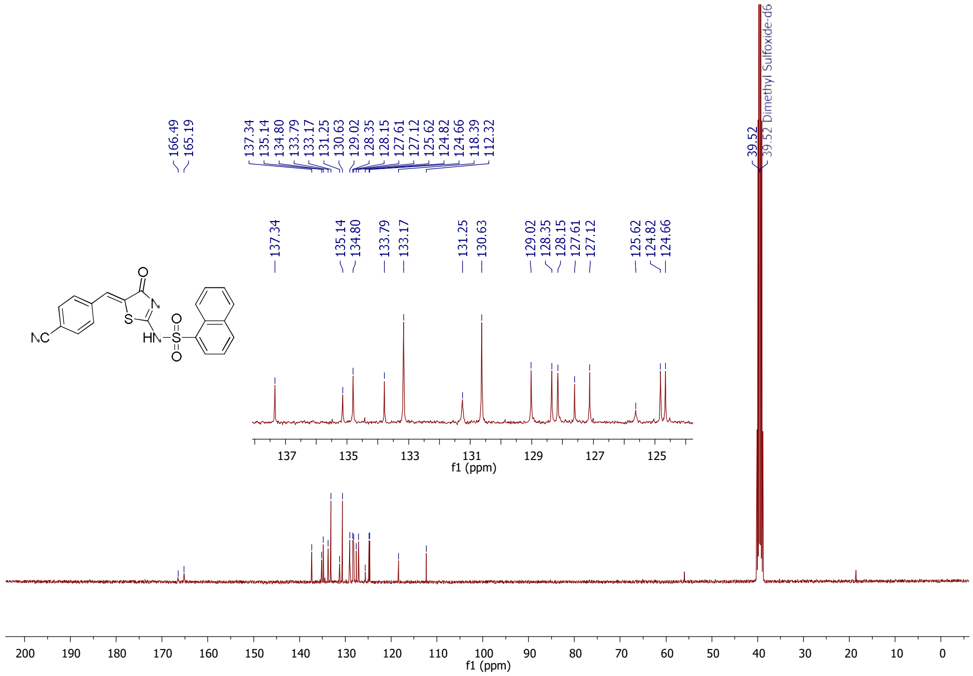
166.49
165.19
137.34
135.14
134.80
133.79
133.17
131.25
130.63
129.02
128.35
128.15
127.61
127.12
125.62
124.82
124.66
118.39
112.32
39.52
39.52 Dimethyl Sulfoxide-d6
f1 (ppm)

LCMS Report

Agilent Technologies

**Data file:**	D:\Chem32\1\Data\KP_DS_IND2 2019-11-15 11-06-04\005-47-KP7114.D		
**Sample name:**	KP7114		
**Description:**			
**Sample amount:**	0.000	**Sample type:**	Sample
**Instrument:**	LCMS	**Location:**	47
**Injection date:**	11/15/2019 11:38:13 AM	**Injection:**	1 of 1
**Acq. method:**	LCMS ISOCRATIC 60% B 0.4MLMIN-1.M	**Injection volume:**	2.000
**Analysis method:**	LCMS ISOCRATIC 60%B 0.4MLMIN-1.M	**Acq. operator:**	SYSTEM
**Last changed:**	5/8/2019 8:55:04 AM		

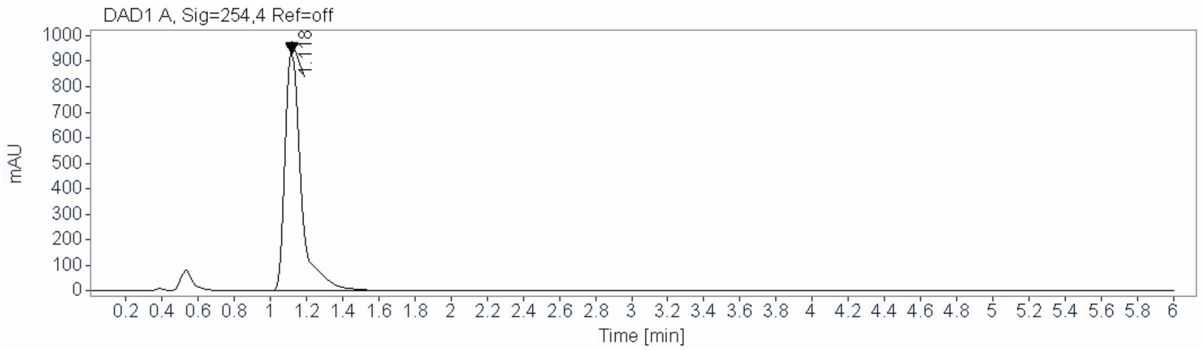

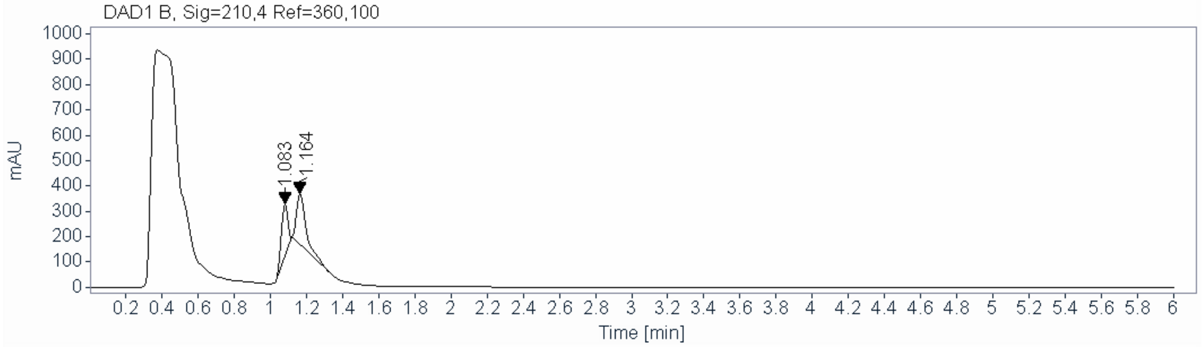

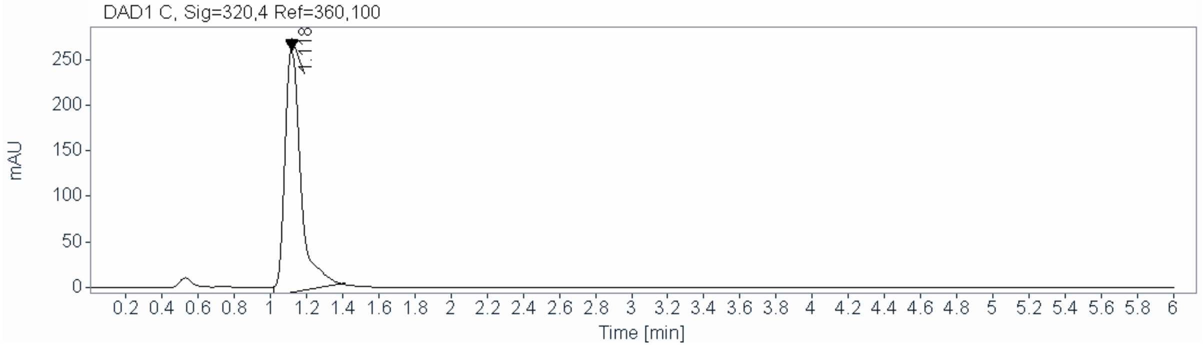

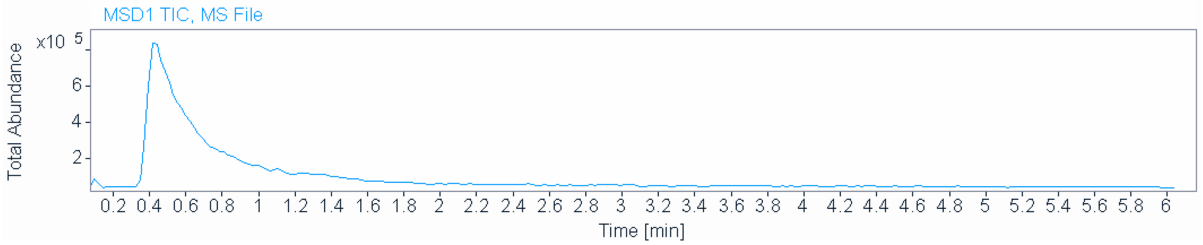

Agilent Technologies

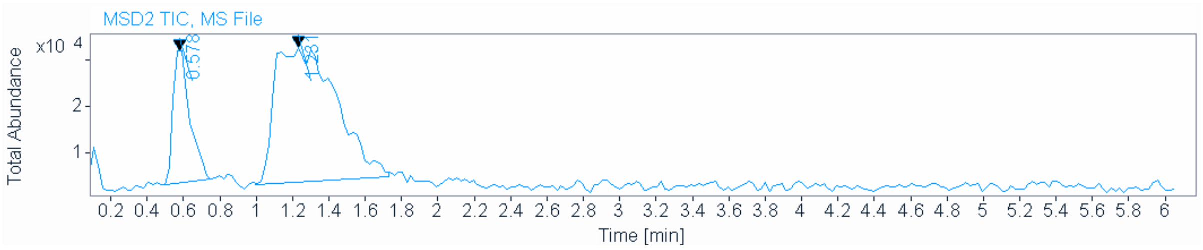

Signal: DAD1 A, Sig=254,4 Ref=off

RT [min]	Type	Width [min]	Area	Height	Area%	Name
1.118	BB	0.0949	5829.8579	932.6088	100.0000	
		Sum	5829.8579			

Signal: DAD1 B, Sig=210,4 Ref=360,100

RT [min]	Type	Width [min]	Area	Height	Area%	Name
1.083	BB	0.0428	524.3751	198.8542	40.0876	
1.164	BB	0.0592	783.6989	202.2733	59.9124	
		Sum	1308.0740			

Signal: DAD1 C, Sig=320,4 Ref=360,100

RT [min]	Type	Width [min]	Area	Height	Area%	Name
1.118	MM	0.1022	1633.7550	266.3363	100.0000	
		Sum	1633.7550			

Signal: MSD2 TIC, MS File

RT [min]	Type	Width [min]	Area	Height	Area%	Name
0.578	BB	0.1014	185048.1406	29237.9063	21.4369	
1.231	MM	0.3902	678174.5625	28963.5566	78.5631	
		Sum	863222.7031			

Agilent Technologies

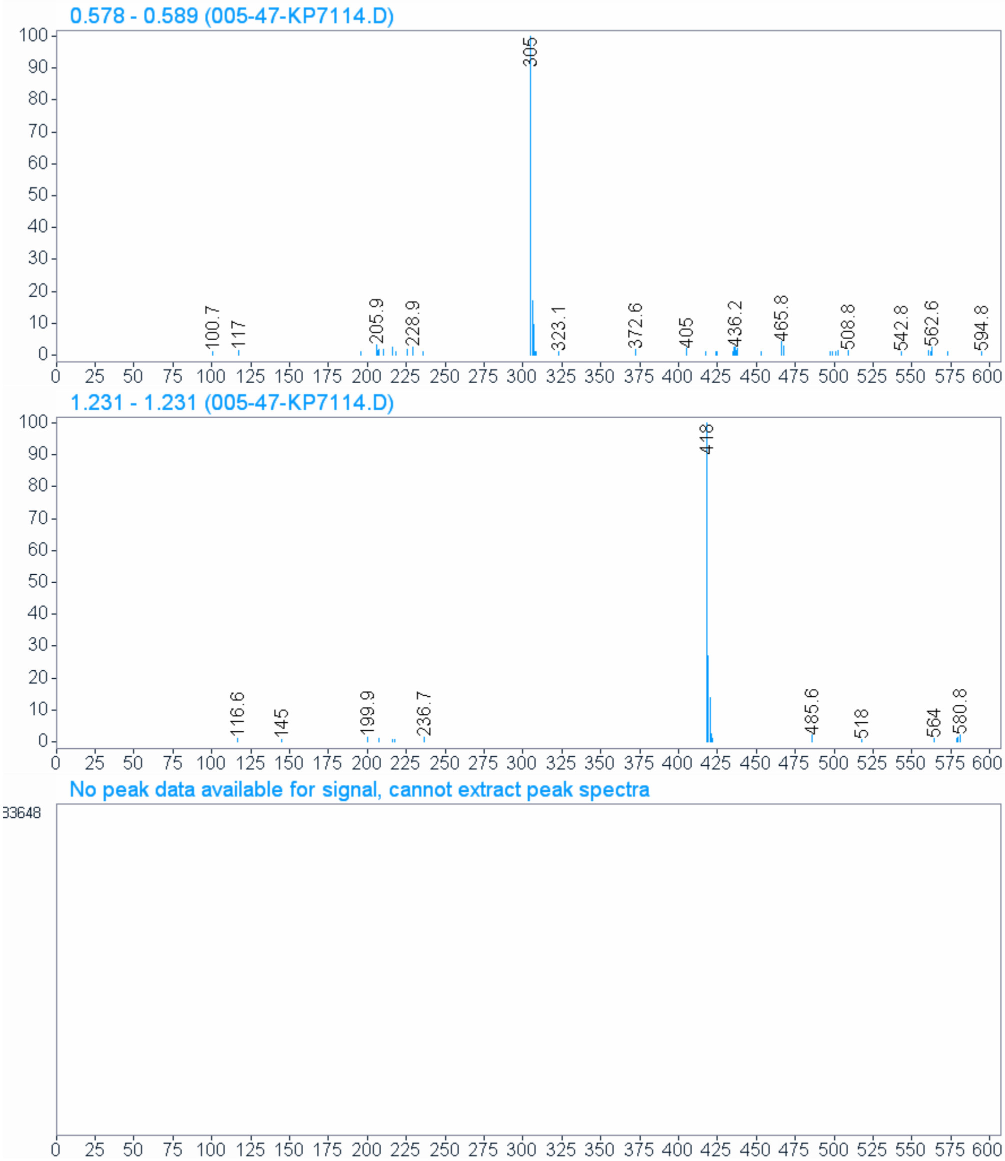

Compound Name: (Z)-N-(5-(3-cyanobenzylidene)-4-oxo-4,5-dihydrothiazol-2-yl)naphthalene-1-sulfonamide

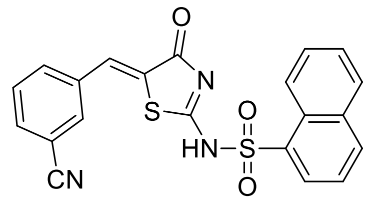

Chemical Formula: $C_{21}H_{13}N_3O_3S_2$
Exact Mass: 419.04
Molecular Weight: 419.48

Compound Code: 12 (KP7117)

Obtained Weight & Yield: 96 mg, 47%

Purity (by LCMS and ^{1}H NMR): >98% by ^{1}H-NMR, >98% by LCMS

Appearance: peach coloured solid

Solubility: DMSO, slightly soluble in acetone and methanol

Melting Point: > 248 °C (dec.)

TLC Rf (and conditions): N/A

IR Analysis (including assignment): IR (neat): 2965 (C-H aromatic), 2816 (C-H), 2234 (CN nitrile), 1708 (C=O), 1564 (aromatic C-C), 1121 (C-N)

^{1}H NMR Analysis: ^{1}H NMR (400 MHz, DMSO) δ 8.60 (d, *J* = 8.6 Hz, 1H), 8.31 (d, *J* = 7.7 Hz, 2H), 8.14 – 8.11 (m, 2H), 7.97 (t, *J* = 7.4 Hz, 2H), 7.84 – 7.75 (m, 3H), 7.70 (td, *J* = 7.4, 5.0 Hz, 2H) ppm.
NH exchanging – not visible.
Ether solvent impurity at 1.09 ppm (1.34%)

^{13}C NMR Analysis: ^{13}C NMR (101 MHz, DMSO) δ 166.6 (br), 165.3, 155.2 (br), 135.2, 134.8, 134.3, 134.2, 133.8, 133.7, 133.4, 131.1, 130.7, 129.0, 128.3, 128.2, 127.6, 127.1, 124.8, 124.7, 118.2, 112.6 ppm.
Broad peaks determined by 2D NMR

MS Analysis (low res): LRMS (ESI-) *m/z* (%): 418 (*M*-H, $C_{21}H_{12}N_3O_3S_2$, 100%); (ESI-) *m/z* (%): 420 (*M*+H, $C_{21}H_{14}N_3O_3S_2$, 100%);

HPLC method details: Column: Zorbax SB-C18 Rapid Resolution HT 2.1x50mm 1.8-Micron; Method: LCMS ISOCRATIC 60%B 0.4MLMIN-1.M filename: KP7117; Peak retention time: 1.086 mins; Area (%): 98

Procedure: To a 10mL microwave vial was added the *N*-(4-oxo-4,5-dihydrothiazol-2-yl)naphthalene-1-sulfonamide (152 mg, 0.49 mmol), 3-cyanobenzaldehyde (77 mg, 0.55 mmol, 1.1 eq), ethanol (3 mL) and a catalytic amount of the benzoic acid/piperidine catalyst (approximately 5 drops). The suspension was heated using microwave irradiation (200 W, 120 °C) for 30 min then allowed to precipitate at in the freezer. The resulting precipitate was collected by vacuum filtration and washed with cold ethanol and cold ether to give the desired product (96 mg, 47%)

Other analyses, reference papers, previously obtained data, comments, etc:

PerkinElmer Spectrum Version 10.4.00
Thursday, 21 November 2019 11:38 AM

Analyst research

Date Thursday, 21 November 2019 11:38 AM

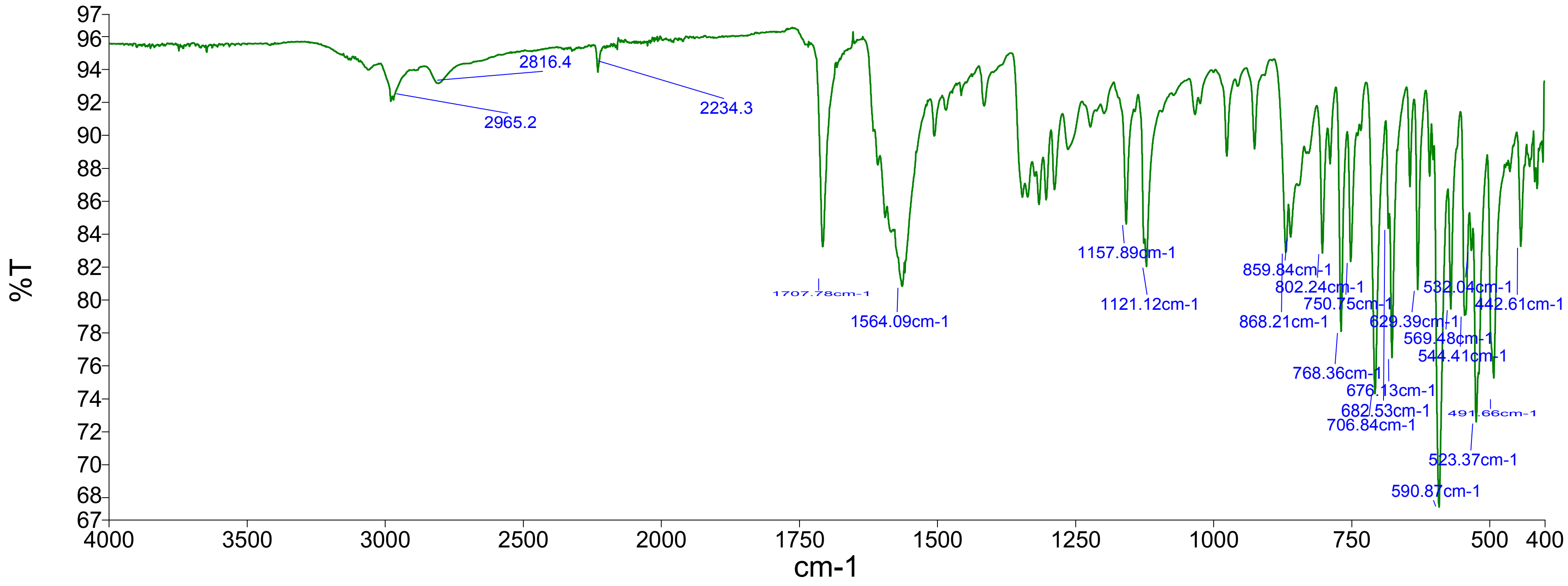

Sample Name	Description	Quality Checks
KP7117	Sample 250 By research Date Thursday, November 21 2019	The Quality Checks give rise to a Weak Bands warning for the sample.

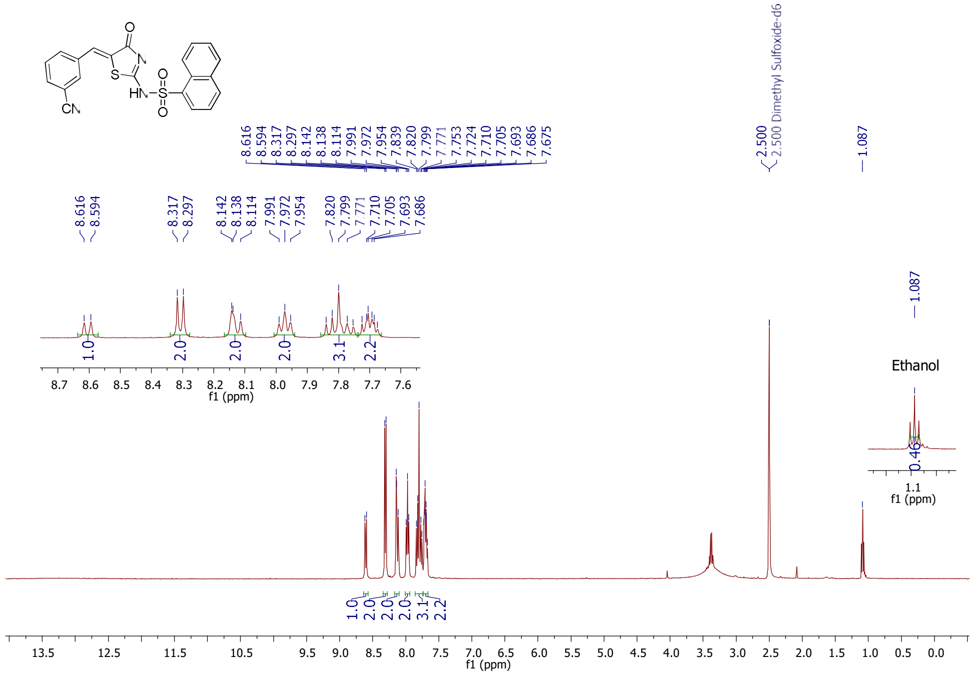

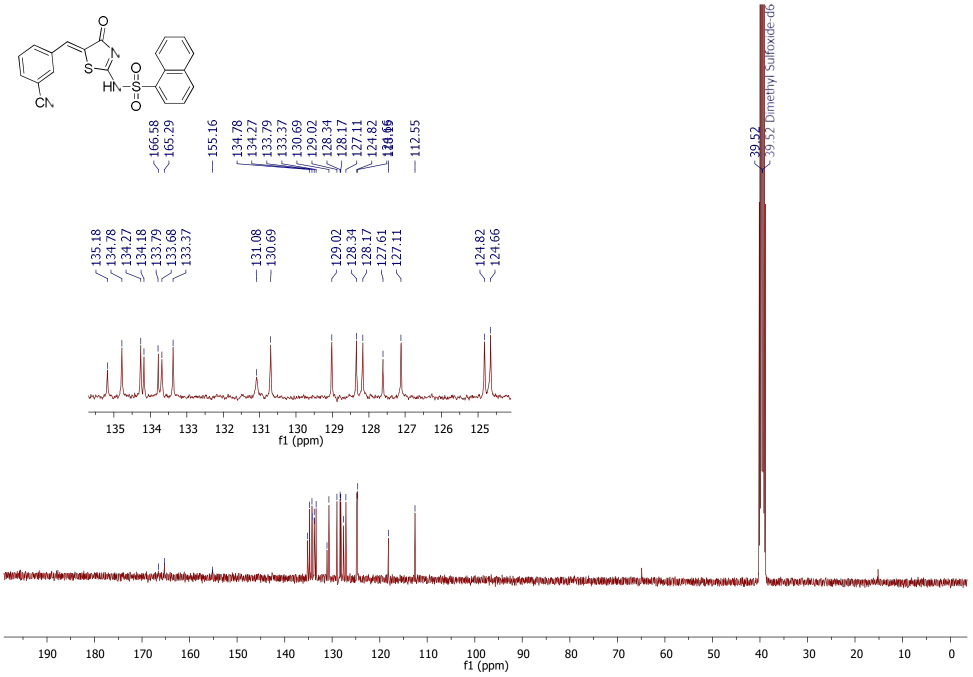

LCMS Report

Agilent Technologies

Data file: D:\Chem32\1\Data\KP_DS_IND3 2019-11-15 12-19-28\001-38-KP7117.D
Sample name: KP7117
Description:
Sample amount: 0.000 **Sample type:** Sample

Instrument: LCMS **Location:** 38
Injection date: 11/15/2019 12:21:06 PM **Injection:** 1 of 1
Acq. method: LCMS ISOCRATIC 60% B 0.4MLMIN-1.M **Injection volume:** 2.000
Analysis method: LCMS ISOCRATIC 60%B 0.4MLMIN-1.M **Acq. operator:** SYSTEM
Last changed: 5/8/2019 8:55:04 AM

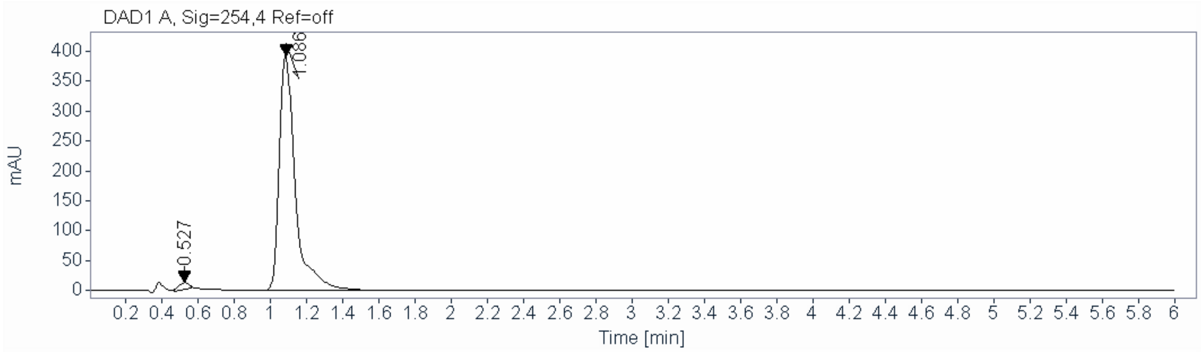

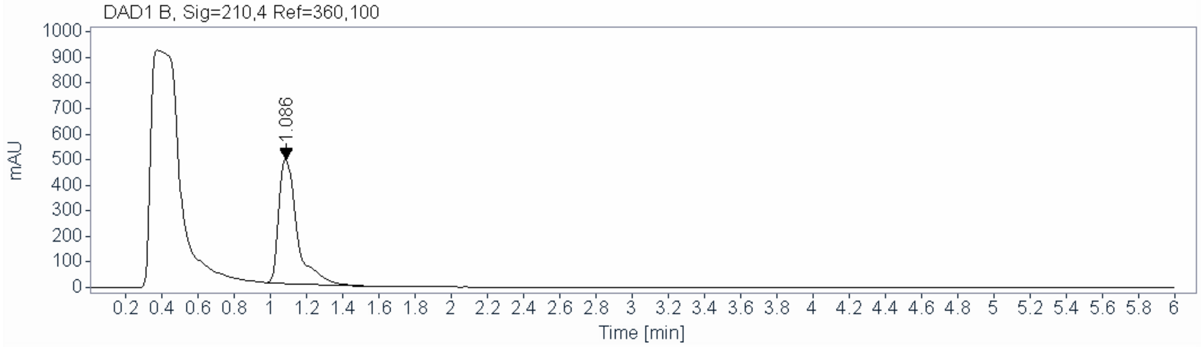

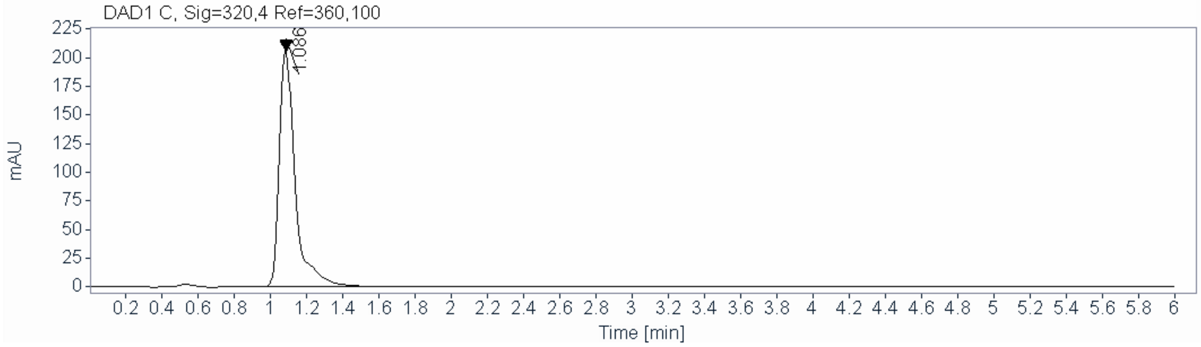

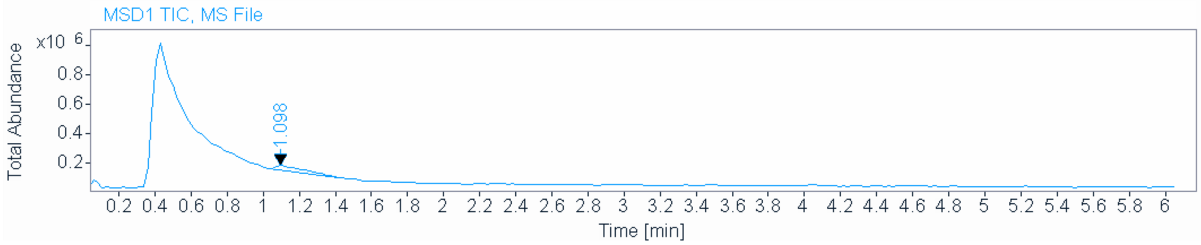

Agilent Technologies

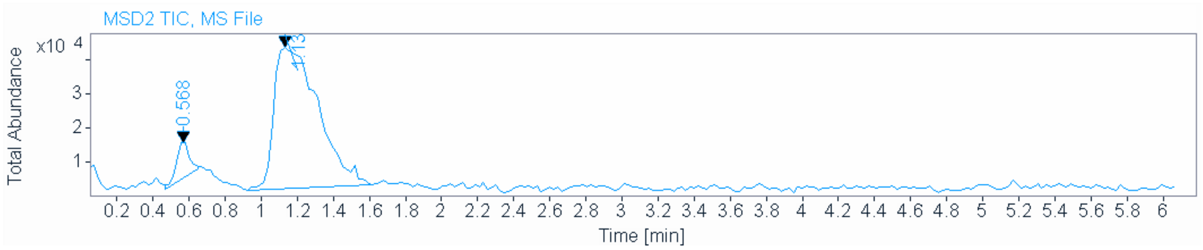

Signal: DAD1 A, Sig=254,4 Ref=off

RT [min]	Type	Width [min]	Area	Height	Area%	Name
0.527	MM	0.0607	40.1338	11.0146	1.6120	
1.086	BB	0.0946	2449.5618	393.7860	98.3880	
		Sum	2489.6956			

Signal: DAD1 B, Sig=210,4 Ref=360,100

RT [min]	Type	Width [min]	Area	Height	Area%	Name
1.086	BB	0.1108	3449.8245	486.3628	100.0000	
		Sum	3449.8245			

Signal: DAD1 C, Sig=320,4 Ref=360,100

RT [min]	Type	Width [min]	Area	Height	Area%	Name
1.086	BB	0.0939	1269.9843	206.1905	100.0000	
		Sum	1269.9843			

Signal: MSD1 TIC, MS File

RT [min]	Type	Width [min]	Area	Height	Area%	Name
1.098	BB	0.1631	380337.6563	31156.9570	100.0000	
		Sum	380337.6563			

Signal: MSD2 TIC, MS File

RT [min]	Type	Width [min]	Area	Height	Area%	Name
0.568	MM	0.0765	52737.3867	11487.0293	6.9033	
1.130	BB	0.2278	711211.2500	41426.5820	93.0967	
		Sum	763948.6367			

Agilent Technologies

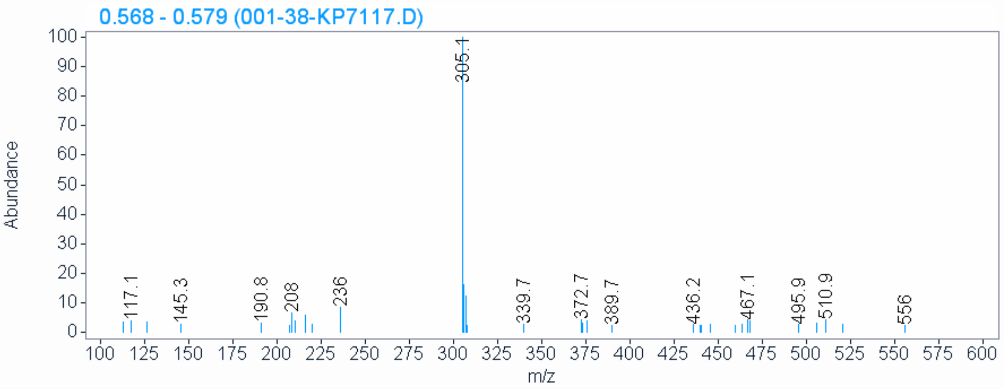

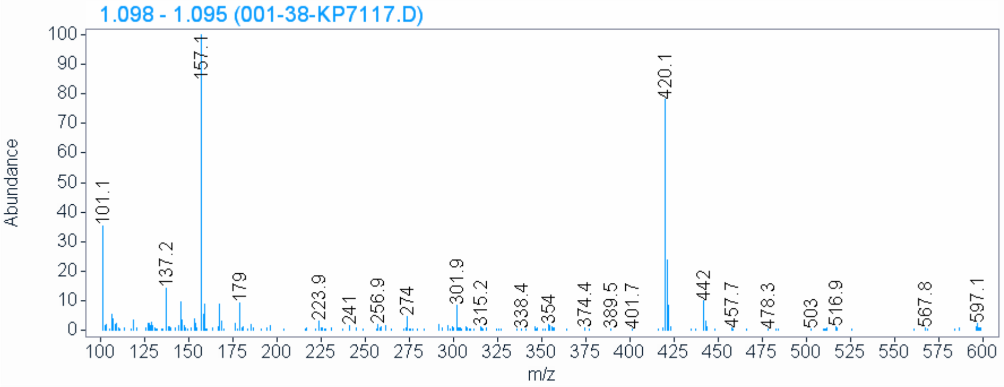

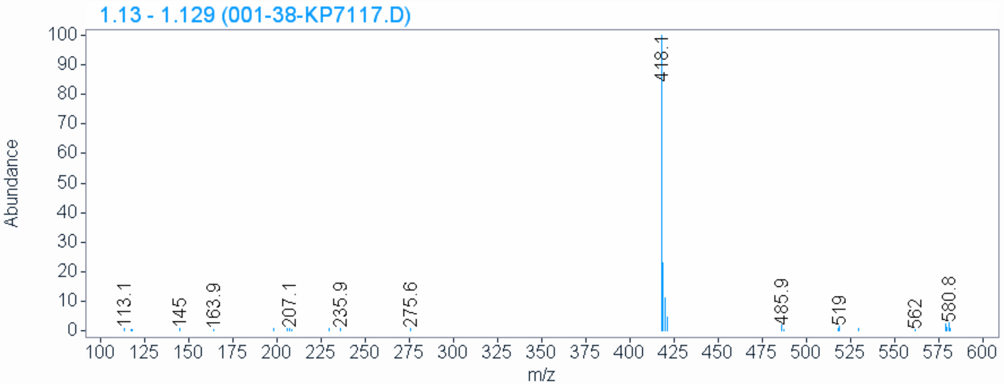

Compound Name: (Z)-*N*-(5-(4-nitrobenzylidene)-4-oxo-4,5-dihydrothiazol-2-yl)naphthalene-1-sulfonamide

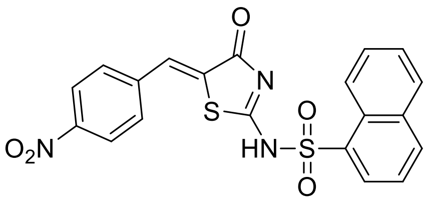

Chemical Formula: $C_{20}H_{13}N_3O_5S_2$
Exact Mass: 439.03
Molecular Weight: 439.46

Compound Code: 13 (KP6019)

Obtained Weight & Yield: 158 mg (73%)

Purity (by LCMS and ^{1}H NMR): >95% by ^{1}H NMR

Appearance: Off-white solid

Solubility: DMSO, slightly soluble in ethyl acetate, acetone, ethanol and methanol

Melting Point: > 258 °C (dec.)

TLC Rf (and conditions): 0.18 (10% MeOH in DCM)

IR Analysis (including assignment): IR (neat): ν_{max} = 3061, 2928, 2776 (C-H aromatic), 1712 (C=O), 1554 (N-H), 1516, 1506 (C-C aromatic), 1343 (N-O), 1130 (C-N) cm^{-1}

^{1}H NMR Analysis: ^{1}H NMR (400 MHz, DMSO) δ 8.62 (d, *J* = 8.6 Hz, 1H), 8.39 (d, *J* = 8.8 Hz, 2H), 8.28 (dd, *J* = 7.8, 3.3 Hz, 2H), 8.11 (d, *J* = 8.0 Hz, 1H), 7.90 (d, *J* = 8.8 Hz, 2H), 7.79 (s, 1H), 7.75 (ddd, *J* = 8.5, 7.0, 1.3 Hz, 1H), 7.71 – 7.67 (m, 2H) ppm.
Piperidine impurity at 1.55, 1.65 and 3.00ppm (3.1%). NH exchanging – not observed

^{13}C NMR Analysis: ^{13}C NMR (151 MHz, DMSO) δ 147.5, 139.6, 135.8 (br), 134.4, 133.8, 131.0 (2C), 129.5 (br), 128.9, 128.2, 128.0, 127.7, 127.1, 125.0, 124.6, 124.4 (2C) ppm.
3 quaternary carbons not visible (8h dept on 600 NMR)
2C assigned by 2D NMR

MS Analysis (low res): LRMS (ESI-) *m/z* (%): 438 (M-H, $C_{20}H_{12}N_3O_5S_2$, 100%)

HPLC method details: Column: Zorbax SB-C18 Rapid Resolution HT 2.1x50mm 1.8-Micron; Method: LCMS ISOCRATIC 60%B 0.4MLMIN-1.M filename: KP6019; Peak retention time: 1.47 mins; Area (%): 100.

Procedure: To a 10mL microwave vial was added the *N*-(4-oxo-4,5-dihydrothiazol-2-yl)naphthalene-1-sulfonamide (KP5193, 148 mg, 0.49 mmol), 4-nitrobenzaldehyde (90 mg, 0.60 mmol), the benzoic acid/piperidine catalyst mix (approximately 10 drops) and ethanol (3 mL). The suspension was heated by microwave irradiation (200 W, 120 °C) for 20 min after which a precipitate was observed. The solution was cooled in the freezer overnight and the solid collected by vacuum filtration. The solid was washed with water then cold ethanol and cold diethyl ether to give the desired product (158 mg, 73%).

Other analyses, reference papers, previously obtained data, comments, etc:

PerkinElmer Spectrum Version 10.4.00
Monday, 27 May 2019 1:35 PM

Analyst research
Date Monday, 27 May 2019 1:35 PM

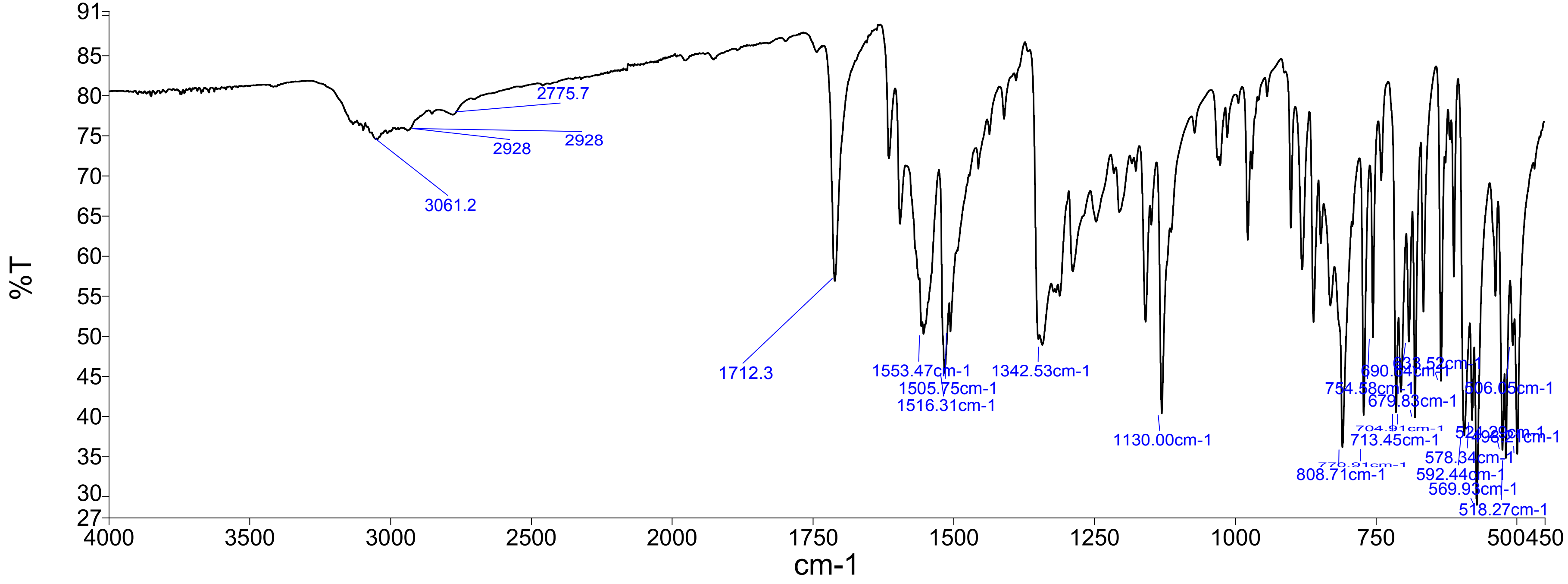

Sample Name	Description	Quality Checks
KP6019	Sample 068 By class Date Wednesday, May 08 2019	The Quality Checks do not report any warnings for the sample.

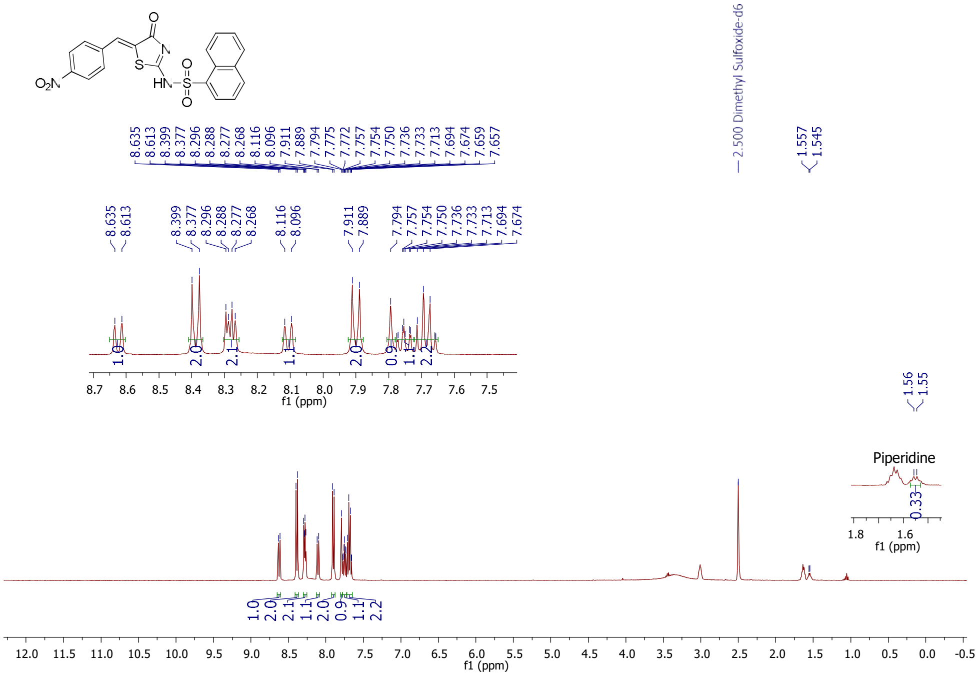

O2N
HN
S
O
N
8.635 8.613 8.399 8.377 8.296 8.288 8.277 8.268 8.116 8.096 7.911 7.889 7.794 7.775 7.772 7.757 7.754 7.750 7.736 7.733 7.713 7.694 7.674 7.659 7.657
2.500 Dimethyl Sulfoxide-d6
1.557
1.545
1.56
1.55
Piperidine
0.33
1.0 2.0 2.1 1.1 2.0 0.9 1.1 2.2
f1 (ppm)

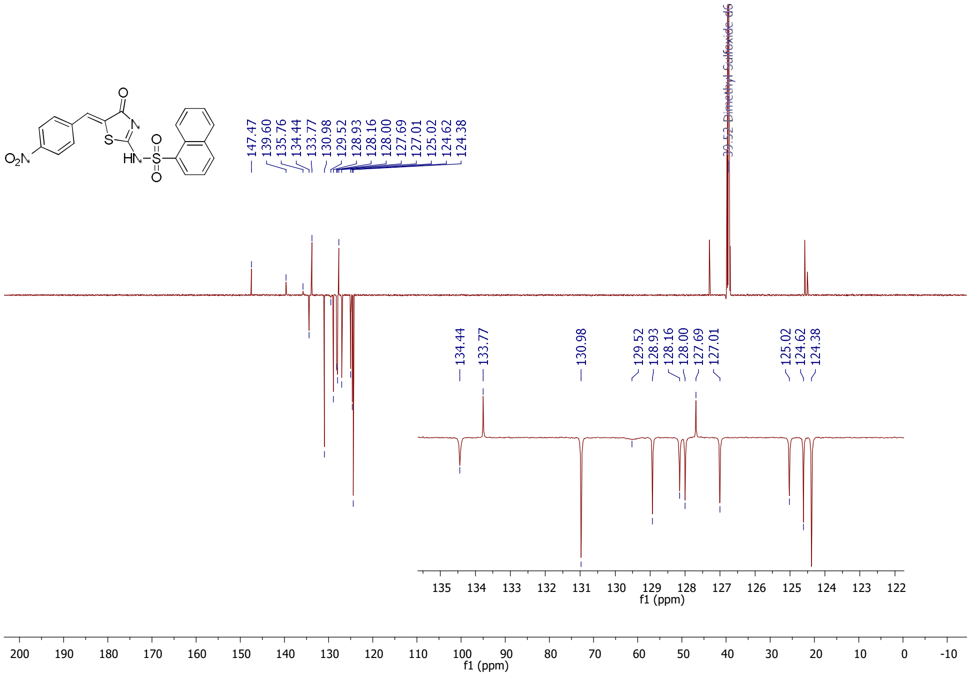

O2N
HN
S
O
N
147.47 139.60 135.76 134.44 133.77 130.98 129.52 128.93 128.16 128.00 127.69 127.01 125.02 124.62 124.38
39.52 Dimethyl Sulfoxide-d6
f1 (ppm)

LCMS Report

Data file: D:\Chem32\1\Data\KP\PRE 11-6-19\KP60514519 2019-05-24 14-00-35\004-37-KP6019.D
Sample name: KP6019
Description:
Sample amount: 0.000
Sample type: Sample

Instrument: LCMS
Location: 37
Injection date: 5/24/2019 2:25:10 PM
Injection: 1 of 1
Acq. method: LCMS ISOCRATIC 60% B 0.4MLMIN-1.M
Injection volume: 2.000
Analysis method: LCMS ISOCRATIC 60%B 0.4MLMIN-1.M
Acq. operator: SYSTEM
Last changed: 5/8/2019 8:55:04 AM

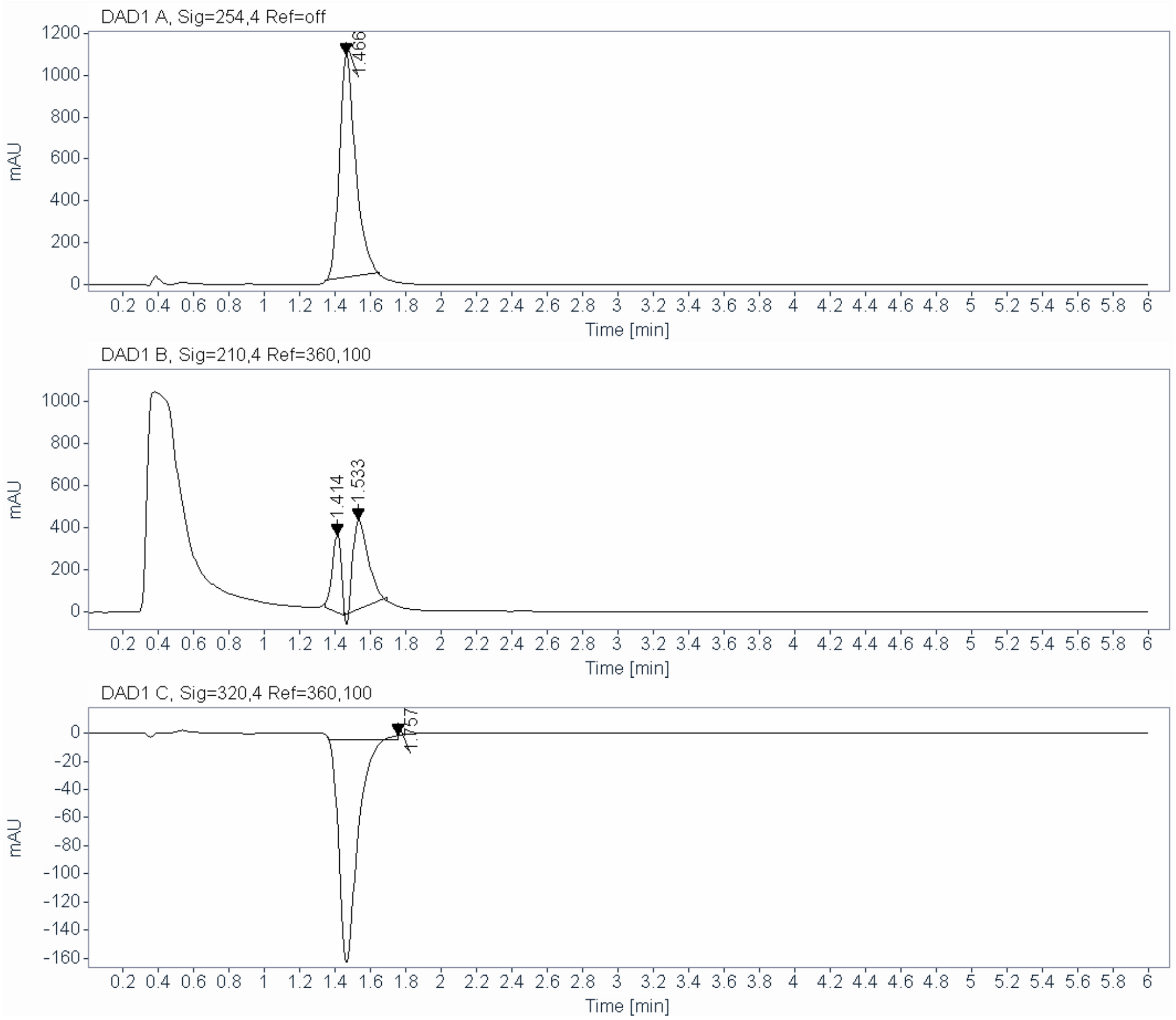

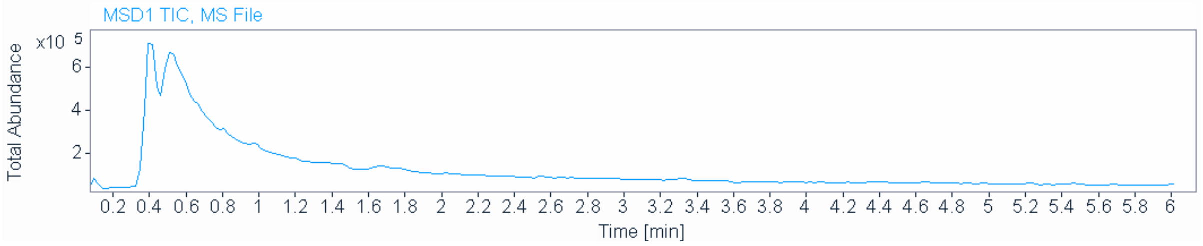

Agilent Technologies

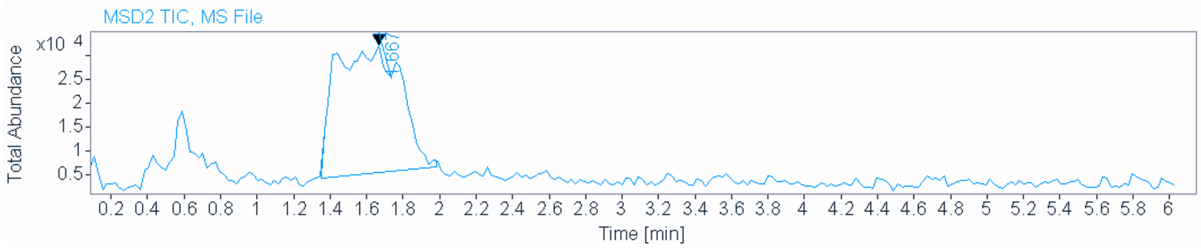

Signal: DAD1 A, Sig=254,4 Ref=off

RT [min]	Type	Width [min]	Area	Height	Area%	Name
1.466	MM	0.1062	6814.5181	1069.8840	100.0000	
		Sum	6814.5181			

Signal: DAD1 B, Sig=210,4 Ref=360,100

RT [min]	Type	Width [min]	Area	Height	Area%	Name
1.414	MM	0.0558	1241.2334	370.8192	33.5299	
1.533	MM	0.0964	2460.6416	425.3568	66.4701	
		Sum	3701.8750			

Signal: DAD1 C, Sig=320,4 Ref=360,100

RT [min]	Type	Width [min]	Area	Height	Area%	Name
1.757	MM	0.0512	9.6157	3.1296	100.0000	
		Sum	9.6157			

Signal: MSD2 TIC, MS File

RT [min]	Type	Width [min]	Area	Height	Area%	Name
1.667	MM	0.4136	656383.6875	26449.5742	100.0000	
		Sum	656383.6875			

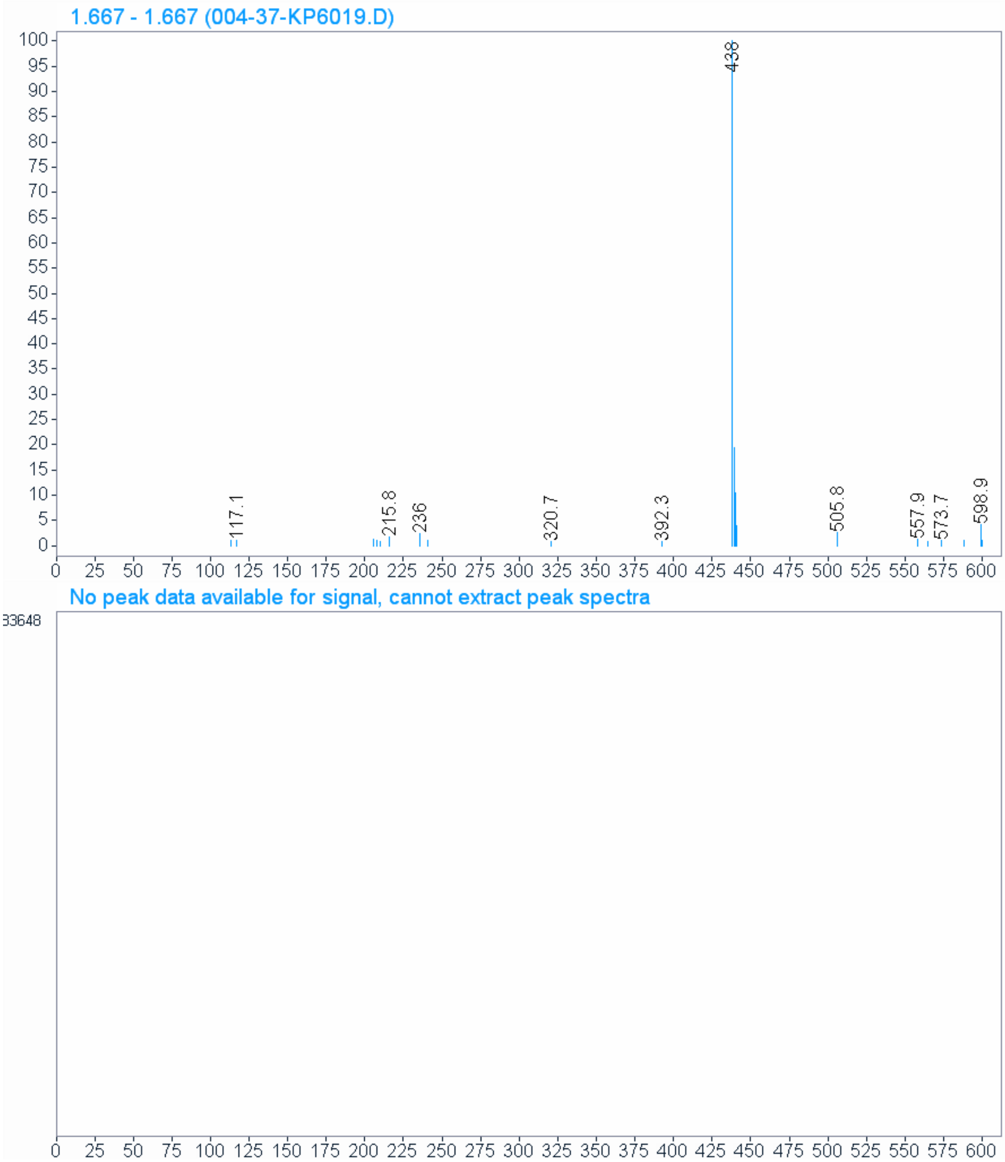
1.667 - 1.667 (004-37-KP6019.D)
100
95
90
85
80
75
70
65
60
55
50
45
40
35
30
25
20
15
10
5
0
438
117.1
215.8
236
320.7
392.3
505.8
557.9
573.7
598.9
0 25 50 75 100 125 150 175 200 225 250 275 300 325 350 375 400 425 450 475 500 525 550 575 600
No peak data available for signal, cannot extract peak spectra
33648
0 25 50 75 100 125 150 175 200 225 250 275 300 325 350 375 400 425 450 475 500 525 550 575 600

Compound Name: (Z)-*N*-(5-(3-nitrobenzylidene)-4-oxo-4,5-dihydrothiazol-2-yl)naphthalene-1-sulfonamide

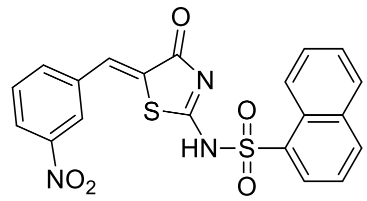

Chemical Formula: $C_{20}H_{13}N_3O_5S_2$
Exact Mass: 439.03
Molecular Weight: 439.46

Compound Code: 14 (KP6020)

Obtained Weight & Yield: 87 mg (40%)

Purity (by LCMS and ^{1}H NMR): 98% by ^{1}H-NMR

Appearance: Off-white solid

Solubility: DMSO, slightly soluble in acetone, ethanol and methanol

Melting Point: > 243 °C (dec.)

TLC Rf (and conditions): 0.21 (10% MeOH in DCM)

IR Analysis (including assignment): IR (neat): ν_{max} = 2979, 2763 (C-H aromatic), 1728 (C=O), 1569 (N-H), 1528 (C-C aromatic), 1354 (N-O), 1126 (C-N) cm^{-1}

^{1}H NMR Analysis: ^{1}H NMR (400 MHz, DMSO) δ 8.61 (d, *J* = 8.6 Hz, 1H), 8.53 (s, 1H), 8.31 – 8.29 (m, 3H), 8.11 (dd, *J* = 14.3, 7.9 Hz, 2H), 7.93 (s, 1H), 7.89 (t, *J* = 8.0 Hz, 1H), 7.77 (t, *J* = 7.2 Hz, 1H), 7.73 – 7.68 (m, 2H) ppm.
NH exchanging – not observed.
Starting material at 4.04 ppm (2.05%)

^{13}C NMR Analysis: ^{13}C NMR (151 MHz, DMSO) δ 166.4, 165.1, 148.3, 135.5, 135.1, 134.9, 134.6, 133.8, 131.2, 131.1, 129.0, 128.4, 128.2, 127.6, 127.1, 124.9 (br), 124.8 (2C), 124.7, 124.7 ppm.
2C assigned by 2D NMR

MS Analysis (low res): LRMS (ESI+) *m/z*: 440 (M+H, $C_{20}H_{14}N_3O_5S_2$, 100%); (ESI-) *m/z*: 438 (M-H, $C_{20}H_{12}N_3O_5S_2$, 100%)

HPLC method details: Column: Zorbax SB-C18 Rapid Resolution HT 2.1x50mm 1.8-Micron; Method: LCMS ISOCRATIC 50% B.M_REDUCED FLOW.M filename: KP6020; Peak retention time: 3.20 mins; Area (%): 95.

Procedure: To a 10mL microwave vial was added the N-(4-oxo-4,5-dihydrothiazol-2-yl)naphthalene-1-sulfonamide (KP5193, 148 mg, 0.49 mmol), 3-nitrobenzaldehyde (86 mg, 0.55 mmol, 1.1 eq), the benzoic acid/piperidine catalyst (approximately 10 drops) and ethanol (3 mL). The suspension was heated by microwave irradiation (200 W, 120 °C) for 30 min after which hexane was added until a precipitate formed and the solution was placed in the freezer. The filtrate was collected by vacuum filtration and the solid washed with water, cold ethanol and cold hexane to give the desired product (87 mg, 40%).

PerkinElmer Spectrum Version 10.4.00
Monday, 27 May 2019 1:35 PM

Analyst	research
Date	Monday, 27 May 2019 1:35 PM

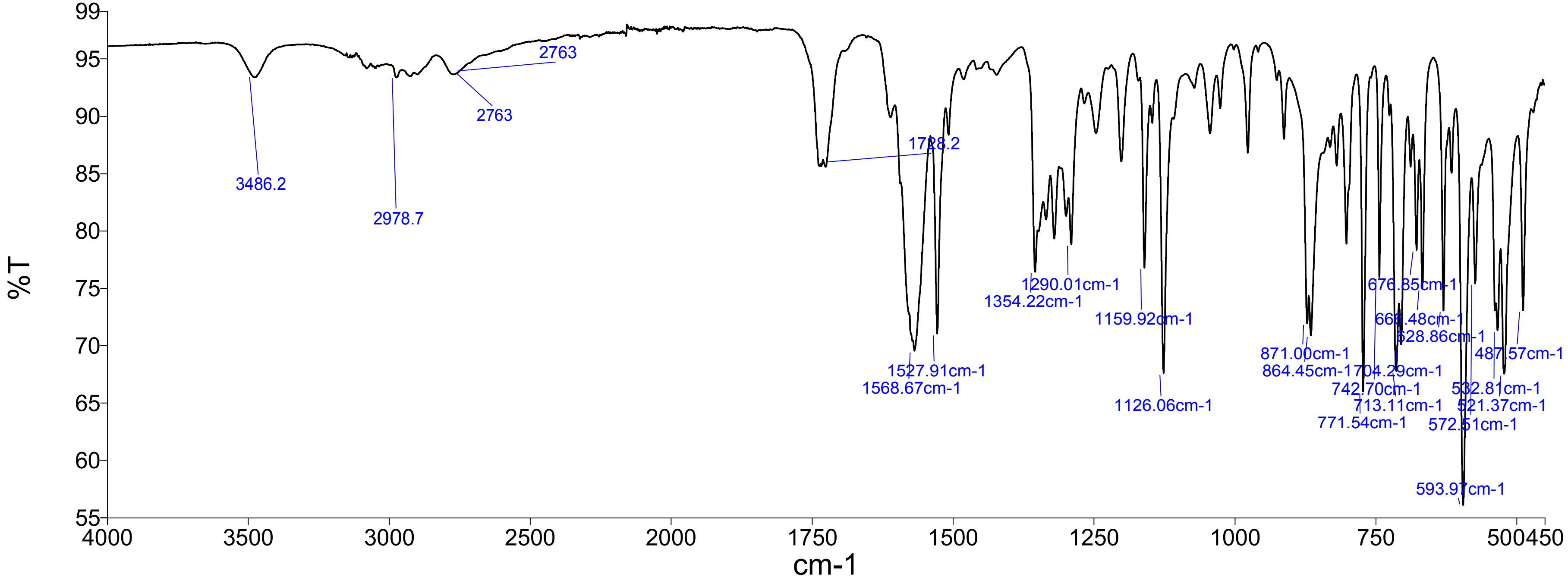

Sample Name	Description	Quality Checks
KP6020	Sample 067 By class Date Wednesday, May 08 2019	The Quality Checks do not report any warnings for the sample.

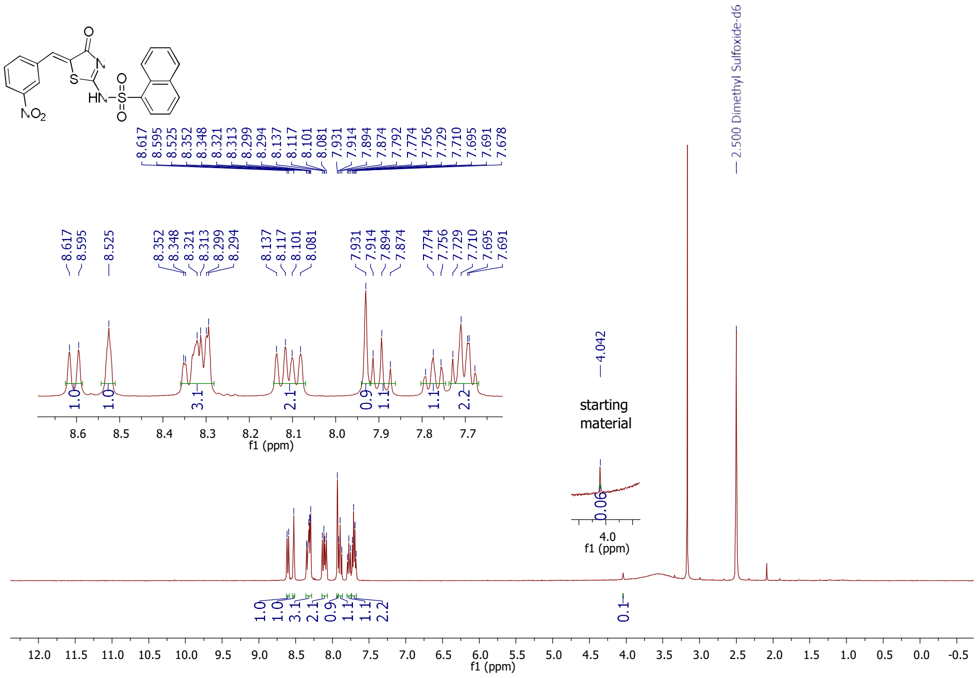
8.617 8.595 8.525 8.352 8.348 8.321 8.313 8.299 8.294 8.137 8.117 8.101 8.081 7.931 7.914 7.894 7.874 7.792 7.774 7.756 7.729 7.710 7.695 7.691 7.678
2.500 Dimethyl Sulfoxide-d6
4.042
starting material
f1 (ppm)

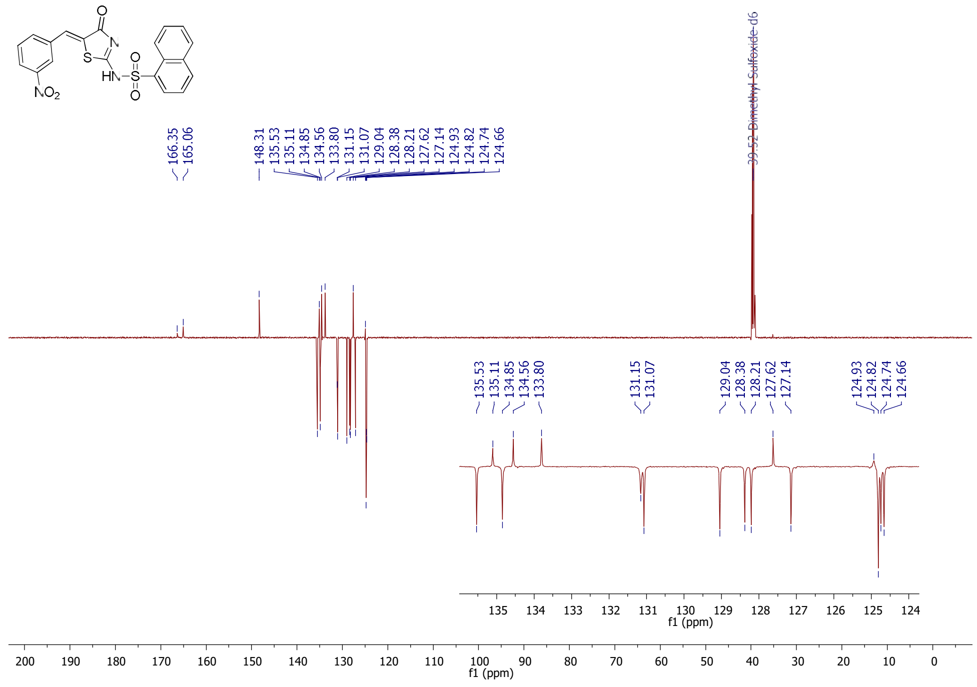
166.35 165.06 148.31 135.53 135.11 134.85 134.56 133.80 131.15 131.07 129.04 128.38 128.21 127.62 127.14 124.93 124.82 124.74 124.66
39.52 Dimethyl Sulfoxide-d6
f1 (ppm)

LCMS Report

Data file: D:\Chem32\1\Data\KP\PRE 11-6-19\KP6019020 2019-05-10 13-34-28\003-77-KP6020.D
Sample name: KP6020
Description:
Sample amount: 0.000
Sample type: Sample

Instrument: LCMS
Location: 77
Injection date: 5/10/2019 1:49:27 PM
Injection: 1 of 1
Acq. method: LCMS ISOCRATIC 50% B.M_REDUCED FLOW.M
Injection volume: 2.000
Analysis method: LCMS ISOCRATIC 50% B.M_REDUCED FLOW.M
Acq. operator: SYSTEM
Last changed: 10/21/2016 12:01:19 PM

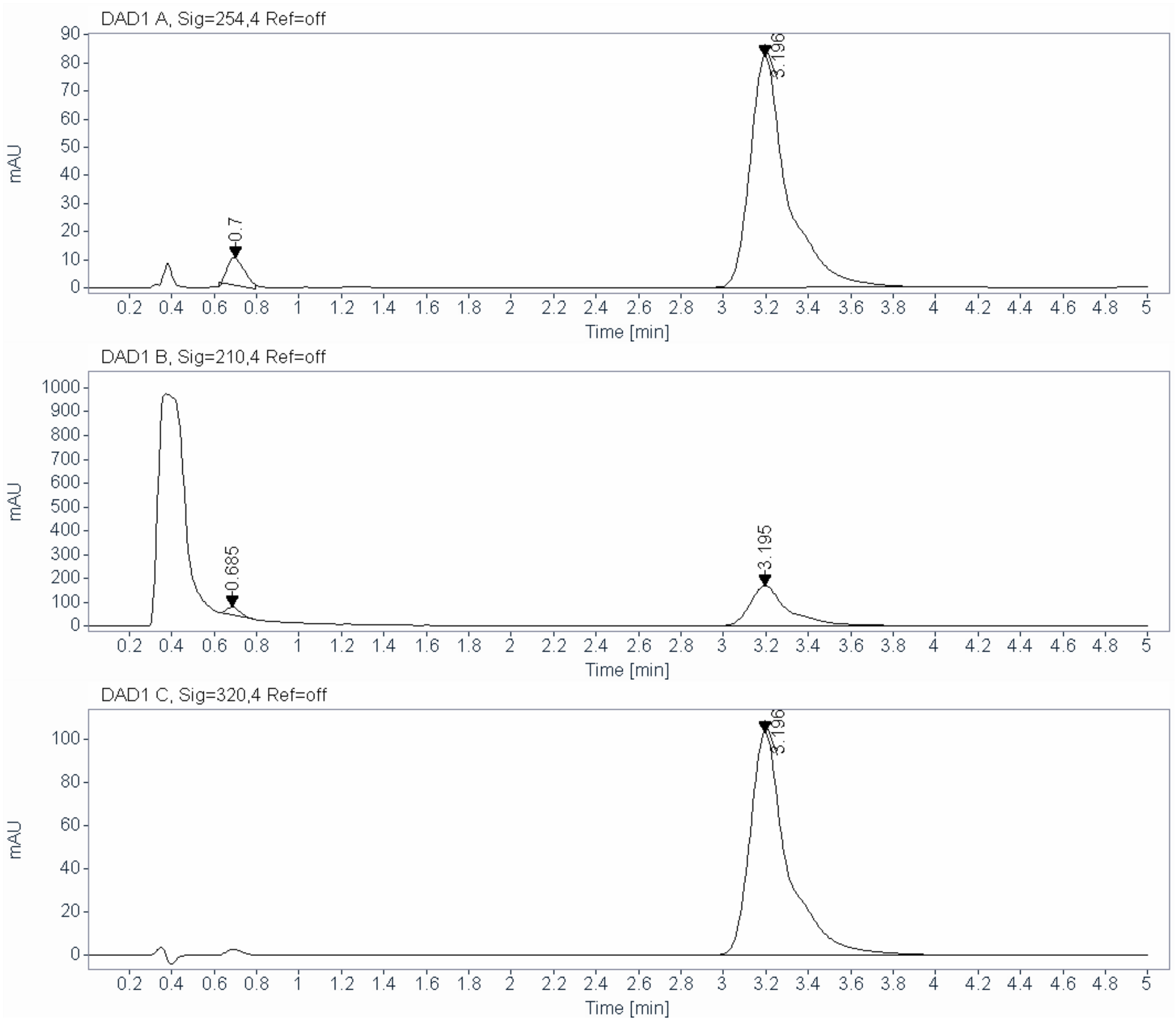

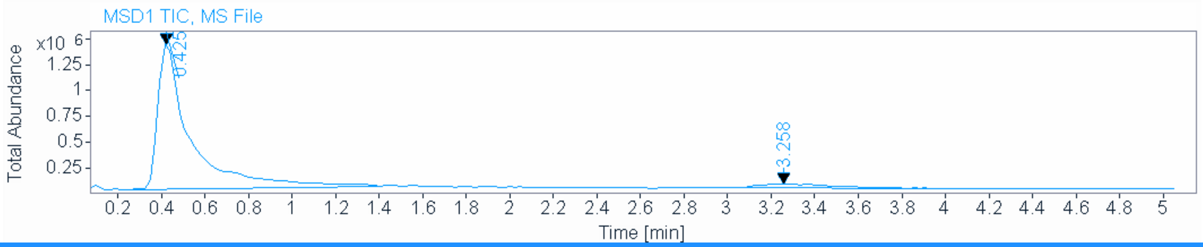

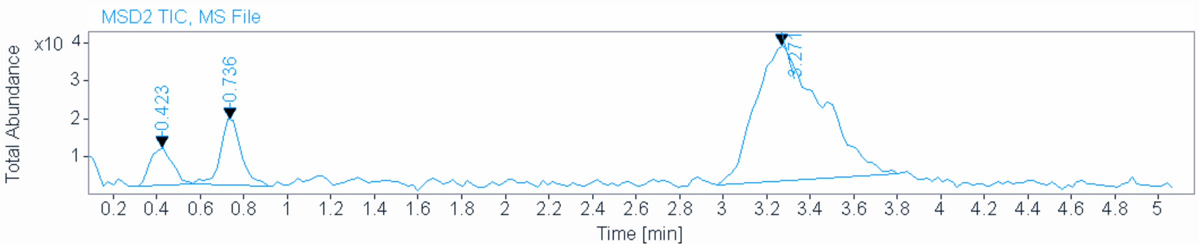

Signal: DAD1 A, Sig=254,4 Ref=off

RT [min]	Type	Width [min]	Area	Height	Area%	Name
0.700	MM	0.0954	55.4864	9.6895	5.2273	
3.196	BB	0.1767	1005.9813	82.2240	94.7727	
		Sum	1061.4678			

Signal: DAD1 B, Sig=210,4 Ref=off

RT [min]	Type	Width [min]	Area	Height	Area%	Name
0.685	BB	0.0730	147.5393	32.5043	6.5771	
3.195	BB	0.1809	2095.7002	168.7423	93.4229	
		Sum	2243.2395			

Signal: DAD1 C, Sig=320,4 Ref=off

RT [min]	Type	Width [min]	Area	Height	Area%	Name
3.196	BB	0.1776	1267.7131	102.9609	100.0000	
		Sum	1267.7131			

Signal: MSD1 TIC, MS File

RT [min]	Type	Width [min]	Area	Height	Area%	Name
0.425	BB	0.1407	14657047.0000	1423874.8750	93.5511	
3.258	BB	0.3141	1010371.9375	41132.8477	6.4489	
		Sum	15667418.93			

Signal: MSD2 TIC, MS File

RT [min]	Type	Width [min]	Area	Height	Area%	Name
0.423	BB	0.1098	70835.7734	10048.2285	7.5292	
0.736	BB	0.1033	117235.8203	18038.7480	12.4610	
3.271	BB	0.2660	752747.8125	35795.6992	80.0098	
		Sum	940819.4063			

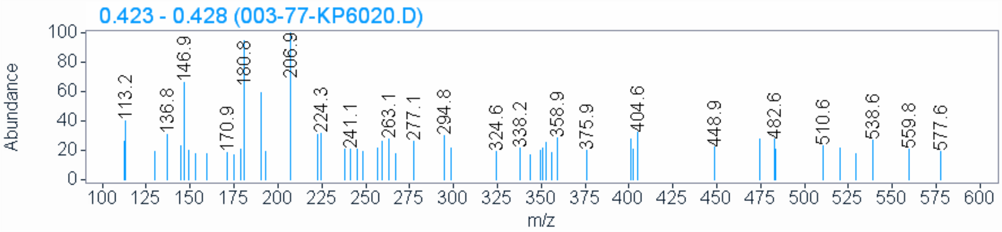
0.423 - 0.428 (003-77-KP6020.D)
Abundance
m/z
113.2
136.8
146.9
170.9
180.8
206.9
224.3
241.1
263.1
277.1
294.8
324.6
338.2
358.9
375.9
404.6
448.9
482.6
510.6
538.6
559.8
577.6

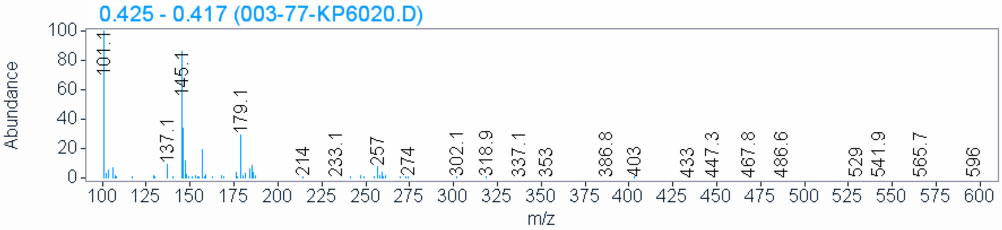
0.425 - 0.417 (003-77-KP6020.D)
Abundance
m/z
101.1
137.1
145.1
179.1
214
233.1
257
274
302.1
318.9
337.1
353
386.8
403
433
447.3
467.8
486.6
529
541.9
565.7
596

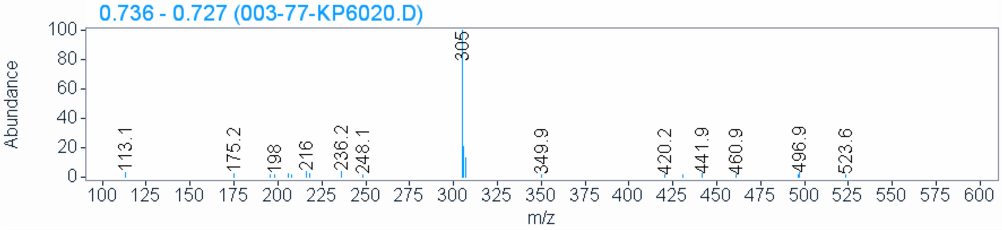
0.736 - 0.727 (003-77-KP6020.D)
Abundance
m/z
113.1
175.2
198
216
236.2
248.1
305
349.9
420.2
441.9
460.9
496.9
523.6

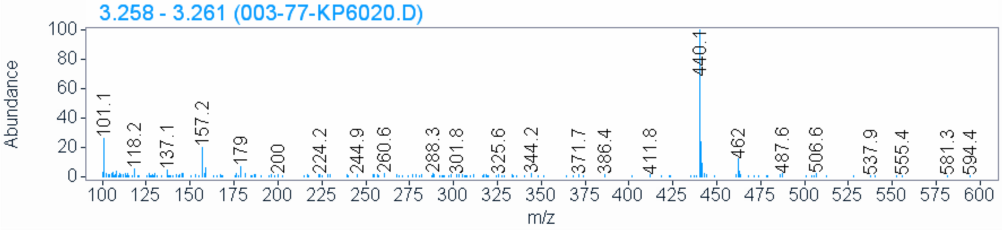
3.258 - 3.261 (003-77-KP6020.D)
Abundance
m/z
101.1
118.2
137.1
157.2
179
200
224.2
244.9
260.6
288.3
301.8
325.6
344.2
371.7
386.4
411.8
440.1
462
487.6
506.6
537.9
555.4
581.3
594.4

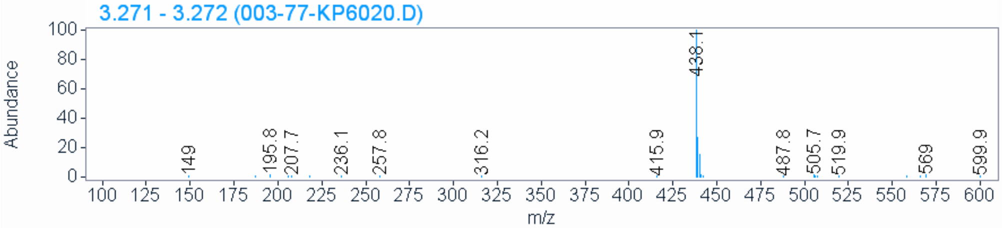
3.271 - 3.272 (003-77-KP6020.D)
Abundance
m/z
149
195.8
207.7
236.1
257.8
316.2
415.9
438.1
487.8
505.7
519.9
569
599.9

Compound Name: (*Z*)-*N*-(5-(2-nitrobenzylidene)-4-oxo-4,5-dihydrothiazol-2-yl)naphthalene-1-sulfonamide

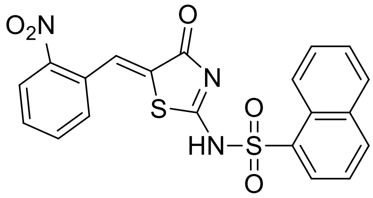

Chemical Formula: $C_{20}H_{13}N_3O_5S_2$
Exact Mass: 439.03
Molecular Weight: 439.46

Compound Code: 15 (KP6013)

Obtained Weight & Yield: 100 mg (48%)

Purity (by LCMS and ^{1}H NMR): > 99% by ^{1}H-NMR

Appearance: pale pink solid

Solubility: DMSO, slightly soluble in ethyl acetate, acetone, ethanol and methanol

Melting Point: > 245 °C (dec.)

TLC Rf (and conditions): 0.26 (10% MeOH in DCM)

IR Analysis (including assignment): IR (neat): ν_{max} = 3055, 2941, 2788 (Aromatic C-H), 1714 (C=O), 1558 (N-H), 1517 (C=C aromatic), 1340 (N-O), 1159, 1131 (C-N) cm^{-1}

^{1}H NMR Analysis: ^{1}H NMR (400 MHz, DMSO) δ 8.58 (d, *J* = 8.5 Hz, 1H), 8.29 (d, *J* = 8.2 Hz, 1H), 8.24 (dd, *J* = 7.7, 2.6 Hz, 2H), 8.12 (d, *J* = 8.0 Hz, 1H), 8.02 (s, 1H), 7.98 (t, *J* = 7.5 Hz, 1H), 7.78 (dd, *J* = 15.1, 7.9 Hz, 3H), 7.71 – 7.66 (m, 2H) ppm.
NH exchanging – not observed

^{13}C NMR Analysis: ^{13}C NMR (101 MHz, DMSO) δ 165.9, 165.6, 147.8, 135.1, 134.8 (2C), 133.8, 131.3, 130.5, 129.6, 129.0, 128.9, 128.3, 128.1, 127.6, 127.1, 126.5, 125.6, 124.8, 124.6 ppm.
2C assigned by 2D NMR

MS Analysis (low res): LRMS (ESI+) *m/z*: 440 (M+H, $C_{20}H_{14}N_3O_5S_2$, 100%); (ESI+) *m/z*: 438 (M+H, $C_{20}H_{12}N_3O_5S_2$, 100%)

HPLC method details: Column: Zorbax SB-C18 Rapid Resolution HT 2.1x50mm 1.8-Micron; Method: LCMS ISOCRATIC 50% B.M_REDUCED FLOW.M filename: KP6013; Peak retention time: 2.70 mins; Area (%): 97.

Procedure: To a 10mL microwave vial was added the *N*-(4-oxo-4,5-dihydrothiazol-2-yl)naphthalene-1-sulfonamide (KP5139, 146 mg, 0.48 mmol), 2-nitrobenzaldehyde (83 mg, 0.54 mmol, 1.1 eq), the benzoic acid catalyst (approximately 10 drops) and ethanol (3 mL). The suspension was heated by microwave irradiation (200 W, 120 °C) for 30 min. Hexane was added until a precipitate formed then the solution was placed in the freezer overnight. The precipitate was collected by vacuum filtration was washed with Water, cold ethanol and cold hexane to give the desired product (100 mg, 48%).

Other analyses, reference papers, previously obtained data, comments, etc:

PerkinElmer Spectrum Version 10.4.00
Monday, 27 May 2019 1:34 PM

Analyst research
Date Monday, 27 May 2019 1:34 PM

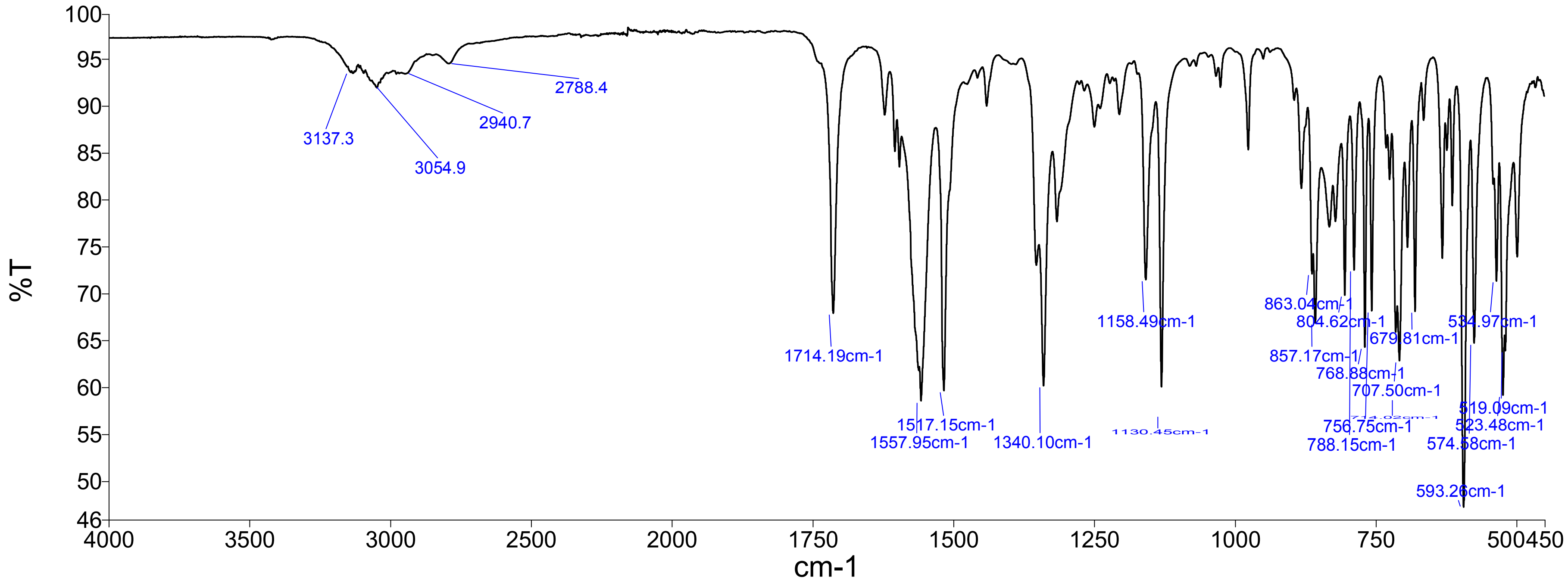

Sample Name	Description	Quality Checks
KP6013	Sample 066 By class Date Wednesday, May 08 2019	The Quality Checks do not report any warnings for the sample.

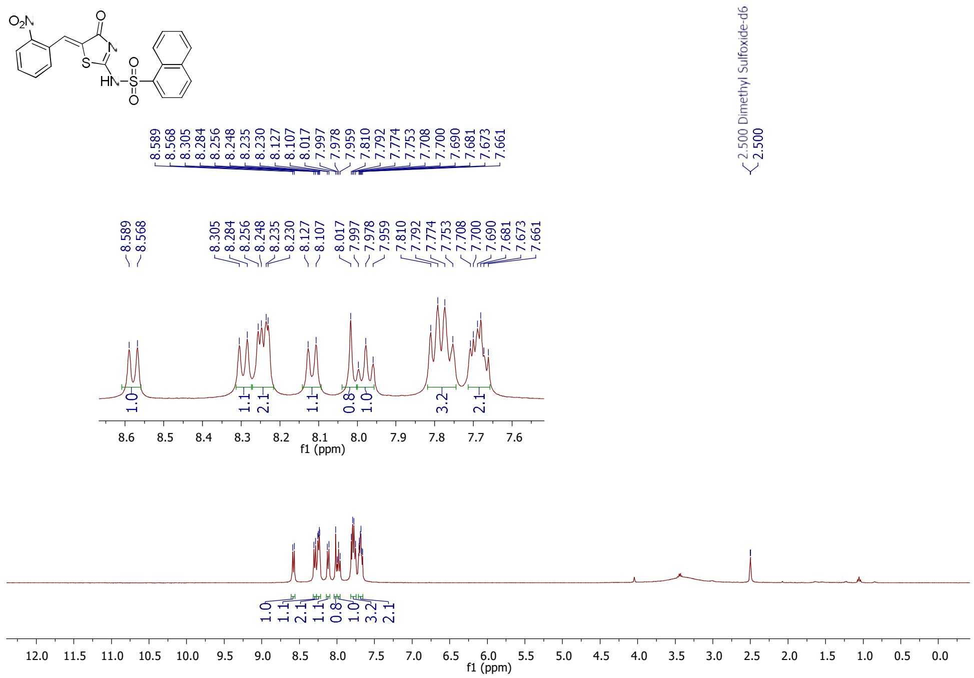
2.500 Dimethyl Sulfoxide-d6
8.589
8.568
8.305
8.284
8.256
8.248
8.235
8.230
8.127
8.107
8.017
7.997
7.978
7.959
7.810
7.792
7.774
7.753
7.708
7.700
7.690
7.681
7.673
7.661
2.500
1.0
1.1
2.1
1.1
0.8
1.0
3.2
2.1
f1 (ppm)

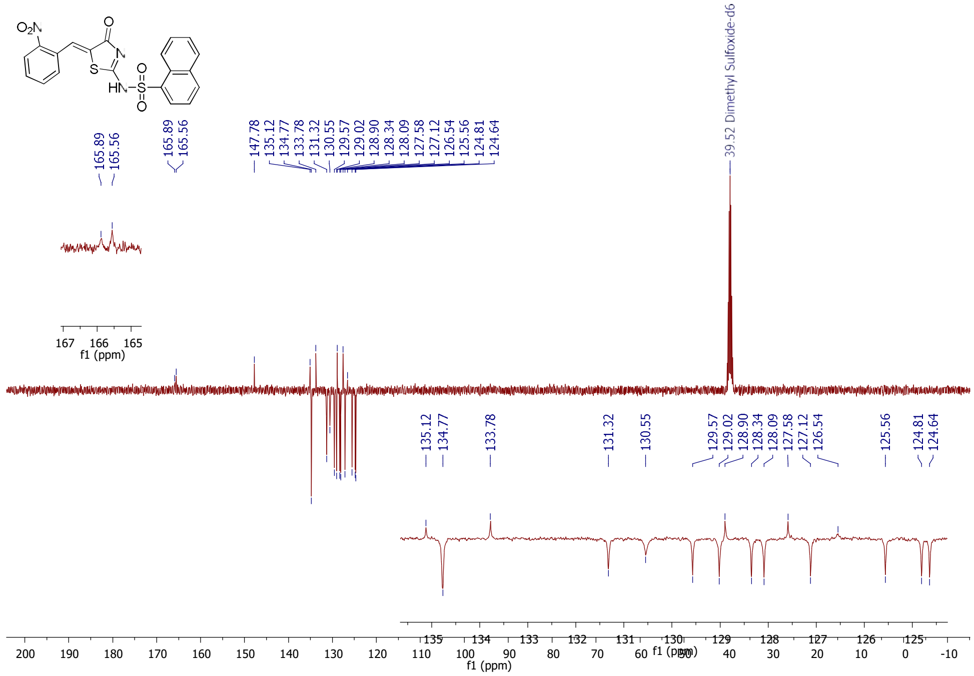
39.52 Dimethyl Sulfoxide-d6
165.89
165.56
147.78
135.12
134.77
133.78
131.32
130.55
129.57
129.02
128.90
128.34
128.09
127.58
127.12
126.54
125.56
124.81
124.64
f1 (ppm)

LCMS Report

Agilent Technologies

**Data file:**	D:\Chem32\1\Data\KP\PRE 11-6-19\KP5189-6020 2019-05-10 13-00-54\003-74-KP6013.D		
**Sample name:**	KP6013		
**Description:**			
**Sample amount:**	0.000	**Sample type:**	Sample
**Instrument:**	LCMS	**Location:**	74
**Injection date:**	5/10/2019 1:15:54 PM	**Injection:**	1 of 1
**Acq. method:**	LCMS ISOCRATIC 50% B.M_REDUCED FLOW.M	**Injection volume:**	2.000
**Analysis method:**	LCMS ISOCRATIC 50% B.M_REDUCED FLOW.M	**Acq. operator:**	SYSTEM
**Last changed:**	10/21/2016 12:01:19 PM		

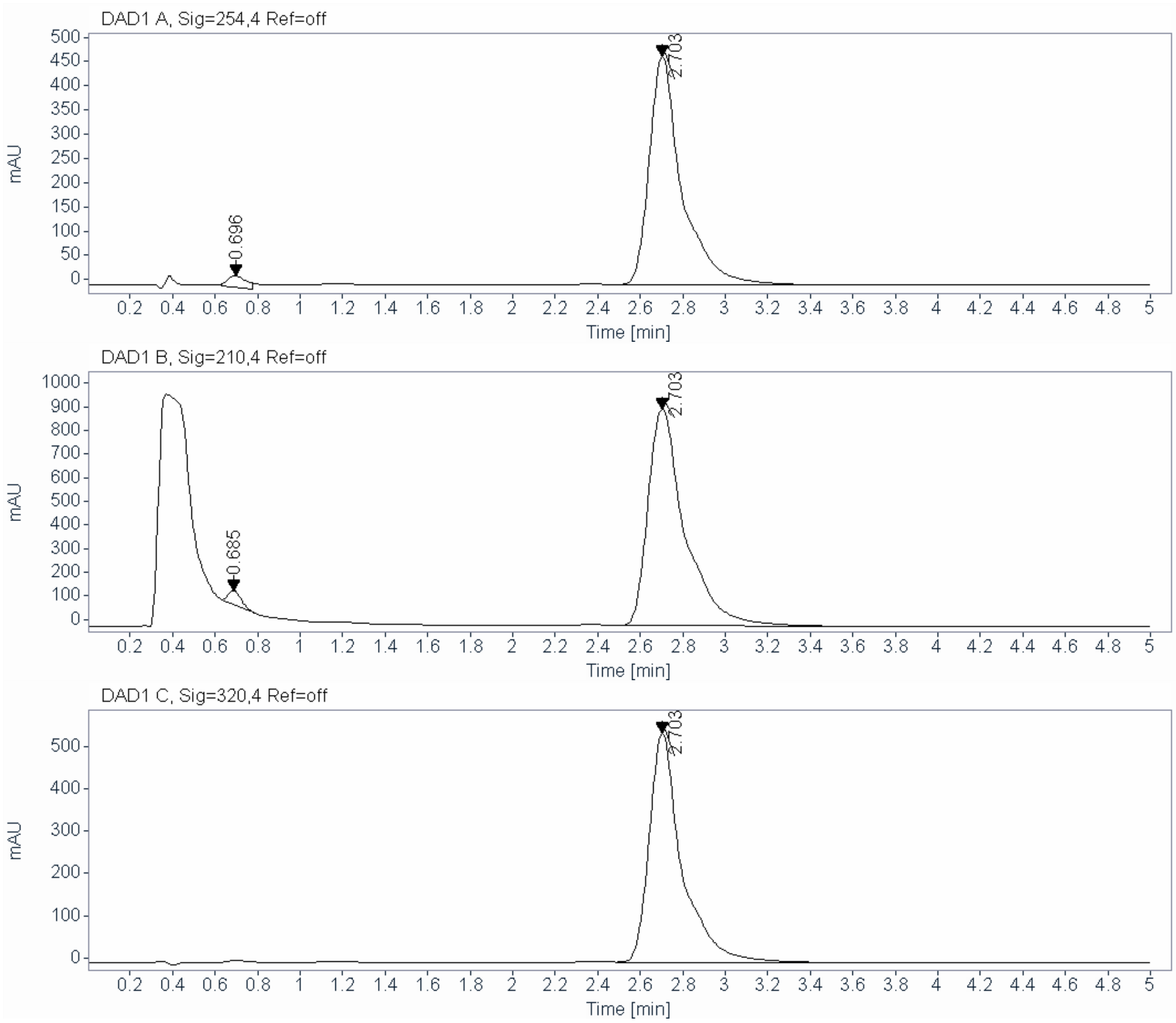

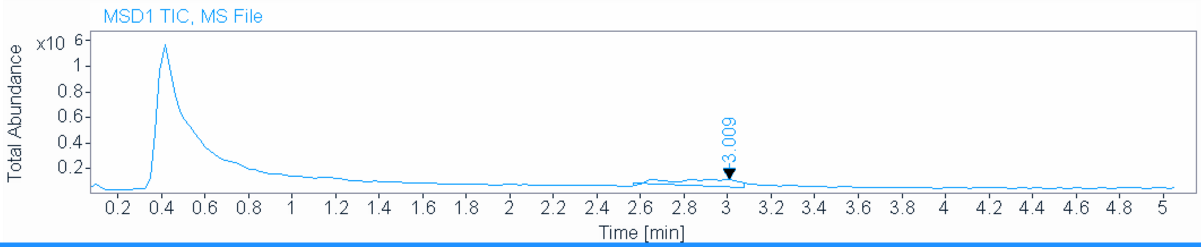

Agilent Technologies

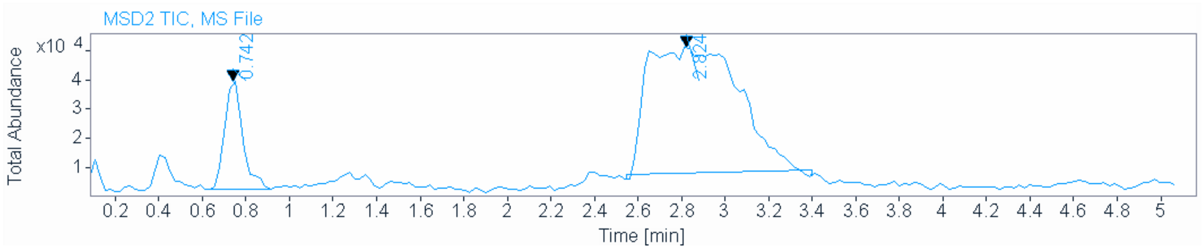

Signal: DAD1 A, Sig=254,4 Ref=off

RT [min]	Type	Width [min]	Area	Height	Area%	Name
0.696	MM	0.1019	150.6070	24.6252	2.9199	
2.703	BB	0.1559	5007.3140	471.6864	97.0801	
		Sum	5157.9210			

Signal: DAD1 B, Sig=210,4 Ref=off

RT [min]	Type	Width [min]	Area	Height	Area%	Name
0.685	BB	0.0703	247.0997	57.2864	2.1945	
2.703	BB	0.1780	11012.6182	917.5587	97.8055	
		Sum	11259.7179			

Signal: DAD1 C, Sig=320,4 Ref=off

RT [min]	Type	Width [min]	Area	Height	Area%	Name
2.703	BB	0.1559	5761.2080	542.4388	100.0000	
		Sum	5761.2080			

Signal: MSD1 TIC, MS File

RT [min]	Type	Width [min]	Area	Height	Area%	Name
3.009	MM	0.3343	1117678.375<br>0	55726.5820	100.0000	
		Sum	1117678.375			

Signal: MSD2 TIC, MS File

RT [min]	Type	Width [min]	Area	Height	Area%	Name
0.742	BB	0.0945	217466.7188	37855.1367	14.6089	
2.824	MM	0.4849	1271127.750<br>0	43690.5156	85.3911	
		Sum	1488594.468			

Agilent Technologies

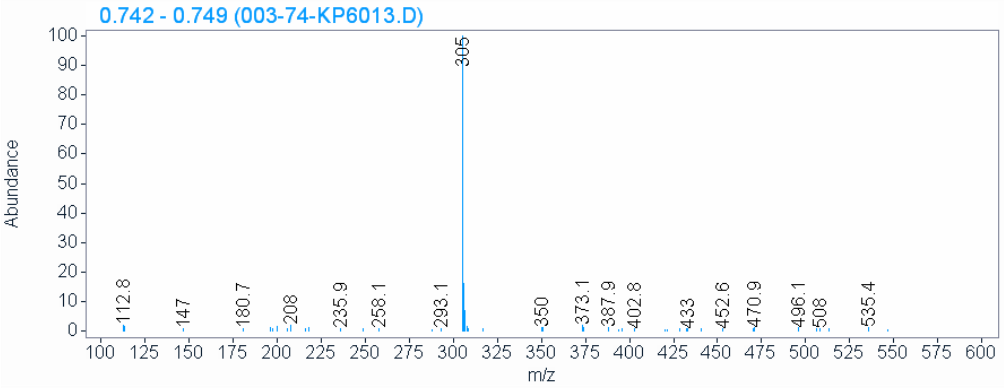

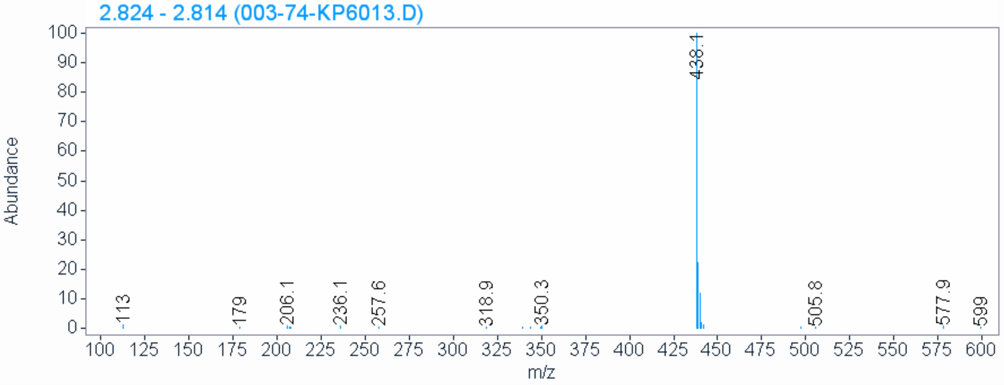

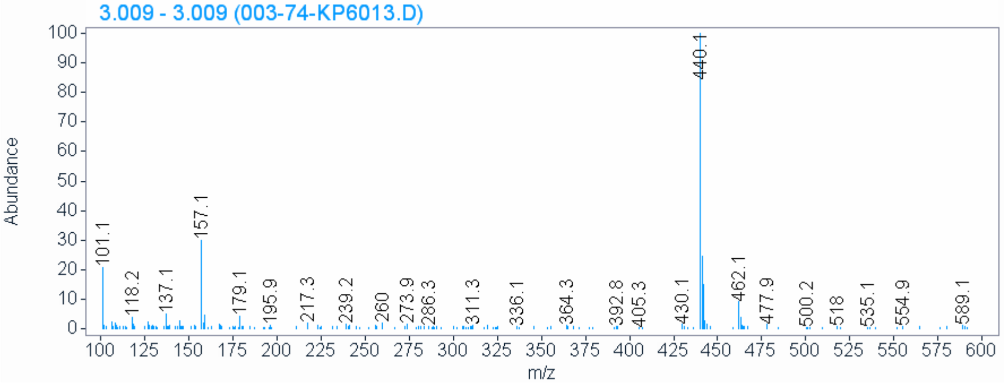

Name of Researcher: Kate Prichard

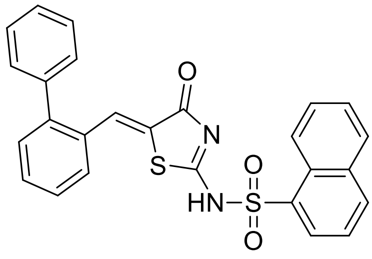

Chemical Formula: $C_{26}H_{18}N_2O_3S_2$
Exact Mass: 470.08
Molecular Weight: 470.56

Compound Name: (*Z*)-*N*-(5-([1,1'-biphenyl]-2-ylmethylene)-4-oxo-4,5-dihydrothiazol-2-yl)naphthalene-1-sulfonamide

Code: 16 (KP9192)

Obtained Weight & Yield: 113 mg (49%)

Purity (by LCMS and ^{1}H NMR): > 95% by ^{1}H-NMR.

Appearance: off white solid

Solubility: DMSO, slightly soluble in acetone and methanol.

Melting Point: 202 – 204 °C

TLC Rf (and conditions): N/A

IR Analysis (including assignment): IR (neat): ν_{max} = 3455 (N-H), 2980 (C-H aromatic), 1711 (C=O), 1570 (C-C aromatic), 1316 (sulfomamide), 1127 (C-N) cm^{-1}

^{1}H NMR Analysis: ^{1}H NMR (400 MHz, DMSO) δ 8.61 (d, *J* = 8.4 Hz, 1H), 8.29 (s, 2H), 8.12 (d, *J* = 7.8 Hz, 1H), 7.79 – 7.60 (m, 6H), 7.52 – 7.45 (m, 5H), 7.35 (d, *J* = 6.3 Hz, 2H) ppm.
NH exchanging – not visible
Ethanol impurity at 1.06 ppm (4.84%)

^{13}C NMR Analysis: ^{13}C NMR (101 MHz, DMSO) δ 166.2, 165.9, 143.3, 139.0, 135.2, 134.7, 133.8, 132.6, 130.8, 130.7 (2C), 129.7 (2C), 129.0, 128.5 (2C), 128.34, 128.31, 128.1, 128.0, 127.95, 127.6, 127.1, 124.9, 124.7, 123.4 ppm.
2C determined by 2D NMR

MS Analysis (low res): LRMS (ESI-): 469 (*M*-H, $C_{26}H_{17}N_2O_3S_2$, 100)

HPLC method details: Column: Zorbax SB-C18 Rapid Resolution HT 2.1x50mm 1.8-Micron; Method: LCMS ISOCRATIC 80%B_3 MINS.M filename: KP9192; Peak retention time: 0.963 mins; Area (%): 100.

Procedure: To a 10 mL microwave vial was added *N*-(4-oxo-4,5-dihydrothiazol-2-yl)naphthalene-1-sulfonamide (150 mg, 0.49 mmol), 2-biphenylcarboxaldehyde (0.1 mL, 0.54 mmol, 1.1 eq), ethanol (3 mL) and a catalytic amount of the benzoic acid/piperidine catalyst (approximately 5 drops). The suspension was heated by microwave irradiation (120 °C, 200 W) for 45 min. A precipitate formed upon cooling. The solid was collected by vacuum filtration to give the desired product as an off white solid (113 mg, 49%).

Other analyses, reference papers, previously obtained data, comments, etc:

PerkinElmer Spectrum Version 10.4.00
Monday, 10 May 2021 11:07 AM

Analyst research
Date Monday, 10 May 2021 11:07 AM

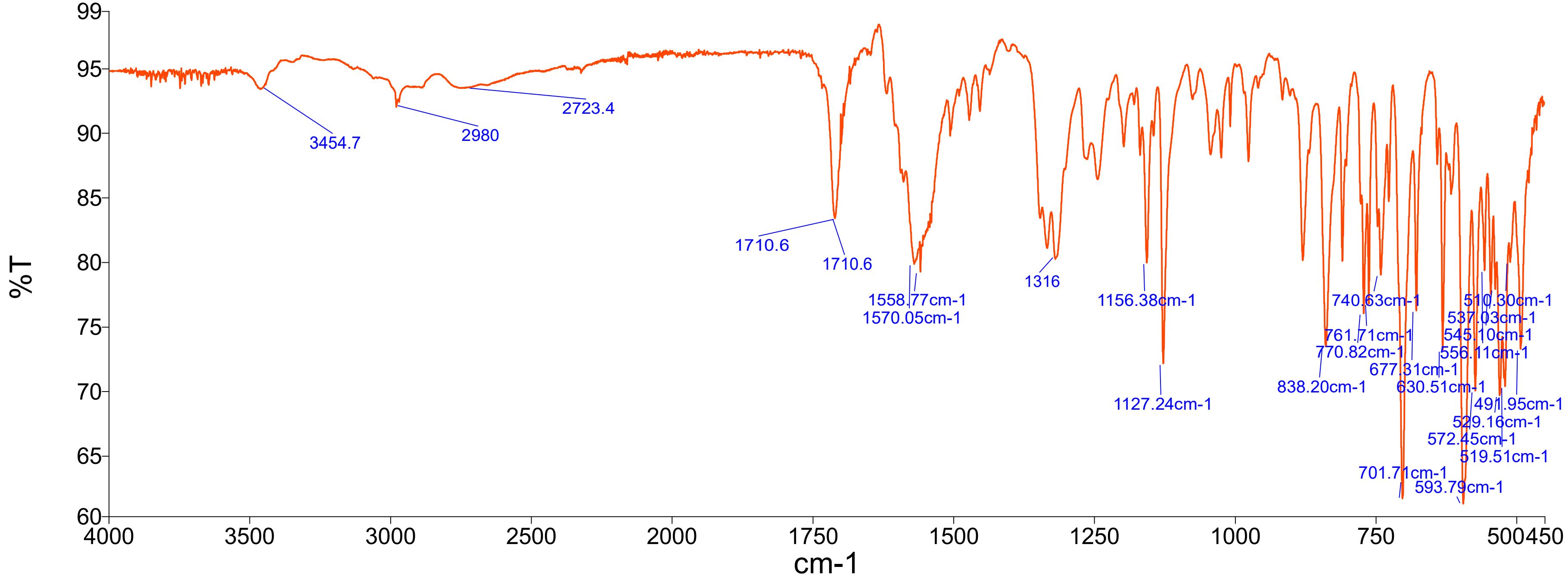

Sample Name	Description	Quality Checks
kp9192	Sample 009 By research Date Monday, May 10 2021	The Quality Checks do not report any warnings for the sample.

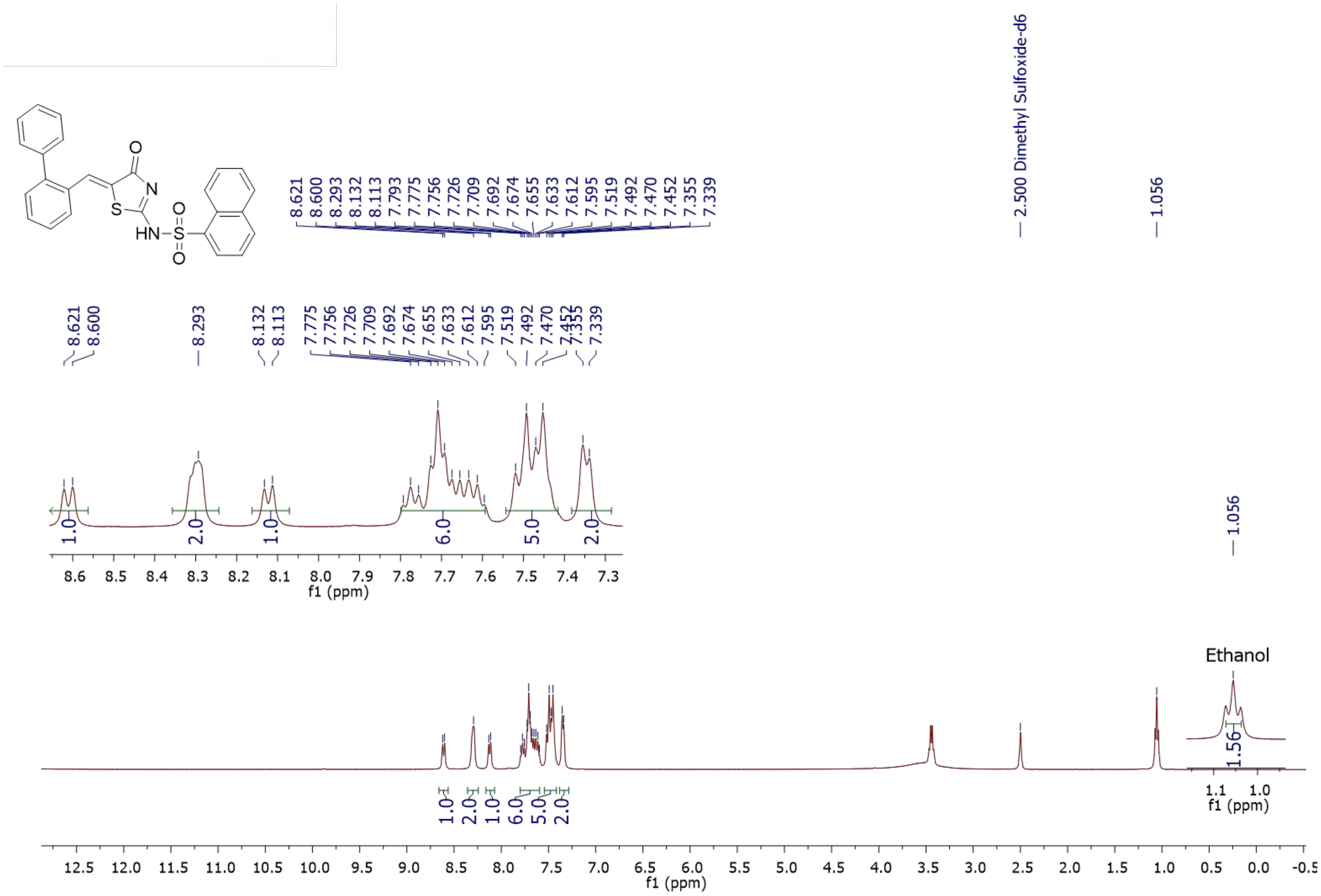

8.621 8.600 8.293 8.132 8.113 7.793 7.775 7.756 7.726 7.709 7.692 7.674 7.655 7.633 7.612 7.595 7.519 7.492 7.470 7.452 7.355 7.339
2.500 Dimethyl Sulfoxide-d6
1.056
Ethanol
1.56
1.0 2.0 1.0 6.0 5.0 2.0
f1 (ppm)

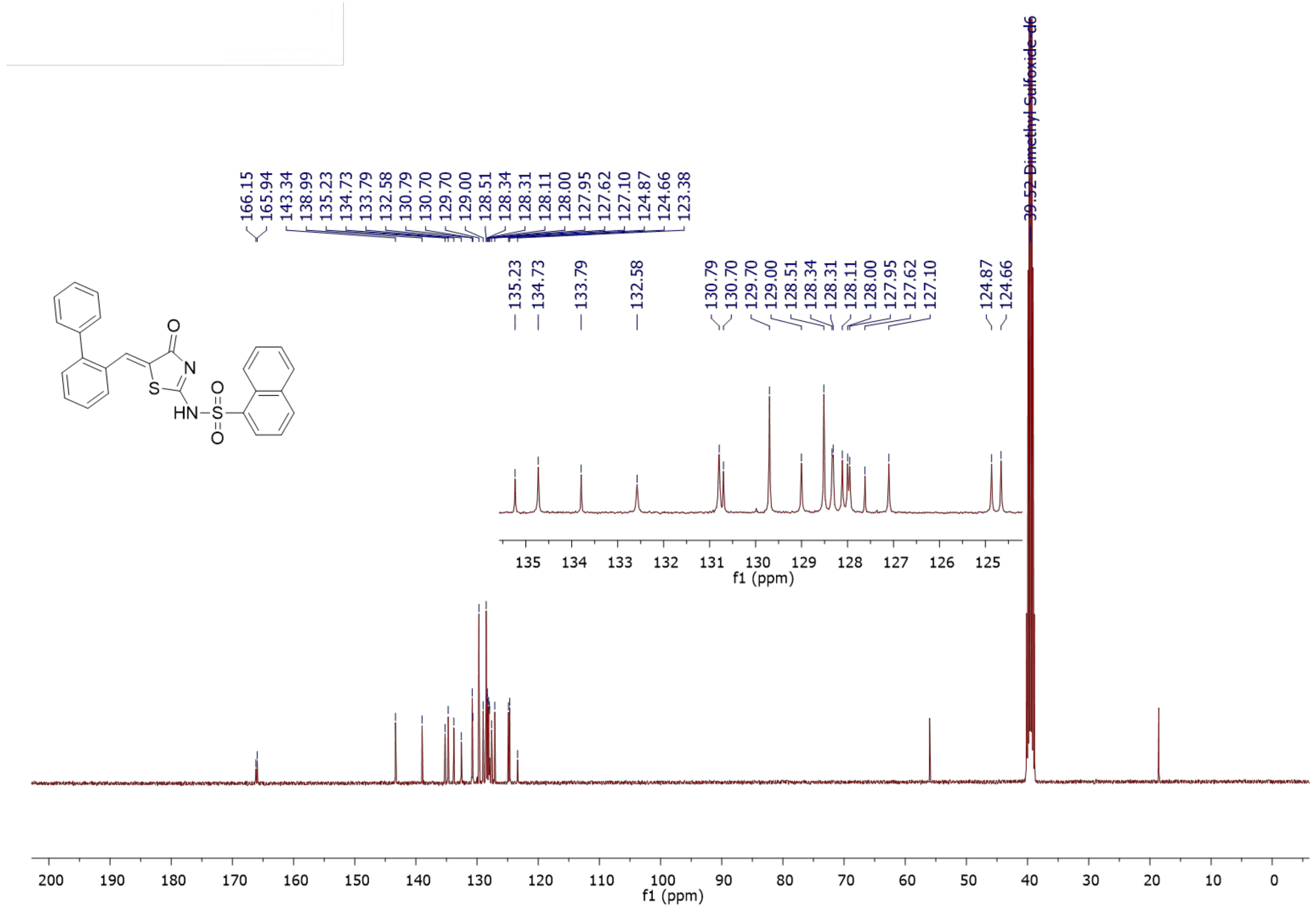

166.15 165.94 143.34 138.99 135.23 134.73 133.79 132.58 130.79 130.70 129.70 129.00 128.51 128.34 128.31 128.11 128.00 127.95 127.62 127.10 124.87 124.66 123.38
39.52 Dimethyl Sulfoxide-d6
f1 (ppm)

**Data file:**	D:\Chem32\1\Data\KP\kp_DEC4TH_80 2020-12-04 11-14-36\003-19-KP9192.D		
**Sample name:**	KP9192		
**Description:**			
**Sample amount:**	0.000	**Sample type:**	Sample
**Instrument:**	LCMS	**Location:**	19
**Injection date:**	12/4/2020 11:25:41 AM	**Injection:**	1 of 1
**Acq. method:**	LCMS ISOCRATIC 80% B_3 MINS.M	**Injection volume:**	2.000
**Analysis method:**	LCMS ISOCRATIC 80%B_3 MINS.M	**Acq. operator:**	SYSTEM
**Last changed:**	10/8/2020 2:52:31 PM		

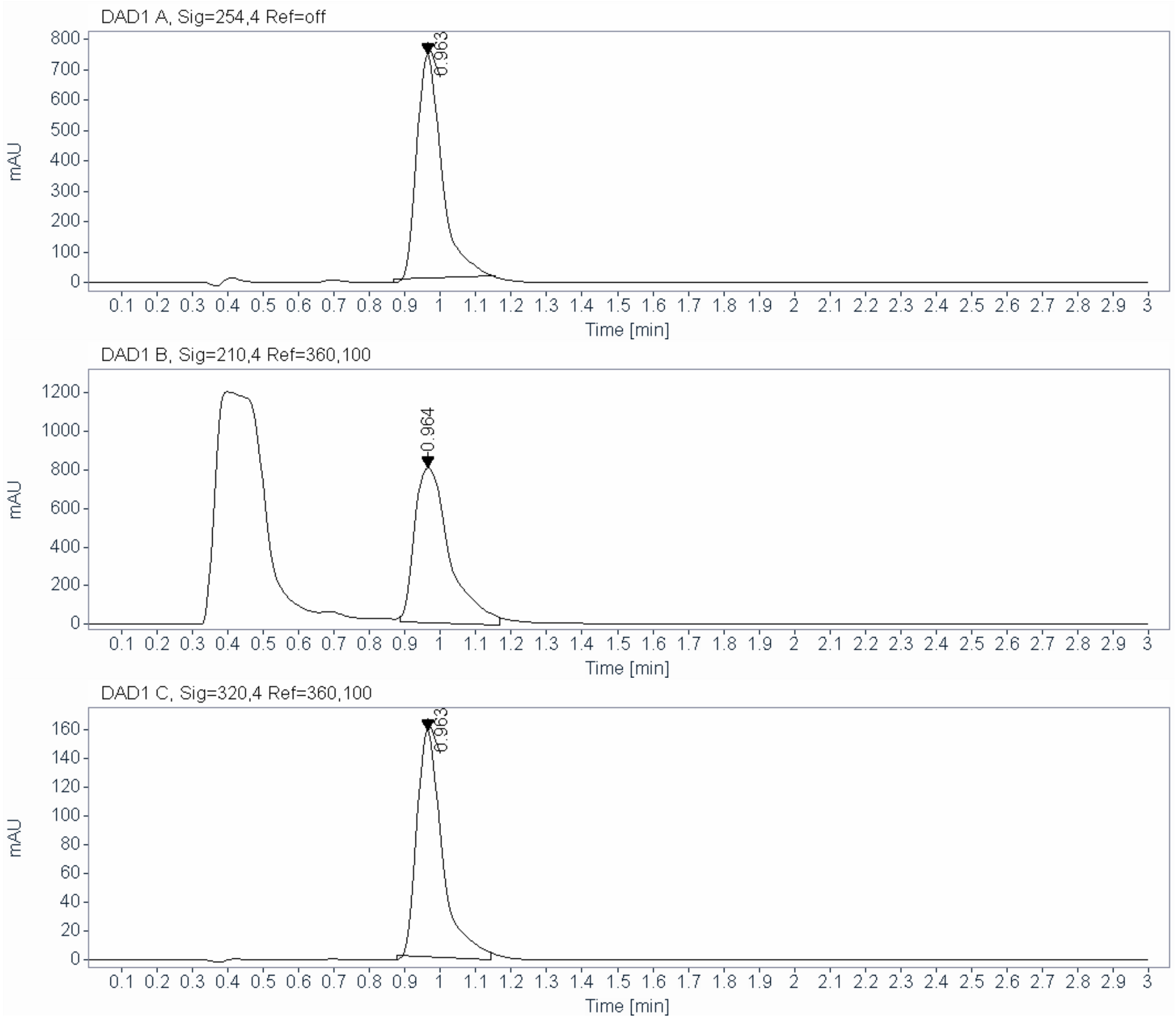

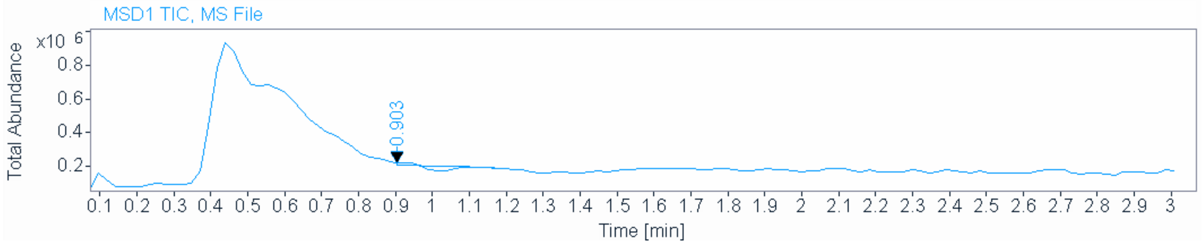

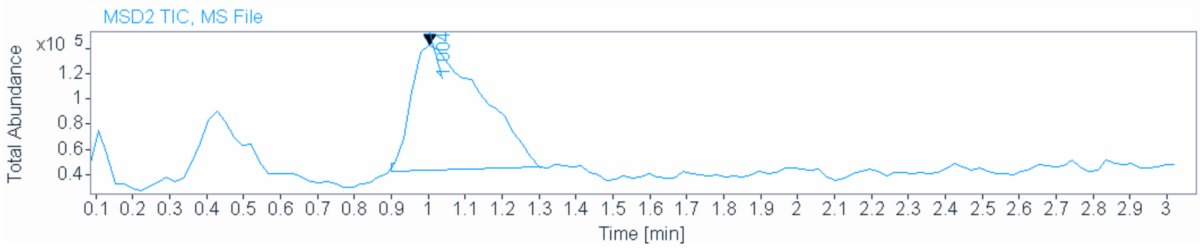

Signal: DAD1 A, Sig=254,4 Ref=off

RT [min]	Type	Width [min]	Area	Height	Area%	Name
0.963	MM	0.0812	3604.2437	739.6370	100.0000	
		Sum	3604.2437			

Signal: DAD1 B, Sig=210,4 Ref=360,100

RT [min]	Type	Width [min]	Area	Height	Area%	Name
0.964	MM	0.1145	5506.7695	801.8960	100.0000	
		Sum	5506.7695			

Signal: DAD1 C, Sig=320,4 Ref=360,100

RT [min]	Type	Width [min]	Area	Height	Area%	Name
0.963	MM	0.0834	789.8947	157.8998	100.0000	
		Sum	789.8947			

Signal: MSD1 TIC, MS File

RT [min]	Type	Width [min]	Area	Height	Area%	Name
0.903	MM	0.0521	41494.7070	16895.6270	100.0000	
		Sum	41494.7070			

Signal: MSD2 TIC, MS File

RT [min]	Type	Width [min]	Area	Height	Area%	Name
1.004	MM	0.2176	1298192.500<br>0	99434.9453	100.0000	
		Sum	1298192.500			

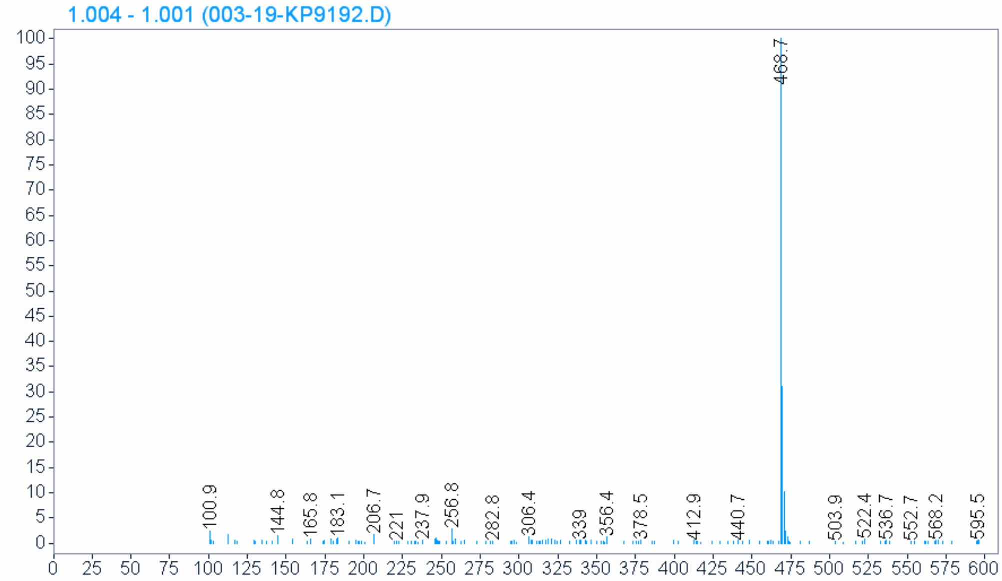
1.004 - 1.001 (003-19-KP9192.D)
468.7
100.9
144.8
165.8
183.1
206.7
221
237.9
256.8
282.8
306.4
339
356.4
378.5
412.9
440.7
503.9
522.4
536.7
552.7
568.2
595.5

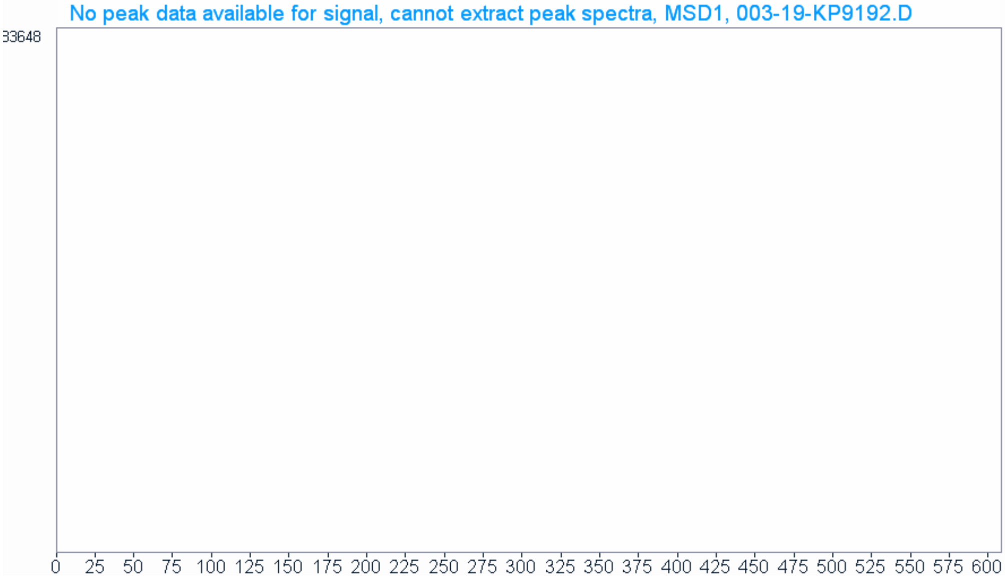
No peak data available for signal, cannot extract peak spectra, MSD1, 003-19-KP9192.D
33648

Name of Researcher: Kate Prichard

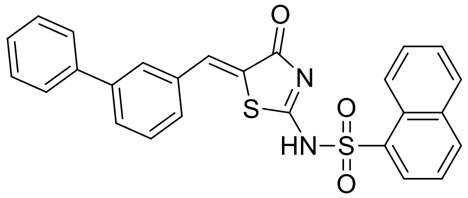

Chemical Formula: $C_{26}H_{18}N_2O_3S_2$
Exact Mass: 470.08
Molecular Weight: 470.56

Compound Name: (*Z*)-*N*-(5-([1,1'-biphenyl]-3-ylmethylene)-4-oxo-4,5-dihydrothiazol-2-yl)naphthalene-1-sulfonamide

Code: 17 (KP9138)

Obtained Weight & Yield: 93 mg (40%)

Purity (by LCMS and ^{1}H NMR): > 97% by ^{1}H NMR and LCMS

Appearance: light pink/orange solid

Solubility: DMSO, slightly soluble in acetone and methanol.

Melting Point: 201 – 202 °C

TLC Rf (and conditions): N/A

IR Analysis (including assignment): IR (neat): ν_{max} = 3131 (N-H), 2977 (C-H aromatic), 2887 (C-H alkyl), 1700 (C=O), 1557 (C-C aromatic), 1340 (sulfonamide), 1130 (C-N) cm^{-1}

^{1}H NMR Analysis: ^{1}H NMR (400 MHz, DMSO) δ 8.61 (d, *J* = 8.5 Hz, 1H), 8.33 – 8.27 (m, 2H), 8.12 (d, *J* = 8.1 Hz, 1H), 7.96 (s, 1H), 7.87 (s, 1H), 7.82 (d, *J* = 7.6 Hz, 1H), 7.79 – 7.64 (m, 7H), 7.53 (t, *J* = 7.6 Hz, 2H), 7.43 (t, *J* = 7.3 Hz, 1H) ppm.
NH exchanging – not visible.
Ethanol impurity at 1.05 ppm (2.42%)

^{13}C NMR Analysis: ^{13}C NMR (101 MHz, DMSO) δ 166.8 (br), 165.8 (br), 141.2, 139.1, 135.3, 134.7, 133.8, 133.64, 133.59, 130.1, 129.3, 129.2 (2C), 129.1, 129.0, 128.33, 128.27, 128.2, 128.1, 127.7, 127.1, 126.8 (2C), 124.9, 124.6, 122.5 ppm.
2C and broad peaks determined by 2D NMR.

MS Analysis (low res): LRMS (ESI-) *m/z*: 469 (*M*-H, $C_{26}H_{17}N_2O_3S_2$, 100)

HPLC method details: Column: Zorbax SB-C18 Rapid Resolution HT 2.1x50mm 1.8-Micron; Method: LCMS ISOCRATIC 80%B_3 MINS.M filename: KP9138; Peak retention time: 1.025 mins; Area (%): 100.

Procedure: To a 10 mL microwave vial was added N-(4-oxo-4,5-dihydrothiazol-2-yl)naphthalene-1-sulfonamide (148 mg, 0.49 mmol), 3-biphenylcarboxaldehyde (0.09 mL, 0.54 mmol, 1.1 eq), ethanol (3 mL) and a catalytic amount of the benzoic acid/piperidine catalyst (approximately 5 drops). The suspension was heated by microwave irradiation (120 °C, 200 W) for 40 min. A precipitate formed upon cooling. The solid was collected by vacuum filtration to give the desired product as a light pink/orange solid (93 mg, 40%).

Other analyses, reference papers, previously obtained data, comments, etc:

PerkinElmer Spectrum Version 10.4.00
Thursday, 26 November 2020 11:59 AM

Analyst	research
Date	Thursday, 26 November 2020 11:59 AM

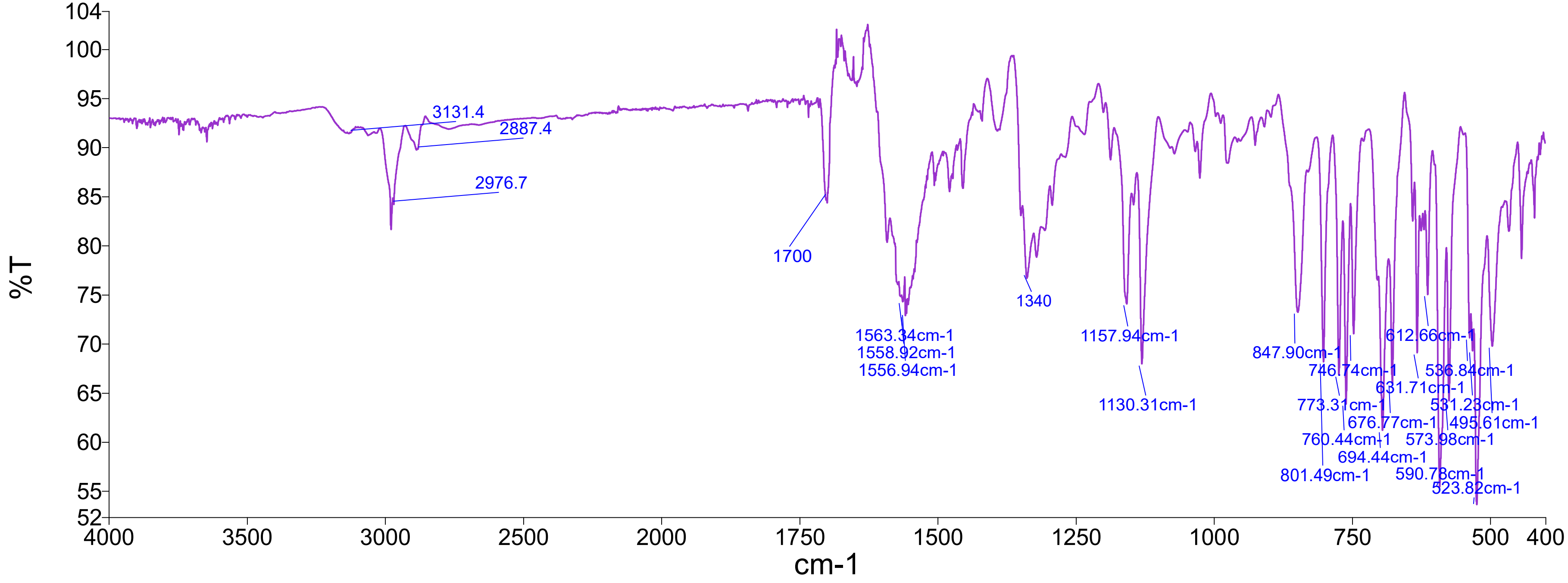

Sample Name	Description	Quality Checks
kp9138	Sample 188 By research Date Thursday, November 26 2020	The Quality Checks do not report any warnings for the sample.

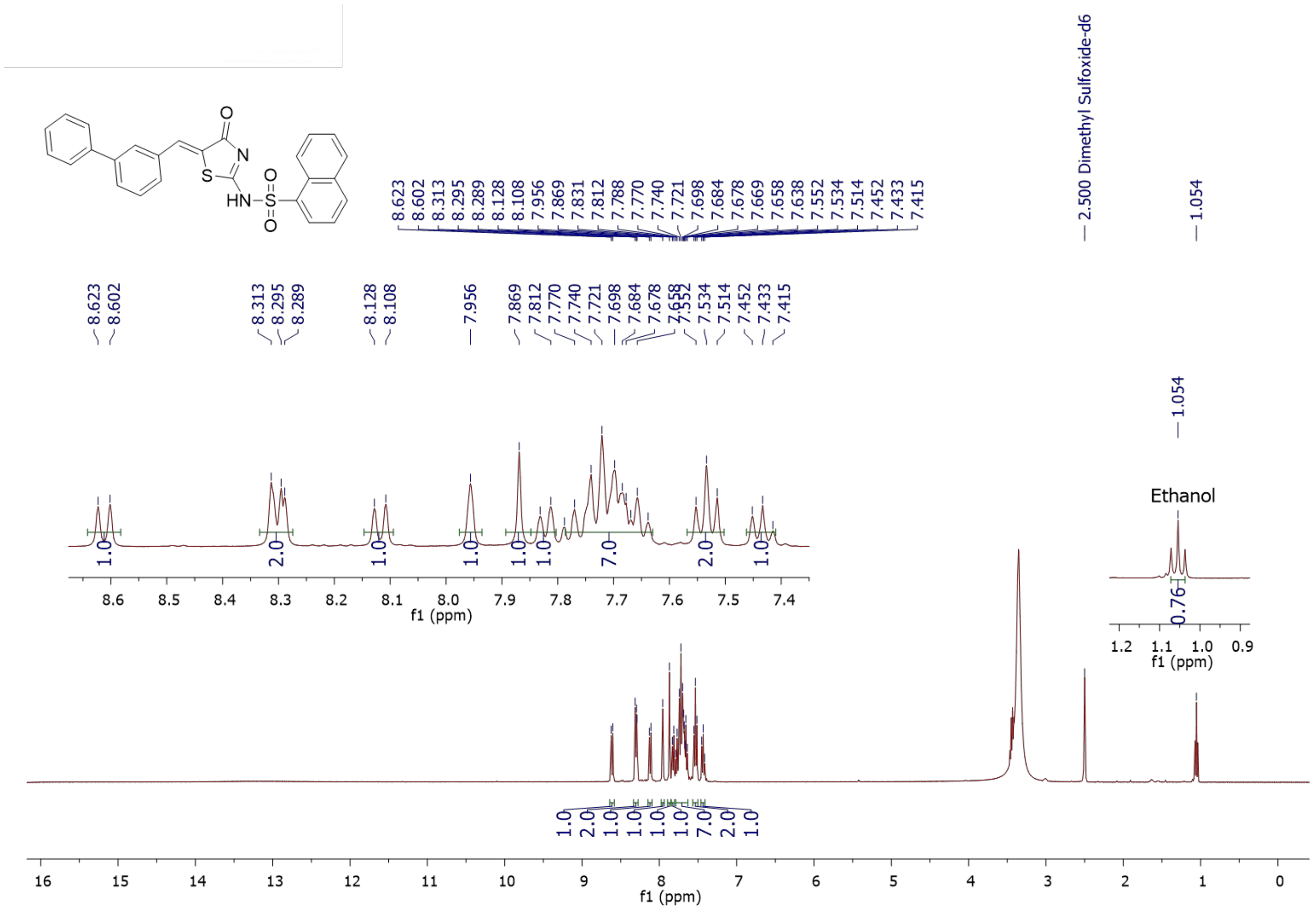

HN
S
N
O
O
O
8.623
8.602
8.313
8.295
8.289
8.128
8.108
7.956
7.869
7.831
7.812
7.788
7.770
7.740
7.721
7.698
7.684
7.678
7.669
7.658
7.638
7.552
7.534
7.514
7.452
7.433
7.415
2.500 Dimethyl Sulfoxide-d6
1.054
Ethanol
0.76
1.0
2.0
1.0
1.0
1.0
1.0
7.0
2.0
1.0
f1 (ppm)

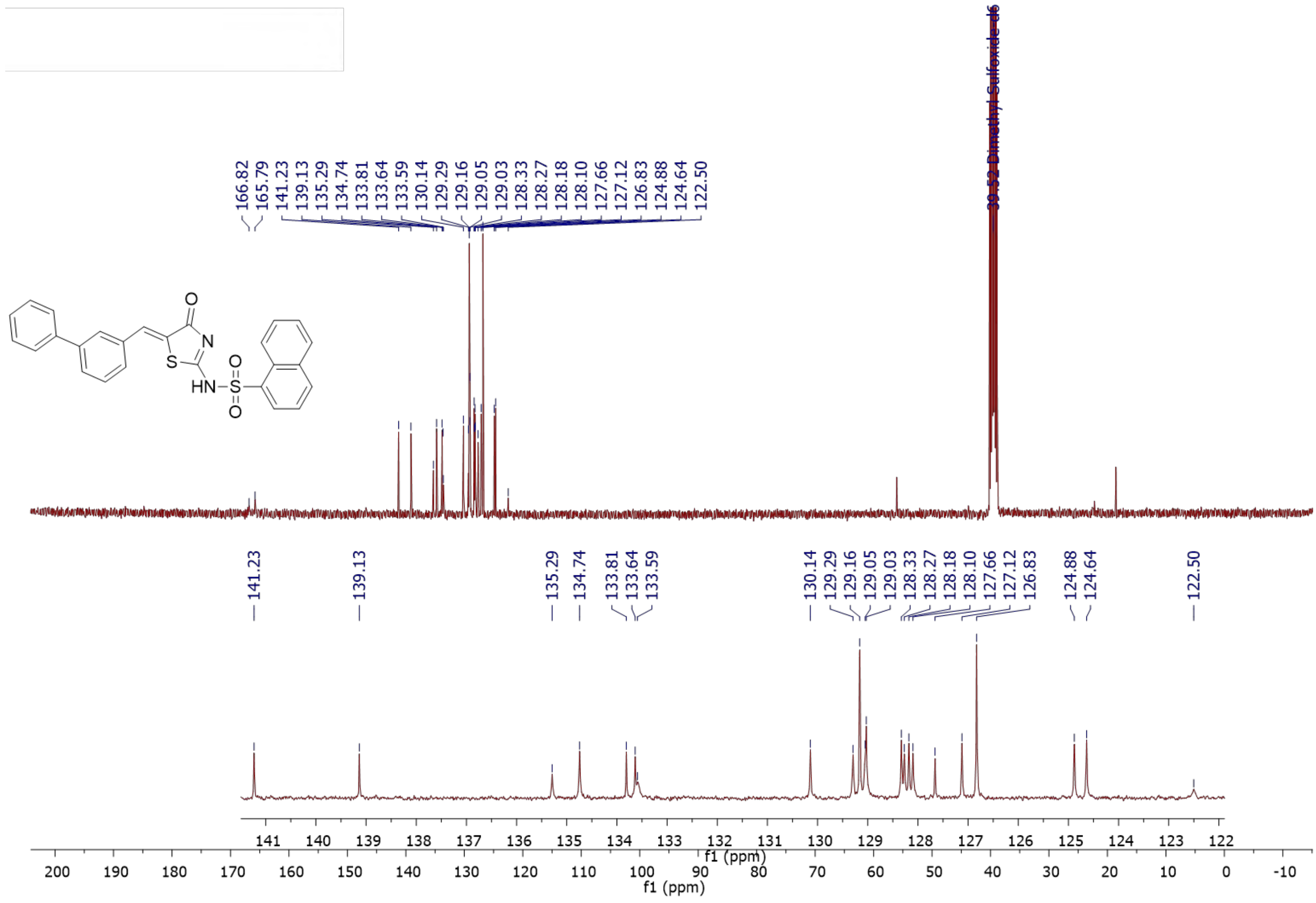

HN
S
N
O
O
O
166.82
165.79
141.23
139.13
135.29
134.74
133.81
133.64
133.59
130.14
129.29
129.16
129.05
129.03
128.33
128.27
128.18
128.10
127.66
127.12
126.83
124.88
124.64
122.50
39.52 Dimethyl Sulfoxide-d6
f1 (ppm)

LCMS Report

Agilent Technologies

**Data file:**	D:\Chem32\1\Data\KP\kp_DEC4TH_80 2020-12-04 11-14-36\004-18-KP9138.D		
**Sample name:**	KP9138		
**Description:**			
**Sample amount:**	0.000	**Sample type:**	Sample
**Instrument:**	LCMS	**Location:**	18
**Injection date:**	12/4/2020 11:30:18 AM	**Injection:**	1 of 1
**Acq. method:**	LCMS ISOCRATIC 80% B_3 MINS.M	**Injection volume:**	2.000
**Analysis method:**	LCMS ISOCRATIC 80%B_3 MINS.M	**Acq. operator:**	SYSTEM
**Last changed:**	10/8/2020 2:52:31 PM		

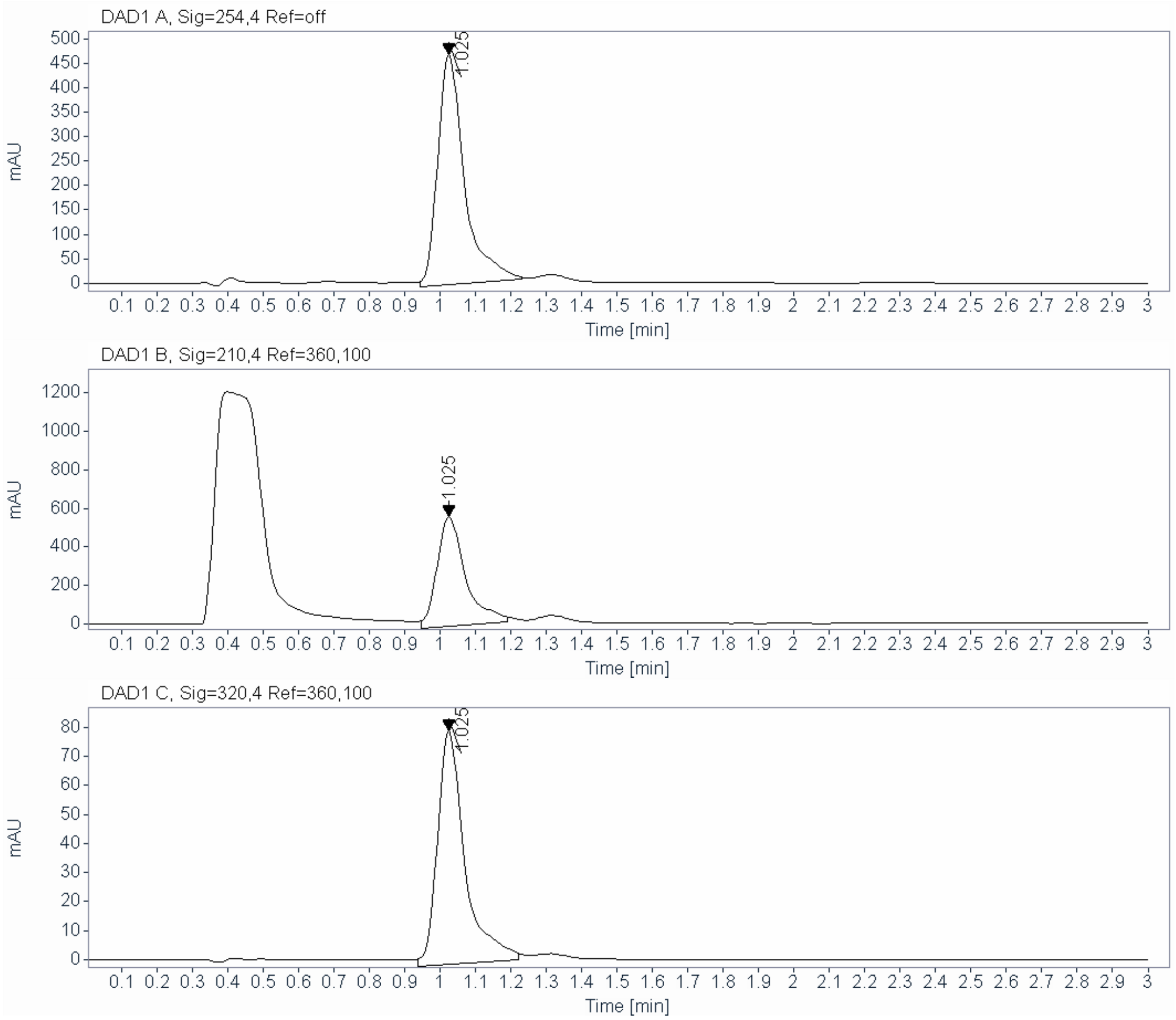

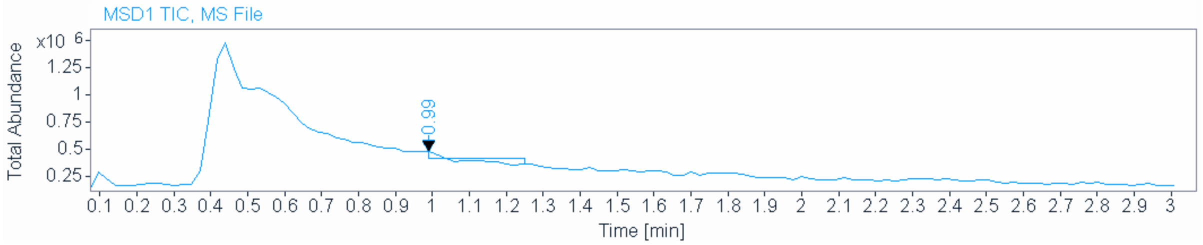

Agilent Technologies

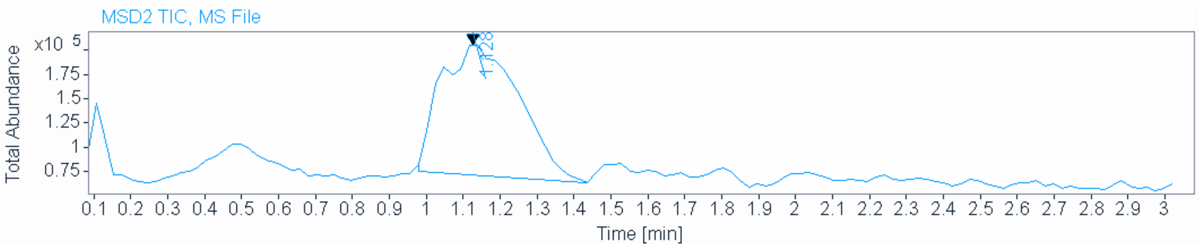

Signal: DAD1 A, Sig=254,4 Ref=off

RT [min]	Type	Width [min]	Area	Height	Area%	Name
1.025	MM	0.0873	2482.2795	473.6700	100.0000	
		Sum	2482.2795			

Signal: DAD1 B, Sig=210,4 Ref=360,100

RT [min]	Type	Width [min]	Area	Height	Area%	Name
1.025	MM	0.0927	3167.2795	569.5281	100.0000	
		Sum	3167.2795			

Signal: DAD1 C, Sig=320,4 Ref=360,100

RT [min]	Type	Width [min]	Area	Height	Area%	Name
1.025	MM	0.0892	432.0627	80.7298	100.0000	
		Sum	432.0627			

Signal: MSD1 TIC, MS File

RT [min]	Type	Width [min]	Area	Height	Area%	Name
0.990	MM	0.0285	66927.2500	59581.0430	100.0000	
		Sum	66927.2500			

Signal: MSD2 TIC, MS File

RT [min]	Type	Width [min]	Area	Height	Area%	Name
1.128	MM	0.2430	1976524.250 0	135558.2344	100.0000	
		Sum	1976524.250			

Agilent Technologies

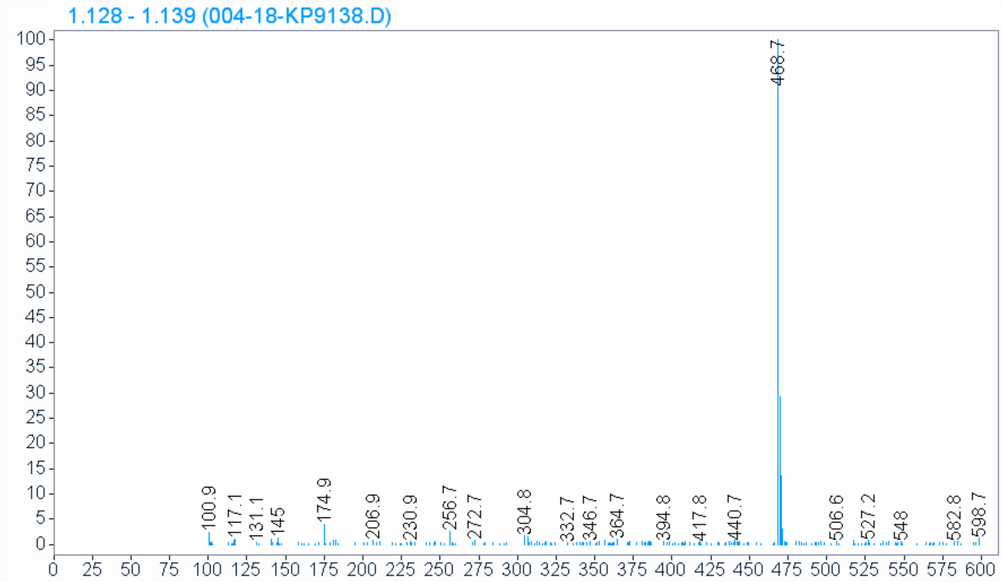

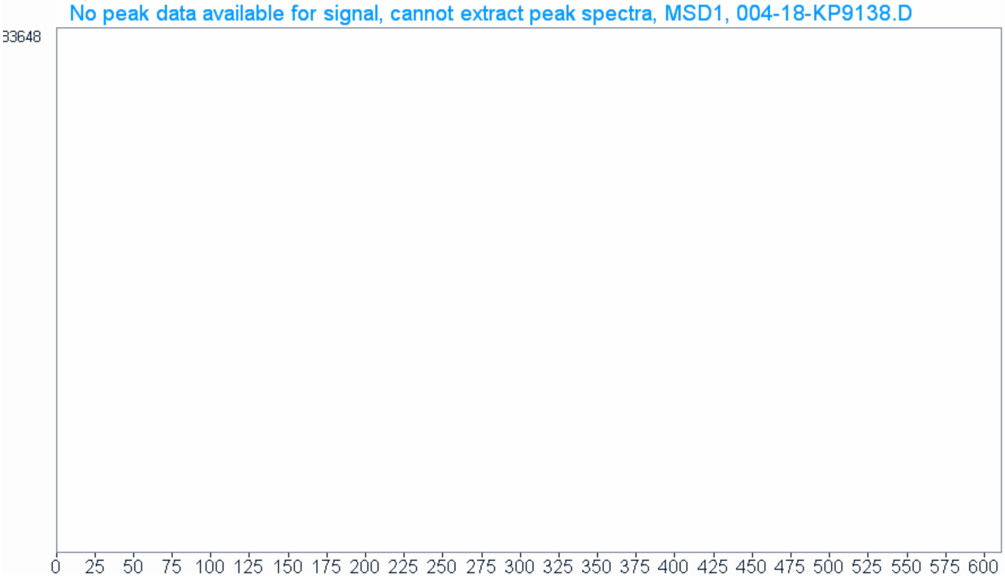

Compound Name: *N*-(5-([1,1'-biphenyl]-4-ylmethylene)-4-oxo-4,5-dihydrothiazol-2-yl)naphthalene-1-sulfonamide

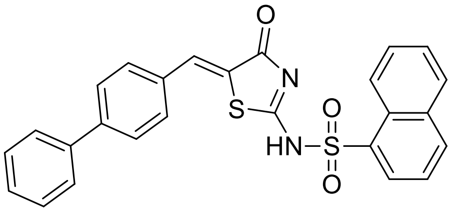

Chemical Formula: $C_{26}H_{18}N_2O_3S_2$
Exact Mass: 470.08
Molecular Weight: 470.56

Compound Code: 18 (KP5048)

Obtained Weight & Yield: 189 mg (82%)

Purity (by LCMS and ^{1}H NMR): > 99 % by ^{1}H-NMR

Appearance: Yellow solid

Solubility: DMSO, slightly soluble in ethyl acetate, acetone, ethanol and water

Melting Point: > 300 °C (dec.)

TLC Rf (and conditions): Rf 0.25 (10% MeOH in DCM)

IR Analysis (including assignment): IR (neat): ν_{max} = 3143, 2785, 2928 (C-H aromatic), 1713 (C=O), 1570 (N-H), 1141 (C-N) cm^{-1}

^{1}H NMR Analysis: ^{1}H NMR (400 MHz, DMSO) δ 8.63 (d, *J* = 8.5 Hz, 1H), 8.31 (t, *J* = 8.8 Hz, 2H), 8.12 (d, *J* = 8.1 Hz, 1H), 7.89 (d, *J* = 8.2 Hz, 2H), 7.79 - 7.67 (m, 8H), 7.51 (t, *J* = 7.5 Hz, 2H), 7.42 (t, *J* = 7.2 Hz, 1H) ppm.
Piperidine impurity at 1.55 ppm (0.90%)
NH exchanging – not observed

^{13}C NMR Analysis: ^{13}C NMR (151 MHz, DMSO) δ 142.1, 138.8, 135.5 (br), 134.6, 133.8, 132.7 (br), 132.0, 130.9 (2C), 129.1 (2C), 129.0, 128.3, 128.3, 128.1, 127.7, 127.6, 127.1 (2C), 126.9 (3C), 125.0, 124.7 ppm.
Carbonyl and sulfonamide quaternary carbons not visible
2C determined by 2D NMR

MS Analysis (low res): LRMS (ESI-) *m/z*: 487 (M-H+H_2O, $C_{26}H_{18}N_2O_3S_2$, 100%), 469 (M-H, $C_{26}H_{18}N_2O_3S_2$, 100%)

HPLC method details: Column: Zorbax SB-C18 Rapid Resolution HT 2.1x50mm 1.8-Micron; Method: LCMS ISOCRATIC 8O%B-6 MINS- 0.4ML.MIN.M filename: KP5048_80; Peak retention time: 1.04 mins; Area (%): 100.

Procedure: To a 10 mL microwave reaction vessel were added *N*-(4-oxo-4,5-dihydrothiazol-2-yl)naphthalene-1-sulfonamide (KP5122, 149 mg, 0.49 mmol, 1 eq), bihenyl-4-carboxaldehyde (98 mg, 0.54 mmol, 1.1 eq), benzoic acid/piperidine mix (1:1 mixture 10% in ethanol, catalytic amount 10 drops) and ethanol (3 mL). The resulting suspension was heated using microwave irradiation (200 W, 120 °C) for 30 min. After cooling overnight in freezer the precipitate was collected by vacuum filtration and washed with cold ethanol and cold diethyl ether to give the desired product as a yellow solid (189 mg, 82%).

Other analyses, reference papers, previously obtained data, comments, etc:

PerkinElmer Spectrum Version 10.4.00
Monday, 27 May 2019 1:37 PM

Analyst research
Date Monday, 27 May 2019 1:37 PM

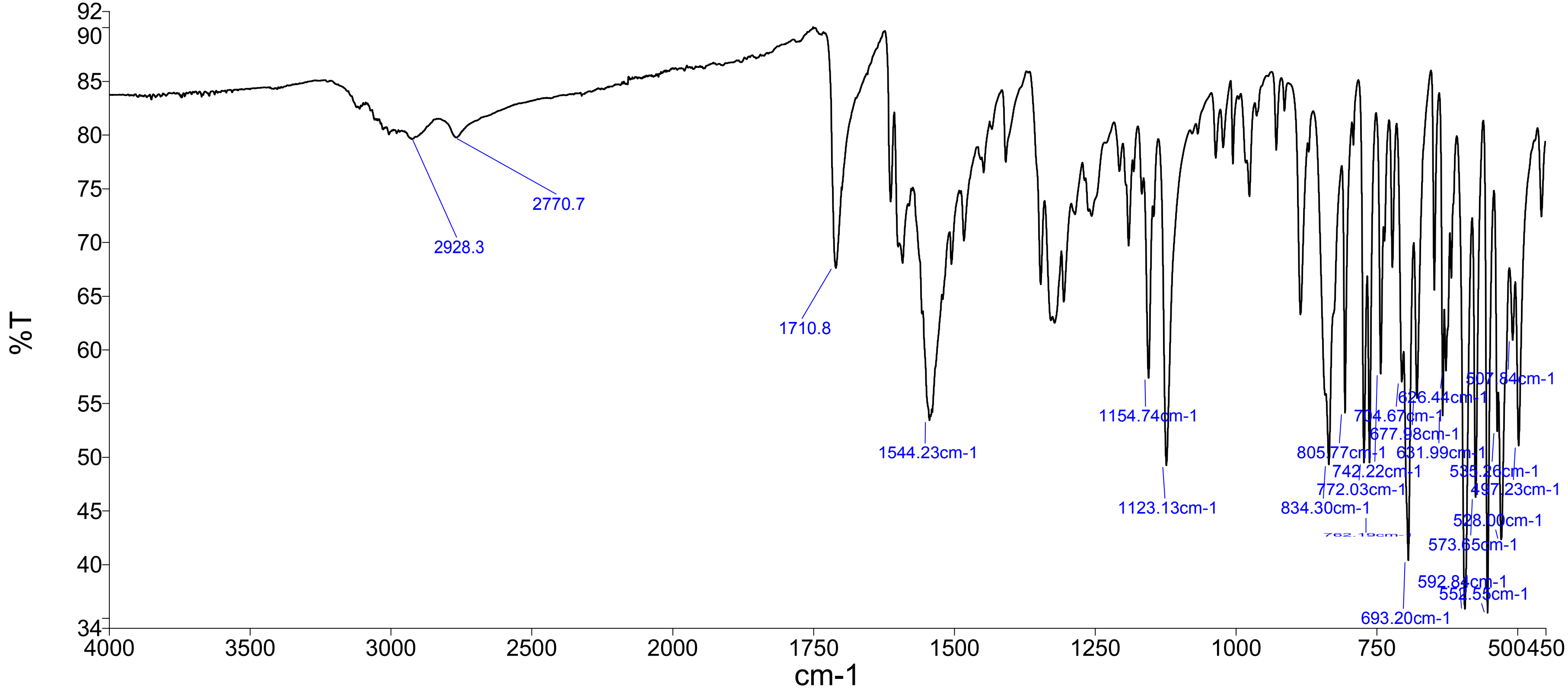

kp5148 Sample 072 By class Date Wednesday, May 08 2019

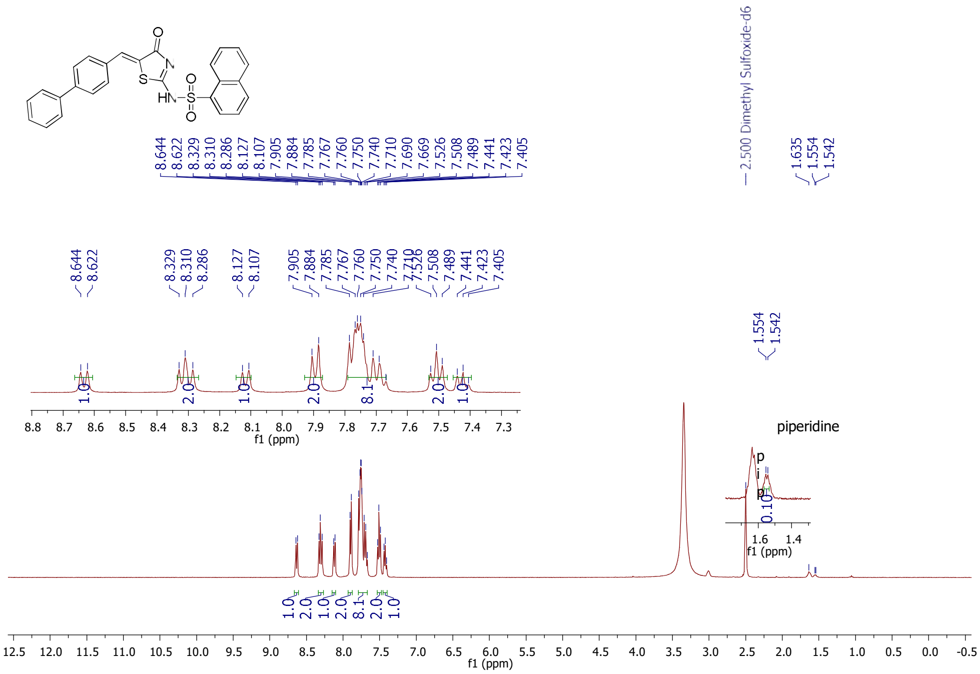

8.644 8.622 8.329 8.310 8.286 8.127 8.107 7.905 7.884 7.785 7.767 7.760 7.750 7.740 7.710 7.690 7.669 7.526 7.508 7.489 7.441 7.423 7.405
2.500 Dimethyl Sulfoxide-d6
1.635 1.554 1.542
piperidine
f1 (ppm)

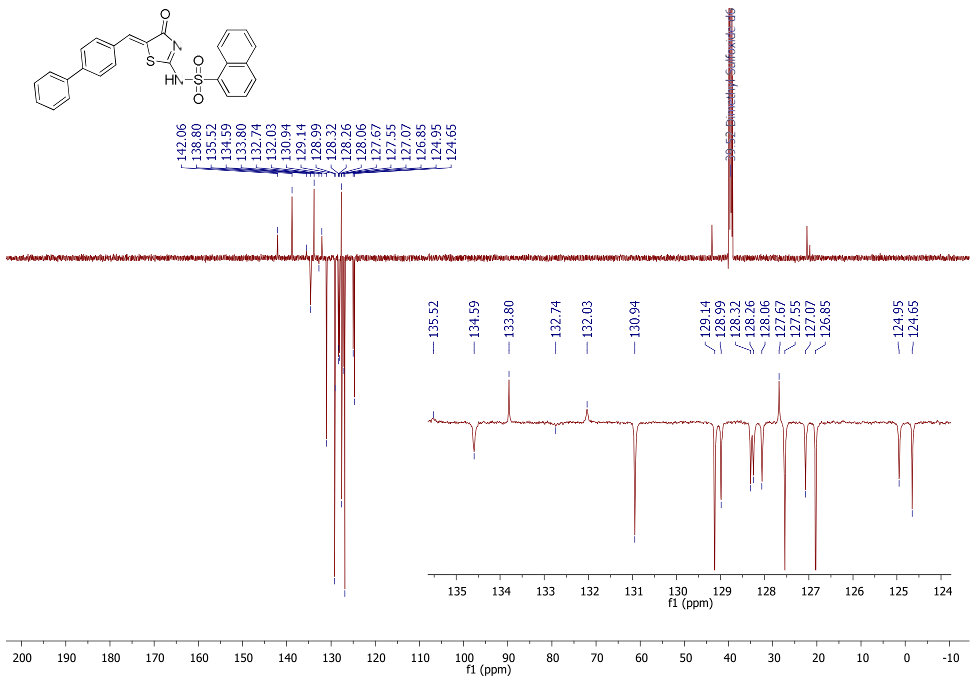

142.06 138.80 135.52 134.59 133.80 132.74 132.03 130.94 129.14 128.99 128.32 128.26 128.06 127.67 127.55 127.07 126.85 124.95 124.65
39.52 Dimethyl Sulfoxide-d6
f1 (ppm)

LCMS Report

Agilent Technologies

Data file: D:\Chem32\1\Data\KP\KP51486046 2019-05-24 10-44-56\002-33-KP5048_80.D
Sample name: KP5048_80
Description:
Sample amount: 0.000
Sample type: Sample

Instrument: LCMS
Injection date: 5/24/2019 10:54:13 AM
Acq. method: LCMS ISOCRATIC 8O%B-6 MINS-0.4ML.MIN.M
Analysis method: LCMS ISOCRATIC 8O%B-6 MINS-0.4ML.MIN.M
Last changed: 9/7/2018 10:31:27 AM
Location: 33
Injection: 1 of 1
Injection volume: 2.000
Acq. operator: SYSTEM

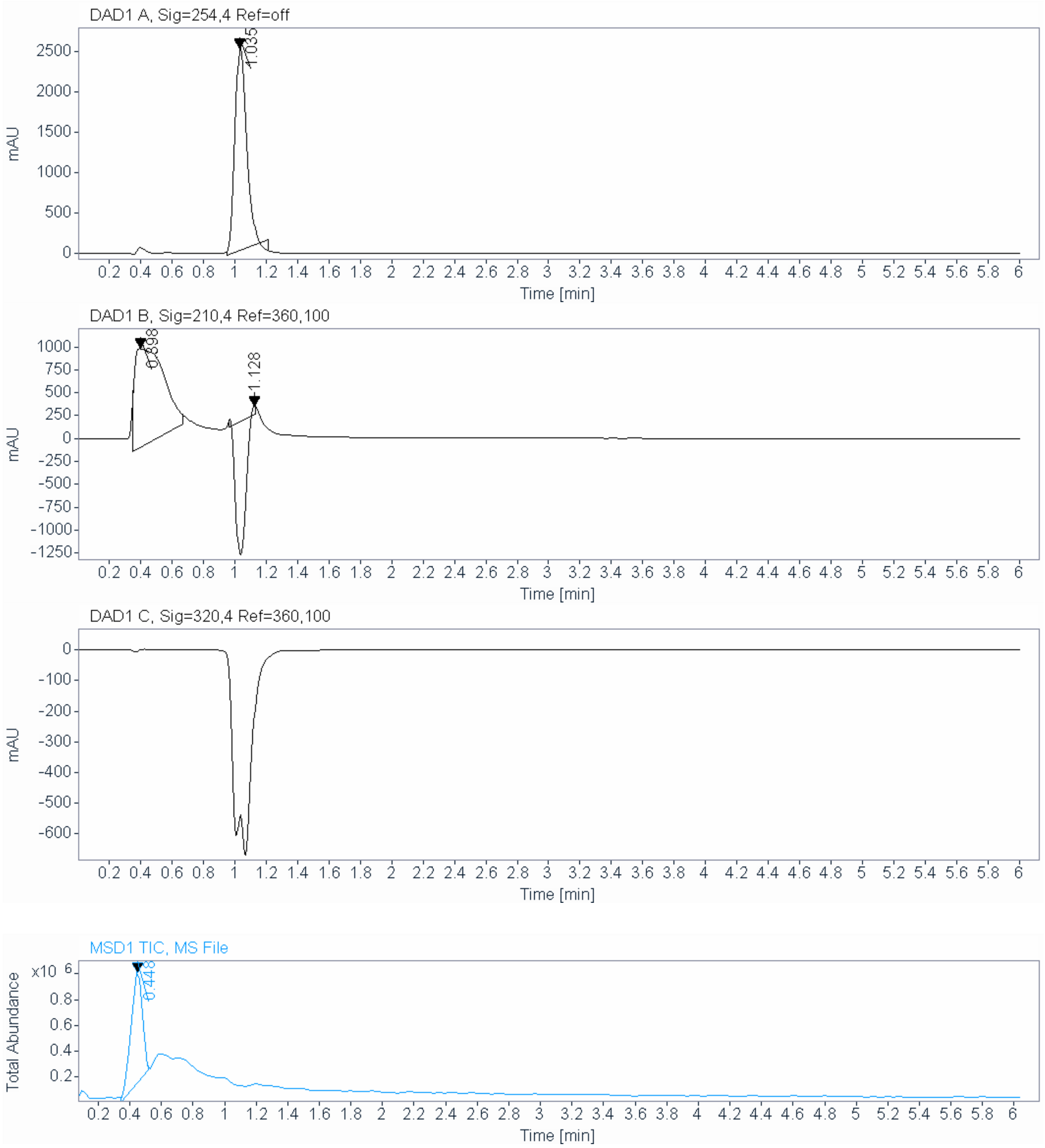

Agilent Technologies

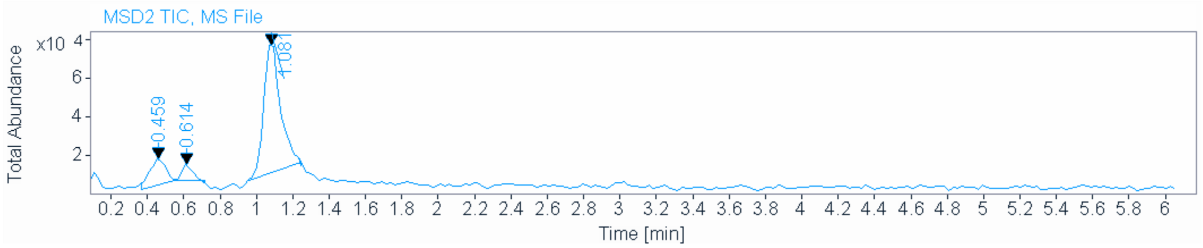

Signal: DAD1 A, Sig=254,4 Ref=off

RT [min]	Type	Width [min]	Area	Height	Area%	Name
1.035	MM	0.0863	12994.8955	2509.3445	100.0000	
		Sum	12994.8955			

Signal: DAD1 B, Sig=210,4 Ref=360,100

RT [min]	Type	Width [min]	Area	Height	Area%	Name
0.398	MM	0.2045	13313.0225	1085.2621	98.8524	
1.128	MM	0.0269	154.5516	95.6005	1.1476	
		Sum	13467.5741			

Signal: MSD1 TIC, MS File

RT [min]	Type	Width [min]	Area	Height	Area%	Name
0.448	MM	0.0793	4306428.000 0	905320.5000	100.0000	
		Sum	4306428.000			

Signal: MSD2 TIC, MS File

RT [min]	Type	Width [min]	Area	Height	Area%	Name
0.459	MM	0.0949	79376.3516	13944.9492	14.7604	
0.614	MM	0.0655	32599.4707	8291.6074	6.0620	
1.081	MM	0.1021	425787.9688	69506.3828	79.1775	
		Sum	537763.7910			

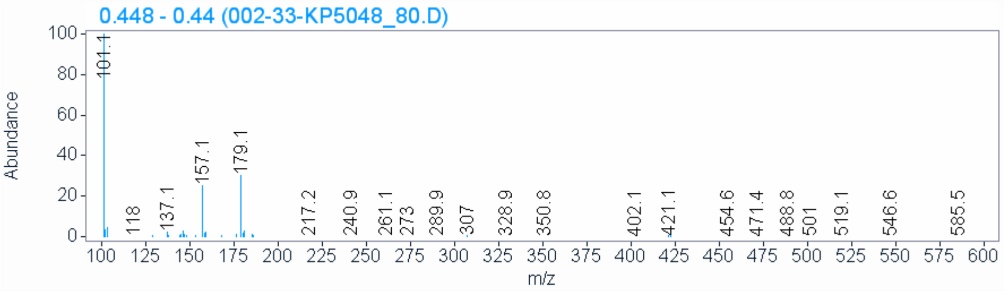
0.448 - 0.44 (002-33-KP5048_80.D)
Abundance
m/z
101.1
118
137.1
157.1
179.1
217.2
240.9
261.1
273
289.9
307
328.9
350.8
402.1
421.1
454.6
471.4
488.8
501
519.1
546.6
585.5

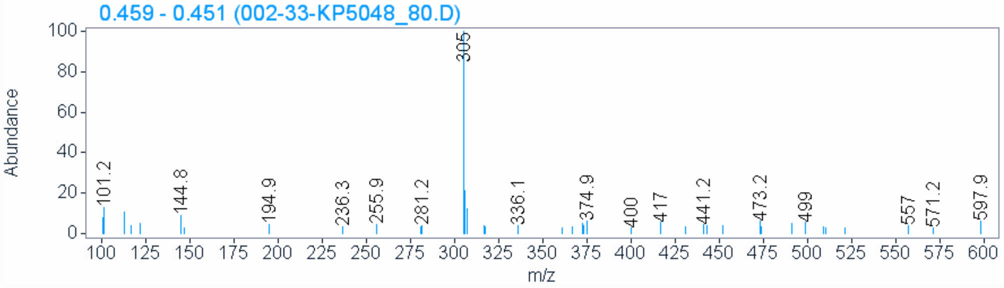
0.459 - 0.451 (002-33-KP5048_80.D)
Abundance
m/z
101.2
144.8
194.9
236.3
255.9
281.2
305
336.1
374.9
400
417
441.2
473.2
499
557
571.2
597.9

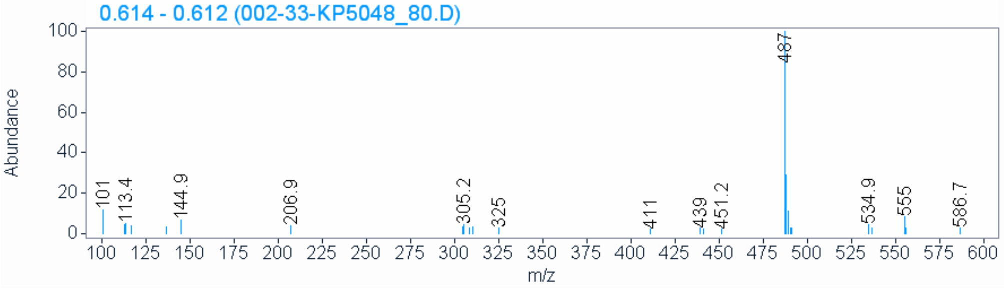
0.614 - 0.612 (002-33-KP5048_80.D)
Abundance
m/z
101
113.4
144.9
206.9
305.2
325
411
439
451.2
487
534.9
555
586.7

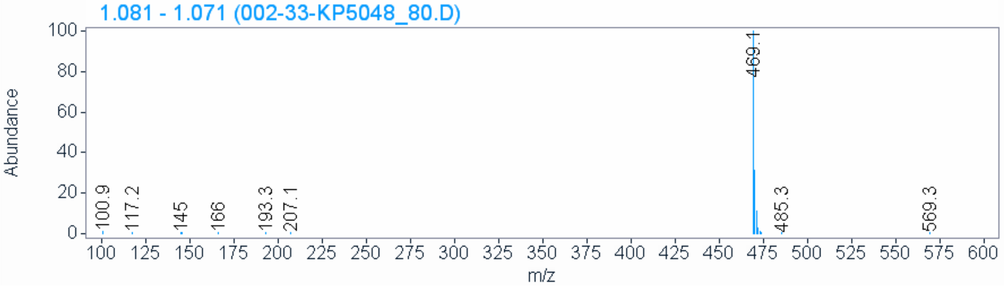
1.081 - 1.071 (002-33-KP5048_80.D)
Abundance
m/z
100.9
117.2
145
166
193.3
207.1
469.1
485.3
569.3

Compound Name: (*Z*)-*N*-(4-((2-(naphthalene-1-sulfonamido)-4-oxothiazol-5(4H)-ylidene)methyl)phenyl)acetamide

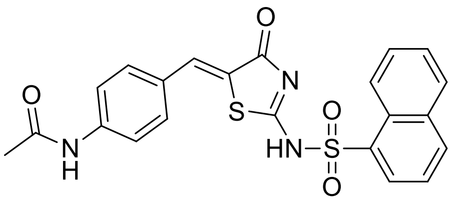

Chemical Formula: $C_{22}H_{17}N_3O_4S_2$
Exact Mass: 451.07
Molecular Weight: 451.52

Code: 19 (KP6193)

Obtained Weight & Yield: 144 mg (46%)

Purity (by LCMS and ^{1}H NMR): > 98% by ^{1}H NMR

Appearance: yellow solid

Solubility: DMSO, slightly soluble in acetone and methanol

Melting Point: > 291 °C (dec.)

TLC Rf (and conditions): N/A

IR Analysis (including assignment): IR (neat): ν_{max} = 2765 (C-H aromatic), 1732 (acetomaide), 1668 (C=O), 1578 (C-C aromatic), 1321 (sulfonamide), 1180 (C-N) cm^{-1}

^{1}H NMR Analysis: ^{1}H NMR (400 MHz, DMSO) δ 13.13 (br, s, 1H, NH), 10.31 (s, 1H, NH), 8.62 (d, *J* = 8.6 Hz, 1H), 8.30 (dd, *J* = 12.1, 7.8 Hz, 2H), 8.11 (d, *J* = 8.1 Hz, 1H), 7.79 (d, *J* = 8.6 Hz, 2H), 7.76 – 7.66 (m, 4H), 7.61 (d, *J* = 8.6 Hz, 2H), 2.09 (s, 3H) ppm.
DMF at 2.73 ppm (0.80%)

^{13}C NMR Analysis: ^{13}C NMR (151 MHz, DMSO) δ 168.9, 141.6, 135.6 (br), 134.5, 133.8, 133.0 (br), 131.5 (2C), 129.0, 128.2, 128.1, 127.7, 127.4, 127.0, 125.0, 124.6, 119.24 (2C), 24.2 ppm.
2C determined by 2D NMR. 3 quaternary carbons missing.

MS Analysis (low res): LRMS (ESI-) *m/z* (%): 450 (*M*-H, $C_{22}H_{16}N_3O_4S_2$, 100%)

HPLC method details: Column: Zorbax SB-C18 Rapid Resolution HT 2.1x50mm 1.8-Micron; Method: LCMS ISOCRATIC 60%B 0.4MLMIN-1.M filename: KP6193; Peak retention time: 0.655 mins; Area (%): 100.

Procedure: To a 10 mL microwave vial was added *N*-(4-oxo-4,5-dihydrothiazol-2-yl)naphthalene-1-sulfonamide (200 mg, 0.65 mmol), 4-acetomide benzaldehyde (118 mg, 0.72 mmol, 1.1 eq), ethanol (3 mL) and a catalytic amount of the benzoic acid/piperidine catalyst (approximately 5 drops). The suspension was heated using microwave irradiation (200 W, 120 °C) for 30 min then allowed to precipitate in the freezer. The resulting precipitate was collected by vacuum filtration and washed with cold ethanol and cold ether to give the desired product (144 mg, 46%).

Other analyses, reference papers, previously obtained data, comments, etc:

PerkinElmer Spectrum Version 10.4.00
Wednesday, 6 November 2019 2:30 PM

Analyst research
Date Wednesday, 6 November 2019 2:30 PM

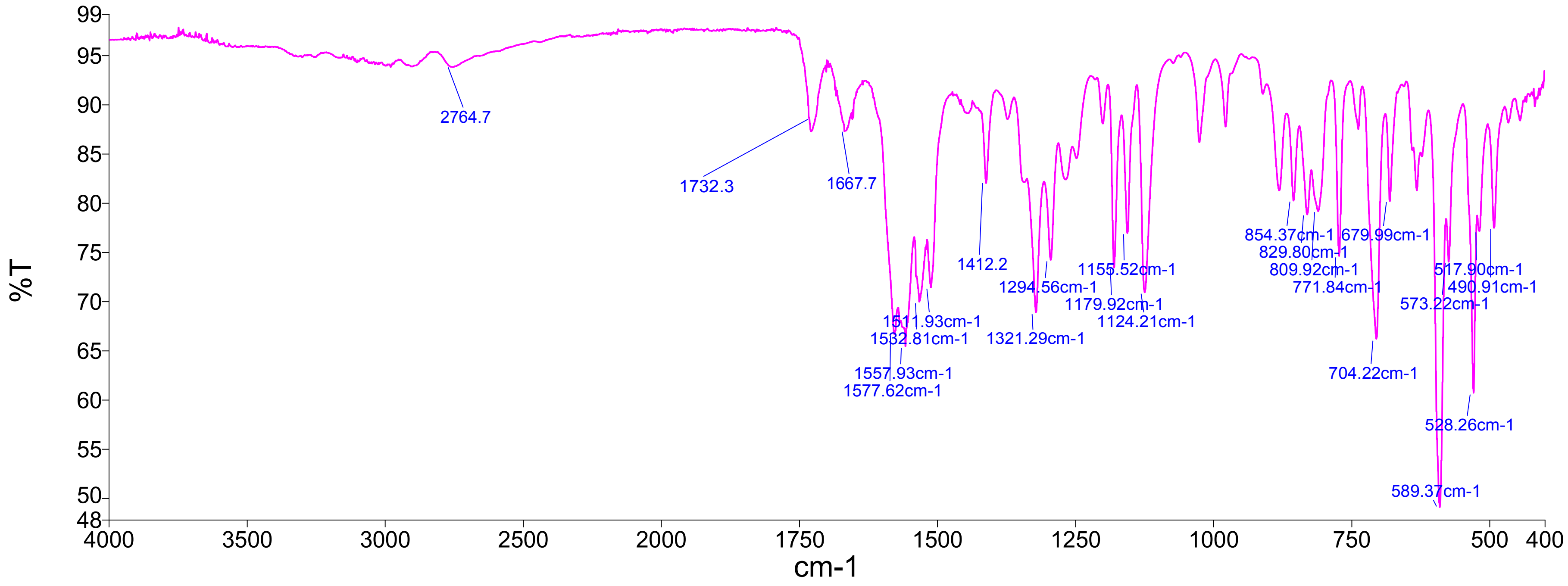

Sample Name	Description	Quality Checks
kp6193	Sample 242 By research Date Wednesday, November 06 2019	The Quality Checks do not report any warnings for the sample.

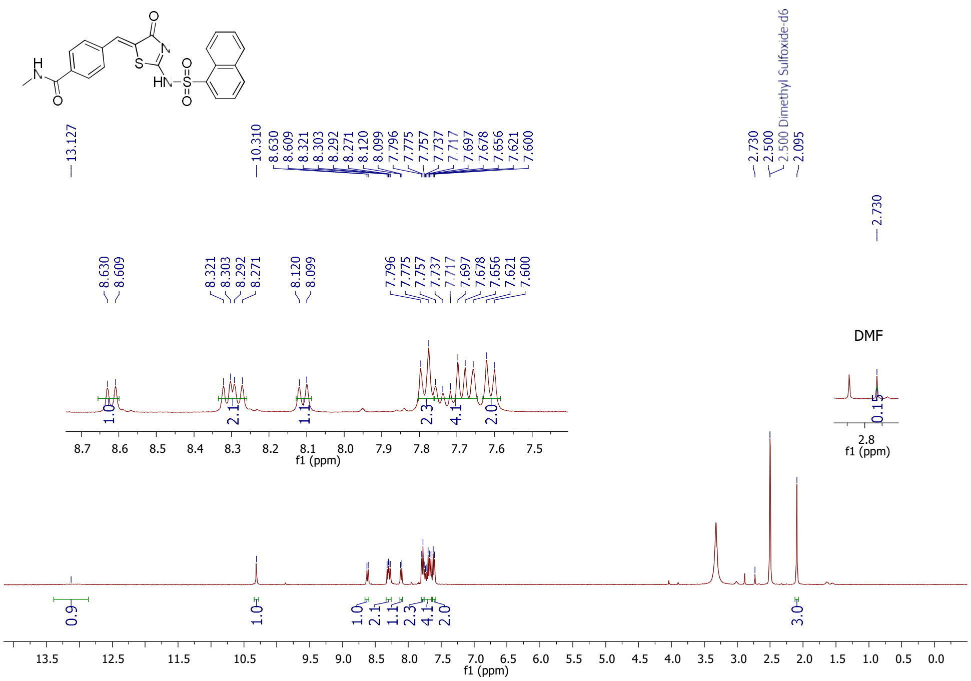
13.127
10.310
8.630
8.609
8.321
8.303
8.292
8.271
8.120
8.099
7.796
7.775
7.757
7.737
7.717
7.697
7.678
7.656
7.621
7.600
2.730
2.500
2.500 Dimethyl Sulfoxide-d6
2.095
DMF
0.9
1.0
2.1
1.1
2.3
4.1
2.0
3.0
0.15
f1 (ppm)

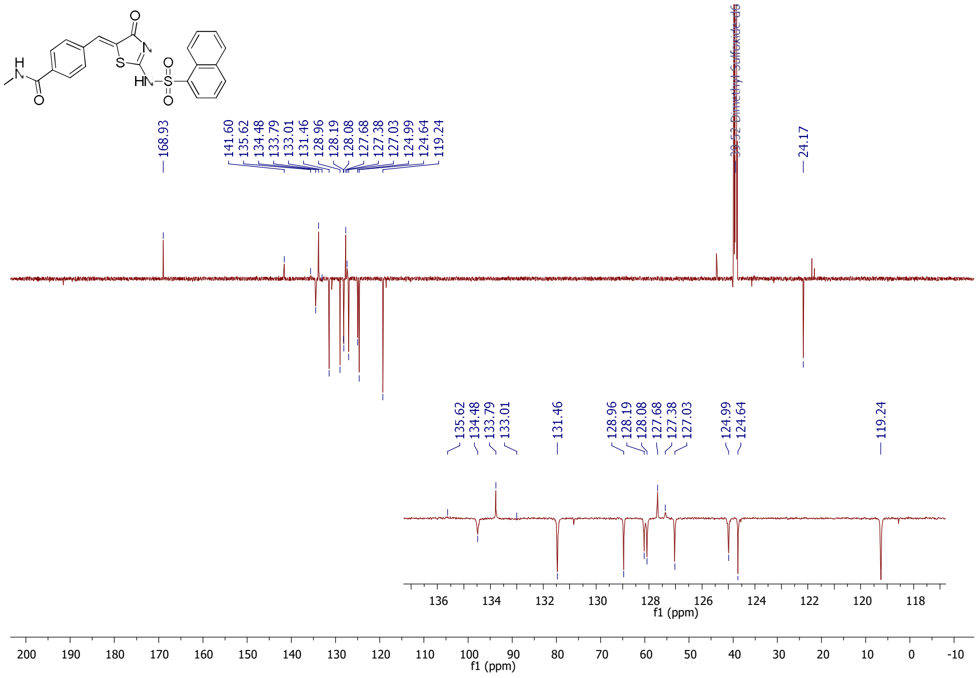
168.93
141.60
135.62
134.48
133.79
133.01
131.46
128.96
128.19
128.08
127.68
127.38
127.03
124.99
124.64
119.24
39.52 Dimethyl Sulfoxide-d6
24.17
f1 (ppm)

Agilent Technologies

**Data file:**	D:\Chem32\1\Data\KP\KP_DS_11NOV 2019-11-11 14-45-23\008-47-KP6193.D		
**Sample name:**	KP6193		
**Description:**			
**Sample amount:**	0.000	**Sample type:**	Sample
**Instrument:**	LCMS	**Location:**	47
**Injection date:**	11/11/2019 3:41:33 PM	**Injection:**	1 of 1
**Acq. method:**	LCMS ISOCRATIC 60% B 0.4MLMIN-1.M	**Injection volume:**	2.000
**Analysis method:**	LCMS ISOCRATIC 60%B 0.4MLMIN-1.M	**Acq. operator:**	SYSTEM
**Last changed:**	5/8/2019 8:55:04 AM		

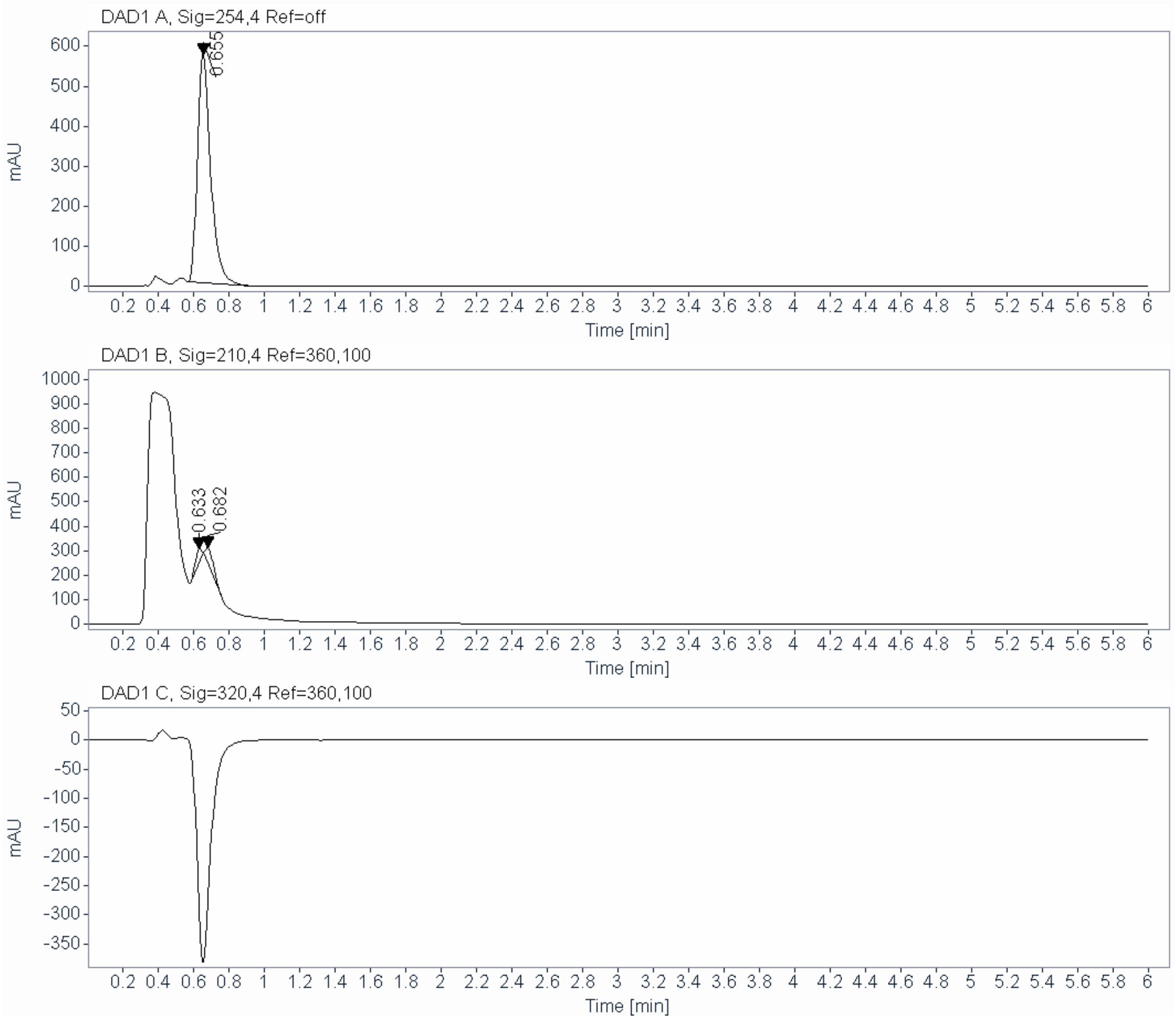

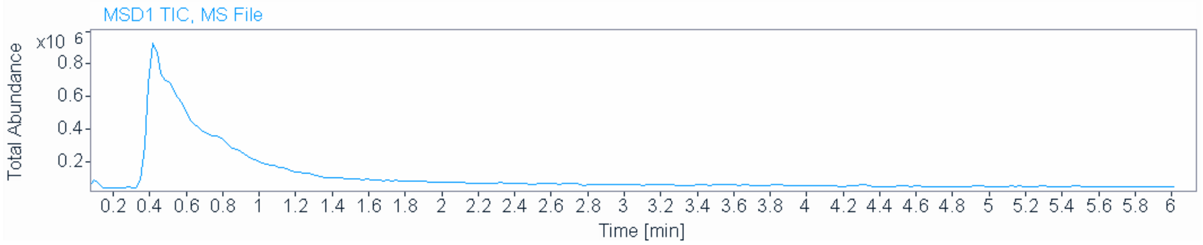

Agilent Technologies

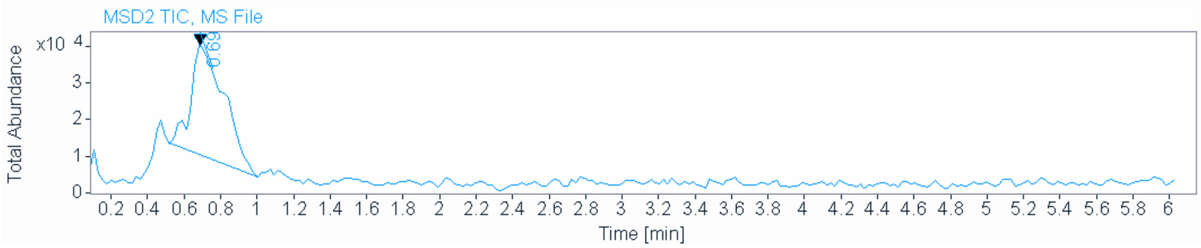

Signal: DAD1 A, Sig=254,4 Ref=off

RT [min]	Type	Width [min]	Area	Height	Area%	Name
0.655	BB	0.0813	3015.1162	573.9601	100.0000	
		Sum	3015.1162			

Signal: DAD1 B, Sig=210,4 Ref=360,100

RT [min]	Type	Width [min]	Area	Height	Area%	Name
0.633	BB	0.0378	138.9244	58.2053	39.2902	
0.682	BB	0.0519	214.6606	66.1239	60.7098	
		Sum	353.5850			

Signal: MSD2 TIC, MS File

RT [min]	Type	Width [min]	Area	Height	Area%	Name
0.690	BB	0.1842	390029.1250	30370.6133	100.0000	
		Sum	390029.1250			

Agilent Technologies

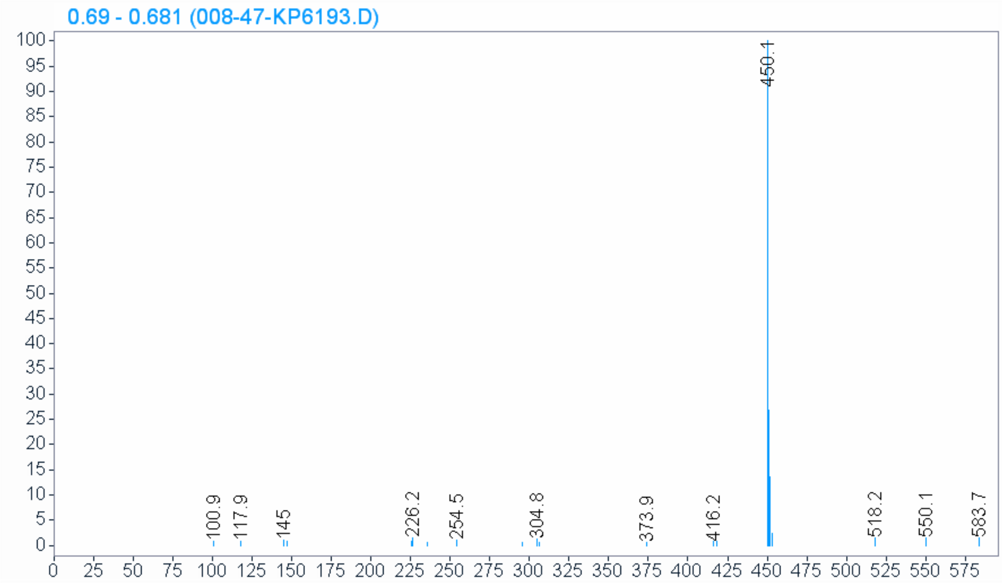

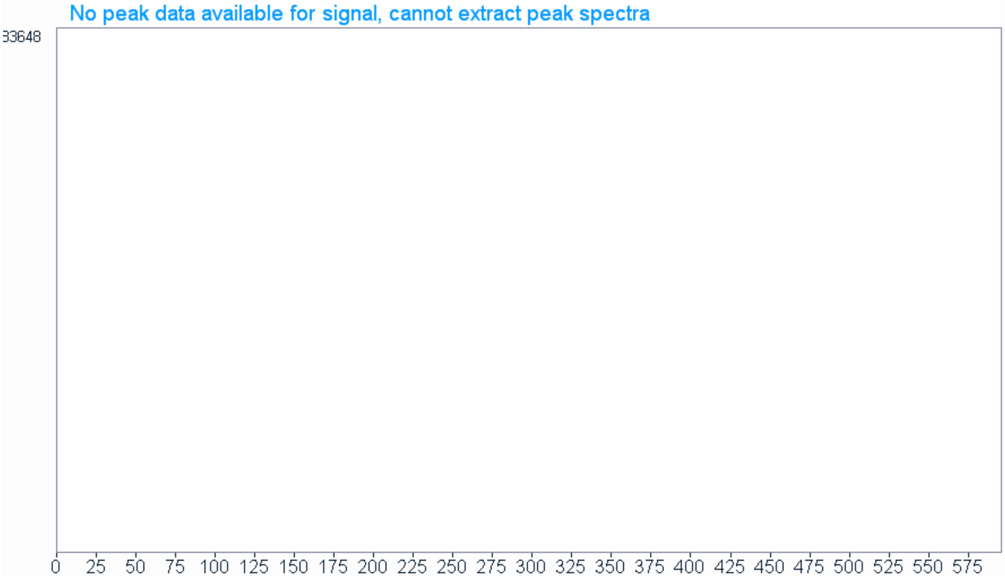

Compound Name: (Z)-*N*-(5-(4-(tert-butyl)benzylidene)-4-oxo-4,5-dihydrothiazol-2-yl)naphthalene-1-sulfonamide

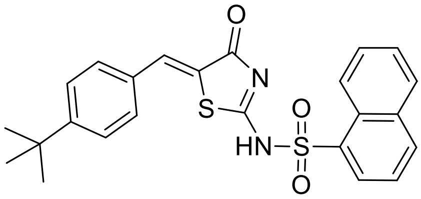

Chemical Formula: $C_{24}H_{22}N_2O_3S_2$
Exact Mass: 450.11
Molecular Weight: 450.57

Compound Code: 20 (KP7118)

Obtained Weight & Yield: 106 mg, 48%

Purity (by LCMS and ^{1}H NMR): >99% by ^{1}H-NMR, >99% by LCMS

Appearance: pale yellow solid

Solubility: DMSO, slightly soluble in acetone and methanol

Melting Point: 261 – 262 °C

TLC Rf (and conditions): N/A

IR Analysis (including assignment): IR (neat): 3127 (N-H), 3062 (C-H aromatic), 2959 (C-H), 1707 (C=O), 1545 (aromatic C-C), 1327 (sulfonamide), 1130 (C-N) cm^{-1}

^{1}H NMR Analysis: ^{1}H NMR (400 MHz, DMSO) δ 8.61 (d, *J* = 8.6 Hz, 1H), 8.31 (dd, *J* = 7.8, 3.0 Hz, 2H), 8.12 (d, *J* = 8.0 Hz, 1H), 7.79 – 7.75 (m, 2H), 7.73 – 7.67 (m, 2H), 7.61 (s, 4H), 1.31 (s, 9H) ppm.
NH exchanging – not visible
Ethanol at 1.05 ppm (0.54%)

^{13}C NMR Analysis: ^{13}C NMR (101 MHz, DMSO) δ 166.5, 165.7, 154.1, 135.3, 134.7, 133.8, 133.7, 130.2 (2C), 130.1, 129.0, 128.3, 128.1, 127.6, 127.1, 126.4 (2C), 124.9, 124.6, 120.8, 34.8, 30.8 (3C) ppm.
2C and 3C determined by 2D NMR.

MS Analysis (low res): LRMS (ESI-) *m/z* (%): 449 (*M*-H, $C_{24}H_{21}N_2O_3S_2$, 100%); (ESI+) *m/z* (%): 451 (*M*+H, $C_{24}H_{23}N_2O_3S_2$, 100%)

MS Analysis (high res): Exact mass calculated for $C_{24}H_{21}N_2O_3S_2$ [*M*-H]$^-$, 449.1000. Found 449.0998.

HPLC method details: Column: Zorbax SB-C18 Rapid Resolution HT 2.1x50mm 1.8-Micron; Method: LCMS ISOCRATIC 80%B_3MIN.M filename: KP7118_80; Peak retention time: 1.291 mins; Area (%): 100

Procedure: To a 10mL microwave vial was added the *N*-(4-oxo-4,5-dihydrothiazol-2-yl)naphthalene-1-sulfonamide (153 mg, 0.49 mmol), 4-(tert-butyl)benzaldehyde (0.1 mL, 0.54 mmol, 1.1 eq), ethanol (3 mL) and a catalytic amount of the benzoic acid/piperidine catalyst (approximately 5 drops). The suspension was heated using microwave irradiation (200 W, 120 °C) for 30 min. Precipitation occurred upon shaking of MW vial. The resulting precipitate was collected by vacuum filtration and washed with cold ethanol and cold ether to give the desired product (106 mg, 48%)

Other analyses, reference papers, previously obtained data, comments, etc:

PerkinElmer Spectrum Version 10.4.00
Thursday, 21 November 2019 11:38 AM

Analyst research

Date Thursday, 21 November 2019 11:38 AM

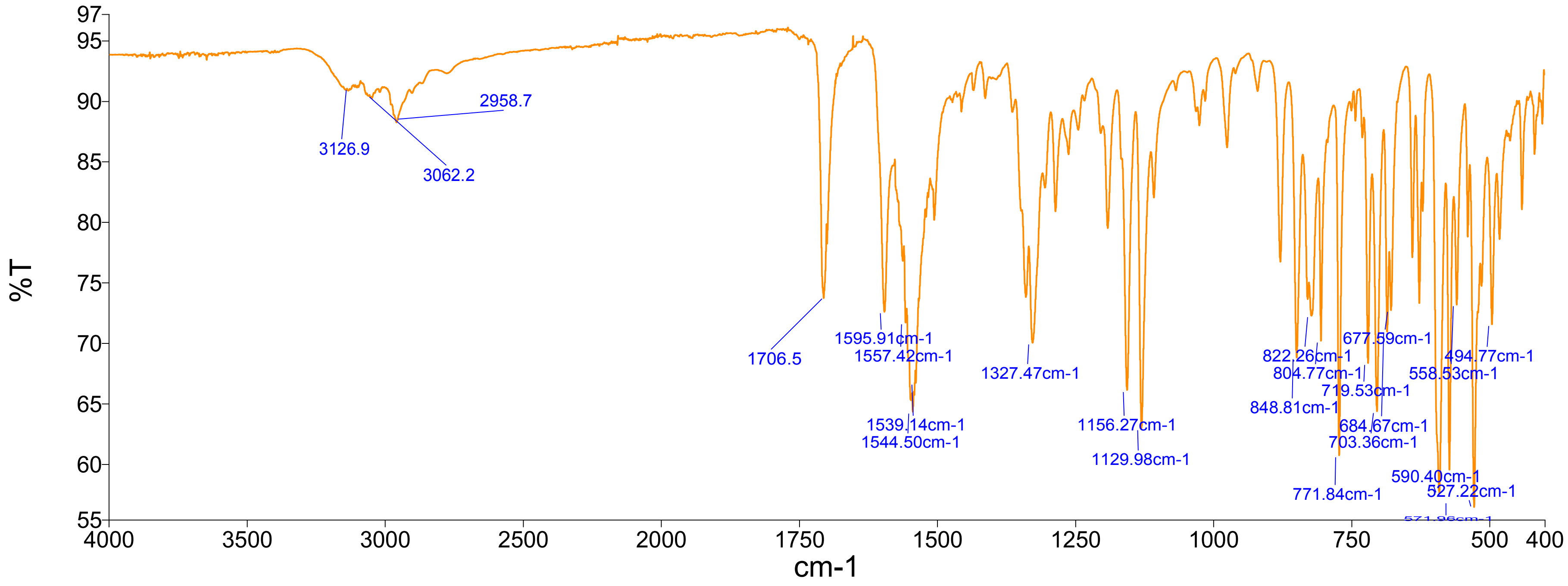

Sample Name	Description	Quality Checks
KP7118	Sample 251 By research Date Thursday, November 21 2019	The Quality Checks do not report any warnings for the sample.

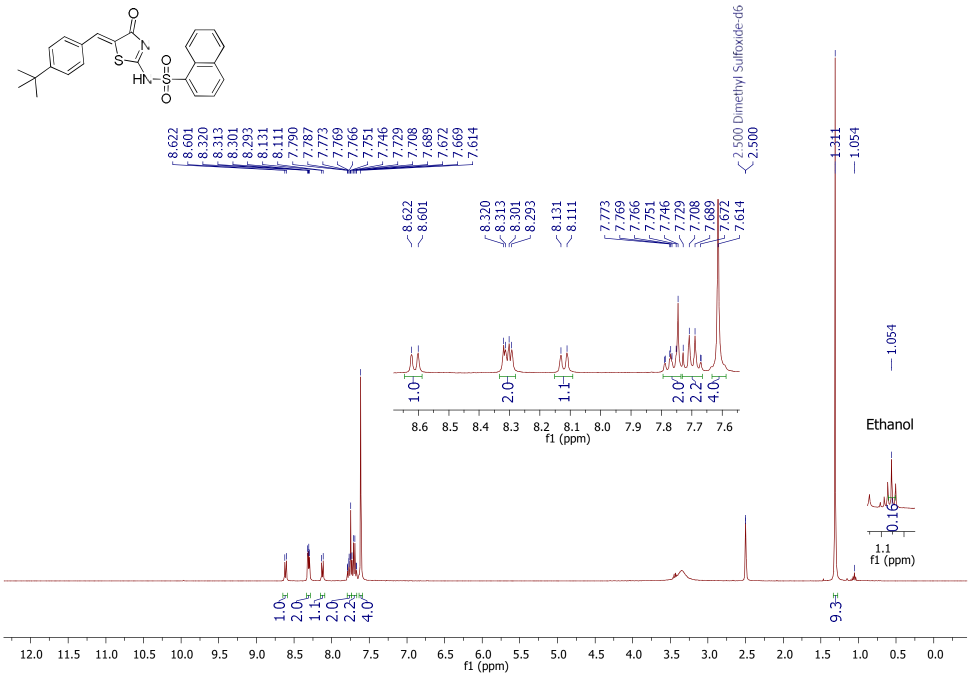

8.622
8.601
8.320
8.313
8.301
8.293
8.131
8.111
7.790
7.787
7.773
7.769
7.766
7.751
7.746
7.729
7.708
7.689
7.672
7.669
7.614
2.500 Dimethyl Sulfoxide-d6
2.500
1.311
1.054
Ethanol
f1 (ppm)

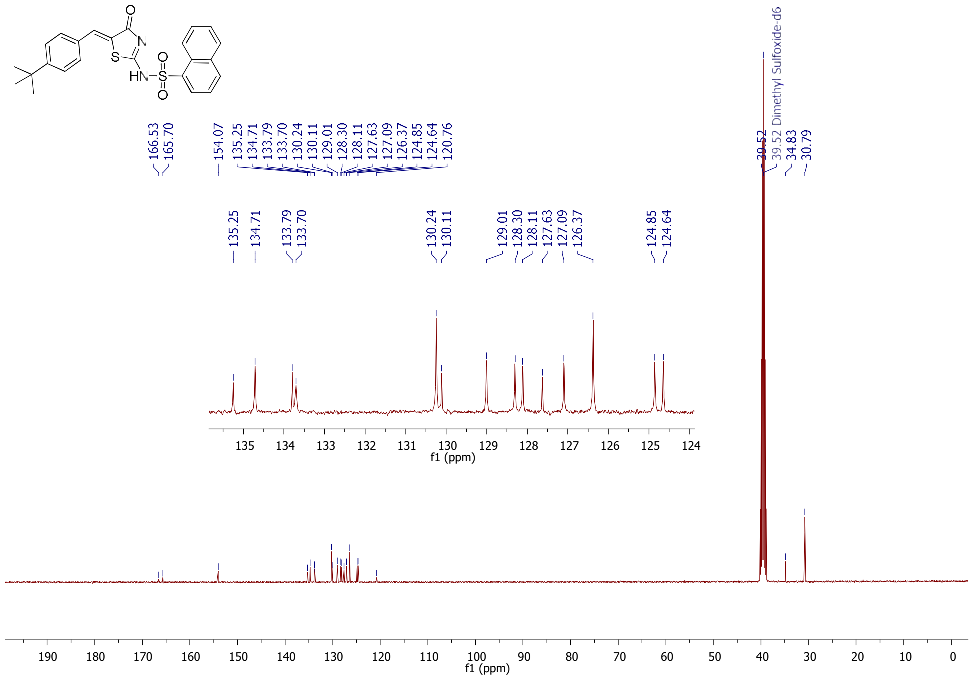

166.53
165.70
154.07
135.25
134.71
133.79
133.70
130.24
130.11
129.01
128.30
128.11
127.63
127.09
126.37
124.85
124.64
120.76
39.52 Dimethyl Sulfoxide-d6
34.83
30.79
f1 (ppm)

LCMS Report

Agilent Technologies

**Data file:**	D:\Chem32\1\Data\KP8117_80P 2019-11-15 14-03-17\002-39-KP7118_80.D		
**Sample name:**	KP7118_80		
**Description:**			
**Sample amount:**	0.000	**Sample type:**	Sample
**Instrument:**	LCMS	**Location:**	39
**Injection date:**	11/15/2019 2:09:33 PM	**Injection:**	1 of 1
**Acq. method:**	LCMS ISOCRATIC 80% B_3 MINS.M	**Injection volume:**	2.000
**Analysis method:**	LCMS ISOCRATIC 80%B_3 MINS.M	**Acq. operator:**	SYSTEM
**Last changed:**	8/6/2019 9:27:41 AM		

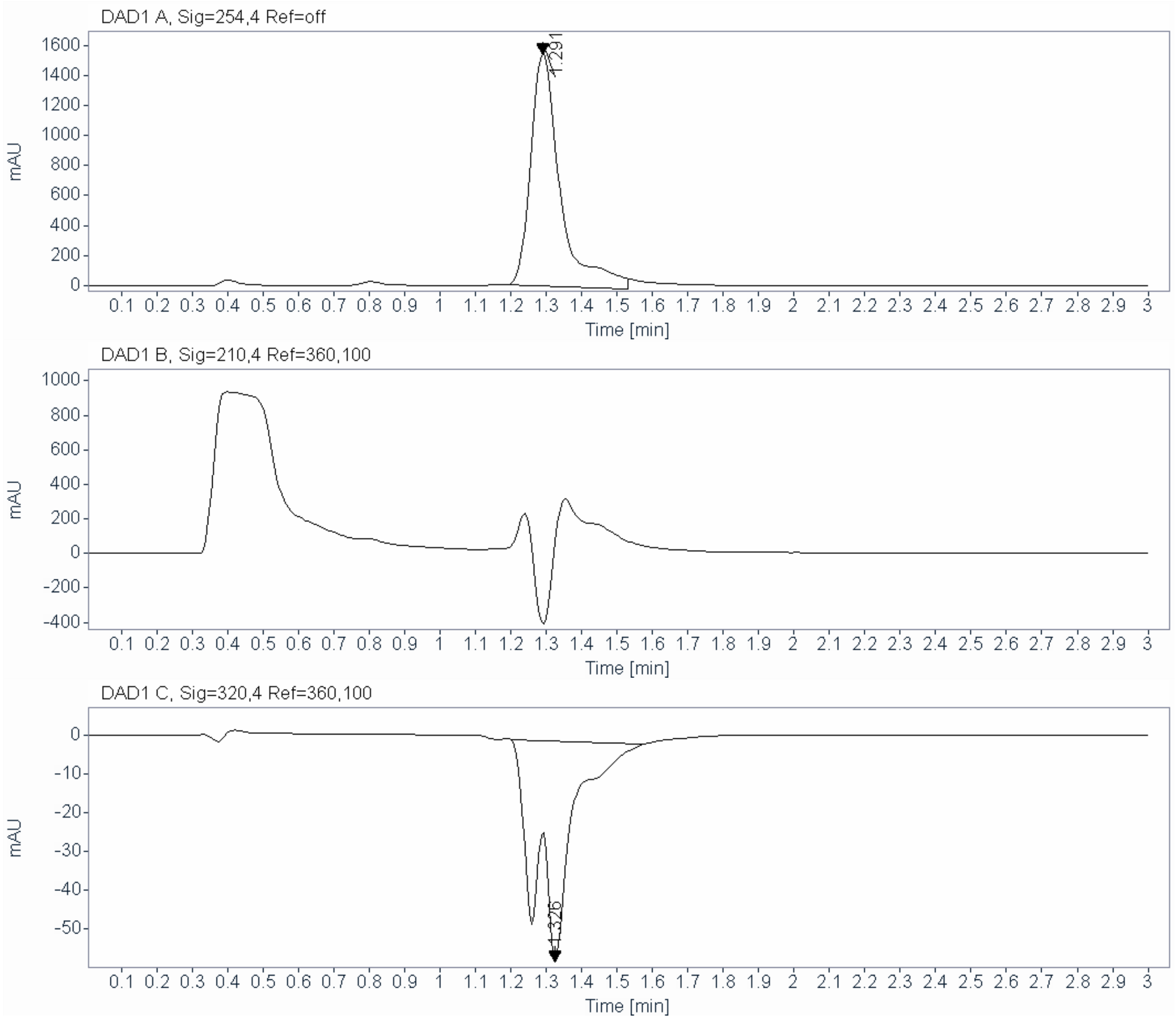

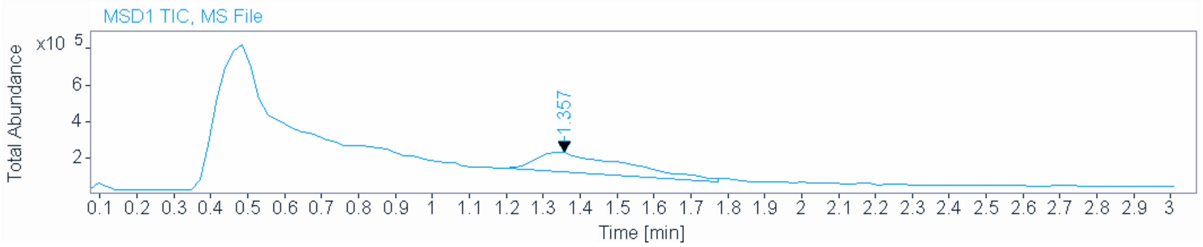

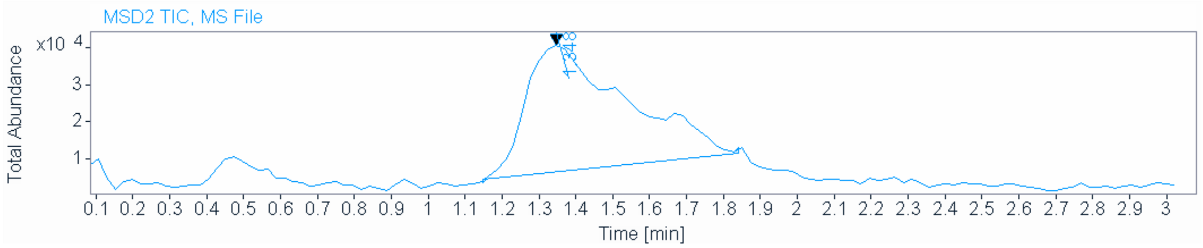

Signal: DAD1 A, Sig=254,4 Ref=off

RT [min]	Type	Width [min]	Area	Height	Area%	Name
1.291	MM	0.0970	9033.3594	1552.6115	100.0000	
		Sum	9033.3594			

Signal: DAD1 C, Sig=320,4 Ref=360,100

RT [min]	Type	Width [min]	Area	Height	Area%	Name
1.326	PM N	0.1180	404.6989	57.1711	100.0000	
		Sum	404.6989			

Signal: MSD1 TIC, MS File

RT [min]	Type	Width [min]	Area	Height	Area%	Name
1.357	MM	0.2962	1855290.125 0	104404.4844	100.0000	
		Sum	1855290.125			

Signal: MSD2 TIC, MS File

RT [min]	Type	Width [min]	Area	Height	Area%	Name
1.348	MM	0.3130	643367.6250	34256.6484	100.0000	
		Sum	643367.6250			

Agilent Technologies

1.348 - 1.346 (002-39-KP7118_80.D)

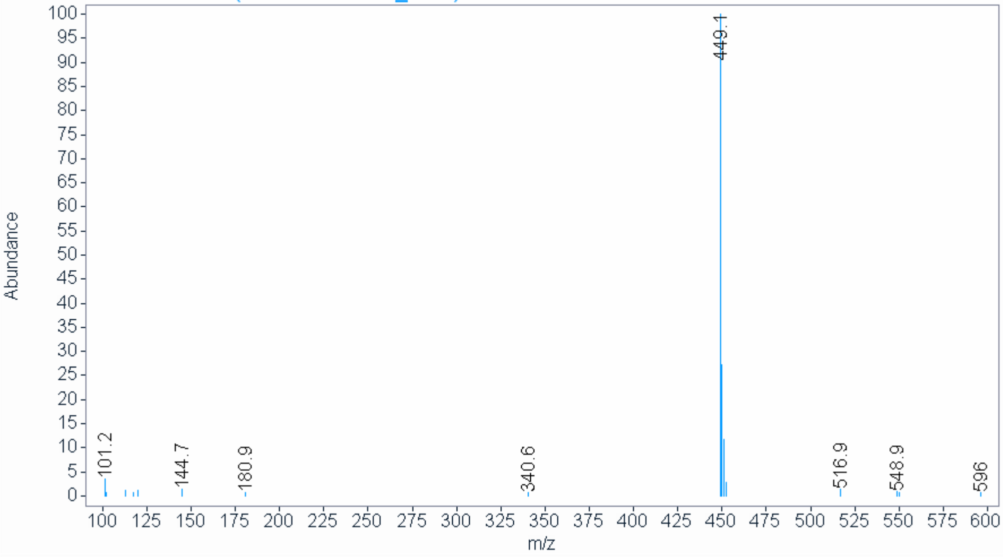

1.357 - 1.357 (002-39-KP7118_80.D)

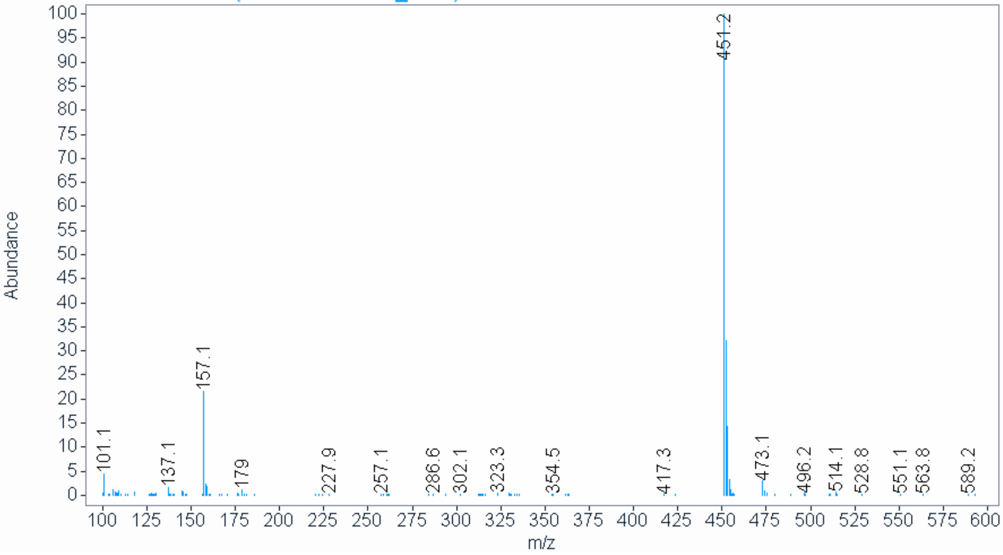

Compound Name: (Z)-*N*-(5-(4-isopropylbenzylidene)-4-oxo-4,5-dihydrothiazol-2-yl)naphthalene-1-sulfonamide

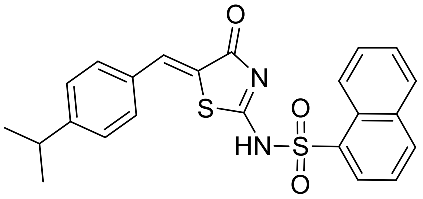

Chemical Formula: $C_{23}H_{20}N_2O_3S_2$
Exact Mass: 436.09
Molecular Weight: 436.55

Compound Code: 21 (KP7121)

Obtained Weight & Yield: 107 mg, 50%

Purity (by LCMS and ^{1}H NMR): >99% by ^{1}H-NMR, >99% by LCMS

Appearance: pale yellow solid

Solubility: DMSO, slightly soluble in acetone and methanol

Melting Point: 227 – 228 °C

TLC Rf (and conditions): N/A

IR Analysis (including assignment): IR (neat): 3153 (N-H), 3056 (C-H aromatic), 2972 (C-H), 1703 (C=O), 1539 (aromatic C-C), 1327 (sulfonamide), 1129 (C-N) cm^{-1}

^{1}H NMR Analysis: ^{1}H NMR (400 MHz, DMSO) δ 8.61 (d, *J* = 8.7 Hz, 1H), 8.32 – 8.29 (m, 2H), 8.12 (d, *J* = 8.0 Hz, 1H), 7.79 – 7.74 (m, 2H), 7.73 – 7.67 (m, 2H), 7.60 (d, *J* = 8.3 Hz, 2H), 7.47 (d, *J* = 8.3 Hz, 2H), 3.02 – 2.91 (m, 1H), 1.24 (s, 3H), 1.22 (s, 3H) ppm.
NH exchanging – not visible.

^{13}C NMR Analysis: ^{13}C NMR (101 MHz, DMSO) δ 166.5, 165.7, 151.9, 135.3, 134.7, 133.83, 133.80 130.51 (2C), 130.49, 129.0, 128.3, 128.1, 127.6, 127.5 (2C), 127.1, 124.9, 124.7, 120.6, 33.5, 23.5 (2C) ppm.

MS Analysis (low res): LRMS (ESI-) *m/z* (%): 435 (*M*-H, $C_{23}H_{19}N_2O_3S_2$, 100%); (ESI+) *m/z* (%): 437 (*M*+H, $C_{23}H_{21}N_2O_3S_2$, 100%);

MS Analysis (high res): Exact mass calculated for $C_{23}H_{19}N_2O_3S_2$ [*M*-H]$^-$, 435.0800. Found 435.0840.

HPLC method details: Column: Zorbax SB-C18 Rapid Resolution HT 2.1x50mm 1.8-Micron; Method: LCMS ISOCRATIC 60%B 0.4MLMIN-1.M filename: KP7121; Peak retention time: 4.199 mins; Area (%): 100

Procedure: To a 10 mL microwave vial was added *N*-(4-oxo-4,5-dihydrothiazol-2-yl)naphthalene-1-sulfonamide (147 mg, 0.49 mmol), 4-isopropylbenzaldehyde (0.08 mL, 0.54 mmol, 1.1 eq), ethanol (3 mL) and a catalytic amount of the benzoic acid/piperidine catalyst (approximately 5 drops). The suspension was heated using microwave irradiation (200 W, 120 °C) for 30 min. Precipitation occurred upon shaking of MW vial. The resulting precipitate was collected by vacuum filtration and washed with cold ethanol and cold ether to give the desired product (107 mg, 50%).

Other analyses, reference papers, previously obtained data, comments, etc:

PerkinElmer Spectrum Version 10.4.00
Thursday, 21 November 2019 11:39 AM

Analyst research
Date Thursday, 21 November 2019 11:39 AM

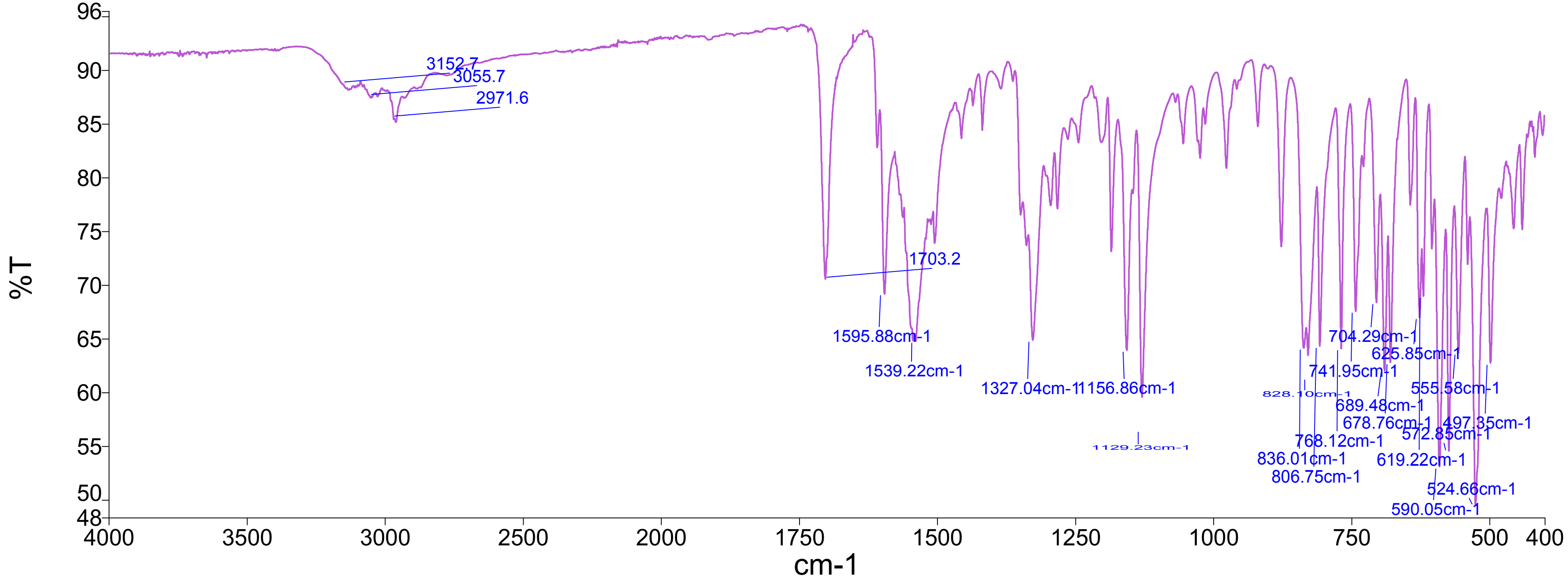

Sample Name	Description	Quality Checks
KP7121	Sample 252 By research Date Thursday, November 21 2019	The Quality Checks do not report any warnings for the sample.

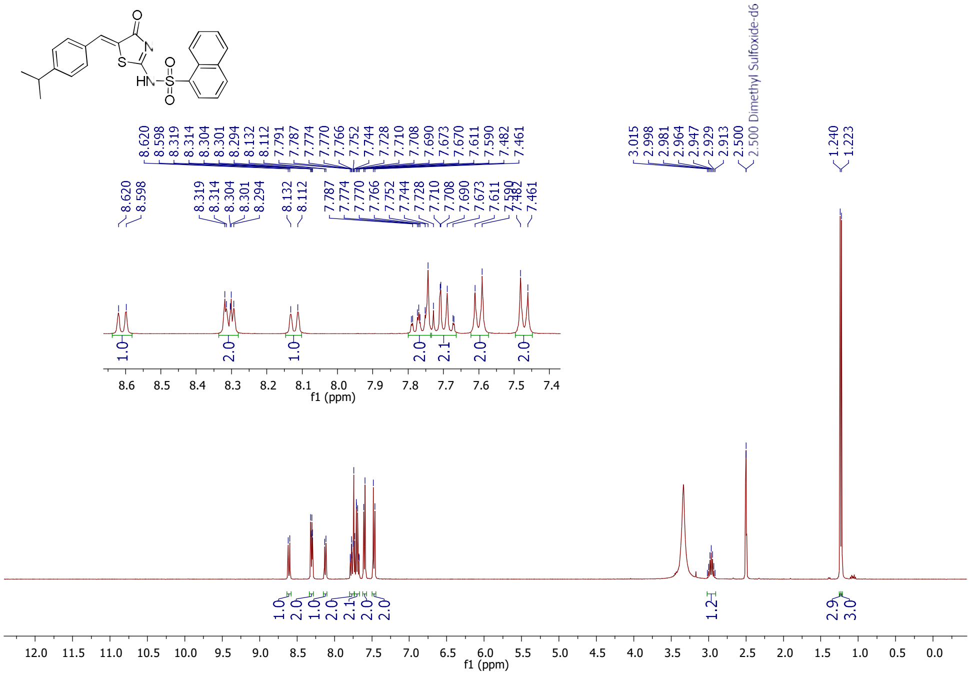

HN
O
S
N
8.620
8.598
8.319
8.314
8.304
8.301
8.294
8.132
8.112
7.791
7.787
7.774
7.770
7.766
7.752
7.744
7.728
7.710
7.708
7.690
7.673
7.670
7.611
7.590
7.482
7.461
3.015
2.998
2.981
2.964
2.947
2.929
2.913
2.500 Dimethyl Sulfoxide-d6
1.240
1.223
1.0
2.0
1.0
2.0
2.1
2.0
2.0
1.2
2.9
3.0
f1 (ppm)

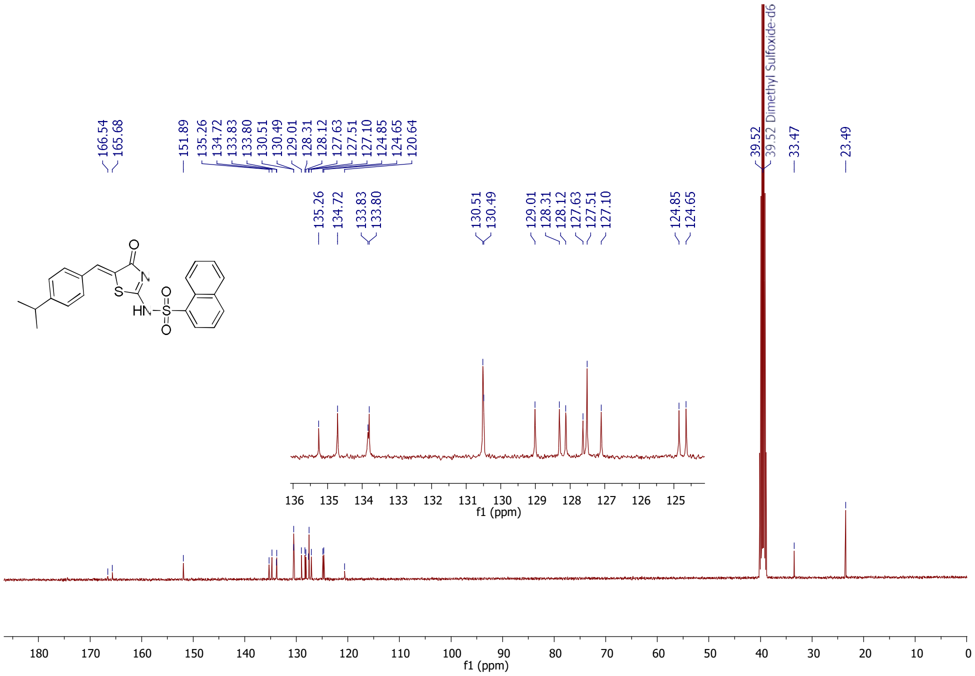

HN
O
S
N
166.54
165.68
151.89
135.26
134.72
133.83
133.80
130.51
130.49
129.01
128.31
128.12
127.63
127.51
127.10
124.85
124.65
120.64
39.52
39.52 Dimethyl Sulfoxide-d6
33.47
23.49
f1 (ppm)

LCMS Report

Agilent Technologies

Data file: D:\Chem32\1\Data\KP_DS_IND3 2019-11-15 12-19-28\003-40-KP7121.D
Sample name: KP7121
Description:
Sample amount: 0.000
Sample type: Sample

Instrument: LCMS
Location: 40
Injection date: 11/15/2019 12:36:25 PM
Injection: 1 of 1
Acq. method: LCMS ISOCRATIC 60% B 0.4MLMIN-1.M
Injection volume: 2.000
Analysis method: LCMS ISOCRATIC 60%B 0.4MLMIN-1.M
Acq. operator: SYSTEM
Last changed: 5/8/2019 8:55:04 AM

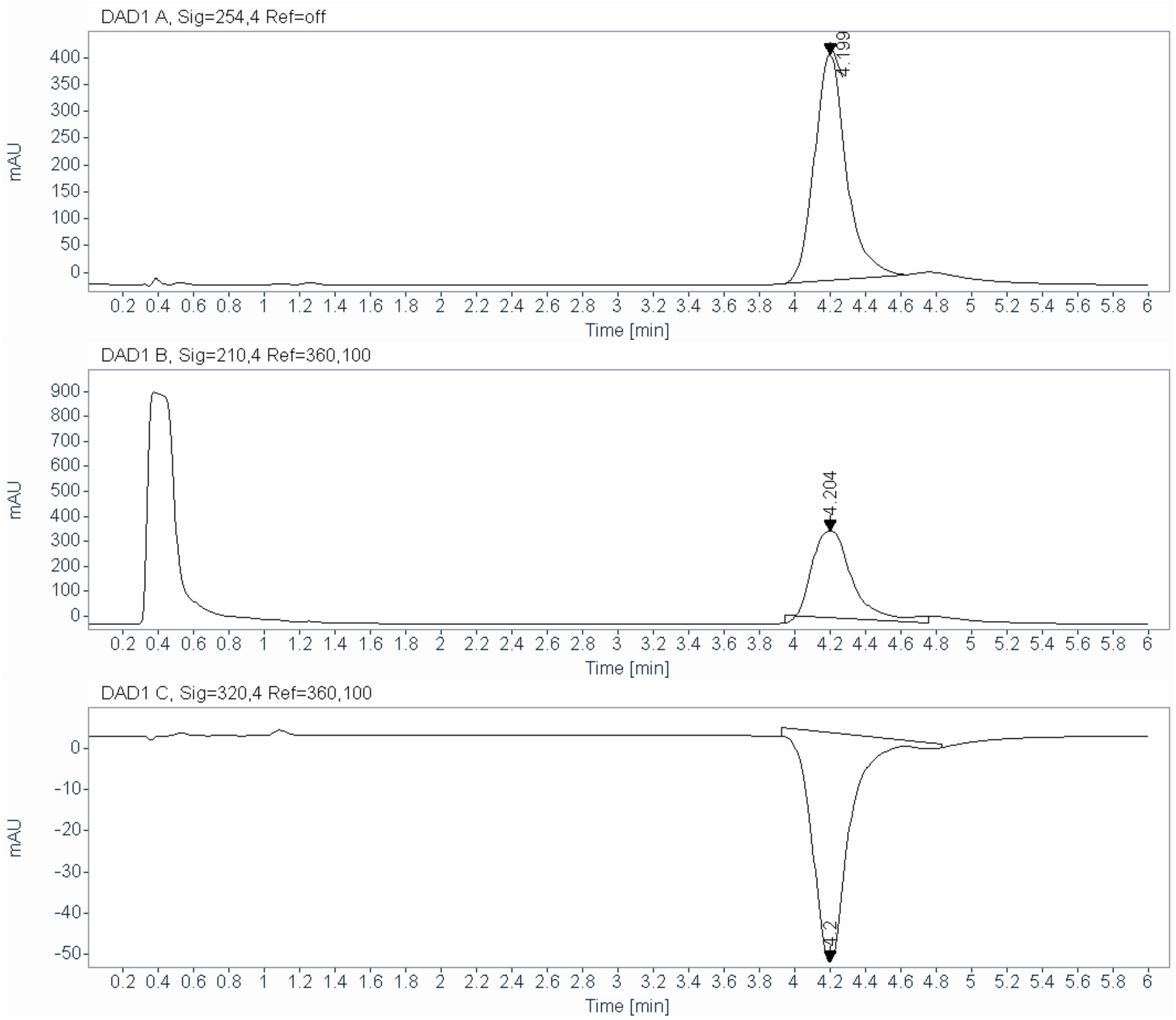

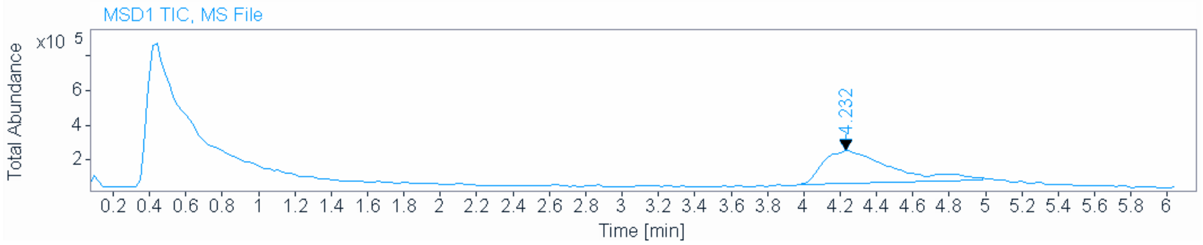

LCMS Report

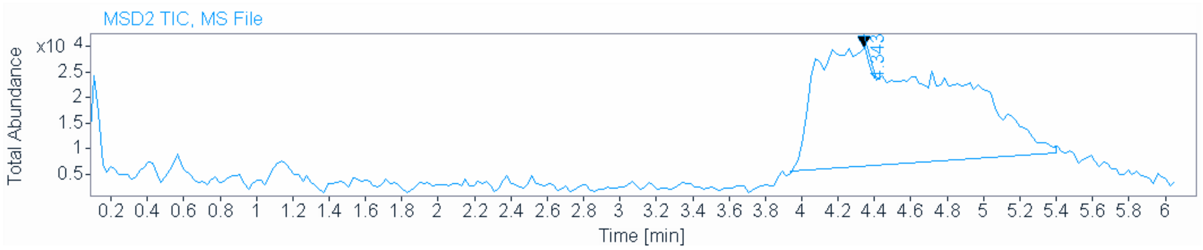

Signal: DAD1 A, Sig=254,4 Ref=off

RT [min]	Type	Width [min]	Area	Height	Area%	Name
4.199	BB	0.1902	5278.8403	421.3227	100.0000	
		Sum	5278.8403			

Signal: DAD1 B, Sig=210,4 Ref=360,100

RT [min]	Type	Width [min]	Area	Height	Area%	Name
4.204	MM	0.2660	5522.5527	345.9664	100.0000	
		Sum	5522.5527			

Signal: DAD1 C, Sig=320,4 Ref=360,100

RT [min]	Type	Width [min]	Area	Height	Area%	Name
4.200	MM N	0.2334	781.6418	55.8077	100.0000	
		Sum	781.6418			

Signal: MSD1 TIC, MS File

RT [min]	Type	Width [min]	Area	Height	Area%	Name
4.232	MM	0.4141	4792532.5000	192875.1406	100.0000	
		Sum	4792532.500			

Signal: MSD2 TIC, MS File

RT [min]	Type	Width [min]	Area	Height	Area%	Name
4.343	MM	0.8410	1189043.0000	23563.2266	100.0000	
		Sum	1189043.000			

Agilent Technologies

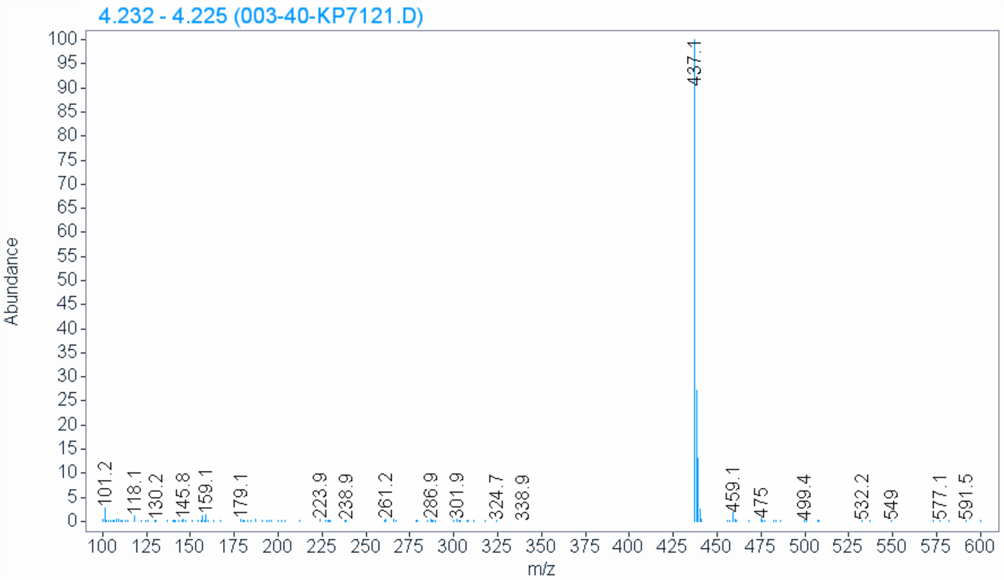

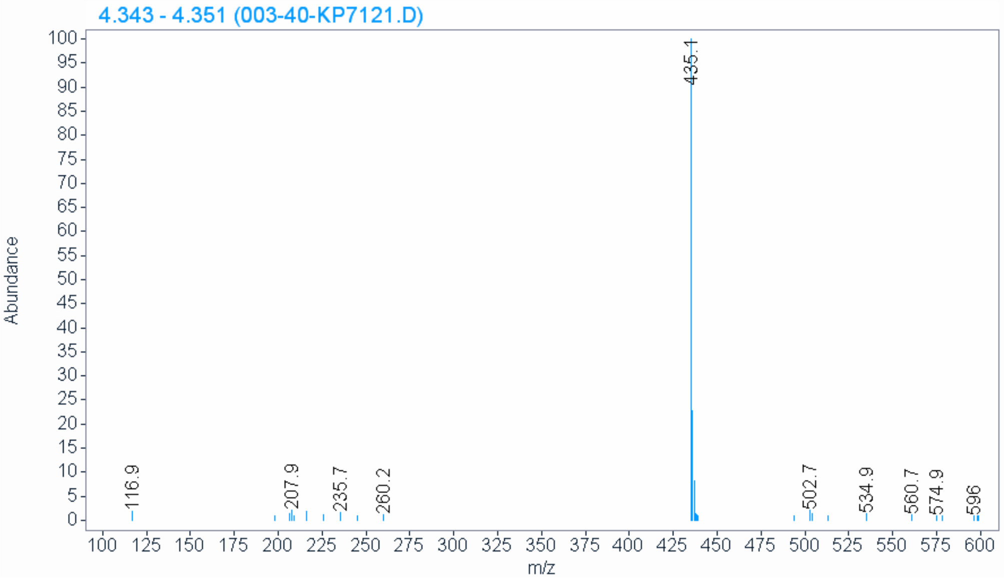

Compound Name: (Z)-*N*-(4-oxo-5-(4-propylbenzylidene)-4,5-dihydrothiazol-2-yl)naphthalene-1-sulfonamide

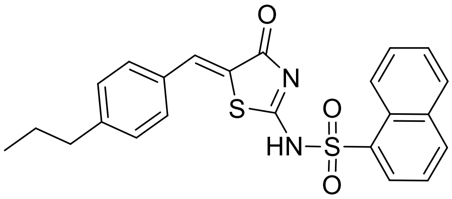

Chemical Formula: $C_{23}H_{20}N_2O_3S_2$
Exact Mass: 436.09
Molecular Weight: 436.55

Compound Code: 22 (KP7124)

Obtained Weight & Yield: 71 mg, 49%

Purity (by LCMS and ^{1}H NMR): > 99% by ^{1}H-NMR, > 99% by LCMS

Appearance: pale orange solid

Solubility: DMSO, slightly soluble in acetone and methanol

Melting Point: 209 – 211 °C

TLC Rf (and conditions): N/A

IR Analysis (including assignment): IR (neat): 2972 (C-H aromatic), 2797 (C-H), 1701 (C=O), 1551 (aromatic C-C), 1328 (sulfonamide), 1130 (C-N) cm^{-1}

^{1}H NMR Analysis: ^{1}H NMR (400 MHz, DMSO) δ 8.62 (d, *J* = 8.7 Hz, 1H), 8.31 (dt, *J* = 8.0, 4.0 Hz, 2H), 8.12 (d, *J* = 8.1 Hz, 1H), 7.77 (ddd, *J* = 8.5, 6.9, 1.4 Hz, 1H), 7.73 – 7.67 (m, 3H), 7.57 (d, *J* = 8.2 Hz, 2H), 7.39 (d, *J* = 8.2 Hz, 2H), 2.63 – 2.59 (m, 2H), 1.65 – 1.56 (m, 2H), 0.90 (t, *J* = 7.3 Hz, 3H) ppm.
NH exchanging – not visible.
Ether impurity at 1.09 ppm (0.37%)

^{13}C NMR Analysis: ^{13}C NMR (101 MHz, DMSO) δ 166.6, 165.7, 145.8, 135.3, 134.7, 133.8 (2C), 130.39 (2C), 130.35, 129.5 (2C), 129.0, 128.3, 128.1, 127.6, 127.1, 124.9, 124.6, 120.6, 37.1, 23.7, 13.6 ppm.
2C determined by 2D NMR and 2C at 133.8 determined by DEPTQ.

MS Analysis (low res): LRMS (ESI-) *m/z* (%): 435 (*M*-H, $C_{23}H_{19}N_2O_3S_2$, 100%); (ESI+) *m/z* (%): 437 (*M*+H, $C_{23}H_{21}N_2O_3S_2$, 100%)

MS Analysis (high res): Exact mass calculated for $C_{23}H_{19}N_2O_3S_2$ [*M*-H]$^-$, 435.0800. Found 435.0840.

HPLC method details: Column: Zorbax SB-C18 Rapid Resolution HT 2.1x50mm 1.8-Micron; Method: LCMS ISOCRATIC 60%B 0.4MLMIN-1.M filename: KP7124; Peak retention time: 4.572 mins; Area (%): 100

Procedure: To a 10 mL microwave vial was added the *N*-(4-oxo-4,5-dihydrothiazol-2-yl)naphthalene-1-sulfonamide (113 mg, 0.33 mmol), 4-propylbenzaldehyde (0.05 mL, 0.36 mmol, 1.1 eq), ethanol (3 mL) and a catalytic amount of the benzoic acid/piperidine catalyst (approximately 5 drops). The suspension was heated using microwave irradiation (200 W, 120 °C) for 30 min. Precipitation occurred upon shaking of MW vial. The resulting precipitate was collected by vacuum filtration and washed with cold ethanol and cold ether to give the desired product (71 mg, 49%).

Other analyses, reference papers, previously obtained data, comments, etc:

PerkinElmer Spectrum Version 10.4.00
Thursday, 21 November 2019 11:39 AM

Analyst	research
Date	Thursday, 21 November 2019 11:39 AM

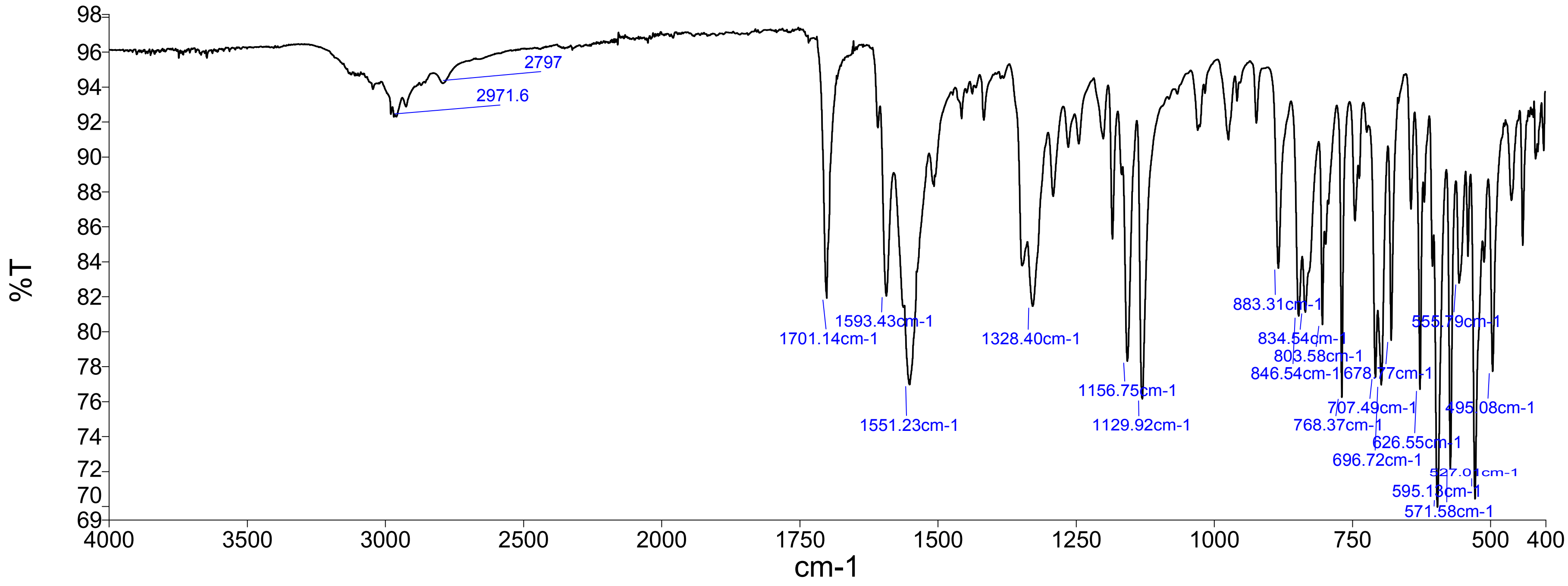

Sample Name	Description	Quality Checks
KP7124	Sample 253 By research Date Thursday, November 21 2019	The Quality Checks give rise to a Weak Bands warning for the sample.

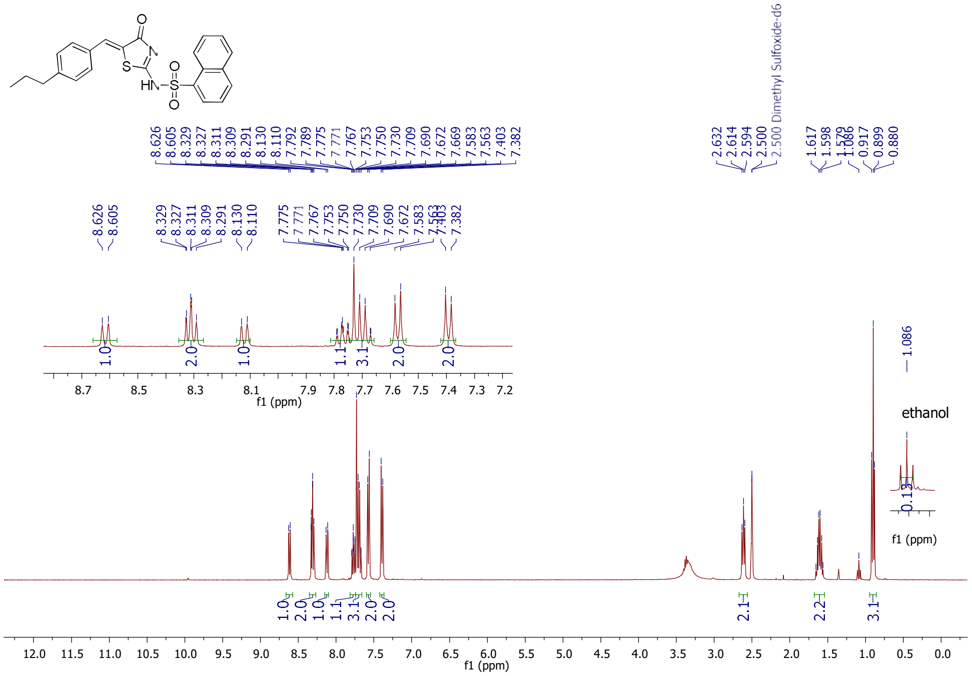
HN
S
O
N
8.626
8.605
8.329
8.327
8.311
8.309
8.291
8.130
8.110
7.792
7.789
7.775
7.771
7.767
7.753
7.750
7.730
7.709
7.690
7.672
7.669
7.583
7.563
7.403
7.382
2.632
2.614
2.594
2.500 Dimethyl Sulfoxide-d6
1.617
1.598
1.579
1.086
0.917
0.899
0.880
ethanol
f1 (ppm)

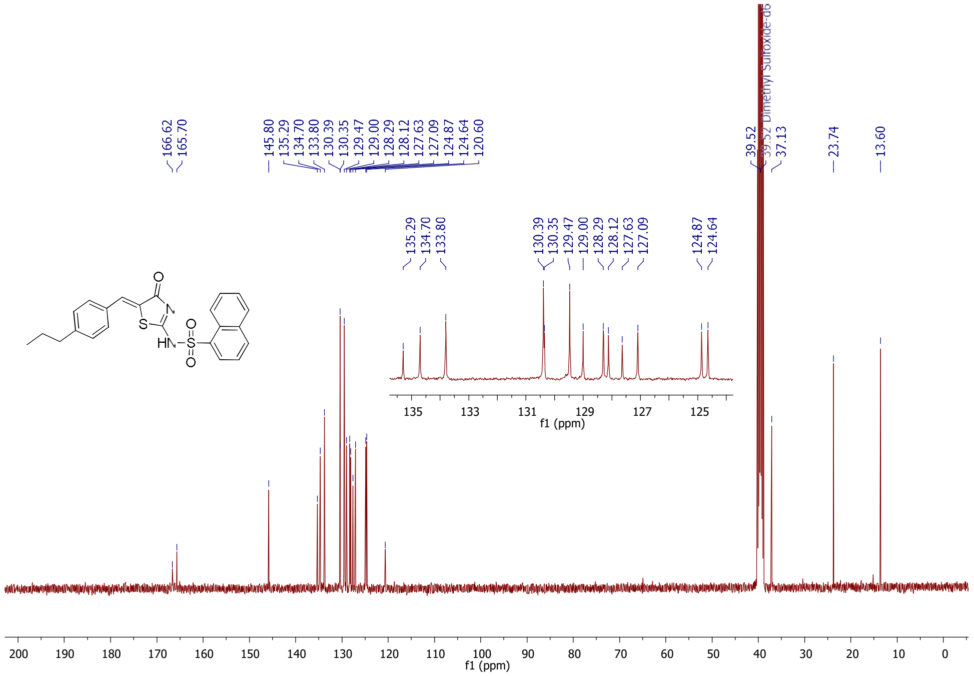
HN
S
O
N
166.62
165.70
145.80
135.29
134.70
133.80
130.39
130.35
129.47
129.00
128.29
128.12
127.63
127.09
124.87
124.64
120.60
39.52
39.52 Dimethyl Sulfoxide-d6
37.13
23.74
13.60
f1 (ppm)

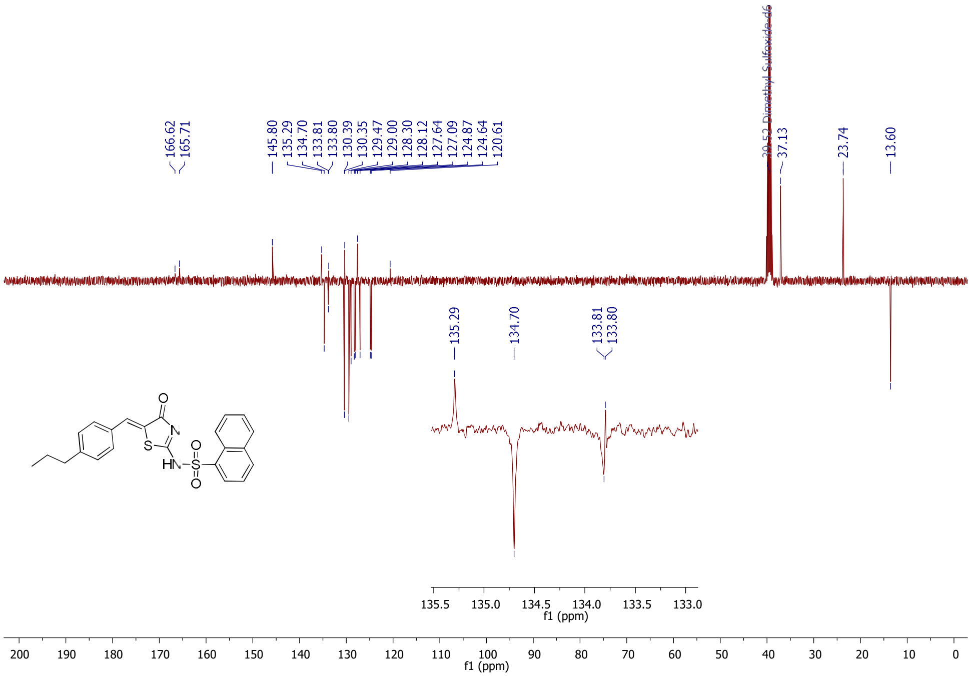

166.62
165.71
145.80
135.29
134.70
133.81
133.80
130.39
130.35
129.47
129.00
128.30
128.12
127.64
127.09
124.87
124.64
120.61
39.52 Dimethyl Sulfoxide-d6
37.13
23.74
13.60
135.29
134.70
133.81
133.80
135.5 135.0 134.5 134.0 133.5 133.0
f1 (ppm)
200 190 180 170 160 150 140 130 120 110 100 90 80 70 60 50 40 30 20 10 0
f1 (ppm)

LCMS Report

Agilent Technologies

Data file: D:\Chem32\1\Data\KP_DS_IND3 2019-11-15 12-19-28\004-50-KP7124.D
Sample name: KP7124
Description:
Sample amount: 0.000
Sample type: Sample

Instrument: LCMS
Injection date: 11/15/2019 12:44:04 PM
Acq. method: LCMS ISOCRATIC 60% B 0.4MLMIN-1.M
Analysis method: LCMS ISOCRATIC 60%B 0.4MLMIN-1.M
Last changed: 5/8/2019 8:55:04 AM
Location: 50
Injection: 1 of 1
Injection volume: 2.000
Acq. operator: SYSTEM

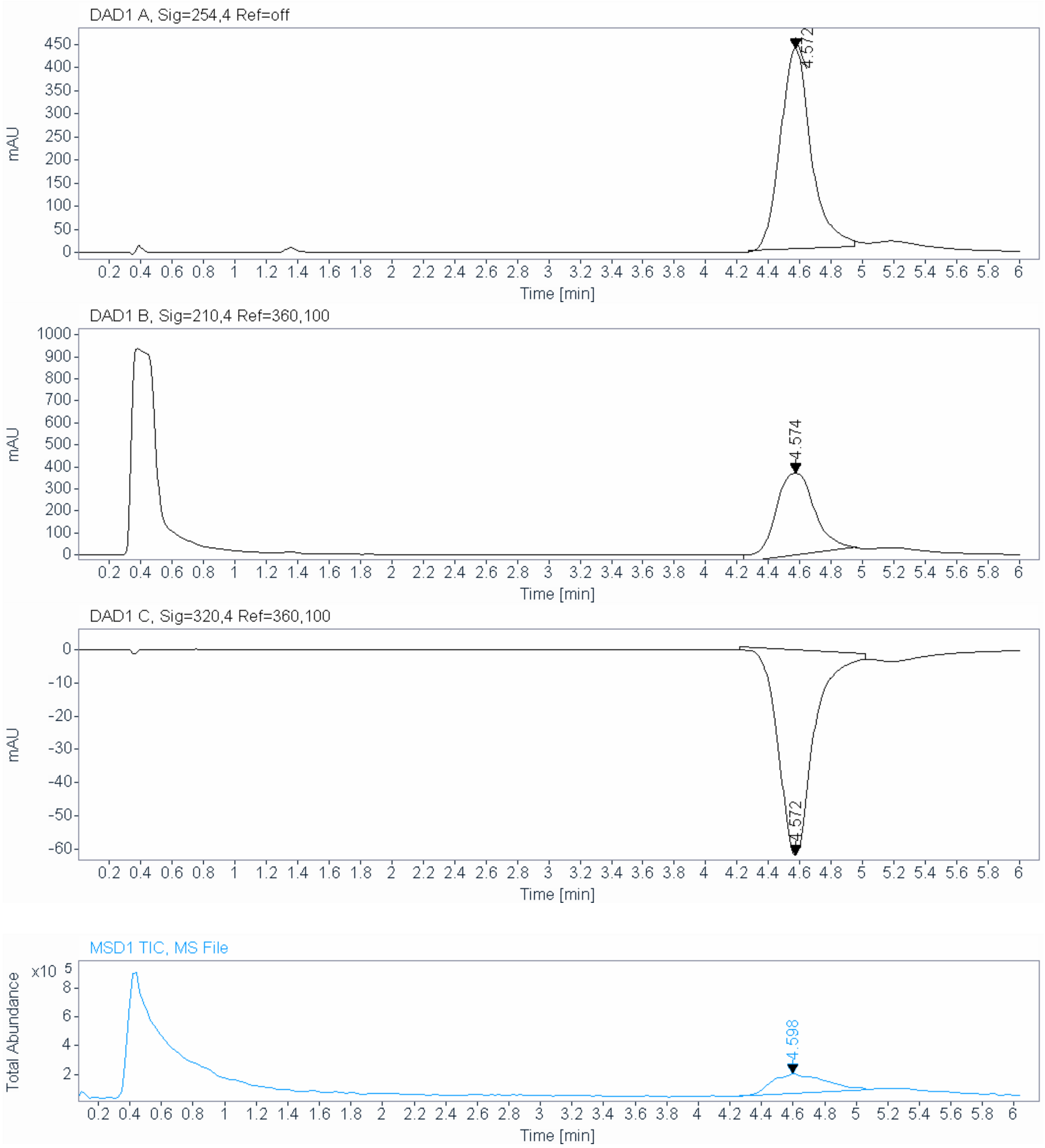

Agilent Technologies

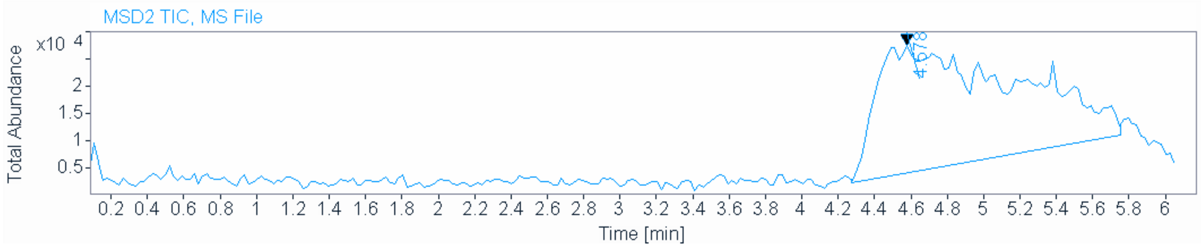

Signal: DAD1 A, Sig=254,4 Ref=off

RT [min]	Type	Width [min]	Area	Height	Area%	Name
4.572	MM	0.2264	5901.2754	434.4597	100.0000	
		Sum	5901.2754			

Signal: DAD1 B, Sig=210,4 Ref=360,100

RT [min]	Type	Width [min]	Area	Height	Area%	Name
4.574	MM	0.2909	6462.2544	370.2687	100.0000	
		Sum	6462.2544			

Signal: DAD1 C, Sig=320,4 Ref=360,100

RT [min]	Type	Width [min]	Area	Height	Area%	Name
4.572	MM N	0.2413	899.3729	62.1175	100.0000	
		Sum	899.3729			

Signal: MSD1 TIC, MS File

RT [min]	Type	Width [min]	Area	Height	Area%	Name
4.598	BB	0.3081	3182796.250<br>0	139189.8125	100.0000	
		Sum	3182796.250			

Signal: MSD2 TIC, MS File

RT [min]	Type	Width [min]	Area	Height	Area%	Name
4.578	MM	0.8501	1201552.375<br>0	23556.8164	100.0000	
		Sum	1201552.375			

Agilent Technologies

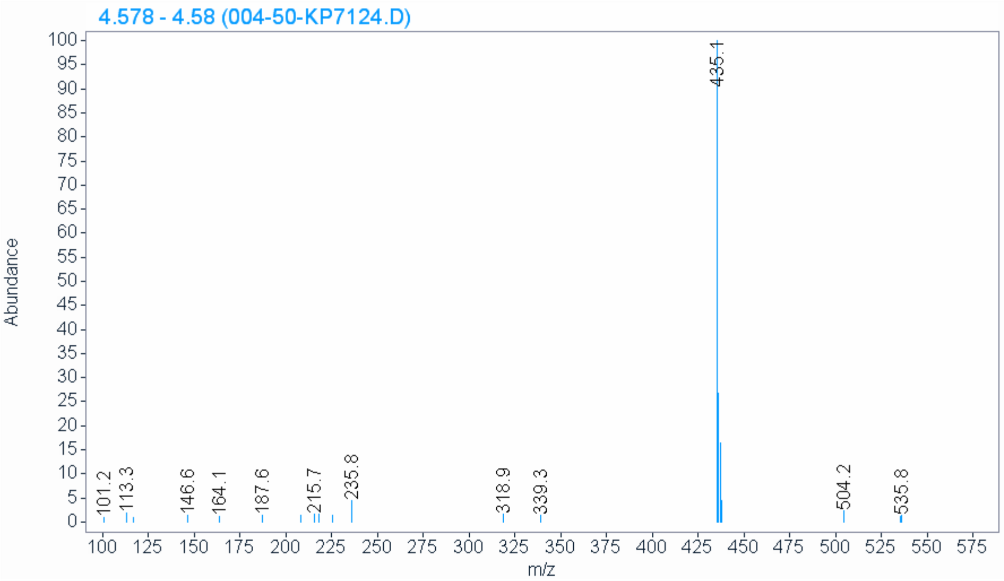

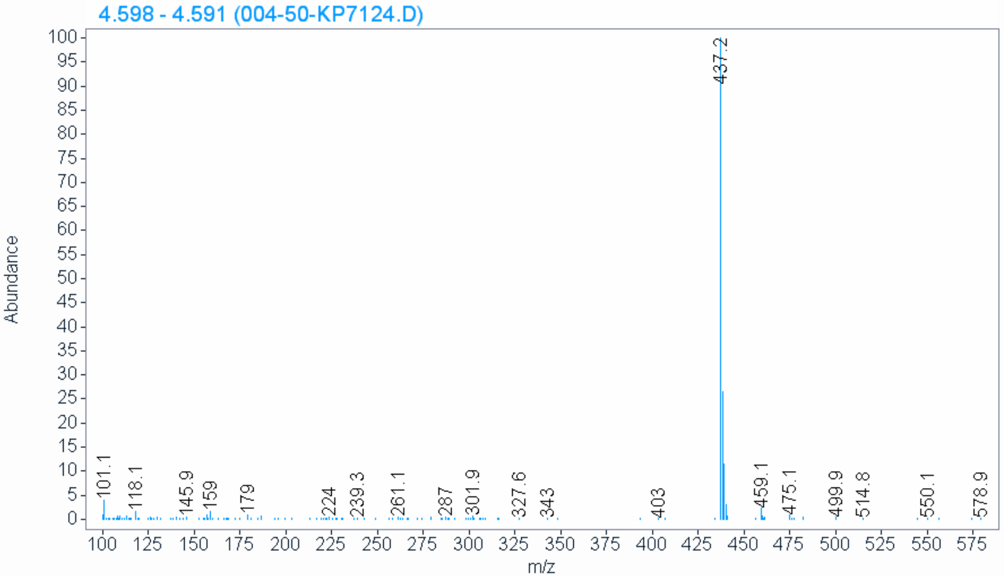

Compound Name: (Z)-*N*-(5-(4-butylbenzylidene)-4-oxo-4,5-dihydrothiazol-2-yl)naphthalene-1-sulfonamide

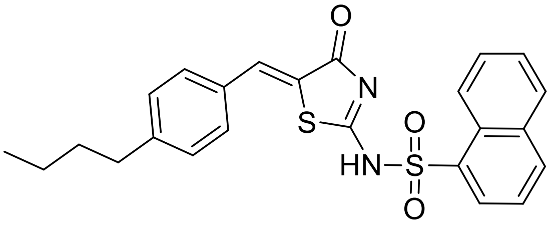

Chemical Formula: $C_{24}H_{22}N_2O_3S_2$
Exact Mass: 450.11
Molecular Weight: 450.57

Compound Code: 23 (KP7125)

Obtained Weight & Yield: 106 mg, 71%

Purity (by LCMS and ^{1}H NMR): > 99% by ^{1}H-NMR, > 99% by LCMS

Appearance: off white solid

Solubility: DMSO, slightly soluble in acetone and methanol

Melting Point: 214 – 216 °C

TLC Rf (and conditions): N/A

IR Analysis (including assignment): IR (neat): 3120 (NH), 2933 (C-H aromatic), 2784 (C-H), 1729 (C=O), 1554 (aromatic C-C), 1324 (sulfonamide), 1123 (C-N) cm^{-1}

^{1}H NMR Analysis: ^{1}H NMR (400 MHz, DMSO) δ 13.13 (s, 1H, NH), 8.62 (d, *J* = 8.6 Hz, 1H), 8.31 (t, *J* = 7.9 Hz, 2H), 8.12 (d, *J* = 8.1 Hz, 1H), 7.77 (t, *J* = 7.6 Hz, 1H), 7.70 (dd, *J* = 15.1, 7.3 Hz, 3H), 7.56 (d, *J* = 8.0 Hz, 2H), 7.38 (d, *J* = 8.0 Hz, 2H), 2.63 (t, *J* = 7.6 Hz, 2H), 1.60 – 1.52 (m, 2H), 1.35 – 1.26 (m, 2H), 0.90 (t, *J* = 7.3 Hz, 3H) ppm.
Ether impurity at 1.08 ppm (0.57%)

^{13}C NMR Analysis: ^{13}C NMR (101 MHz, DMSO) δ 166.6, 165.7, 146.0, 135.3, 134.7, 133.8, 133.8, 130.4 (2C), 130.3, 129.4 (2C), 129.0, 128.3, 128.1, 127.6, 127.1, 124.8, 124.6, 120.5, 34.8, 32.7, 21.7, 13.7 ppm.

MS Analysis (low res): LRMS (ESI-) *m/z* (%): 449 (*M*-H, $C_{24}H_2N_2O_3S_2$, 100); LRMS (ESI+) *m/z* (%): 451 (*M*+H, $C_{24}H_{23}N_2O_3S_2$, 100)

MS Analysis (high res): Exact mass calculated for $C_{24}H_{20}N_2O_3S_2$ [*M*-H]$^-$, 449.1000. Found 449.0998.

HPLC method details: Column: Zorbax SB-C18 Rapid Resolution HT 2.1x50mm 1.8-Micron; Method: LCMS ISOCRATIC_80%B_0.4MLMIN-1_5MINS.M filename: KP7125; Peak retention time: 1.500 mins; Area (%): 100

Procedure: To a 10 mL microwave vial was added the *N*-(4-oxo-4,5-dihydrothiazol-2-yl)naphthalene-1-sulfonamide (108 mg, 0.33 mmol), 4-propylbenzaldehyde (0.06 mL, 0.36 mmol, 1.1 eq), ethanol (3 mL) and a catalytic amount of the benzoic acid/piperidine catalyst (approximately 5 drops). The suspension was heated using microwave irradiation (200 W, 120 °C) for 30 min. Precipitation occurred upon shaking of MW vial. The resulting precipitate was collected by vacuum filtration and washed with cold ethanol and cold ether to give the desired product (106 mg, 71%).

Other analyses, reference papers, previously obtained data, comments, etc:

PerkinElmer Spectrum Version 10.4.00
Thursday, 21 November 2019 11:39 AM

Analyst	research
Date	Thursday, 21 November 2019 11:39 AM

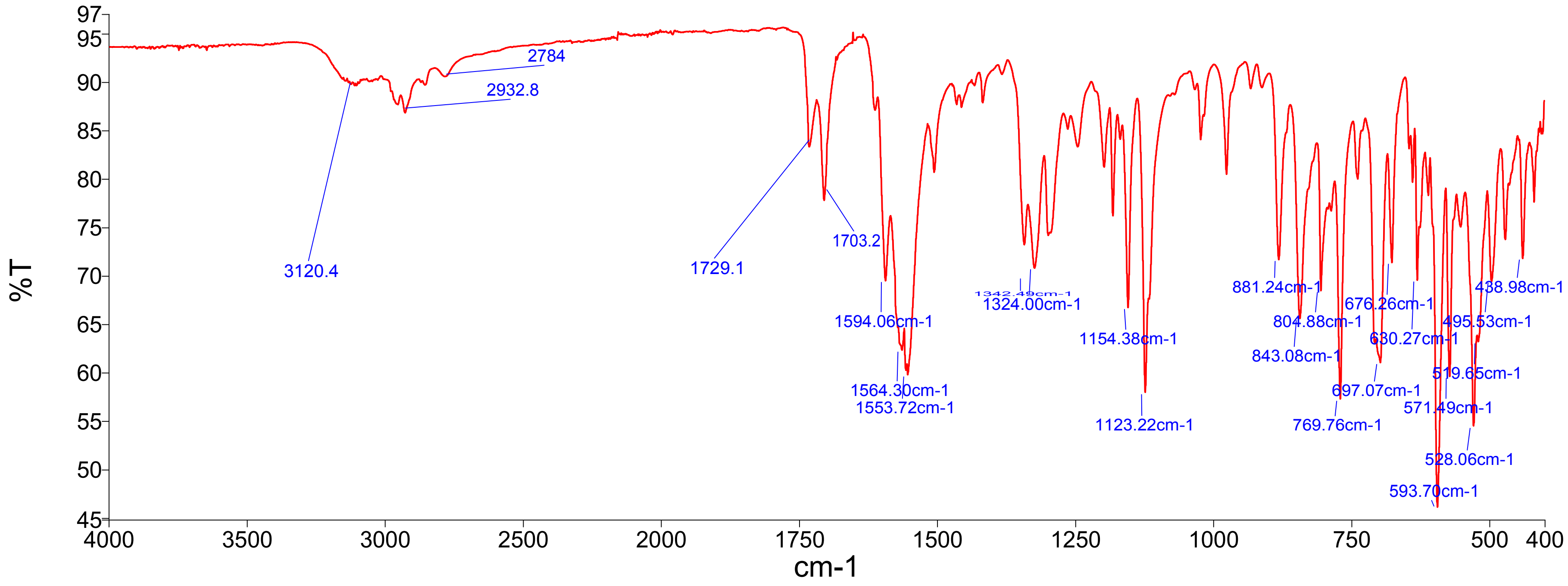

Sample Name	Description	Quality Checks
KP7125	Sample 254 By research Date Thursday, November 21 2019	The Quality Checks do not report any warnings for the sample.

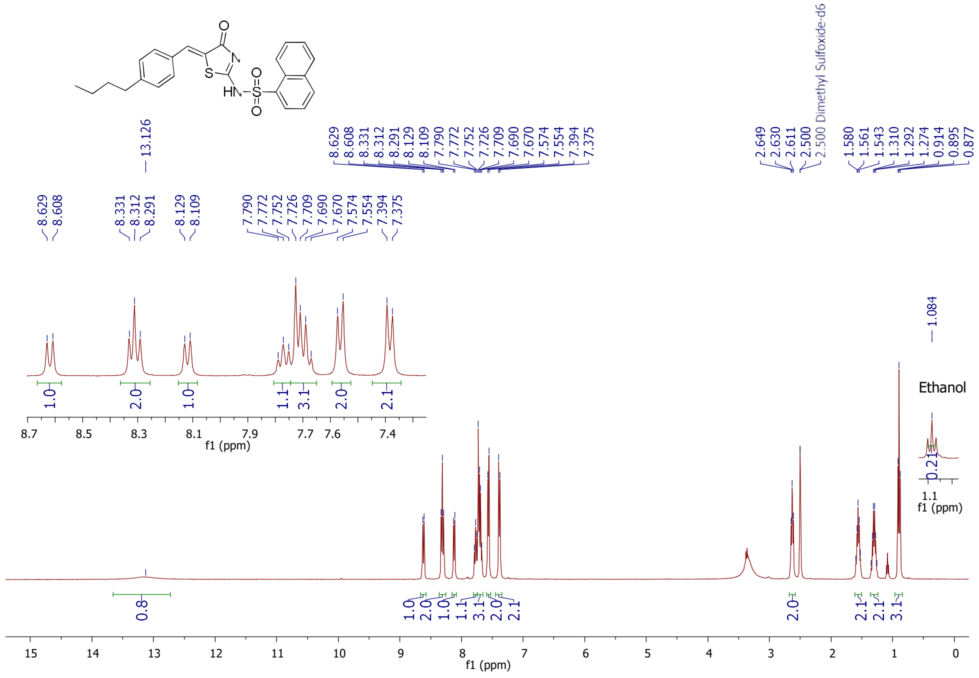

HN
S
O
13.126
8.629 8.608 8.331 8.312 8.291 8.129 8.109 7.790 7.772 7.752 7.726 7.709 7.690 7.670 7.574 7.554 7.394 7.375
2.649 2.630 2.611 2.500 2.500 Dimethyl Sulfoxide-d6
1.580 1.561 1.543 1.310 1.292 1.274 0.914 0.895 0.877
1.084
Ethanol
0.8
1.0 2.0 1.0 1.1 3.1 2.0 2.1
2.0
2.1 2.1 3.1
0.21
f1 (ppm)

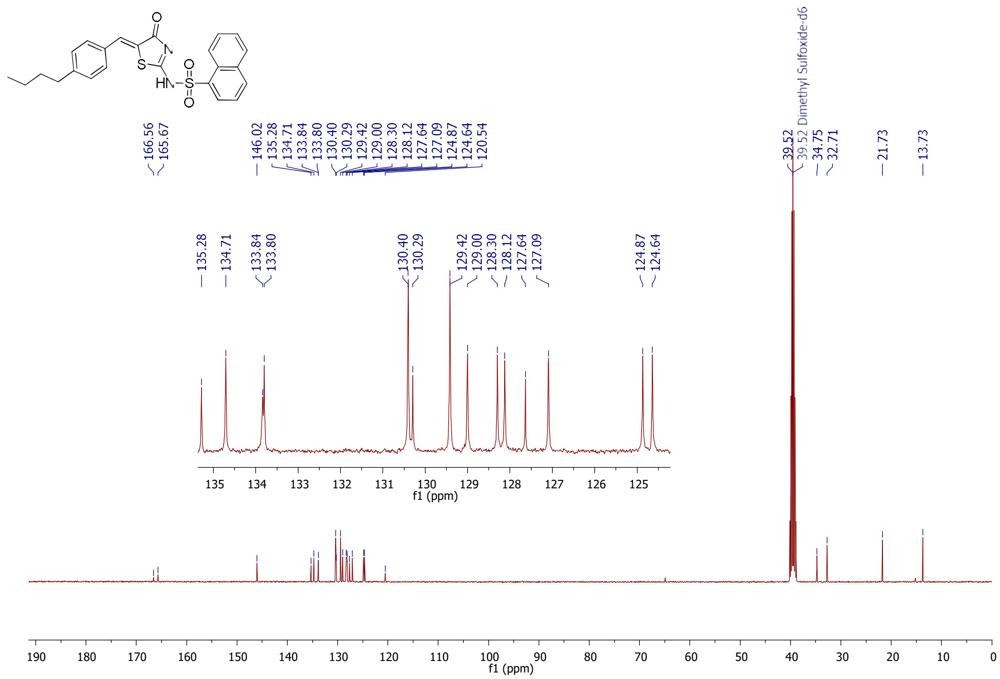

HN
S
O
166.56 165.67
146.02 135.28 134.71 133.84 133.80 130.40 130.29 129.42 129.00 128.30 128.12 127.64 127.09 124.87 124.64 120.54
39.52 39.52 Dimethyl Sulfoxide-d6
34.75 32.71
21.73
13.73
f1 (ppm)

LCMS Report

**Data file:**	D:\Chem32\1\Data\KP\KP7125_NEW 2020-07-13 11-30-13\002-31-KP7125.D		
**Sample name:**	KP7125		
**Description:**			
**Sample amount:**	0.000	**Sample type:**	Sample
**Instrument:**	LCMS	**Location:**	31
**Injection date:**	7/13/2020 11:38:23 AM	**Injection:**	1 of 1
**Acq. method:**	LCMS ISOCRATIC_80% B_0.4MLMIN-1_5MINS.M	**Injection volume:**	2.000
**Analysis method:**	LCMS ISOCRATIC_80% B_0.4MLMIN-1_5MINS.M	**Acq. operator:**	SYSTEM
**Last changed:**	7/8/2019 11:58:37 AM		

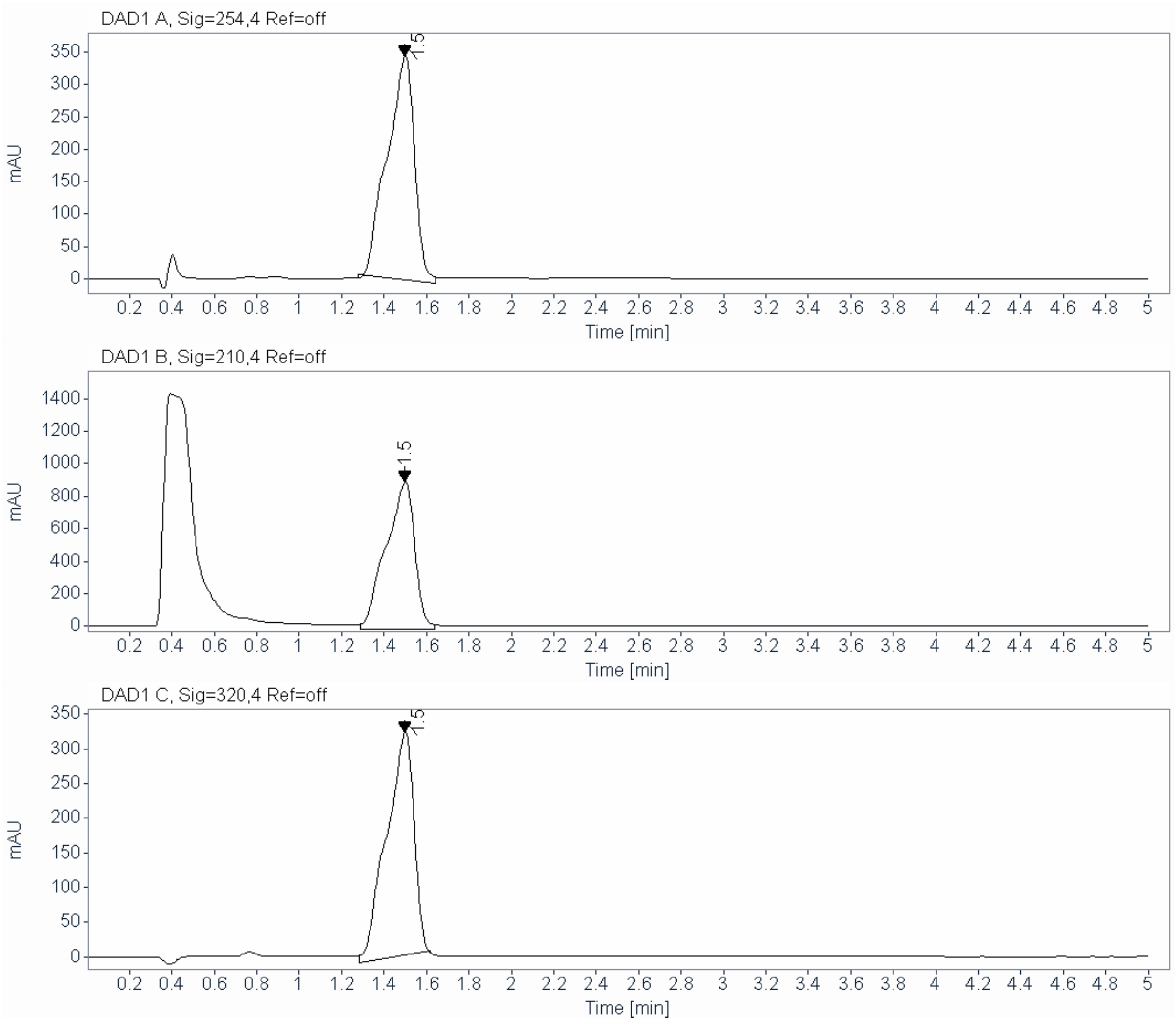

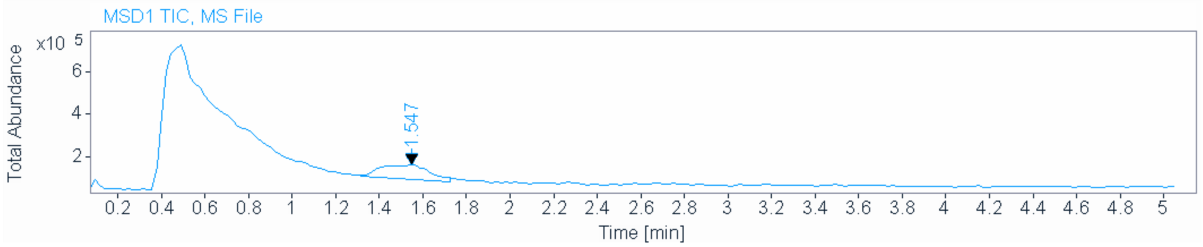

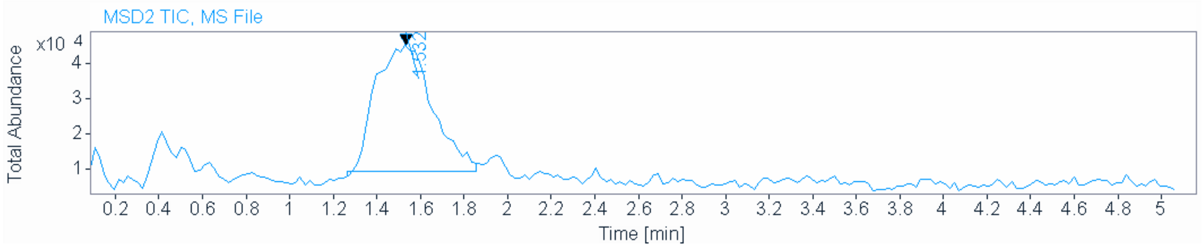

Signal: DAD1 A, Sig=254,4 Ref=off

RT [min]	Type	Width [min]	Area	Height	Area%	Name
1.500	MM	0.1489	3093.9614	346.2611	100.0000	
		Sum	3093.9614			

Signal: DAD1 B, Sig=210,4 Ref=off

RT [min]	Type	Width [min]	Area	Height	Area%	Name
1.500	MM	0.1573	8573.0439	908.2371	100.0000	
		Sum	8573.0439			

Signal: DAD1 C, Sig=320,4 Ref=off

RT [min]	Type	Width [min]	Area	Height	Area%	Name
1.500	MM	0.1500	2901.0376	322.2332	100.0000	
		Sum	2901.0376			

Signal: MSD1 TIC, MS File

RT [min]	Type	Width [min]	Area	Height	Area%	Name
1.547	MM	0.2506	999195.6875	66448.6328	100.0000	
		Sum	999195.6875			

Signal: MSD2 TIC, MS File

RT [min]	Type	Width [min]	Area	Height	Area%	Name
1.532	MM	0.3023	654469.7500	36087.6758	100.0000	
		Sum	654469.7500			

Agilent Technologies

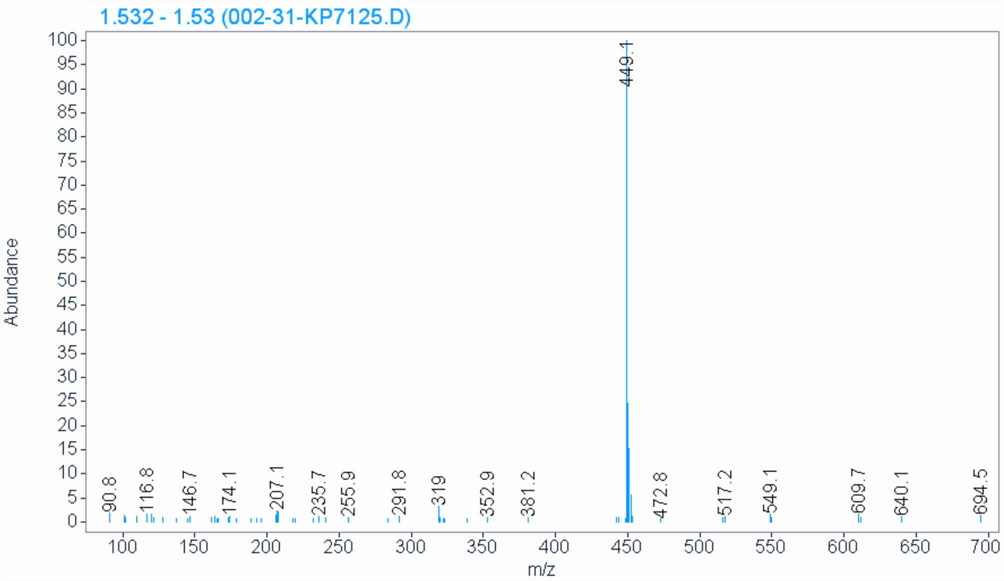

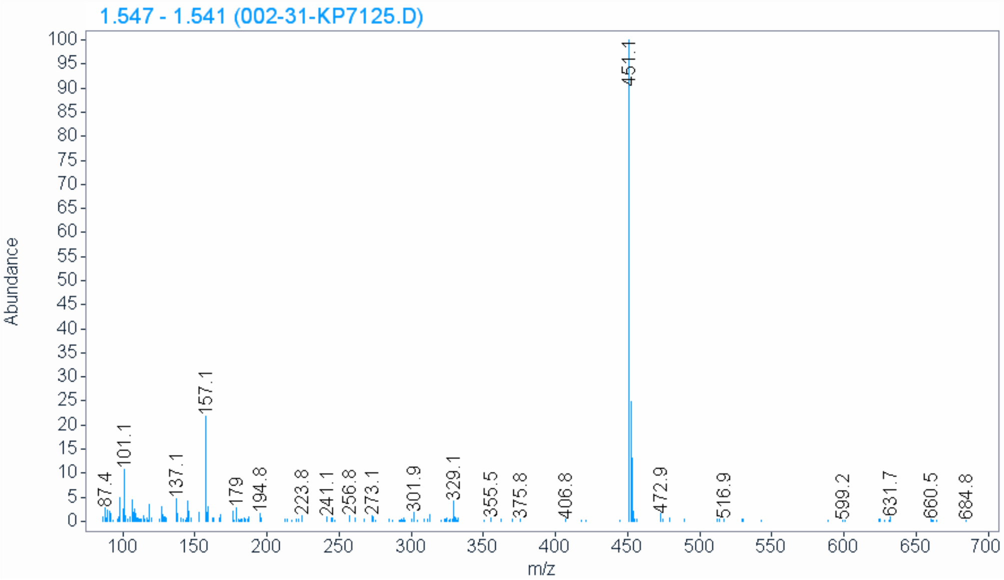

Compound Name: (*Z*)-*N*-(5-(2,3-dihydroxybenzylidene)-4-oxo-4,5-dihydrothiazol-2-yl)naphthalene-1-sulfonamide

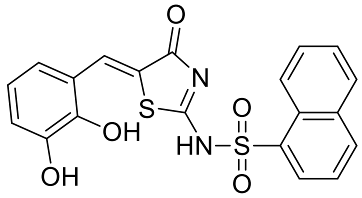

Chemical Formula: $C_{20}H_{14}N_2O_5S_2$
Exact Mass: 426.03
Molecular Weight: 426.47

Compound Code: 24 (KP9009)

Obtained Weight & Yield: 53 mg (25%)

Purity (by LCMS and ^{1}H NMR): > 99% by ^{1}H-NMR and LCMS

Appearance: yellow solid

Solubility: DMSO, slightly soluble in acetone and methanol.

Melting Point: > 158 °C (dec.)

TLC Rf (and conditions): N/A

IR Analysis (including assignment): IR (neat): v_{max} = 3441 (OH), 2983, 2887, 2745 (C-H aromatic), 1694 (C=O), 1554 (C-C aromatic), 1349 (sulfonamide), 1275 (C-O), 1120 (C-N) cm^{-1}

^{1}H NMR Analysis: ^{1}H NMR (600 MHz, DMSO) δ 13.08 (s, 1H, br, NH), 9.90 (s, 1H, OH), 9.56 (s, 1H, OH), 8.60 (d, *J* = 8.6 Hz, 1H), 8.29 (d, *J* = 7.5 Hz, 2H), 8.12 (d, *J* = 8.1 Hz, 1H), 8.00 (s, 1H), 7.76 (t, *J* = 7.7 Hz, 1H), 7.71 – 7.67 (m, 2H), 6.96 – 6.92 (m, 2H), 6.88 – 6.85 (m, 1H) ppm.
Starting material at 3.86 ppm (1.76%)

^{13}C NMR Analysis: ^{13}C DMPTQ NMR (151 MHz, DMSO) δ 166.6, 166.0, 146.3, 146.0, 135.3, 134.7, 133.8, 129.7, 129.0, 128.3, 128.1, 127.6, 127.1, 124.9, 124.7, 120.4, 119.9 (br), 119.8, 118.9, 118.0 ppm.

MS Analysis (low res): LRMS (ESI-) *m/z*: 425 (*M*-H, $C_{20}H_{13}N_2O_5S_2$, 100)

MS Analysis (high res): Exact mass calculated for $C_{20}H_{13}N_2O_5S_2$ [*M*-H]$^-$, 425.0300. Found 425.0274.

HPLC method details: Column: Zorbax SB-C18 Rapid Resolution HT 2.1x50mm 1.8-Micron; Method: LCMS ISOCRATIC 60%B_3 MINS.M filename: KP9008; Peak retention time: 0.681 mins; Area (%): 99.

Procedure: To a 10mL microwave vial was added N-(4-oxo-4,5-dihydrothiazol-2-yl)naphthalene-1-sulfonamide (146 mg, 0.49 mmol), 2,3-dihydroxybenzaldehyde (96 mg, 0.54 mmol, 1.1 eq), ethanol (3 mL) and a catalytic amount of the benzoic acid/piperidine catalyst (approximately 5 drops). The suspension was heated by microwave irradiation (120 °C, 200 W) for 30 min. A precipitate formed upon addition of H_2O (5 mL) and stirring at RT (5 h). The solid was collected by vacuum filtration to give the desired product as a yellow solid (53 mg, 25%).

Other analyses, reference papers, previously obtained data, comments, etc:

PerkinElmer Spectrum Version 10.4.00
Thursday, 26 November 2020 11:57 AM

Analyst	research
Date	Thursday, 26 November 2020 11:57 AM

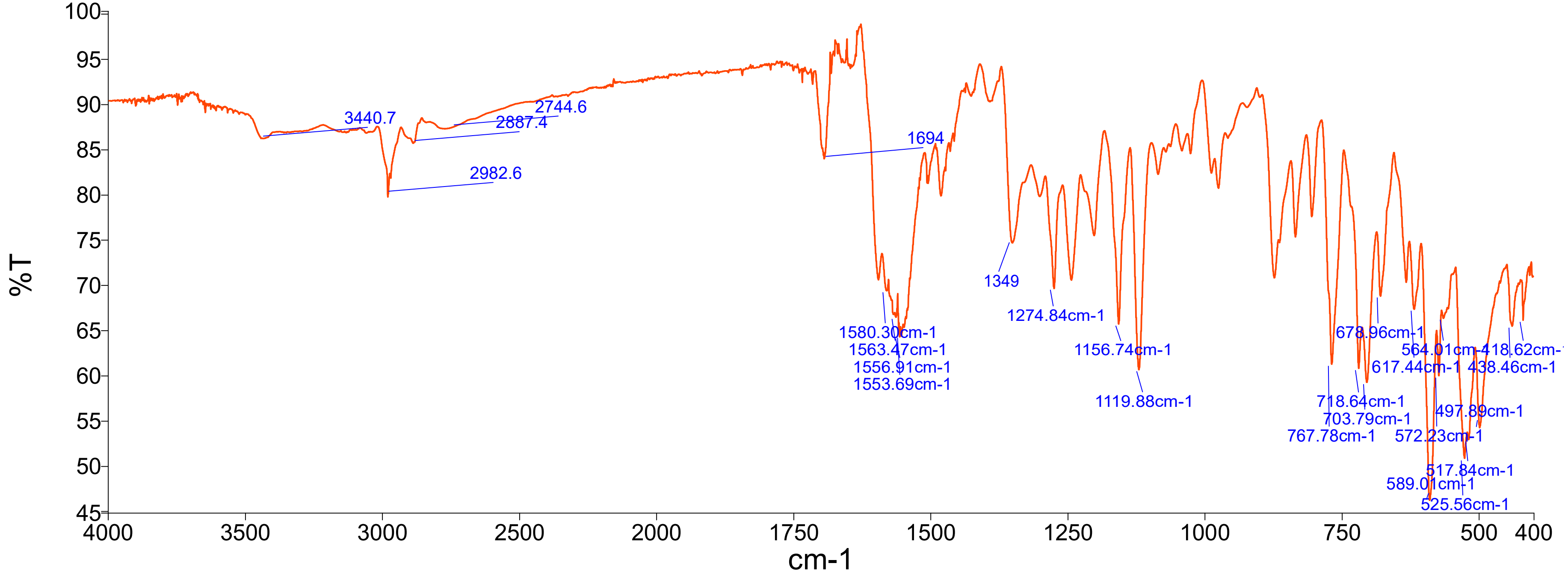

Sample Name	Description	Quality Checks
kp9009	Sample 183 By research Date Thursday, November 26 2020	The Quality Checks do not report any warnings for the sample.

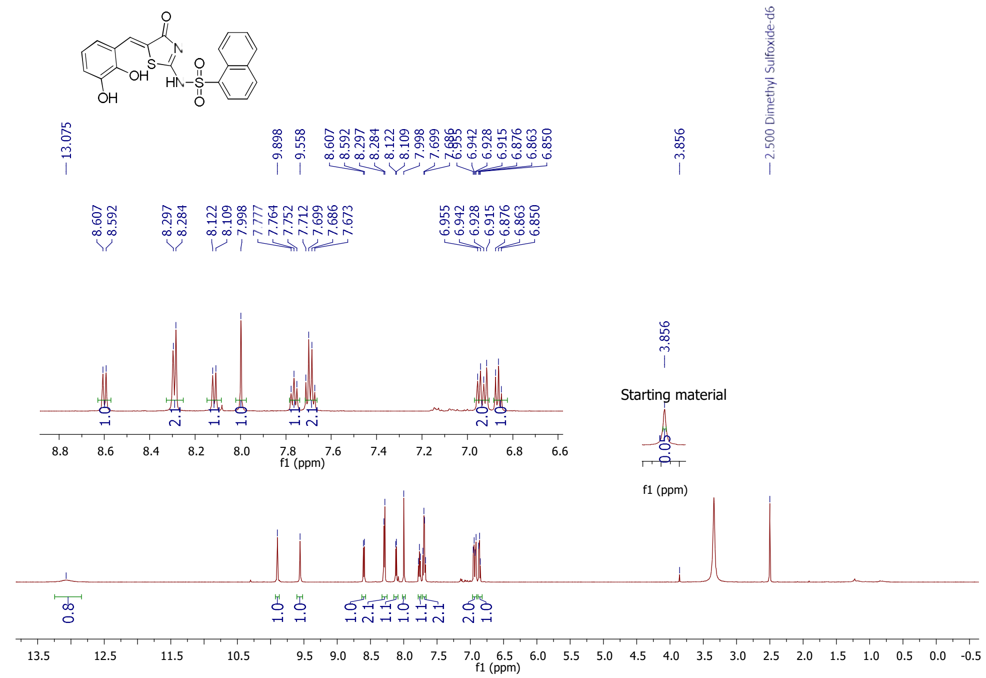

13.075
9.898
9.558
8.607
8.592
8.297
8.284
8.122
8.109
7.998
7.699
7.686
6.955
6.942
6.928
6.915
6.876
6.863
6.850
3.856
2.500 Dimethyl Sulfoxide-d6
8.607
8.592
8.297
8.284
8.122
8.109
7.998
7.777
7.764
7.752
7.712
7.699
7.686
7.673
6.955
6.942
6.928
6.915
6.876
6.863
6.850
3.856
Starting material
0.05
f1 (ppm)
f1 (ppm)
f1 (ppm)

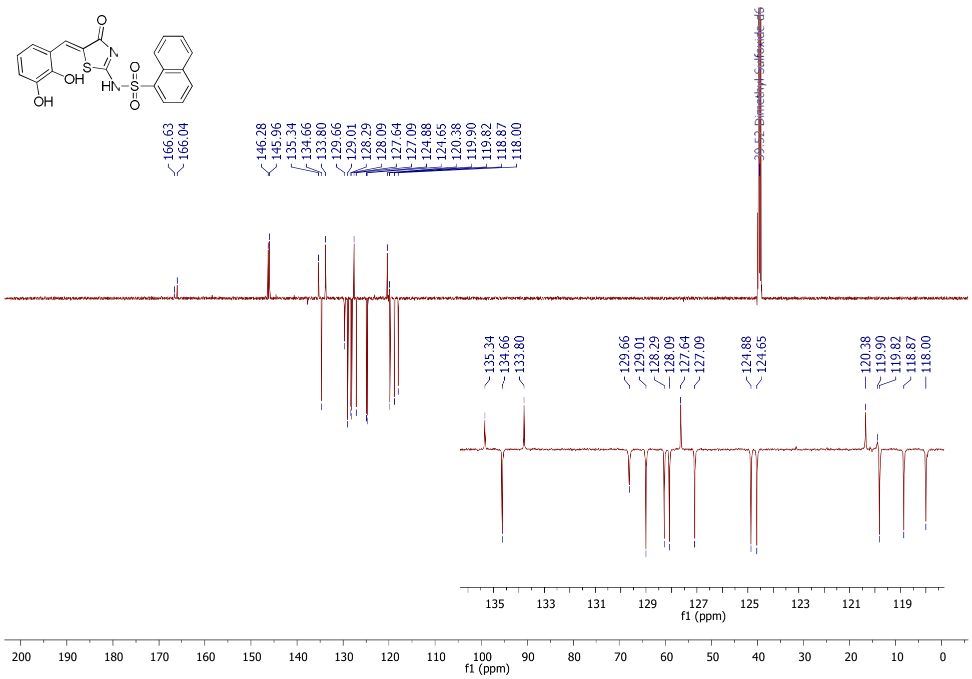

166.63
166.04
146.28
145.96
135.34
134.66
133.80
129.66
129.01
128.29
128.09
127.64
127.09
124.88
124.65
120.38
119.90
119.82
118.87
118.00
39.52 Dimethyl Sulfoxide-d6
135.34
134.66
133.80
129.66
129.01
128.29
128.09
127.64
127.09
124.88
124.65
120.38
119.90
119.82
118.87
118.00
f1 (ppm)
f1 (ppm)

LCMS Report

Agilent Technologies

**Data file:**	D:\Chem32\1\Data\KP\KP_DS_NOV2 2020-11-02 12-46-10\004-14-KP9009.D		
**Sample name:**	KP9009		
**Description:**			
**Sample amount:**	0.000	**Sample type:**	Sample
**Instrument:**	LCMS	**Location:**	14
**Injection date:**	11/2/2020 1:01:33 PM	**Injection:**	1 of 1
**Acq. method:**	LCMS ISOCRATIC 60% B_3MINS.M	**Injection volume:**	2.000
**Analysis method:**	LCMS ISOCRATIC 60%B_3MINS.M	**Acq. operator:**	SYSTEM
**Last changed:**	5/19/2016 3:52:53 PM		

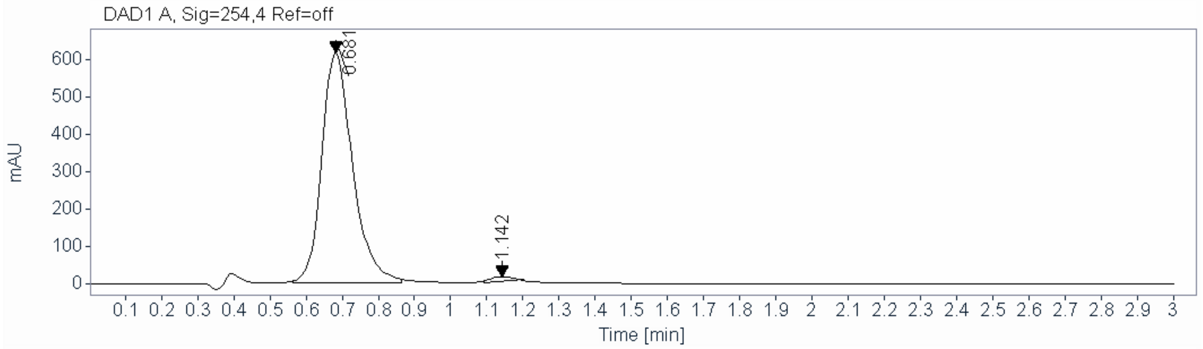

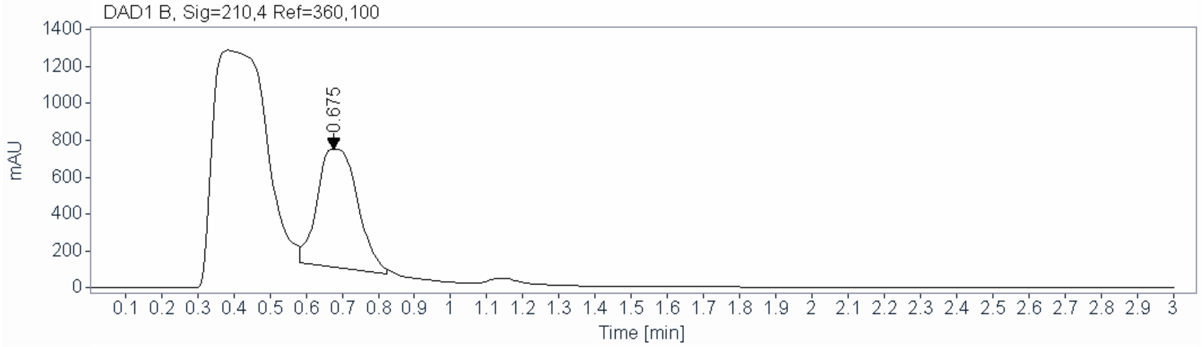

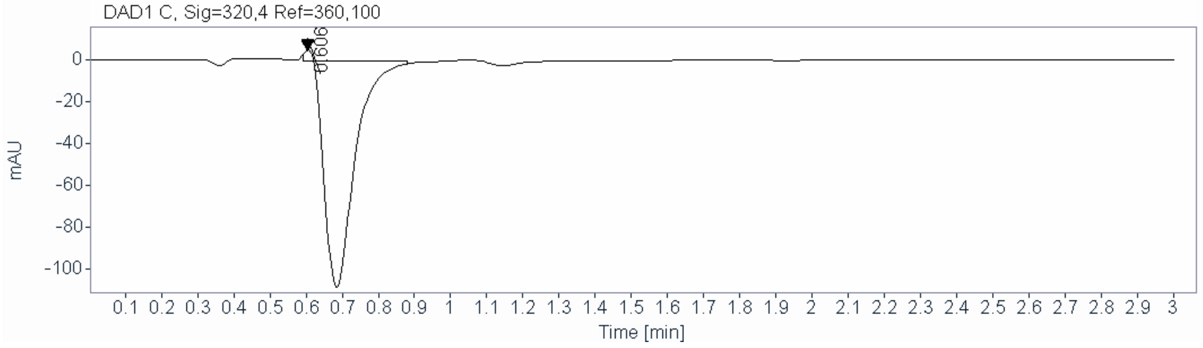

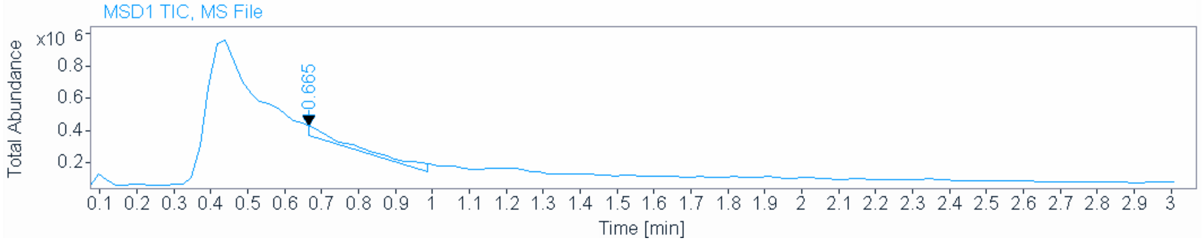

Agilent Technologies

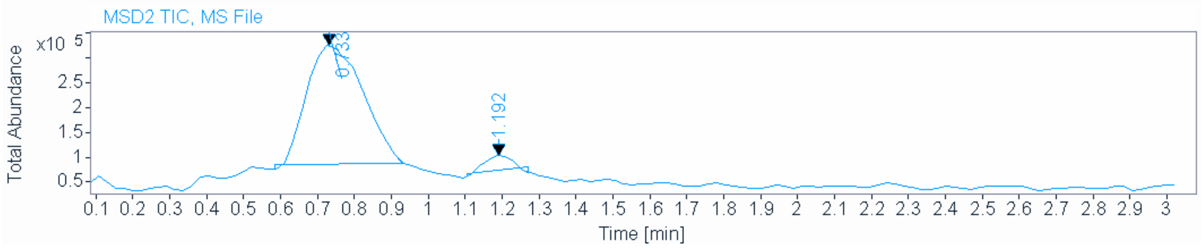

Signal: DAD1 A, Sig=254,4 Ref=off

RT [min]	Type	Width [min]	Area	Height	Area%	Name
0.681	MM	0.0993	3682.5947	618.0132	98.6329	
1.142	MM	0.0658	51.0435	12.9256	1.3671	
		Sum	3733.6382			

Signal: DAD1 B, Sig=210,4 Ref=360,100

RT [min]	Type	Width [min]	Area	Height	Area%	Name
0.675	MM	0.1294	4966.5649	639.4543	100.0000	
		Sum	4966.5649			

Signal: DAD1 C, Sig=320,4 Ref=360,100

RT [min]	Type	Width [min]	Area	Height	Area%	Name
0.606	MM	0.0184	5.8888	5.3410	100.0000	
		Sum	5.8888			

Signal: MSD1 TIC, MS File

RT [min]	Type	Width [min]	Area	Height	Area%	Name
0.665	MM	0.1529	566339.6875	61746.1328	100.0000	
		Sum	566339.6875			

Signal: MSD2 TIC, MS File

RT [min]	Type	Width [min]	Area	Height	Area%	Name
0.733	MM	0.1751	2529183.750 0	240768.8438	94.4962	
1.192	MM	0.0758	147308.7188	32369.1445	5.5038	
		Sum	2676492.468			

Agilent Technologies

0.733 - 0.726 (004-14-KP9009.D)

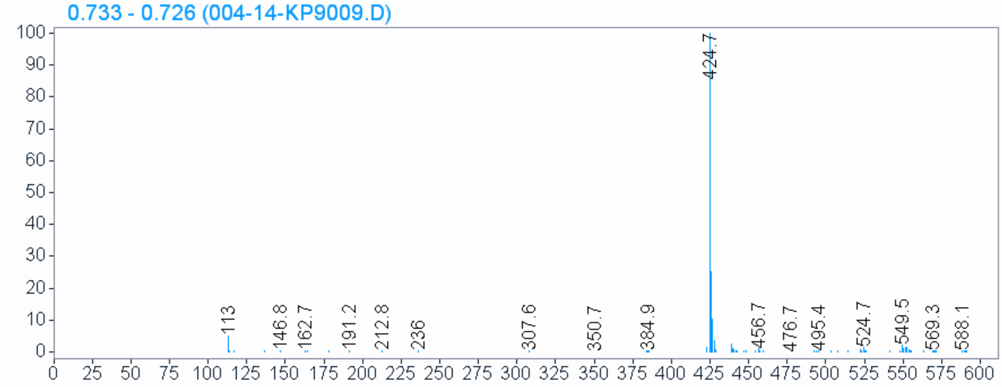

1.192 - 1.185 (004-14-KP9009.D)

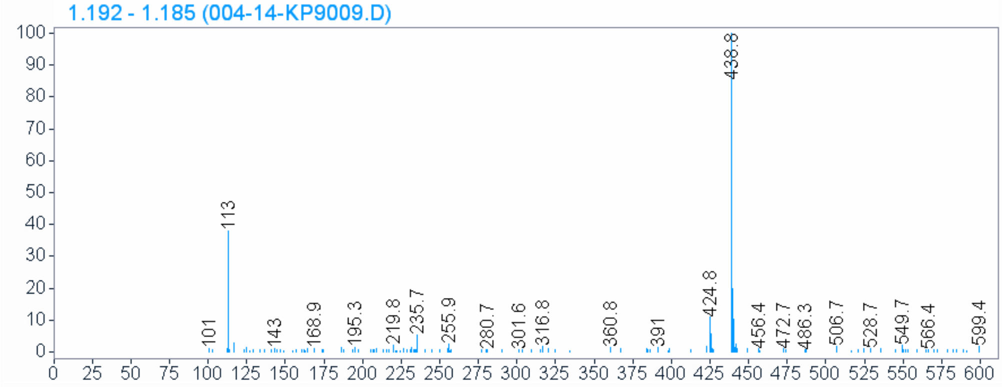

No peak data available for signal, cannot extract peak spectra, MSD1, 004-14-KP9009.D

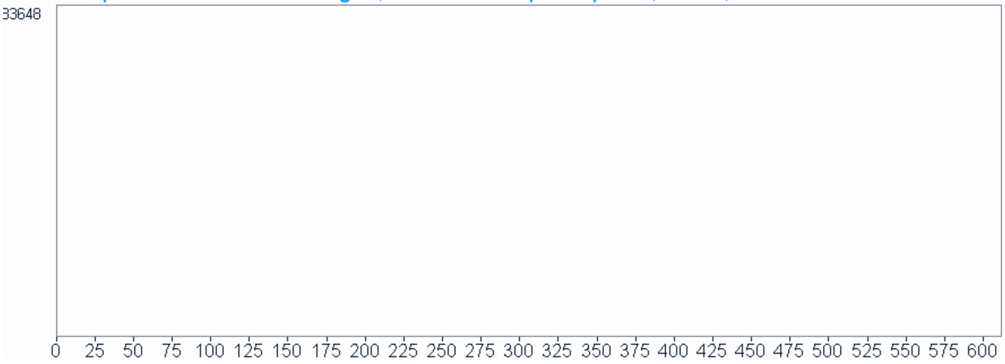

Compound Name: (*Z*)-*N*-(5-(2,4-dihydroxybenzylidene)-4-oxo-4,5-dihydrothiazol-2-yl)naphthalene-1-sulfonamide

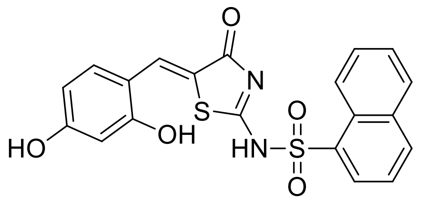

Chemical Formula: $C_{20}H_{14}N_2O_5S_2$
Exact Mass: 426.03
Molecular Weight: 426.47

Compound Code: 25 (KP9014)

Obtained Weight & Yield: 123 mg (59%)

Purity (by LCMS and ^{1}H NMR): > 99% by ^{1}H-NMR and LCMS

Appearance: bright yellow solid

Solubility: DMSO, slightly soluble in acetone and methanol.

Melting Point: > 236 °C (dec.)

TLC Rf (and conditions): N/A

IR Analysis (including assignment): IR (neat): ν_{max} = 3352 (OH), 2983, 2893, 2786 (C-H aromatic), 1685 (C=O), 1542 (C-C aromatic), 1211 (C-O), 1126 (C-N) cm^{-1}

^{1}H NMR Analysis: ^{1}H NMR (600 MHz, DMSO) δ 12.92 (s, 1H, br, NH), 10.67 (s, 1H, OH), 10.33 (s, 1H, OH), 8.60 (d, *J* = 8.6 Hz, 1H), 8.29 (d, *J* = 7.4 Hz, 2H), 8.11 (d, *J* = 8.1 Hz, 1H), 7.92 (s, 1H), 7.76 (t, *J* = 7.6 Hz, 1H), 7.71 – 7.67 (m, 2H), 7.30 (d, *J* = 8.6 Hz, 1H), 6.49 (d, *J* = 8.6 Hz, 1H), 6.44 (s, 1H) ppm. Ethanol at 1.05 ppm (0.86%)

^{13}C NMR Analysis: ^{13}C NMR (151 MHz, DMSO) δ 166.8, 166.1, 162.5, 159.7, 135.5, 134.6, 133.8, 131.1, 130.1, 129.0, 128.2, 128.0, 127.7, 127.1, 124.9, 124.6, 114.7, 111.5, 108.7, 102.6 ppm.

MS Analysis (low res): LRMS (ESI-) *m/z*: 425 (*M*-H, $C_{20}H_{13}N_2O_5S_2$, 100)

MS Analysis (high res): Exact mass calculated for $C_{20}H_{13}N_2O_5S_2$ [*M*-H]$^-$, 425.0300. Found 425.0272.

HPLC method details: Column: Zorbax SB-C18 Rapid Resolution HT 2.1x50mm 1.8-Micron; Method: LCMS ISOCRATIC 60%B_3 MINS.M filename: KP9014; Peak retention time: 0.59 mins; Area (%): 100.

Procedure: To a 10mL microwave vial was added *N*-(4-oxo-4,5-dihydrothiazol-2-yl)naphthalene-1-sulfonamide (152 mg, 0.49 mmol), 2,4-dihydroxybenzaldehyde (95 mg, 0.54 mmol, 1.1 eq), ethanol (3 mL) and a catalytic amount of the benzoic acid/piperidine catalyst (approximately 5 drops). The suspension was heated by microwave irradiation (120 °C, 200 W) for 30 min. A precipitate formed upon addition of H_2O (5 mL) and stirring at RT (5 h). Some starting material remaining – re-reacted with 2,4-dihydroxybenzaldehyde (66 mg) in ethanol (3 mL) with catalytic amount of the benzoic acid/piperidine catalyst at 120 °C for 30 min. A precipitate formed upon addition of H_2O (5 mL) and stirring at RT and the solid was collected by vacuum filtration to give the desired product as a yellow solid (123 mg, 59%).

Other analyses, reference papers, previously obtained data, comments, etc:

PerkinElmer Spectrum Version 10.4.00
Thursday, 26 November 2020 11:58 AM

Analyst research
Date Thursday, 26 November 2020 11:58 AM

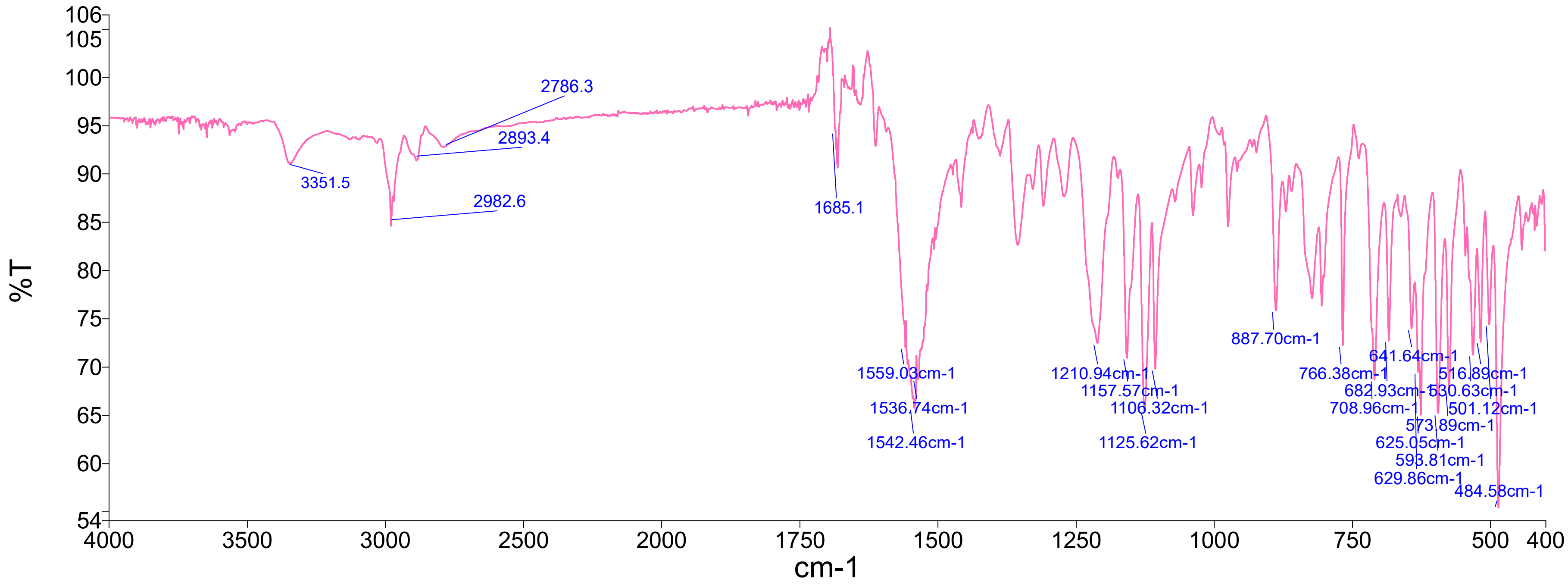

Sample Name	Description	Quality Checks
kp9014	Sample 185 By research Date Thursday, November 26 2020	The Quality Checks do not report any warnings for the sample.

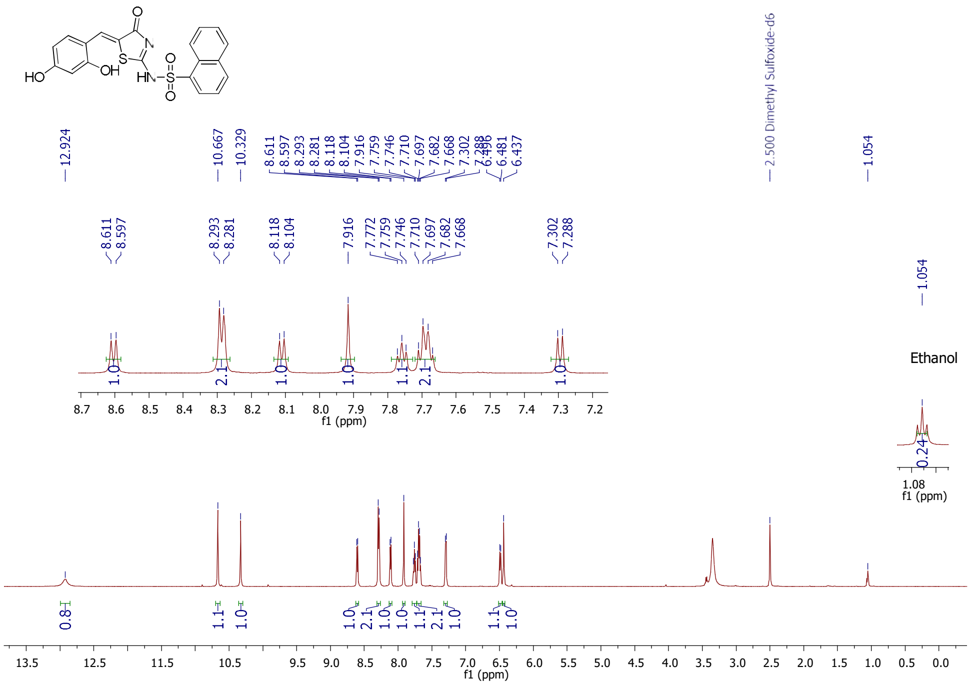
HO
OH
S
N
O
HN
S
O
O
12.924
10.667
10.329
8.611
8.597
8.293
8.281
8.118
8.104
7.916
7.759
7.746
7.710
7.697
7.682
7.668
7.302
7.288
6.496
6.481
6.437
2.500 Dimethyl Sulfoxide-d6
1.054
8.611
8.597
8.293
8.281
8.118
8.104
7.916
7.772
7.759
7.746
7.710
7.697
7.682
7.668
7.302
7.288
1.0
2.1
1.0
1.0
1.1
2.1
1.0
8.7 8.6 8.5 8.4 8.3 8.2 8.1 8.0 7.9 7.8 7.7 7.6 7.5 7.4 7.3 7.2
f1 (ppm)
1.054
Ethanol
0.24
1.08
f1 (ppm)
0.8
1.1
1.0
1.0
2.1
1.0
1.0
1.1
2.1
1.0
1.1
1.0
13.5 12.5 11.5 10.5 9.5 9.0 8.5 8.0 7.5 7.0 6.5 6.0 5.5 5.0 4.5 4.0 3.5 3.0 2.5 2.0 1.5 1.0 0.5 0.0
f1 (ppm)

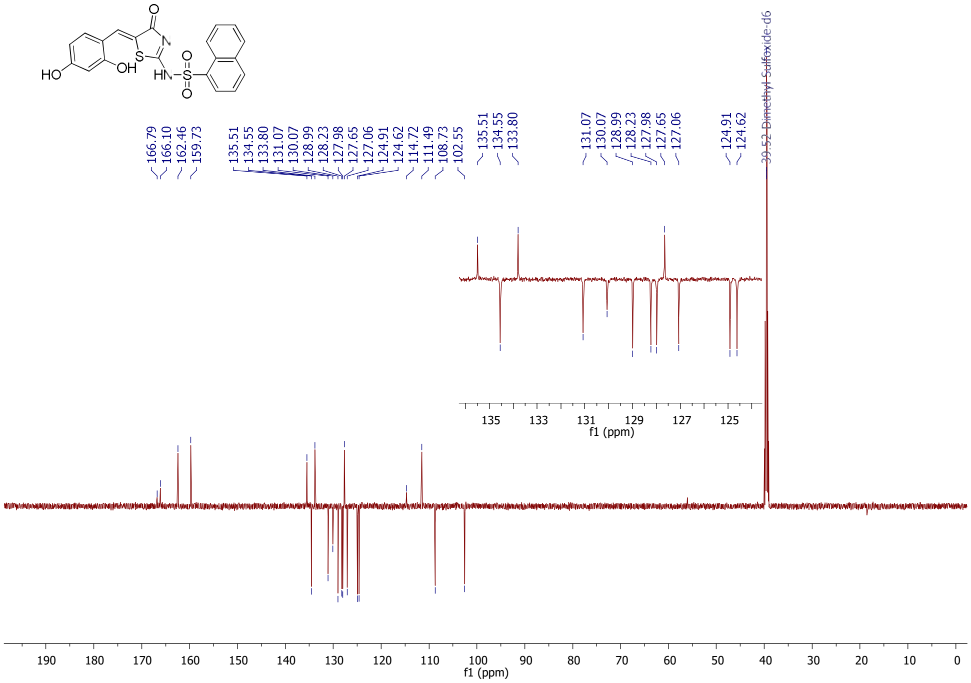
HO
OH
S
N
O
HN
S
O
O
166.79
166.10
162.46
159.73
135.51
134.55
133.80
131.07
130.07
128.99
128.23
127.98
127.65
127.06
124.91
124.62
114.72
111.49
108.73
102.55
135.51
134.55
133.80
131.07
130.07
128.99
128.23
127.98
127.65
127.06
124.91
124.62
39.52 Dimethyl Sulfoxide-d6
135 133 131 129 127 125
f1 (ppm)
190 180 170 160 150 140 130 120 110 100 90 80 70 60 50 40 30 20 10 0
f1 (ppm)

LCMS Report

**Data file:**	D:\Chem32\1\Data\KP\KP_DS_NOV2 2020-11-02 12-46-10\005-13-KP9014.D		
**Sample name:**	KP9014		
**Description:**			
**Sample amount:**	0.000	**Sample type:**	Sample
**Instrument:**	LCMS	**Location:**	13
**Injection date:**	11/2/2020 1:06:04 PM	**Injection:**	1 of 1
**Acq. method:**	LCMS ISOCRATIC 60% B_3MINS.M	**Injection volume:**	2.000
**Analysis method:**	LCMS ISOCRATIC 60%B_3MINS.M	**Acq. operator:**	SYSTEM
**Last changed:**	5/19/2016 3:52:53 PM		

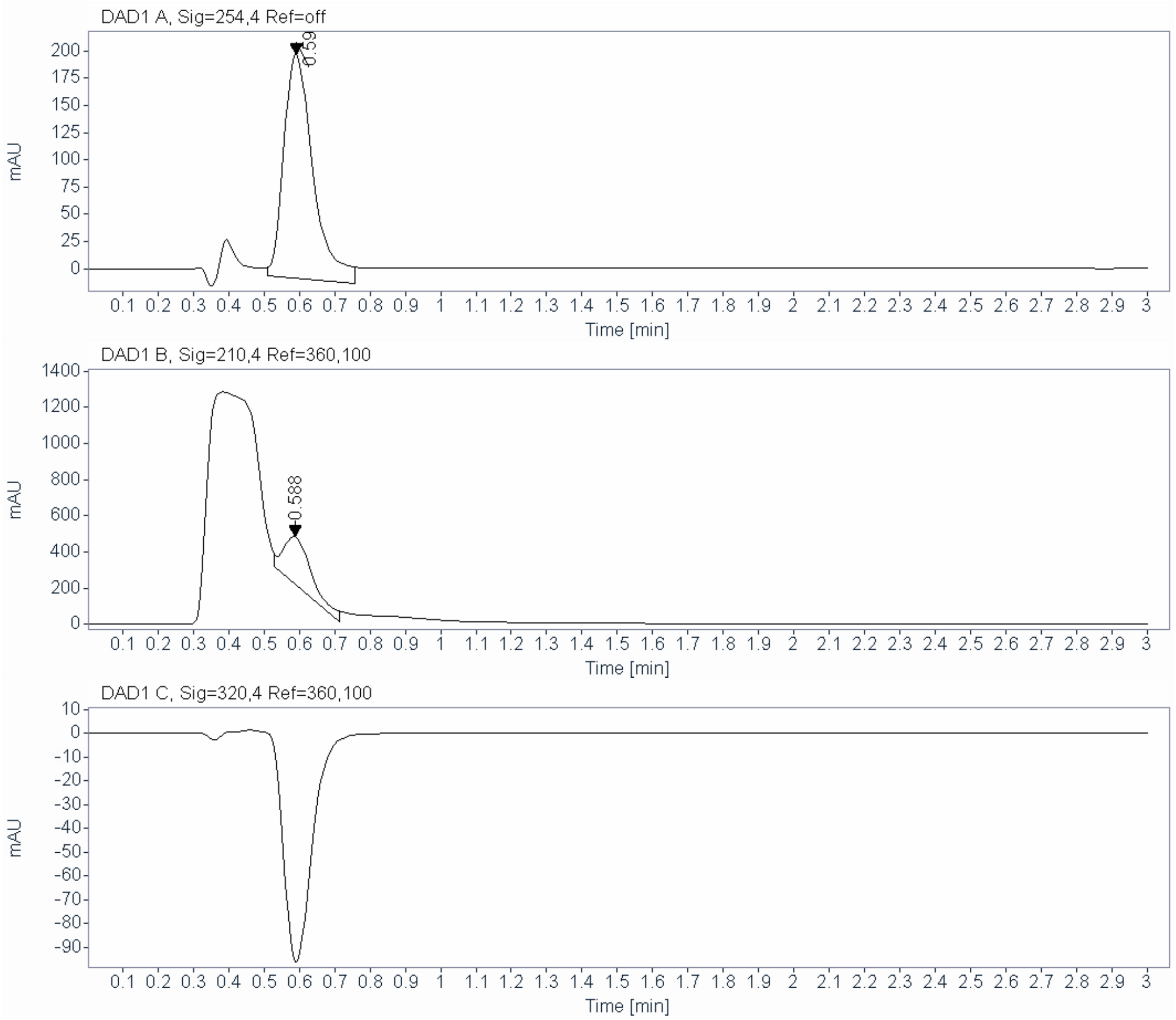

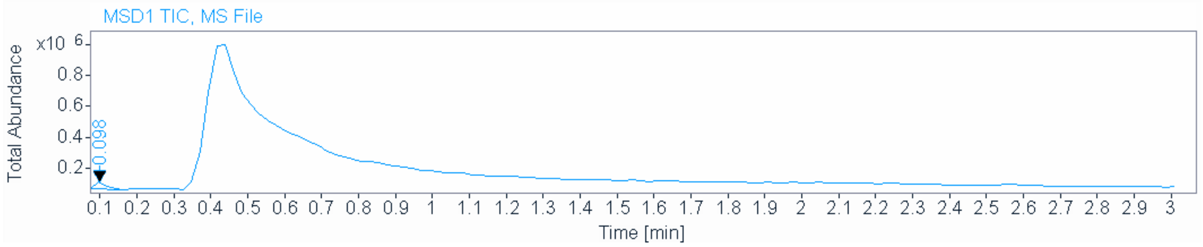

LCMS Report

Agilent Technologies

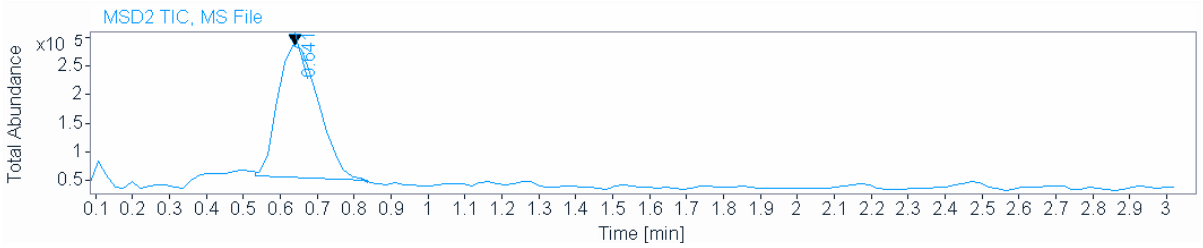

Signal: DAD1 A, Sig=254,4 Ref=off

RT [min]	Type	Width [min]	Area	Height	Area%	Name
0.590	MM	0.0958	1185.4271	206.2753	100.0000	
		Sum	1185.4271			

Signal: DAD1 B, Sig=210,4 Ref=360,100

RT [min]	Type	Width [min]	Area	Height	Area%	Name
0.588	MM	0.0929	1476.6904	265.0026	100.0000	
		Sum	1476.6904			

Signal: MSD1 TIC, MS File

RT [min]	Type	Width [min]	Area	Height	Area%	Name
0.098	BB	0.0339	102370.1953	50400.0313	100.0000	
		Sum	102370.1953			

Signal: MSD2 TIC, MS File

RT [min]	Type	Width [min]	Area	Height	Area%	Name
0.641	MM	0.1262	1768187.750 0	233594.2344	100.0000	
		Sum	1768187.750			

0.098 - 0.096 (005-13-KP9014.D)

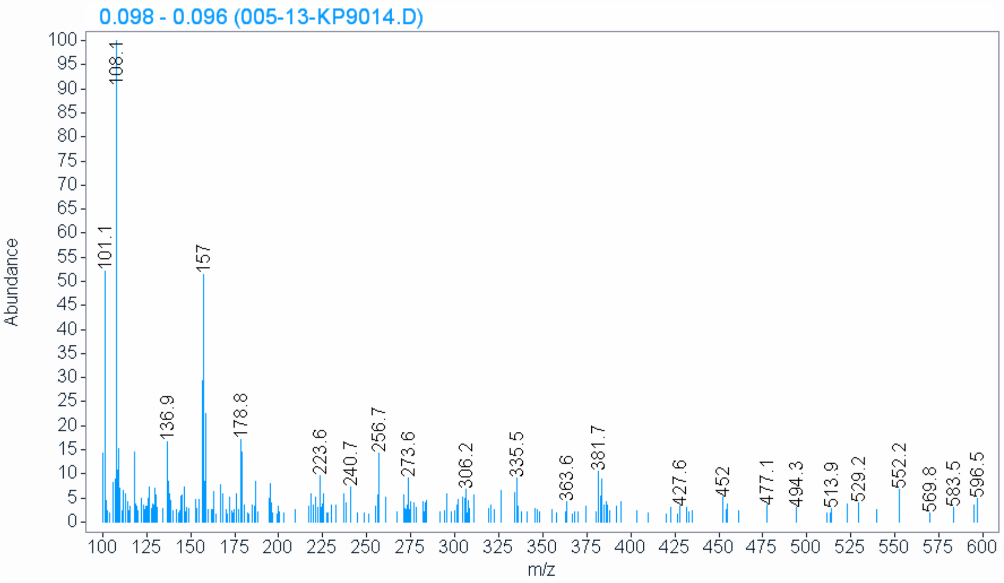

0.641 - 0.635 (005-13-KP9014.D)

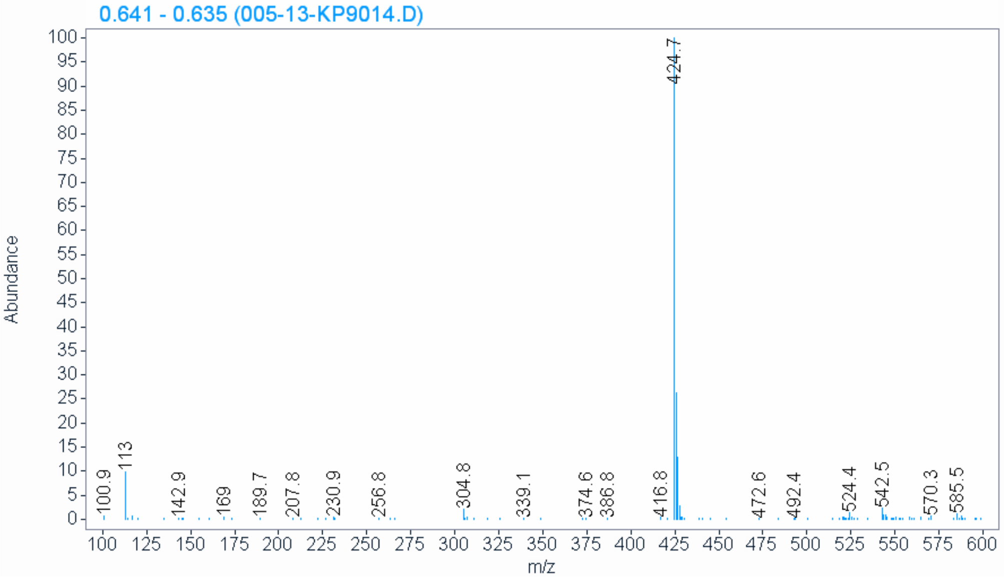

Compound Name: (*Z*)-*N*-(5-(2,5-dihydroxybenzylidene)-4-oxo-4,5-dihydrothiazol-2-yl)naphthalene-1-sulfonamide

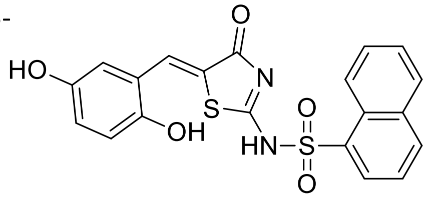

Chemical Formula: $C_{20}H_{14}N_2O_5S_2$
Exact Mass: 426.03
Molecular Weight: 426.47

Compound Code: 26 (KP9015)

Obtained Weight & Yield: 86 mg (44%)

Purity (by LCMS and ^{1}H NMR): > 97% by ^{1}H-NMR and > 99% LCMS

Appearance: bright yellow solid

Solubility: DMSO, slightly soluble in acetone and methanol.

Melting Point: > 225 °C (dec.)

TLC Rf (and conditions): N/A

IR Analysis (including assignment): IR (neat): ν_{max} = 3518, 3381 (O-H), 3167 (N-H), 2977, 2887 (C-H aromatic), 1706 (C=O), 1561 (C-C aromatic), 1306 (sulfonamide), 1158 (C-O), 1121 (C-N) cm^{-1}

^{1}H NMR Analysis: ^{1}H NMR (600 MHz, DMSO) δ 13.09 (s, 1H, br, NH), 9.95 (s, 1H, OH), 9.34 (s, 1H, OH), 8.60 (d, *J* = 8.6 Hz, 1H), 8.31 – 8.29 (m, 2H), 8.12 (d, *J* = 8.1 Hz, 1H), 7.93 (s, 1H), 7.77 (t, *J* = 7.7 Hz, 1H), 7.72 – 7.68 (m, 2H), 6.89 (s, 1H), 6.82 (s, 2H) ppm.
Ethanol at 1.05 ppm (1.14%) and some starting material remaining at 4.04 ppm (1.42%).

^{13}C NMR Analysis: ^{13}C NMR (151 MHz, DMSO) δ 166.8 (br), 166.0 (br), 150.7, 150.2, 135.4, 134.7, 133.8, 129.2, 129.0, 128.3, 128.0, 127.6, 127.1, 124.9, 124.6, 120.7, 119.6, 119.5 (br), 117.3, 113.5 ppm.

MS Analysis (low res): LRMS (ESI-) *m/z*: 425 (*M*-H, $C_{20}H_{13}N_2O_5S_2$, 100)

MS Analysis (high res): Exact mass calculated for $C_{20}H_{13}N_2O_5S_2$ [*M*-H]$^-$, 425.0300. Found 425.0272.

HPLC method details: Column: Zorbax SB-C18 Rapid Resolution HT 2.1x50mm 1.8-Micron; Method: LCMS ISOCRATIC 60%B_3 MINS.M filename: KP9015; Peak retention time: 0.579 mins; Area (%): 100.

Procedure: To a 10mL microwave vial was added *N*-(4-oxo-4,5-dihydrothiazol-2-yl)naphthalene-1-sulfonamide (152 mg, 0.49 mmol), 2,5-dihydroxybenzaldehyde (75 mg, 0.54 mmol, 1.1 eq), ethanol (3 mL) and a catalytic amount of the benzoic acid/piperidine catalyst (approximately 5 drops). The suspension was heated by microwave irradiation (120 °C, 200 W) for 30 min. A precipitate formed upon addition of H_2O (5 mL) and stirring at RT (overnight). The solid was collected by vacuum filtration to give the desired product as a bright yellow solid (86 mg, 44%).

Other analyses, reference papers, previously obtained data, comments, etc:

PerkinElmer Spectrum Version 10.4.00
Thursday, 26 November 2020 11:57 AM

Analyst research
Date Thursday, 26 November 2020 11:57 AM

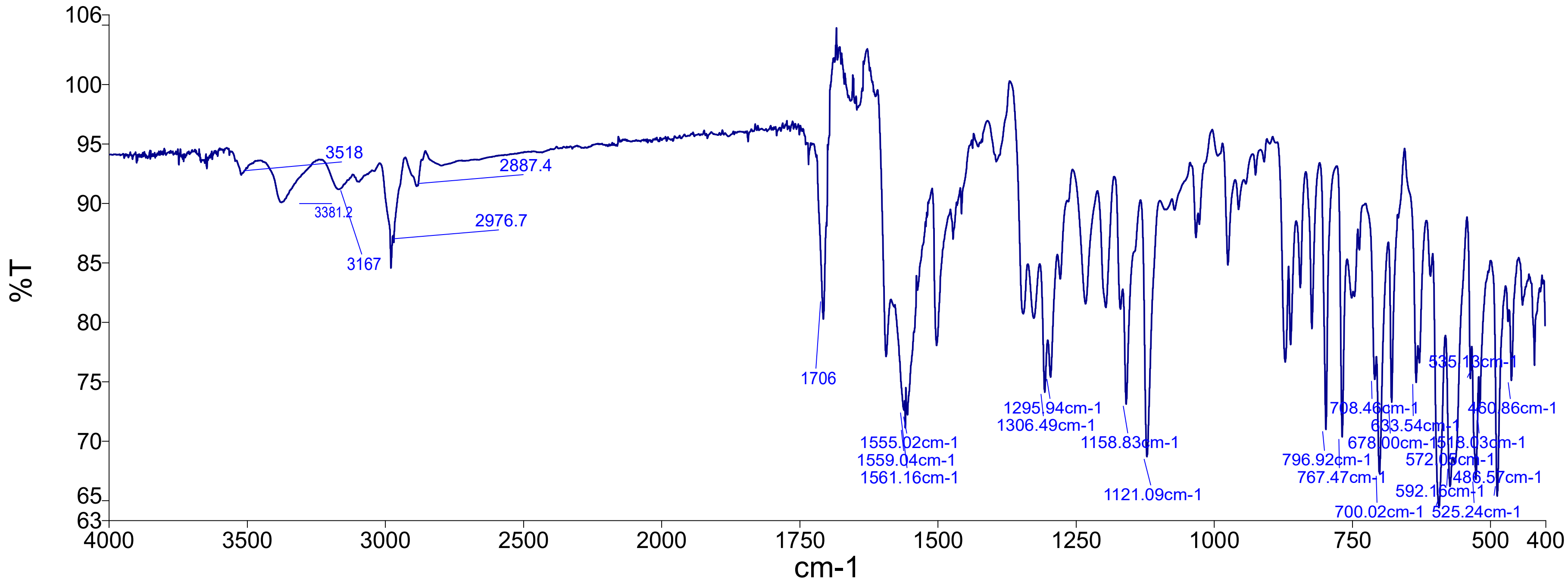

Sample Name	Description	Quality Checks
kp9015	Sample 184 By research Date Thursday, November 26 2020	The Quality Checks give rise to a Weak Bands warning for the sample.

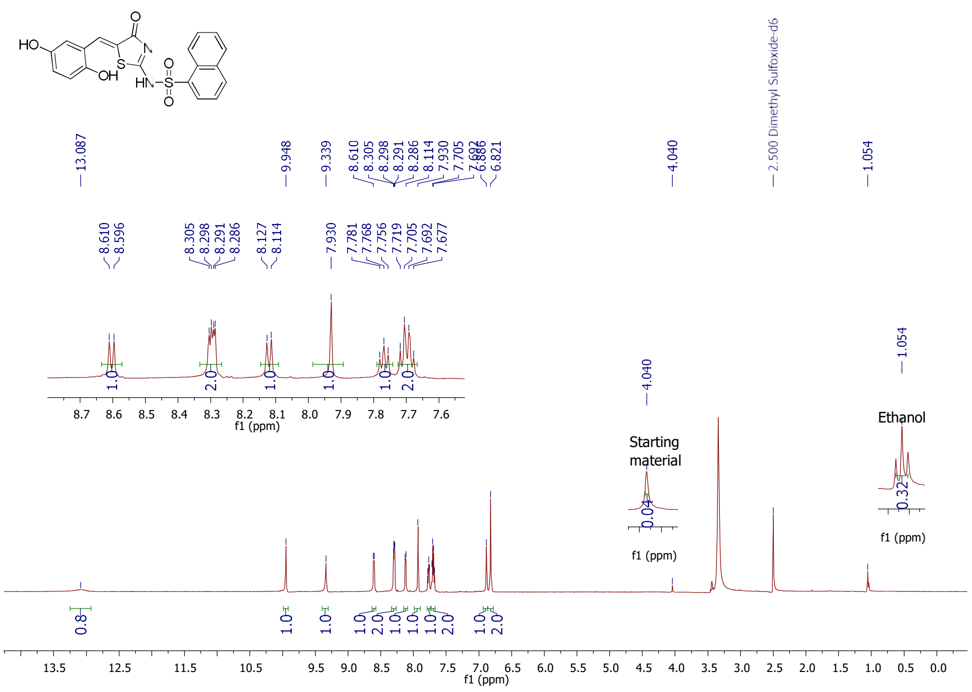

HO
OH
S
N
O
HN
S
O
O
13.087
9.948
9.339
8.610
8.305
8.298
8.291
8.286
8.114
7.930
7.705
6.886
6.821
4.040
2.500 Dimethyl Sulfoxide-d6
1.054
Starting material
Ethanol
0.04
0.32
f1 (ppm)
f1 (ppm)
f1 (ppm)

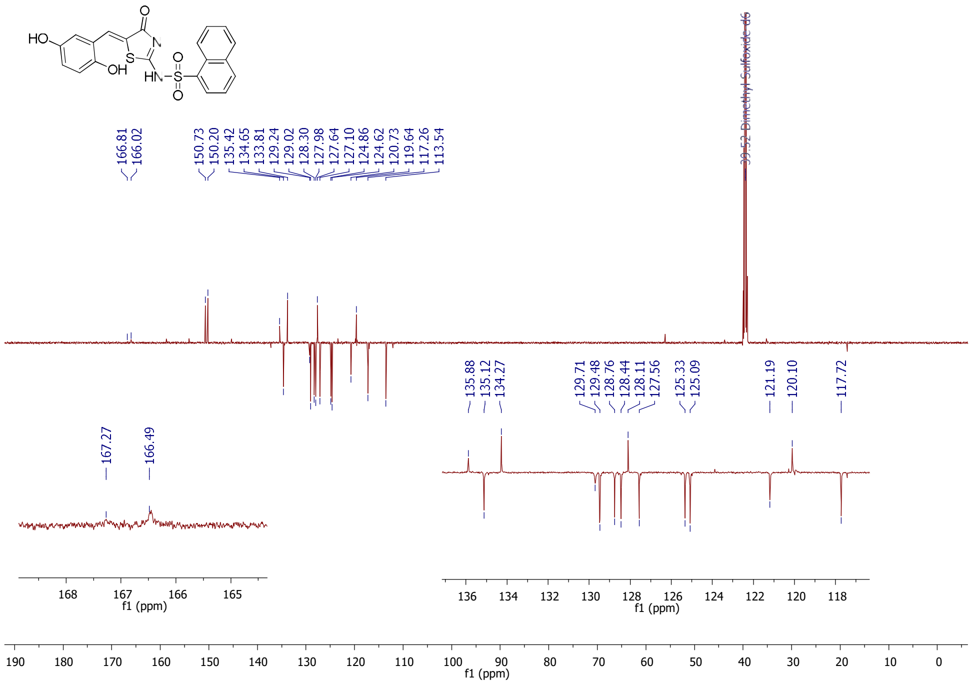

HO
OH
S
N
O
HN
S
O
O
166.81
166.02
150.73
150.20
135.42
134.65
133.81
129.24
129.02
128.30
127.98
127.64
127.10
124.86
124.62
120.73
119.64
117.26
113.54
39.52 Dimethyl Sulfoxide-d6
167.27
166.49
135.88
135.12
134.27
129.71
129.48
128.76
128.44
128.11
127.56
125.33
125.09
121.19
120.10
117.72
f1 (ppm)
f1 (ppm)
f1 (ppm)

LCMS Report

Agilent Technologies

**Data file:**	D:\Chem32\1\Data\KP\KP_DS_NOV3 2020-11-02 13-09-16\002-30-KP9015.D		
**Sample name:**	KP9015		
**Description:**			
**Sample amount:**	0.000	**Sample type:**	Sample
**Instrument:**	LCMS	**Location:**	30
**Injection date:**	11/2/2020 1:15:35 PM	**Injection:**	1 of 1
**Acq. method:**	LCMS ISOCRATIC 60% B_3MINS.M	**Injection volume:**	2.000
**Analysis method:**	LCMS ISOCRATIC 60%B_3MINS.M	**Acq. operator:**	SYSTEM
**Last changed:**	5/19/2016 3:52:53 PM		

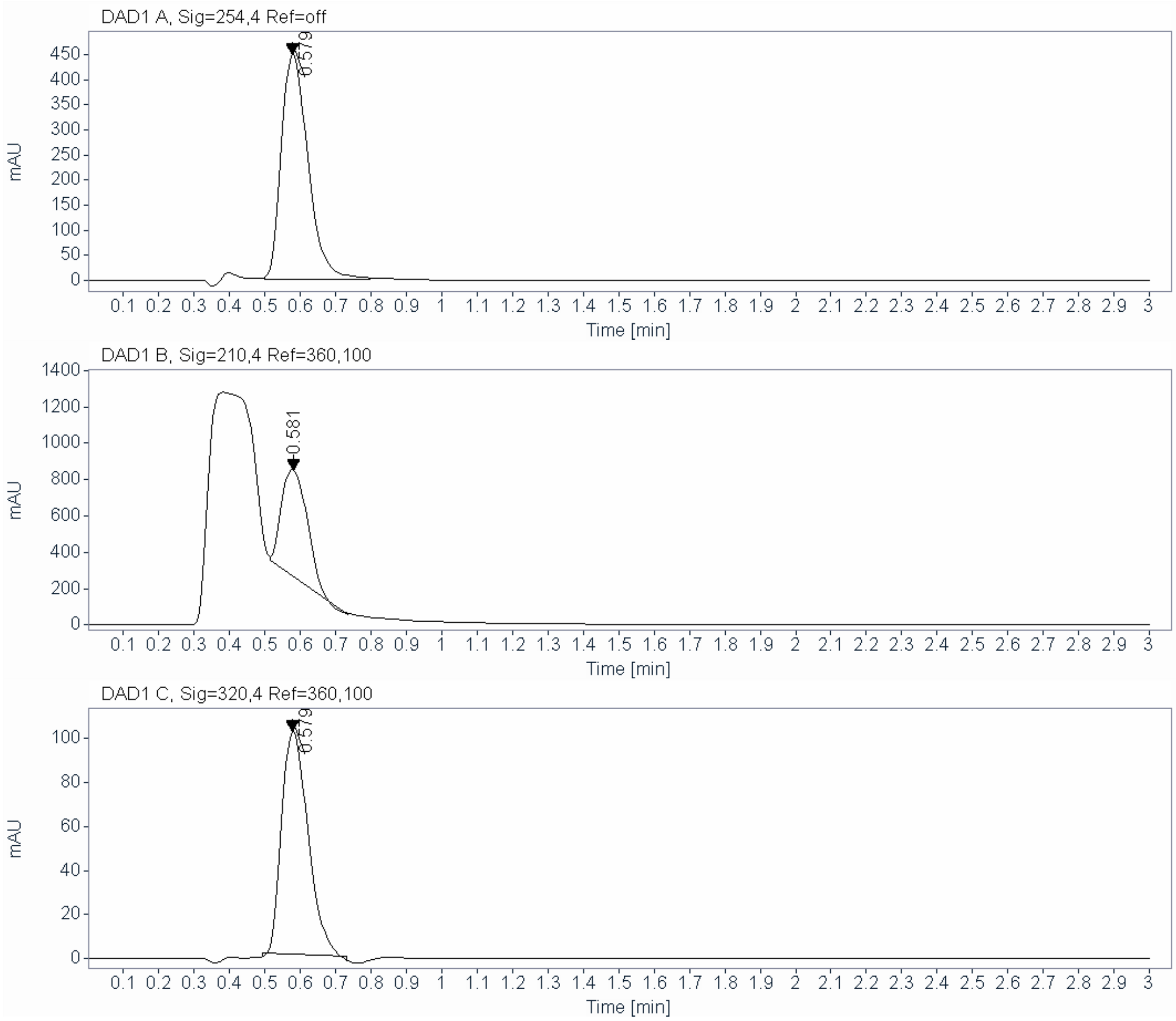

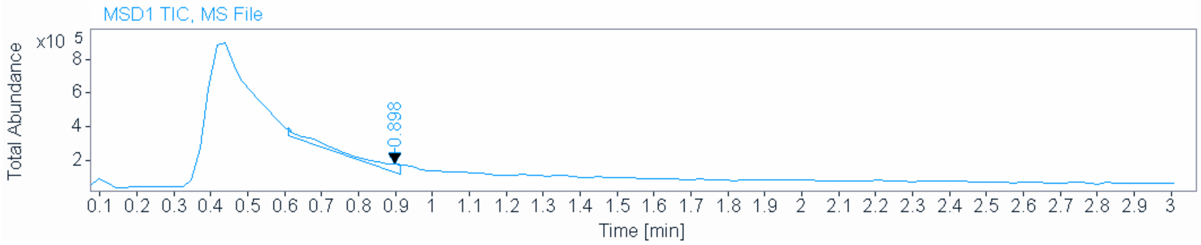

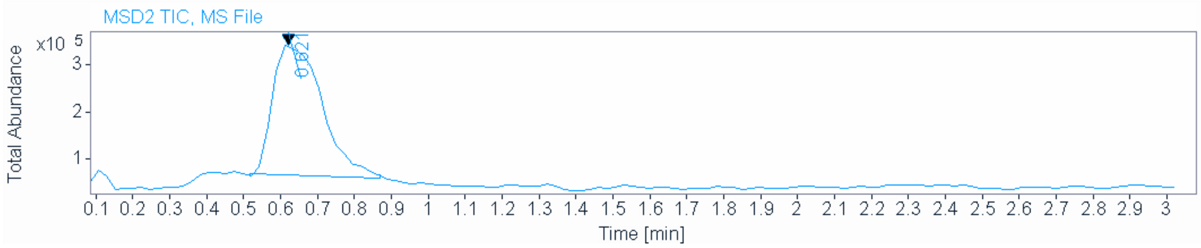

Signal: DAD1 A, Sig=254,4 Ref=off

RT [min]	Type	Width [min]	Area	Height	Area%	Name
0.579	MM	0.0878	2376.0732	450.9687	100.0000	
		Sum	2376.0732			

Signal: DAD1 B, Sig=210,4 Ref=360,100

RT [min]	Type	Width [min]	Area	Height	Area%	Name
0.581	MM	0.0847	2964.8955	583.3640	100.0000	
		Sum	2964.8955			

Signal: DAD1 C, Sig=320,4 Ref=360,100

RT [min]	Type	Width [min]	Area	Height	Area%	Name
0.579	MM	0.0863	524.2222	101.2887	100.0000	
		Sum	524.2222			

Signal: MSD1 TIC, MS File

RT [min]	Type	Width [min]	Area	Height	Area%	Name
0.898	MM	0.1609	451174.2813	46733.4766	100.0000	
		Sum	451174.2813			

Signal: MSD2 TIC, MS File

RT [min]	Type	Width [min]	Area	Height	Area%	Name
0.621	MM	0.1519	2583067.750 0	283398.7500	100.0000	
		Sum	2583067.750			

0.621 - 0.612 (002-30-KP9015.D)

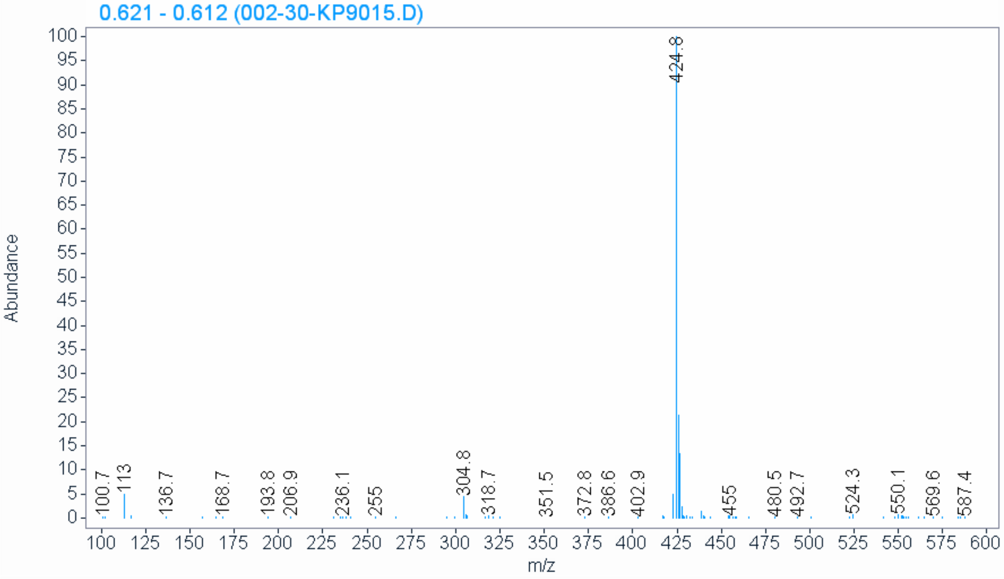

0.898 - 0.898 (002-30-KP9015.D)

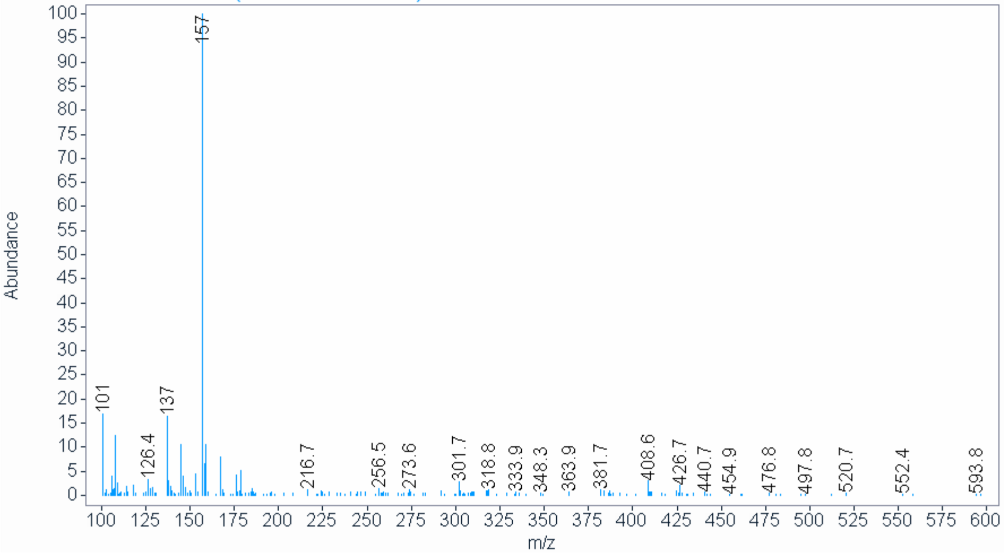

Compound Name: (*Z*)-*N*-(5-(3,4-dihydroxybenzylidene)-4-oxo-4,5-dihydrothiazol-2-yl)naphthalene-1-sulfonamide

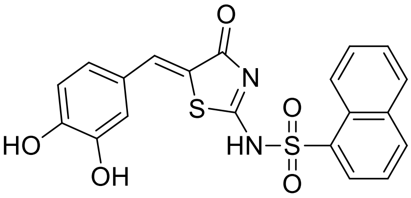

Chemical Formula: $C_{20}H_{14}N_2O_5S_2$
Exact Mass: 426.03
Molecular Weight: 426.47

Compound Code: 27 (KP9035)

Obtained Weight & Yield: 175 mg (84%)

Purity (by LCMS and ^{1}H NMR): > 94% by ^{1}H-NMR and > 99% by LCMS

Appearance: yellow/orange solid

Solubility: DMSO, slightly soluble in acetone and methanol.

Melting Point: 247 – 249 °C (dec.)

TLC Rf (and conditions): N/A

IR Analysis (including assignment): IR (neat): ν_{max} = 3468 (NH), 3356 (OH), 2974 (C-H aromatic), 2768 (C-H alkyl), 1707 (C=O), 1559 (C=C), 1292 (sulfonamide), 1157 (C-N) cm^{-1}

^{1}H NMR Analysis: ^{1}H NMR (400 MHz, DMSO) δ 13.03 (s, 1H, NH), 9.97 (s, 1H, OH), 9.69 (s, 1H, OH), 8.61 (d, *J* = 8.6 Hz, 1H), 8.32 – 8.28 (m, 2H), 8.12 (d, *J* = 8.1 Hz, 1H), 7.77 (t, *J* = 7.3 Hz, 1H), 7.73 – 7.67 (m, 2H), 7.60 (s, 1H), 7.11 (d, *J* = 1.9 Hz, 1H), 7.05 (dd, *J* = 8.3, 1.9 Hz, 1H), 6.92 (d, *J* = 8.2 Hz, 1H) ppm. Ethanol impurity at 1.05 ppm (5.99%).

^{13}C NMR Analysis: ^{13}C NMR (101 MHz, DMSO) δ 166.7, 165.9, 149.4, 146.1, 135.4, 134.9, 134.7, 133.8, 129.0, 128.3, 128.0, 127.7, 127.1, 125.0, 124.9, 124.6, 124.1, 116.7, 116.4, 116.3 ppm.

MS Analysis (low res): LRMS (ESI-): 425 (*M*-H, $C_{20}H_{13}N_2O_5S_2$, 100)

MS Analysis (high res): Exact mass calculated for $C_{20}H_{13}N_2O_5S_2$ [*M*-H]$^-$, 425.0300. Found 425.0271.

HPLC method details: Column: Zorbax SB-C18 Rapid Resolution HT 2.1x50mm 1.8-Micron; Method: LCMS ISOCRATIC 80%B_3 MINS.M filename: KP9035; Peak retention time: 0.425 mins; Area (%): 100.

Procedure: To a 10 mL microwave vial was added *N*-(4-oxo-4,5-dihydrothiazol-2-yl)naphthalene-1-sulfonamide (150 mg, 0.49 mmol), 3,4-dihydroxybenzaldehyde (102 mg, 0.734 mmol, 1.1 eq), ethanol (3 mL) and a catalytic amount of the benzoic acid/piperidine catalyst (approximately 5 drops). The suspension was heated by microwave irradiation (120 °C, 200 W) for 1 h. A precipitate formed upon cooling. The solid was collected by vacuum filtration to give the desired product as a yellow/orange solid (175 mg, 84%).

Other analyses, reference papers, previously obtained data, comments, etc:

PerkinElmer Spectrum Version 10.4.00
Monday, 10 May 2021 11:21 AM

Analyst research
Date Monday, 10 May 2021 11:21 AM

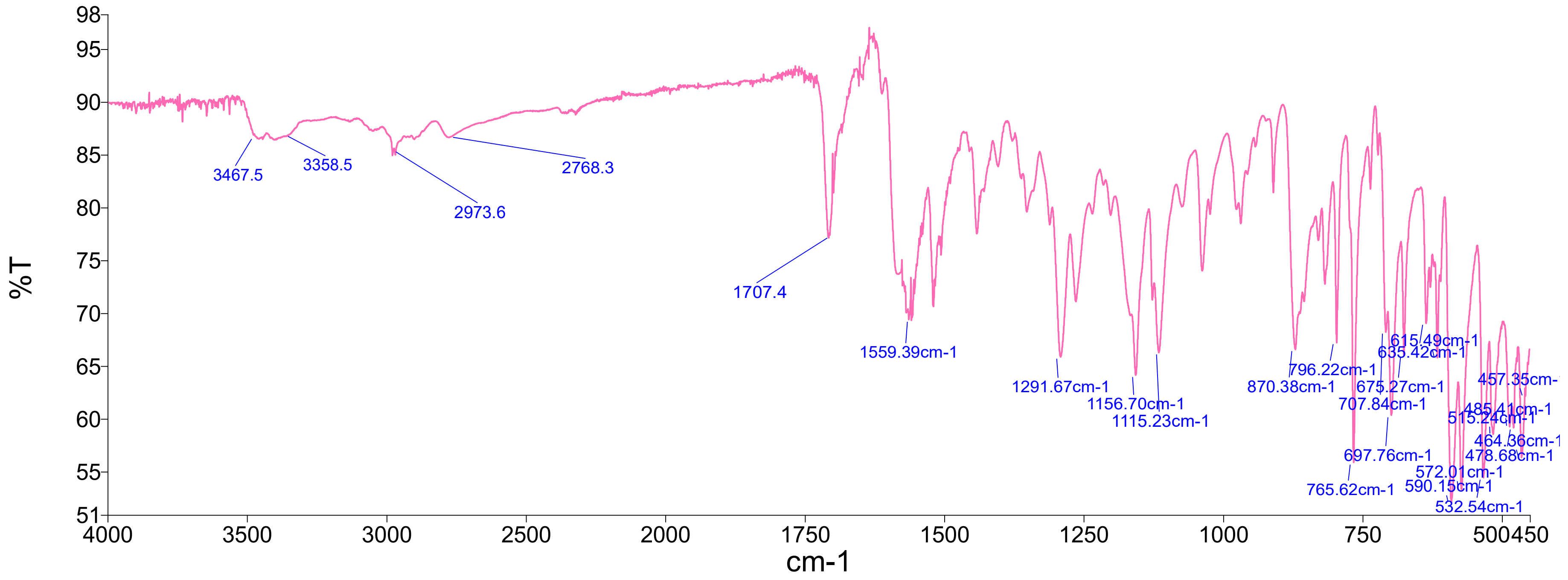

Sample Name	Description	Quality Checks
kp9035_001	Sample 011 By research Date Monday, May 10 2021	The Quality Checks do not report any warnings for the sample.

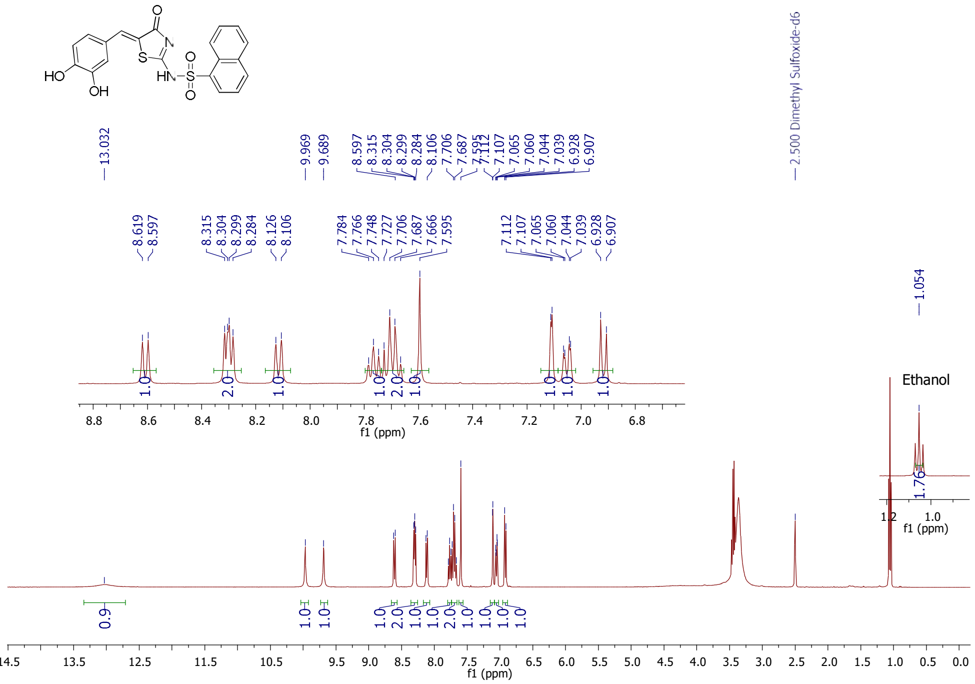
HO
OH
O
N
S
HN
S
O
O
13.032
9.969
9.689
8.597 8.315 8.304 8.299 8.284 8.106 7.706 7.687 7.595 7.112 7.107 7.065 7.060 7.044 7.039 6.928 6.907
2.500 Dimethyl Sulfoxide-d6
8.619 8.597
8.315 8.304 8.299 8.284
8.126 8.106
7.784 7.766 7.748 7.727 7.706 7.687 7.666 7.595
7.112 7.107 7.065 7.060 7.044 7.039 6.928 6.907
1.054
Ethanol
1.76
f1 (ppm)
0.9
1.0 1.0
1.0 2.0 1.0 1.0 2.0 1.0 1.0 1.0

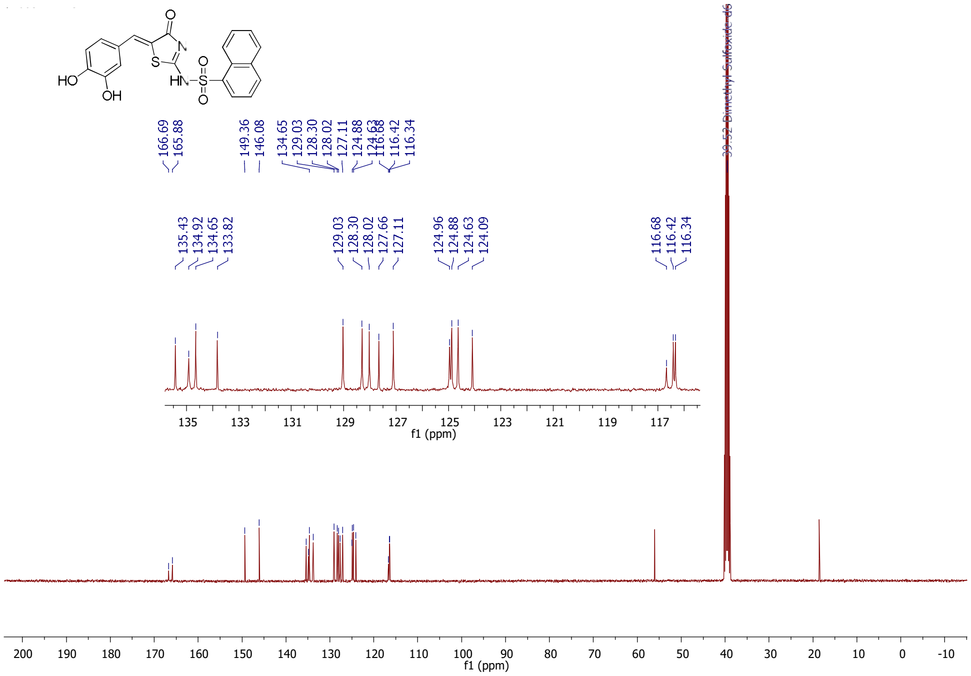
HO
OH
O
N
S
HN
S
O
O
166.69 165.88
149.36 146.08
134.65 129.03 128.30 128.02 127.11 124.88 124.63 116.68 116.42 116.34
39.52 Dimethyl Sulfoxide-d6
135.43 134.92 134.65 133.82
129.03 128.30 128.02 127.66 127.11
124.96 124.88 124.63 124.09
116.68 116.42 116.34
f1 (ppm)

LCMS Report

Agilent Technologies

Data file: D:\Chem32\1\Data\KP\KP_DS_NOV12 2020-11-06 10-08-02\006-16-KP9035.D
Sample name: KP9035
Description:
Sample amount: 0.000
Sample type: Sample

Instrument: LCMS
Location: 16
Injection date: 11/6/2020 10:32:39 AM
Injection: 1 of 1
Acq. method: LCMS ISOCRATIC 80% B_3 MINS.M
Injection volume: 2.000
Analysis method: LCMS ISOCRATIC 80%B_3 MINS.M
Acq. operator: SYSTEM
Last changed: 10/8/2020 2:52:31 PM

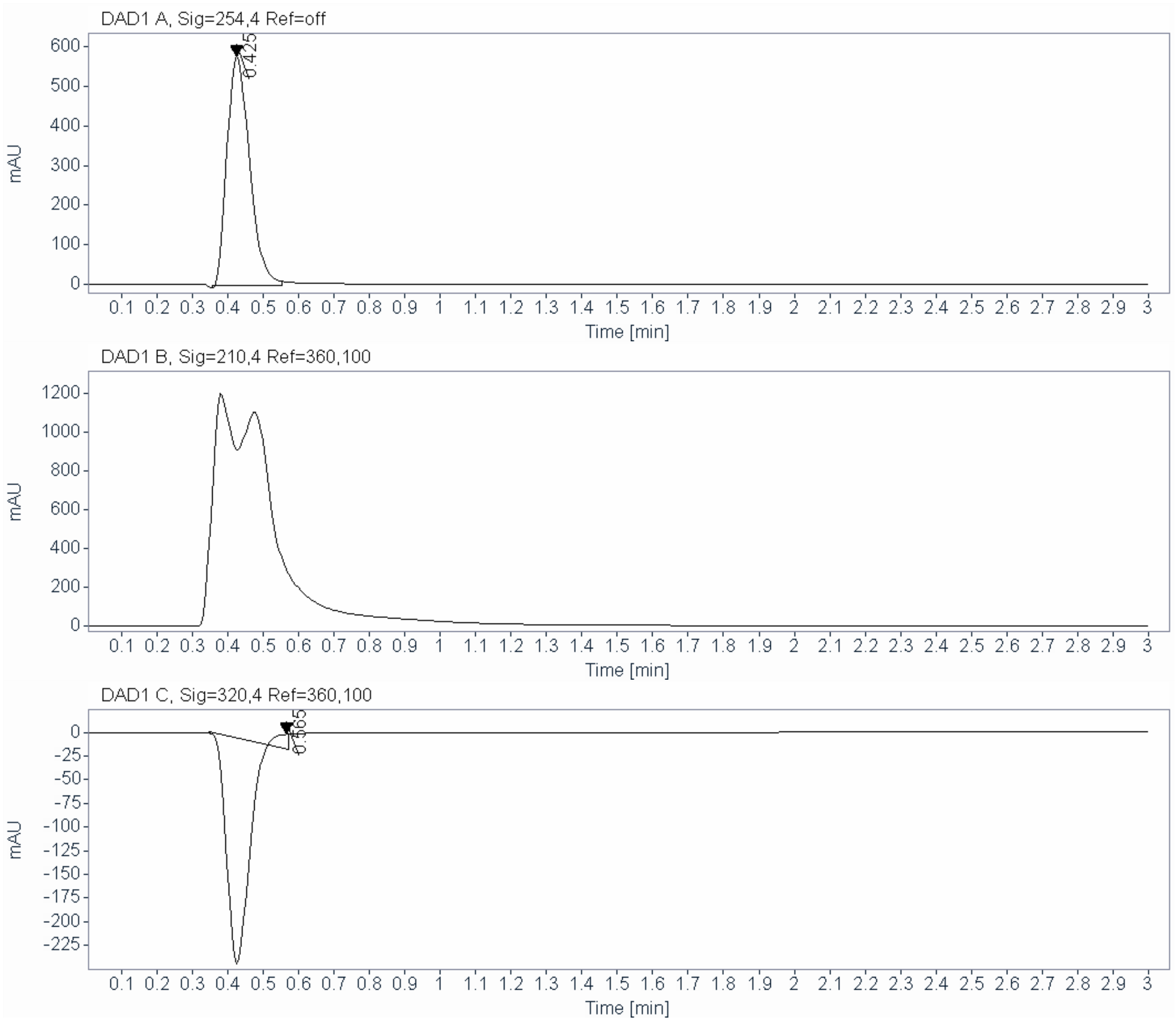

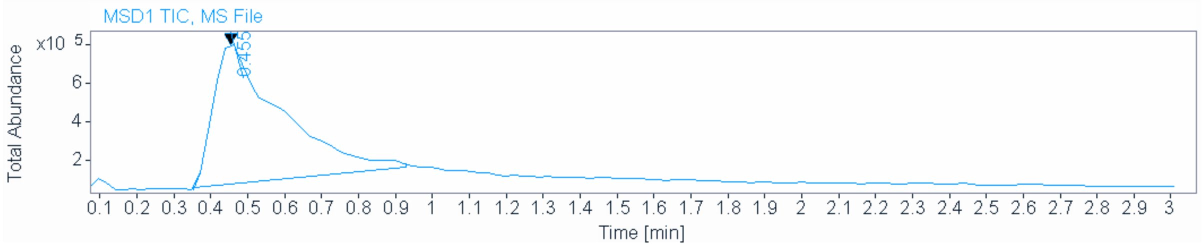

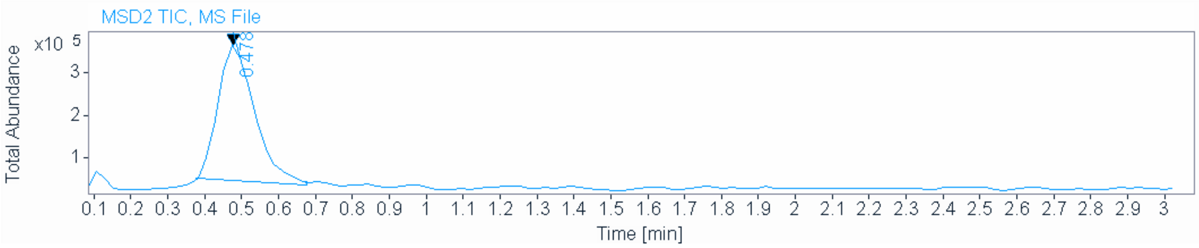

Signal: DAD1 A, Sig=254,4 Ref=off

RT [min]	Type	Width [min]	Area	Height	Area%	Name
0.425	MM	0.0732	2547.7766	580.0610	100.0000	
		Sum	2547.7766			

Signal: DAD1 C, Sig=320,4 Ref=360,100

RT [min]	Type	Width [min]	Area	Height	Area%	Name
0.565	MM	0.0437	41.7063	15.9059	100.0000	
		Sum	41.7063			

Signal: MSD1 TIC, MS File

RT [min]	Type	Width [min]	Area	Height	Area%	Name
0.455	MM	0.2091	9192923.000 0	732660.5625	100.0000	
		Sum	9192923.000			

Signal: MSD2 TIC, MS File

RT [min]	Type	Width [min]	Area	Height	Area%	Name
0.478	MM	0.1134	2181752.250 0	320567.0313	100.0000	
		Sum	2181752.250			

Agilent Technologies

0.455 - 0.463 (006-16-KP9035.D)

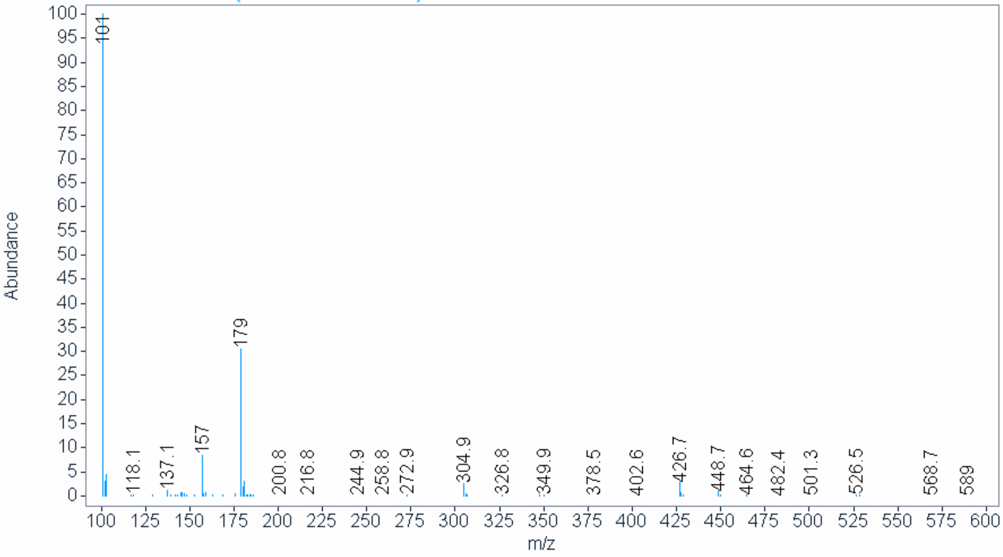

0.478 - 0.474 (006-16-KP9035.D)

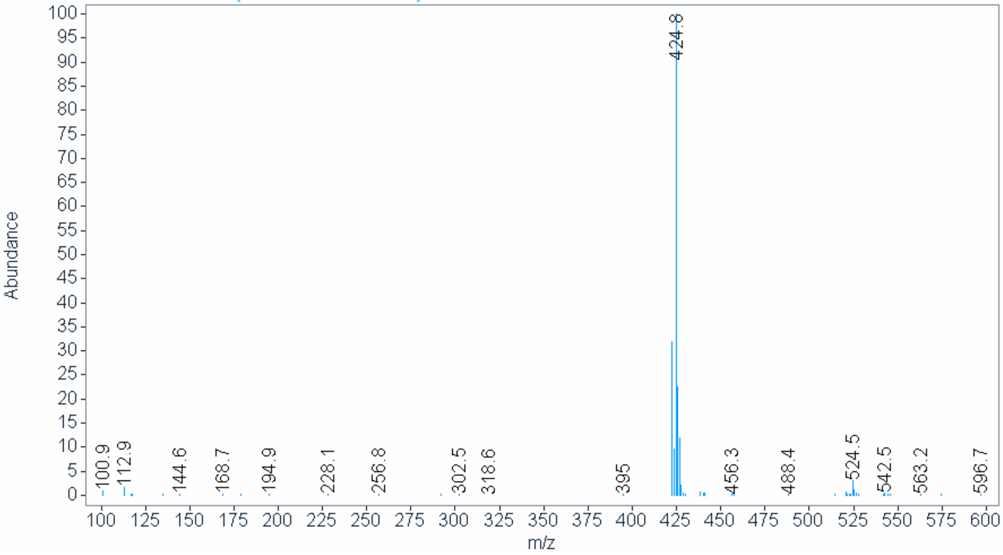

Compound Name: (*Z*)-*N*-(4-oxo-5-(2,3,4-trihydroxybenzylidene)-4,5-dihydrothiazol-2-yl)naphthalene-1-sulfonamide

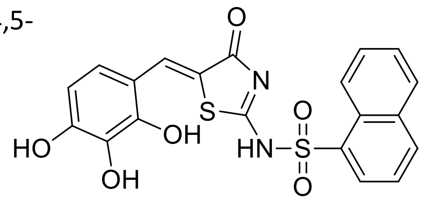

Chemical Formula: $C_{20}H_{14}N_2O_6S_2$
Exact Mass: 442.03
Molecular Weight: 442.46

Compound Code: 28 (KP9037)

Obtained Weight & Yield: 100 mg (46%)

Purity (by LCMS and ^{1}H NMR): > 99% by ^{1}H-NMR and LCMS

Appearance: dark yellow solid

Solubility: DMSO, slightly soluble in acetone and methanol.

Melting Point: > 199 °C (dec.)

TLC Rf (and conditions): N/A

IR Analysis (including assignment): IR (neat): ν_{max} = 3423 (OH), 2977, 2881, 2774 (C-C aromatic), 1688 (C=O), 1556 (C-C aromatic), 1333 (sulfonamide), 1223 (C-O), 1122 (C-N) cm^{-1}

^{1}H NMR Analysis: ^{1}H NMR (400 MHz, DMSO) δ 10.21 (s, 1H, OH), 9.58 (s, 1H, OH), 8.80 (s, 1H, OH), 8.60 (d, *J* = 8.5 Hz, 1H), 8.30 – 8.27 (m, 2H), 8.11 (d, *J* = 8.0 Hz, 1H), 7.96 (s, 1H), 7.76 (t, *J* = 7.3 Hz, 1H), 7.72 – 7.66 (m, 2H), 6.85 (d, *J* = 8.7 Hz, 1H), 6.58 (d, *J* = 8.7 Hz, 1H) ppm.
NH exchanging – not visible.
Some aldehyde starting material remaining (peaks in aromatic region, 0.69%)

^{13}C NMR Analysis: ^{13}C NMR (101 MHz, DMSO) δ 167.0 (br), 166.4 (br), 150.2, 148.0, 135.6, 134.5, 133.8, 133.2, 130.3 (br), 129.0, 128.2, 128.0, 127.7, 127.1, 124.9, 124.6, 120.2, 115.4 (br), 112.7, 108.4 ppm.

MS Analysis (low res): LRMS (ESI-) *m/z*: 441 (*M*-H, $C_{20}H_{13}N_2O_6S_2$, 100), 459 (*M*-H+H_2O, $C_{20}H_{15}N_2O_7S_2$, 100)

MS Analysis (high res): Exact mass calculated for $C_{20}H_{13}N_2O_6S_2$ [*M*-H]$^-$, 441.0200. Found 441.0219. Exact mass calculated for $C_{20}H_{11}N_2O_6S_2$ [*M*-3H]$^-$, 439.0100. Found 439.0064.

HPLC method details: Column: Zorbax SB-C18 Rapid Resolution HT 2.1x50mm 1.8-Micron; Method: LCMS ISOCRATIC 80%B_3 MINS.M filename: KP9037; Peak retention time: 0.403 mins; Area (%): 100.

Procedure: To a 10 mL microwave vial was added *N*-(4-oxo-4,5-dihydrothiazol-2-yl)naphthalene-1-sulfonamide (155 mg, 0.49 mmol), 2,3,4-trihydroxybenzaldehyde (117 mg, 0.54 mmol, 1.1 eq), ethanol (3 mL) and a catalytic amount of the benzoic acid/piperidine catalyst (approximately 5 drops). The suspension was heated by microwave irradiation (120 °C, 200 W) for 1 h. A precipitate formed upon addition at H_2O. The solid was collected by vacuum filtration to give the desired product as a dark yellow solid (100 mg, 46%).

Other analyses, reference papers, previously obtained data, comments, etc:

PerkinElmer Spectrum Version 10.4.00
Thursday, 26 November 2020 11:59 AM

Analyst research
Date Thursday, 26 November 2020 11:59 AM

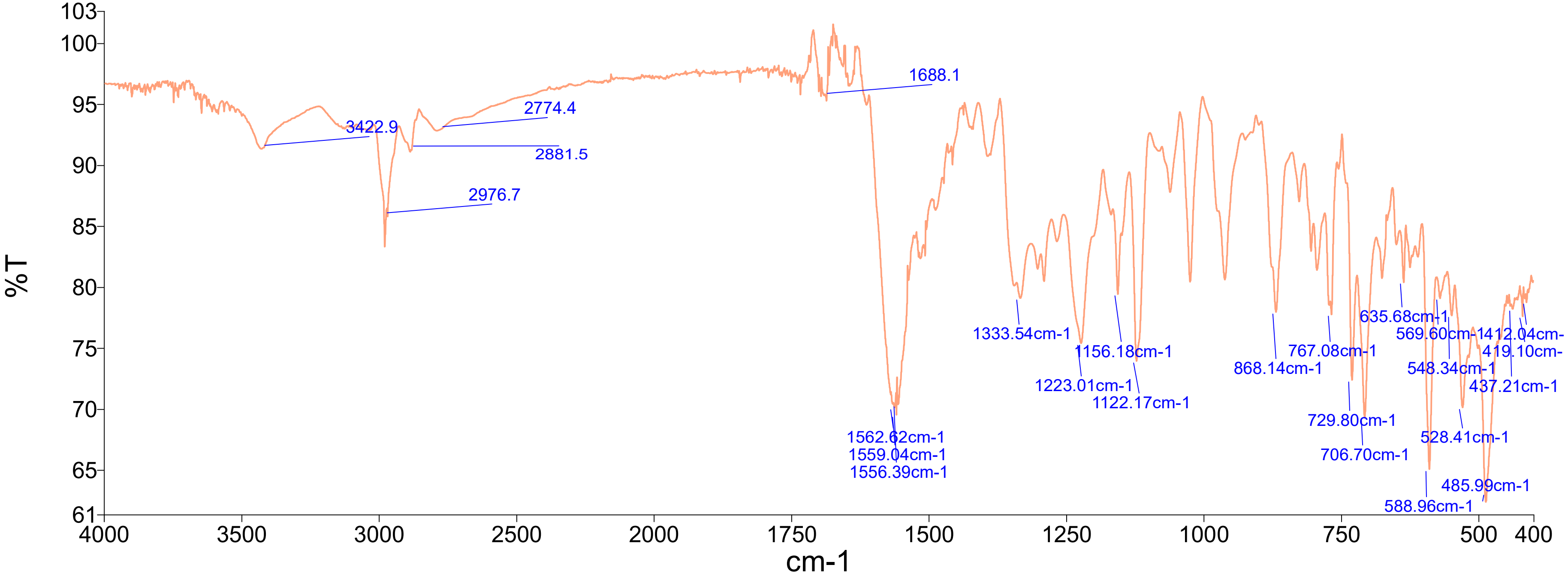

Sample Name	Description	Quality Checks
kp9037	Sample 187 By research Date Thursday, November 26 2020	The Quality Checks give rise to a Weak Bands warning for the sample.

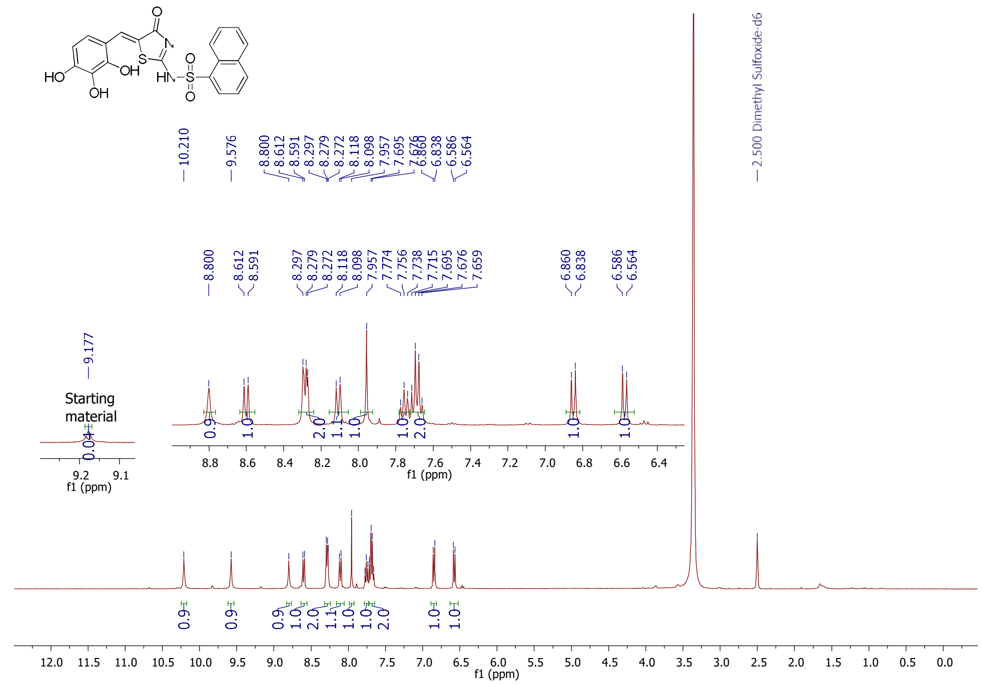
HO
OH
OH
S
O
HN
O
S
O
10.210
9.576
8.800
8.612
8.591
8.297
8.279
8.272
8.118
8.098
7.957
7.695
6.860
6.838
6.586
6.564
2.500 Dimethyl Sulfoxide-d6
9.177
Starting material
0.04
8.800
8.612
8.591
8.297
8.279
8.272
8.118
8.098
7.957
7.774
7.756
7.738
7.715
7.695
7.676
7.659
6.860
6.838
6.586
6.564
f1 (ppm)

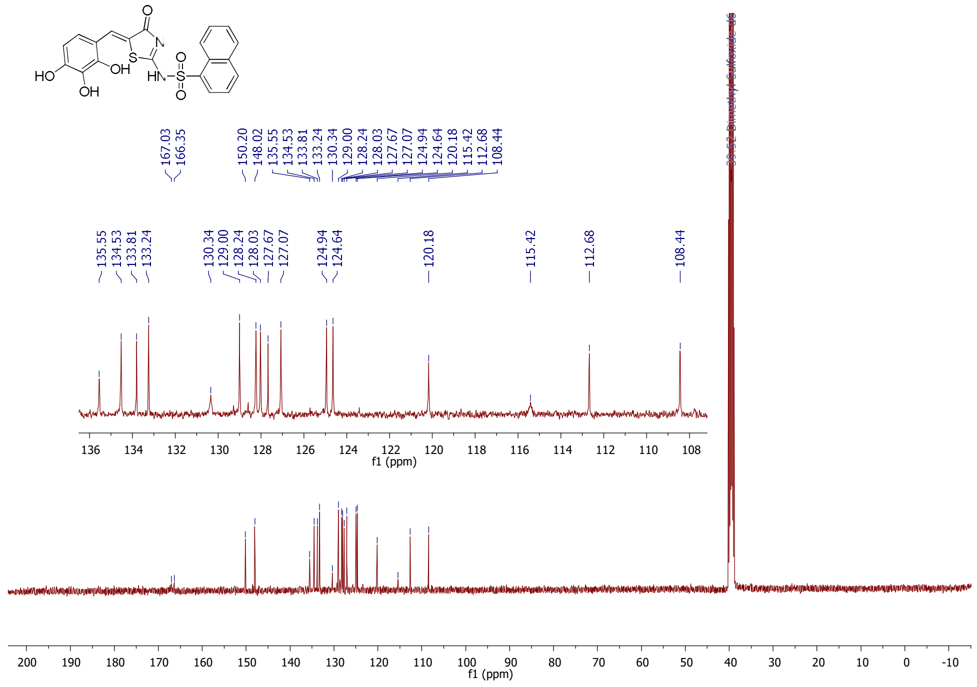
HO
OH
OH
S
O
HN
O
S
O
167.03
166.35
150.20
148.02
135.55
134.53
133.81
133.24
130.34
129.00
128.24
128.03
127.67
127.07
124.94
124.64
120.18
115.42
112.68
108.44
Dimethyl Sulfoxide-d6
f1 (ppm)

LCMS Report

Agilent Technologies

**Data file:**	D:\Chem32\1\Data\KP\KP_DS_NOV12 2020-11-06 10-08-02\003-19-KP9037.D		
**Sample name:**	KP9037		
**Description:**			
**Sample amount:**	0.000	**Sample type:**	Sample
**Instrument:**	LCMS	**Location:**	19
**Injection date:**	11/6/2020 10:18:54 AM	**Injection:**	1 of 1
**Acq. method:**	LCMS ISOCRATIC 80% B_3 MINS.M	**Injection volume:**	2.000
**Analysis method:**	LCMS ISOCRATIC 80%B_3 MINS.M	**Acq. operator:**	SYSTEM
**Last changed:**	10/8/2020 2:52:31 PM		

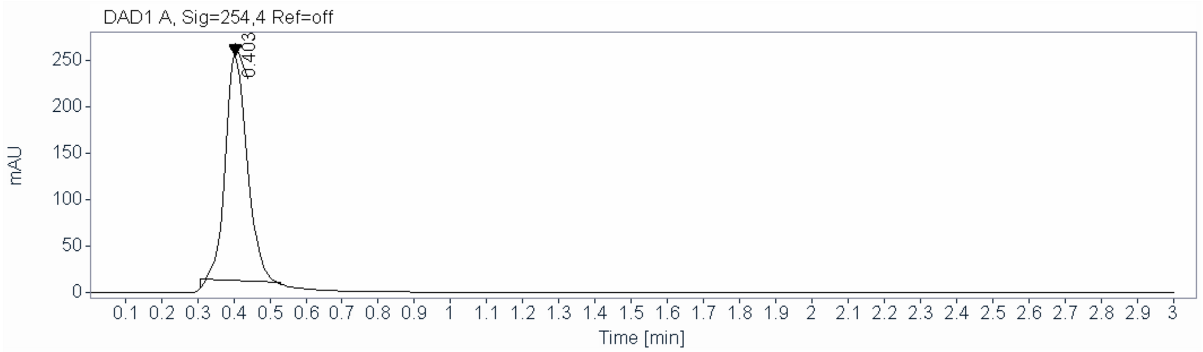

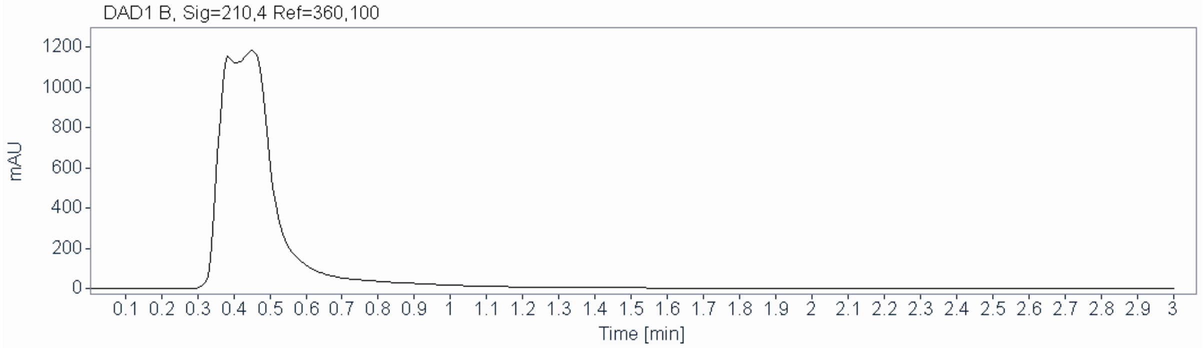

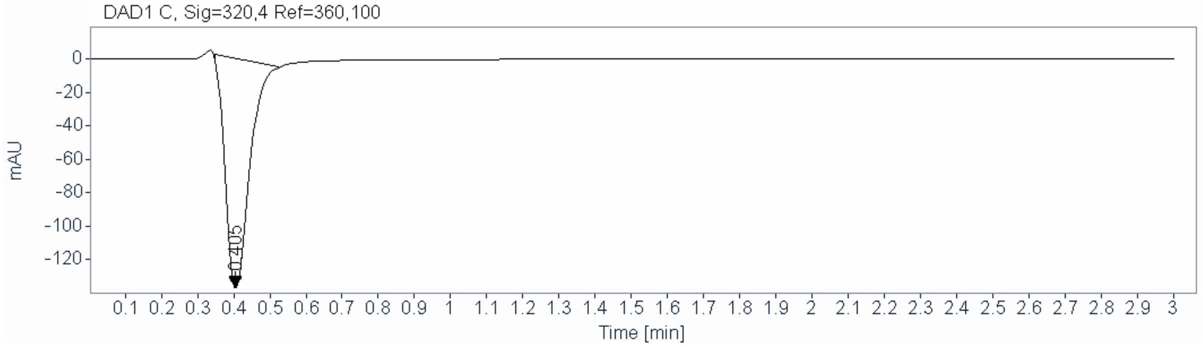

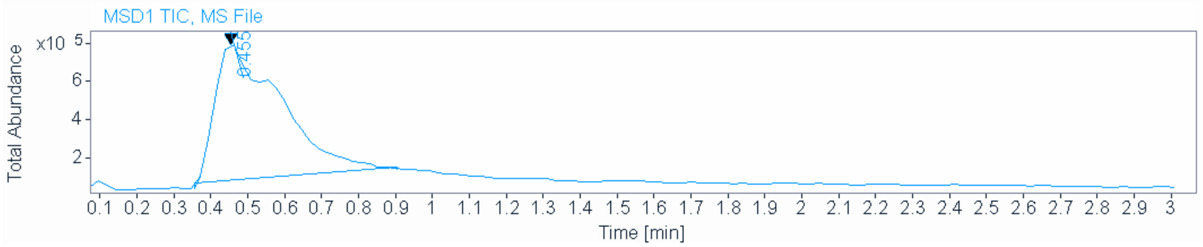

LCMS Report

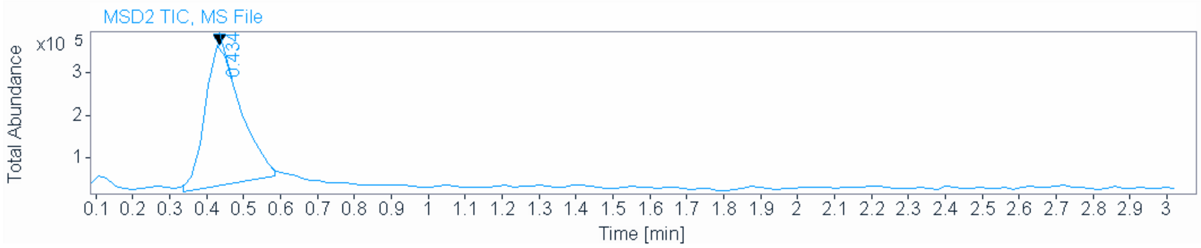

Signal: DAD1 A, Sig=254,4 Ref=off

RT [min]	Type	Width [min]	Area	Height	Area%	Name
0.403	MM	0.0699	1019.0309	242.9025	100.0000	
		Sum	1019.0309			

Signal: DAD1 C, Sig=320,4 Ref=360,100

RT [min]	Type	Width [min]	Area	Height	Area%	Name
0.405	PP N	0.0709	590.6592	138.8404	100.0000	
		Sum	590.6592			

Signal: MSD1 TIC, MS File

RT [min]	Type	Width [min]	Area	Height	Area%	Name
0.455	MM	0.2026	8749822.000 0	719960.3125	100.0000	
		Sum	8749822.000			

Signal: MSD2 TIC, MS File

RT [min]	Type	Width [min]	Area	Height	Area%	Name
0.434	MM	0.1075	2190222.000 0	339414.5938	100.0000	
		Sum	2190222.000			

0.434 - 0.428 (003-19-KP9037.D)

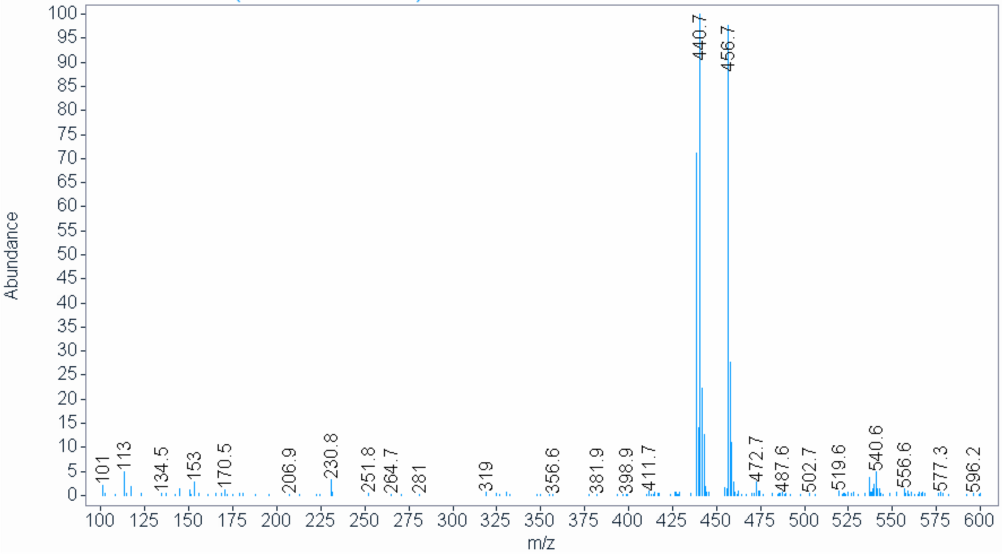

0.455 - 0.463 (003-19-KP9037.D)

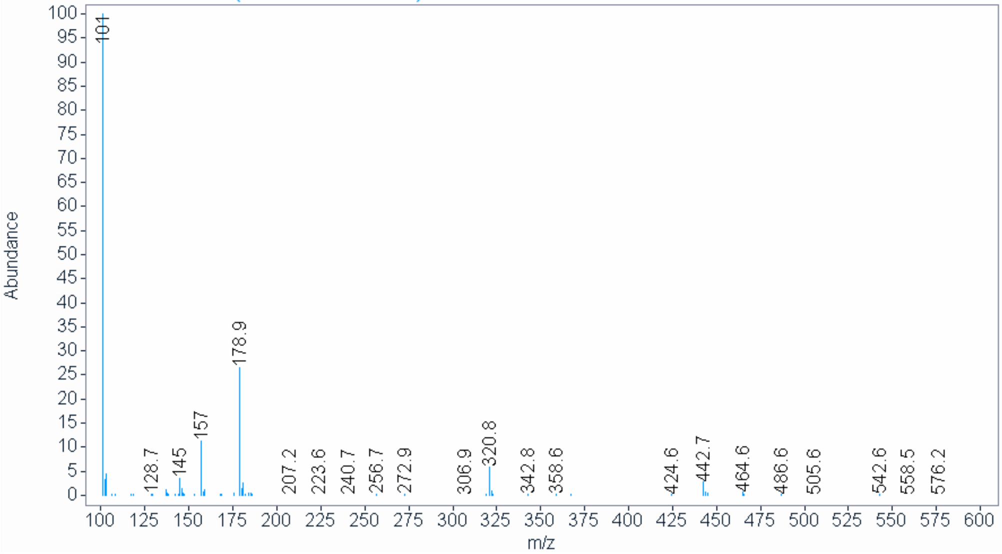

Compound Name: (*Z*)-*N*-(5-(2,3-dimethoxybenzylidene)-4-oxo-4,5-dihydrothiazol-2-yl)naphthalene-1-sulfonamide

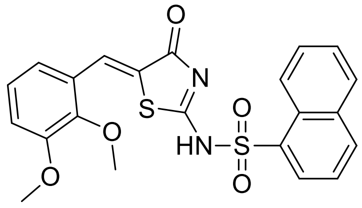
Chemical Formula: $C_{22}H_{18}N_2O_5S_2$
Exact Mass: 454.07
Molecular Weight: 454.52

Compound Code: 29 (KP9005)

Obtained Weight & Yield: 188 mg (84%)

Purity (by LCMS and ^{1}H NMR): > 99% by ^{1}H-NMR and LCMS

Appearance: yellow solid

Solubility: DMSO, slightly soluble in acetone and methanol.

Melting Point: > 263 °C (dec.)

TLC Rf (and conditions): N/A

IR Analysis (including assignment): IR (neat): ν_{max} = 3161 (N-H), 3060, 2977 (C-H aromatic), 2882, 2792 (C-H alkyl), 1700 (C=O), 1563 (C-C aromatic), 1300 (sulfonamide), 1268 (ether), 1124 (C-N) cm^{-1}

^{1}H NMR Analysis: ^{1}H NMR (600 MHz, DMSO) δ 13.18 (s, 1H, br, NH), 8.62 (d, *J* = 8.6 Hz, 1H), 8.31 – 8.29 (m, 2H), 8.11 (d, *J* = 8.1 Hz, 1H), 7.86 (s, 1H), 7.77 (t, *J* = 7.7 Hz, 1H), 7.72 – 7.67 (m, 2H), 7.29 (t, *J* = 7.9 Hz, 1H), 7.24 (d, *J* = 8.1 Hz, 1H), 7.13 (d, *J* = 7.6 Hz, 1H), 3.86 (s, 3H), 3.79 (s, 3H) ppm.
Ethanol at 1.06 ppm (0.27%).

^{13}C NMR Analysis: ^{13}C DEPTQ (151 MHz, DMSO) δ 166.5, 165.8, 152.8, 148.2, 135.2, 134.7, 133.8, 129.0, 128.4, 128.3, 128.1, 127.6, 127.1, 126.4, 124.92, 124.86, 124.6, 122.9, 120.3, 115.9, 61.2, 56.0 ppm.

MS Analysis (low res): LRMS (ESI-) *m/z*: 453 (*M*-H, $C_{22}H_{17}N_2O_5S_2$, 100); (ESI+) *m/z*: 455 (*M*+H, $C_{22}H_{19}N_2O_5S_2$, 100);

MS Analysis (high res): Exact mass calculated for $C_{22}H_{17}N_2O_5S_2$ $[M\text{-}H]^-$, 453.0600. Found 453.0585.

HPLC method details: Column: Zorbax SB-C18 Rapid Resolution HT 2.1x50mm 1.8-Micron; Method: LCMS ISOCRATIC 60%B_3 MINS.M filename: KP9005; Peak retention time: 1.799 mins; Area (%): 100.

Procedure: To a 10mL microwave vial was added N-(4-oxo-4,5-dihydrothiazol-2-yl)naphthalene-1-sulfonamide (156 mg, 0.49 mmol), 2,3-dimethoxybenzaldehyde (101 mg, 0.54 mmol, 1.1 eq), ethanol (3 mL) and a catalytic amount of the benzoic acid/piperidine catalyst (approximately 5 drops). The suspension was heated by microwave irradiation (120 °C, 200 W) for 30 min. A precipitate formed upon cooling and was collected to give the desired product as a yellow solid (188 mg, 84%).

Other analyses, reference papers, previously obtained data, comments, etc:

PerkinElmer Spectrum Version 10.4.00
Thursday, 26 November 2020 11:55 AM

Analyst research
Date Thursday, 26 November 2020 11:55 AM

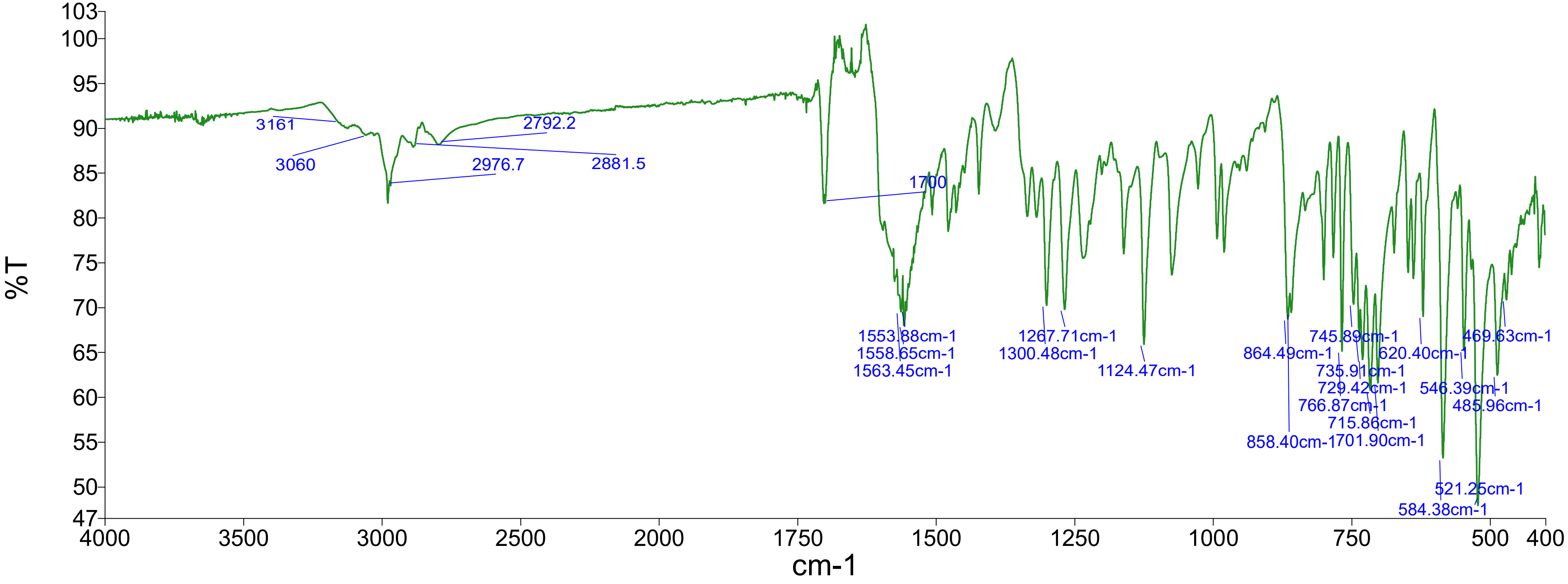

Sample Name	Description	Quality Checks
kp9005	Sample 179 By research Date Thursday, November 26 2020	The Quality Checks do not report any warnings for the sample.

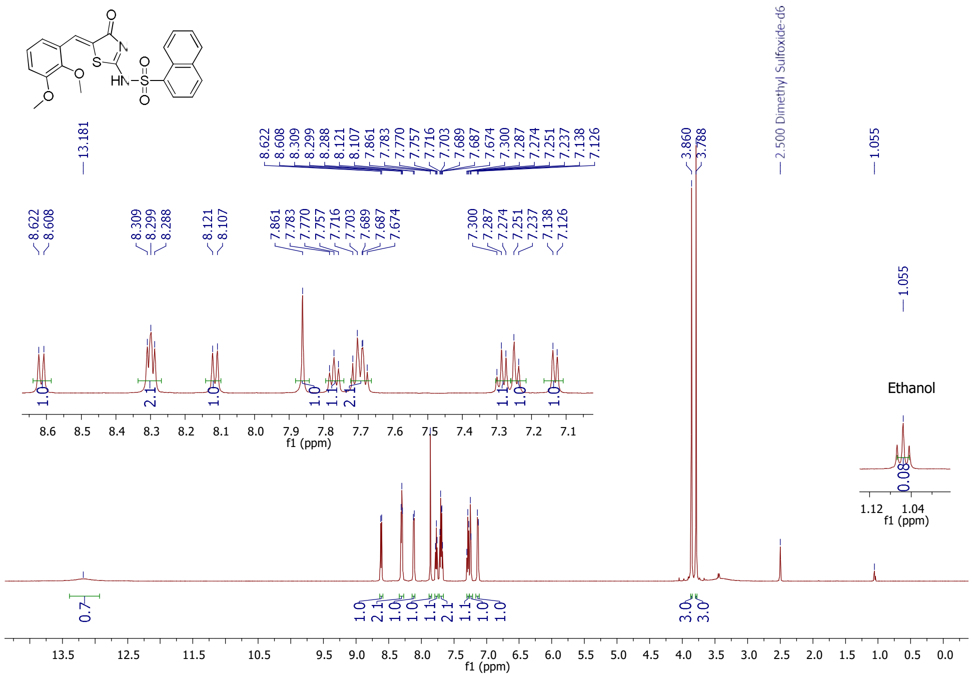
13.181
8.622
8.608
8.309
8.299
8.288
8.121
8.107
7.861
7.783
7.770
7.757
7.716
7.703
7.689
7.687
7.674
7.300
7.287
7.274
7.251
7.237
7.138
7.126
3.860
3.788
2.500 Dimethyl Sulfoxide-d6
1.055
Ethanol
0.08
1.0
2.1
1.0
1.0
1.1
2.1
1.1
1.0
1.0
0.7
3.0
3.0
f1 (ppm)

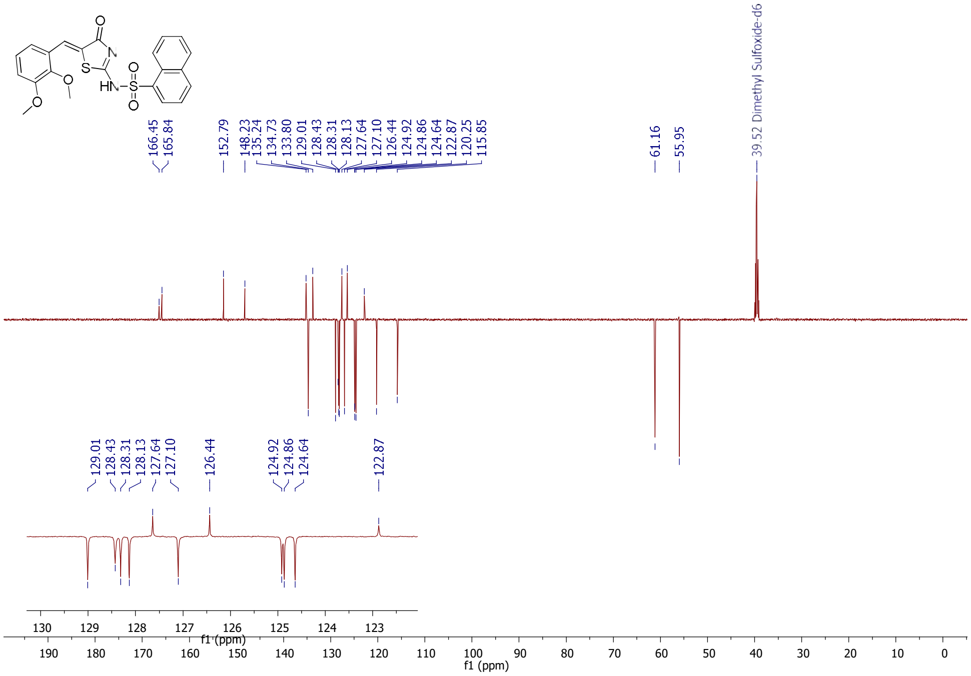
166.45
165.84
152.79
148.23
135.24
134.73
133.80
129.01
128.43
128.31
128.13
127.64
127.10
126.44
124.92
124.86
124.64
122.87
120.25
115.85
61.16
55.95
39.52 Dimethyl Sulfoxide-d6
f1 (ppm)

LCMS Report

Data file: D:\Chem32\1\Data\KP\KP_DS_NOV1 2020-11-02 12-21-40\004-18-KP9005.D
Sample name: KP9005
Description:
Sample amount: 0.000
Sample type: Sample

Instrument: LCMS
Location: 18
Injection date: 11/2/2020 12:38:25 PM
Injection: 1 of 1
Acq. method: LCMS ISOCRATIC 60% B_3MINS.M
Injection volume: 2.000
Analysis method: LCMS ISOCRATIC 60%B_3MINS.M
Acq. operator: SYSTEM
Last changed: 5/19/2016 3:52:53 PM

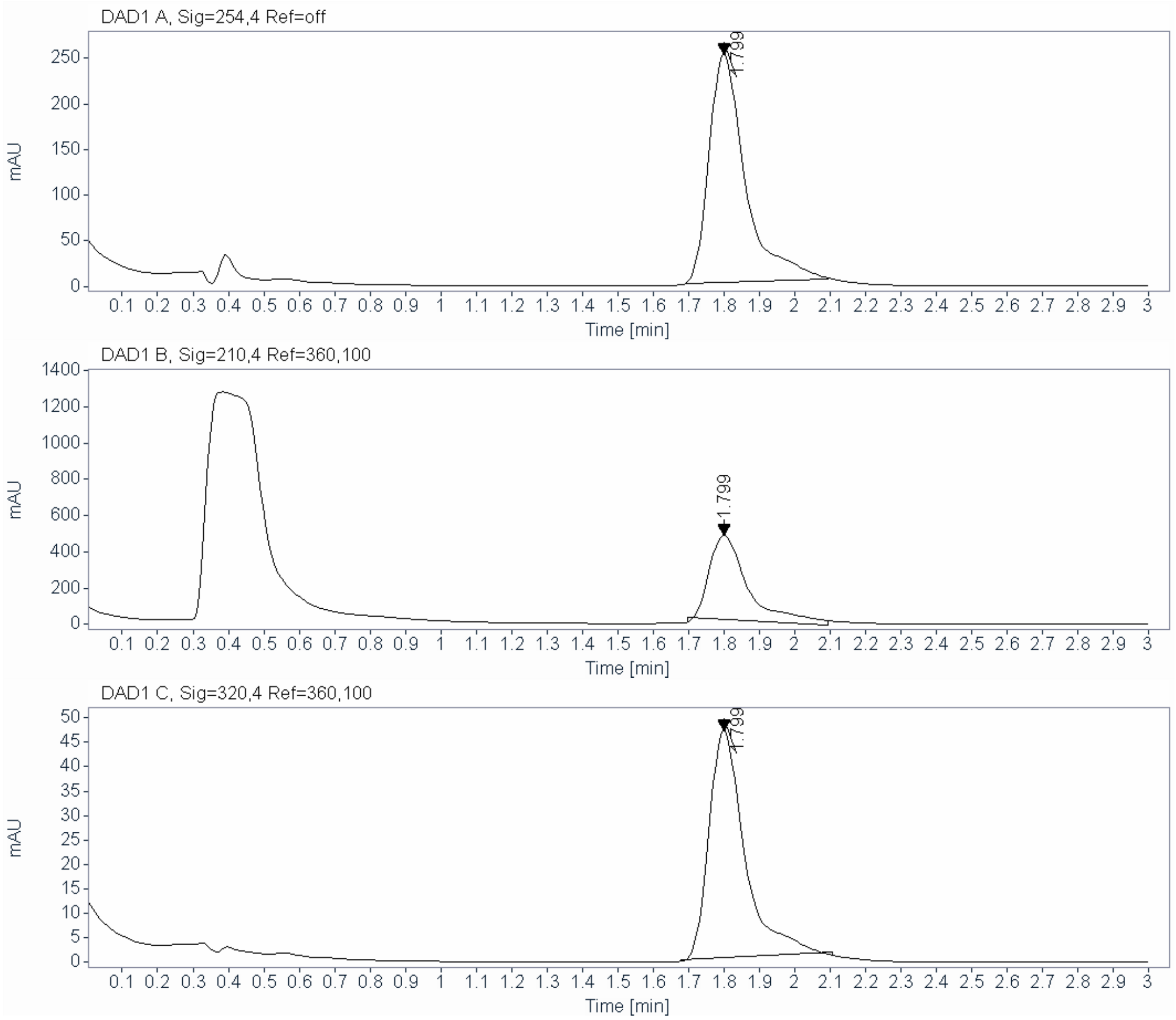

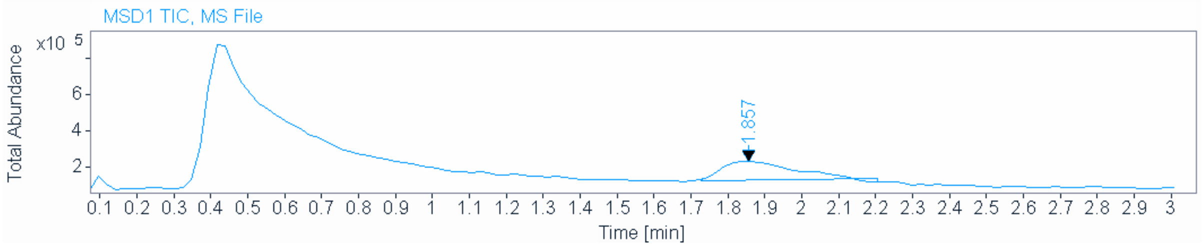

Agilent Technologies

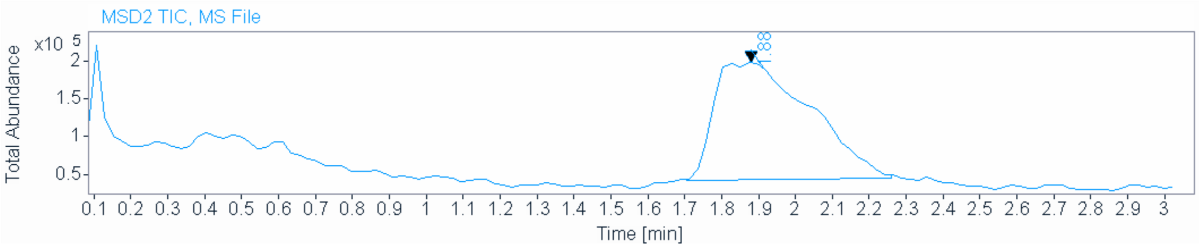

Signal: DAD1 A, Sig=254,4 Ref=off

RT [min]	Type	Width [min]	Area	Height	Area%	Name
1.799	MM	0.1189	1788.5698	250.7264	100.0000	
		Sum	1788.5698			

Signal: DAD1 B, Sig=210,4 Ref=360,100

RT [min]	Type	Width [min]	Area	Height	Area%	Name
1.799	MM	0.1255	3505.8523	465.4037	100.0000	
		Sum	3505.8523			

Signal: DAD1 C, Sig=320,4 Ref=360,100

RT [min]	Type	Width [min]	Area	Height	Area%	Name
1.799	MM	0.1162	324.1618	46.4968	100.0000	
		Sum	324.1618			

Signal: MSD1 TIC, MS File

RT [min]	Type	Width [min]	Area	Height	Area%	Name
1.857	MM	0.2234	1438169.125 0	107293.5625	100.0000	
		Sum	1438169.125			

Signal: MSD2 TIC, MS File

RT [min]	Type	Width [min]	Area	Height	Area%	Name
1.880	MM	0.2996	2800535.000 0	155788.7969	100.0000	
		Sum	2800535.000			

Agilent Technologies

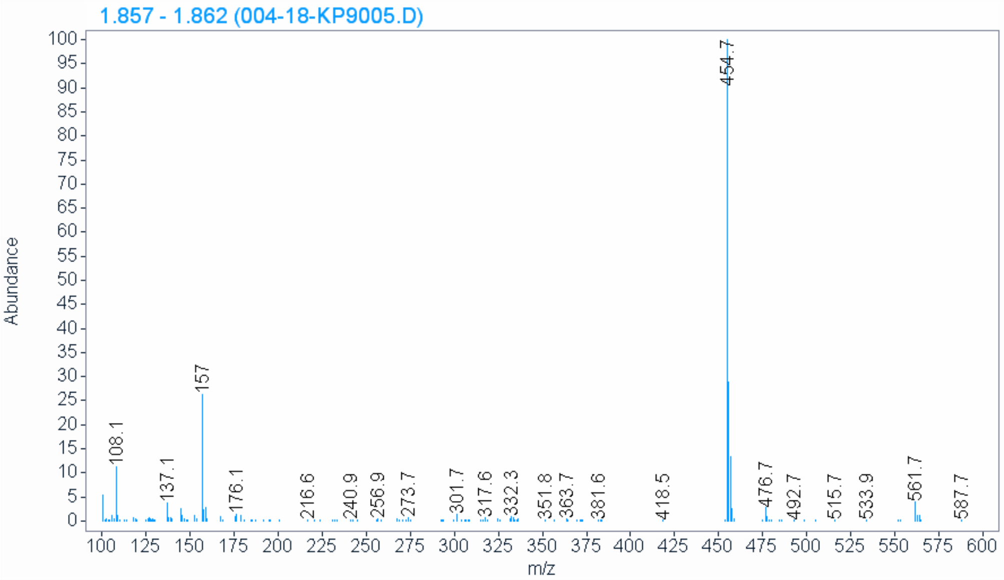

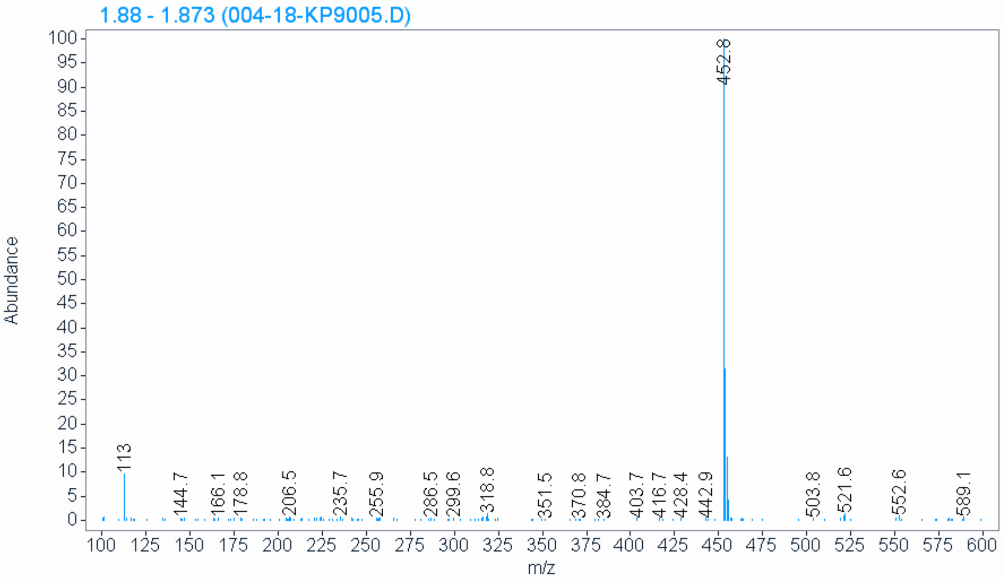

Compound Name: (*Z*)-*N*-(5-(2,4-dimethoxybenzylidene)-4-oxo-4,5-dihydrothiazol-2-yl)naphthalene-1-sulfonamide

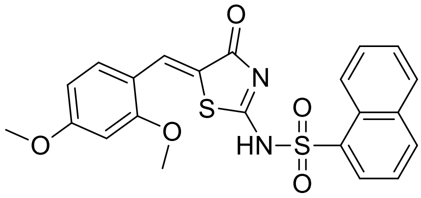

Chemical Formula: $C_{22}H_{18}N_2O_5S_2$
Exact Mass: 454.07
Molecular Weight: 454.52

Compound Code: 30 (KP9006)

Obtained Weight & Yield: 185 mg (83%)

Purity (by LCMS and ^{1}H NMR): > 98% by ^{1}H-NMR and LCMS

Appearance: bright orange solid

Solubility: DMSO, slightly soluble in acetone and methanol.

Melting Point: > 255 °C (dec.)

TLC Rf (and conditions): N/A

IR Analysis (including assignment): IR (neat): ν_{max} = 2989, 2887 (C-H aromatic), 2786 (C-H alkyl), 1682 (C=O), 1557 (C=C aromatic), 1268 (ether), 1126 (C-N) cm^{-1}

^{1}H NMR Analysis: ^{1}H NMR (600 MHz, DMSO) δ 13.02 (s, 1H, br, NH), 8.61 (d, *J* = 8.5 Hz, 1H), 8.32 – 8.29 (m, 2H), 8.12 (d, *J* = 8.0 Hz, 1H), 7.85 (s, 1H), 7.77 (t, *J* = 7.6 Hz, 1H), 7.73 – 7.67 (m, 2H), 7.44 (d, *J* 2= 8.6 Hz, 1H), 6.73 (d, *J* = 8.6 Hz, 1H), 6.69 (s, 1H), 3.90 (s, 3H), 3.84 (s, 3H) ppm.
Ethanol at 1.06 ppm (0.77%)

^{13}C NMR Analysis: ^{13}C NMR (151 MHz, DMSO) δ 166.6, 166.0, 163.7, 160.0, 135.4, 134.6, 133.8, 131.1, 129.0, 128.9, 128.3, 128.1, 127.7, 127.1, 124.9, 124.6, 117.8, 114.0, 106.8, 98.7, 56.0, 55.7 ppm.

MS Analysis (low res): LRMS (ESI-) *m/z*: 453 (*M*-H, $C_{22}H_{17}N_2O_5S_2$, 100); (ESI+) *m/z*: 455 (*M*+H, $C_{22}H_{19}N_2O_5S_2$, 100)

MS Analysis (high res): Exact mass calculated for $C_{22}H_{17}N_2O_5S_2$ [*M*-H]$^-$, 453.0600. Found 453.0586.

HPLC method details: Column: Zorbax SB-C18 Rapid Resolution HT 2.1x50mm 1.8-Micron; Method: LCMS ISOCRATIC 60%B_3 MINS.M filename: KP9004; Peak retention time: 1.925 mins; Area (%): 100.

Procedure: To a 10mL microwave vial was added N-(4-oxo-4,5-dihydrothiazol-2-yl)naphthalene-1-sulfonamide (152 mg, 0.49 mmol), 2,4-dimethoxybenzaldehyde (93 mg, 0.54 mmol, 1.1 eq), ethanol (3 mL) and a catalytic amount of the benzoic acid/piperidine catalyst (approximately 5 drops). The suspension was heated by microwave irradiation (120 °C, 200 W) for 30 min. A precipitate formed upon cooling and the precipitate was collected to give the desired product as a bright orange solid (185 mg, 83%).

Other analyses, reference papers, previously obtained data, comments, etc:

PerkinElmer Spectrum Version 10.4.00
Thursday, 26 November 2020 11:55 AM

Analyst research
Date Thursday, 26 November 2020 11:55 AM

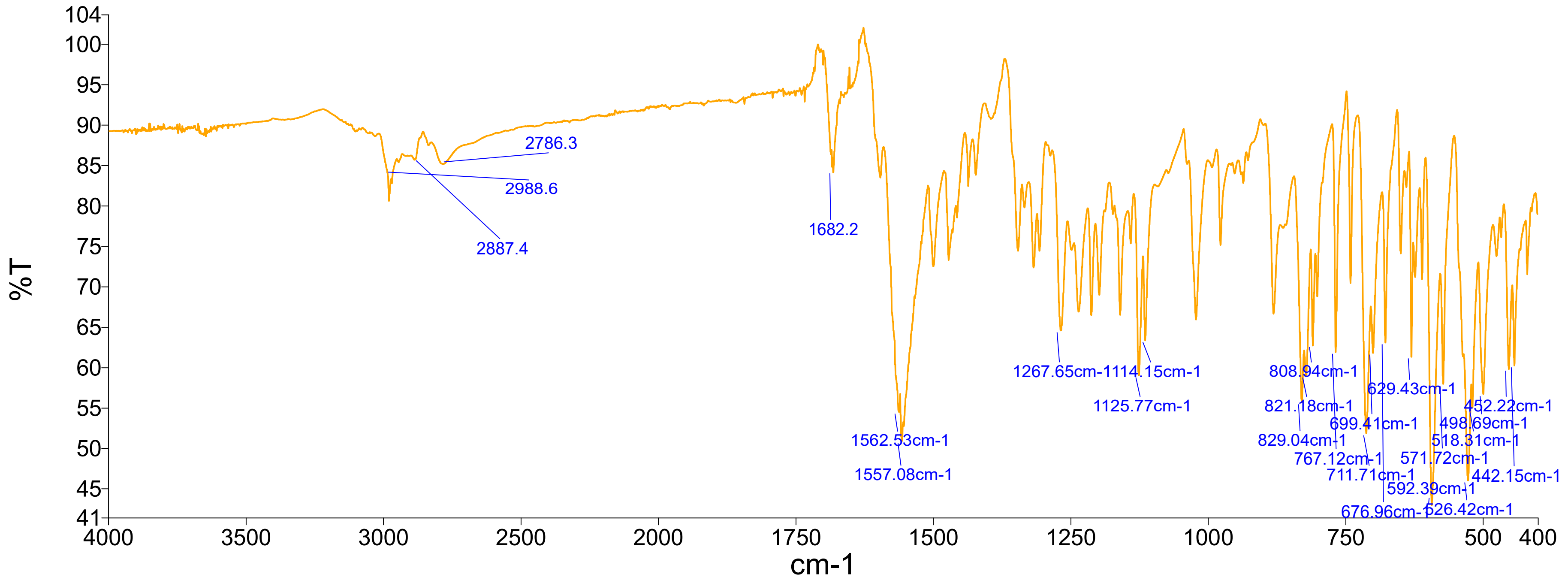

Sample Name	Description	Quality Checks
kp9006	Sample 180 By research Date Thursday, November 26 2020	The Quality Checks do not report any warnings for the sample.

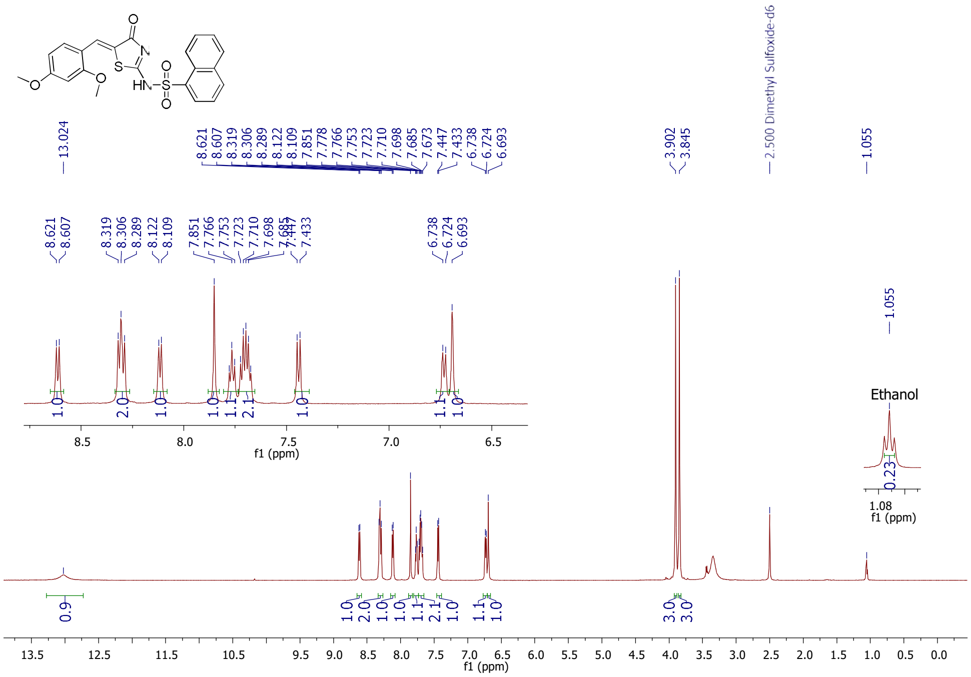

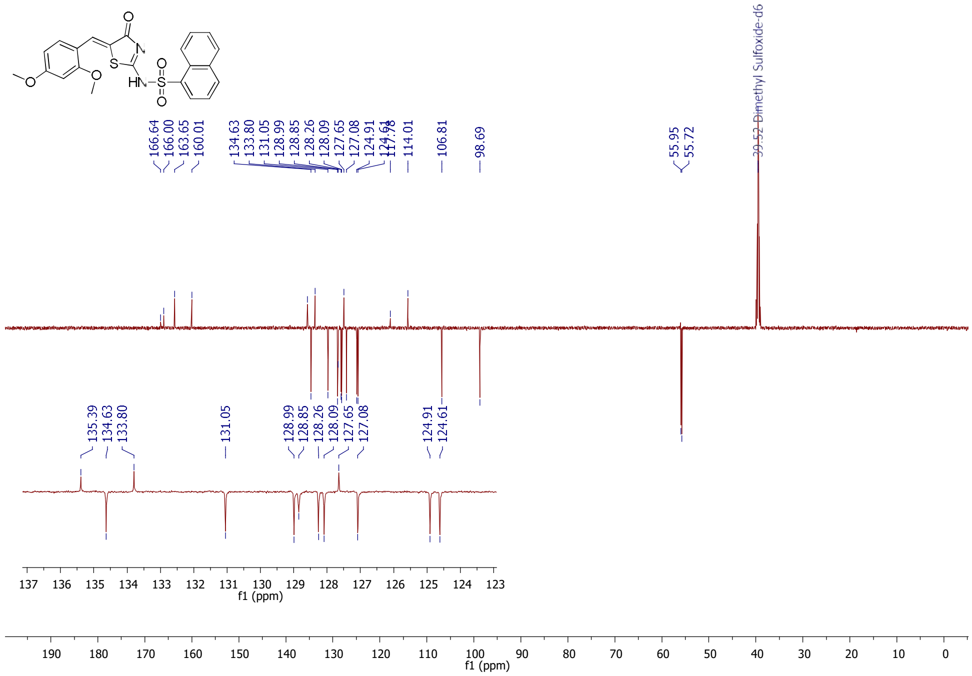

LCMS Report

Agilent Technologies

**Data file:**	D:\Chem32\1\Data\KP\KP_DS_NOV1 2020-11-02 12-21-40\005-17-KP9006.D		
**Sample name:**	KP9006		
**Description:**			
**Sample amount:**	0.000	**Sample type:**	Sample
**Instrument:**	LCMS	**Location:**	17
**Injection date:**	11/2/2020 12:42:58 PM	**Injection:**	1 of 1
**Acq. method:**	LCMS ISOCRATIC 60% B_3MINS.M	**Injection volume:**	2.000
**Analysis method:**	LCMS ISOCRATIC 60%B_3MINS.M	**Acq. operator:**	SYSTEM
**Last changed:**	5/19/2016 3:52:53 PM		

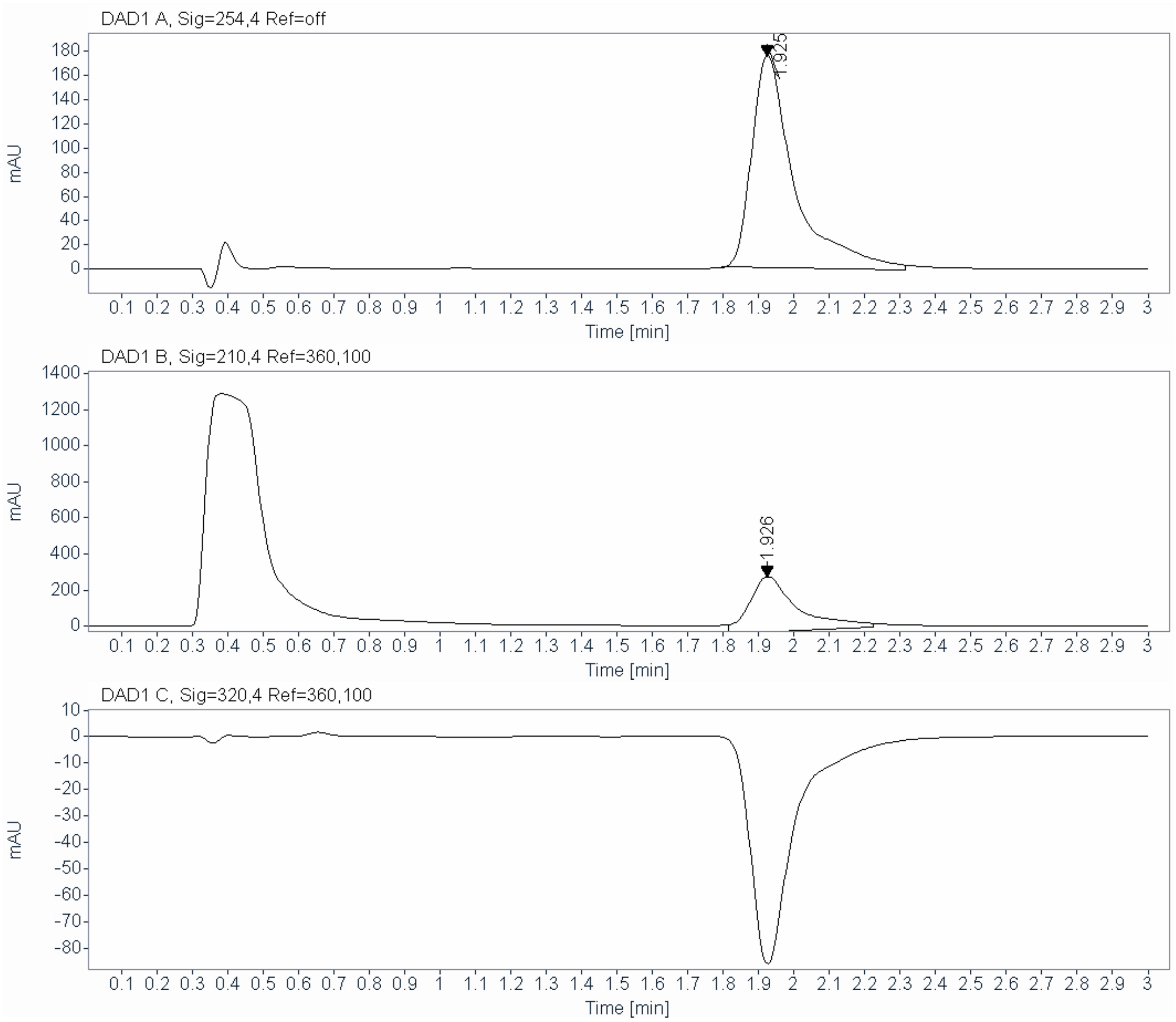

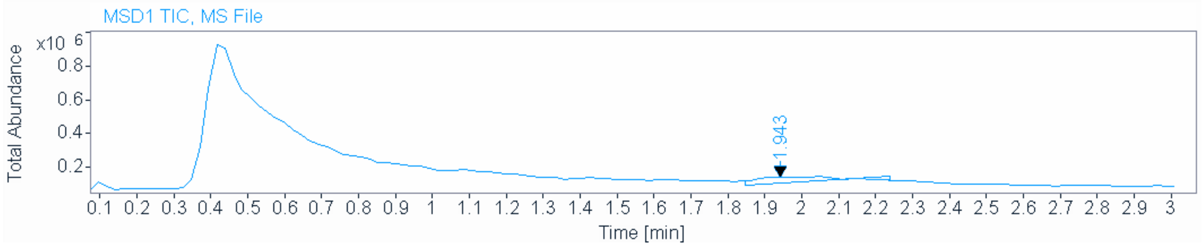

Agilent Technologies

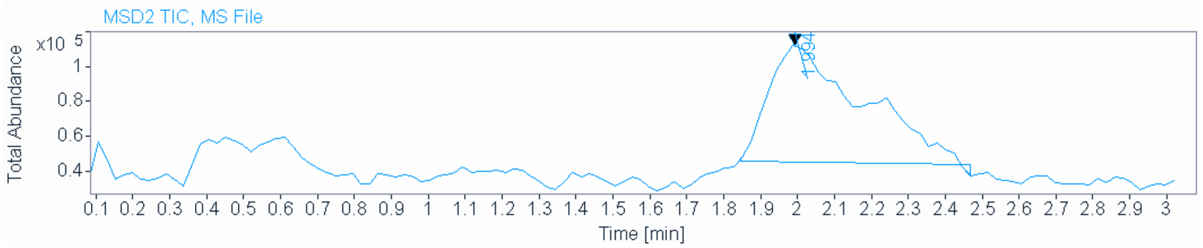

Signal: DAD1 A, Sig=254,4 Ref=off

RT [min]	Type	Width [min]	Area	Height	Area%	Name
1.925	MM	0.1399	1470.9150	175.2640	100.0000	
		Sum	1470.9150			

Signal: DAD1 B, Sig=210,4 Ref=360,100

RT [min]	Type	Width [min]	Area	Height	Area%	Name
1.926	MM	0.1592	2942.0510	307.9693	100.0000	
		Sum	2942.0510			

Signal: MSD1 TIC, MS File

RT [min]	Type	Width [min]	Area	Height	Area%	Name
1.943	MM	0.1742	431405.0938	41279.6445	100.0000	
		Sum	431405.0938			

Signal: MSD2 TIC, MS File

RT [min]	Type	Width [min]	Area	Height	Area%	Name
1.994	MM	0.2945	1192883.625 0	67513.9609	100.0000	
		Sum	1192883.625			

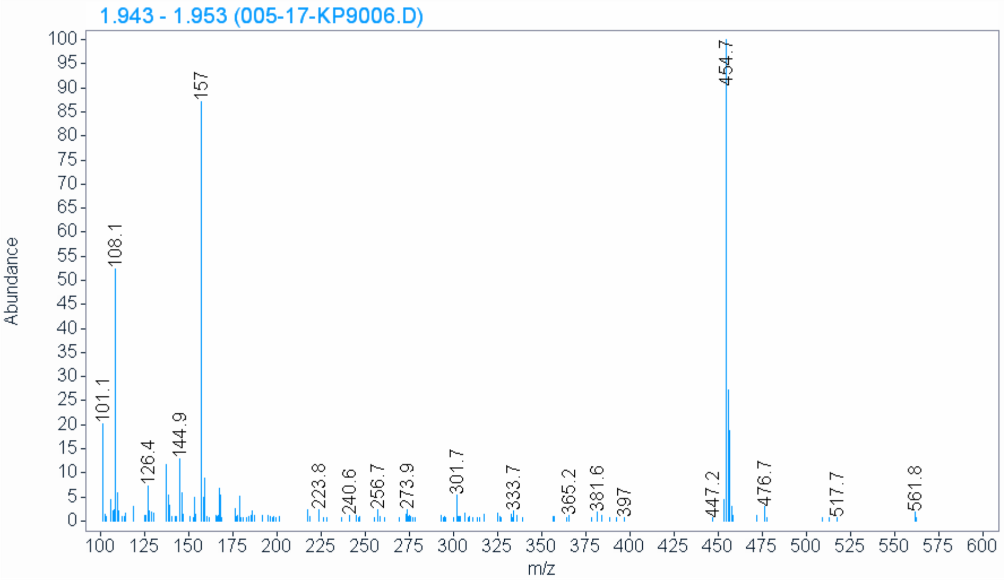

1.943 - 1.953 (005-17-KP9006.D)
Abundance
m/z
101.1
108.1
126.4
144.9
157
223.8
240.6
256.7
273.9
301.7
333.7
365.2
381.6
397
447.2
454.7
476.7
517.7
561.8

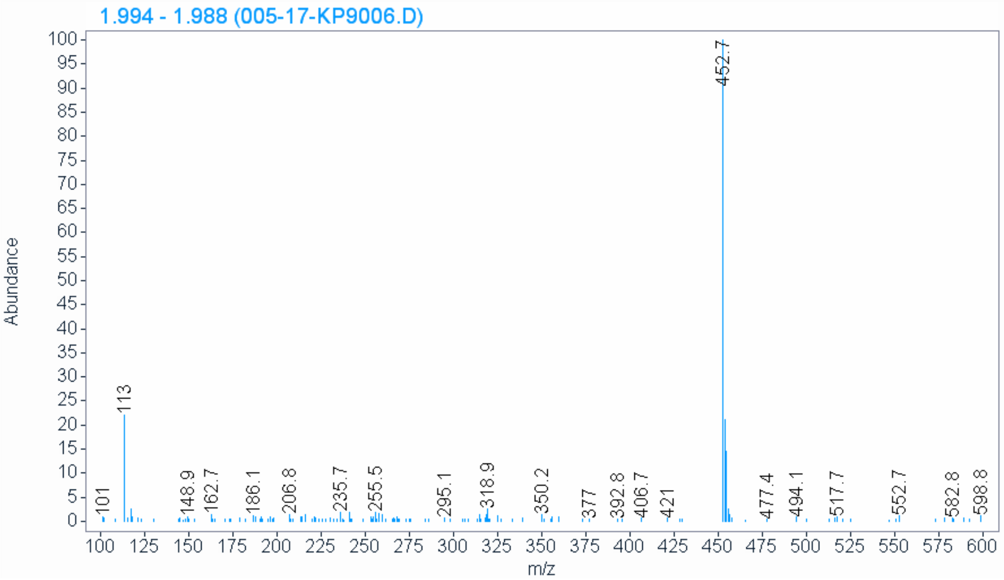

1.994 - 1.988 (005-17-KP9006.D)
Abundance
m/z
101
113
148.9
162.7
186.1
206.8
235.7
255.5
295.1
318.9
350.2
377
392.8
406.7
421
452.7
477.4
494.1
517.7
552.7
582.8
598.8

Compound Name: (*Z*)-*N*-(5-(3,4-dimethoxybenzylidene)-4-oxo-4,5-dihydrothiazol-2-yl)naphthalene-1-sulfonamide

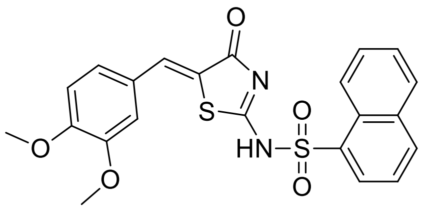

Chemical Formula: $C_{22}H_{18}N_2O_5S_2$
Exact Mass: 454.07
Molecular Weight: 454.52

Compound Code: 31 (KP9007)

Obtained Weight & Yield: 163 mg (73%)

Purity (by LCMS and ^{1}H NMR): > 99% by ^{1}H-NMR and LCMS

Appearance: bright yellow/orange solid

Solubility: DMSO, slightly soluble in acetone and methanol.

Melting Point: > 255 °C (dec.)

TLC Rf (and conditions): N/A

IR Analysis (including assignment): IR (neat): ν_{max} = 2996, 2905 (C-H aromatic), 2768 (C-H alkyl), 1694 (C=O), 1506 (C-C aromatic), 1300 (sulfonamide), 1126 (C-N) cm^{-1}

^{1}H NMR Analysis: ^{1}H NMR (600 MHz, DMSO) δ 13.10 (s, 1H, br, NH), 8.62 (d, *J* = 8.6 Hz, 1H), 8.31 (dd, *J* = 15.7, 7.8 Hz, 2H), 8.12 (d, *J* = 8.2 Hz, 1H), 7.77 (t, *J* = 7.7 Hz, 1H), 7.72 – 7.68 (m, 3H), 7.26 (s, 1H), 7.24 – 7.22 (m, 1H), 7.14 (d, *J* = 8.4 Hz, 1H), 3.83 (s, 6H) ppm.

^{13}C NMR Analysis: ^{13}C NMR (151 MHz, DMSO) δ 166.4, 165.7, 151.3, 148.0, 135.3, 134.7, 134.3, 133.8, 129.0, 128.3, 128.2, 127.7, 127.1, 125.5, 124.9, 124.6, 123.6, 118.5, 114.2, 112.2, 55.7, 55.6 ppm.

MS Analysis (low res): LRMS (ESI-) *m/z*: 453 (*M*-H, $C_{22}H_{17}N_2O_5S_2$, 100); (ESI+) *m/z*: 455 (*M*+H, $C_{22}H_{19}N_2O_5S_2$, 100)

MS Analysis (high res): Exact mass calculated for $C_{22}H_{17}N_2O_5S_2$ [*M*-H]$^-$, 453.0600. Found 453.0586.

HPLC method details: Column: Zorbax SB-C18 Rapid Resolution HT 2.1x50mm 1.8-Micron; Method: LCMS ISOCRATIC 60%B_3 MINS.M filename: KP9007; Peak retention time: 1.285 mins; Area (%): 100.

Procedure: To a 10mL microwave vial was added *N*-(4-oxo-4,5-dihydrothiazol-2-yl)naphthalene-1-sulfonamide (143 mg, 0.49 mmol), 3,4-dimethoxybenzaldehyde (94 mg, 0.54 mmol, 1.1 eq), ethanol (3 mL) and a catalytic amount of the benzoic acid/piperidine catalyst (approximately 5 drops). The suspension was heated by microwave irradiation (120 °C, 200 W) for 30 min. A precipitate formed upon cooling and the precipitate was collected to give the desired product as a bright yellow/orange solid (163 mg, 73%).

Other analyses, reference papers, previously obtained data, comments, etc:

PerkinElmer Spectrum Version 10.4.00
Thursday, 26 November 2020 11:56 AM

Analyst research
Date Thursday, 26 November 2020 11:56 AM

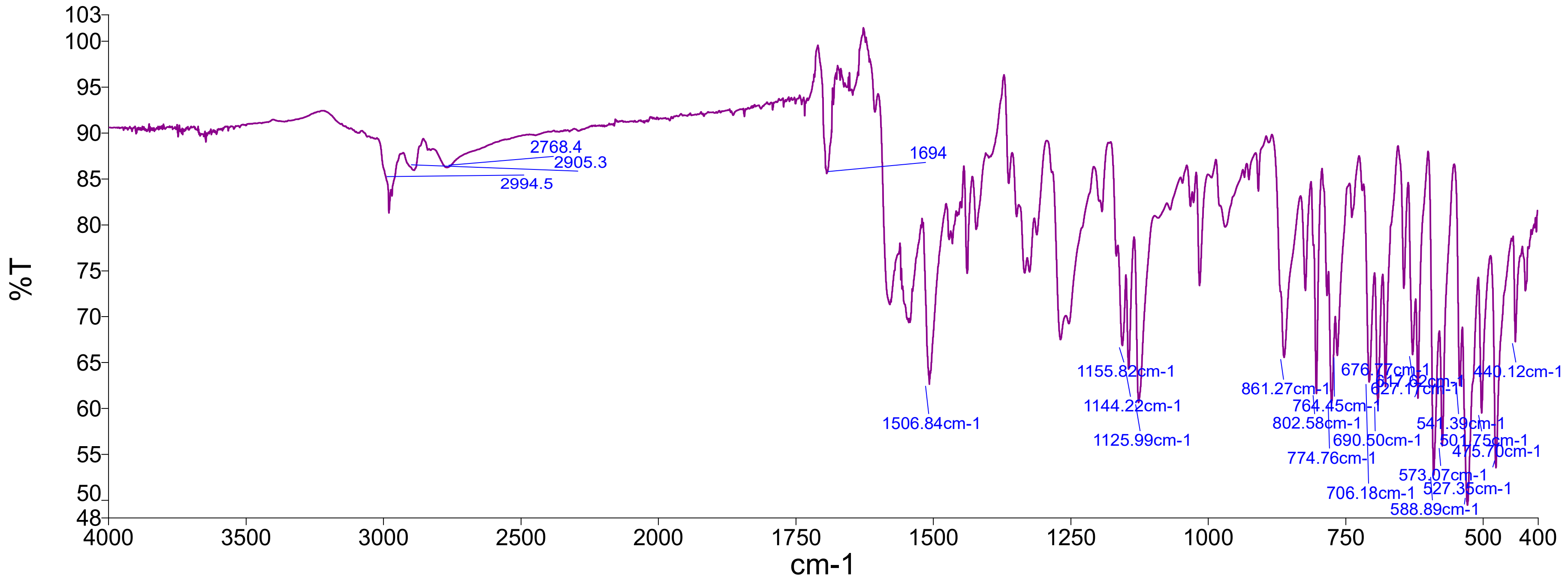

Sample Name	Description	Quality Checks
kp9007	Sample 181 By research Date Thursday, November 26 2020	The Quality Checks do not report any warnings for the sample.

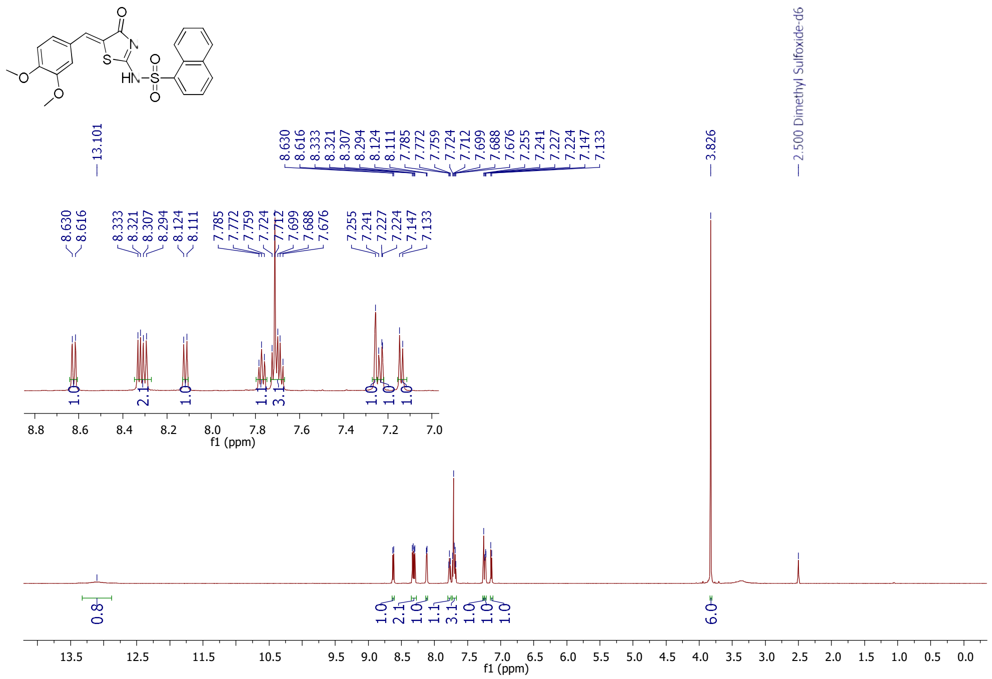

13.101
8.630 8.616 8.333 8.321 8.307 8.294 8.124 8.111 7.785 7.772 7.759 7.724 7.712 7.699 7.688 7.676 7.255 7.241 7.227 7.224 7.147 7.133
3.826
2.500 Dimethyl Sulfoxide-d6
1.0 2.1 1.0 1.1 3.1 1.0 1.0 1.0
0.8 1.0 2.1 1.0 1.1 3.1 1.0 1.0 1.0 6.0
f1 (ppm)
HN
S
O

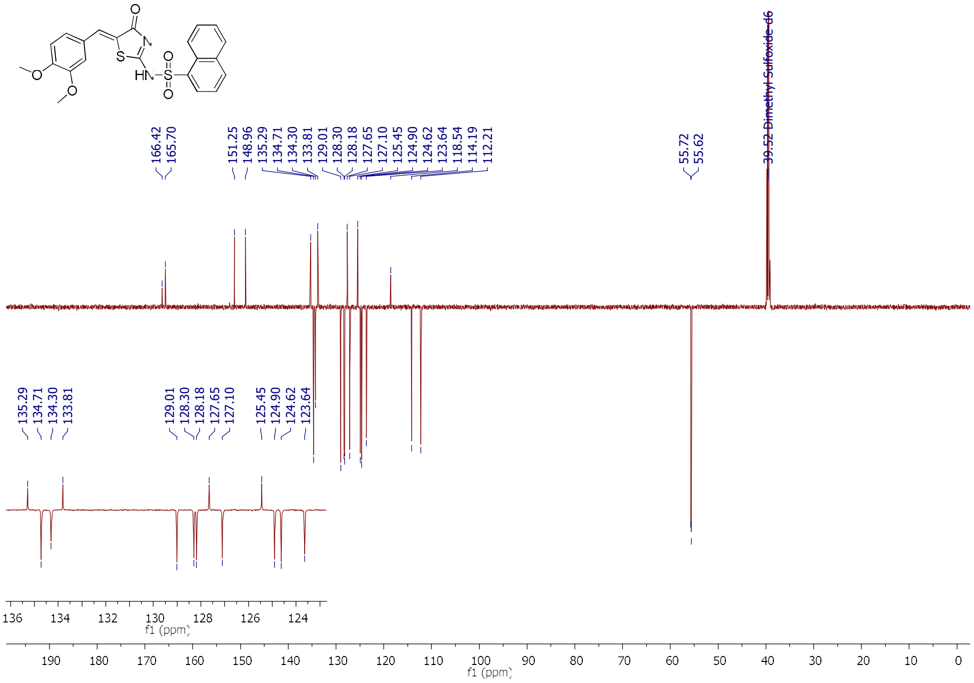

166.42 165.70
151.25 148.96 135.29 134.71 134.30 133.81 129.01 128.30 128.18 127.65 127.10 125.45 124.90 124.62 123.64 118.54 114.19 112.21
55.72 55.62
39.52 Dimethyl Sulfoxide-d6
135.29 134.71 134.30 133.81 129.01 128.30 128.18 127.65 127.10 125.45 124.90 124.62 123.64
f1 (ppm)
HN
S
O

LCMS Report

**Data file:**	D:\Chem32\1\Data\KP\KP_DS_NOV2 2020-11-02 12-46-10\002-16-KP9007.D		
**Sample name:**	KP9007		
**Description:**			
**Sample amount:**	0.000	**Sample type:**	Sample
**Instrument:**	LCMS	**Location:**	16
**Injection date:**	11/2/2020 12:52:21 PM	**Injection:**	1 of 1
**Acq. method:**	LCMS ISOCRATIC 60% B_3MINS.M	**Injection volume:**	2.000
**Analysis method:**	LCMS ISOCRATIC 60%B_3MINS.M	**Acq. operator:**	SYSTEM
**Last changed:**	5/19/2016 3:52:53 PM		

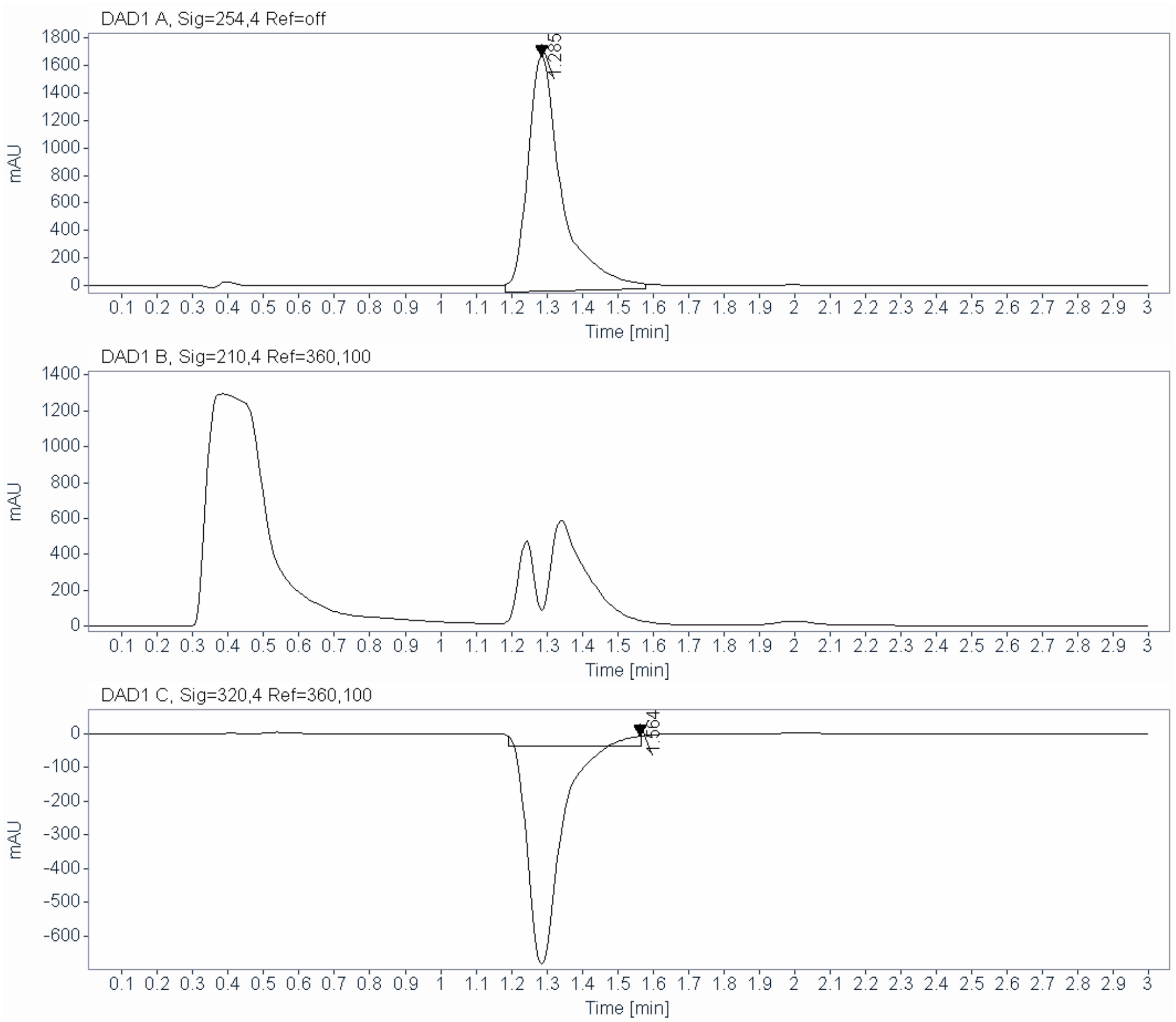

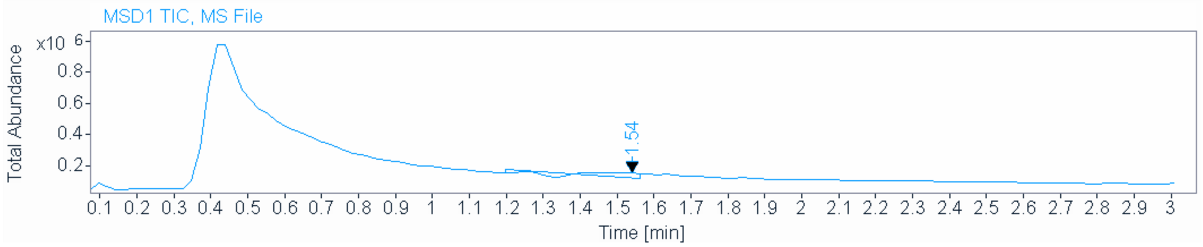

LCMS Report

Agilent Technologies

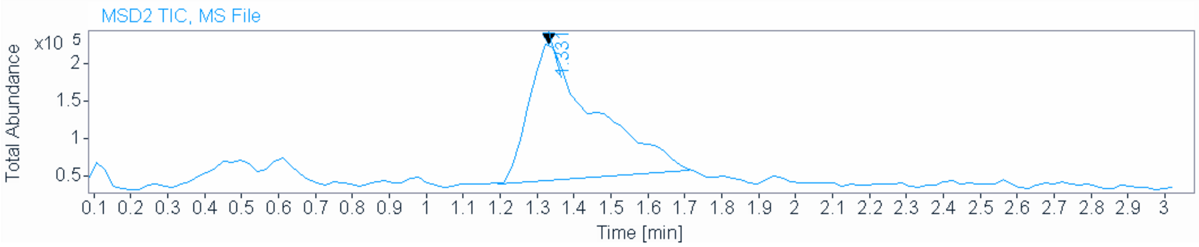

Signal: DAD1 A, Sig=254,4 Ref=off

RT [min]	Type	Width [min]	Area	Height	Area%	Name
1.285	MM	0.1121	11506.7139	1711.4491	100.0000	
		Sum	11506.7139			

Signal: DAD1 C, Sig=320,4 Ref=360,100

RT [min]	Type	Width [min]	Area	Height	Area%	Name
1.564	MM	0.0667	119.8202	29.9341	100.0000	
		Sum	119.8202			

Signal: MSD1 TIC, MS File

RT [min]	Type	Width [min]	Area	Height	Area%	Name
1.540	MM	0.1273	257114.9844	33658.3984	100.0000	
		Sum	257114.9844			

Signal: MSD2 TIC, MS File

RT [min]	Type	Width [min]	Area	Height	Area%	Name
1.331	MM	0.2051	2279799.000 0	185303.9063	100.0000	
		Sum	2279799.000			

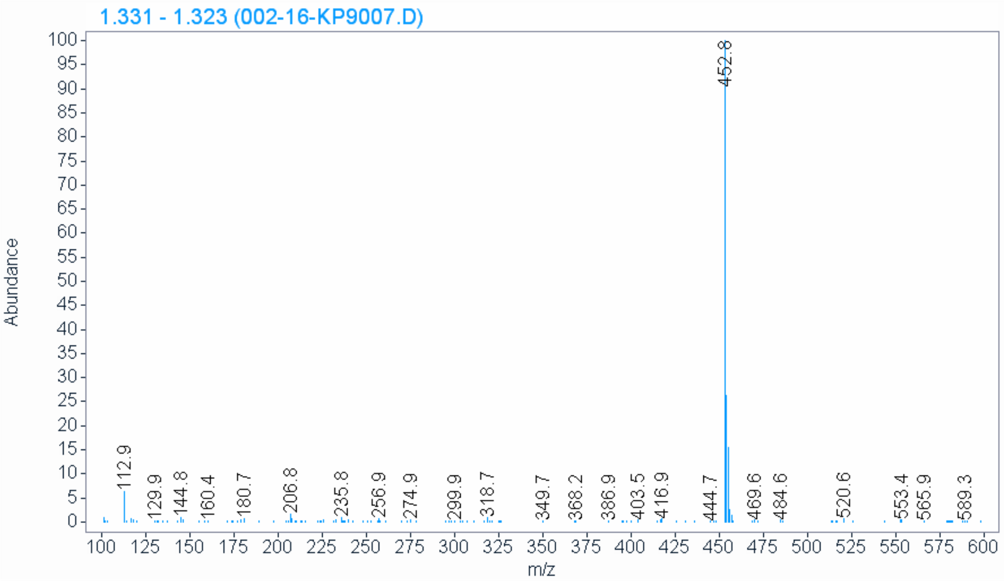
1.331 - 1.323 (002-16-KP9007.D)
Abundance
m/z
452.8
112.9
129.9
144.8
160.4
180.7
206.8
235.8
256.9
274.9
299.9
318.7
349.7
368.2
386.9
403.5
416.9
444.7
469.6
484.6
520.6
553.4
565.9
589.3

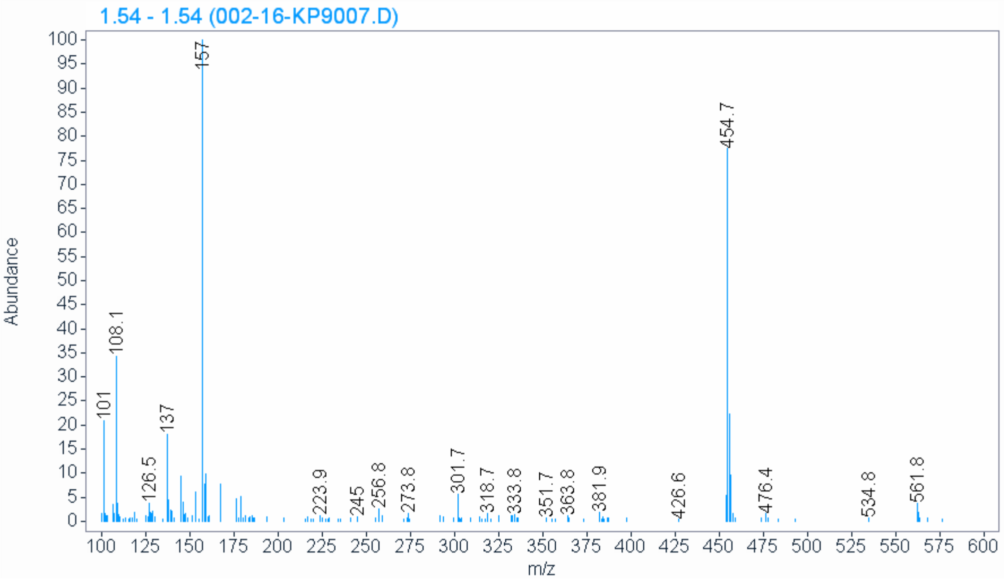
1.54 - 1.54 (002-16-KP9007.D)
Abundance
m/z
157
454.7
108.1
101
137
126.5
223.9
245
256.8
273.8
301.7
318.7
333.8
351.7
363.8
381.9
426.6
476.4
534.8
561.8

Compound Name: (Z)-*N*-(5-(3,5-dimethoxybenzylidene)-4-oxo-4,5-dihydrothiazol-2-yl)naphthalene-1-sulfonamide

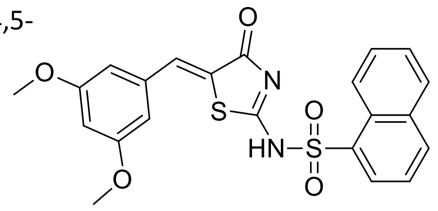

Chemical Formula: $C_{22}H_{18}N_2O_5S_2$
Exact Mass: 454.07
Molecular Weight: 454.52

Compound Code: 32 (KP7131)

Obtained Weight & Yield: 127 mg, 57%

Purity (by LCMS and ^{1}H NMR): > 95% by ^{1}H-NMR, > 99% by LCMS

Appearance: pale pink/ orange solid

Solubility: DMSO, slightly soluble in acetone and methanol

Melting Point: 222 – 225 °C

TLC Rf (and conditions): N/A

IR Analysis (including assignment): IR (neat): 3179 (NH), 2965 (C-H aromatic), 1723 (C=O), 1594 (aromatic C-C), 1299 (sulfonamide), 1124 (C-N) cm^{-1}

^{1}H NMR Analysis: ^{1}H NMR (400 MHz, DMSO) δ 8.61 (d, *J* = 8.6 Hz, 1H), 8.30 (d, *J* = 7.7 Hz, 2H), 8.12 (d, *J* = 8.0 Hz, 1H), 7.79 – 7.75 (m, 1H), 7.72 – 7.67 (m, 3H), 6.80 (d, *J* = 2.0 Hz, 2H), 6.69 (t, *J* = 2.0 Hz, 1H), 3.82 (s, 6H) ppm.
NH exchanging – not visible.
Ethanol solvent impurity 1.06 ppm (4.27%).

^{13}C NMR Analysis: ^{13}C NMR (101 MHz, DMSO) δ 166.3, 165.5, 160.9 (2C), 135.2, 134.8, 134.7, 133.8, 133.7, 129.0, 128.3, 128.2, 127.6, 127.1, 124.9, 124.6, 122.5, 108.2 (2C), 102.4, 55.5 (2C) ppm.

MS Analysis (low res): LRMS (ESI-) *m/z* (%): 453 (*M*-H, $C_{22}H_{17}N_2O_5S_2$, 100%); (ESI+) *m/z* (%): 455 (*M*+H, $C_{22}H_{19}N_2O_5S_2$, 100%).

MS Analysis (high res): Exact mass calculated for $C_{22}H_{17}N_2O_5S_2$ [*M*-H]$^-$, 453.0600. Found 453.0584.

HPLC method details: Column: Zorbax SB-C18 Rapid Resolution HT 2.1x50mm 1.8-Micron; Method: LCMS ISOCRATIC 60%B 0.4MLMIN-1.M filename: KP7131; Peak retention time: 1.677 mins; Area (%): 100

Procedure: To a 10mL microwave vial was added the *N*-(4-oxo-4,5-dihydrothiazol-2-yl)naphthalene-1-sulfonamide (148 mg, 0.49 mmol), 3,5-dimethoxybenzaldehyde (66 mg, 0.54 mmol, 1.1 eq), ethanol (3 mL) and a catalytic amount of the benzoic acid/piperidine catalyst (approximately 5 drops). The suspension was heated using microwave irradiation (200 W, 120 °C) for 30 min. The resulting precipitate was collected by vacuum filtration and washed with cold ethanol and cold ether to give the desired product (127 mg, 57%).

Other analyses, reference papers, previously obtained data, comments, etc:

PerkinElmer Spectrum Version 10.4.00
Thursday, 21 November 2019 11:44 AM

Analyst research
Date Thursday, 21 November 2019 11:44 AM

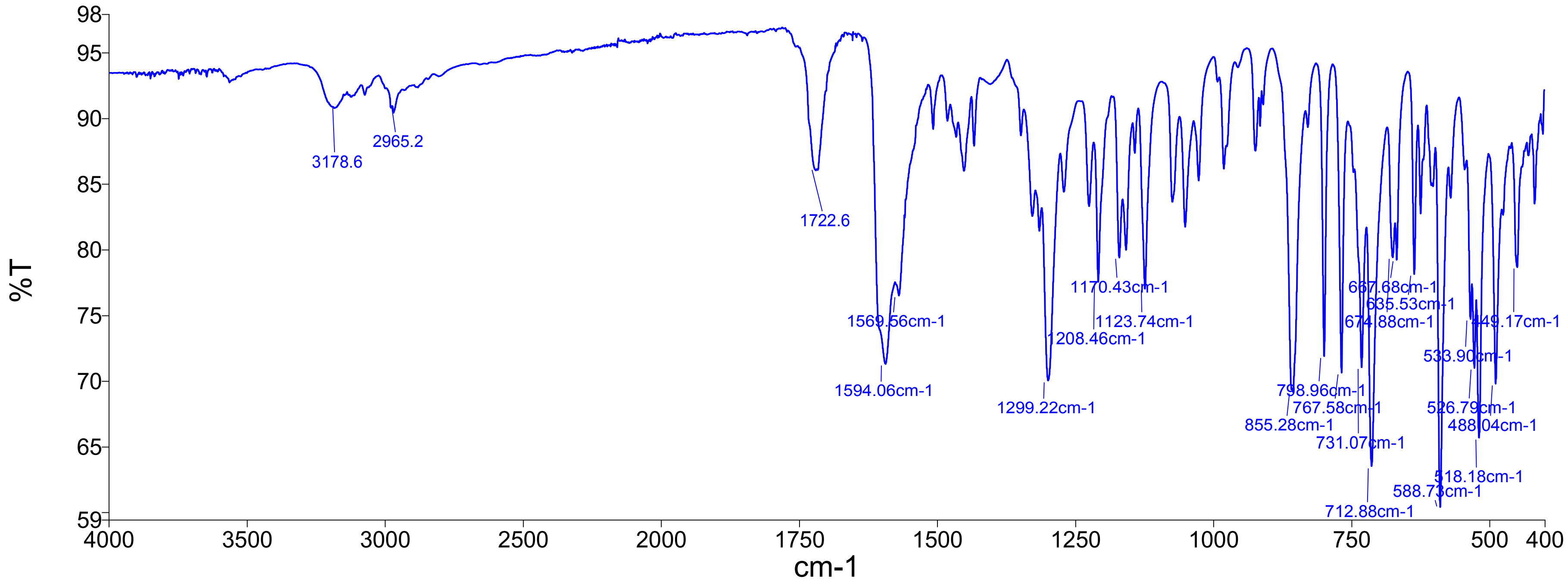

Sample Name	Description	Quality Checks
KP7131	Sample 255 By research Date Thursday, November 21 2019	The Quality Checks do not report any warnings for the sample.

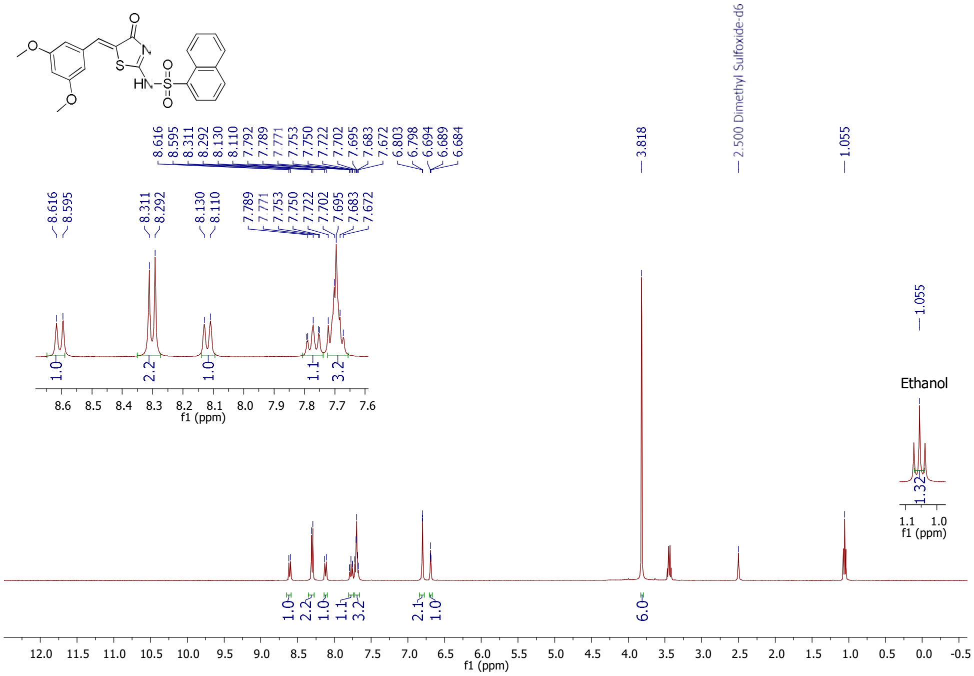

HN
S
O
N
O
O
S
O
O
8.616
8.595
8.311
8.292
8.130
8.110
7.792
7.789
7.771
7.753
7.750
7.722
7.702
7.695
7.683
7.672
6.803
6.798
6.694
6.689
6.684
3.818
2.500 Dimethyl Sulfoxide-d6
1.055
Ethanol
1.32
1.0
2.2
1.0
1.1
3.2
2.1
1.0
6.0
f1 (ppm)

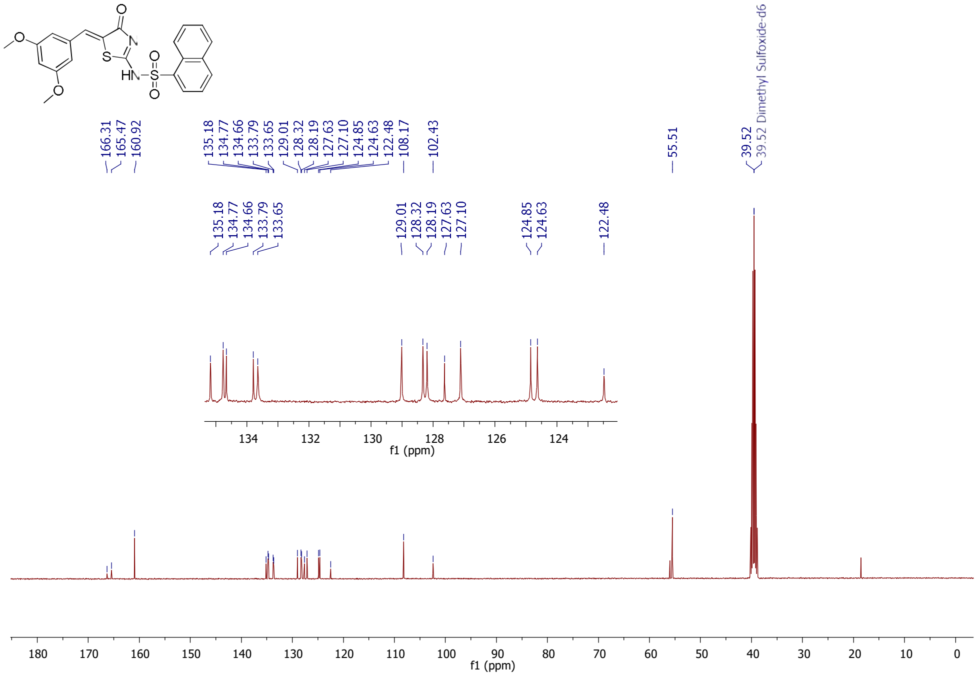

166.31
165.47
160.92
135.18
134.77
134.66
133.79
133.65
129.01
128.32
128.19
127.63
127.10
124.85
124.63
122.48
108.17
102.43
55.51
39.52
39.52 Dimethyl Sulfoxide-d6
f1 (ppm)

LCMS Report

Agilent Technologies

**Data file:**	D:\Chem32\1\Data\KP\KP7131-7133 2019-11-15 13-40-07\001-53-KP7131.D		
**Sample name:**	KP7131		
**Description:**			
**Sample amount:**	0.000	**Sample type:**	Sample
**Instrument:**	LCMS	**Location:**	53
**Injection date:**	11/15/2019 1:41:47 PM	**Injection:**	1 of 1
**Acq. method:**	LCMS ISOCRATIC 60% B 0.4MLMIN-1.M	**Injection volume:**	2.000
**Analysis method:**	LCMS ISOCRATIC 60%B 0.4MLMIN-1.M	**Acq. operator:**	SYSTEM
**Last changed:**	5/8/2019 8:55:04 AM		

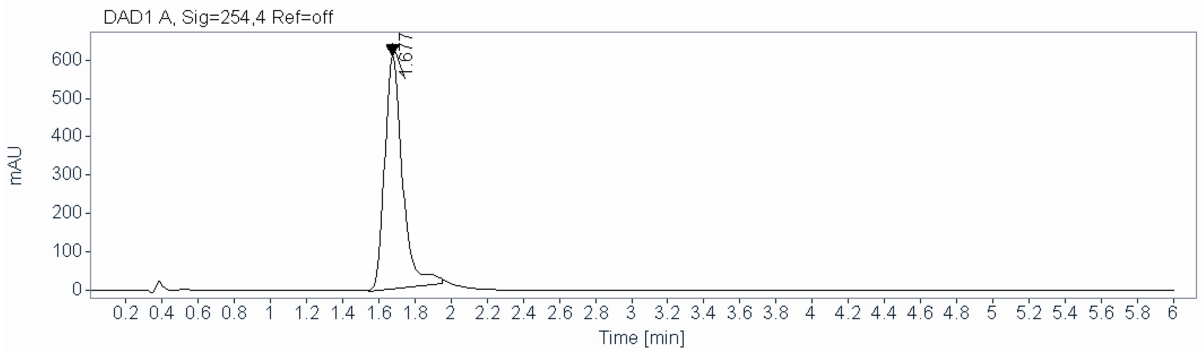

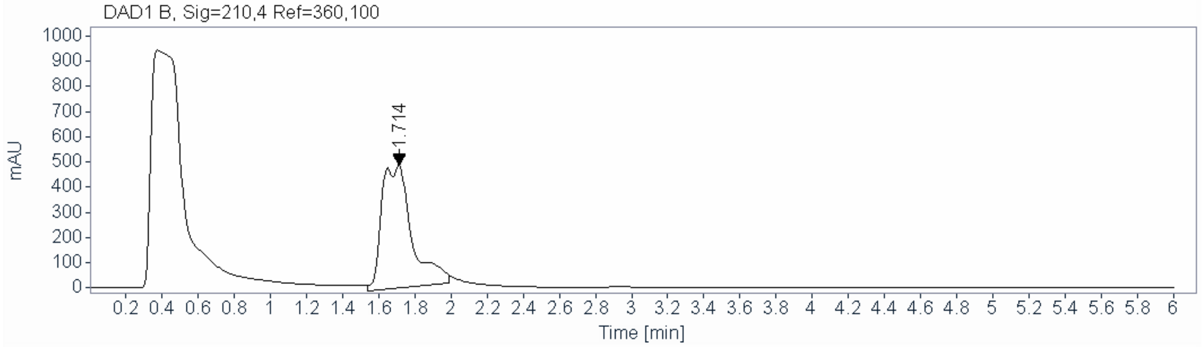

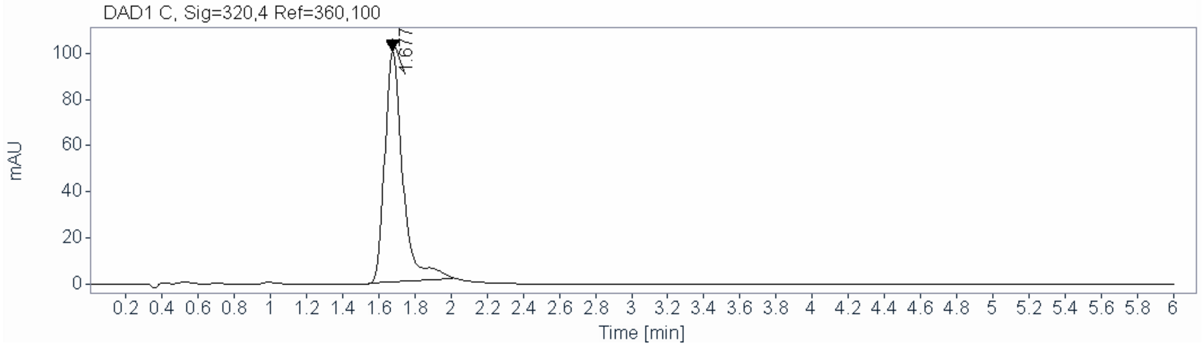

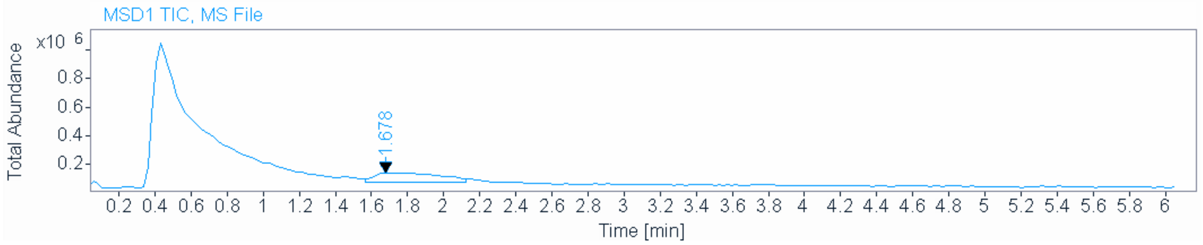

Agilent Technologies

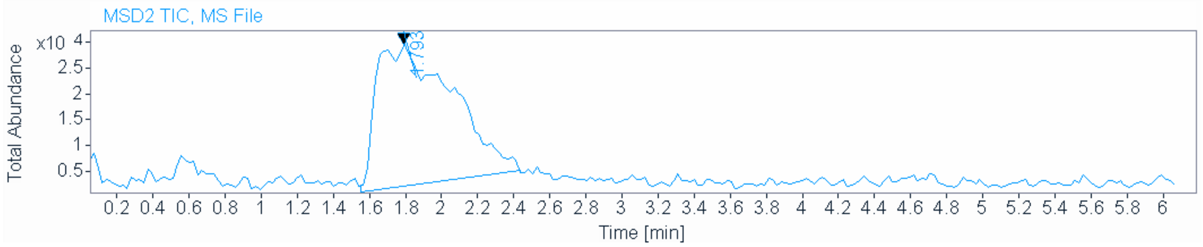

Signal: DAD1 A, Sig=254,4 Ref=off

RT [min]	Type	Width [min]	Area	Height	Area%	Name
1.677	MM	0.1152	4207.1694	608.8918	100.0000	
		Sum	4207.1694			

Signal: DAD1 B, Sig=210,4 Ref=360,100

RT [min]	Type	Width [min]	Area	Height	Area%	Name
1.714	MM	0.1921	5650.2622	490.2712	100.0000	
		Sum	5650.2622			

Signal: DAD1 C, Sig=320,4 Ref=360,100

RT [min]	Type	Width [min]	Area	Height	Area%	Name
1.677	MM	0.1153	692.9960	100.1353	100.0000	
		Sum	692.9960			

Signal: MSD1 TIC, MS File

RT [min]	Type	Width [min]	Area	Height	Area%	Name
1.678	MM	0.4126	1612387.250<br>0	65132.7656	100.0000	
		Sum	1612387.250			

Signal: MSD2 TIC, MS File

RT [min]	Type	Width [min]	Area	Height	Area%	Name
1.793	MM	0.4831	799450.5000	27583.2637	100.0000	
		Sum	799450.5000			

Agilent Technologies

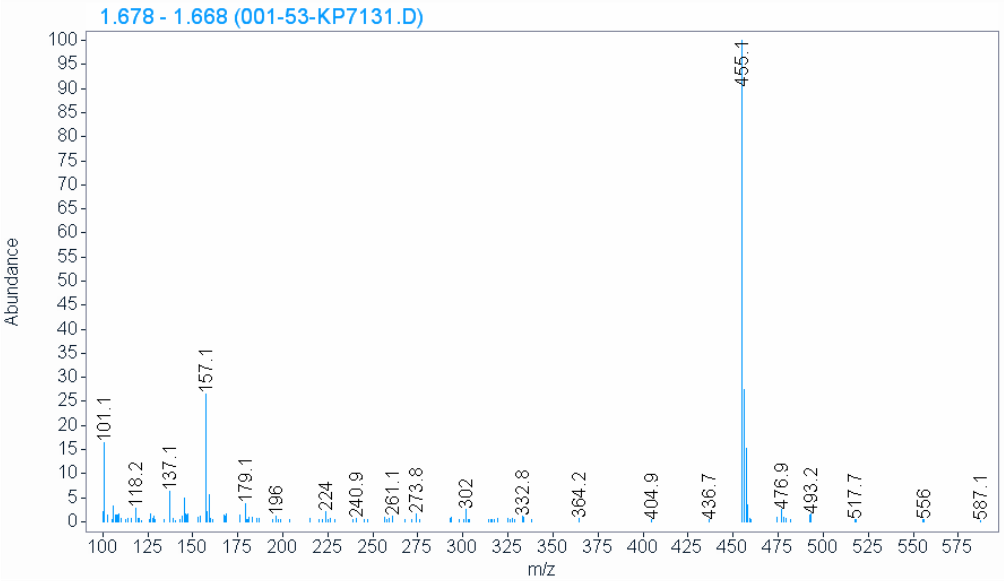

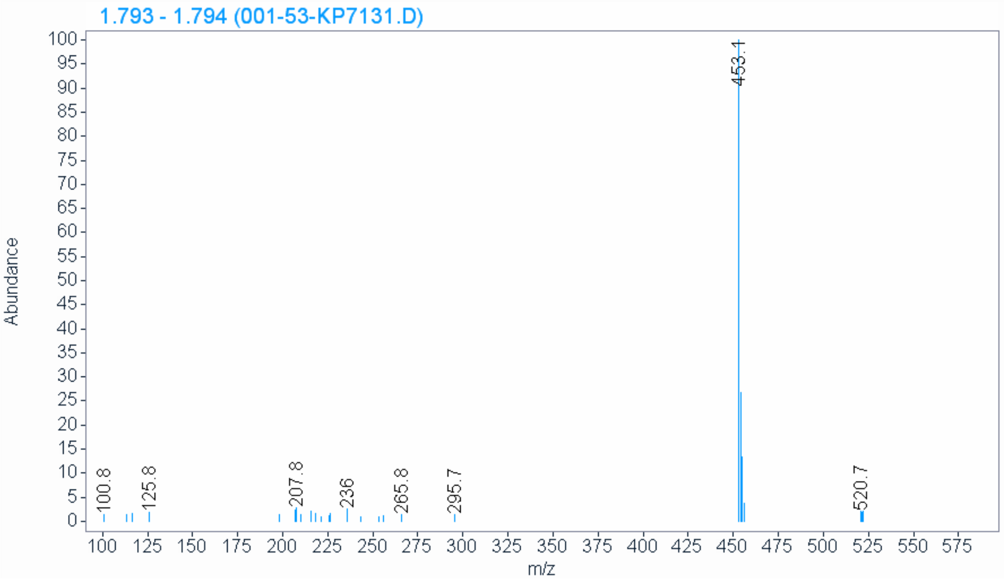

Compound Name: (*Z*)-*N*-(4-oxo-5-(3,4,5-trimethoxybenzylidene)-4,5-dihydrothiazol-2-yl)naphthalene-1-sulfonamide

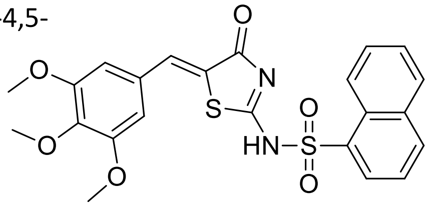

Chemical Formula: $C_{23}H_{20}N_2O_6S_2$
Exact Mass: 484.08
Molecular Weight: 484.54

Compound Code: 33 (KP9008)

Obtained Weight & Yield: 137 mg (58%)

Purity (by LCMS and ^{1}H NMR): > 97% by ^{1}H-NMR and LCMS

Appearance: bright yellow/orange solid

Solubility: DMSO, slightly soluble in acetone and methanol.

Melting Point: > 231 °C (dec.)

TLC Rf (and conditions): N/A

IR Analysis (including assignment): IR (neat): ν_{max} = 3060 (N-H), 2983, 2893 (C-H aromatic), 2786 (C-H alkyl), 1706 (C=O), 1559 (C-C aromatic), 1232 (sulfonamide), 1125 (C-N) cm^{-1}

^{1}H NMR Analysis: ^{1}H NMR (600 MHz, DMSO) δ 8.61 (d, *J* = 8.6 Hz, 1H), 8.30 (t, *J* = 7.9 Hz, 2H), 8.11 (d, *J* = 8.1 Hz, 1H), 7.77 (t, *J* = 7.6 Hz, 1H), 7.71 – 7.67 (m, 3H), 6.97 (s, 2H), 3.86 (s, 6H), 3.76 (s, 3H) ppm.
NH exchanging – not visible.
Ethanol at 1.05 ppm (1.47%), also some aldehyde SM at 9.88 ppm (0.40%)

^{13}C NMR Analysis: ^{13}C NMR (151 MHz, DMSO) δ 165.8 (br, 2C), 153.3 (2C), 139.9, 135.2 (br), 134.7, 134.0 (br), 133.8, 129.0, 128.4, 128.34, 128.31, 127.7, 127.1, 124.9, 124.6, 120.9 (br), 107.9 (2C), 60.3, 56.1 (2C) ppm.
2C determined by 2D NMR.

MS Analysis (low res): LRMS (ESI-) *m/z*: 483 (*M*-H, $C_{23}H_{19}N_2O_6S_2$, 100); (ESI+) *m/z*: 485 (*M*+H, $C_{23}H_{21}N_2O_6S_2$, 100)

MS Analysis (high res): Exact mass calculated for $C_{23}H_{19}N_2O_6S_2$ [*M*-H]$^-$, 483.0700. Found 483.0690.

HPLC method details: Column: Zorbax SB-C18 Rapid Resolution HT 2.1x50mm 1.8-Micron; Method: LCMS ISOCRATIC 60%B_3 MINS.M filename: KP9008; Peak retention time: 1.477 mins; Area (%): 100.

Procedure: To a 10mL microwave vial was added *N*-(4-oxo-4,5-dihydrothiazol-2-yl)naphthalene-1-sulfonamide (144 mg, 0.49 mmol), 3,4,5-trimethoxybenzaldehyde (114 mg, 0.54 mmol, 1.1 eq), ethanol (3 mL) and a catalytic amount of the benzoic acid/piperidine catalyst (approximately 5 drops). The suspension was heated by microwave irradiation (120 °C, 200 W) for 30 min. A precipitate formed upon cooling and sonication and was collected to give the desired product as a bright yellow/orange solid (137 mg, 58%).

Other analyses, reference papers, previously obtained data, comments, etc:

PerkinElmer Spectrum Version 10.4.00
Thursday, 26 November 2020 11:56 AM

Analyst research

Date Thursday, 26 November 2020 11:56 AM

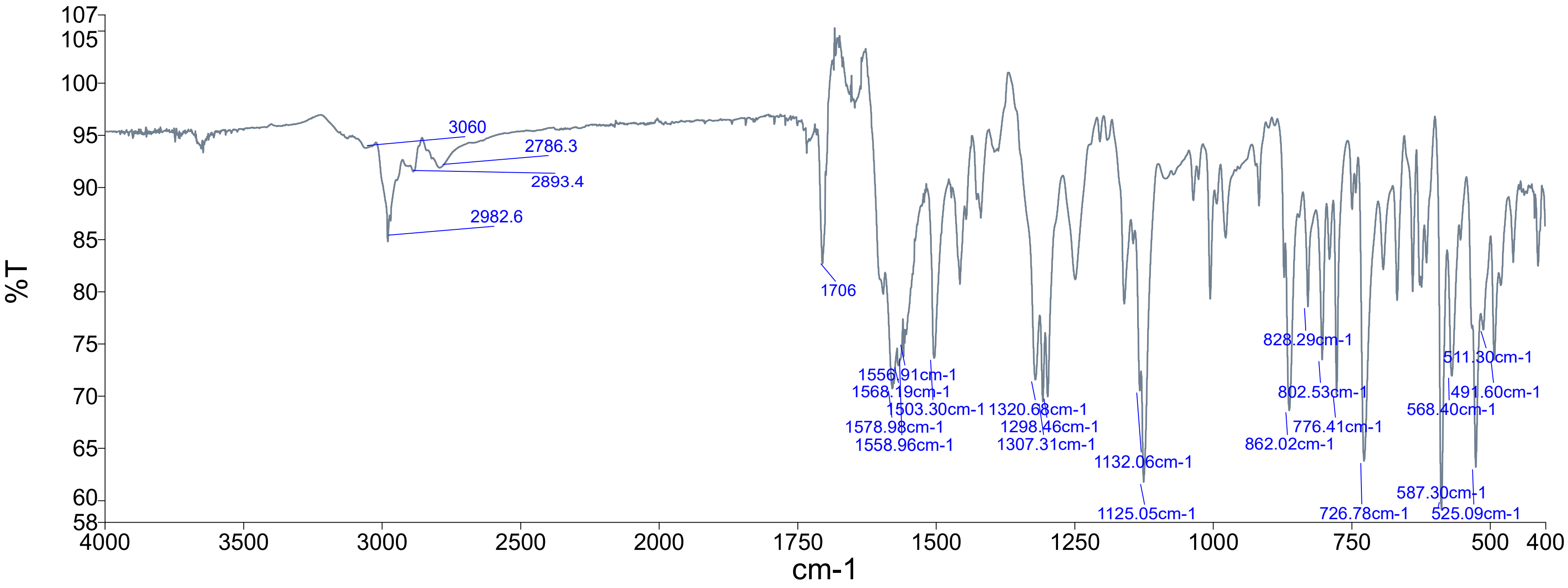

Sample Name	Description	Quality Checks
kp9008	Sample 182 By research Date Thursday, November 26 2020	The Quality Checks do not report any warnings for the sample.

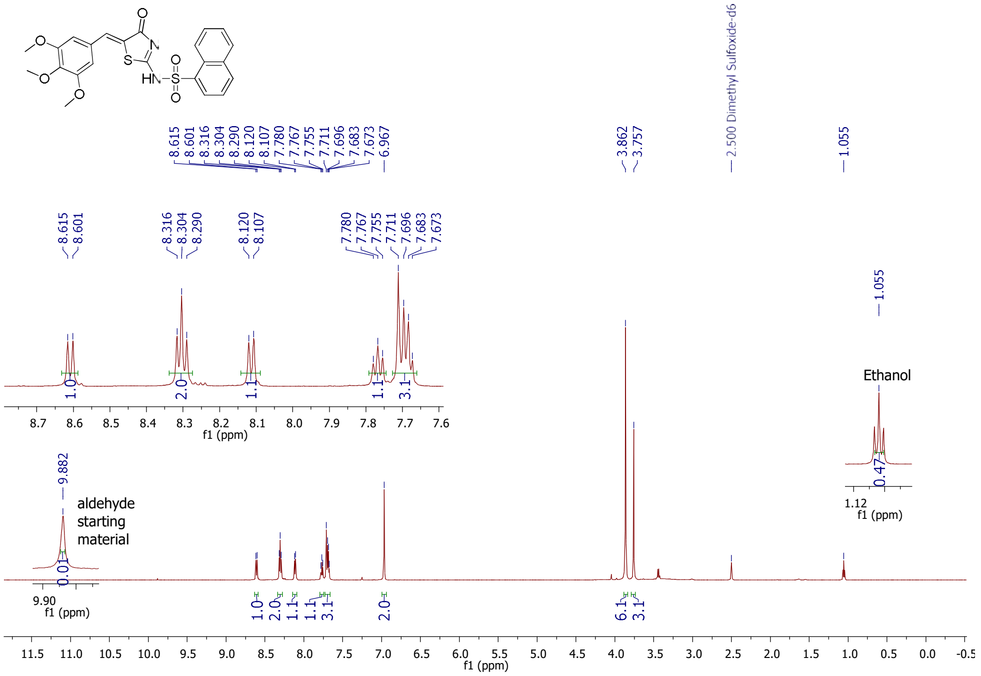

O
N
S
HN
S
O
O
8.615
8.601
8.316
8.304
8.290
8.120
8.107
7.780
7.767
7.755
7.711
7.696
7.683
7.673
6.967
3.862
3.757
2.500 Dimethyl Sulfoxide-d6
1.055
1.0
2.0
1.1
1.1
3.1
Ethanol
0.47
1.12
f1 (ppm)
9.882
aldehyde
starting
material
0.01
9.90
f1 (ppm)
2.0
6.1
3.1
f1 (ppm)

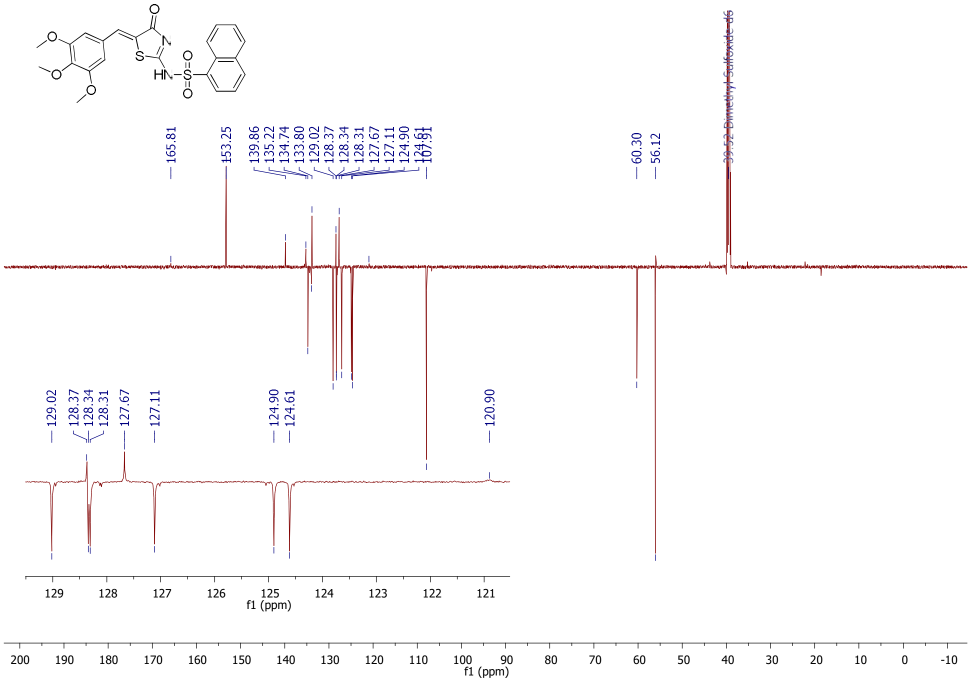

O
N
S
HN
S
O
O
165.81
153.25
139.86
135.22
134.74
133.80
129.02
128.37
128.34
128.31
127.67
127.11
124.90
124.61
107.91
60.30
56.12
Dimethyl Sulfoxide-d6
129.02
128.37
128.34
128.31
127.67
127.11
124.90
124.61
120.90
f1 (ppm)
f1 (ppm)

LCMS Report

Agilent Technologies

**Data file:**	D:\Chem32\1\Data\KP\KP_DS_NOV2 2020-11-02 12-46-10\003-15-KP9008.D		
**Sample name:**	KP9008		
**Description:**			
**Sample amount:**	0.000	**Sample type:**	Sample
**Instrument:**	LCMS	**Location:**	15
**Injection date:**	11/2/2020 12:56:59 PM	**Injection:**	1 of 1
**Acq. method:**	LCMS ISOCRATIC 60% B_3MINS.M	**Injection volume:**	2.000
**Analysis method:**	LCMS ISOCRATIC 60%B_3MINS.M	**Acq. operator:**	SYSTEM
**Last changed:**	5/19/2016 3:52:53 PM		

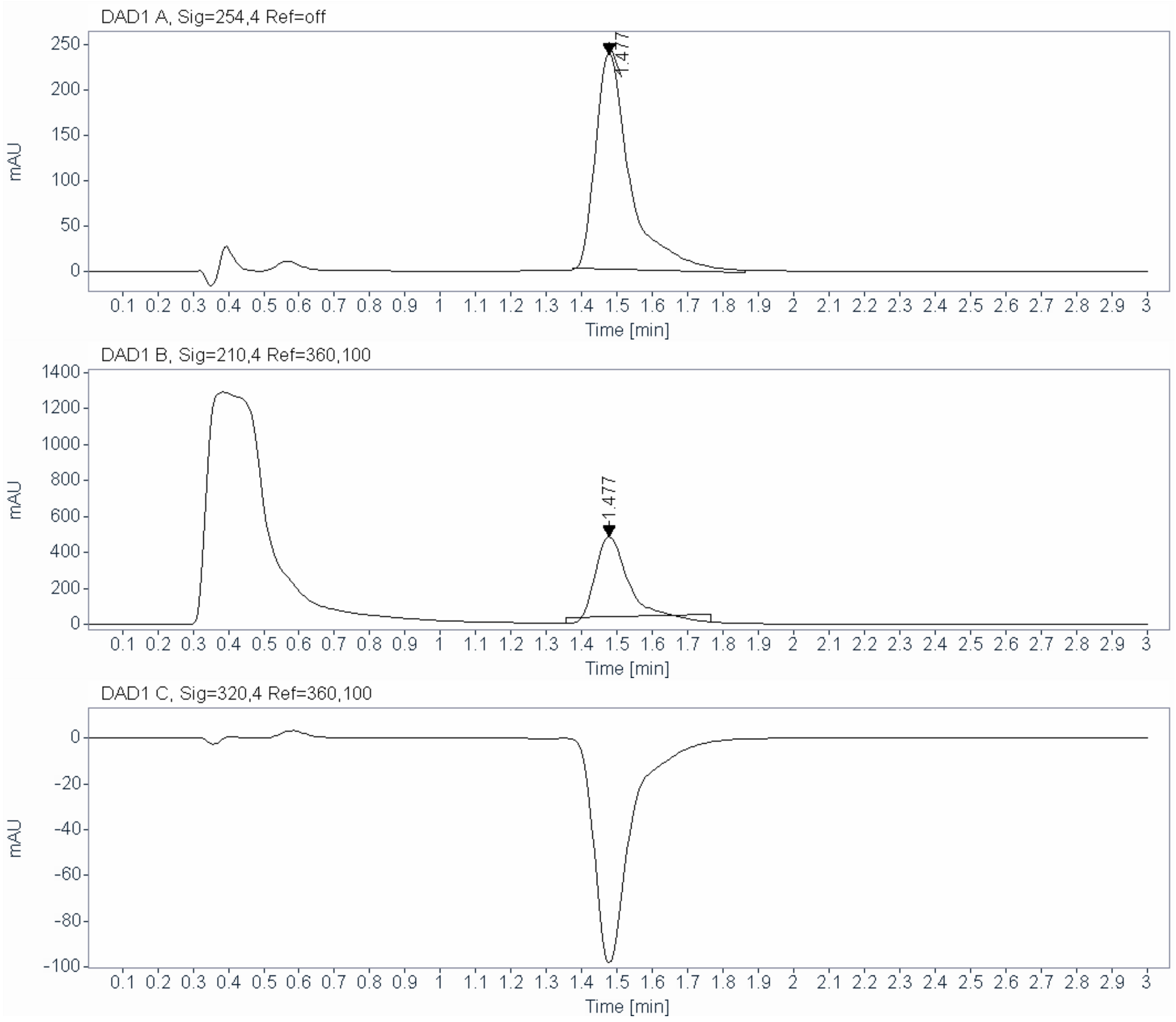

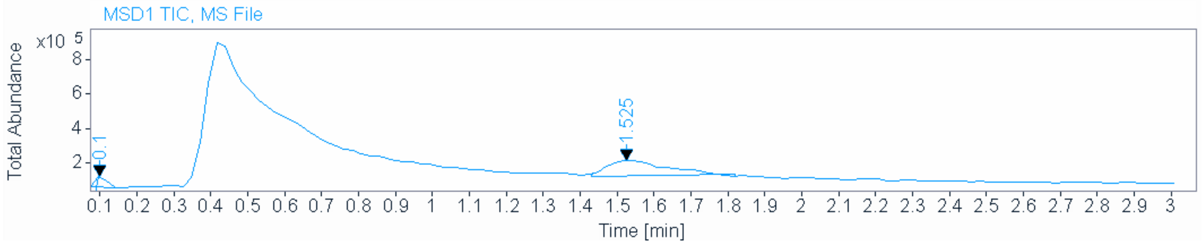

Agilent Technologies

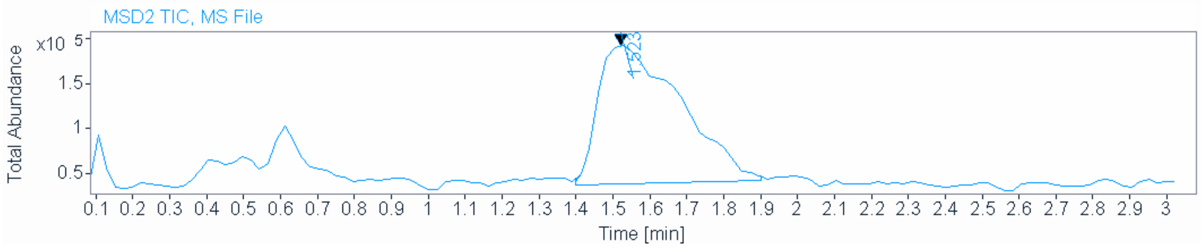

Signal: DAD1 A, Sig=254,4 Ref=off

RT [min]	Type	Width [min]	Area	Height	Area%	Name
1.477	MM	0.1144	1632.5392	237.9191	100.0000	
		Sum	1632.5392			

Signal: DAD1 B, Sig=210,4 Ref=360,100

RT [min]	Type	Width [min]	Area	Height	Area%	Name
1.477	MM	0.0994	2651.1733	444.6078	100.0000	
		Sum	2651.1733			

Signal: MSD1 TIC, MS File

RT [min]	Type	Width [min]	Area	Height	Area%	Name
0.100	BB	0.0357	129616.0859	60467.4336	11.3221	
1.525	MM	0.1777	1015189.062<br>5	95215.8984	88.6779	
		Sum	1144805.148			

Signal: MSD2 TIC, MS File

RT [min]	Type	Width [min]	Area	Height	Area%	Name
1.523	MM	0.2561	2357642.750<br>0	153454.2969	100.0000	
		Sum	2357642.750			

Agilent Technologies

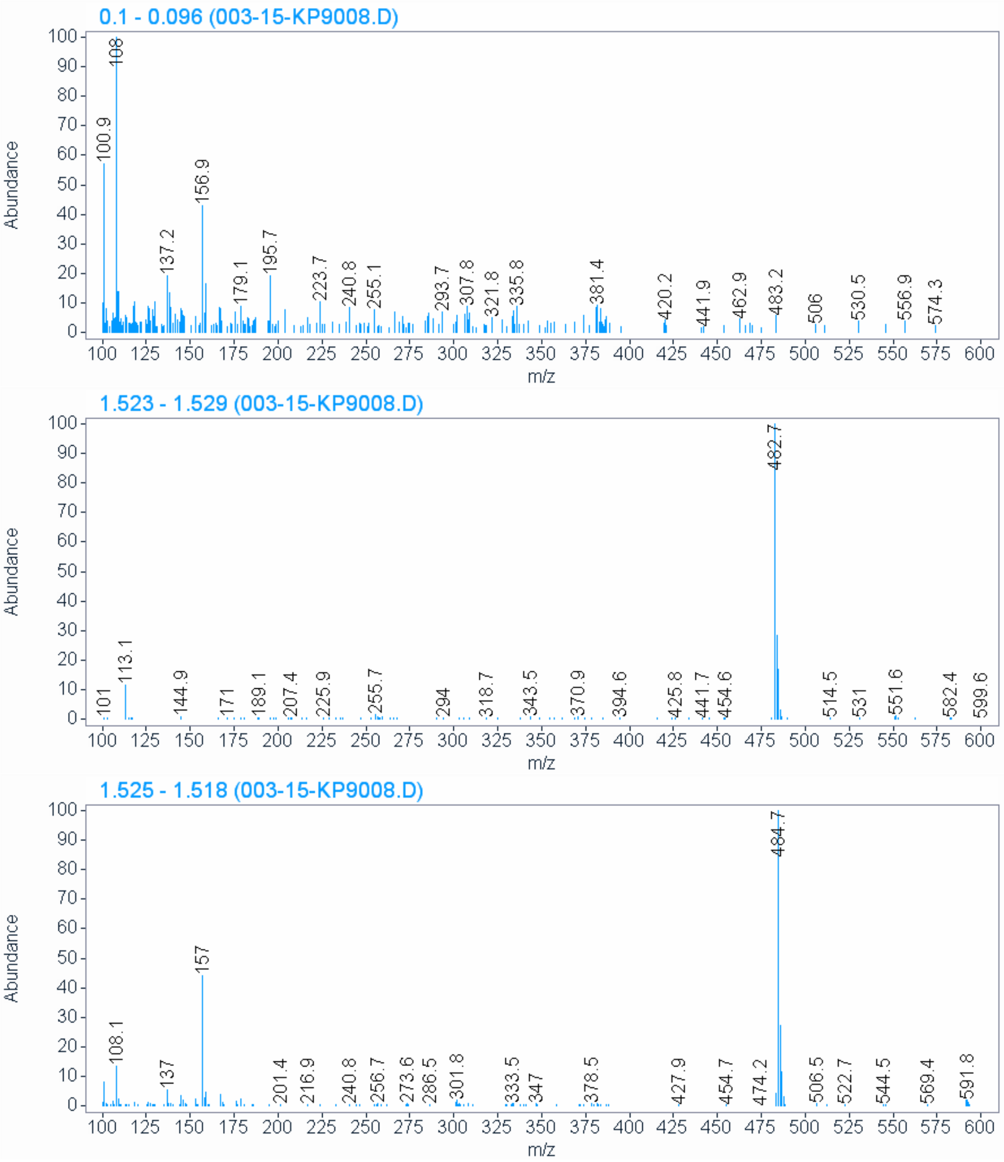

Compound Name: (*Z*)-*N*-(5-(2,3-dichlorobenzylidene)-4-oxo-4,5-dihydrothiazol-2-yl)naphthalene-1-sulfonamide

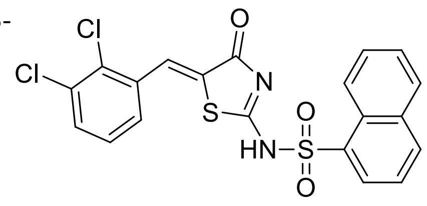

Chemical Formula: $C_{20}H_{12}Cl_2N_2O_3S_2$
Exact Mass: 461.97
Molecular Weight: 463.36

Compound Code: 34 (KP9189)

Obtained Weight & Yield: 178 mg (78%)

Purity (by LCMS and ^{1}H NMR): > 98% by ^{1}H-NMR and LCMS

Appearance: off white solid

Solubility: DMSO, slightly soluble in acetone and methanol.

Melting Point: 270 °C (dec.)

TLC Rf (and conditions): N/A

IR Analysis (including assignment): IR (neat): ν_{max} = 3125 (N-H), 3055, 3025 (C-H aromatic), 2962, 2787 (C-H alkyl), 1716 (C=O), 1565 (C=C aromatic), 1346, 1156 (sulfonamide), 1129 (C-N), 767, 714 (C-Cl) cm^{-1}

^{1}H NMR Analysis: ^{1}H NMR (400 MHz, DMSO) δ 8.59 (d, *J* = 8.5 Hz, 1H), 8.29 (dd, *J* = 13.1, 7.8 Hz, 2H), 8.12 (d, *J* = 8.1 Hz, 1H), 7.85 (s, 1H), 7.82 – 7.75 (m, 2H), 7.71 – 7.61 (m, 4H) ppm.
NH exchanging – not visible.
Ethanol impurity at 1.06 ppm (0.38%) and aldehyde starting material at 10.33 ppm (0.66%).

^{13}C NMR Analysis: ^{13}C NMR (101 MHz, DMSO) δ 166.2, 165.3, 135.1, 134.8, 133.8, 133.5, 133.1, 132.2, 132.1, 129.2, 129.0, 128.4 (2C), 128.1, 127.8, 127.6, 127.2, 127.1, 124.8, 124.6 ppm
2C determined by 2D NMRs.

MS Analysis (low res): LRMS (ESI-): 461 (*M*-H, $C_{20}H_{11}{}^{35}Cl_2N_2O_3S_2$, 100), 463 (*M*-H, $C_{20}H_{11}{}^{35}Cl^{37}ClN_2O_3S_2$, 65), 465 (*M*-H, $C_{20}H_{11}{}^{37}Cl_2N_2O_3S_2$, 15)

HPLC method details: Column: Zorbax SB-C18 Rapid Resolution HT 2.1x50mm 1.8-Micron; Method: LCMS ISOCRATIC 80%B_3 MINS.M filename: KP9189; Peak retention time: 0.910 mins; Area (%): 100.

Procedure: To a 10 mL microwave vial was added *N*-(4-oxo-4,5-dihydrothiazol-2-yl)naphthalene-1-sulfonamide (149 mg, 0.49 mmol), 2,3-dichlorobenzaldehyde (137 mg, 0.735 mmol, 1.1 eq), ethanol (3 mL) and a catalytic amount of the benzoic acid/piperidine catalyst (approximately 5 drops). The suspension was heated by microwave irradiation (120 °C, 200 W) for 1.5 h. A precipitate formed upon cooling. The solid was collected by vacuum filtration to give the desired product as a yellow/orange solid (178 mg, 78%).

Other analyses, reference papers, previously obtained data, comments, etc:

Analyst	A1
Date	Wednesday, 16 March 2022 2:58 PM

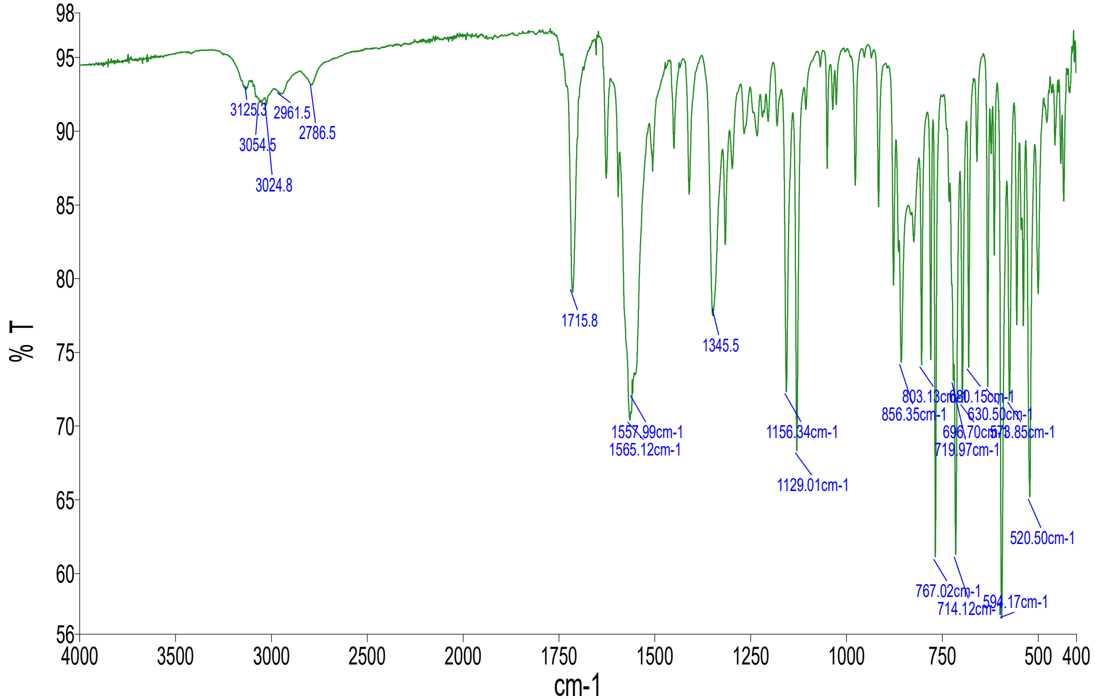

Sample Name	Description	Quality Checks
KP9189	Sample 032 By A1 Date Wednesday, March 16 2022	The Quality Checks do not report any warnings for the sample.

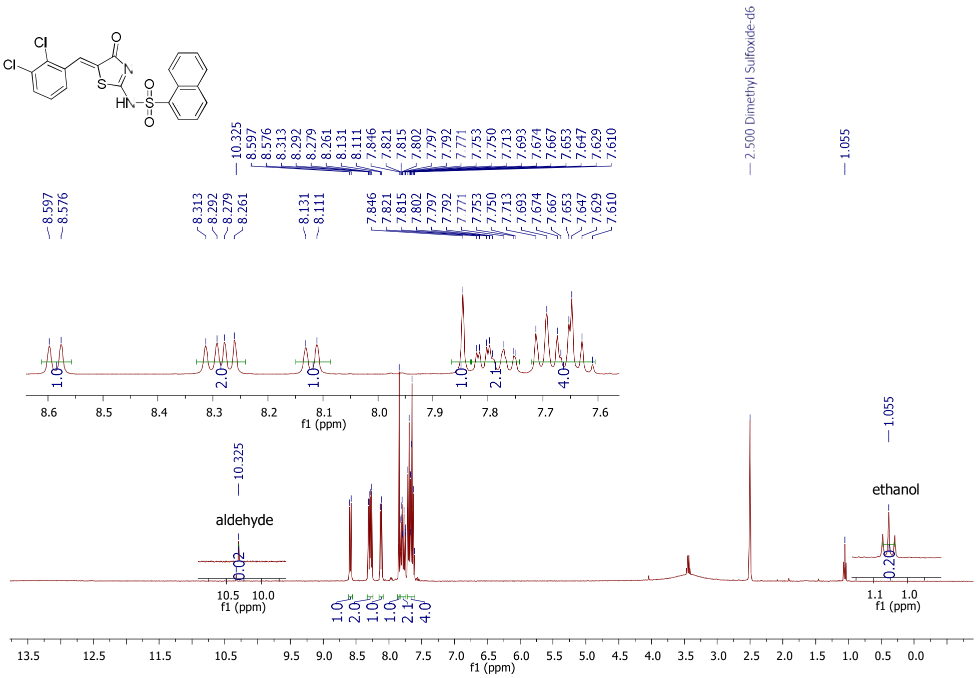

Cl
Cl
O
N
S
HN
S
O
O
10.325
8.597
8.576
8.313
8.292
8.279
8.261
8.131
8.111
7.846
7.821
7.815
7.802
7.797
7.792
7.771
7.753
7.750
7.713
7.693
7.674
7.667
7.653
7.647
7.629
7.610
2.500 Dimethyl Sulfoxide-d6
1.055
1.0
2.0
1.0
1.0
2.1
4.0
f1 (ppm)
aldehyde
0.02
ethanol
0.20
f1 (ppm)

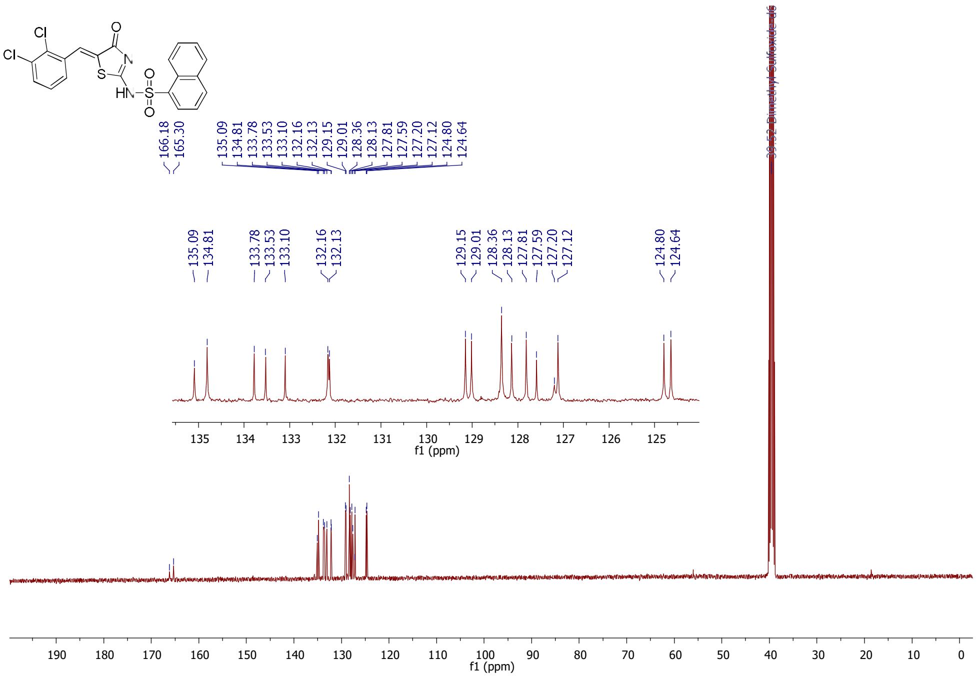

Cl
Cl
O
N
S
HN
S
O
O
166.18
165.30
135.09
134.81
133.78
133.53
133.10
132.16
132.13
129.15
129.01
128.36
128.13
127.81
127.59
127.20
127.12
124.80
124.64
f1 (ppm)
f1 (ppm)

LCMS Report

Agilent Technologies

Data file: D:\Chem32\1\Data\KP\kp_DEC4TH_80 2020-12-04 11-14-36\002-20-KP9189.D
Sample name: KP9189
Description:
Sample amount: 0.000 **Sample type:** Sample

Instrument: LCMS **Location:** 20
Injection date: 12/4/2020 11:21:04 AM **Injection:** 1 of 1
Acq. method: LCMS ISOCRATIC 80% B_3 MINS.M **Injection volume:** 2.000
Analysis method: LCMS ISOCRATIC 80%B_3 MINS.M **Acq. operator:** SYSTEM
Last changed: 10/8/2020 2:52:31 PM

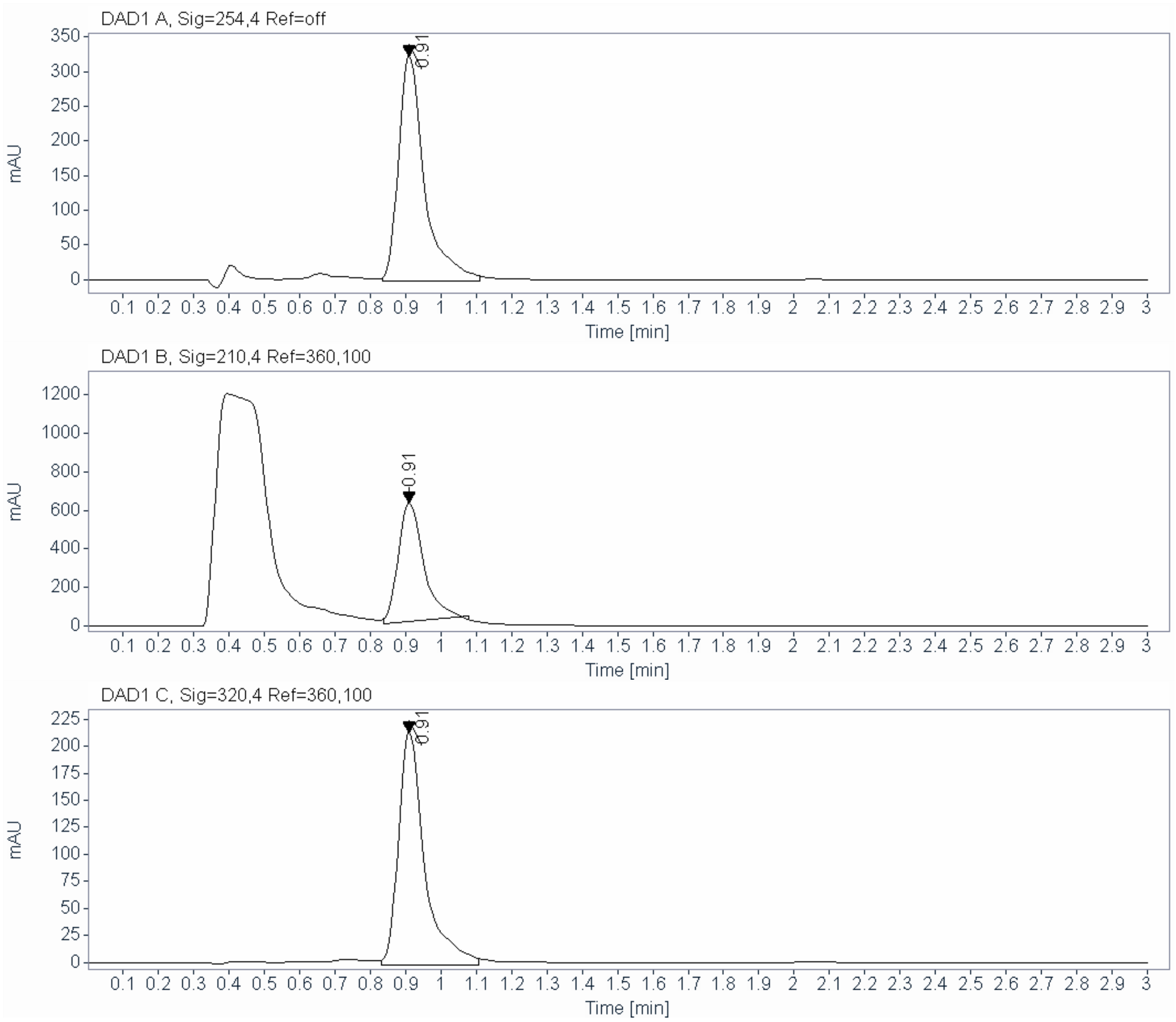

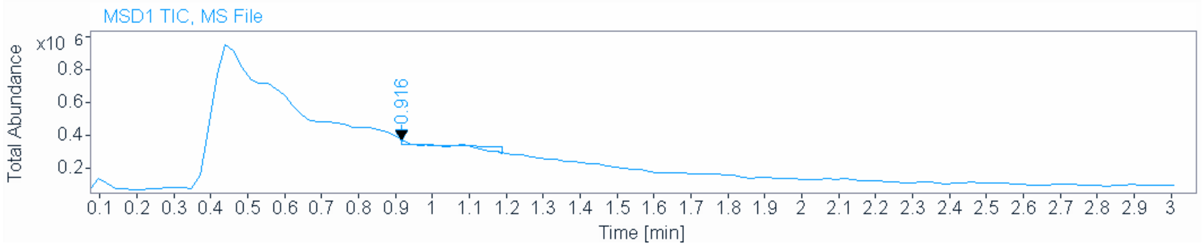

Agilent Technologies

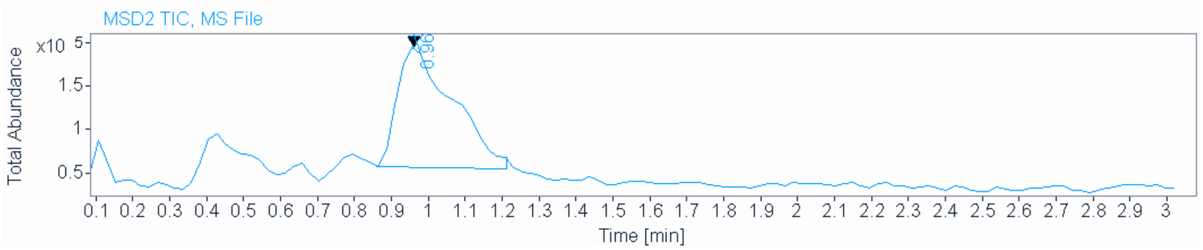

Signal: DAD1 A, Sig=254,4 Ref=off

RT [min]	Type	Width [min]	Area	Height	Area%	Name
0.910	MM	0.0846	1661.5396	327.4124	100.0000	
		Sum	1661.5396			

Signal: DAD1 B, Sig=210,4 Ref=360,100

RT [min]	Type	Width [min]	Area	Height	Area%	Name
0.910	MM	0.0822	3044.4578	617.4374	100.0000	
		Sum	3044.4578			

Signal: DAD1 C, Sig=320,4 Ref=360,100

RT [min]	Type	Width [min]	Area	Height	Area%	Name
0.910	MM	0.0852	1107.4795	216.6191	100.0000	
		Sum	1107.4795			

Signal: MSD1 TIC, MS File

RT [min]	Type	Width [min]	Area	Height	Area%	Name
0.916	MM	0.0307	28825.3281	27419.4648	100.0000	
		Sum	28825.3281			

Signal: MSD2 TIC, MS File

RT [min]	Type	Width [min]	Area	Height	Area%	Name
0.960	MM	0.1732	1445416.1250	139097.7188	100.0000	
		Sum	1445416.125			

Agilent Technologies

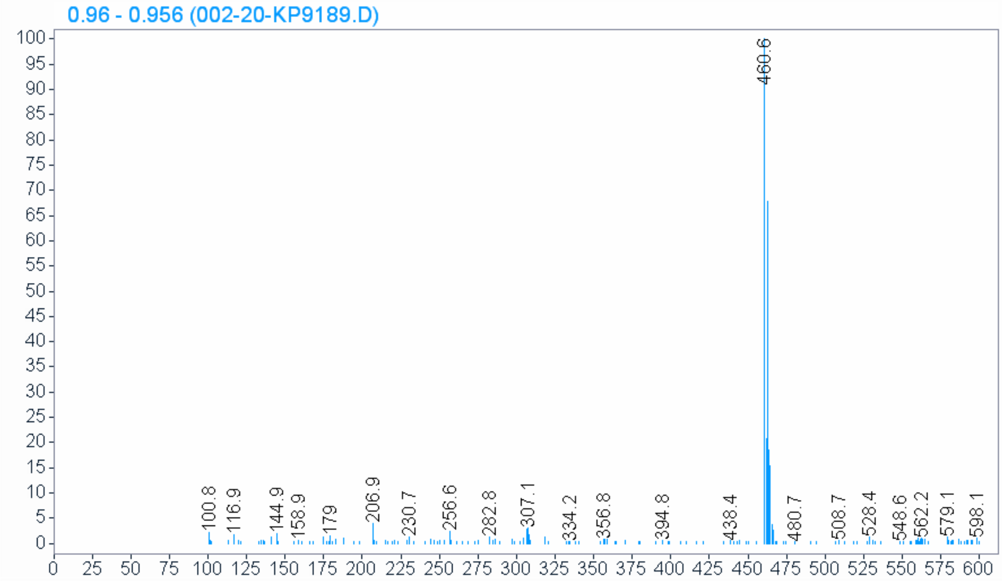

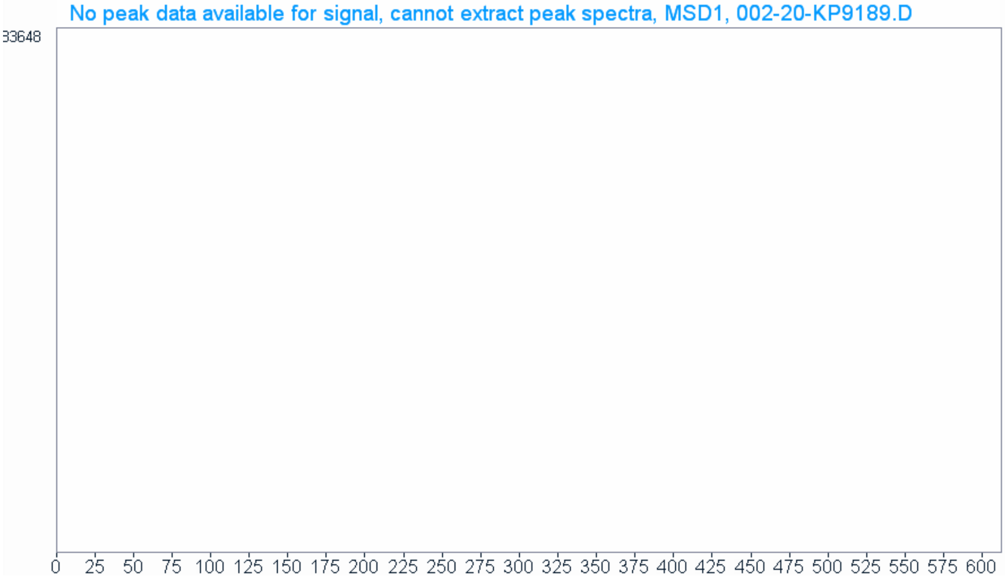

Compound Name: (*Z*)-*N*-(5-(2,4-dichlorobenzylidene)-4-oxo-4,5-dihydrothiazol-2-yl)naphthalene-1-sulfonamide

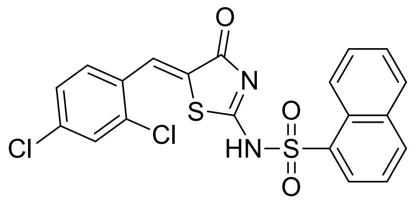

Chemical Formula: $C_{20}H_{12}Cl_2N_2O_3S_2$
Exact Mass: 461.97
Molecular Weight: 463.36

Compound Code: 35 (KP9034)

Obtained Weight & Yield: 110 mg (49%)

Purity (by LCMS and ^{1}H NMR): > 99% by ^{1}H-NMR and LCMS

Appearance: pale orange solid

Solubility: DMSO, slightly soluble in acetone and methanol.

Melting Point: > 202 °C (dec.)

TLC Rf (and conditions): N/A

IR Analysis (including assignment): IR (neat): ν_{max} = 3054, 2983 (C-H aromatic), 2893, 2810 (C-H alkyl), 1703 (C=O), 1569 (C-C aromatic), 1331 (sulfonamide), 1130 (C-N), 764 (C-Cl) cm^{-1}

^{1}H NMR Analysis: ^{1}H NMR (600 MHz, DMSO) δ 8.59 (d, *J* = 8.6 Hz, 1H), 8.29 (dd, *J* = 14.3, 7.8 Hz, 2H), 8.12 (d, *J* = 8.1 Hz, 1H), 7.85 (d, *J* = 1.6 Hz, 1H), 7.79 – 7.76 (m, 2H), 7.71 – 7.67 (m, 4H) ppm.
NH exchanging – not visible. Ethanol at 1.05 ppm (0.86%).

^{13}C NMR Analysis: ^{13}C NMR (151 MHz, DMSO) δ 166.2, 165.1, 135.8, 135.5, 135.1, 134.8, 133.8, 130.3, 130.0, 129.9, 129.0, 128.6, 128.4, 128.1, 127.6, 127.2, 127.1, 126.3, 124.8, 124.6 ppm.

MS Analysis (low res): LRMS (ESI-) *m/z*: 461 (*M*-H, $C_{20}H_{11}{}^{35}Cl_2N_2O_3S_2$, 100), 463 (*M*-H, $C_{20}H_{11}{}^{35/37}Cl_2N_2O_3S_2$, 75), 465 (*M*-H, $C_{20}H_{11}{}^{37}Cl_2N_2O_3S_2$, 17)

MS Analysis (high res): Exact mass calculated for $C_{20}H_{11}Cl_2N_2O_3S_2$ [*M*-H]$^-$, 460.9600. Found 460.9596.

HPLC method details: Column: Zorbax SB-C18 Rapid Resolution HT 2.1x50mm 1.8-Micron; Method: LCMS ISOCRATIC 80%B_3 MINS.M filename: KP9034; Peak retention time: 1.176 mins; Area (%): 100.

Procedure: To a 10mL microwave vial was added *N*-(4-oxo-4,5-dihydrothiazol-2-yl)naphthalene-1-sulfonamide (150 mg, 0.49 mmol), 2,4-dichlorobenzaldehyde (129 mg, 0.734 mmol, 1.1 eq), ethanol (3 mL) and a catalytic amount of the benzoic acid/piperidine catalyst (approximately 5 drops). The suspension was heated by microwave irradiation (120 °C, 200 W) for 1 h. A precipitate formed upon cooling. The solid was collected by vacuum filtration to give the desired product as a pale orange solid (110 mg, 49%).

Other analyses, reference papers, previously obtained data, comments, etc:

PerkinElmer Spectrum Version 10.4.00
Thursday, 26 November 2020 11:54 AM

Analyst research

Date Thursday, 26 November 2020 11:54 AM

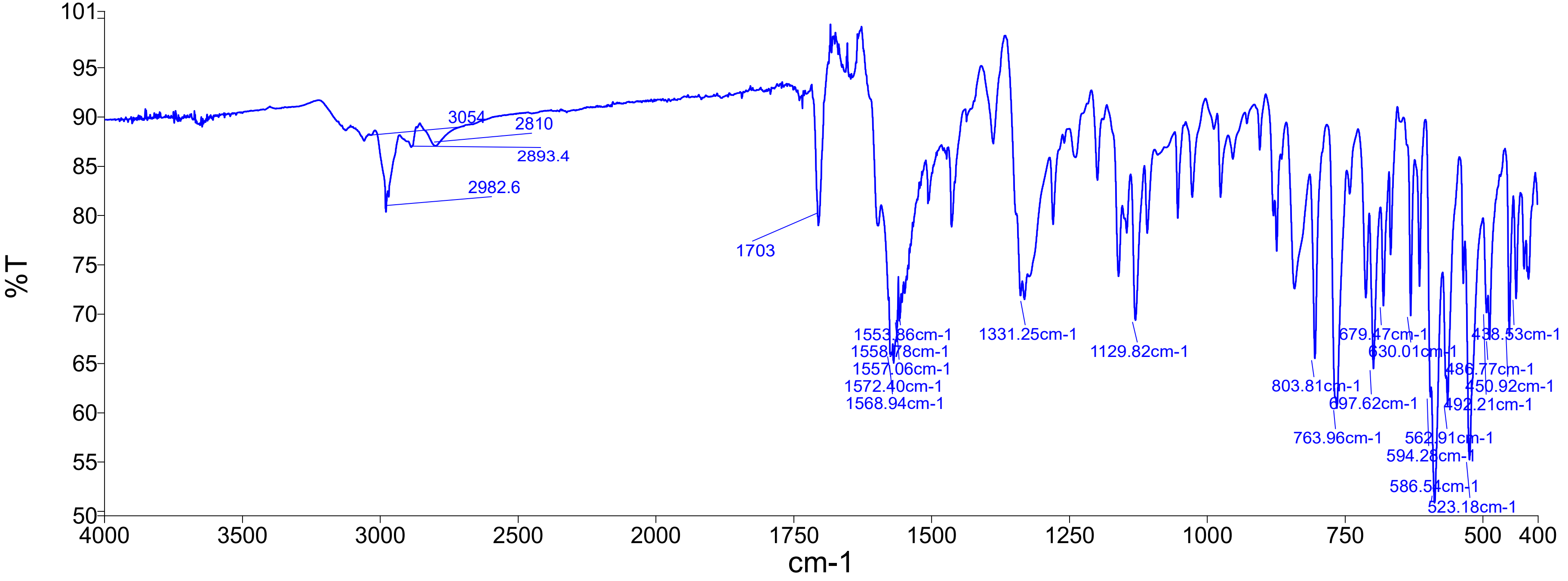

Sample Name	Description	Quality Checks
kp9034	Sample 177 By research Date Thursday, November 26 2020	The Quality Checks do not report any warnings for the sample.

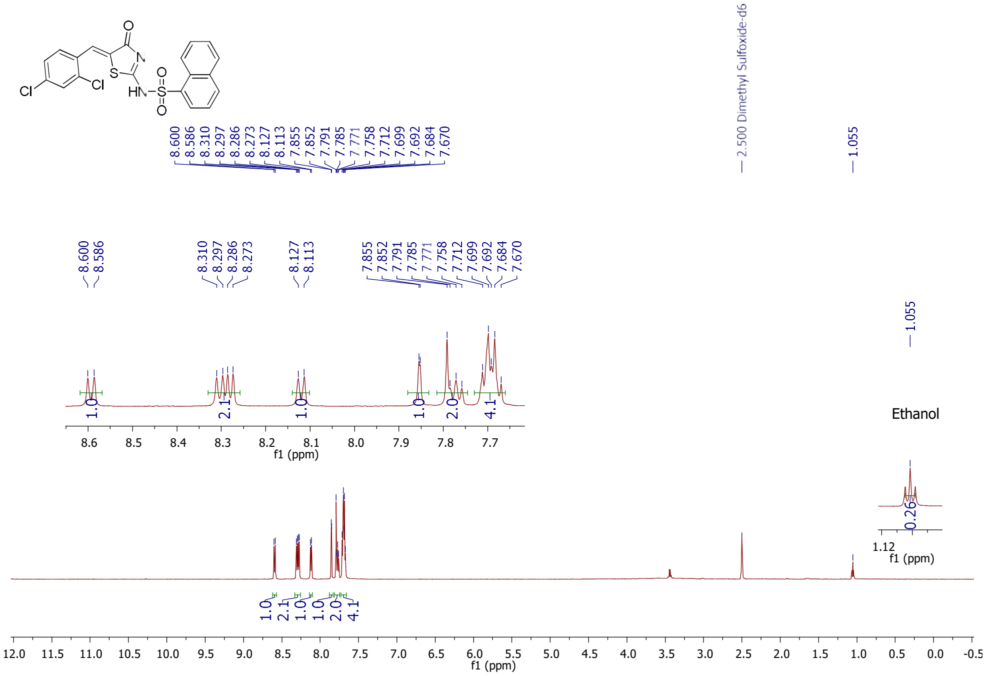
Cl
Cl
S
HN
O
2.500 Dimethyl Sulfoxide-d6
1.055
Ethanol
f1 (ppm)

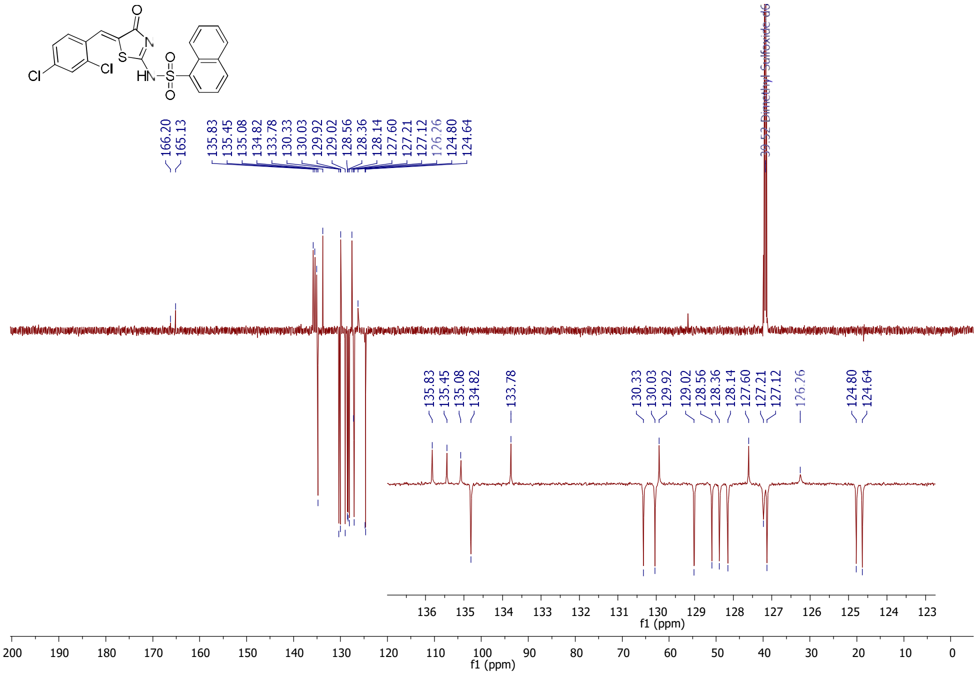
Cl
Cl
S
HN
O
166.20
165.13
135.83
135.45
135.08
134.82
133.78
130.33
130.03
129.92
129.02
128.56
128.36
128.14
127.60
127.21
127.12
126.26
124.80
124.64
f1 (ppm)

LCMS Report

Agilent Technologies

**Data file:**	D:\Chem32\1\Data\KP\KP_DS_NOV4 2020-11-02 13-34-37\005-17-KP9034.D		
**Sample name:**	KP9034		
**Description:**			
**Sample amount:**	0.000	**Sample type:**	Sample
**Instrument:**	LCMS	**Location:**	17
**Injection date:**	11/2/2020 1:54:40 PM	**Injection:**	1 of 1
**Acq. method:**	LCMS ISOCRATIC 80% B_3 MINS.M	**Injection volume:**	2.000
**Analysis method:**	LCMS ISOCRATIC 80%B_3 MINS.M	**Acq. operator:**	SYSTEM
**Last changed:**	10/8/2020 2:52:31 PM		

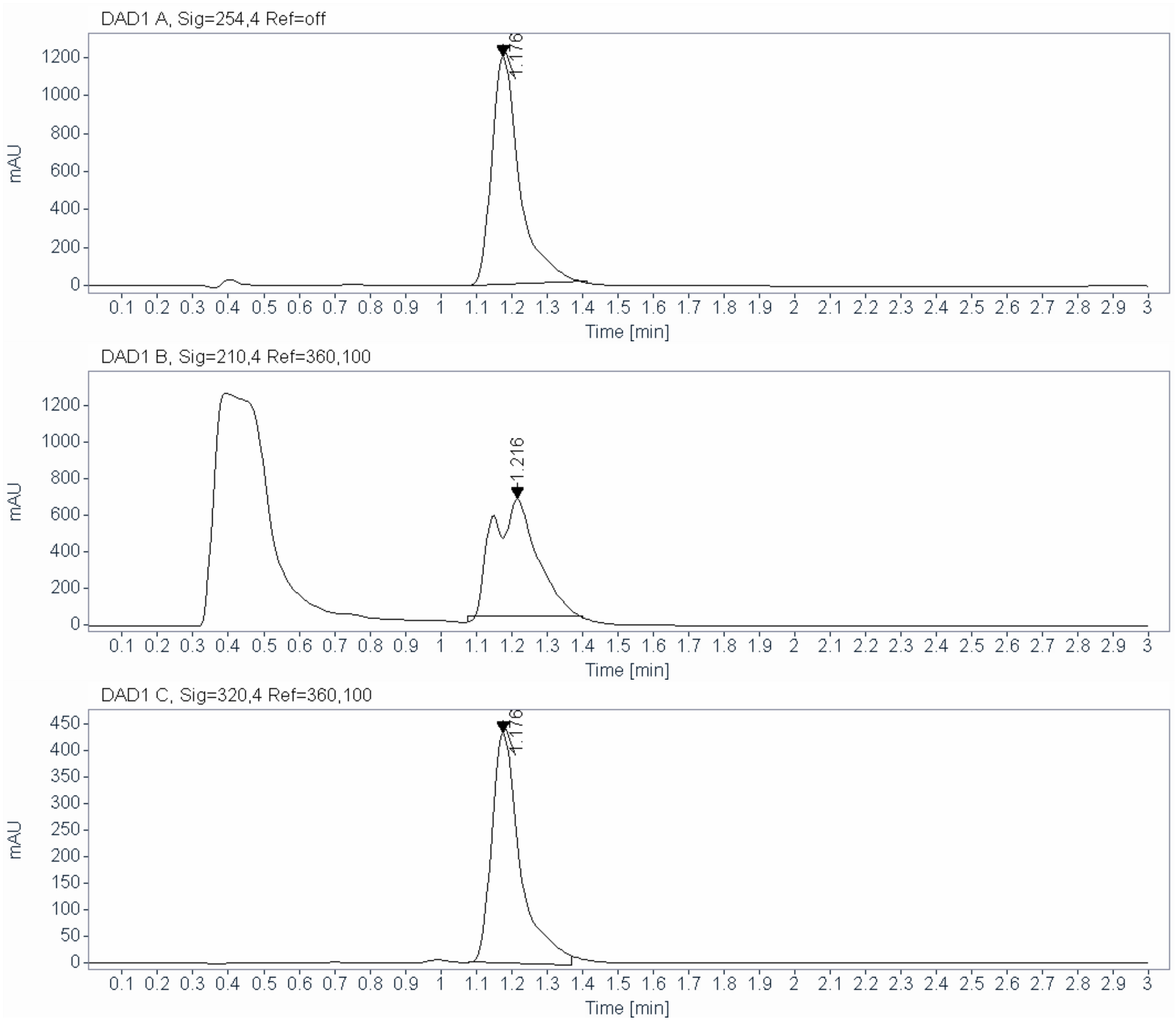

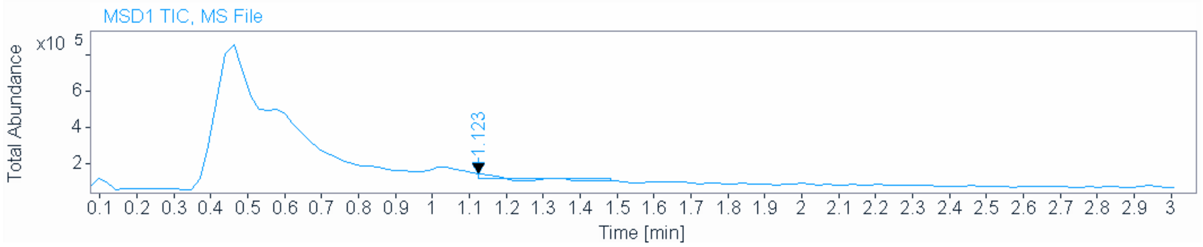

Agilent Technologies

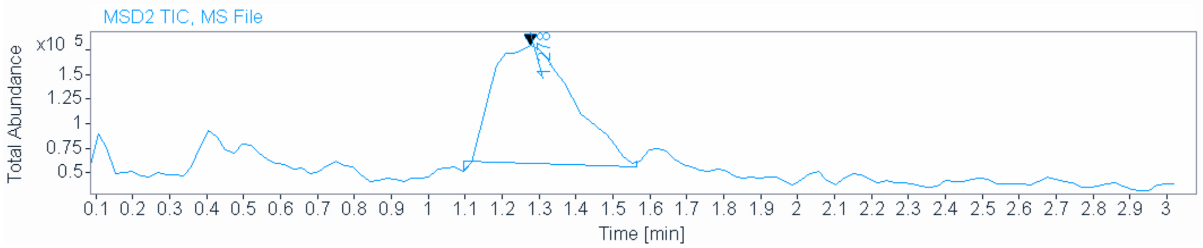

Signal: DAD1 A, Sig=254,4 Ref=off

RT [min]	Type	Width [min]	Area	Height	Area%	Name
1.176	MM	0.0910	6545.7134	1198.2708	100.0000	
		Sum	6545.7134			

Signal: DAD1 B, Sig=210,4 Ref=360,100

RT [min]	Type	Width [min]	Area	Height	Area%	Name
1.216	MM	0.1439	5592.1113	647.7466	100.0000	
		Sum	5592.1113			

Signal: DAD1 C, Sig=320,4 Ref=360,100

RT [min]	Type	Width [min]	Area	Height	Area%	Name
1.176	MM	0.0932	2422.1914	433.1400	100.0000	
		Sum	2422.1914			

Signal: MSD1 TIC, MS File

RT [min]	Type	Width [min]	Area	Height	Area%	Name
1.123	MM	0.0404	66871.4219	27585.1270	100.0000	
		Sum	66871.4219			

Signal: MSD2 TIC, MS File

RT [min]	Type	Width [min]	Area	Height	Area%	Name
1.278	MM	0.2543	1829926.1250	119922.6719	100.0000	
		Sum	1829926.125			

1.278 - 1.277 (005-17-KP9034.D)

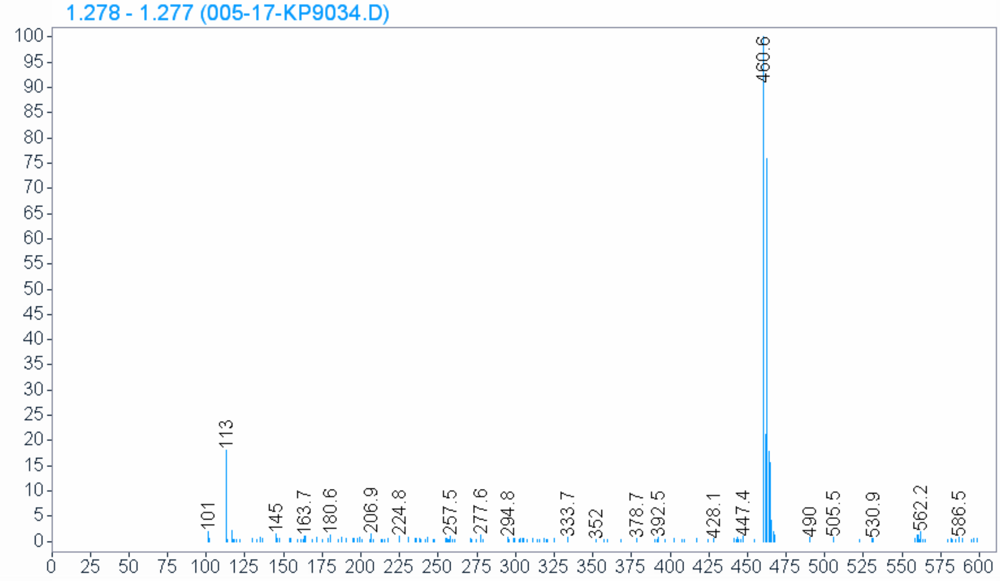

No peak data available for signal, cannot extract peak spectra, MSD1, 005-17-KP9034.D

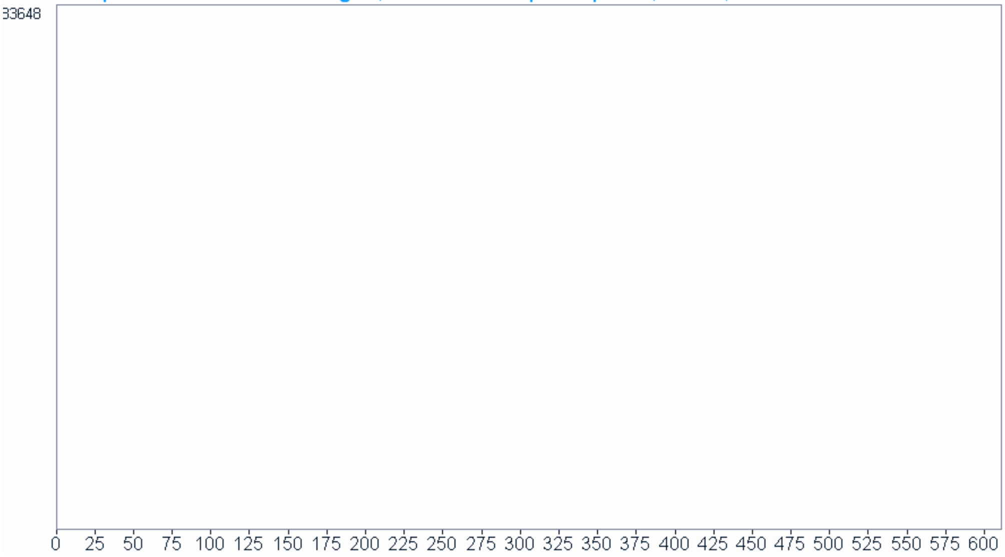

Compound Name: (*Z*)-*N*-(5-(3,4-dichlorobenzylidene)-4-oxo-4,5-dihydrothiazol-2-yl)naphthalene-1-sulfonamide

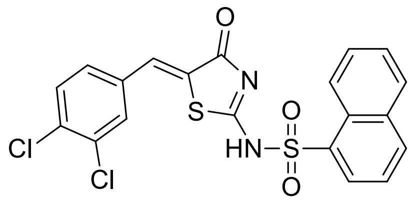

Chemical Formula: $C_{20}H_{12}Cl_2N_2O_3S_2$
Exact Mass: 461.97
Molecular Weight: 463.36

Compound Code: 36 (KP9004)

Obtained Weight & Yield: 155 mg (68%)

Purity (by LCMS and ^{1}H NMR): > 98% by ^{1}H-NMR and LCMS

Appearance: pale orange solid

Solubility: DMSO, slightly soluble in acetone and methanol.

Melting Point: > 201 °C (dec.)

TLC Rf (and conditions): N/A

IR Analysis (including assignment): IR (neat): $ν_{max}$ = 3155 (N-H), 3060, 2987, 2780 (C-H aromatic), 1706 (C=O), 1557 (C-C aromatic), 1315 (sulfonamide), 1129 (C-N), 766 (C-Cl) cm^{-1}

^{1}H NMR Analysis: ^{1}H NMR (600 MHz, DMSO) δ 13.29 (s, 1H, br, NH), 8.61 (d, *J* = 8.6 Hz, 1H), 8.30 (d, *J* = 7.5 Hz, 2H), 8.12 (d, *J* = 8.2 Hz, 1H), 7.94 (d, *J* = 1.8 Hz, 1H), 7.85 (d, *J* = 8.4 Hz, 1H), 7.77 (t, *J* = 7.8 Hz, 1H), 7.74 (s, 1H) 7.72 – 7.67 (m 2H), 7.61 (dd, *J* = 8.4, 1.9 Hz, 1H) ppm.
Ethanol at 1.05 ppm (0.20%) and some starting material at 4.05 ppm (0.66%)

^{13}C NMR Analysis: ^{13}C NMR (151 MHz, DMSO) δ 165.4 (br), 165.2 (br), 135.2, 134.8, 133.8, 133.6, 133.1, 132.5, 132.1, 131.6, 130.7, 129.0, 129.0, 128.3, 128.2, 127.6, 127.1, 124.8, 124.6, 124.3 (br) ppm.

MS Analysis (low res): LRMS (ESI-) *m/z*: 461 (*M*-H, $C_{20}H_{11}{}^{35}Cl_2N_2O_3S_2$, 100), 463 (*M*-H, $C_{20}H_{11}{}^{35/37}Cl_2N_2O_3S_2$, 65), 465 (*M*-H, $C_{20}H_{11}{}^{37}Cl_2N_2O_3S_2$, 15)

MS Analysis (high res): Exact mass calculated for $C_{20}H_{11}Cl_2N_2O_3S_2$ $[M\text{-H}]^-$, 460.9600. Found 460.9596.

HPLC method details: Column: Zorbax SB-C18 Rapid Resolution HT 2.1x50mm 1.8-Micron; Method: LCMS ISOCRATIC 80%B_3 MINS.M filename: KP9004; Peak retention time: 1.177 mins; Area (%): 100.

Procedure: To a 10mL microwave vial was added *N*-(4-oxo-4,5-dihydrothiazol-2-yl)naphthalene-1-sulfonamide (147 mg, 0.49 mmol), 3,4-dichlorobenzaldehyde (111 mg, 0.54 mmol, 1.1 eq), ethanol (3 mL) and a catalytic amount of the benzoic acid/piperidine catalyst (approximately 5 drops). The suspension was heated by microwave irradiation (120 °C, 200 W) for 30 min. A precipitate formed upon cooling and the precipitate was collected to give the desired product as a pale orange solid (155 mg, 68%).

Other analyses, reference papers, previously obtained data, comments, etc:

PerkinElmer Spectrum Version 10.4.00
Thursday, 26 November 2020 11:55 AM

Analyst	research
Date	Thursday, 26 November 2020 11:55 AM

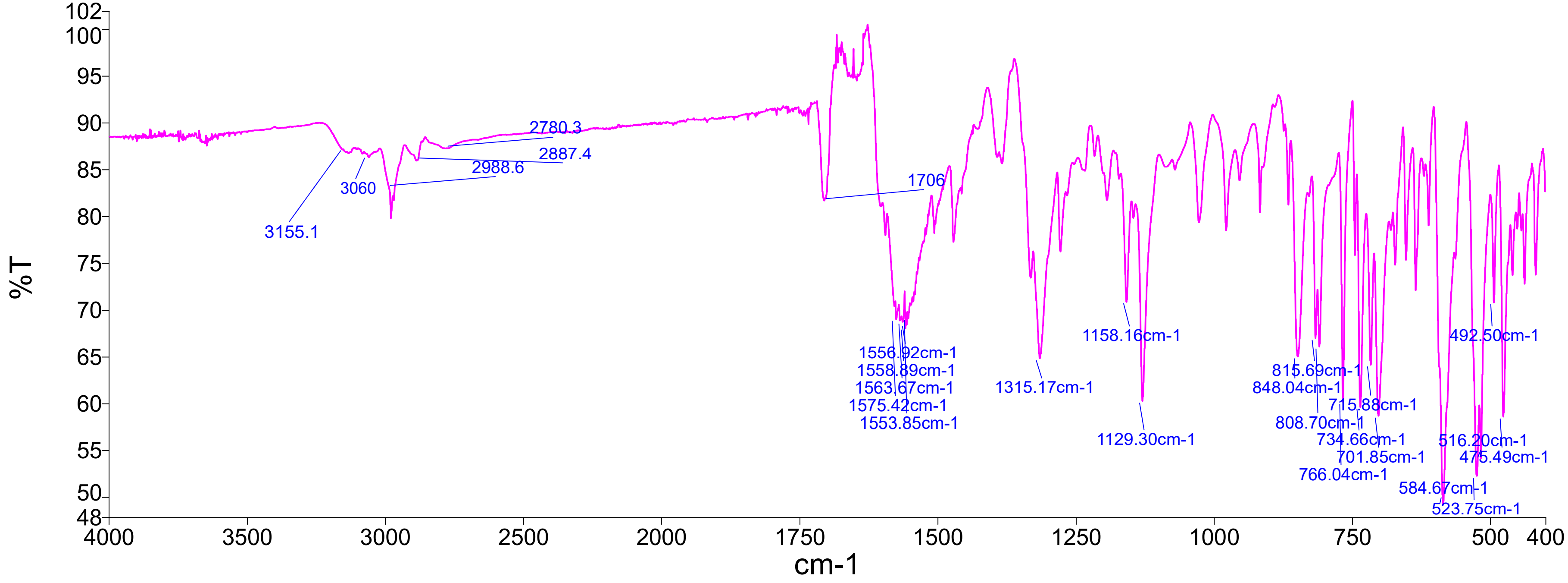

Sample Name	Description	Quality Checks
kp9004	Sample 178 By research Date Thursday, November 26 2020	The Quality Checks do not report any warnings for the sample.

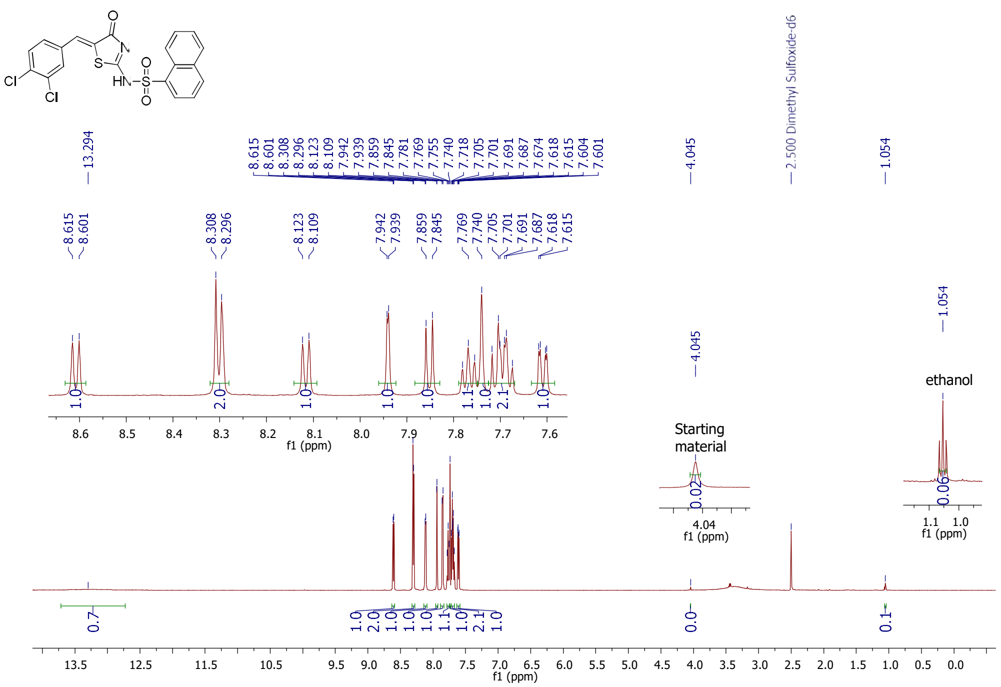

Cl
Cl
O
N
S
HN
S
O
O
2.500 Dimethyl Sulfoxide-d6
Starting material
ethanol
f1 (ppm)

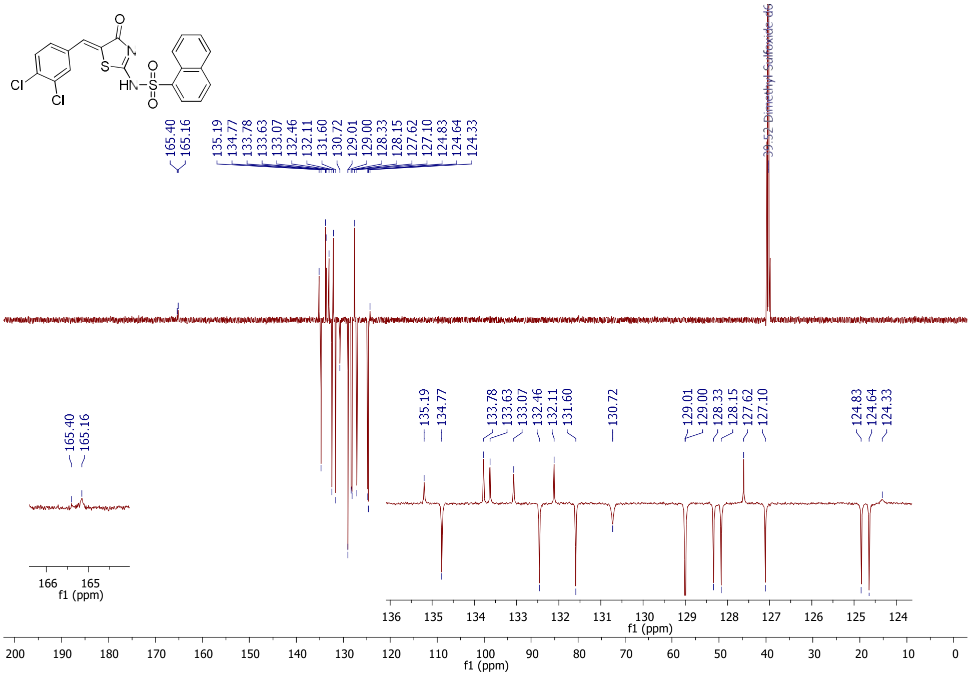

Cl
Cl
O
N
S
HN
S
O
O
39.52 Dimethyl Sulfoxide-d6
f1 (ppm)

LCMS Report

Agilent Technologies

**Data file:**	D:\Chem32\1\Data\KP\KP_DS_NOV4 2020-11-02 13-34-37\002-20-KP9004.D		
**Sample name:**	KP9004		
**Description:**			
**Sample amount:**	0.000	**Sample type:**	Sample
**Instrument:**	LCMS	**Location:**	20
**Injection date:**	11/2/2020 1:40:50 PM	**Injection:**	1 of 1
**Acq. method:**	LCMS ISOCRATIC 80% B_3 MINS.M	**Injection volume:**	2.000
**Analysis method:**	LCMS ISOCRATIC 80%B_3 MINS.M	**Acq. operator:**	SYSTEM
**Last changed:**	10/8/2020 2:52:31 PM		

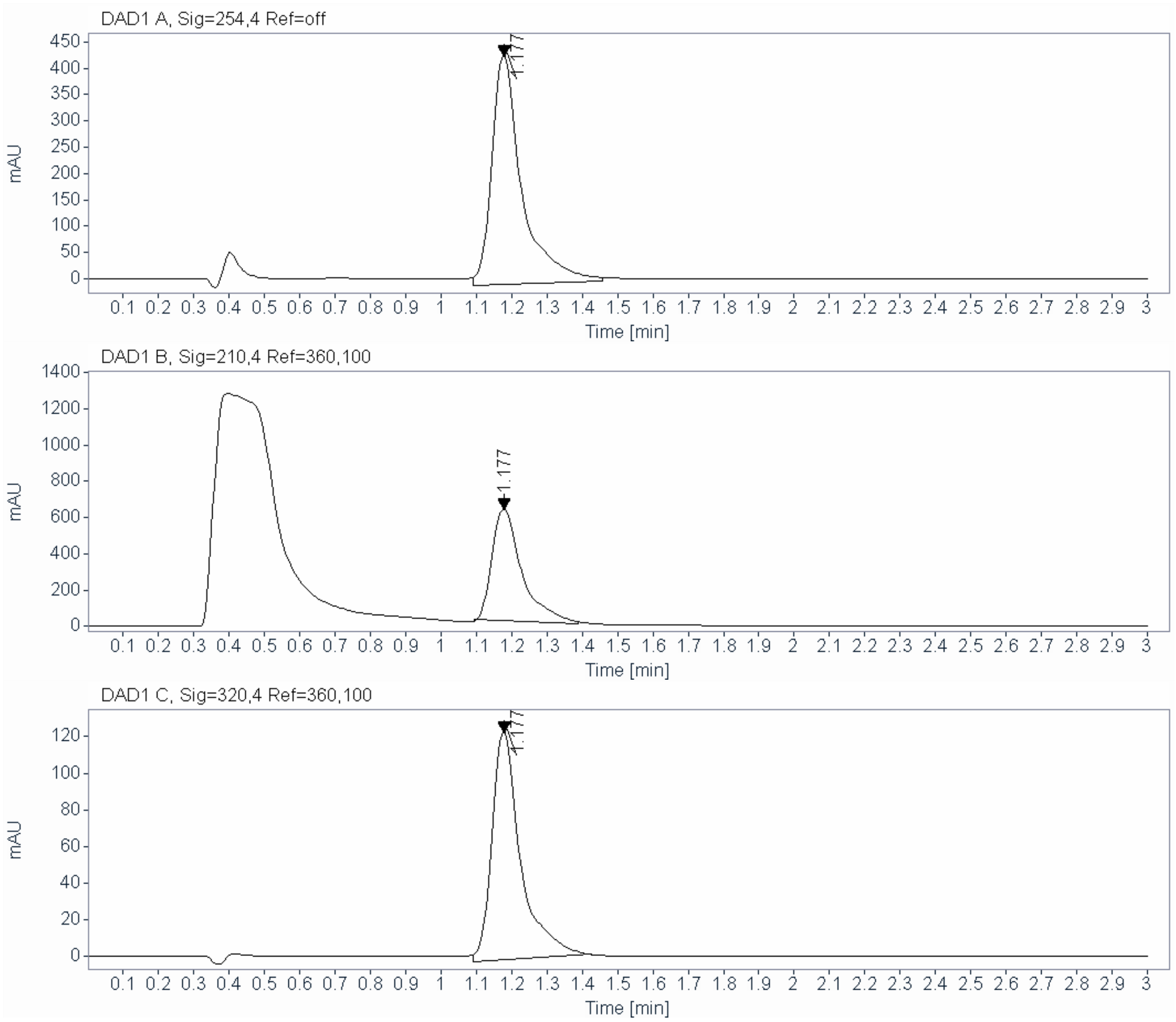

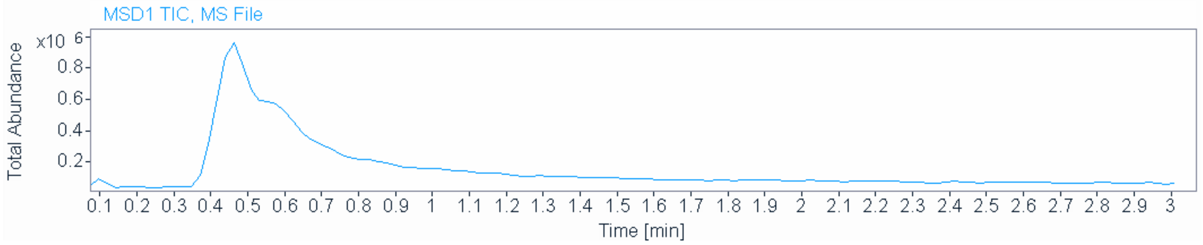

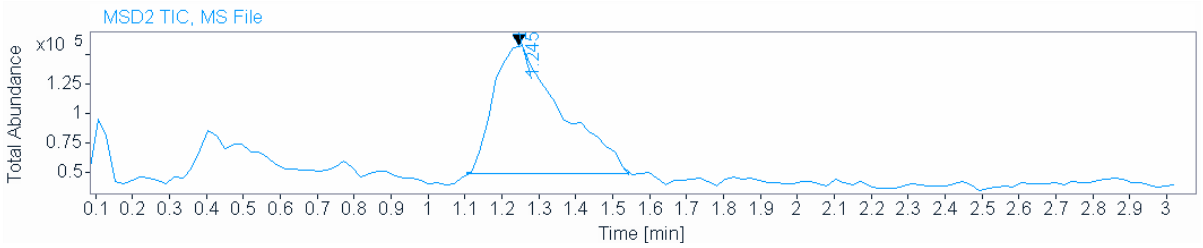

Signal:	DAD1 A, Sig=254,4 Ref=off				
RT [min] Type	Width [min]	Area	Height	Area%	Name
1.177 MM	0.0983	2574.1101	436.3784	100.0000	
	Sum	2574.1101			

Signal:	DAD1 B, Sig=210,4 Ref=360,100				
RT [min] Type	Width [min]	Area	Height	Area%	Name
1.177 MM	0.0974	3610.6948	617.6491	100.0000	
	Sum	3610.6948			

Signal:	DAD1 C, Sig=320,4 Ref=360,100				
RT [min] Type	Width [min]	Area	Height	Area%	Name
1.177 MM	0.0930	696.3872	124.8024	100.0000	
	Sum	696.3872			

Signal:	MSD2 TIC, MS File				
RT [min] Type	Width [min]	Area	Height	Area%	Name
1.245 MM	0.2083	1385323.625 0	110863.0313	100.0000	
	Sum	1385323.625			

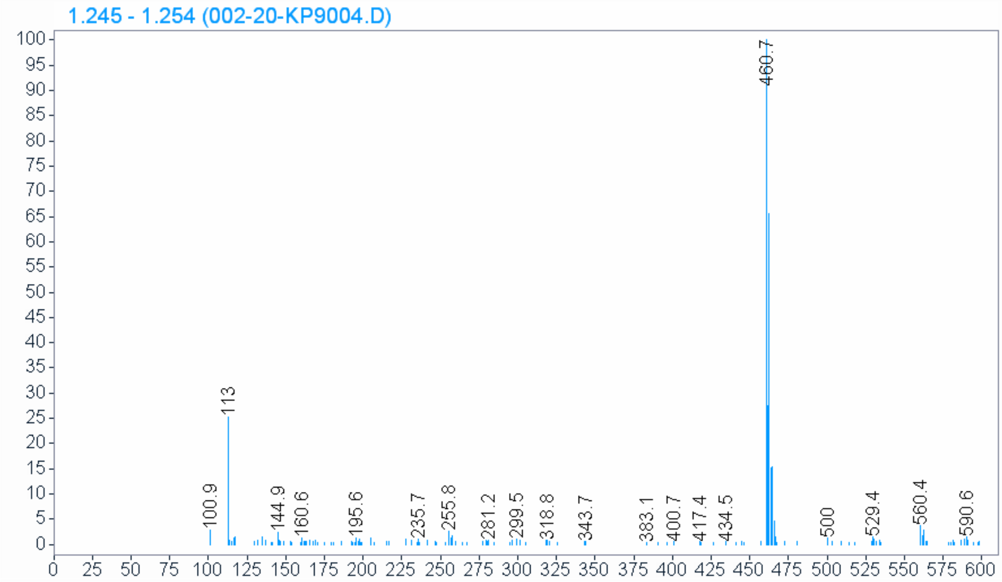
1.245 - 1.254 (002-20-KP9004.D)
460.7
113
100.9
144.9
160.6
195.6
235.7
255.8
281.2
299.5
318.8
343.7
383.1
400.7
417.4
434.5
500
529.4
560.4
590.6

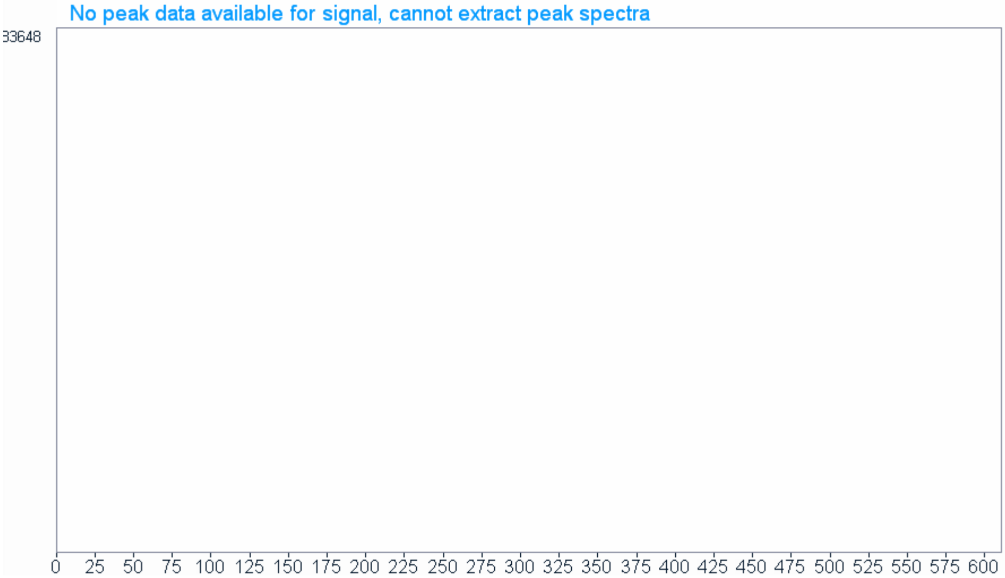
No peak data available for signal, cannot extract peak spectra
33648

Name of Researcher: Kate Prichard

Compound Name: (*Z*)-*N*-(5-(2,6-dichlorobenzylidene)-4-oxo-4,5-dihydrothiazol-2-yl)naphthalene-1-sulfonamide

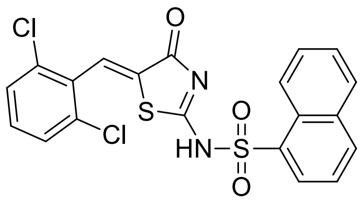

Chemical Formula: $C_{20}H_{12}Cl_2N_2O_3S_2$
Exact Mass: 461.97
Molecular Weight: 463.36

Code: 37 (KP9190)

Obtained Weight & Yield: 127 mg (56%)

Purity (by LCMS and ^{1}H NMR): > 99% by ^{1}H-NMR and LCMS

Appearance: off white solid

Solubility: DMSO, slightly soluble in acetone and methanol.

Melting Point: 232 – 235 °C

TLC Rf (and conditions): N/A

IR Analysis (including assignment): IR (neat): ν_{max} = 3237 (N-H), 2980 (C-H aromatic), 1736 (C=O), 1569 (C-C aromatic), 1335 (sulfonamide), 1126 (C-N), 767 (C-Cl) cm^{-1}

^{1}H NMR Analysis: ^{1}H NMR (400 MHz, DMSO) δ 8.57 (d, *J* = 8.5 Hz, 1H), 8.30 (d, *J* = 8.2 Hz, 1H), 8.21 (d, *J* = 7.2 Hz, 1H), 8.12 (d, *J* = 8.2 Hz, 1H), 7.76 (t, *J* = 7.2 Hz, 1H), 7.72 – 7.61 (m, 5H), 7.56 – 7.52 (m, 1H) ppm.
NH exchanging – not visible.
Ethanol impurity at 1.06 ppm (0.63%) and aldehyde starting material at 10.37 ppm (0.19%).

^{13}C NMR Analysis: ^{13}C NMR (101 MHz, DMSO) δ 165.3, 164.9, 135.0, 134.8, 133.8, 133.1, 132.1, 131.2, 130.5, 130.0, 129.04, 128.95, 128.89 (2C), 128.3, 128.1, 127.6, 127.1, 124.8, 124.6 ppm.
2C determined by 2D NMR

MS Analysis (low res): LRMS (ESI-): 461 (*M*-H, $C_{20}H_{11}{}^{35}Cl_2N_2O_3S_2$, 100), 463 (*M*-H, $C_{20}H_{11}{}^{35}Cl^{37}ClN_2O_3S_2$, 65), 465 (*M*-H, $C_{20}H_{11}{}^{37}Cl_2N_2O_3S_2$, 19); LRMS (ESI+): 463 (*M*-H, $C_{20}H_{13}{}^{35}Cl_2N_2O_3S_2$, 100), 465 (*M*-H, $C_{20}H_{13}{}^{35}Cl^{37}ClN_2O_3S_2$, 85), 467 (*M*-H, $C_{20}H_{13}{}^{37}Cl_2N_2O_3S_2$, 20).

HPLC method details: Column: Zorbax SB-C18 Rapid Resolution HT 2.1x50mm 1.8-Micron; Method: LCMS ISOCRATIC 60%B_3 MINS.M filename: KP9190; Peak retention time: 2.274 mins; Area (%): 100.

Procedure: To a 10 mL microwave vial was added *N*-(4-oxo-4,5-dihydrothiazol-2-yl)naphthalene-1-sulfonamide (149 mg, 0.49 mmol), 2,6-dichlorobenzaldehyde (128 mg, 0.735 mmol, 1.1 eq), ethanol (3 mL) and a catalytic amount of the benzoic acid/piperidine catalyst (approximately 5 drops). The suspension was heated by microwave irradiation (120 °C, 200 W) for 1.5 h. A precipitate formed upon cooling. The solid was collected by vacuum filtration to give the desired product as an off white solid (127 mg, 56%).

Other analyses, reference papers, previously obtained data, comments, etc:

PerkinElmer Spectrum Version 10.4.00
Monday, 10 May 2021 11:22 AM

Analyst research
Date Monday, 10 May 2021 11:22 AM

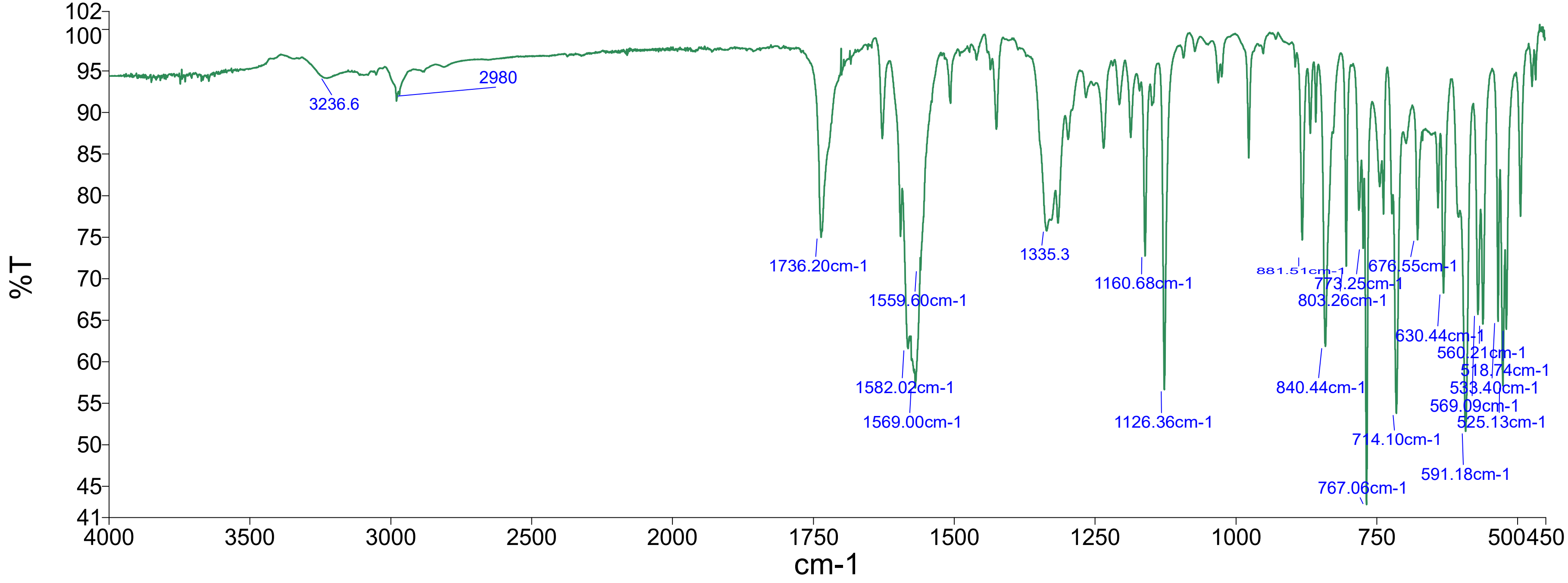

Sample Name	Description	Quality Checks
kp9190	Sample 012 By research Date Monday, May 10 2021	The Quality Checks do not report any warnings for the sample.

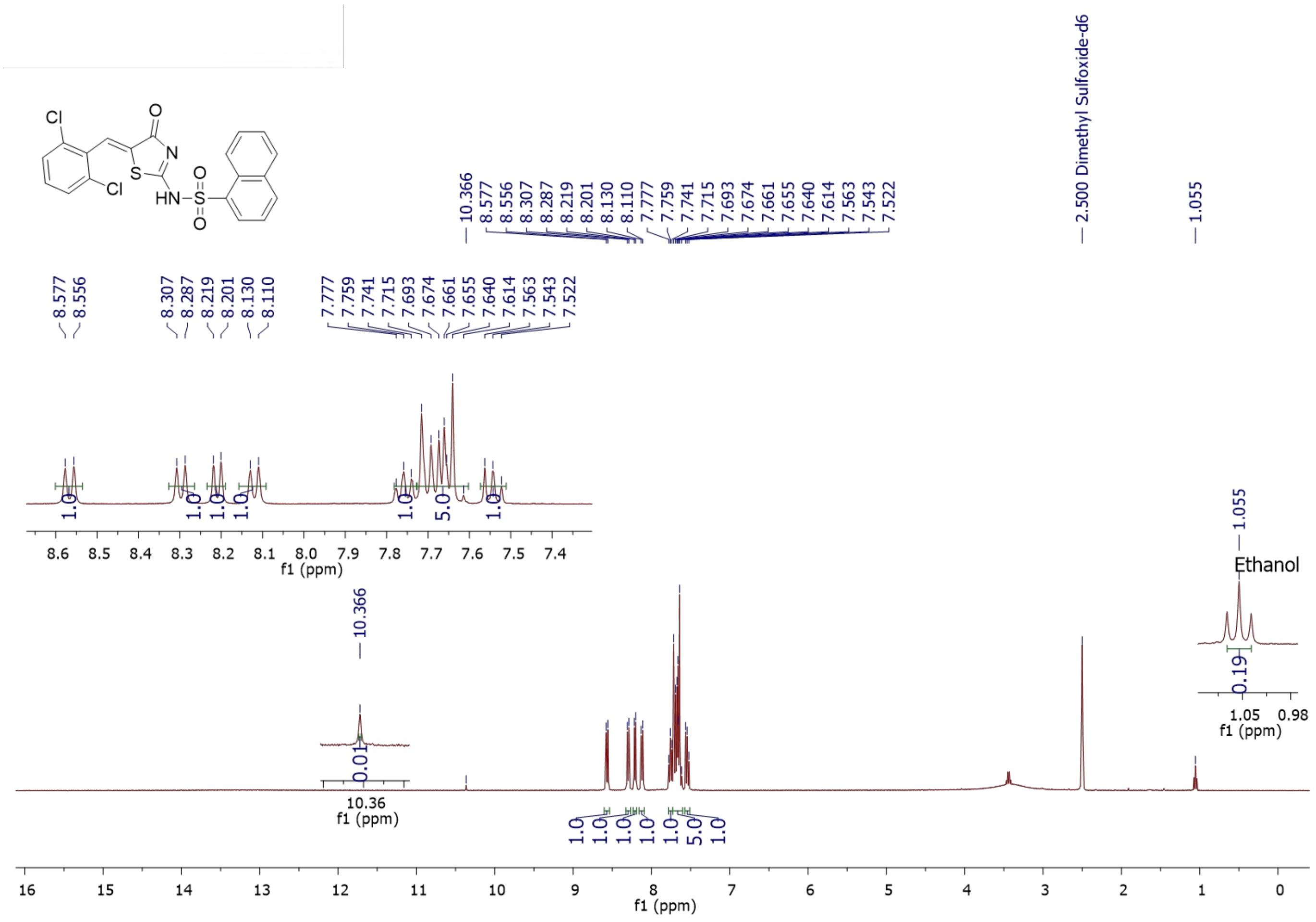
Cl
O
N
S
Cl
HN
O
S
O
10.366
8.577
8.556
8.307
8.287
8.219
8.201
8.130
8.110
7.777
7.759
7.741
7.715
7.693
7.674
7.661
7.655
7.640
7.614
7.563
7.543
7.522
2.500 Dimethyl Sulfoxide-d6
1.055
Ethanol
0.19
1.05 0.98
0.01
10.36
f1 (ppm)

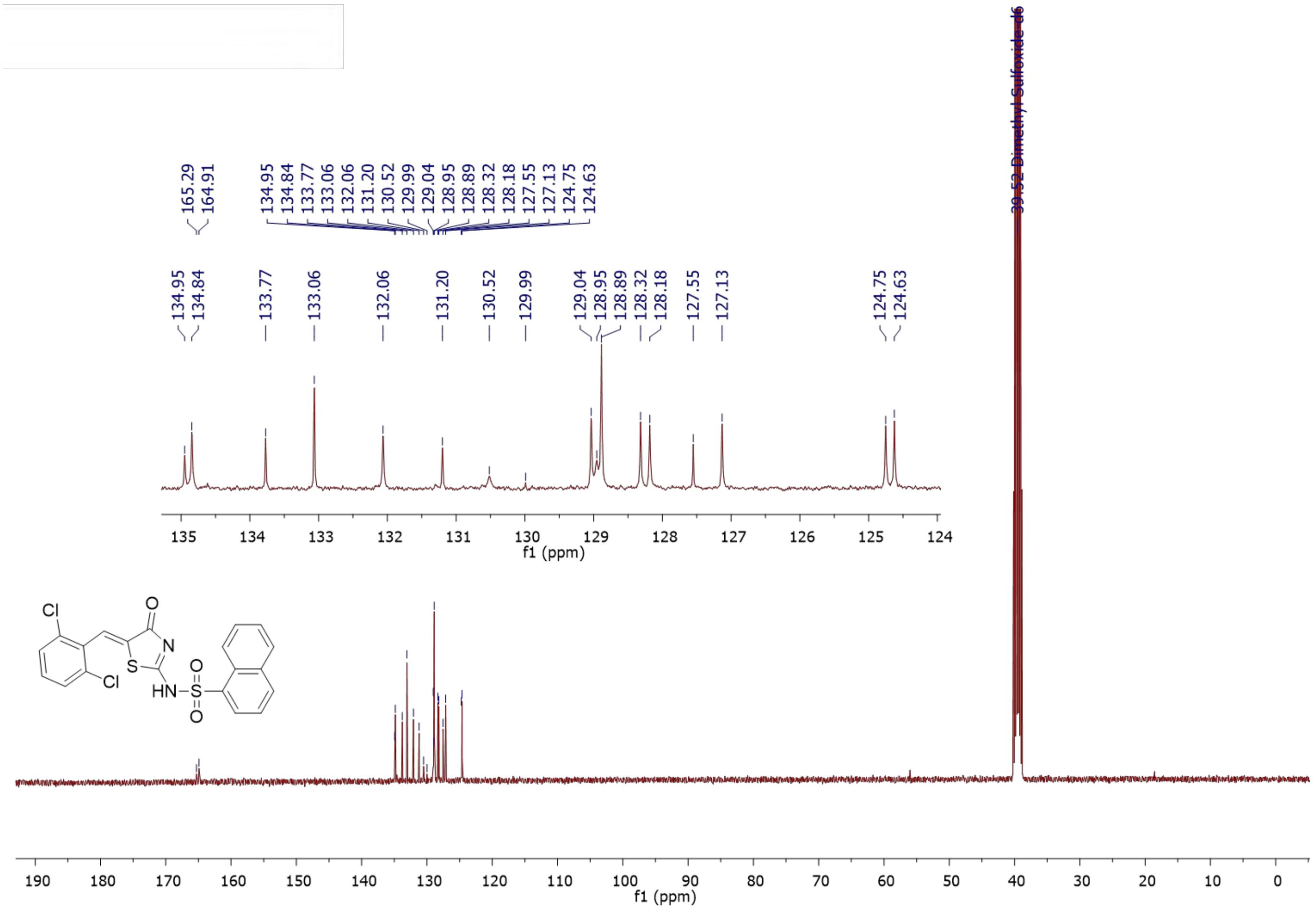
165.29
164.91
134.95
134.84
133.77
133.06
132.06
131.20
130.52
129.99
129.04
128.95
128.89
128.32
128.18
127.55
127.13
124.75
124.63
39.52 Dimethyl Sulfoxide-d6
Cl
O
N
S
Cl
HN
O
S
O
f1 (ppm)

LCMS Report

Agilent Technologies

**Data file:**	D:\Chem32\1\Data\KP\KP_3DEC 2020-12-03 11-15-05\003-19-KP9190.D		
**Sample name:**	KP9190		
**Description:**			
**Sample amount:**	0.000	**Sample type:**	Sample
**Instrument:**	LCMS	**Location:**	19
**Injection date:**	12/3/2020 11:25:56 AM	**Injection:**	1 of 1
**Acq. method:**	LCMS ISOCRATIC 60% B_3MINS.M	**Injection volume:**	2.000
**Analysis method:**	LCMS ISOCRATIC 60%B_3MINS.M	**Acq. operator:**	SYSTEM
**Last changed:**	5/19/2016 3:52:53 PM		

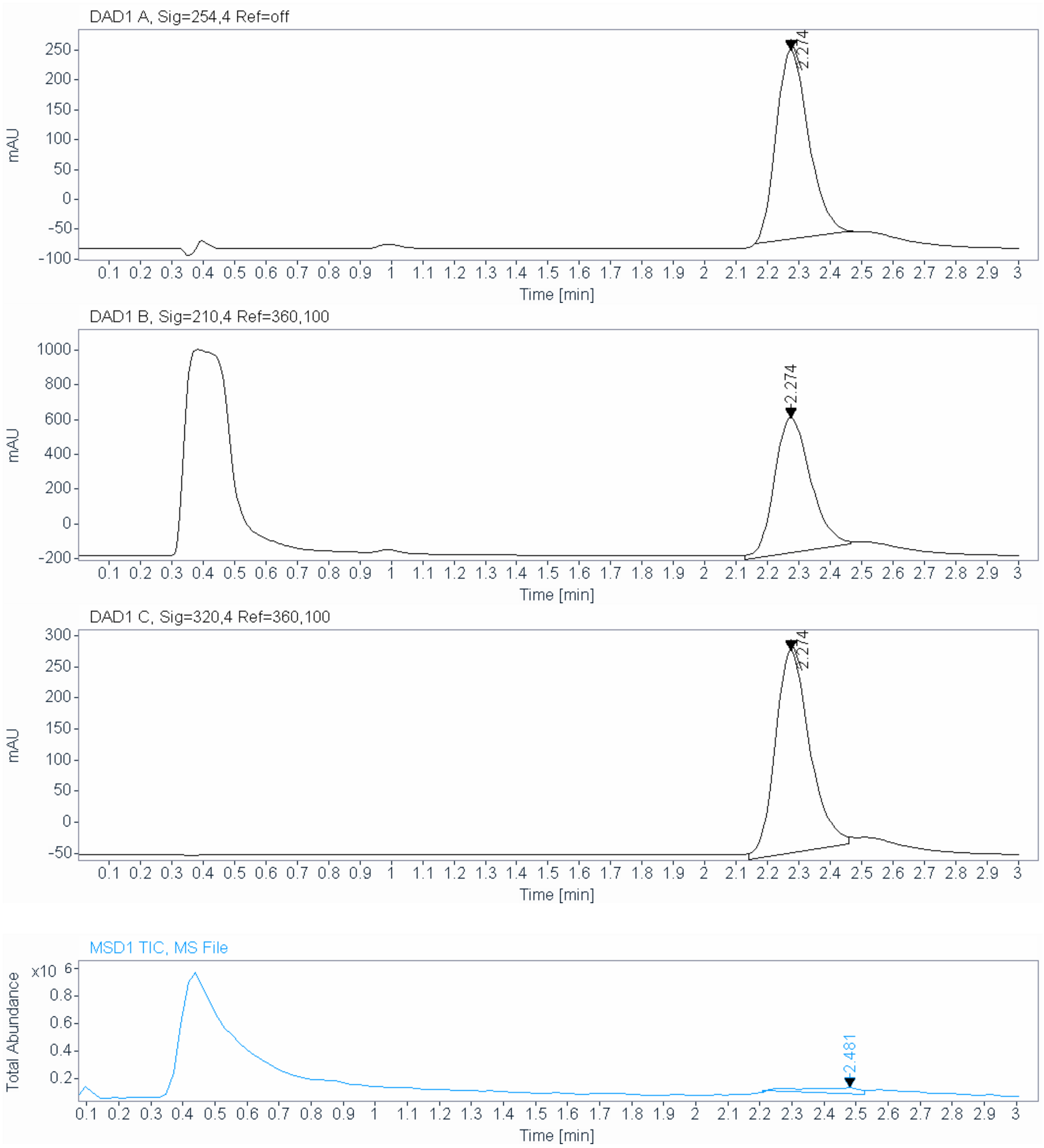

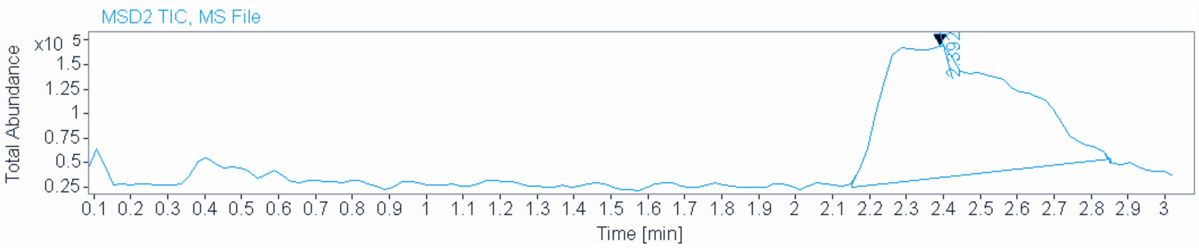

Signal: DAD1 A, Sig=254,4 Ref=off

RT [min]	Type	Width [min]	Area	Height	Area%	Name
2.274	MM	0.1193	2270.3408	317.1933	100.0000	
		Sum	2270.3408			

Signal: DAD1 B, Sig=210,4 Ref=360,100

RT [min]	Type	Width [min]	Area	Height	Area%	Name
2.274	MM	0.1333	6227.9136	778.3921	100.0000	
		Sum	6227.9136			

Signal: DAD1 C, Sig=320,4 Ref=360,100

RT [min]	Type	Width [min]	Area	Height	Area%	Name
2.274	MM	0.1272	2491.5298	326.5450	100.0000	
		Sum	2491.5298			

Signal: MSD1 TIC, MS File

RT [min]	Type	Width [min]	Area	Height	Area%	Name
2.481	MM	0.1943	524257.1875	44965.6328	100.0000	
		Sum	524257.1875			

Signal: MSD2 TIC, MS File

RT [min]	Type	Width [min]	Area	Height	Area%	Name
2.392	MM	0.4098	3343051.750<br>0	135974.7656	100.0000	
		Sum	3343051.750			

Agilent Technologies

2.392 - 2.401 (003-19-KP9190.D)

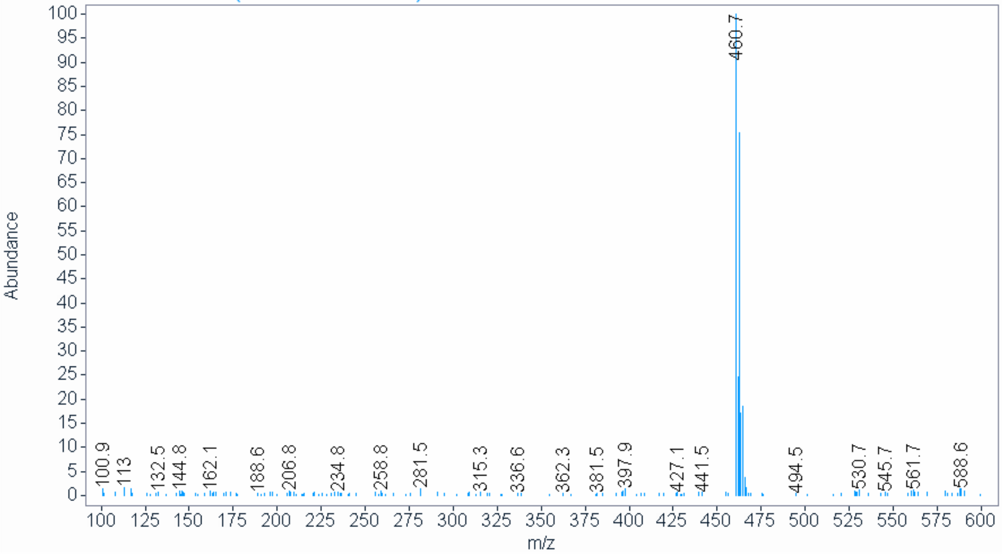

2.481 - 2.481 (003-19-KP9190.D)

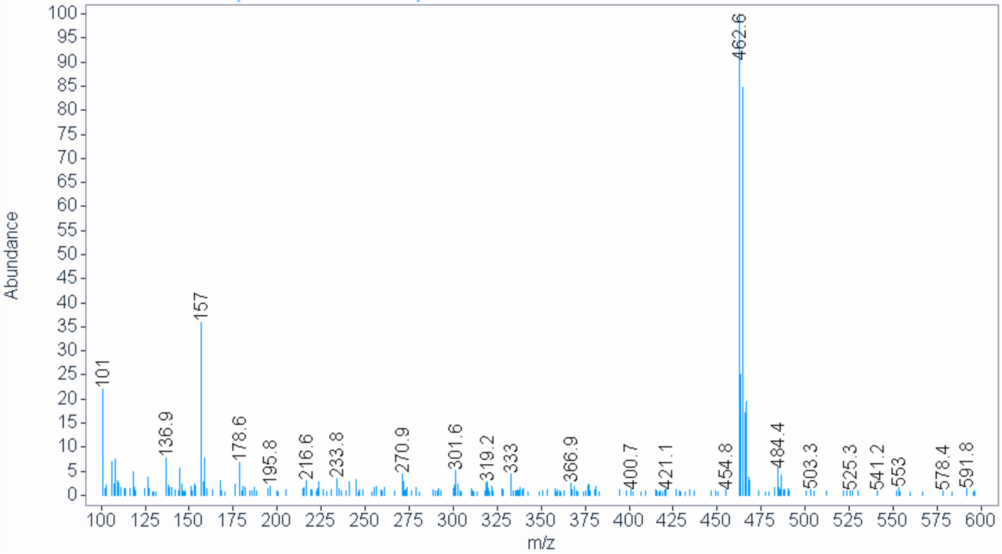

Compound Name: (*Z*)-*N*-(5-(3,4-difluorobenzylidene)-4-oxo-4,5-dihydrothiazol-2-yl)naphthalene-1-sulfonamide

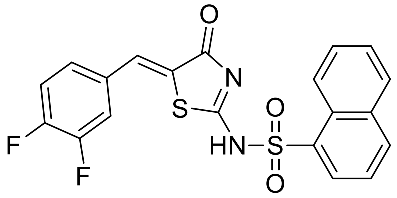

Chemical Formula: $C_{20}H_{12}F_2N_2O_3S_2$
Exact Mass: 430.03
Molecular Weight: 430.45

Compound Code: 38 (KP8196)

Obtained Weight & Yield: 92 mg (44%)

Purity (by LCMS and ^{1}H NMR): > 98% by ^{1}H-NMR and LCMS

Appearance: Off white solid

Solubility: DMSO, slightly soluble in acetone and methanol.

Melting Point: > 125 °C (dec.)

TLC Rf (and conditions): N/A

IR Analysis (including assignment): IR (neat): ν_{max} = 3280 (N-H), 2983, 2887 (C-H aromatic), 1709 (C=O), 1587 (C-C aromatic), 1318 (sulfonamide), 1125 (C-N) cm^{-1}

^{1}H NMR Analysis: ^{1}H NMR (600 MHz, DMSO) δ 13.24 (s, 1H, br, NH), 8.62 (d, *J* = 8.3 Hz, 1H), 8.30 (t, *J* = 7.9 Hz, 2H), 8.11 (d, *J* = 7.8 Hz, 1H), 7.77 – 7.68 (m, 6H), 7.52 (s, 1H) ppm.
Ethanol at 1.05 ppm (0.85%) and some piperidine at 1.54 ppm (0.79%)

^{13}C NMR Analysis: ^{13}C NMR (151 MHz, DMSO) δ 165.67, 150.39 (dd, $^{1/2}J_{CF}$ = 12.86, 252.58 Hz), 149.63 (dd, $^{1/2}J_{CF}$ = 13.18, 247.53 Hz), 135.33, 134.68, 133.78, 131.01, 130.76 (unresolved quartet), 128.99, 128.28, 128.12, 127.64, 127.08, 126.87 (unresolved quartet), 124.89, 124.64, 123.76 (br), 119.65 (d, $^2J_{CF}$ = 17.83 Hz), 118.70 (d, $^2J_{CF}$ = 17.89 Hz) ppm.
One carbon missing

^{19}F NMR Analysis: ^{19}F NMR (376 MHz, DMSO) δ -130.71, -133.36 ppm*

MS Analysis (low res): LRMS (ESI-) *m/z* (%): 429 (*M*-H, $C_{20}H_{11}F_2N_2O_3S_2$, 100); (ESI+) *m/z* (%): 431 (*M*+H, $C_{20}H_{13}F_2N_2O_3S_2$, 100)

MS Analysis (high res): Exact mass calculated for $C_{20}H_{11}F_2N_2O_3S_2$ [*M*-H]$^-$, 429.0200. Found 429.0182.

HPLC method details: Column: Zorbax SB-C18 Rapid Resolution HT 2.1x50mm 1.8-Micron; Method: LCMS ISOCRATIC 60%B_3MINS.M filename: KP8196; Peak retention time: 2.12 mins; Area (%): 100.

Procedure: To a 10mL microwave vial was added *N*-(4-oxo-4,5-dihydrothiazol-2-yl)naphthalene-1-sulfonamide (155 mg, 0.49 mmol), 3,4-difluorobenzaldehyde (0.06 mL, 0.54 mmol, 1.1 eq), ethanol (3 mL) and a catalytic amount of the benzoic acid/piperidine catalyst (approximately 5 drops). The suspension was heated by microwave irradiation (120 °C, 200 W) for 30 min. A precipitate formed upon sonication and after cooling overnight the precipitate was collected to give the desired product as an off white solid (92 mg, 44%).

Other analyses, reference papers, previously obtained data, comments, etc:
* TFA internal standard, TFA internal reference peak set at -75.9 ppm.[1] The peak shift is consistent with published values.[2]

[1]Exposing the Origins of Irreproducibility in Fluorine NMR Spectroscopy Angewandte. Angew. Chemie - Int. Ed. 2018, 57, 9528–9533.

[2]An Overview of Fluorine NMR. In Guide to Fluorine NMR for Organic Chemists; 2016; pp 9–53

PerkinElmer Spectrum Version 10.4.00
Thursday, 26 November 2020 11:54 AM

Analyst	research
Date	Thursday, 26 November 2020 11:54 AM

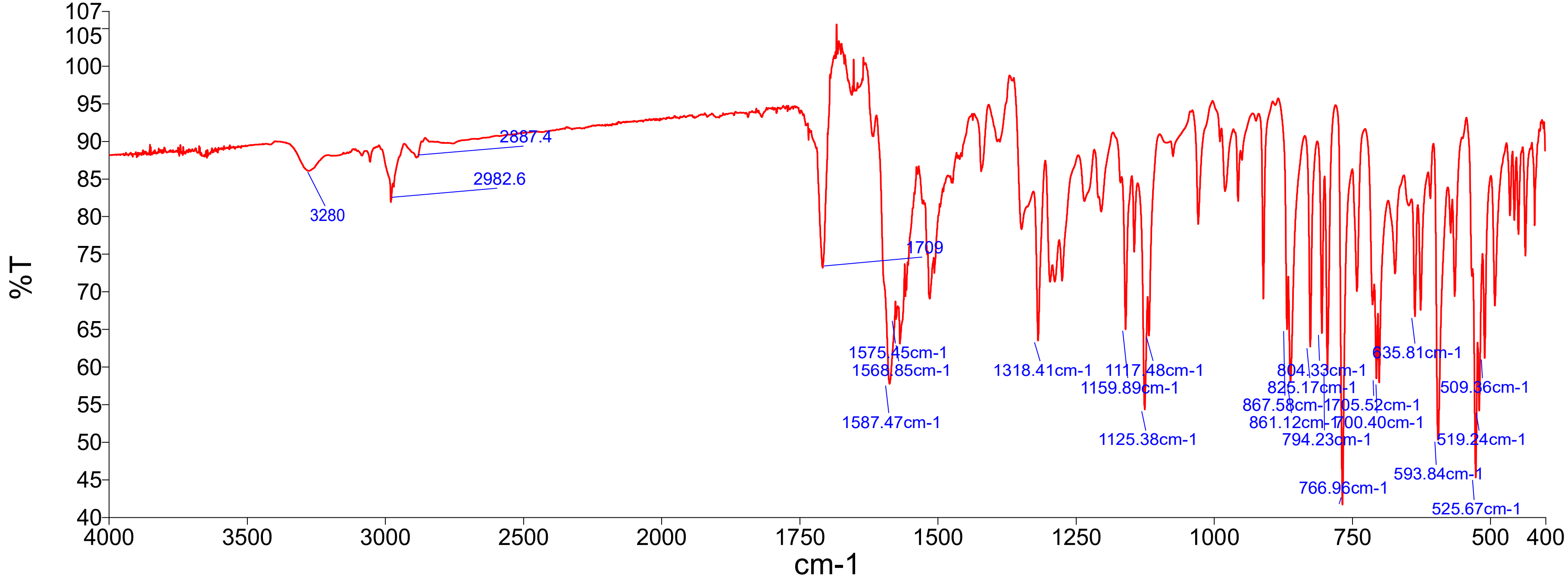

Sample Name	Description	Quality Checks
kp8196	Sample 176 By research Date Thursday, November 26 2020	The Quality Checks give rise to a Baseline High warning for the sample.

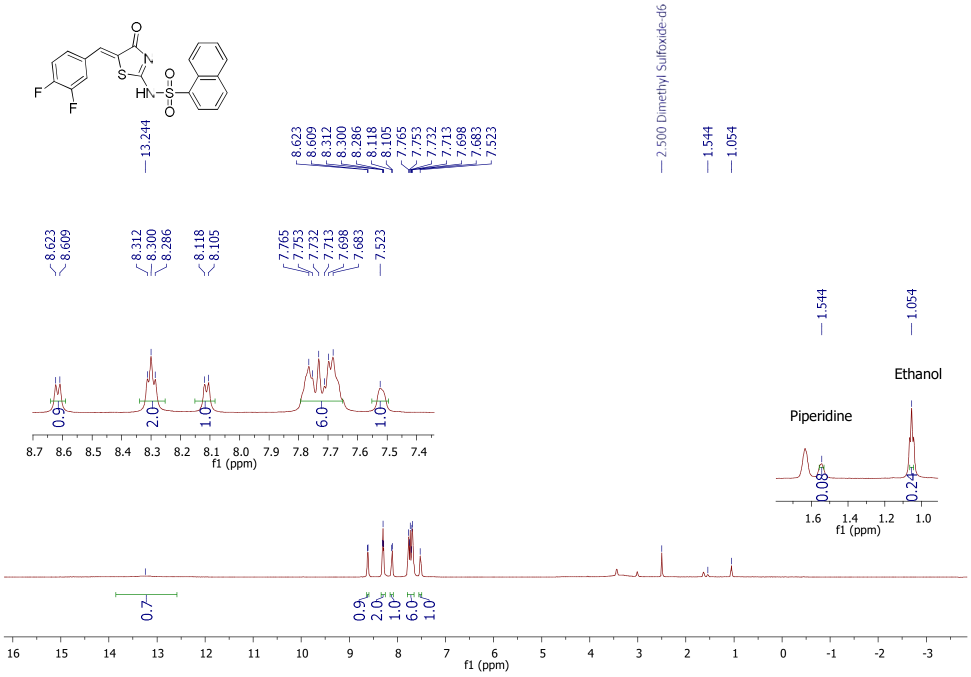

13.244
8.623
8.609
8.312
8.300
8.286
8.118
8.105
7.765
7.753
7.732
7.713
7.698
7.683
7.523
2.500 Dimethyl Sulfoxide-d6
1.544
1.054
Ethanol
Piperidine
f1 (ppm)

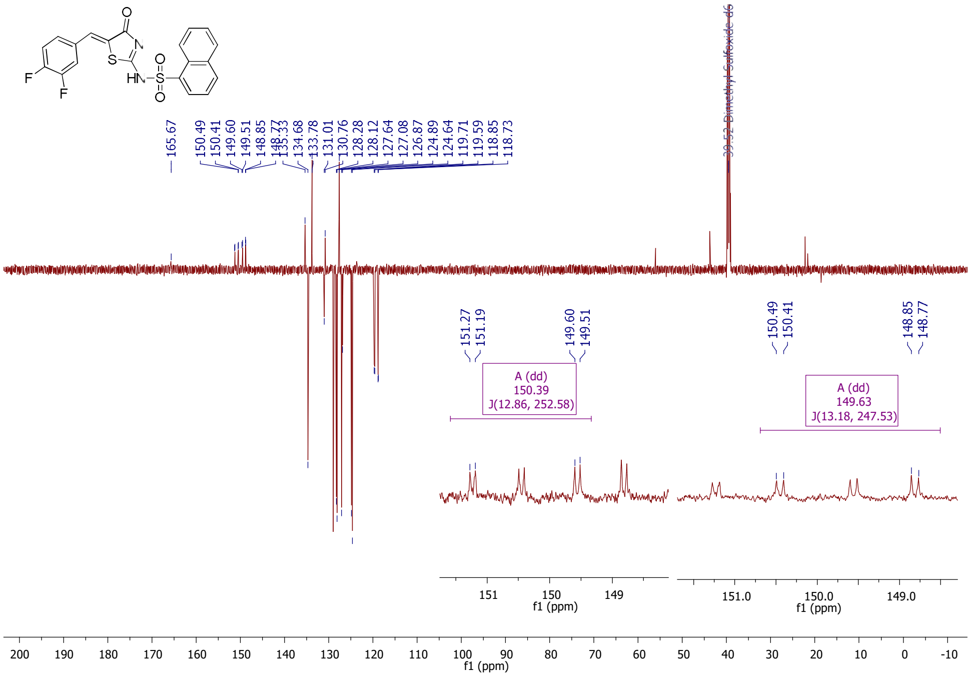

165.67
150.49
150.41
149.60
149.51
148.85
134.68
133.78
131.01
130.76
128.28
128.12
127.64
127.08
126.87
124.89
124.64
119.71
119.59
118.85
118.73
151.27
151.19
A (dd)
150.39
J(12.86, 252.58)
A (dd)
149.63
J(13.18, 247.53)
f1 (ppm)

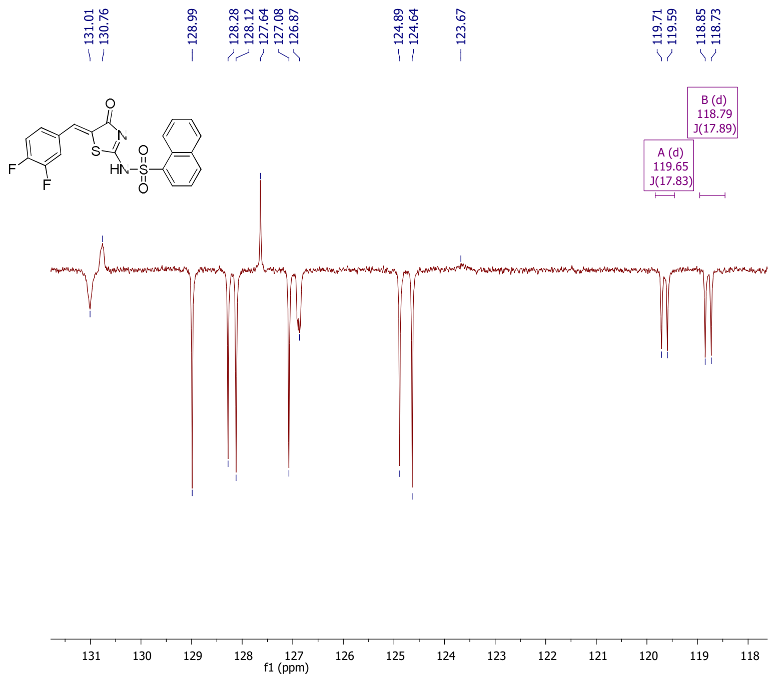

131.01
130.76
128.99
128.28
128.12
127.64
127.08
126.87
124.89
124.64
123.67
119.71
119.59
118.85
118.73
B (d)
118.79
J(17.89)
A (d)
119.65
J(17.83)
O
N
S
HN
S
O
O
F
F
f1 (ppm)

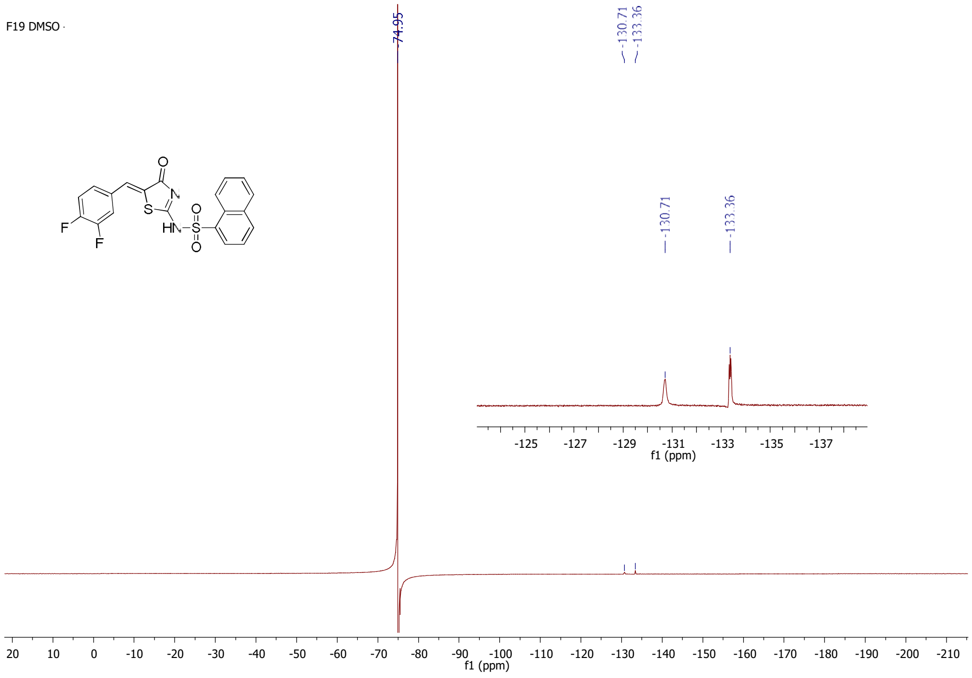

F19 DMSO
-74.95
-130.71
-133.36
O
N
S
HN
S
O
O
F
F
f1 (ppm)

LCMS Report

Agilent Technologies

**Data file:**	D:\Chem32\1\Data\KP\KP_DS_NOV1 2020-11-02 12-21-40\002-20-KP8196.D		
**Sample name:**	KP8196		
**Description:**			
**Sample amount:**	0.000	**Sample type:**	Sample
**Instrument:**	LCMS	**Location:**	20
**Injection date:**	11/2/2020 12:29:11 PM	**Injection:**	1 of 1
**Acq. method:**	LCMS ISOCRATIC 60% B_3MINS.M	**Injection volume:**	2.000
**Analysis method:**	LCMS ISOCRATIC 60%B_3MINS.M	**Acq. operator:**	SYSTEM
**Last changed:**	5/19/2016 3:52:53 PM		

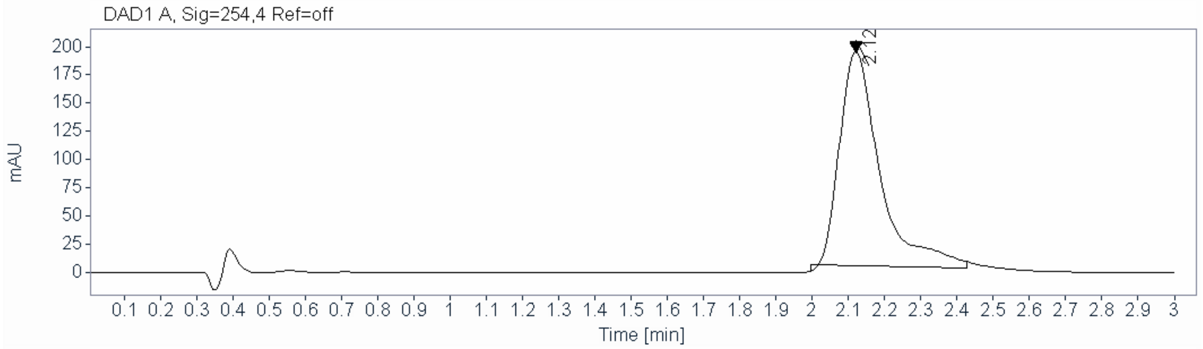

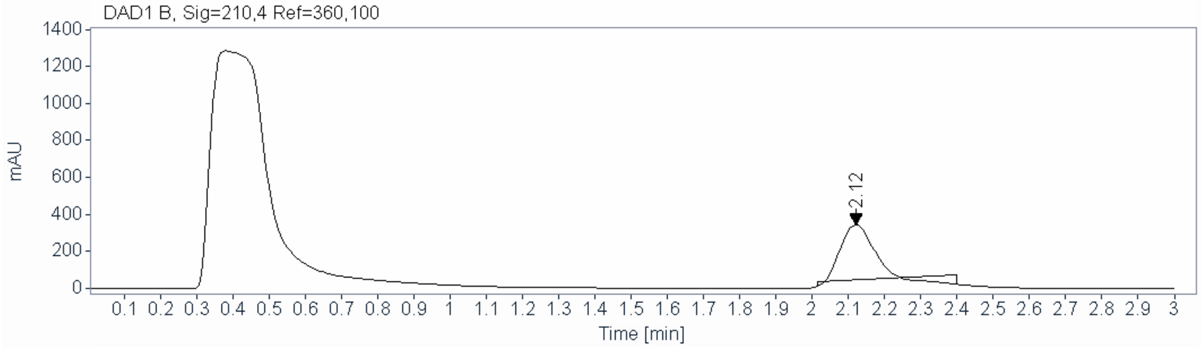

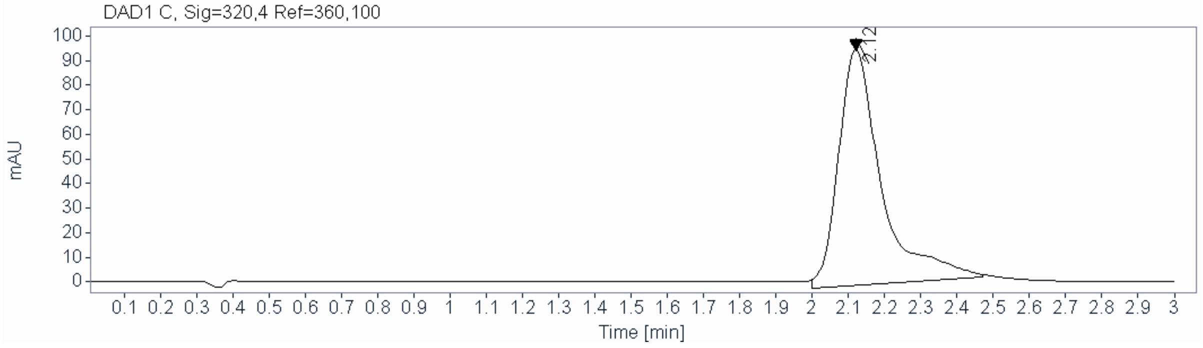

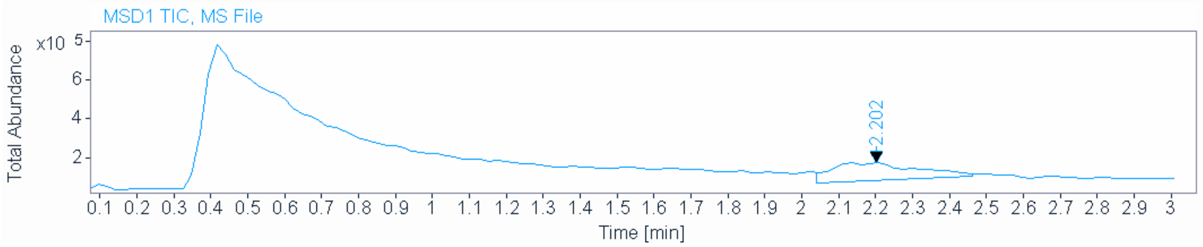

Agilent Technologies

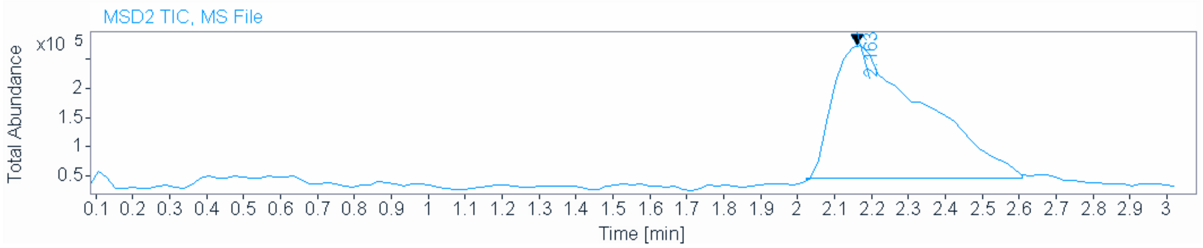

Signal: DAD1 A, Sig=254,4 Ref=off

RT [min]	Type	Width [min]	Area	Height	Area%	Name
2.120	MM	0.1292	1464.5482	188.9402	100.0000	
		Sum	1464.5482			

Signal: DAD1 B, Sig=210,4 Ref=360,100

RT [min]	Type	Width [min]	Area	Height	Area%	Name
2.120	MM	0.1037	1856.5771	298.3882	100.0000	
		Sum	1856.5771			

Signal: DAD1 C, Sig=320,4 Ref=360,100

RT [min]	Type	Width [min]	Area	Height	Area%	Name
2.120	MM	0.1375	790.4172	95.7921	100.0000	
		Sum	790.4172			

Signal: MSD1 TIC, MS File

RT [min]	Type	Width [min]	Area	Height	Area%	Name
2.202	MM	0.2663	1507900.625<br>0	94369.3047	100.0000	
		Sum	1507900.625			

Signal: MSD2 TIC, MS File

RT [min]	Type	Width [min]	Area	Height	Area%	Name
2.163	MM	0.2837	3869365.500<br>0	227335.6406	100.0000	
		Sum	3869365.500			

Agilent Technologies

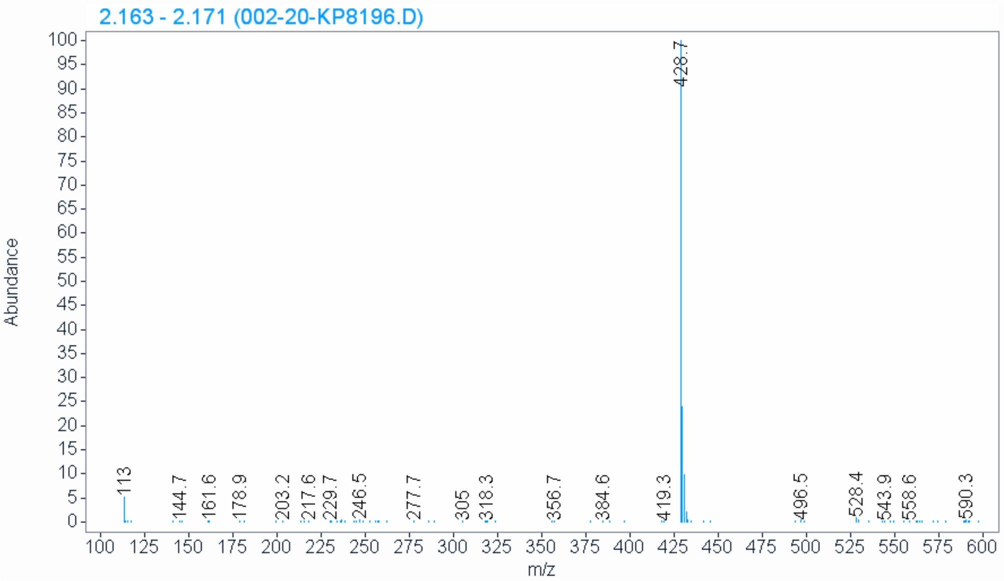

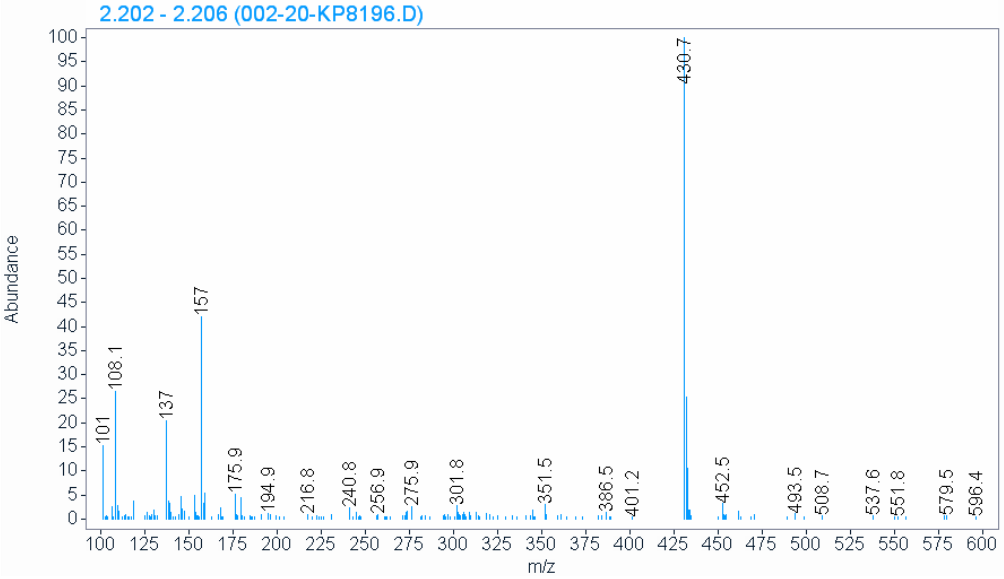

Compound Name: (Z)-*N*-(5-(cyclohex-3-en-1-ylmethylene)-4-oxo-4,5-dihydrothiazol-2-yl)naphthalene-1-sulfonamide

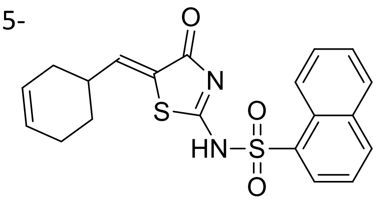

Chemical Formula: $C_{20}H_{18}N_2O_3S_2$
Exact Mass: 398.08
Molecular Weight: 398.50

Compound Code: 39 (KP6087)

Obtained Weight & Yield: 56 mg (28%)

Purity (by LCMS and ^{1}H NMR): > 95% (by ^{1}H-NMR and LCMS)

Appearance: Pale pink solid

Solubility: DMSO, slightly soluble in acetone

Melting Point: > 202 °C (dec.)

TLC Rf (and conditions): 0.58 (10% MeOH in DCM)

IR Analysis (including assignment): IR (neat): ν_{max} = 3137 (N-H), 3025 (Aromatic C-H), 2901 (C-H), 1711 (C=O), 1635 (C=C alkene), 1543 (C-C Aromatic), 1342 (sulfonamide), 1132 (C-N) cm^{-1}

^{1}H NMR Analysis: ^{1}H NMR (400 MHz, DMSO) δ 12.93 (br, s, 1H, NH), 8.58 (d, *J* = 8.6 Hz, 1H), 8.30 – 8.25 (m, 2H), 8.11 (d, *J* = 8.0 Hz, 1H), 7.78 – 7.66 (m, 3H), 6.84 (d, *J* = 9.7 Hz, 1H), 5.74 – 5.66 (m, 2H), 2.50 – 2.43 (m, 1H, partially hidden under DMSO solvent peak), 2.17 – 1.96 (m, 4H), 1.77 – 1.74 (m, 1H), 1.59 – 1.53 (m, 1H) ppm.
Ethanol impurity at 1.06 ppm (0.92%). Starting material at 4.04 ppm (3.70%)

^{13}C NMR Analysis: ^{13}C NMR (101 MHz, DMSO) δ 165.7, 165.5, 142.7, 135.3, 134.6, 133.8, 129.0, 128.2, 128.1, 127.6, 127.1, 126.8, 124.9, 124.7, 124.6, 124.0, 36.5, 29.0, 26.4, 23.3 ppm.

MS Analysis (low res): LRMS (ESI+) *m/z*: 399 (*M*+H, $C_{20}H_{19}N_2O_3S_2$, 100%); (ESI-) *m/z*: 397 (*M*-H, $C_{20}H_{17}$-$N_2O_3S_2$, 100%).

HPLC method details: Column: Zorbax SB-C18 Rapid Resolution HT 2.1x50mm 1.8-Micron; Method; LCMS ISOCRATIC 60%B 0.4MLMIN-1.M filename: KP6087; Peak retention time: 2.10 mins; Area (%): 95

Procedure: To a microwave vial was added the *N*-(4-oxo-4,5-dihydrothiazol-2-yl)naphthalene-1-sulfonamide (150 mg, 0.49 mmol), 1,2,3,6-tetrahydrobenzaldehyde (0.06 mL, 0.54 mmol, 1.1 eq), ethanol (3 mL) and the benzoic acid/piperidine catalyst (3 drops). The reaction mixture was treated with microwave irradiation (120°C, 20 min). After cooling, no precipitate was observed. Solvent was removed and the residue dissolved in ethyl acetate (3 mL). The dropwise addition of hexane resulted in formation of a precipitate which was collected after cooling by vacuum filtration, and washed with cold hexane to give a pale pink solid (56 mg, 28%).

Analyst analyst1

Date Thursday, 4 July 2019 12:47 PM

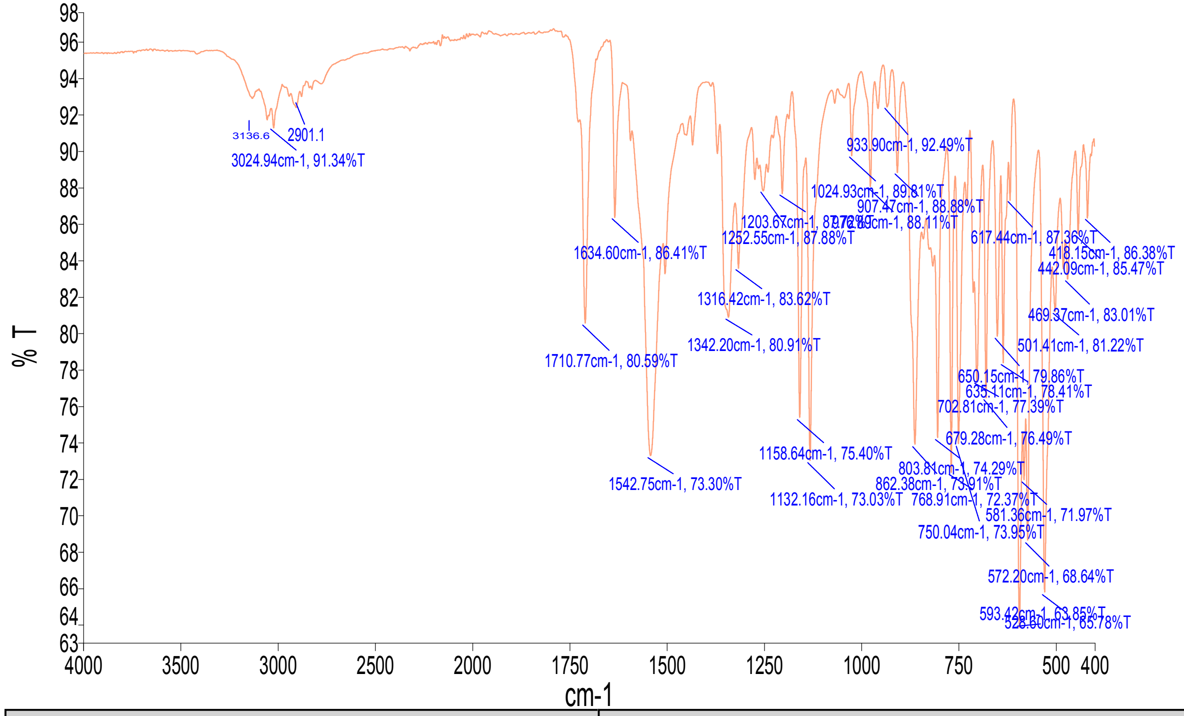

Sample Name	Description	Quality Checks
KP6087	Sample 015 By Analyst1 Date Thursday, July 04 2019	The Quality Checks give rise to a Weak Bands warning for the sample.

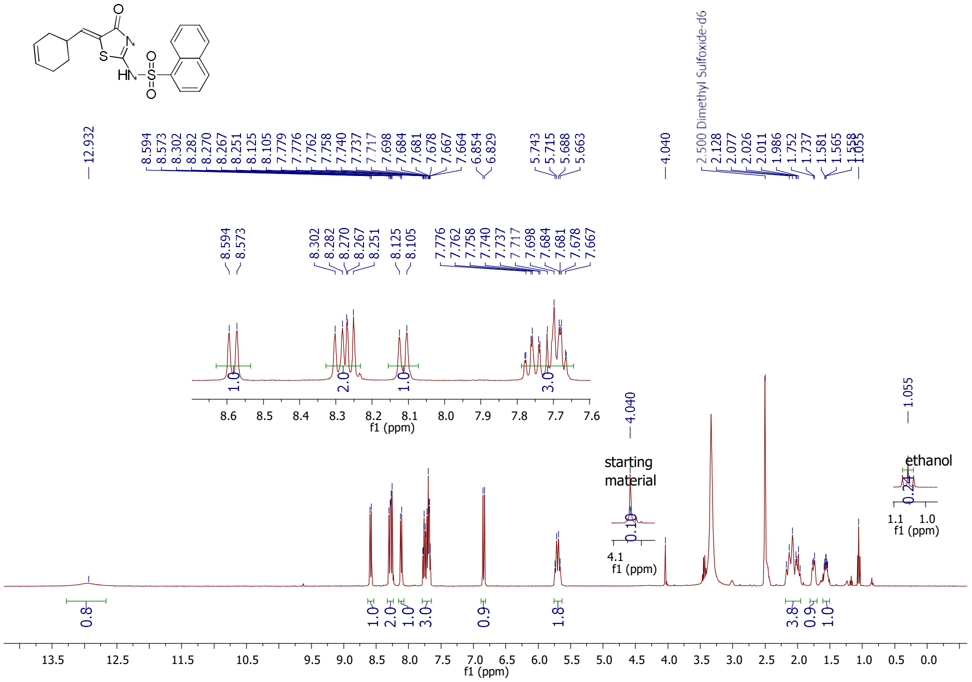
starting material
ethanol
f1 (ppm)
2.500 Dimethyl Sulfoxide-d6

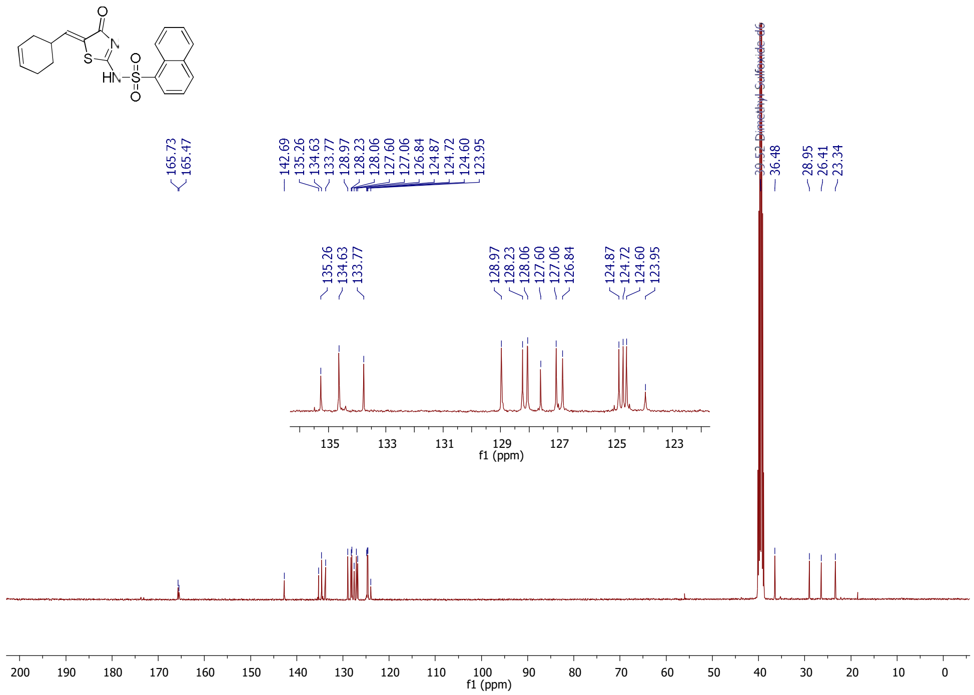
f1 (ppm)
39.52 Dimethyl Sulfoxide-d6

LCMS Report

Agilent Technologies

**Data file:**	D:\Chem32\1\Data\KP\KP60788587 2019-08-14 14-27-41\004-53-KP6087.D
**Sample name:**	KP6087
**Description:**	

**Sample amount:**	0.000	**Sample type:**	Sample
**Instrument:**	LCMS	**Location:**	53
**Injection date:**	8/14/2019 2:52:01 PM	**Injection:**	1 of 1
**Acq. method:**	LCMS ISOCRATIC 60% B 0.4MLMIN-1.M	**Injection volume:**	2.000
**Analysis method:**	LCMS ISOCRATIC 60%B 0.4MLMIN-1.M	**Acq. operator:**	SYSTEM
**Last changed:**	5/8/2019 8:55:04 AM		

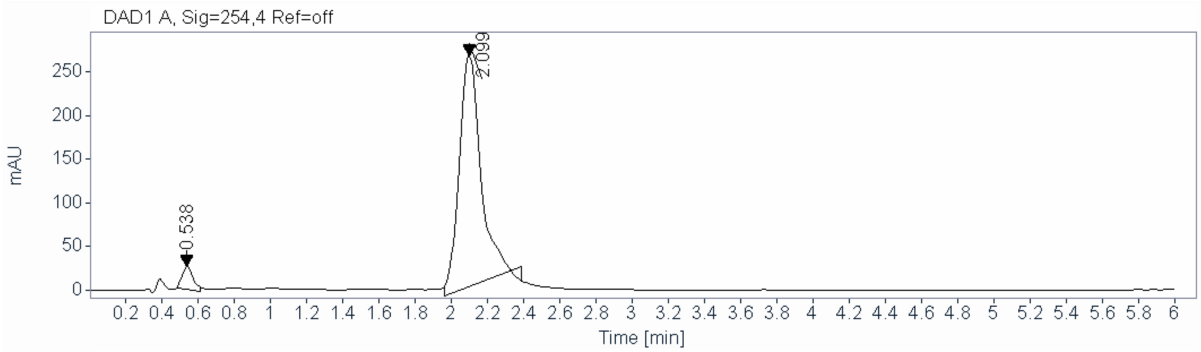

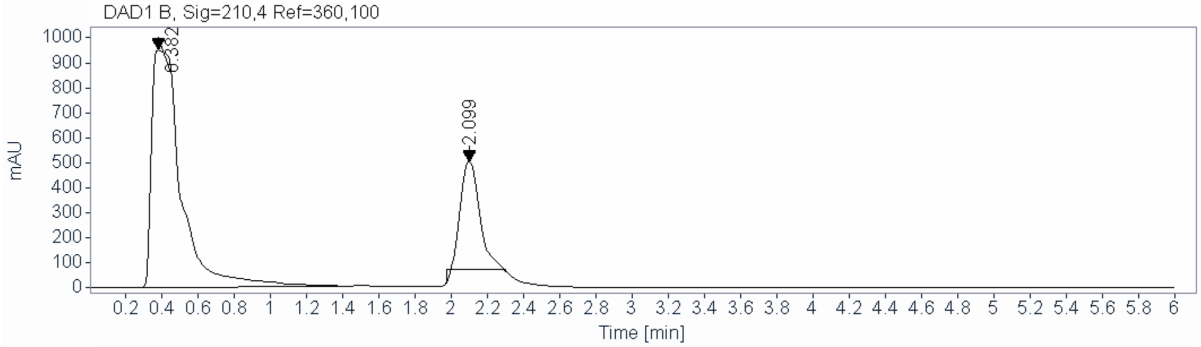

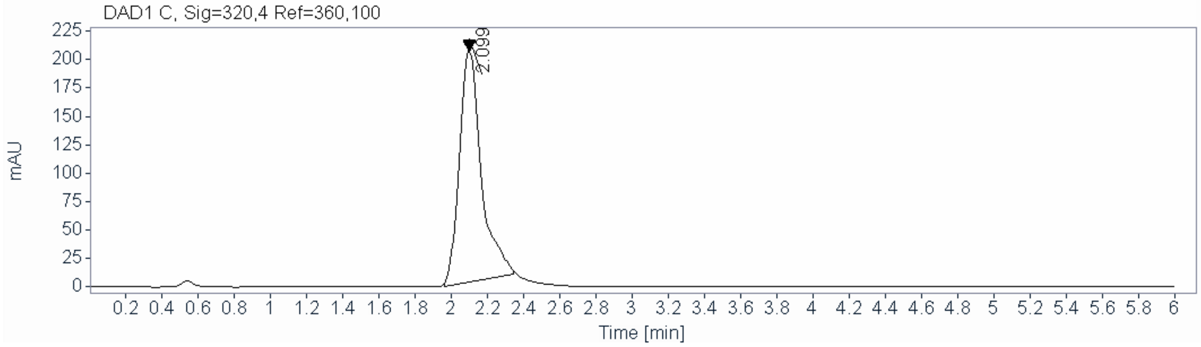

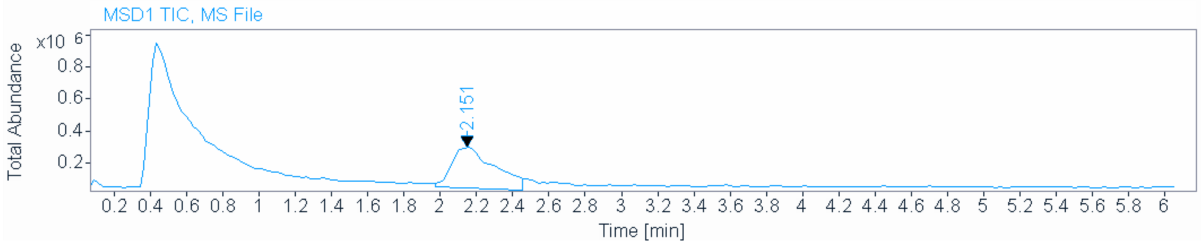

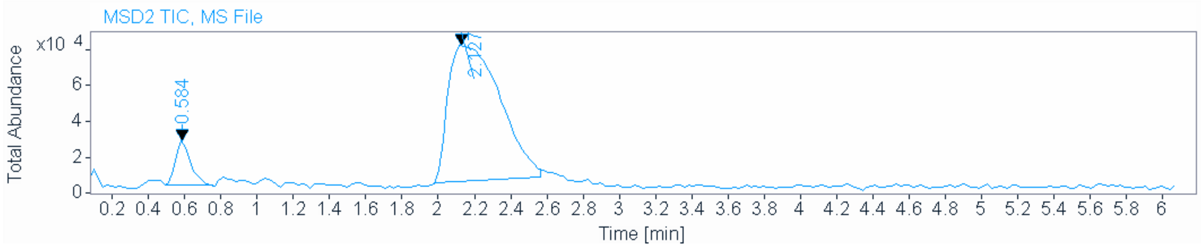

Signal: DAD1 A, Sig=254,4 Ref=off

RT [min]	Type	Width [min]	Area	Height	Area%	Name
0.538	MM	0.0718	113.1357	26.2644	4.8816	
2.099	MM	0.1381	2204.4382	265.9914	95.1184	
		Sum	2317.5739			

Signal: DAD1 B, Sig=210,4 Ref=360,100

RT [min]	Type	Width [min]	Area	Height	Area%	Name
0.382	BB	0.1520	10903.7197	949.5358	77.0762	
2.099	MM	0.1242	3242.9504	435.0414	22.9238	
		Sum	14146.6702			

Signal: DAD1 C, Sig=320,4 Ref=360,100

RT [min]	Type	Width [min]	Area	Height	Area%	Name
2.099	MM	0.1409	1726.7837	204.3083	100.0000	
		Sum	1726.7837			

Signal: MSD1 TIC, MS File

RT [min]	Type	Width [min]	Area	Height	Area%	Name
2.151	MM	0.2773	4288459.500<br>0	257785.2813	100.0000	
		Sum	4288459.500			

Signal: MSD2 TIC, MS File

RT [min]	Type	Width [min]	Area	Height	Area%	Name
0.584	BB	0.0873	141867.2969	24811.4219	8.6506	
2.127	MM	0.3293	1498107.625<br>0	75834.1250	91.3494	
		Sum	1639974.921			

Agilent Technologies

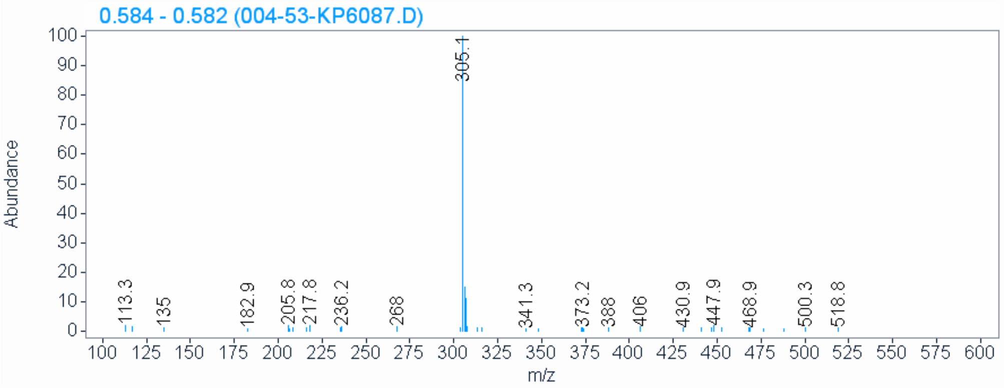

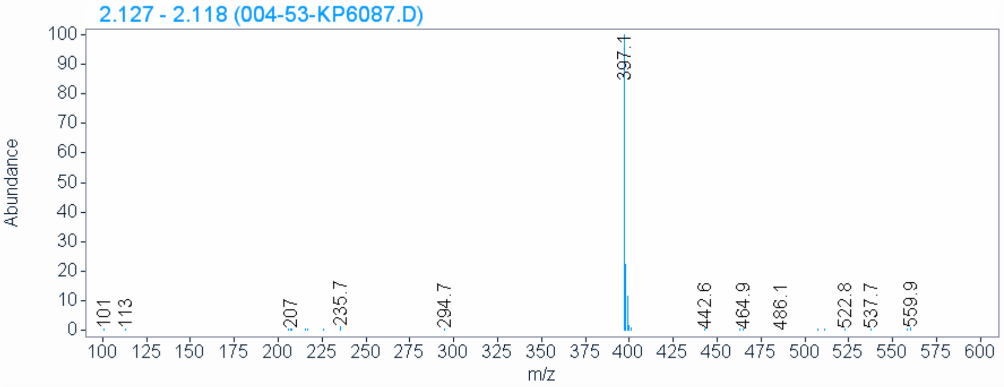

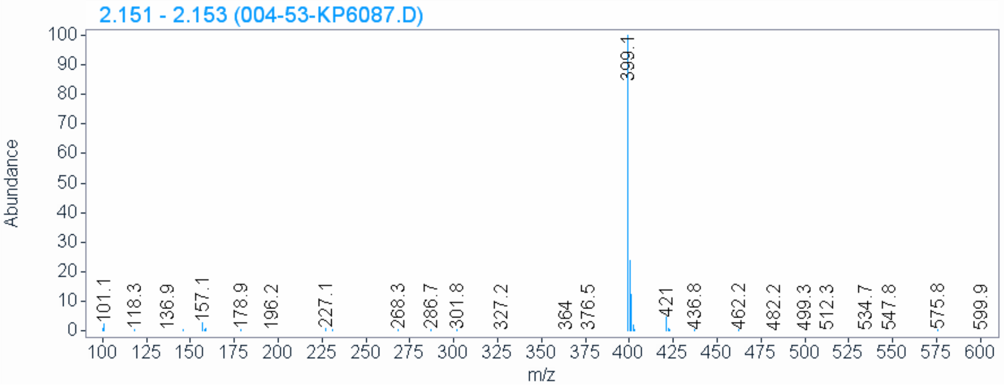

Compound Name: (*Z*)-*N*-(5-(cyclohexylmethylene)-4-oxo-4,5-dihydrothiazol-2-yl)naphthalene-1-sulfonamide

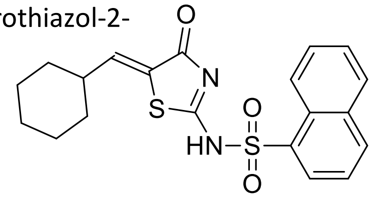

Chemical Formula: $C_{20}H_{20}N_2O_3S_2$
Exact Mass: 400.09
Molecular Weight: 400.51

Compound Code: 40 (KP9025)

Obtained Weight & Yield: 64 mg (33%)

Purity (by LCMS and ^{1}H NMR): > 99% by ^{1}H-NMR and LCMS

Appearance: white solid

Solubility: DMSO, slightly soluble in acetone and methanol.

Melting Point: > 221 °C (dec.)

TLC Rf (and conditions): N/A

IR Analysis (including assignment): IR (neat): ν_{max} = 3125 (N-H), 3060, 2997 (C-H aromatic), 2929, 2852 (C-H alkyl), 1711 (C=O), 1544 (C-C aromatic), 1344 (sulfonamide), 1134 (C-N) cm^{-1}

^{1}H NMR Analysis: ^{1}H NMR (600 MHz, DMSO) δ 12.96 (s, 1H, br, NH), 8.57 (d, *J* = 8.6 Hz, 1H), 8.28 (dd, *J* = 24.7, 7.7 Hz, 2H), 8.12 (d, *J* = 8.1 Hz, 1H), 7.77 – 7.75 (m, 1H), 7.70 (dd, *J* = 16.1, 8.1 Hz, 2H), 6.78 (d, *J* = 9.7 Hz, 1H), 2.23 (q, *J* = 10.2 Hz, 1H), 1.72 – 1.68 (m, 4H), 1.65 – 1.63 (m, 1H), 1.36 – 1.26 (m, 4H), 1.24 – 1.17 (m, 1H) ppm.
Ethanol at 1.05 ppm (0.72%)

^{13}C NMR Analysis: ^{13}C DEPTQ (151 MHz, DMSO) δ 165.6, 165.4, 143.5, 135.3, 134.7, 133.8, 129.0, 128.3, 128.0, 127.6, 127.1, 124.9, 124.6, 123.2, 40.6, 30.5 (2C), 25.1, 24.6 (2C) ppm.

MS Analysis (low res): LRMS (ESI-) *m/z*: 399 (*M*-H, $C_{20}H_{19}N_2O_3S_2$, 100); (ESI+) *m/z*: 401 (*M*+H, $C_{20}H_{21}N_2O_3S_2$, 100)

MS Analysis (high res): Exact mass calculated for $C_{20}H_{19}N_2O_3S_2$ [*M*-H]$^-$, 399.0800. Found 399.0842.

HPLC method details: Column: Zorbax SB-C18 Rapid Resolution HT 2.1x50mm 1.8-Micron; Method: LCMS ISOCRATIC 80%B_3 MINS.M filename: KP9025; Peak retention time: 1.01 mins; Area (%): 100.

Procedure: To a 10 mL microwave vial was added *N*-(4-oxo-4,5-dihydrothiazol-2-yl)naphthalene-1-sulfonamide (154 mg, 0.49 mmol), cyclohexane carboxaldehyde (0.1 mL, 0.735 mmol, 1.1 eq), ethanol (3 mL) and a catalytic amount of the benzoic acid/piperidine catalyst (approximately 5 drops). The suspension was heated by microwave irradiation (120 °C, 200 W) for 1 h. A precipitate formed upon cooling overnight. The solid was collected by vacuum filtration to give the desired product as a white yellow solid (64 mg, 33%).

Other analyses, reference papers, previously obtained data, comments, etc:

PerkinElmer Spectrum Version 10.4.00
Thursday, 26 November 2020 11:21 AM

Analyst research
Date Thursday, 26 November 2020 11:21 AM

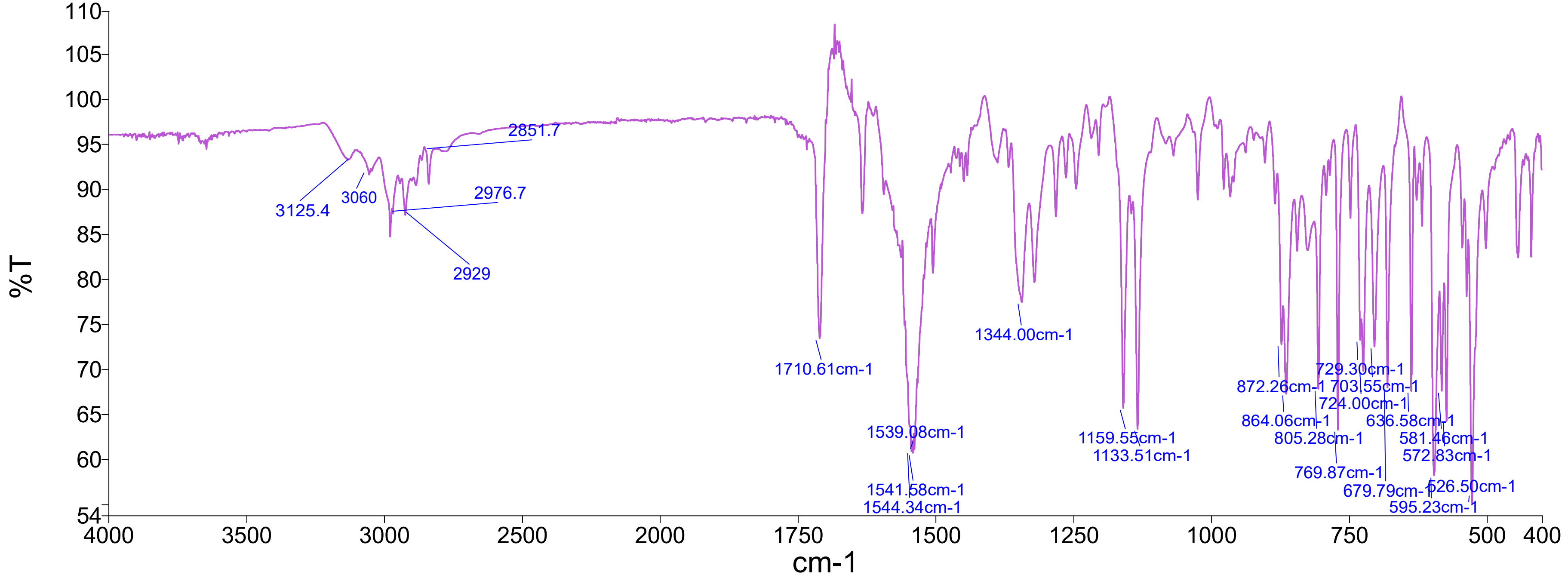

Sample Name	Description	Quality Checks
kp9025	Sample 174 By research Date Thursday, November 26 2020	The Quality Checks give rise to a Baseline High warning for the sample.

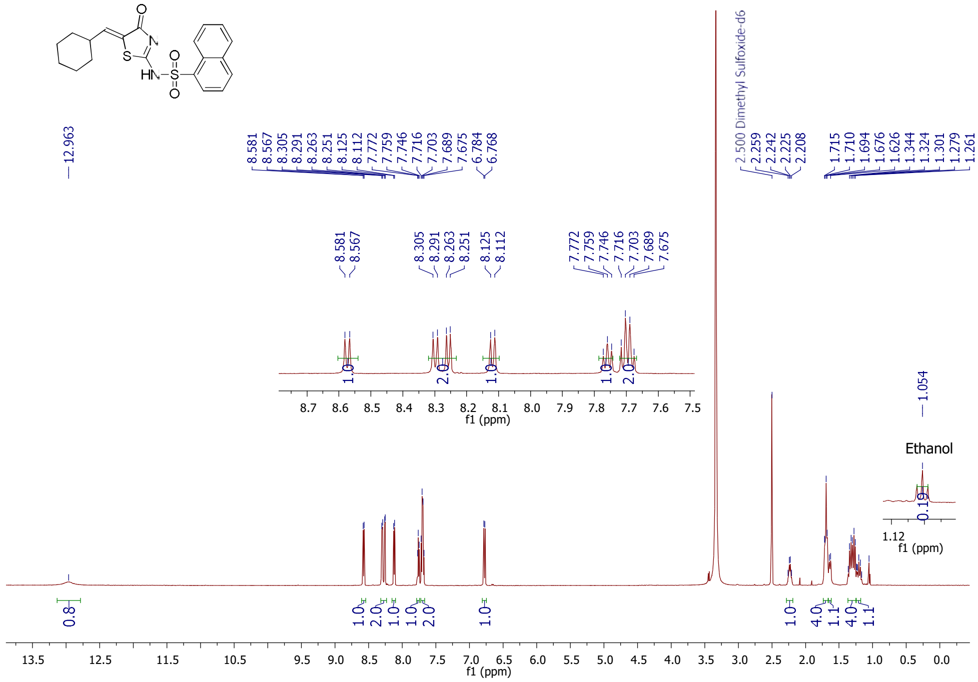

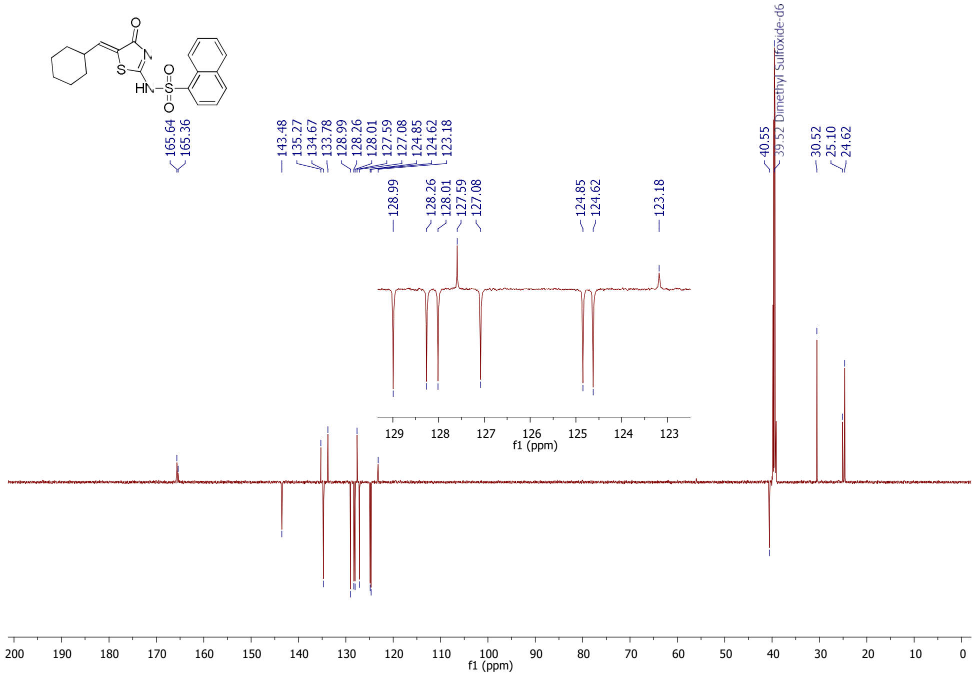

LCMS Report

Agilent Technologies

**Data file:**	D:\Chem32\1\Data\KP\KP_DS_NOV4 2020-11-02 13-34-37\003-19-KP9025.D		
**Sample name:**	KP9025		
**Description:**			
**Sample amount:**	0.000	**Sample type:**	Sample
**Instrument:**	LCMS	**Location:**	19
**Injection date:**	11/2/2020 1:45:31 PM	**Injection:**	1 of 1
**Acq. method:**	LCMS ISOCRATIC 80% B_3 MINS.M	**Injection volume:**	2.000
**Analysis method:**	LCMS ISOCRATIC 80%B_3 MINS.M	**Acq. operator:**	SYSTEM
**Last changed:**	10/8/2020 2:52:31 PM		

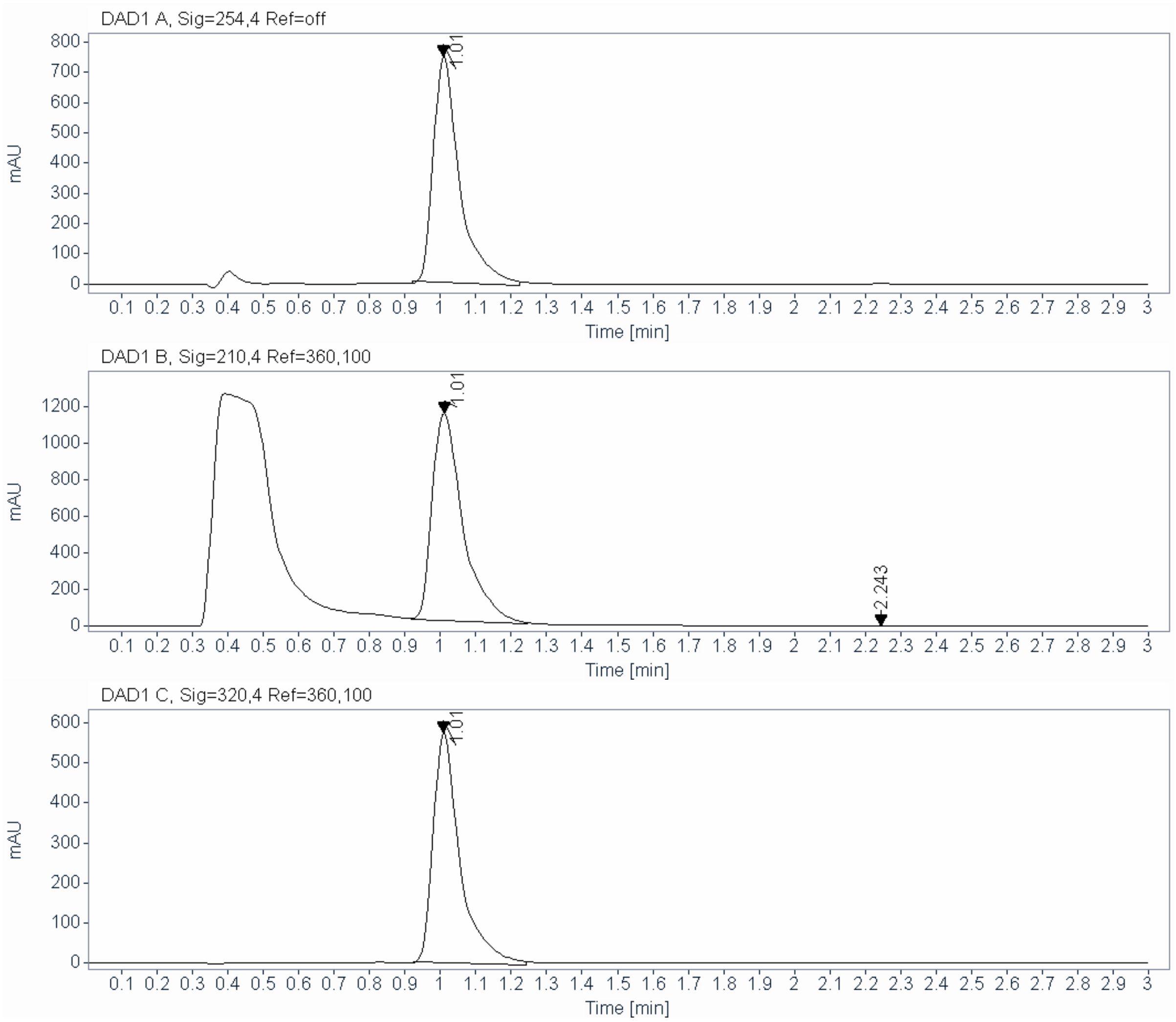

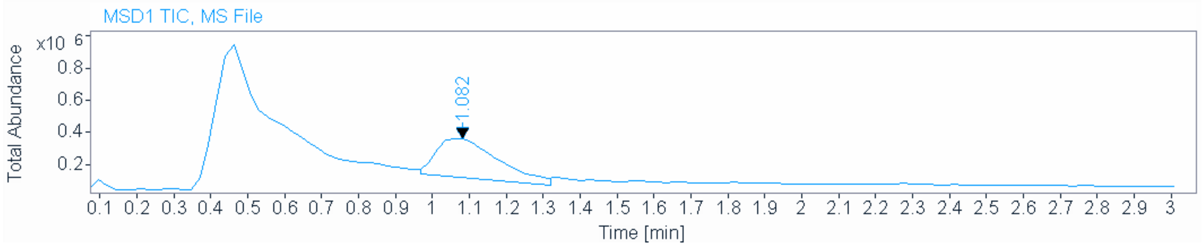

LCMS Report

Agilent Technologies

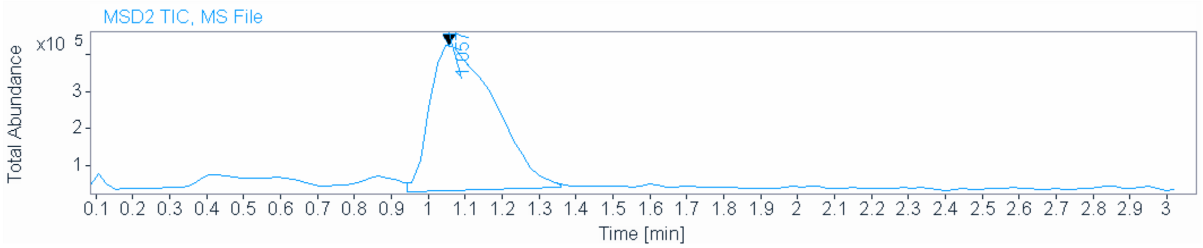

Signal: DAD1 A, Sig=254,4 Ref=off

RT [min]	Type	Width [min]	Area	Height	Area%	Name
1.010	MM	0.0860	3874.4460	750.7292	100.0000	
		Sum	3874.4460			

Signal: DAD1 B, Sig=210,4 Ref=360,100

RT [min]	Type	Width [min]	Area	Height	Area%	Name
1.010	MM	0.1007	6882.9717	1139.0465	99.7888	
2.243	BB	0.1106	14.5643	1.9625	0.2112	
		Sum	6897.5360			

Signal: DAD1 C, Sig=320,4 Ref=360,100

RT [min]	Type	Width [min]	Area	Height	Area%	Name
1.010	MM	0.0876	3030.0793	576.2108	100.0000	
		Sum	3030.0793			

Signal: MSD1 TIC, MS File

RT [min]	Type	Width [min]	Area	Height	Area%	Name
1.082	MM	0.1994	2910843.2500	243258.1406	100.0000	
		Sum	2910843.250			

Signal: MSD2 TIC, MS File

RT [min]	Type	Width [min]	Area	Height	Area%	Name
1.057	MM	0.1984	4737728.0000	398077.6250	100.0000	
		Sum	4737728.000			

1.057 - 1.047 (003-19-KP9025.D)

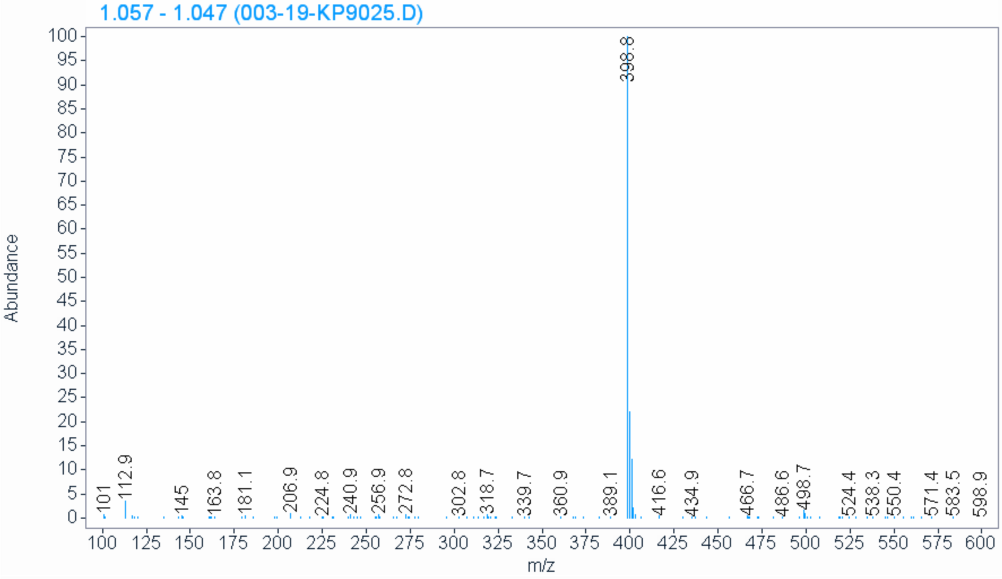

1.082 - 1.082 (003-19-KP9025.D)

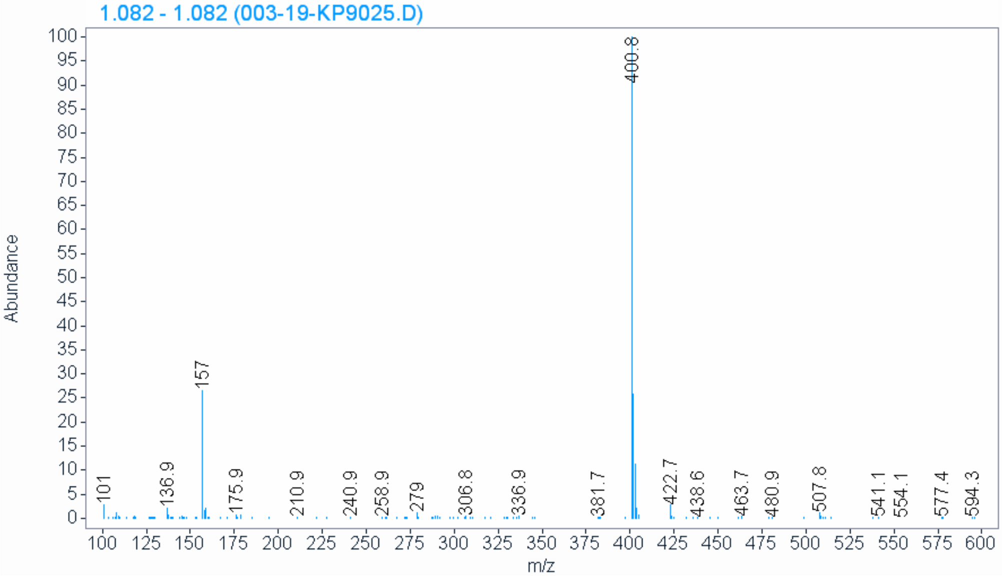

Compound Name: (Z)-*N*-(5-(cyclopentylmethylene)-4-oxo-4,5-dihydrothiazol-2-yl)naphthalene-1-sulfonamide

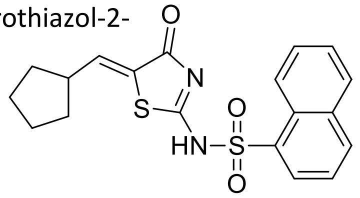

Chemical Formula: $C_{19}H_{18}N_2O_3S_2$
Exact Mass: 386.08
Molecular Weight: 386.49

Compound Code: 41 (KP6098)

Obtained Weight & Yield: 85 mg, 63%

Purity (by LCMS and ^{1}H NMR): >99% by ^{1}H-NMR and LCMS

Appearance: white solid

Solubility: DMSO, slightly soluble in methanol and acetone

Melting Point: > 218 °C (dec.)

TLC Rf (and conditions): N/A

IR Analysis (including assignment): IR (neat): v_{max} = 3146 (N-H), 3049 (aromatic C-H), 2945 (C-H), 1710 (C=O), 1539 (aromatic C-C), 1337 (sulfonamide), 1158 (C-N) cm^{-1}

^{1}H NMR Analysis: ^{1}H NMR (400 MHz, DMSO) δ 12.94 (s, 1H), 8.58 (d, *J* = 8.6 Hz, 1H), 8.31 – 8.24 (m, 2H), 8.11 (d, *J* = 8.0 Hz, 1H), 7.76 (ddd, *J* = 8.5, 6.9, 1.3 Hz, 1H), 7.69 (dt, *J* = 6.7, 4.2 Hz, 2H), 6.88 (d, *J* = 9.8 Hz, 1H), 2.67 – 2.56 (m, 1H), 1.92 – 1.88 (m, 2H), 1.70 – 1.59 (m, 4H), 1.48 – 1.39 (m, 2H) ppm.
Ethanol at 1.05 ppm (0.20%)

^{13}C NMR Analysis: ^{13}C NMR (101 MHz, DMSO) δ 165.7, 165.2, 144.0, 135.3, 134.7, 133.8, 129.0, 128.3, 128.1, 127.6, 127.1, 124.9, 124.6, 123.5, 42.3, 32.1 (2C), 25.1 (2C) ppm.
2C determined by 2D NMR.

MS Analysis (low res): LRMS (ESI-) *m/z* (%): 385 (*M*-H, $C_{19}H_{17}N_2O_3S_2$, 100%); (ESI+) *m/z*: 387 (*M*+H, $C_{19}H_{19}N_2O_3S_2$, 100%)

MS Analysis (high res): Exact mass calculated for $C_{19}H_{17}N_2O_3S_2$ [*M*-H]$^-$, 385.0700. Found 385.0685.

HPLC method details: Column: Zorbax SB-C18 Rapid Resolution HT 2.1x50mm 1.8-Micron; Method: LCMS ISOCRATIC 60%B 0.4MLMIN-1.M filename: KP6098; Peak retention time: 1.962 mins; Area (%): 99

Procedure: To a 10 mL microwave vial was added the *N*-(4-oxo-4,5-dihydrothiazol-2-yl)naphthalene-1-sulfonamide (108 mg, 0.35 mmol), cyclopentanecarbaldehyde (0.04 mL, 0.39 mmol, 1.1 eq), ethanol (3 mL) and a catalytic amount of the benzoic acid/piperidine catalyst (approximately 10 drops). The suspension was heated using microwave irradiation (200 W, 120 °C) for 30 min then placed in the freezer overnight. The precipitate was collected by vacuum filtration and washed with cold ethanol and cold ether to give the desired product (85 mg, 63%).

Other analyses, reference papers, previously obtained data, comments, etc:

PerkinElmer Spectrum Version 10.4.00
Tuesday, 23 July 2019 11:34 AM

Analyst research
Date Tuesday, 23 July 2019 11:34 AM

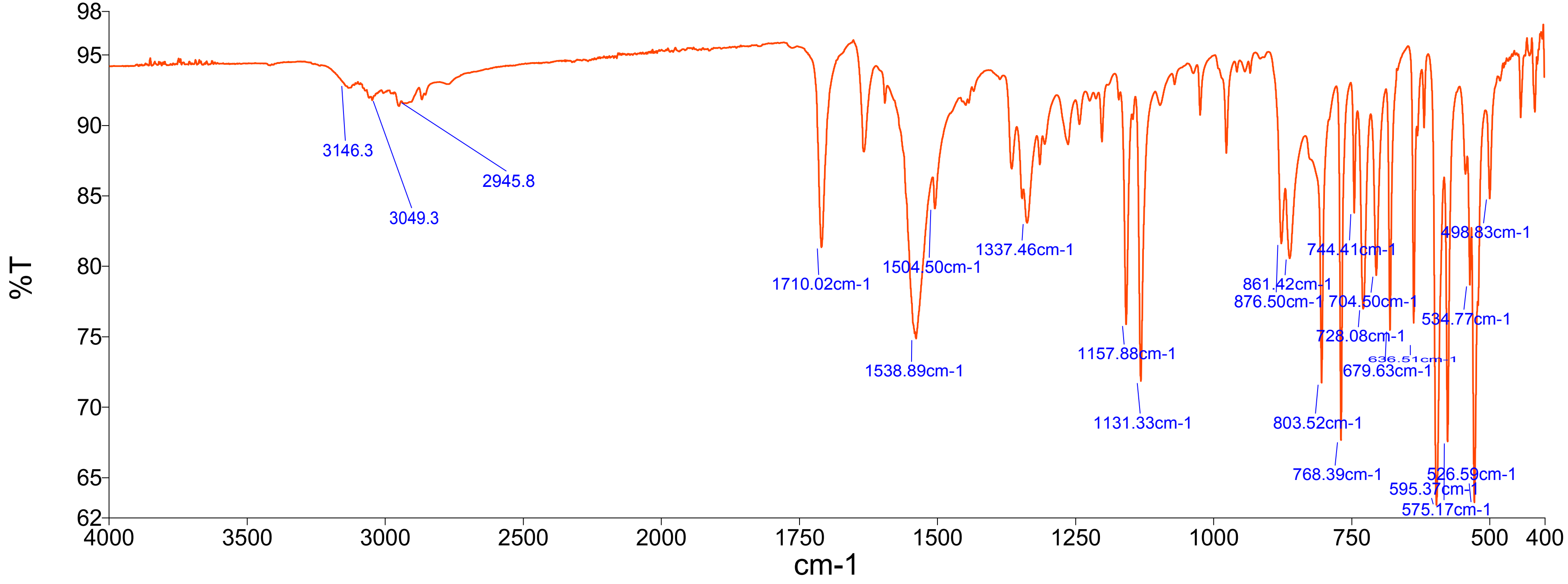

Sample Name	Description	Quality Checks
KP6098	Sample 233 By research Date Tuesday, July 23 2019	The Quality Checks do not report any warnings for the sample.

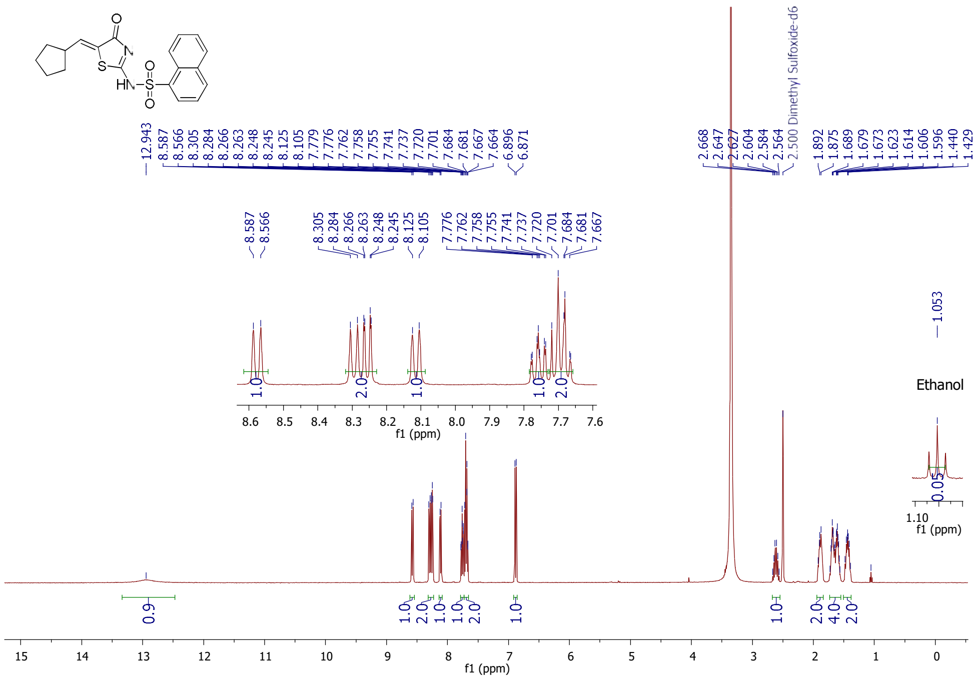
O
N
S
HN
S
O
O
12.943
8.587
8.566
8.305
8.284
8.266
8.263
8.248
8.245
8.125
8.105
7.779
7.776
7.762
7.758
7.755
7.741
7.737
7.720
7.701
7.684
7.681
7.667
7.664
6.896
6.871
2.668
2.647
2.627
2.604
2.584
2.564
2.500 Dimethyl Sulfoxide-d6
1.892
1.875
1.689
1.679
1.673
1.623
1.614
1.606
1.596
1.440
1.429
1.053
Ethanol
f1 (ppm)

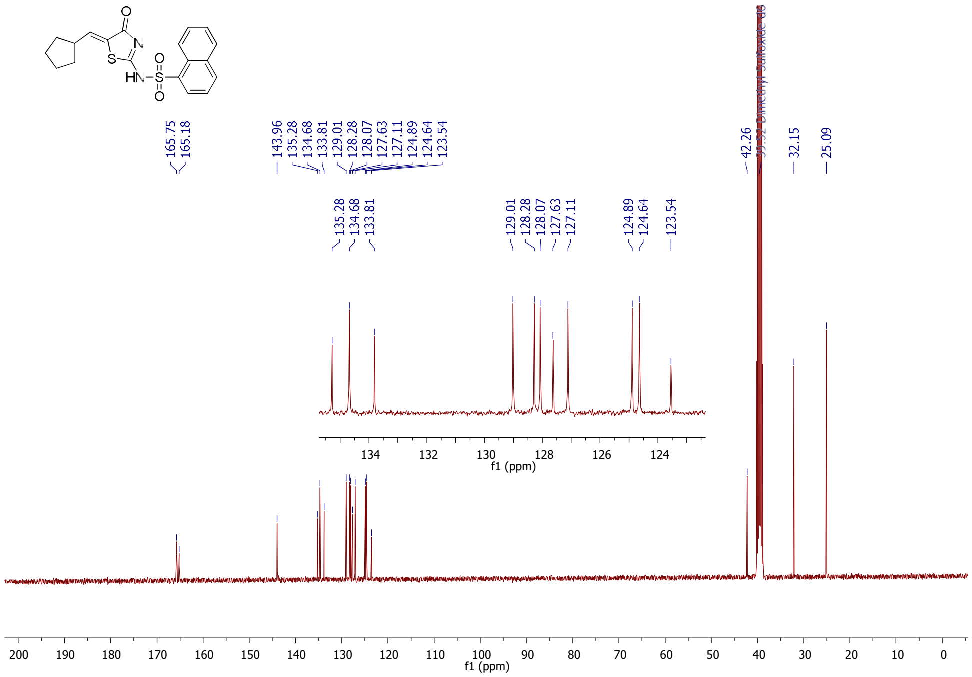
O
N
S
HN
S
O
O
165.75
165.18
143.96
135.28
134.68
133.81
129.01
128.28
128.07
127.63
127.11
124.89
124.64
123.54
42.26
32.15
25.09
f1 (ppm)

Agilent Technologies

**Data file:**	D:\Chem32\1\Data\KP\KP_DS_11NOV 2019-11-11 14-45-23\002-41-KP6098.D		
**Sample name:**	KP6098		
**Description:**			
**Sample amount:**	0.000	**Sample type:**	Sample
**Instrument:**	LCMS	**Location:**	41
**Injection date:**	11/11/2019 2:55:47 PM	**Injection:**	1 of 1
**Acq. method:**	LCMS ISOCRATIC 60% B 0.4MLMIN-1.M	**Injection volume:**	2.000
**Analysis method:**	LCMS ISOCRATIC 60%B 0.4MLMIN-1.M	**Acq. operator:**	SYSTEM
**Last changed:**	5/8/2019 8:55:04 AM		

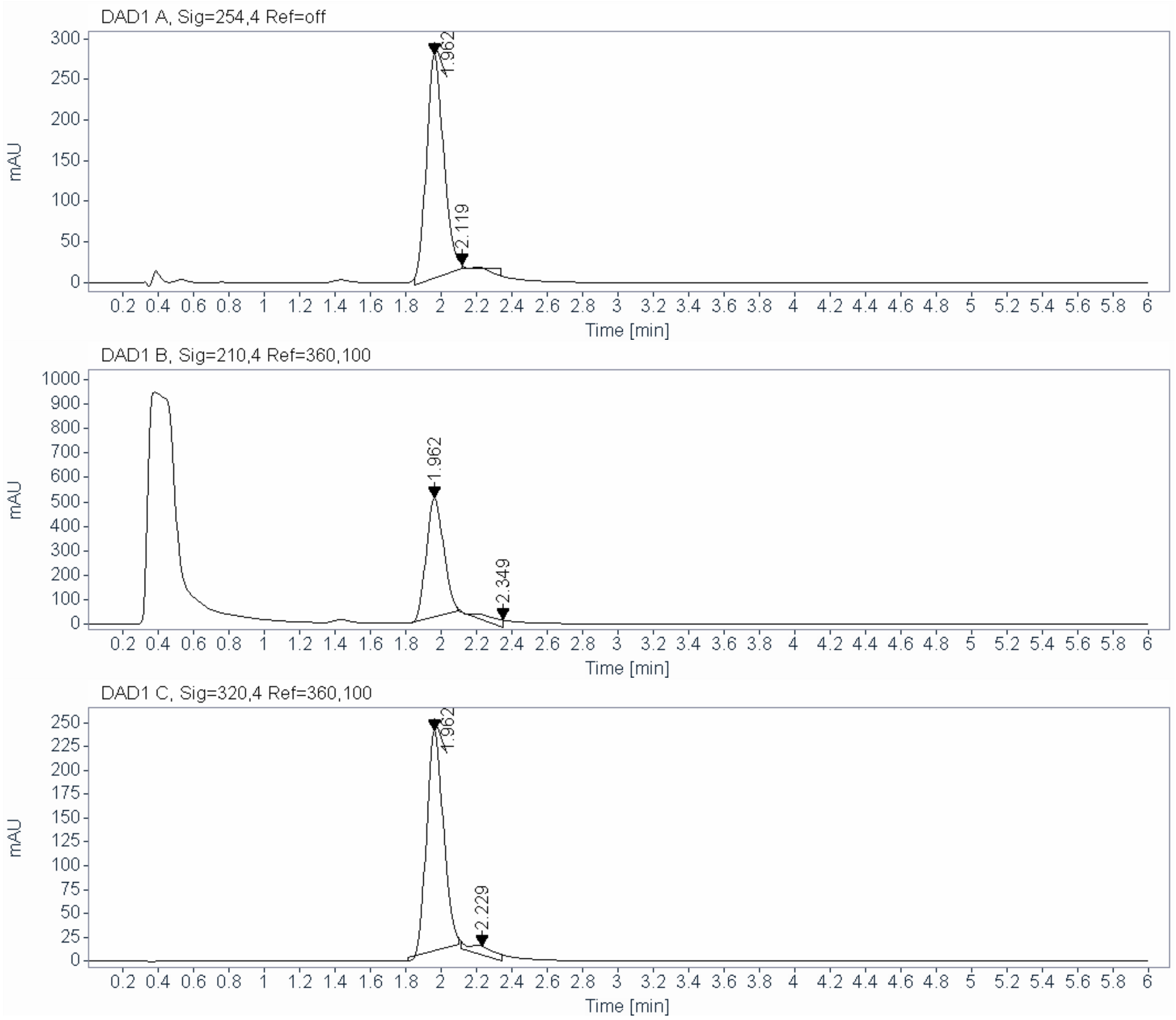

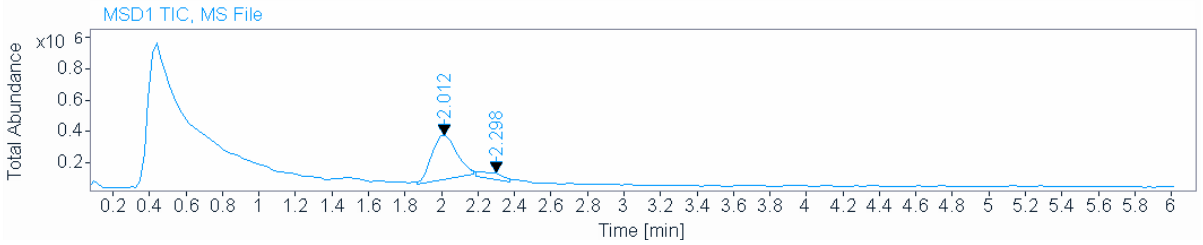

Agilent Technologies

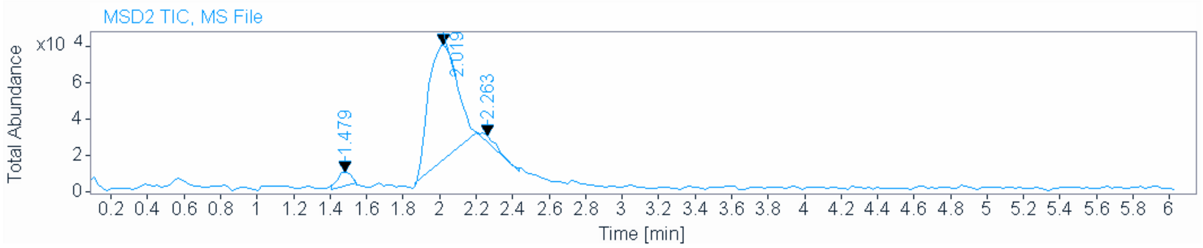

Signal: DAD1 A, Sig=254,4 Ref=off

RT [min]	Type	Width [min]	Area	Height	Area%	Name
1.962	MM	0.1136	1883.1329	276.2331	99.4580	
2.119	MM	0.0525	10.2619	4.5641	0.5420	
		Sum	1893.3949			

Signal: DAD1 B, Sig=210,4 Ref=360,100

RT [min]	Type	Width [min]	Area	Height	Area%	Name
1.962	MM	0.1138	3331.7026	487.9232	92.8434	
2.349	MM	0.1395	256.8172	30.6780	7.1566	
		Sum	3588.5198			

Signal: DAD1 C, Sig=320,4 Ref=360,100

RT [min]	Type	Width [min]	Area	Height	Area%	Name
1.962	MM	0.1099	1527.3099	231.5853	93.6453	
2.229	MM	0.1838	103.6415	9.3959	6.3547	
		Sum	1630.9515			

Signal: MSD1 TIC, MS File

RT [min]	Type	Width [min]	Area	Height	Area%	Name
2.012	MM	0.1544	2702684.7500	291829.6250	88.4175	
2.298	MM	0.1487	354046.0938	39671.8398	11.5825	
		Sum	3056730.843			

Signal: MSD2 TIC, MS File

RT [min]	Type	Width [min]	Area	Height	Area%	Name
1.479	MM	0.0812	38942.7383	7991.4639	5.2245	
2.019	MM	0.1785	681195.1250	63620.9805	91.3879	
2.263	MM	0.1155	25251.2305	3643.9482	3.3877	
		Sum	745389.0938			

Agilent Technologies

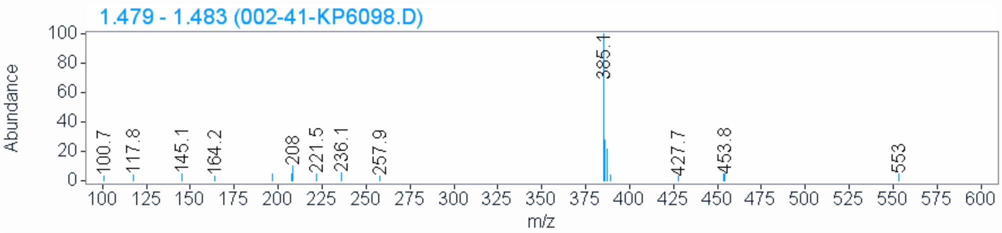

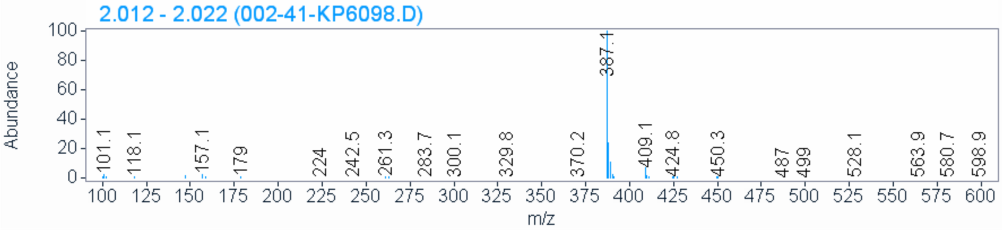

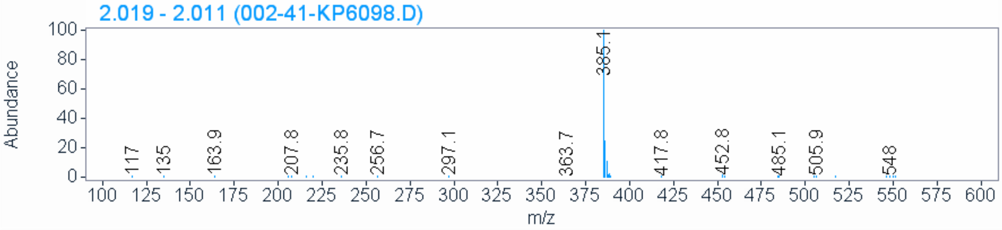

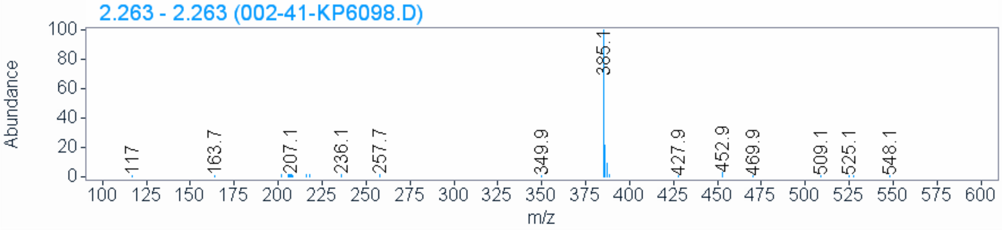

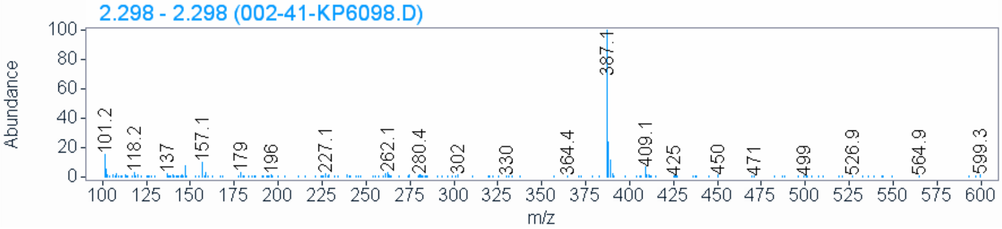

Compound Name: (*Z*)-*N*-(4-oxo-5-pentylidene-4,5-dihydrothiazol-2-yl)naphthalene-1-sulfonamide

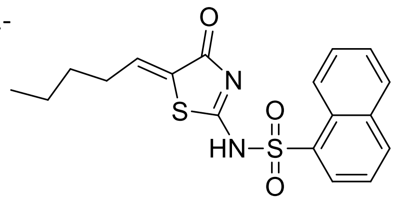

Chemical Formula: $C_{18}H_{18}N_2O_3S_2$
Exact Mass: 374.08
Molecular Weight: 374.48

Compound Code: 42 (KP6099)

Obtained Weight & Yield: 210 mg, 57%

Purity (by LCMS and ^{1}H NMR): 99% by ^{1}H-NMR and LCMS

Appearance: off white solid (chalk white)

Solubility: DMSO, soluble in methanol and acetone

Melting Point: > 145 °C (dec.)

TLC Rf (and conditions): N/A

IR Analysis (including assignment): IR (neat): ν_{max} = 3153 (N-H), 3062 (aromatic C-H), 2965, 2962 (C-H), 1715 (C=O), 1546 (aromatic C-C), 1350 (sulfonamide), 1131 (C-N) cm^{-1}

^{1}H NMR Analysis: ^{1}H NMR (400 MHz, DMSO) δ 12.95 (s, 1H), 8.58 (d, *J* = 8.6 Hz, 1H), 8.31 – 8.25 (m, 2H), 8.12 (d, *J* = 8.1 Hz, 1H), 7.78 – 7.74 (m, 1H), 7.69 (dt, *J* = 6.5, 4.2 Hz, 2H), 6.91 (t, *J* = 7.8 Hz, 1H), 2.27 (q, *J* = 7.4 Hz, 2H), 1.51 – 1.44 (m, 2H), 1.36 – 1.27 (m, 2H), 0.88 (t, *J* = 7.3 Hz, 3H) ppm.

^{13}C NMR Analysis: ^{13}C NMR (101 MHz, DMSO) δ 165.6, 164.9, 139.7, 135.2, 134.7, 133.8, 129.0, 128.3, 128.1, 127.6, 127.1, 124.9, 124.8, 124.6, 30.9, 29.4, 21.8, 13.6 ppm

MS Analysis (low res): LRMS (ESI-) *m/z* (%): 373 (*M*-H, $C_{18}H_{17}N_2O_3S_2$, 100%); (ESI+) *m/z*: 375 (*M*+H, $C_{18}H_{19}N_2O_3S_2$, 100%)

MS Analysis (high res): Exact mass calculated for $C_{18}H_{17}N_2O_3S_2$ [*M*-H]$^-$, 373.0700. Found 373.0683.

HPLC method details: Column: Zorbax SB-C18 Rapid Resolution HT 2.1x50mm 1.8-Micron; Method: LCMS ISOCRATIC 60%B 0.4MLMIN-1.M filename: KP6099; Peak retention time: 1.805 mins; Area (%): 99.

Procedure: To a 10 mL microwave vial was added the *N*-(4-oxo-4,5-dihydrothiazol-2-yl)naphthalene-1-sulfonamide (300 mg, 0.98 mmol), valaraldehyde (0.12 mL, 1.0 mmol, 1.1 eq), ethanol (3 mL) and a catalytic amount of the benzoic acid/piperidine catalyst (approximately 10 drops). The suspension was heated using microwave irradiation (200 W, 120 °C) for 30 min then placed in the freezer over an extended period. The resulting precipitate was collected by vacuum filtration and washed with cold ethanol and cold ether to give the desired product (210 mg, 57%).

Other analyses, reference papers, previously obtained data, comments, etc:

PerkinElmer Spectrum Version 10.4.00
Tuesday, 23 July 2019 11:37 AM

Analyst research

Date Tuesday, 23 July 2019 11:37 AM

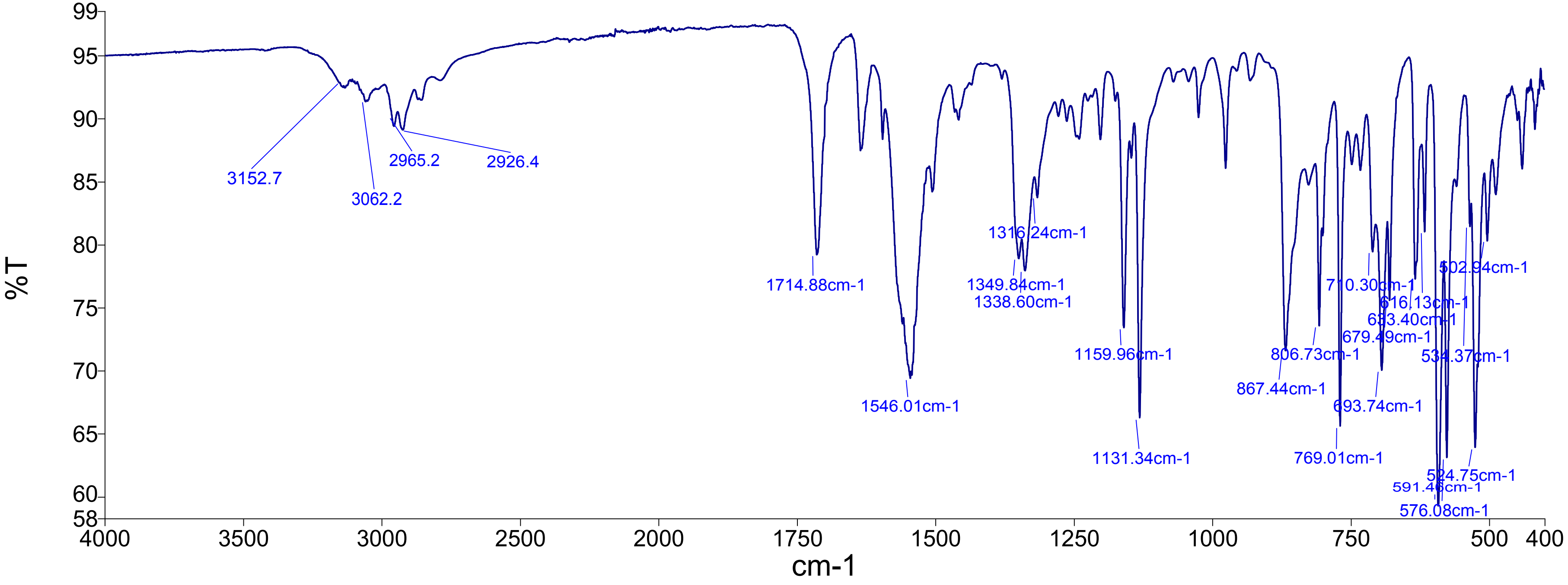

Sample Name	Description	Quality Checks
KP6099	Sample 234 By research Date Tuesday, July 23 2019	The Quality Checks do not report any warnings for the sample.

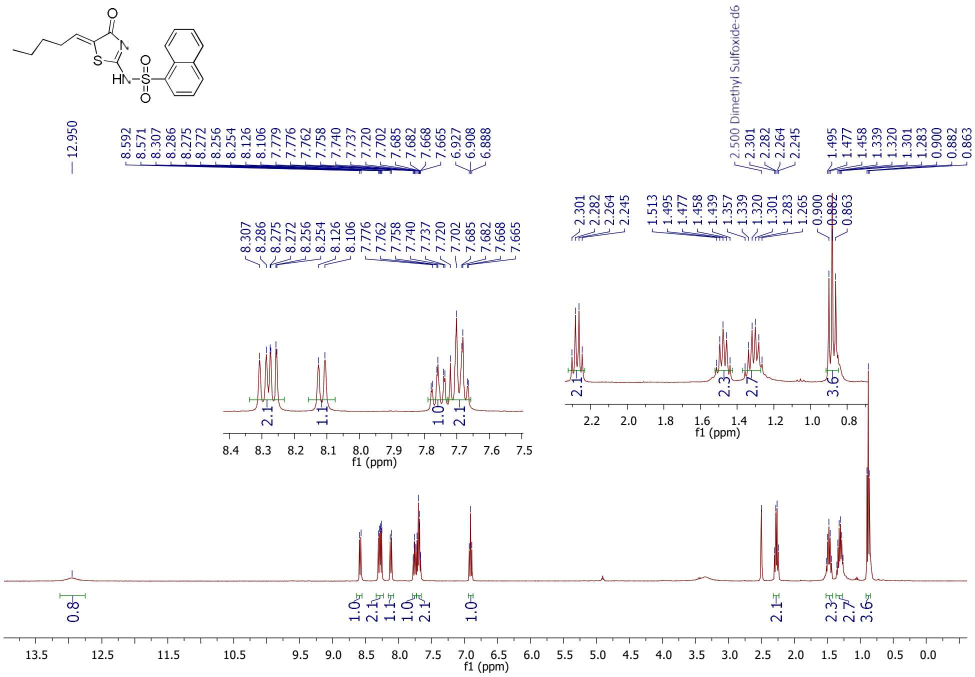
O
N
S
HN
S
O
O
12.950
8.592
8.571
8.307
8.286
8.275
8.272
8.256
8.254
8.126
8.106
7.779
7.776
7.762
7.758
7.740
7.737
7.720
7.702
7.685
7.682
7.668
7.665
6.927
6.908
6.888
2.500 Dimethyl Sulfoxide-d6
2.301
2.282
2.264
2.245
1.495
1.477
1.458
1.339
1.320
1.301
1.283
0.900
0.882
0.863
1.513
1.439
1.357
1.265
0.8
1.0
2.1
1.1
1.0
2.1
1.0
2.1
2.3
2.7
3.6
f1 (ppm)

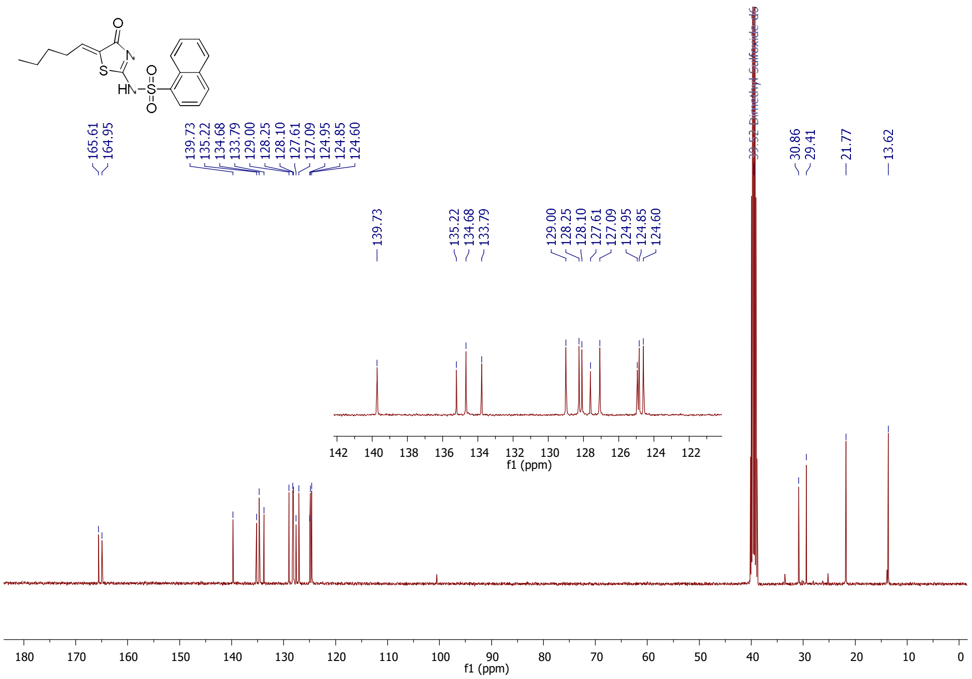
O
N
S
HN
S
O
O
165.61
164.95
139.73
135.22
134.68
133.79
129.00
128.25
128.10
127.61
127.09
124.95
124.85
124.60
39.52 Dimethyl Sulfoxide-d6
30.86
29.41
21.77
13.62
f1 (ppm)

LCMS Report

Agilent Technologies

Data file: D:\Chem32\1\Data\KP\KP_DS_11NOV 2019-11-11 14-45-23\003-42-KP6099.D
Sample name: KP6099
Description:
Sample amount: 0.000
Sample type: Sample

Instrument: LCMS
Location: 42
Injection date: 11/11/2019 3:03:26 PM
Injection: 1 of 1
Acq. method: LCMS ISOCRATIC 60% B 0.4MLMIN-1.M
Injection volume: 2.000
Analysis method: LCMS ISOCRATIC 60%B 0.4MLMIN-1.M
Acq. operator: SYSTEM
Last changed: 5/8/2019 8:55:04 AM

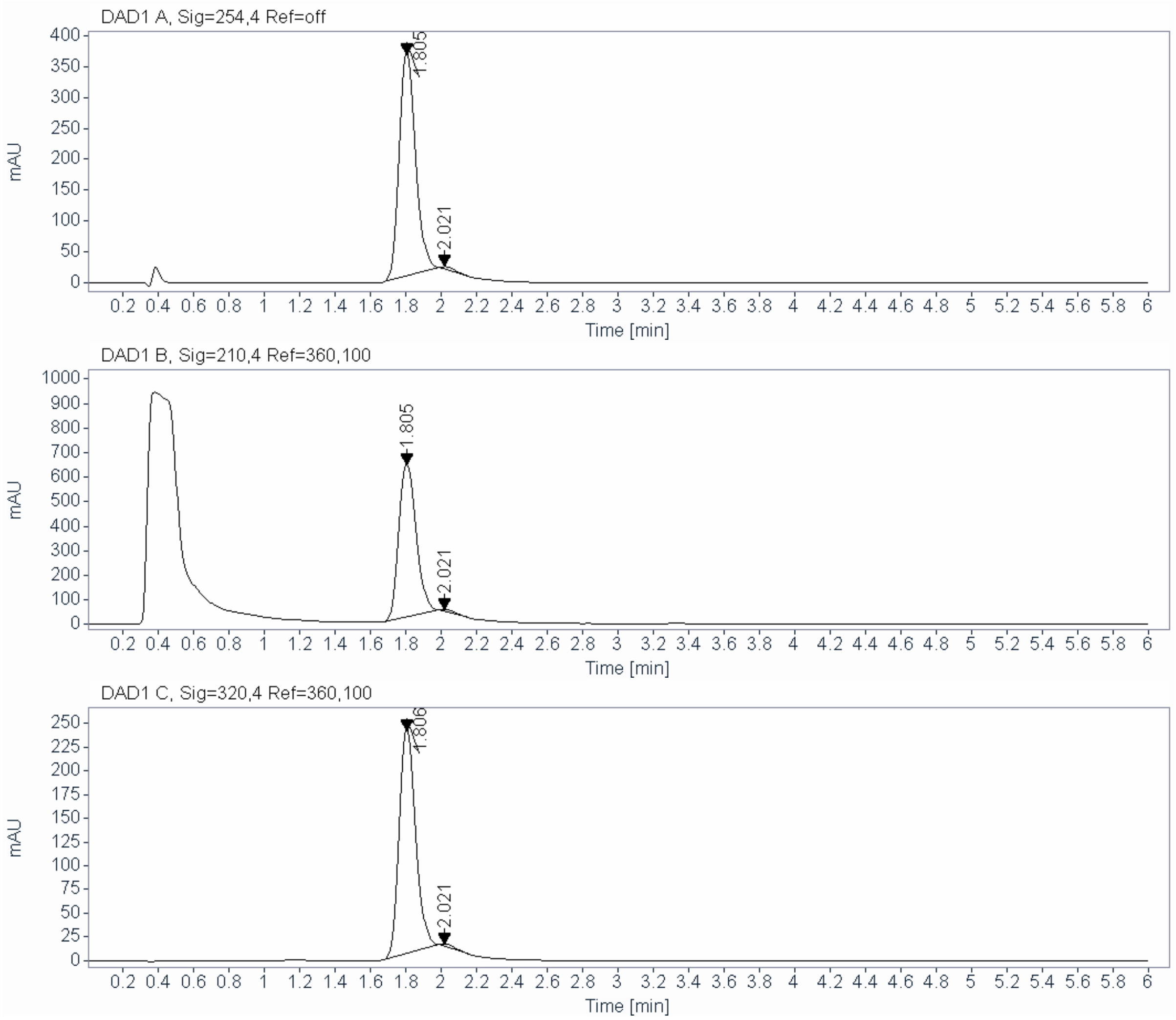

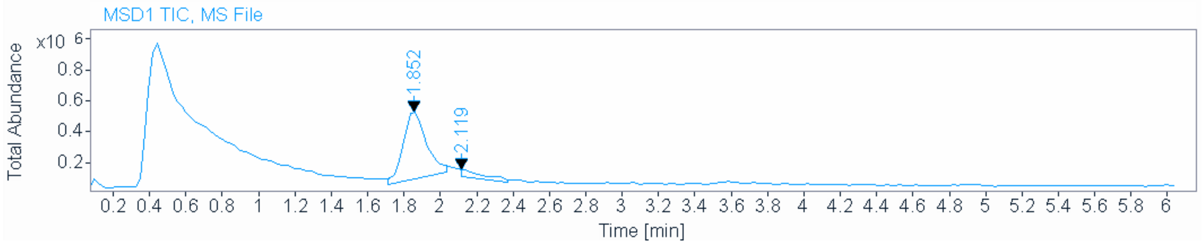

Agilent Technologies

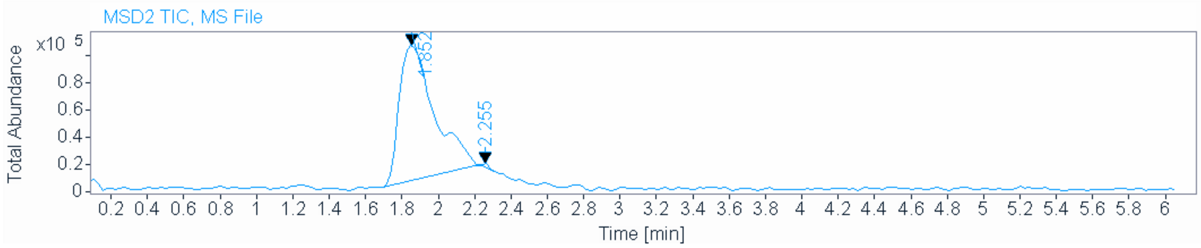

Signal: DAD1 A, Sig=254,4 Ref=off

RT [min]	Type	Width [min]	Area	Height	Area%	Name
1.805	BB	0.0992	2313.2998	359.0864	98.9767	
2.021	BB	0.0994	23.9179	3.7028	1.0233	
		Sum	2337.2177			

Signal: DAD1 B, Sig=210,4 Ref=360,100

RT [min]	Type	Width [min]	Area	Height	Area%	Name
1.805	BB	0.1085	4277.0776	619.9849	98.7618	
2.021	BB	0.0939	53.6215	8.4684	1.2382	
		Sum	4330.6991			

Signal: DAD1 C, Sig=320,4 Ref=360,100

RT [min]	Type	Width [min]	Area	Height	Area%	Name
1.806	BB	0.0994	1525.2117	235.9245	98.9501	
2.021	BB	0.1016	16.1825	2.4970	1.0499	
		Sum	1541.3942			

Signal: MSD1 TIC, MS File

RT [min]	Type	Width [min]	Area	Height	Area%	Name
1.852	MM	0.1444	3831371.500<br>0	442224.1875	90.4917	
2.119	MM	0.1411	402574.1563	47541.3945	9.5083	
		Sum	4233945.656			

Signal: MSD2 TIC, MS File

RT [min]	Type	Width [min]	Area	Height	Area%	Name
1.852	BB	0.2045	1328083.625<br>0	99080.3281	99.5167	
2.255	BB	0.0376	6449.6875	2858.1807	0.4833	
		Sum	1334533.312			

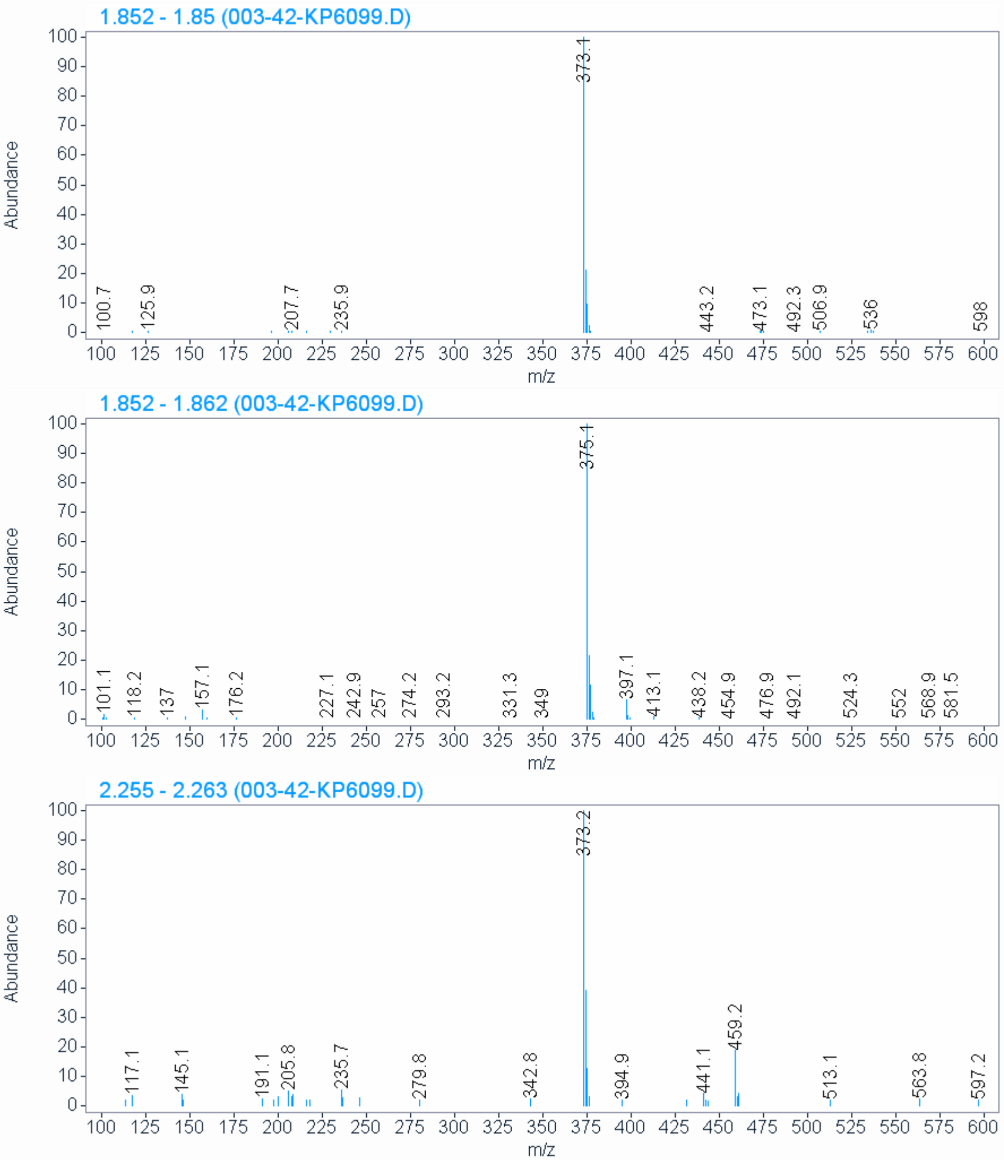
1.852 - 1.85 (003-42-KP6099.D)
Abundance
m/z
373.1
100.7
125.9
207.7
235.9
443.2
473.1
492.3
506.9
536
598
1.852 - 1.862 (003-42-KP6099.D)
375.1
101.1
118.2
137
157.1
176.2
227.1
242.9
257
274.2
293.2
331.3
349
397.1
413.1
438.2
454.9
476.9
492.1
524.3
552
568.9
581.5
2.255 - 2.263 (003-42-KP6099.D)
373.2
117.1
145.1
191.1
205.8
235.7
279.8
342.8
394.9
441.1
459.2
513.1
563.8
597.2

Compound Name: (*Z*)-*N*-(5-octylidene-4-oxo-4,5-dihydrothiazol-2-yl)naphthalene-1-sulfonamide

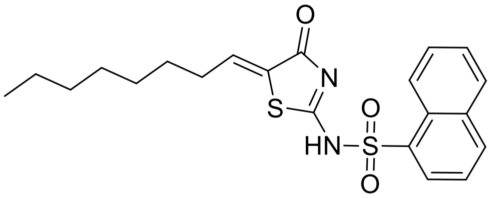

Chemical Formula: $C_{21}H_{24}N_2O_3S_2$
Exact Mass: 416.12
Molecular Weight: 416.56

Compound Code: 43 (KP9027)

Obtained Weight & Yield: 144 mg (71%)

Purity (by LCMS and ^{1}H NMR): > 99% by ^{1}H-NMR and LCMS

Appearance: white solid

Solubility: DMSO, slightly soluble in acetone and methanol.

Melting Point: > 125 °C (dec.)

TLC Rf (and conditions): N/A

IR Analysis (including assignment): IR (neat): ν_{max} = 3060 (C-H aromatic), 2947, 2929, 2858 (C-H alkyl), 1714 (C=O), 1542 (C-C aromatic), 1345 (sulfonamide), 1131 (C-N) cm^{-1}

^{1}H NMR Analysis: ^{1}H NMR (600 MHz, DMSO) δ 12.95 (s, 1H, br, NH), 8.58 (d, *J* = 8.4 Hz, 1H), 8.27 (dd, *J* = 23.3, 7.4 Hz, 2H), 8.11 (d, *J* = 7.6 Hz, 1H), 7.75 (t, *J* = 7.4 Hz, 1H), 7.69 (d, *J* = 6.7 Hz, 2H), 6.90 (t, *J* = 7.4 Hz, 1H), 2.26 (d, *J* = 6.3 Hz, 2H), 1.49 (s, 2H), 1.27 (s, 8H), 0.85 (s, 3H) ppm.
Some aldehyde starting material at 9.34 ppm (0.76%)

^{13}C NMR Analysis: ^{13}C DEPTQ (151 MHz, DMSO) δ 165.7, 165.0, 139.7, 135.2, 134.7, 133.8, 129.0, 128.2, 128.1, 127.6, 127.1, 125.0, 124.9, 124.6, 31.1 (2C), 28.6, 28.4, 27.3, 22.0, 13.9 ppm.

MS Analysis (low res): LRMS (ESI-) *m/z*: 415 (*M*-H, $C_{21}H_{23}N_2O_3S_2$, 100); (ESI+) *m/z*: 417 (*M*+H, $C_{21}H_{25}N_2O_3S_2$, 100)

MS Analysis (high res): Exact mass calculated for $C_{21}H_{23}N_2O_3S_2$ [*M*-H]$^-$, 415.1200. Found 415.1156.

HPLC method details: Column: Zorbax SB-C18 Rapid Resolution HT 2.1x50mm 1.8-Micron; Method: LCMS ISOCRATIC 80%B_3 MINS.M filename: KP9027; Peak retention time: 1.596 mins; Area (%): 100.

Procedure: To a 10 mL microwave vial was added *N*-(4-oxo-4,5-dihydrothiazol-2-yl)naphthalene-1-sulfonamide (157 mg, 0.49 mmol), octanal (0.11 mL, 0.735 mmol, 1.1 eq), ethanol (3 mL) and a catalytic amount of the benzoic acid/piperidine catalyst (approximately 5 drops). The suspension was heated by microwave irradiation (120 °C, 200 W) for 45 min. A precipitate formed upon addition of H_2O (5 mL) and stirring at RT (overnight). The solid was collected by vacuum filtration to give the desired product as a white solid (144 mg, 71%).

Other analyses, reference papers, previously obtained data, comments, etc:

PerkinElmer Spectrum Version 10.4.00
Thursday, 26 November 2020 11:53 AM

Analyst research
Date Thursday, 26 November 2020 11:53 AM

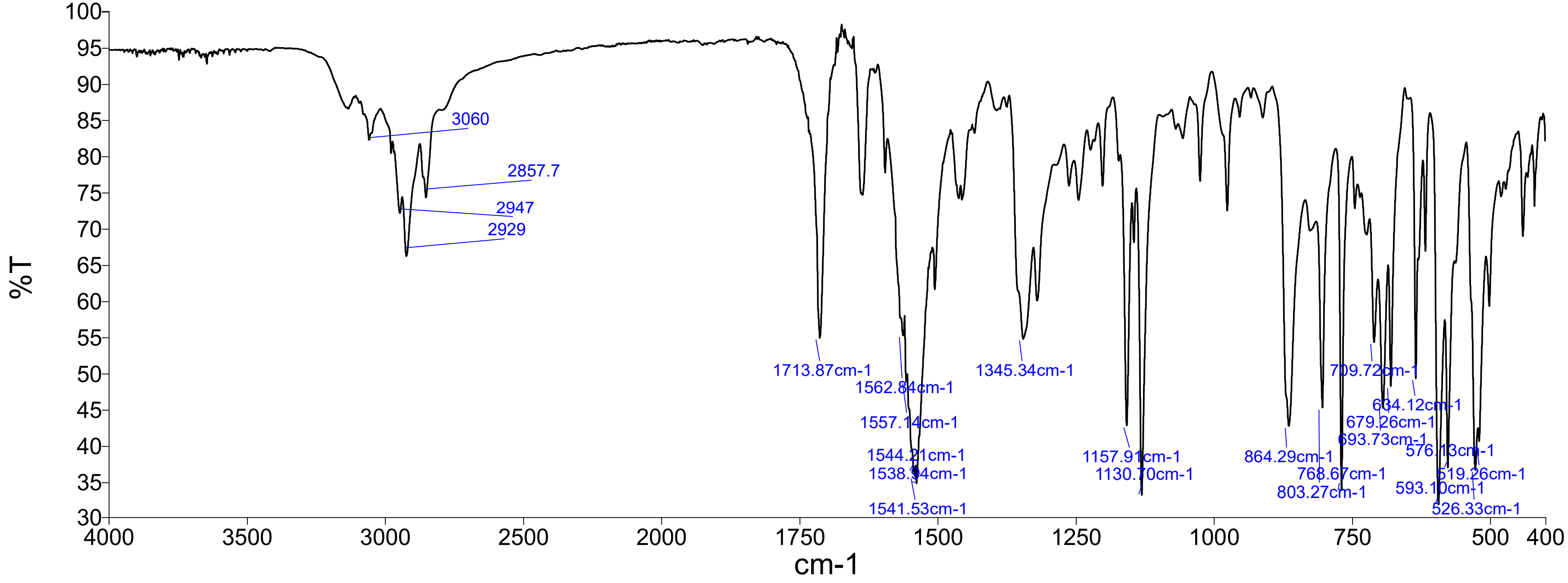

Sample Name	Description	Quality Checks
kp9027	Sample 175 By research Date Thursday, November 26 2020	The Quality Checks do not report any warnings for the sample.

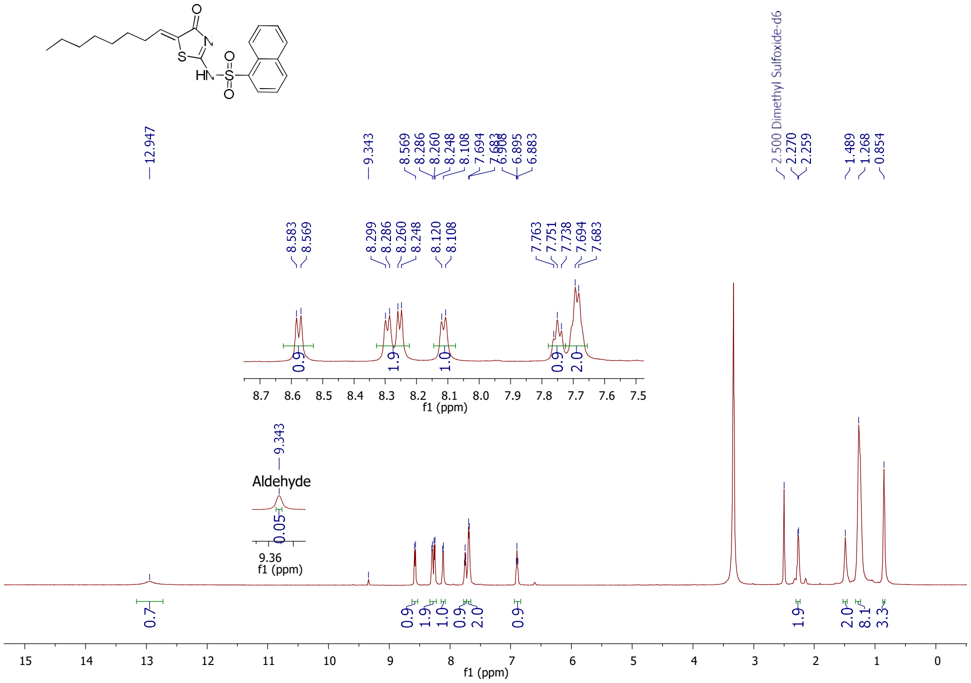

12.947
9.343
8.569
8.286
8.260
8.248
8.108
7.694
7.683
6.908
6.895
6.883
2.500 Dimethyl Sulfoxide-d6
2.270
2.259
1.489
1.268
0.854
8.583
8.569
8.299
8.286
8.260
8.248
8.120
8.108
7.763
7.751
7.738
7.694
7.683
Aldehyde
9.343
0.05
9.36
f1 (ppm)
f1 (ppm)

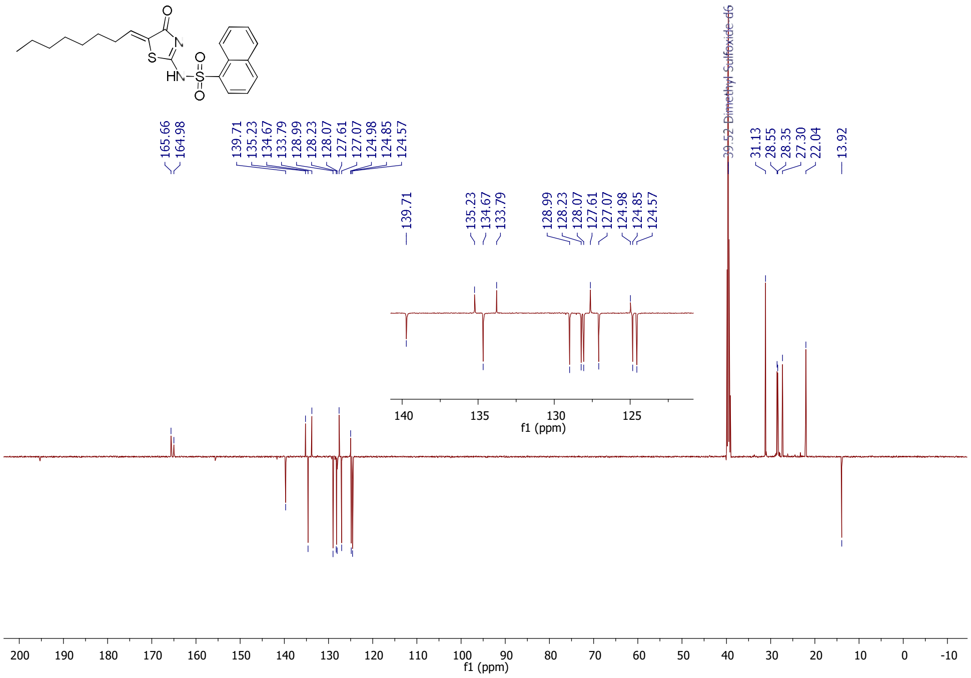

165.66
164.98
139.71
135.23
134.67
133.79
128.99
128.23
128.07
127.61
127.07
124.98
124.85
124.57
39.52 Dimethyl Sulfoxide-d6
31.13
28.55
28.35
27.30
22.04
13.92
f1 (ppm)
f1 (ppm)

LCMS Report

Data file: D:\Chem32\1\Data\KP\KP_DS_NOV4 2020-11-02 13-34-37\004-18-KP9027.D
Sample name: KP9027
Description:
Sample amount: 0.000
Sample type: Sample

Instrument: LCMS
Location: 18
Injection date: 11/2/2020 1:50:06 PM
Injection: 1 of 1
Acq. method: LCMS ISOCRATIC 80% B_3 MINS.M
Injection volume: 2.000
Analysis method: LCMS ISOCRATIC 80%B_3 MINS.M
Acq. operator: SYSTEM
Last changed: 10/8/2020 2:52:31 PM

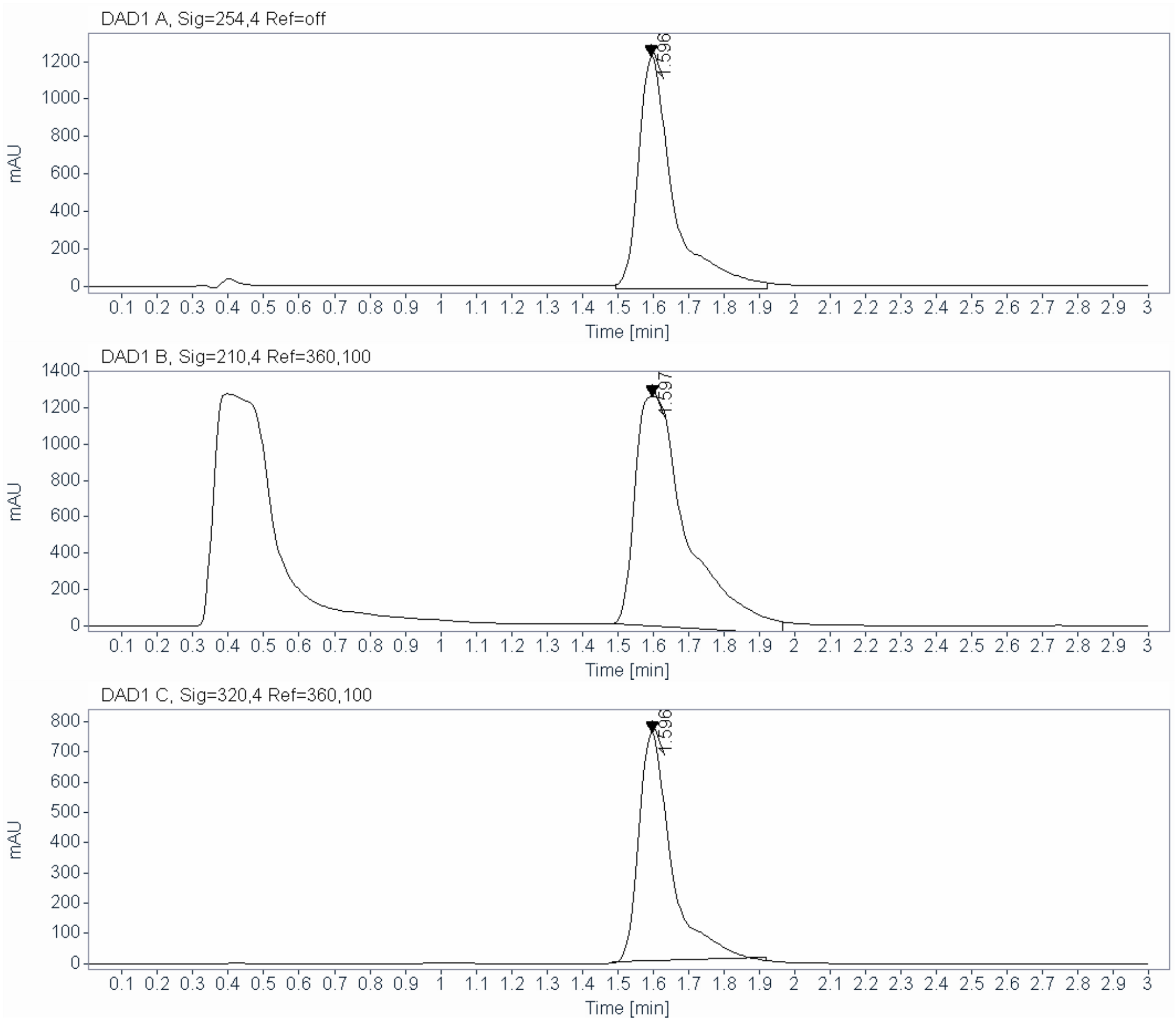

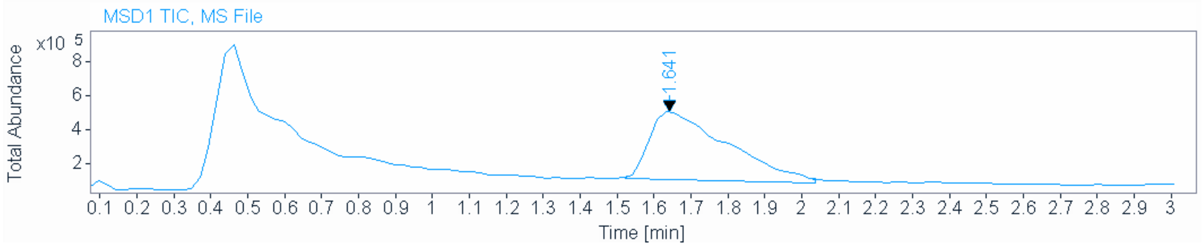

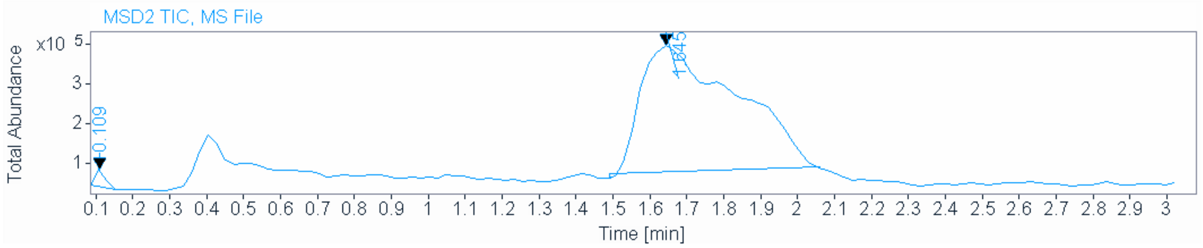

Signal: DAD1 A, Sig=254,4 Ref=off

RT [min]	Type	Width [min]	Area	Height	Area%	Name
1.596	MM	0.1136	8497.7832	1246.6945	100.0000	
		Sum	8497.7832			

Signal: DAD1 B, Sig=210,4 Ref=360,100

RT [min]	Type	Width [min]	Area	Height	Area%	Name
1.597	MM	0.1726	13020.5938	1257.0244	100.0000	
		Sum	13020.5938			

Signal: DAD1 C, Sig=320,4 Ref=360,100

RT [min]	Type	Width [min]	Area	Height	Area%	Name
1.596	MM	0.1054	4782.4580	756.0252	100.0000	
		Sum	4782.4580			

Signal: MSD1 TIC, MS File

RT [min]	Type	Width [min]	Area	Height	Area%	Name
1.641	MM	0.2430	5963144.5000	409017.7500	100.0000	
		Sum	5963144.500			

Signal: MSD2 TIC, MS File

RT [min]	Type	Width [min]	Area	Height	Area%	Name
0.109	BB	0.0330	87204.0000	44000.4258	1.5126	
1.645	MM	0.2980	5677856.0000	317590.6250	98.4874	
		Sum	5765060.000			

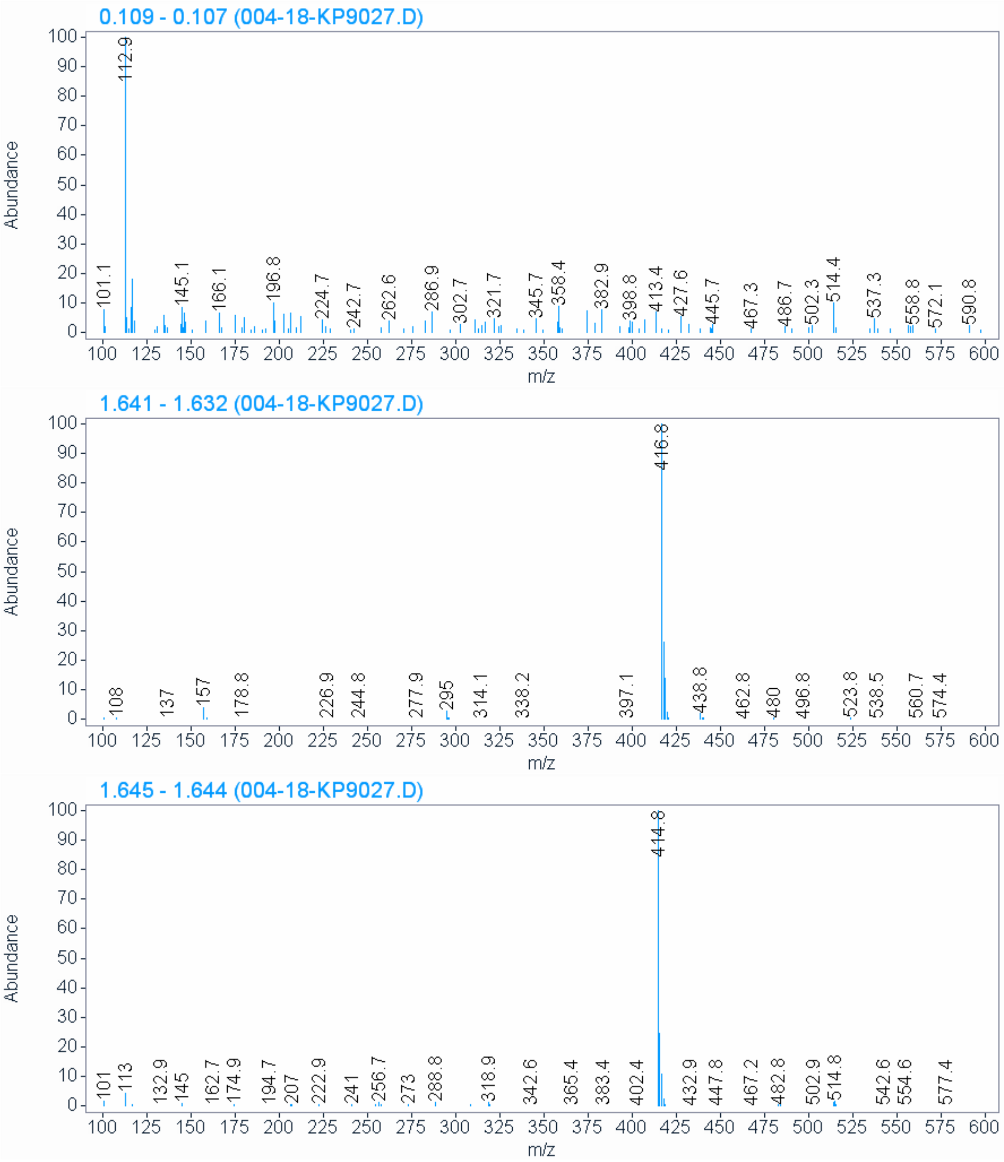
0.109 - 0.107 (004-18-KP9027.D)
1.641 - 1.632 (004-18-KP9027.D)
1.645 - 1.644 (004-18-KP9027.D)
Abundance
m/z

Compound Name: (Z)-*N*-(4-oxo-5-(thiophen-3-ylmethylene)-4,5-dihydrothiazol-2-yl)naphthalene-1-sulfonamide

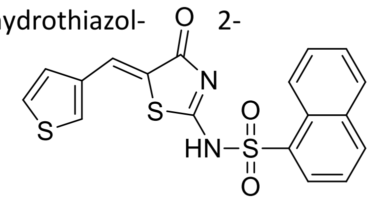

Chemical Formula: $C_{18}H_{12}N_2O_3S_3$
Exact Mass: 400.00
Molecular Weight: 400.49

Compound Code: 44 (KP6085)

Obtained Weight & Yield: 141 mg (73%)

Purity (by LCMS and ^{1}H NMR): > 98% by NMR

Appearance: yellow solid

Solubility: DMSO, slightly soluble in methanol and acetone

Melting Point: < 259 °C (dec.)

TLC Rf (and conditions): 0.46 (10% MeOH in DCM)

IR Analysis (including assignment): IR (neat): ν_{max} = 3102 (N-H), 3062, 3007 (aromatic C-H), 1698 (C=O), 1557 (C=C alkene), 1317 (sulfonamide), 1126 (C-N) cm^{-1}

^{1}H NMR Analysis: ^{1}H NMR (400 MHz, DMSO) δ 13.15 (s, 1H, br), 8.61 (d, *J* = 8.6 Hz, 1H), 8.30 (d, *J* = 7.6 Hz, 2H), 8.12 (d, *J* = 8.0 Hz, 1H), 8.08 (d, *J* = 5.0 Hz, 1H), 8.04 (s, 1H), 7.77 – 7.69 (m, 4H), 7.32 (dd, *J* = 5.0, 3.7 Hz, 1H) ppm.
Diethyl ether at 1.09 ppm (0.22%) and acetone 2.08 ppm (0.84%)

^{13}C NMR Analysis: ^{13}C NMR (101 MHz, DMSO) δ 166.3, 164.9, 137.0, 135.7, 135.3, 134.7, 134.0, 133.8, 129.2, 129.0, 128.3, 128.1, 127.6, 127.1, 126.8, 124.9, 124.6, 119.0 ppm.

MS Analysis (low res): LRMS (ESI-) *m/z*: 399 (*M*-H, $C_{18}H_{11}N_2O_3S_3$, 100%)

HPLC method details: Column: Zorbax SB-C18 Rapid Resolution HT 2.1x50mm 1.8-Micron; Method: LCMS ISOCRATIC 60%B 0.4MLMIN-1.M filename: KP6085R; Peak retention time: 1.25 mins; Area (%): 100.

Procedure: To a microwave vial was added the sulfonamide intermediate (147 mg, 0.48 mmol), 2-thiophene carboxaldehyde (0.05 mL, 0.54 mmol, 1.1 eq), ethanol (3 mL) and the benzoic acid/piperidine catalyst (3 drops). The reaction mixture was treated with microwave irradiation (120°C, 30 min). After cooling a precipitate was collected by vacuum filtration. The precipitate was washed with H_2O (2 mL), cold ethanol (5 mL) and cold diethyl ether (10 mL) to give the desired product as a yellow solid (141 mg, 73%).

Other analyses, reference papers, previously obtained data, comments, etc:

Analyst	analyst1
Date	Thursday, 4 July 2019 12:45 PM

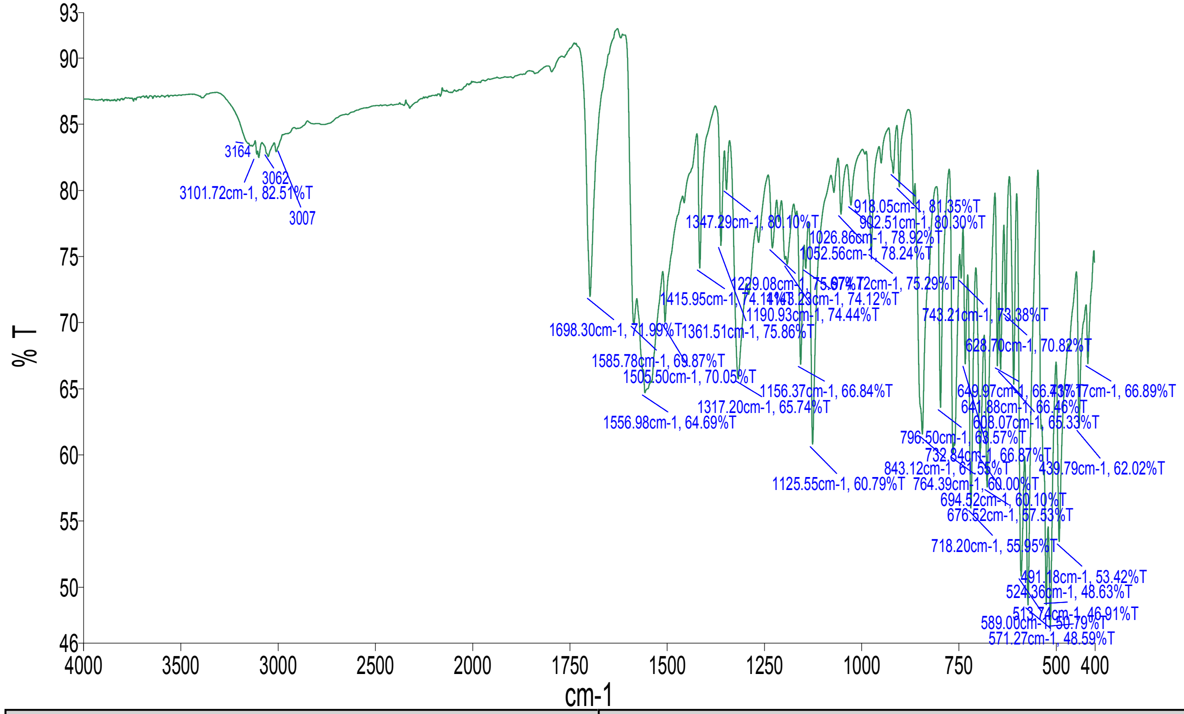

Sample Name	Description	Quality Checks
KP6085	Sample 014 By Analyst1 Date Thursday, July 04 2019	The Quality Checks do not report any warnings for the sample.

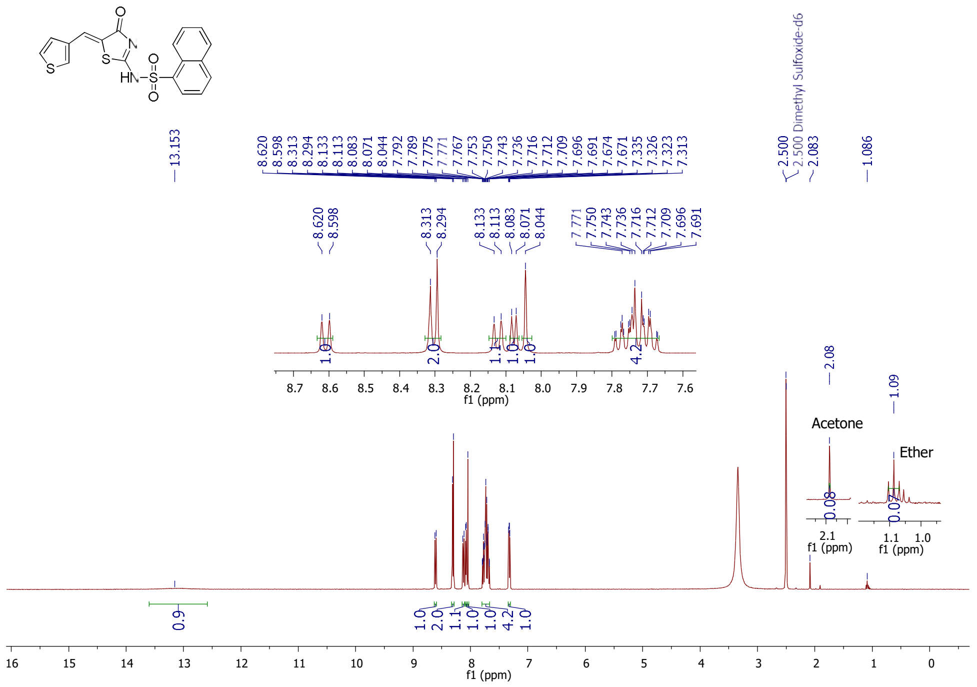
13.153
8.620
8.598
8.313
8.294
8.133
8.113
8.083
8.071
8.044
7.792
7.789
7.775
7.771
7.767
7.753
7.750
7.743
7.736
7.716
7.712
7.709
7.696
7.691
7.674
7.671
7.335
7.326
7.323
7.313
2.500
2.500 Dimethyl Sulfoxide-d6
2.083
1.086
Acetone
Ether
f1 (ppm)

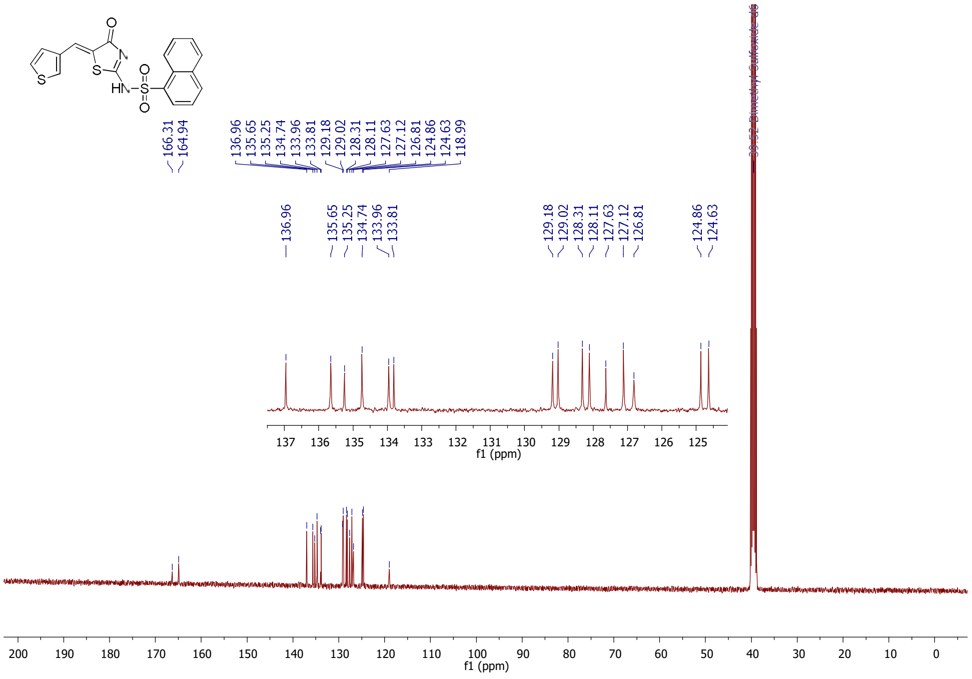
166.31
164.94
136.96
135.65
135.25
134.74
133.96
133.81
129.18
129.02
128.31
128.11
127.63
127.12
126.81
124.86
124.63
118.99
f1 (ppm)

LCMS Report

Agilent Technologies

**Data file:**	D:\Chem32\1\Data\KP\KP60788587 2019-08-14 14-27-41\003-52-KP6085R.D		
**Sample name:**	KP6085R		
**Description:**			
**Sample amount:**	0.000	**Sample type:**	Sample
**Instrument:**	LCMS	**Location:**	52
**Injection date:**	8/14/2019 2:44:26 PM	**Injection:**	1 of 1
**Acq. method:**	LCMS ISOCRATIC 60% B 0.4MLMIN-1.M	**Injection volume:**	2.000
**Analysis method:**	LCMS ISOCRATIC 60%B 0.4MLMIN-1.M	**Acq. operator:**	SYSTEM
**Last changed:**	5/8/2019 8:55:04 AM		

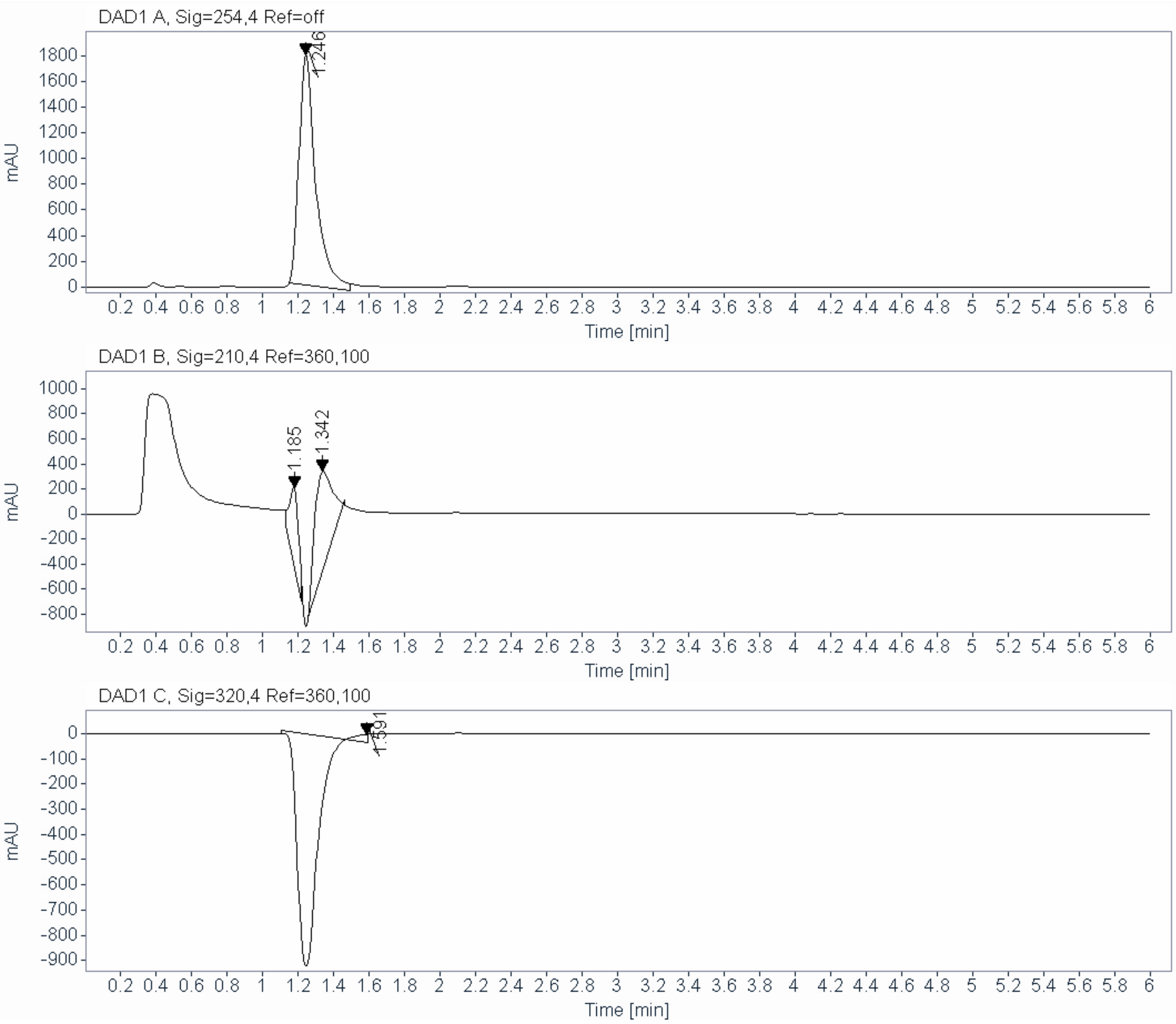

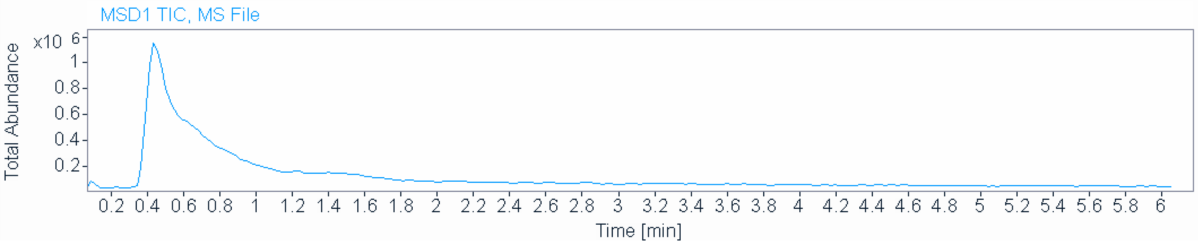

LCMS Report

Agilent Technologies

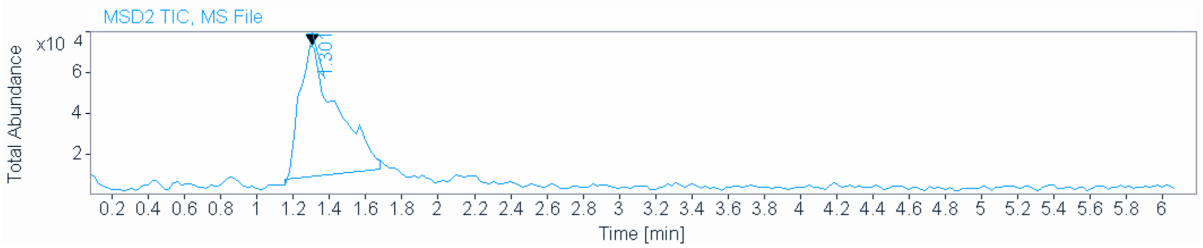

Signal: DAD1 A, Sig=254,4 Ref=off

RT [min]	Type	Width [min]	Area	Height	Area%	Name
1.246	MM	0.1095	11817.3145	1799.3264	100.0000	
		Sum	11817.3145			

Signal: DAD1 B, Sig=210,4 Ref=360,100

RT [min]	Type	Width [min]	Area	Height	Area%	Name
1.185	MM	0.0567	2237.9558	657.9059	29.8763	
1.342	MM	0.1097	5252.7900	797.8452	70.1237	
		Sum	7490.7458			

Signal: DAD1 C, Sig=320,4 Ref=360,100

RT [min]	Type	Width [min]	Area	Height	Area%	Name
1.591	MM	0.0744	143.4748	32.1227	100.0000	
		Sum	143.4748			

Signal: MSD2 TIC, MS File

RT [min]	Type	Width [min]	Area	Height	Area%	Name
1.301	MM	0.2353	925785.2500	65573.3828	100.0000	
		Sum	925785.2500			

Agilent Technologies

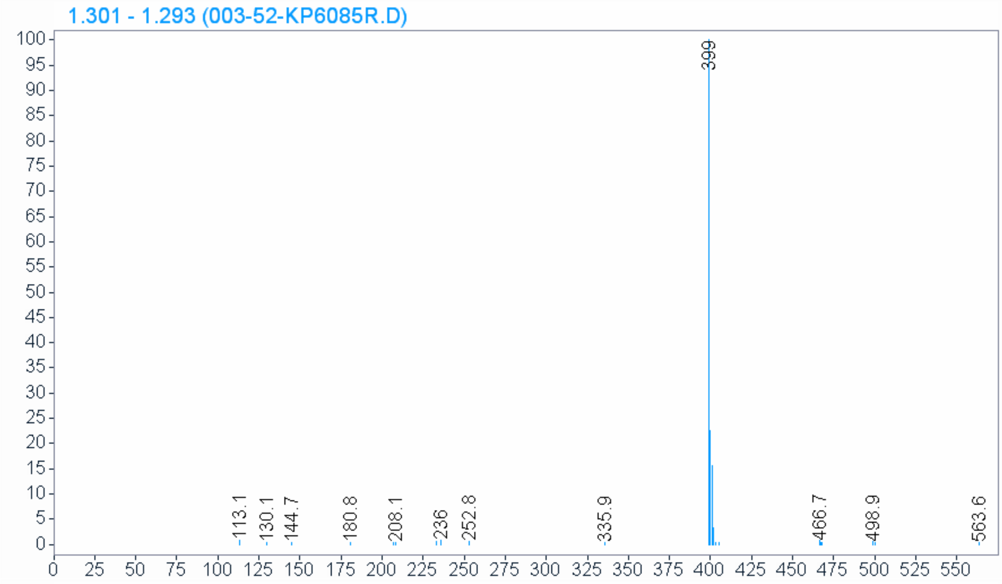

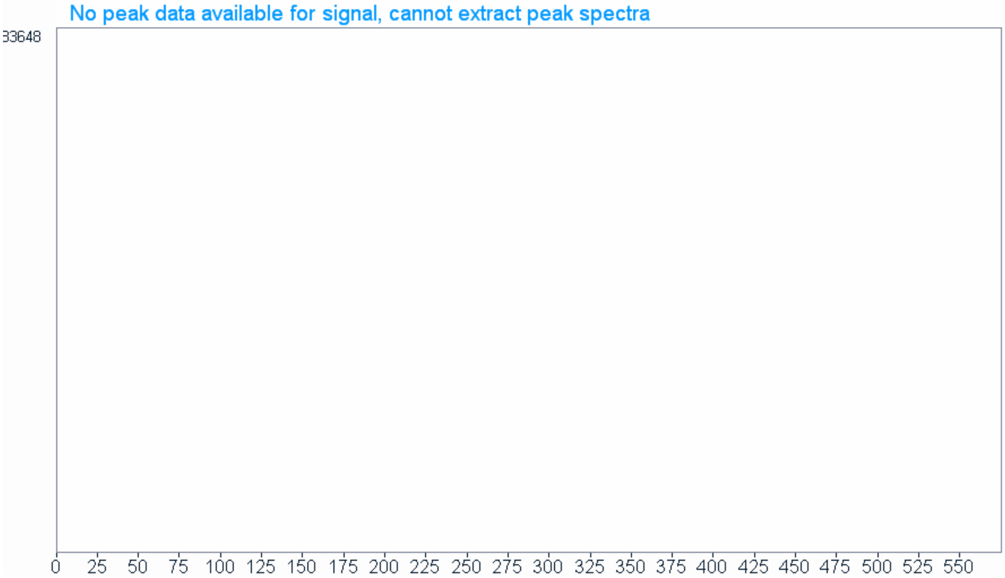

Compound Name: (Z)-*N*-(5-((1H-pyrrol-3-yl)methylene)-4-oxo-4,5-dihydrothiazol-2-yl)naphthalene-1-sulfonamide

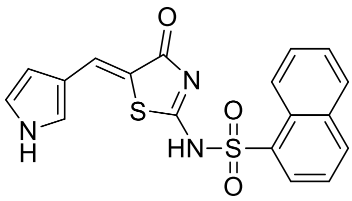

Chemical Formula: $C_{18}H_{13}N_3O_3S_2$
Exact Mass: 383.04
Molecular Weight: 383.44

Compound Code: 45 (KP6084)

Obtained Weight & Yield: 53 mg (28%)

Purity (by LCMS and ^{1}H NMR): > 95% by ^{1}H-NMR

Appearance: yellow/brown solid

Solubility: DMSO, slightly soluble in methanol.

Melting Point: < 264 °C (dec.)

TLC Rf (and conditions): 0.42 (10% MeOH in DCM)

IR Analysis (including assignment): IR (neat): ν_{max} = 3639, 3498 (N-H), 3177, 3042 (aromatic C-H),1695 (C=O), 1591 (C=C alkene), 1538 (C-C aromatic), 1355 (sulfonamide), 1123 (C-N) cm^{-1}

^{1}H NMR Analysis: ^{1}H NMR (400 MHz, DMSO) δ 12.84 (s, 1H), 11.70 (s, 1H), 8.61 (d, *J* = 8.6 Hz, 1H), 8.32 – 8.27 (m, 2H), 8.11 (d, *J* = 8.0 Hz, 1H), 7.78 – 7.66 (m, 4H), 7.48 – 7.47 (m, 1H), 7.04 (m, 1H), 6.37 (d, *J* = 2.3 Hz, 1H) ppm.
Acetone at 2.08 ppm (1.35%) and diethyl ether at 1.09ppm (1.07%)

^{13}C NMR Analysis: ^{13}C NMR (101 MHz, DMSO) δ 166.5, 165.9, 135.5, 134.5, 133.8, 130.4, 129.0, 128.2, 128.1, 127.7, 127.0, 126.6, 124.9, 124.6, 122.0 118.0, 113.2, 107.9 ppm.

MS Analysis (low res): LRMS (ESI+) *m/z*: 384 (*M*+H, $C_{18}H_{14}N_3O_3S_2$, 40%); (ESI-) *m/z*: 382 (*M*-H, $C_{18}H_{12}N_3O_3S_2$, 100%)

HPLC method details: Column: Zorbax SB-C18 Rapid Resolution HT 2.1x50mm 1.8-Micron; Method: LCMS ISOCRATIC 60%B 0.4MLMIN-1.M filename: KP6084; Peak retention time: 0.66 mins; Area (%): 100.

Procedure: To a microwave vial was added the *N*-(4-oxo-4,5-dihydrothiazol-2-yl)naphthalene-1-sulfonamide (150 mg, 0.49 mmol), pyrrole-3-carbonaldehyde (55 mg, 0.58 mmol, 1.2 eq), ethanol (3 mL) and the benzoic acid/piperidine catalyst (3 drops). The reaction mixture was treated with microwave irradiation (120°C, 30 min). After cooling a precipitate was collected by vacuum filtration. The precipitate was washed with H_2O (2 mL), cold ethanol (5 mL) and cold diethyl ether (10 mL) to give the desired product as a yellow/brown solid (53 mg, 28%).

Other analyses, reference papers, previously obtained data, comments, etc:

PerkinElmer Spectrum Version 10.4.4
Thursday, 4 July 2019 12:42 PM

Analyst analyst1
Date Thursday, 4 July 2019 12:42 PM

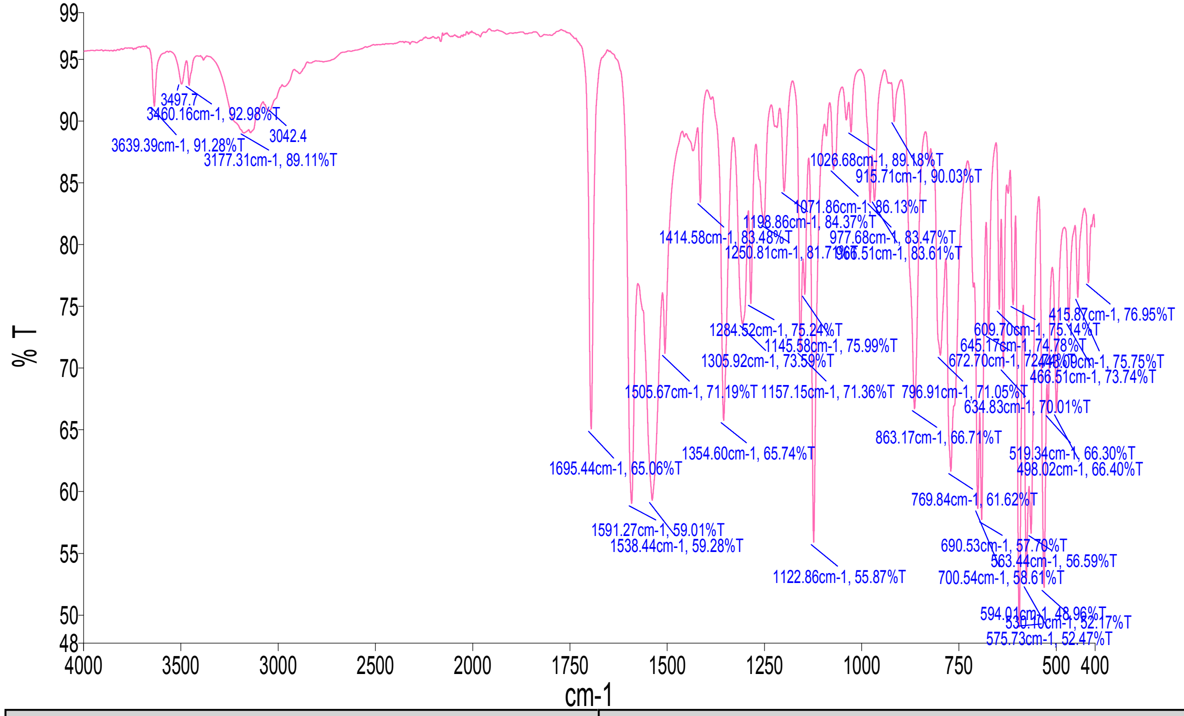

Sample Name	Description	Quality Checks
KP6084	Sample 013 By Analyst1 Date Thursday, July 04 2019	The Quality Checks do not report any warnings for the sample.

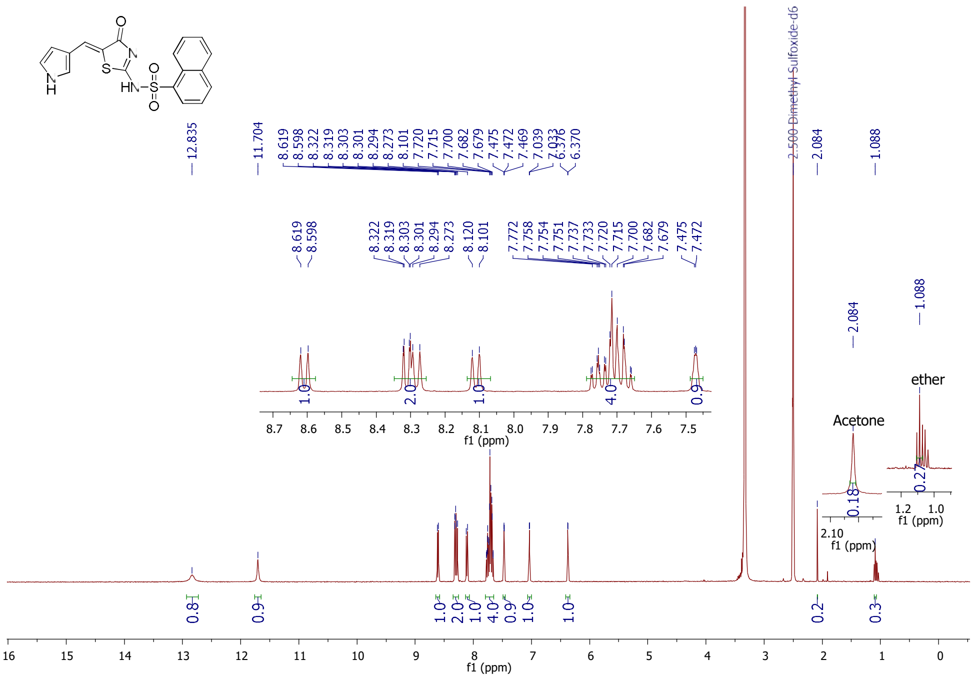

12.835
11.704
8.619 8.598 8.322 8.319 8.303 8.301 8.294 8.273 8.101 7.720 7.715 7.700 7.682 7.679 7.475 7.472 7.469 7.039 7.033 6.376 6.370
2.500 Dimethyl Sulfoxide-d6
2.084
1.088
8.619 8.598 8.322 8.319 8.303 8.301 8.294 8.273 8.120 8.101 7.772 7.758 7.754 7.751 7.737 7.733 7.720 7.715 7.700 7.682 7.679 7.475 7.472
1.0 2.0 1.0 4.0 0.9
Acetone
0.18
ether
0.27
0.8 0.9 1.0 2.0 1.0 4.0 0.9 1.0 1.0 0.2 0.3
f1 (ppm)

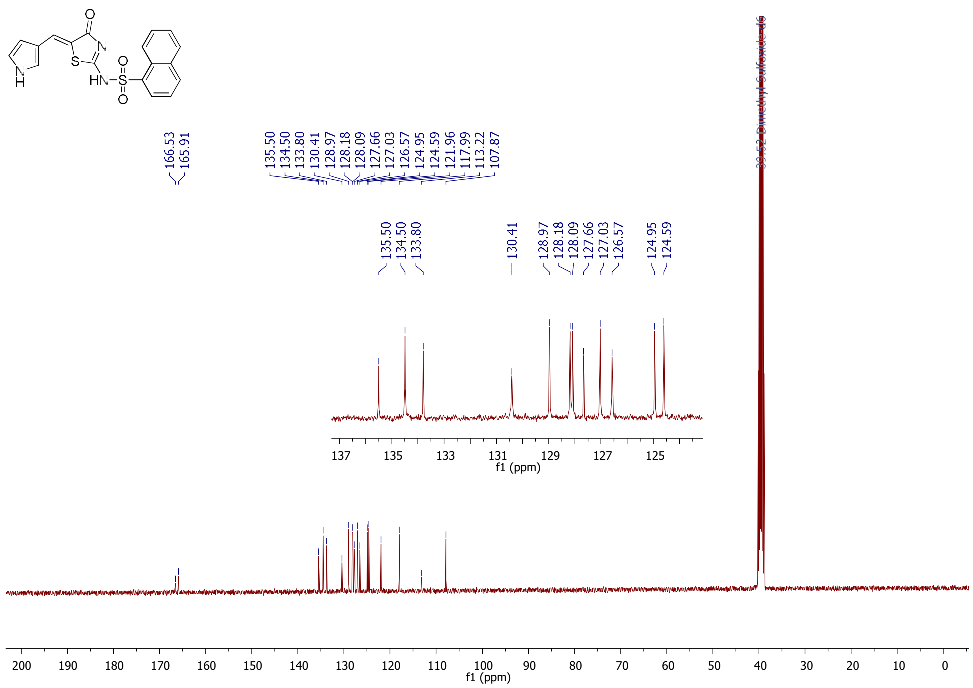

166.53 165.91
135.50 134.50 133.80 130.41 128.97 128.18 128.09 127.66 127.03 126.57 124.95 124.59 121.96 117.99 113.22 107.87
39.52 Dimethyl Sulfoxide-d6
135.50 134.50 133.80
130.41
128.97 128.18 128.09 127.66 127.03 126.57
124.95 124.59
f1 (ppm)

LCMS Report

Agilent Technologies

**Data file:**	D:\Chem32\1\Data\KP\KP60838485 2019-08-13 14-20-02\003-48-KP6084.D		
**Sample name:**	KP6084		
**Description:**			
**Sample amount:**	0.000	**Sample type:**	Sample
**Instrument:**	LCMS	**Location:**	48
**Injection date:**	8/13/2019 2:36:55 PM	**Injection:**	1 of 1
**Acq. method:**	LCMS ISOCRATIC 60% B 0.4MLMIN-1.M	**Injection volume:**	2.000
**Analysis method:**	LCMS ISOCRATIC 60%B 0.4MLMIN-1.M	**Acq. operator:**	SYSTEM
**Last changed:**	5/8/2019 8:55:04 AM		

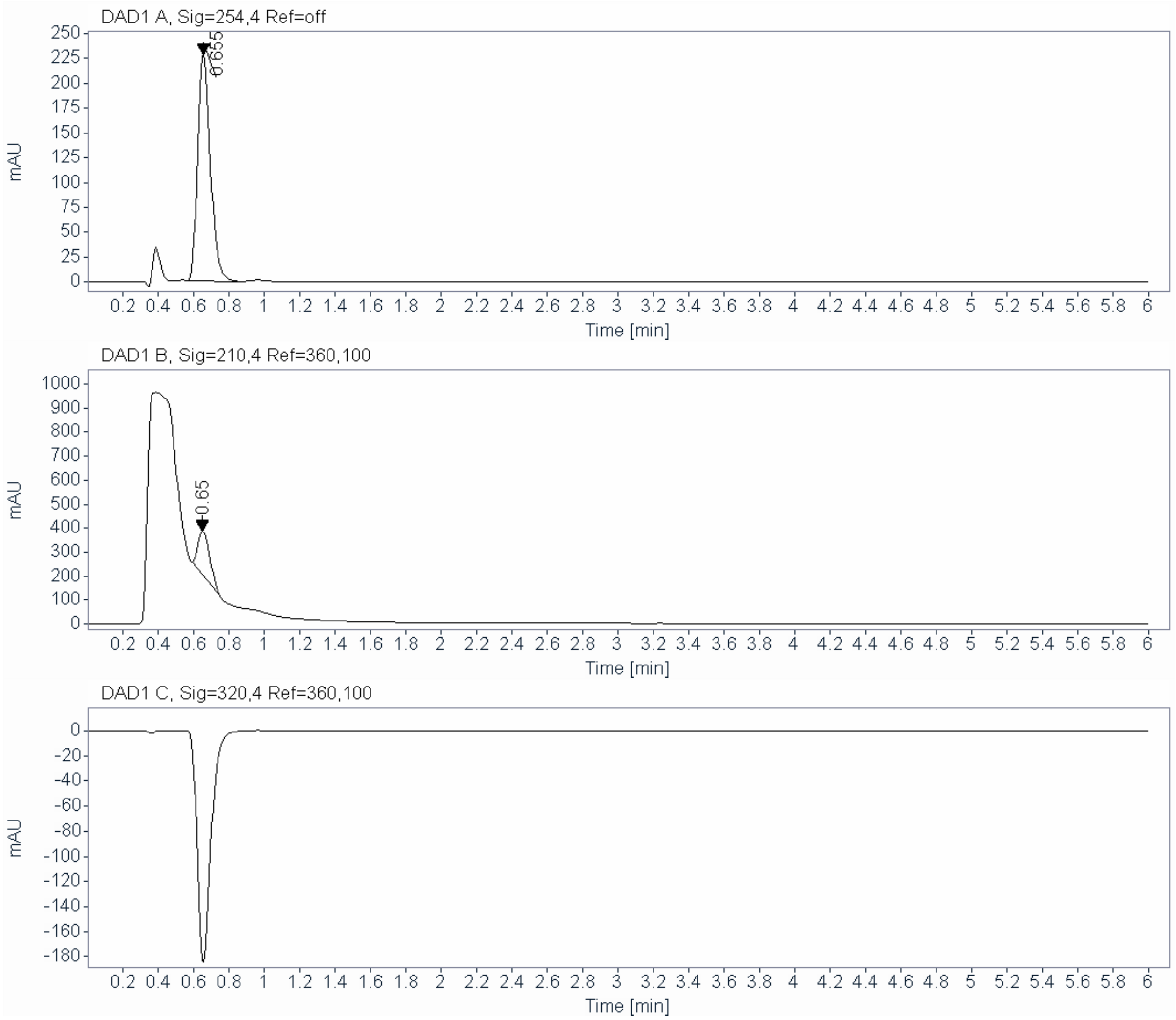

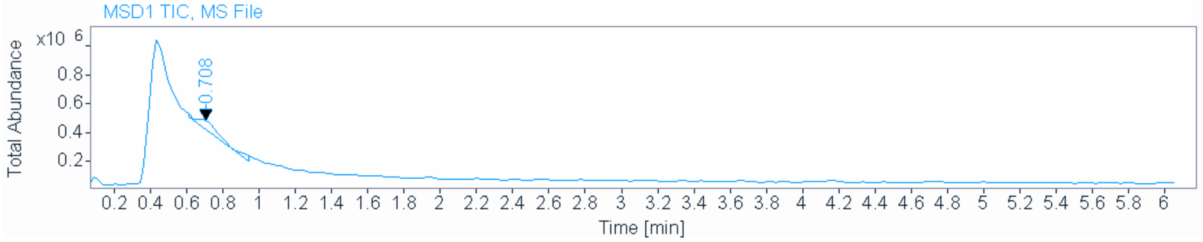

Agilent Technologies

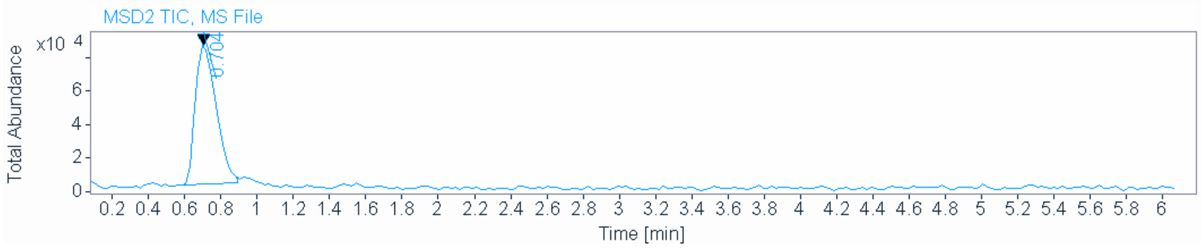

Signal: DAD1 A, Sig=254,4 Ref=off

RT [min]	Type	Width [min]	Area	Height	Area%	Name
0.655	BB	0.0788	1149.4003	228.2130	100.0000	
		Sum	1149.4003			

Signal: DAD1 B, Sig=210,4 Ref=360,100

RT [min]	Type	Width [min]	Area	Height	Area%	Name
0.650	BB	0.0812	889.4092	181.3343	100.0000	
		Sum	889.4092			

Signal: MSD1 TIC, MS File

RT [min]	Type	Width [min]	Area	Height	Area%	Name
0.708	MM	0.1494	535944.6875	59790.1719	100.0000	
		Sum	535944.6875			

Signal: MSD2 TIC, MS File

RT [min]	Type	Width [min]	Area	Height	Area%	Name
0.704	MM	0.1310	659296.2500	83887.8750	100.0000	
		Sum	659296.2500			

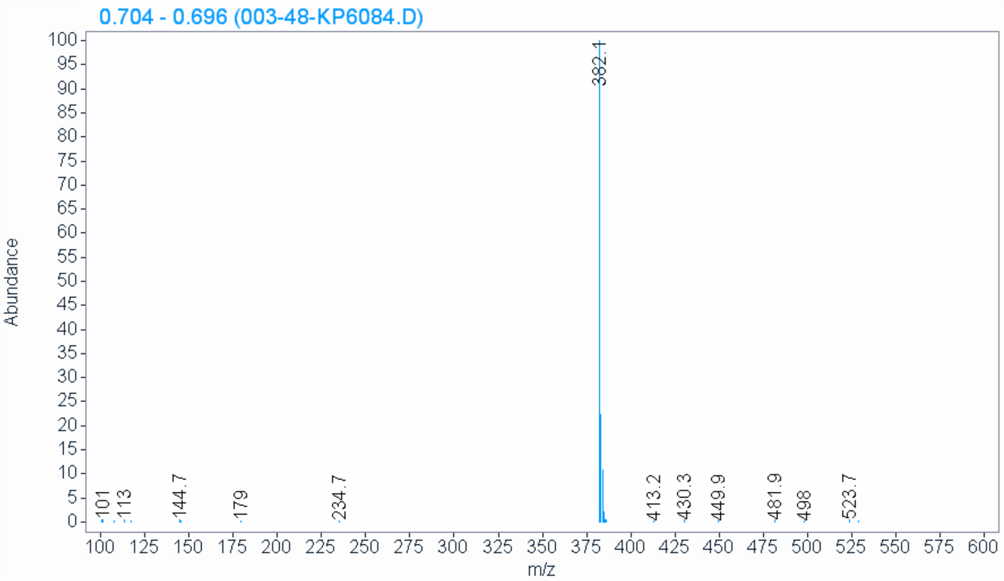
0.704 - 0.696 (003-48-KP6084.D)
Abundance
m/z
382.1
101
113
144.7
179
234.7
413.2
430.3
449.9
481.9
498
523.7

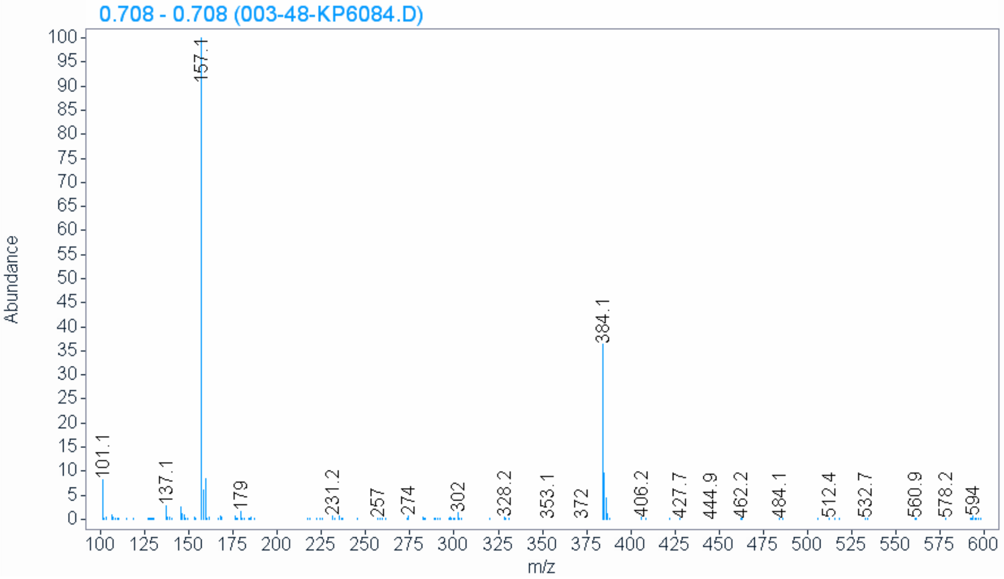
0.708 - 0.708 (003-48-KP6084.D)
Abundance
m/z
157.1
384.1
101.1
137.1
179
231.2
257
274
302
328.2
353.1
372
406.2
427.7
444.9
462.2
484.1
512.4
532.7
560.9
578.2
594

Compound Name: (Z)-*N*-(5-(furan-3-ylmethylene)-4-oxo-4,5-dihydrothiazol-2-yl)naphthalene-1-sulfonamide

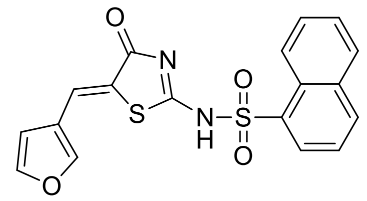

Chemical Formula: $C_{18}H_{12}N_2O_4S_2$
Exact Mass: 384.02
Molecular Weight: 384.43

Compound Code: 46 (KP6091)

Obtained Weight & Yield: 145 mg (78%)

Purity (by LCMS and ^{1}H NMR): > 99% by ^{1}NMR and LCMS

Appearance: Light brown solid

Solubility: DMSO, slightly soluble in acetone

Melting Point: > 245 °C (dec.)

TLC Rf (and conditions): 0.42 (10% MeOH in DCM)

IR Analysis (including assignment): IR (neat): v_{max} = 3138 (N-H), 3026, 2958 (aromatic C-H), 1705 (C=O), 1611 (C=C), 1543 (aromatic C-C), 1338 (sulfonamide), 1558 (C-O-C), 1130 (C-N) cm^{-1}

^{1}H NMR Analysis: ^{1}H NMR (400 MHz, DMSO) δ 13.08 (br, s, 1H, NH), 8.61 (d, *J* = 8.5 Hz, 1H), 8.34 – 8.29 (m, 3H), 8.12 (d, *J* = 8.1 Hz, 1H), 7.93 (s, 1H), 7.79 – 7.75 (m, 1H), 7.73 – 7.67 (m, 3H), 6.80 (d, *J* = 1.3 Hz, 1H) ppm.

^{13}C NMR Analysis: ^{13}C NMR (101 MHz, DMSO) δ 166.2, 165.2, 148.2, 146.1, 135.2, 134.7, 133.8, 129.0, 128.28, 128.25, 127.6, 127.1, 125.0, 124.9, 124.6, 120.49, 120.46, 108.9 ppm.

MS Analysis (low res): LRMS (ESI+) *m/z*: 385 (*M*+H, $C_{18}H_{13}N_2O_4S_2$, 100%); (ESI-) *m/z*: 383 (*M*-H, $C_{18}H_{11}N_2O_4S_2$, 100%)

HPLC method details: Column: Zorbax SB-C18 Rapid Resolution HT 2.1x50mm 1.8-Micron; Method: LCMS ISOCRATIC 60%B 0.4MLMIN-1.M filename: KP6091; Peak retention time: 1.02 mins; Area (%): 100.

Procedure: To a microwave vial was added the *N*-(4-oxo-4,5-dihydrothiazol-2-yl)naphthalene-1-sulfonamide (148 mg, 0.48 mmol), 3-furanaldehyde (0.05 mL, 0.54 mmol, 1.1 eq), ethanol (3 mL) and the benzoic acid/piperidine catalyst (3 drops). The reaction mixture was treated with microwave irradiation (120 °C, 30 min). After cooling, a precipitate was collected by vacuum filtration. The precipitate was washed with H_2O (2 mL), cold ethanol (5 mL) and cold diethyl ether (10 mL) to give the desired product as a light brown solid (145 mg, 78%).

Other analyses, reference papers, previously obtained data, comments, etc:

Analyst analyst1
Date Thursday, 4 July 2019 12:49 PM

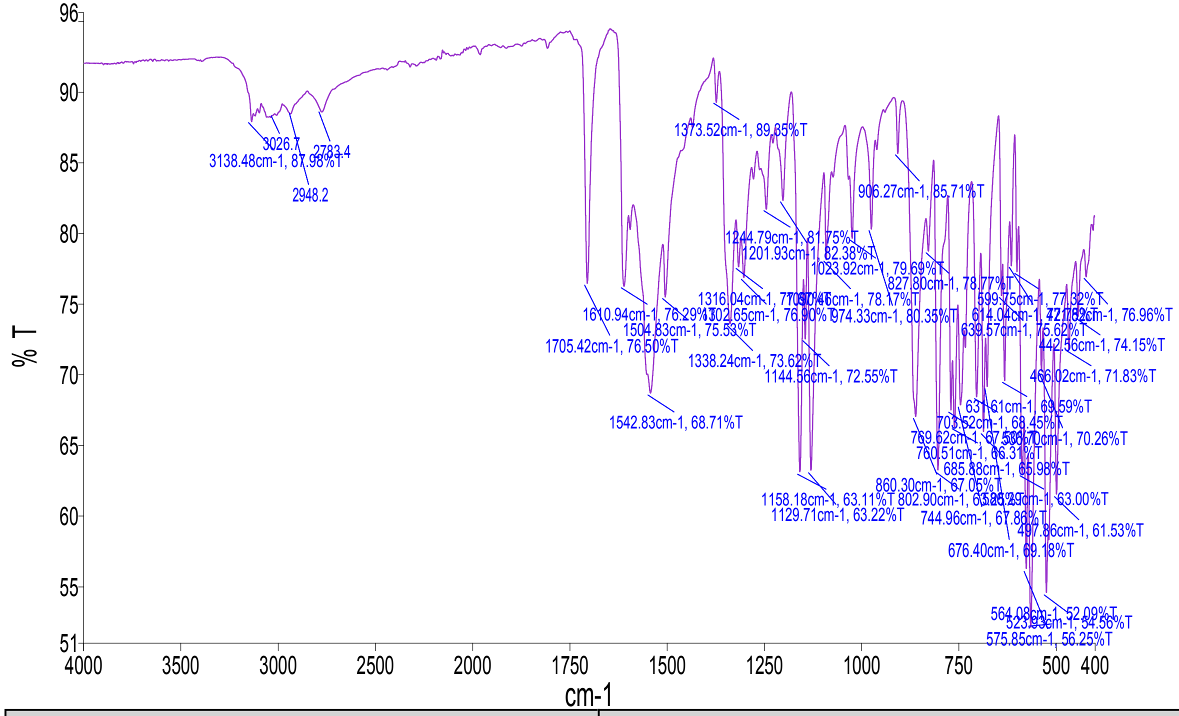

Sample Name	Description	Quality Checks
KP6091	Sample 016 By Analyst1 Date Thursday, July 04 2019	The Quality Checks do not report any warnings for the sample.

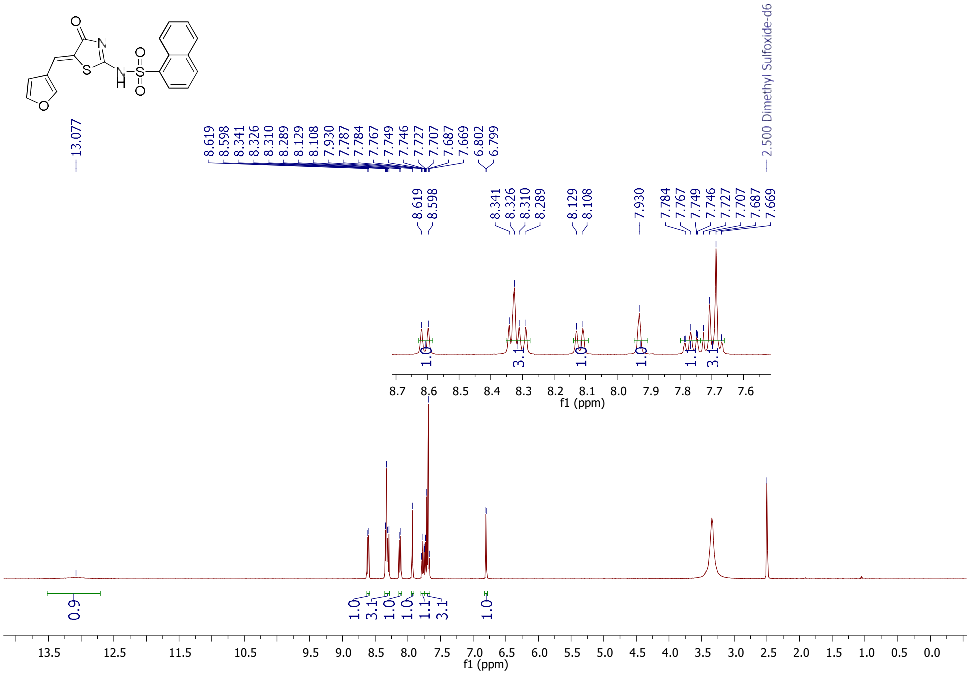

13.077
8.619 8.598 8.341 8.326 8.310 8.289 8.129 8.108 7.930 7.787 7.784 7.767 7.749 7.746 7.727 7.707 7.687 7.669 6.802 6.799
2.500 Dimethyl Sulfoxide-d6
f1 (ppm)

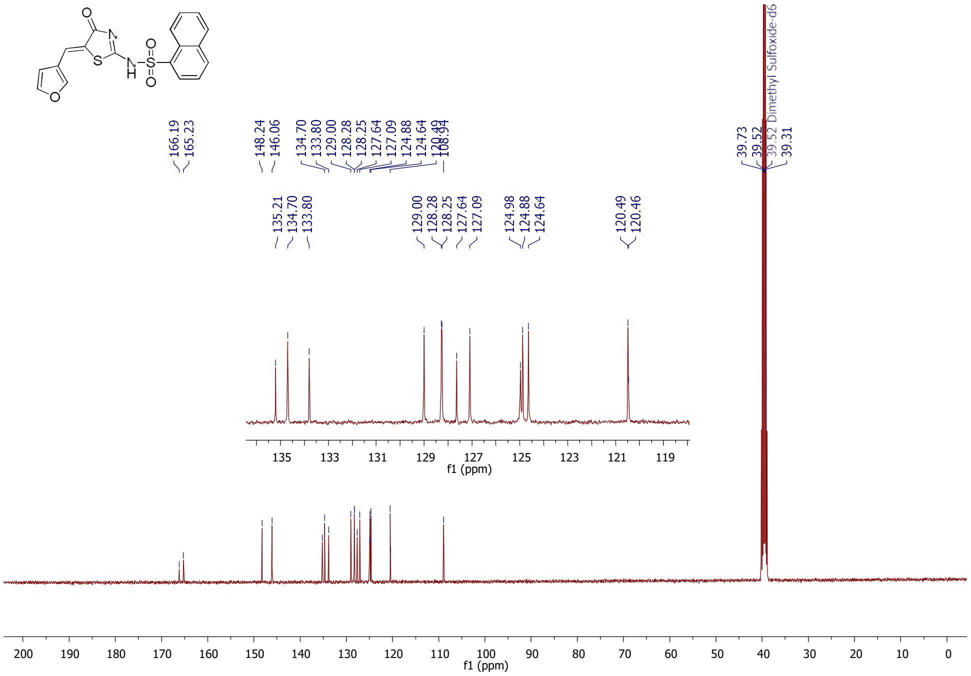

166.19 165.23
148.24 146.06
135.21 134.70 133.80 129.00 128.28 128.25 127.64 127.09 124.98 124.88 124.64 120.49 120.46 108.94
39.73 39.52 Dimethyl Sulfoxide-d6 39.52 39.31
f1 (ppm)

Agilent Technologies

Data file: D:\Chem32\1\Data\KP\KP609192 2019-08-14 15-23-30\002-54-KP6091.D
Sample name: KP6091
Description:
Sample amount: 0.000 **Sample type:** Sample

Instrument: LCMS **Location:** 54
Injection date: 8/14/2019 3:32:42 PM **Injection:** 1 of 1
Acq. method: LCMS ISOCRATIC 60% B 0.4MLMIN-1.M **Injection volume:** 2.000
Analysis method: LCMS ISOCRATIC 60%B 0.4MLMIN-1.M **Acq. operator:** SYSTEM
Last changed: 5/8/2019 8:55:04 AM

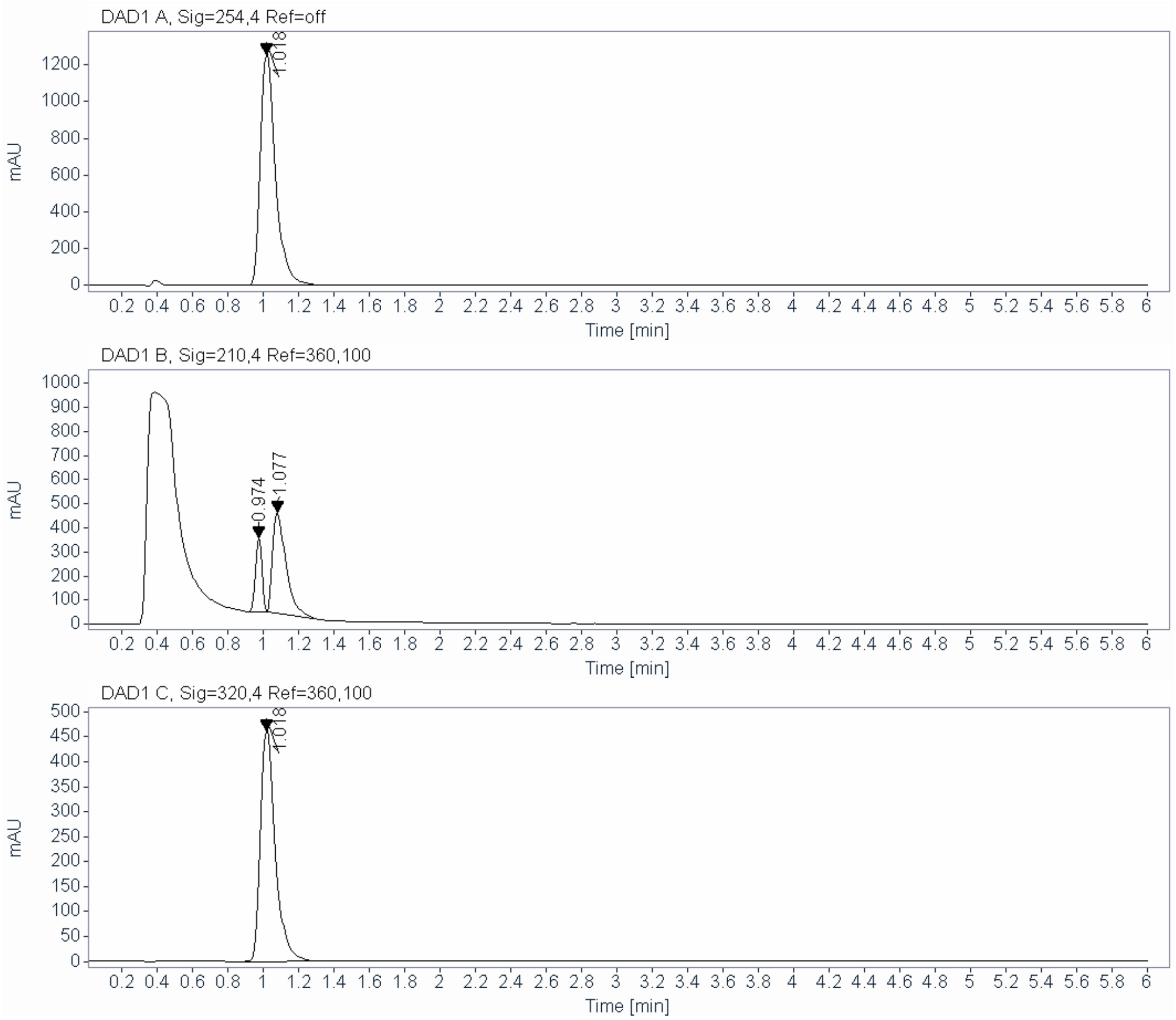

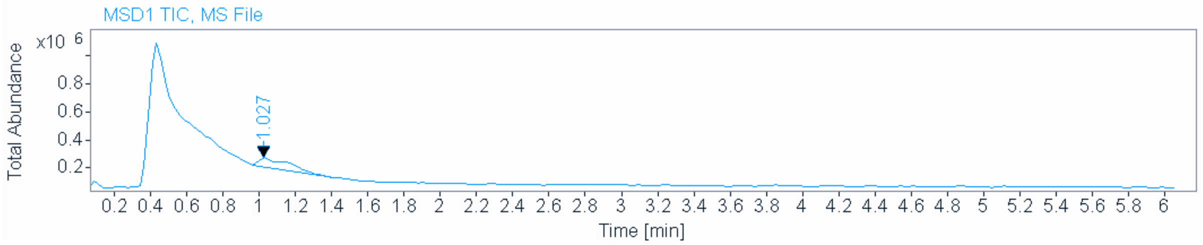

Agilent Technologies

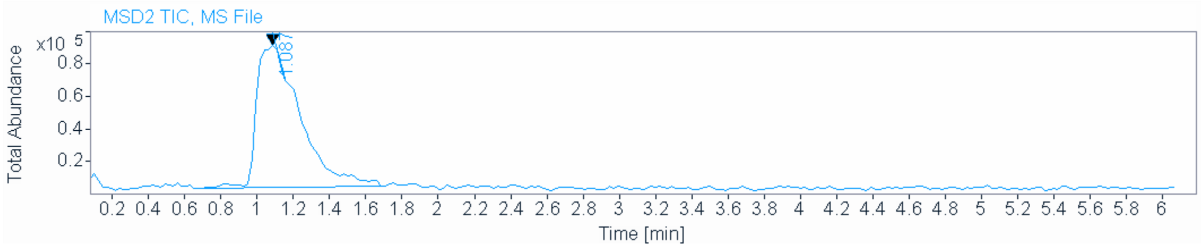

Signal: DAD1 A, Sig=254,4 Ref=off

RT [min]	Type	Width [min]	Area	Height	Area%	Name
1.018	BB	0.0889	7441.2383	1260.1158	100.0000	
		Sum	7441.2383			

Signal: DAD1 B, Sig=210,4 Ref=360,100

RT [min]	Type	Width [min]	Area	Height	Area%	Name
0.974	BB	0.0434	829.9445	308.7173	26.8451	
1.077	BB	0.0830	2261.6555	419.0422	73.1549	
		Sum	3091.6000			

Signal: DAD1 C, Sig=320,4 Ref=360,100

RT [min]	Type	Width [min]	Area	Height	Area%	Name
1.018	BB	0.0874	2684.6277	464.5733	100.0000	
		Sum	2684.6277			

Signal: MSD1 TIC, MS File

RT [min]	Type	Width [min]	Area	Height	Area%	Name
1.027	BB	0.1601	807897.3125	64391.6406	100.0000	
		Sum	807897.3125			

Signal: MSD2 TIC, MS File

RT [min]	Type	Width [min]	Area	Height	Area%	Name
1.087	BB	0.2359	1454853.875 0	87208.6172	100.0000	
		Sum	1454853.875			

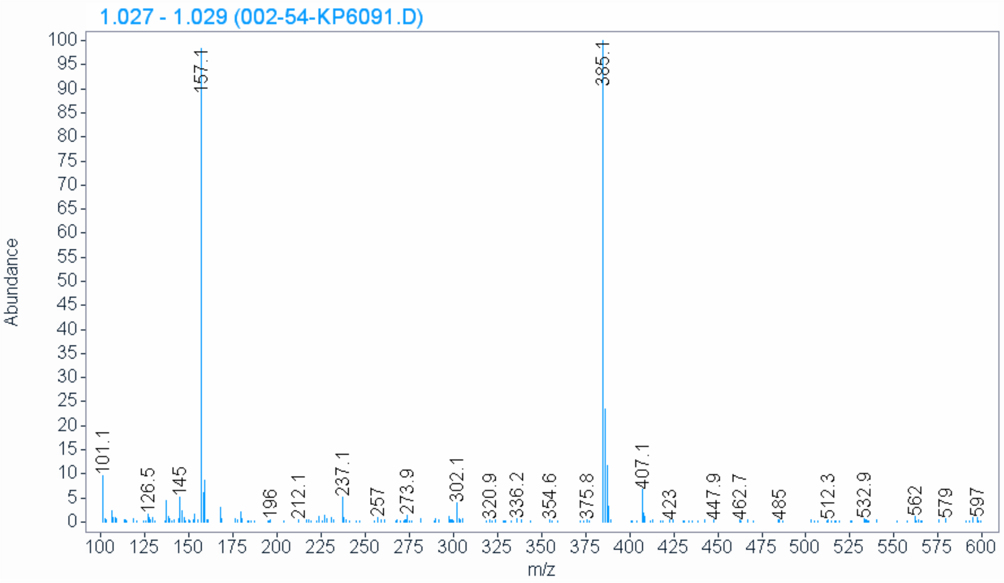
1.027 - 1.029 (002-54-KP6091.D)
Abundance
m/z
157.1
385.1
101.1
126.5
145
196
212.1
237.1
257
273.9
302.1
320.9
336.2
354.6
375.8
407.1
423
447.9
462.7
485
512.3
532.9
562
579
597

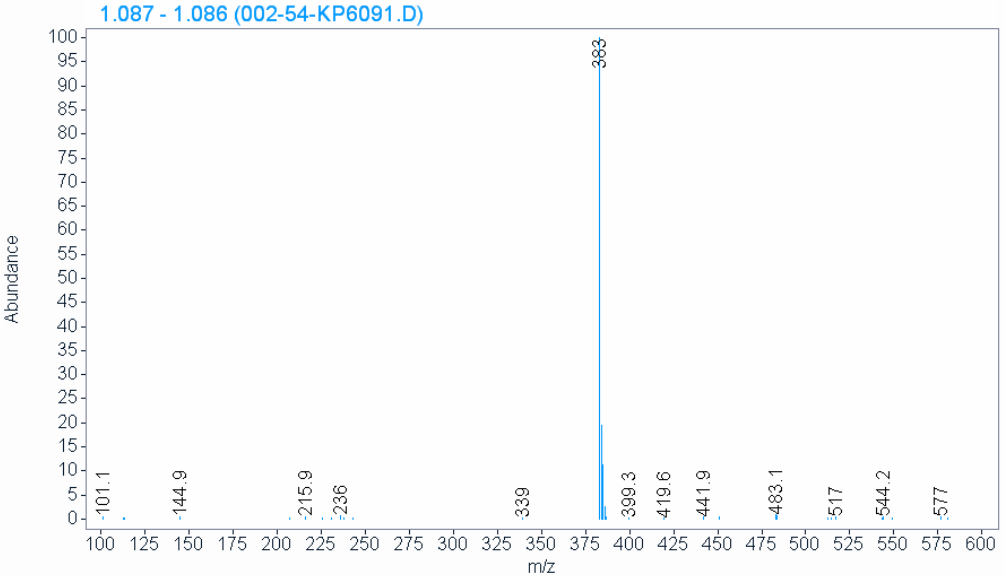
1.087 - 1.086 (002-54-KP6091.D)
Abundance
m/z
383
101.1
144.9
215.9
236
339
399.3
419.6
441.9
483.1
517
544.2
577

Compound Name: (Z)-*N*-(5-((1H-pyrrol-2-yl)methylene)-4-oxo-4,5-dihydrothiazol-2-yl)naphthalene-1-sulfonamide

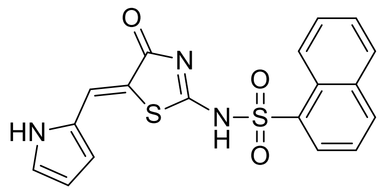

Chemical Formula: $C_{18}H_{13}N_3O_3S_2$
Exact Mass: 383.04
Molecular Weight: 383.44

Compound Code: 47 (KP6076)

Obtained Weight & Yield: 110 mg (60%)

Purity (by LCMS and ^{1}H NMR): > 99% (^{1}H NMR)

Appearance: dark orange solid

Solubility: DMSO slightly soluble in acetone and methanol

Melting Point: < 278 °C (dec.)

TLC Rf (and conditions): 0.50 (10% MeOH in DCM)

IR Analysis (including assignment): IR (neat): ν_{max} = 3266, 3112 (aromatic C-H), 1705 (C=O), 1557 (C-N), 1337 (sulfonamide), 1125 (N-H) cm^{-1}

^{1}H NMR Analysis: ^{1}H NMR (400 MHz, DMSO) δ 12.91 (br. s, 1H), 11.81 (s, 1H), 8.61 (d, *J* = 8.7 Hz, 1H), 8.33 – 8.28 (m, 2H), 8.11 (d, *J* = 8.0 Hz, 1H), 7.78 – 7.74 (m, 1H), 7.73 – 7.66 (m, 2H), 7.62 (s, 1H), 7.29 – 7.28 (m, 1H), 6.62 (s, 1H), 6.44 – 6.42 (m, 1H) ppm

^{13}C NMR Analysis: ^{13}C NMR (101 MHz, DMSO) δ 166.4, 165.6, 135.4, 134.6, 133.8, 129.0, 128.2, 128.1, 127.7, 127.1, 126.7, 125.6, 124.9, 124.6, 124.0, 114.7, 112.8, 112.5 ppm.

MS Analysis (low res): LRMS (ESI+) *m/z* (%): 384 (*M*+H, $C_{18}H_{14}N_3O_3S_2$, 100); (ESI-) *m/z* (%): 382 (*M*-H, $C_{18}H_{12}N_3O_3S_2$, 100)

HPLC method details: Column: Zorbax SB-C18 Rapid Resolution HT 2.1x50mm 1.8-Micron; Method LCMS ISOCRATIC 50%B 0.4MLMIN-1.M filename: KP6076; Peak retention time: 1.439 mins; Area (%): 98
NOTE: there is an additional peak at 2.465 mins – the same mass in both positive and negative.

Procedure: To a microwave vial was added the *N*-(4-oxo-4,5-dihydrothiazol-2-yl)naphthalene-1-sulfonamide (148 mg, 0.48 mmol), pyrrol-2-carboxaldehyde (52.3 mg, 0.55 mmol, 1.1 eq), the benzoic acid/piperidine catalyst (10 drops) and ethanol (3 mL). The reaction mixture was treated with microwave irradiation (120°C, 30 min) then cooled overnight. The precipitate was collected by vacuum filtration and washed with H_2O, cold ethanol and cold diethyl ether to give the desired product as a bright yellow solid (110 mg, 60%).

Other analyses, reference papers, previously obtained data, comments, etc:

Analyst	analyst1
Date	Thursday, 4 July 2019 12:29 PM

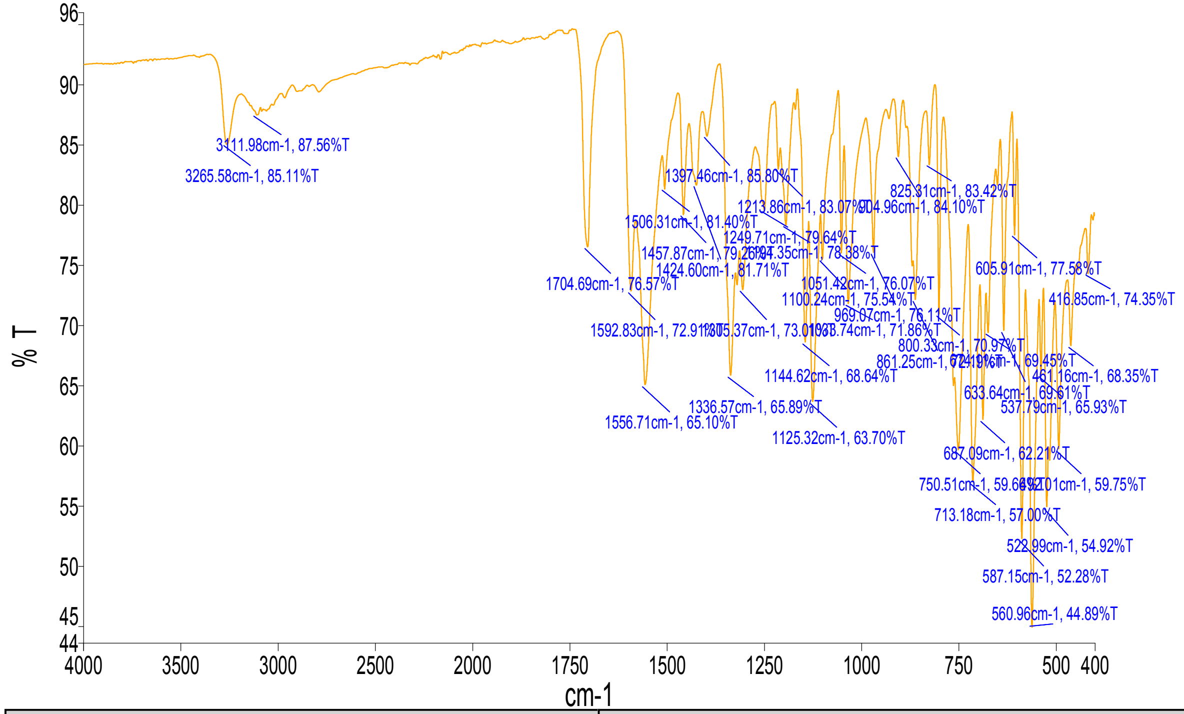

Sample Name	Description	Quality Checks
KP6076	Sample 008 By Analyst1 Date Thursday, July 04 2019	The Quality Checks do not report any warnings for the sample.

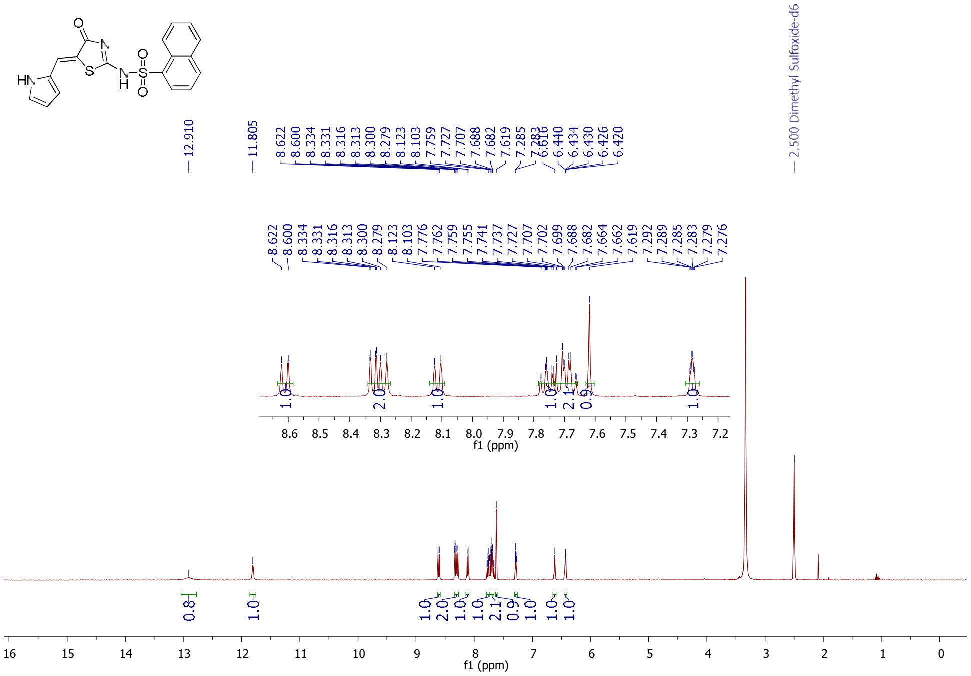
12.910
11.805
8.622 8.600 8.334 8.331 8.316 8.313 8.300 8.279 8.123 8.103 7.759 7.727 7.707 7.688 7.682 7.619 7.285 7.283 6.616 6.440 6.434 6.430 6.426 6.420
2.500 Dimethyl Sulfoxide-d6
8.622 8.600 8.334 8.331 8.316 8.313 8.300 8.279 8.123 8.103 7.776 7.762 7.759 7.755 7.741 7.737 7.727 7.707 7.702 7.699 7.688 7.682 7.664 7.662 7.619 7.292 7.289 7.285 7.283 7.279 7.276
1.0 2.0 1.0 1.0 2.1 0.9 1.0
f1 (ppm)
0.8 1.0 1.0 2.0 1.0 1.0 2.1 0.9 1.0 1.0 1.0
f1 (ppm)

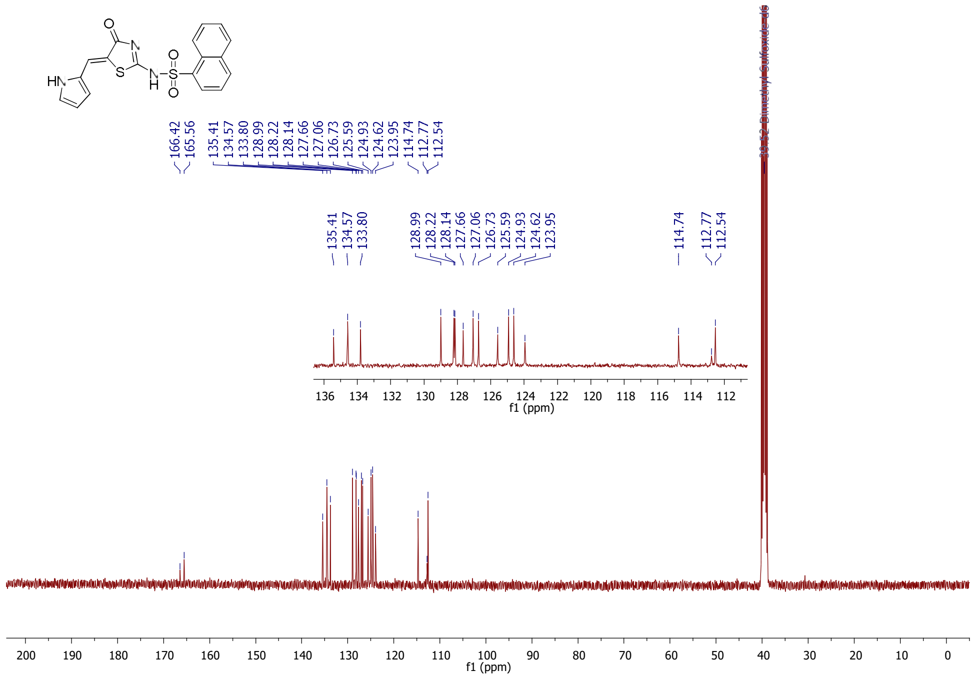
166.42 165.56
135.41 134.57 133.80 128.99 128.22 128.14 127.66 127.06 126.73 125.59 124.93 124.62 123.95
114.74 112.77 112.54
Dimethyl Sulfoxide-d6
f1 (ppm)
f1 (ppm)

Agilent Technologies

**Data file:**	D:\Chem32\1\Data\KP\KP6076_DSNEW2 2019-10-28 11-35-48\001-45-KP6076.D		
**Sample name:**	KP6076		
**Description:**			
**Sample amount:**	0.000	**Sample type:**	Sample
**Instrument:**	LCMS	**Location:**	45
**Injection date:**	10/28/2019 11:37:31 AM	**Injection:**	1 of 1
**Acq. method:**	LCMS ISOCRATIC 50% B.M_REDUCED FLOW.M	**Injection volume:**	2.000
**Analysis method:**	LCMS ISOCRATIC 50% B.M_REDUCED FLOW.M	**Acq. operator:**	SYSTEM
**Last changed:**	5/15/2019 9:20:00 AM		

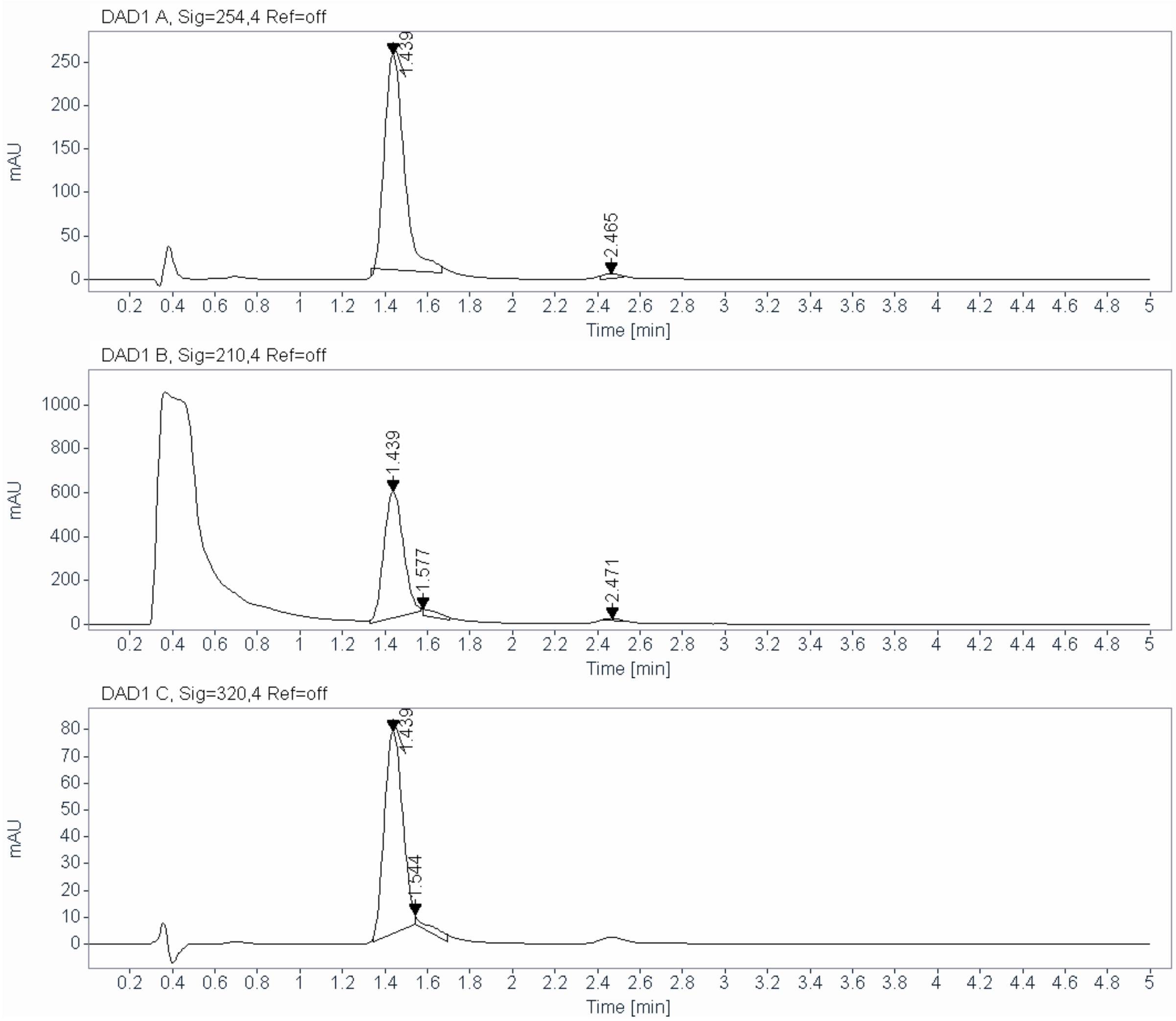

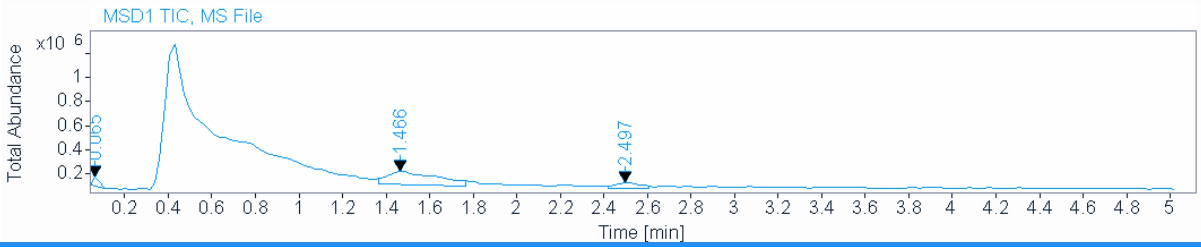

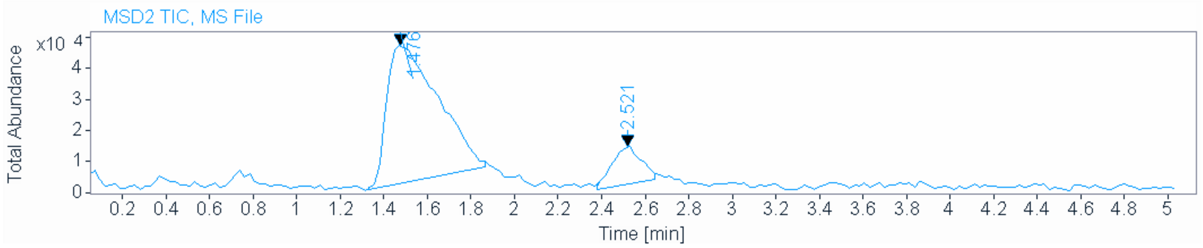

Signal: DAD1 A, Sig=254,4 Ref=off

RT [min]	Type	Width [min]	Area	Height	Area%	Name
1.439	MM	0.1076	1607.0616	248.9041	98.2834	
2.465	MM	0.0867	28.0690	5.3987	1.7166	
		Sum	1635.1307			

Signal: DAD1 B, Sig=210,4 Ref=off

RT [min]	Type	Width [min]	Area	Height	Area%	Name
1.439	MM	0.1057	3657.4958	576.9650	94.2654	
1.577	MM	0.0704	164.4961	29.0951	4.2396	
2.471	MM	0.0802	58.0081	12.0507	1.4951	
		Sum	3880.0000			

Signal: DAD1 C, Sig=320,4 Ref=off

RT [min]	Type	Width [min]	Area	Height	Area%	Name
1.439	MM	0.0989	450.2690	75.8440	95.7000	
1.544	MM	0.0806	20.2313	3.5674	4.3000	
		Sum	470.5003			

Signal: MSD1 TIC, MS File

RT [min]	Type	Width [min]	Area	Height	Area%	Name
0.065	BB	0.0355	172113.9063	80883.6406	7.2694	
1.466	MM	0.2643	1778179.2500	112140.2266	75.1028	
2.497	MM	0.1380	417366.9063	50401.9883	17.6278	
		Sum	2367660.062			

Signal: MSD2 TIC, MS File

RT [min]	Type	Width [min]	Area	Height	Area%	Name
1.476	MM	0.2692	719552.9375	44542.9961	86.2031	
2.521	MM	0.1525	115165.5781	12584.3252	13.7969	
		Sum	834718.5156			

Agilent Technologies

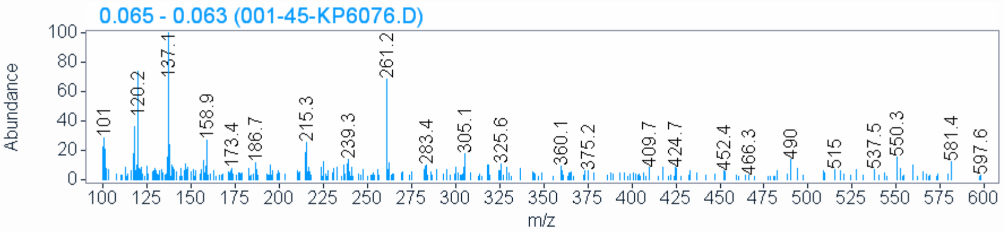

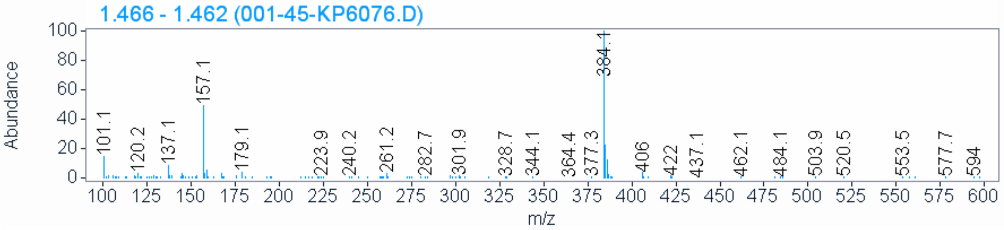

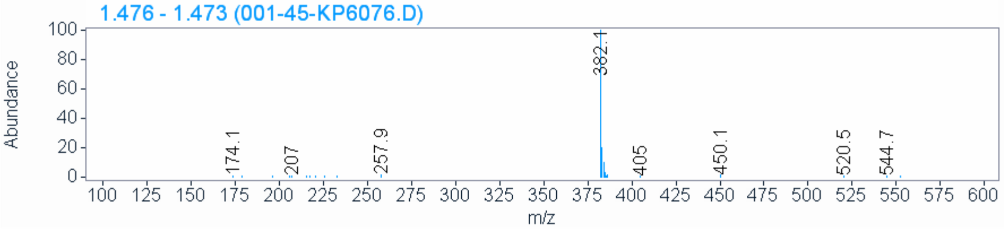

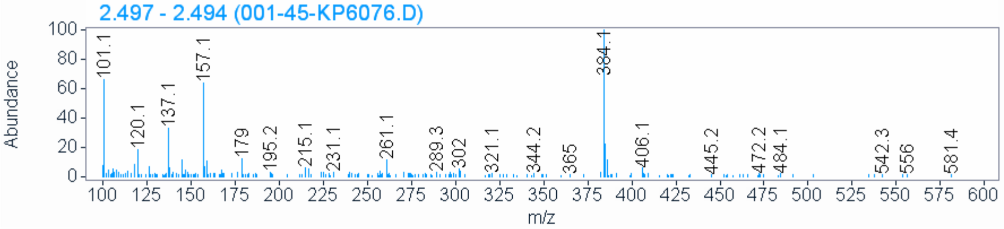

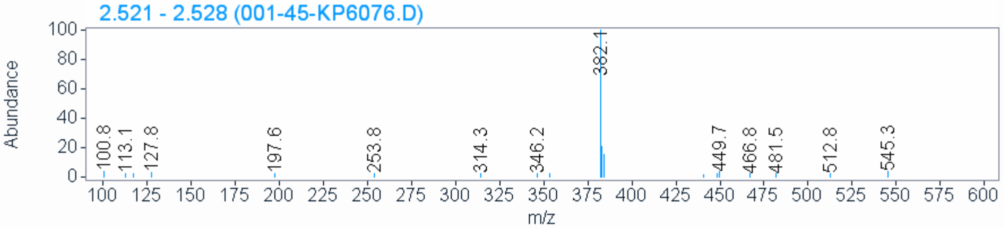

Compound Name: (Z)-*N*-(5-(furan-2-ylmethylene)-4-oxo-4,5-dihydrothiazol-2-yl)naphthalene-1-sulfonamide

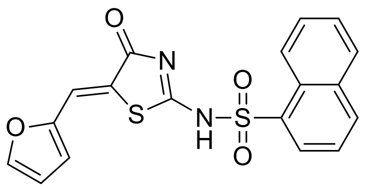

Chemical Formula: $C_{18}H_{12}N_2O_4S_2$
Exact Mass: 384.02
Molecular Weight: 384.43

Compound Code: 48 (KP6078)

Obtained Weight & Yield: 103 mg (55%)

Purity (by LCMS and ^{1}H NMR): > 99% (^{1}H NMR)

Appearance: Pale brown solid

Solubility: DMSO, slightly soluble in methanol

Melting Point: < 249 °C (dec.)

TLC Rf (and conditions): 0.35 (10% MeOH in DCM)

IR Analysis (including assignment): IR (neat): ν_{max} = 3132, 3029 (aromatic C-H), 1699 (C=O), 1608 (C=C), 1543 (N-H), 1333 (sulfonamide), 1158 (C-O), 1129 (N-C) cm^{-1}

^{1}H NMR Analysis: ^{1}H NMR (400 MHz, DMSO) δ 13.03 (s, 1H, br), 8.60 (d, *J* = 8.7 Hz, 1H), 8.31 – 8.27 (m, 2H), 8.19 (d, *J* = 1.7 Hz, 1H), 8.12 (d, *J* = 8.1 Hz, 1H), 7.79 – 7.74 (m, 1H), 7.73 – 7.67 (m, 2H), 7.61 (s, 1H), 7.18 (d, *J* = 3.5 Hz, 1H), 6.80 (dd, *J* = 3.5, 1.7 Hz, 1H) ppm.
Ethanol impurity at 1.05 ppm (0.64%)

^{13}C NMR Analysis: ^{13}C NMR (101 MHz, DMSO) δ 166.6, 166.4, 149.2, 148.2, 135.4, 134.6, 133.8, 129.0, 128.3, 127.9, 127.6, 127.1, 124.9, 124.6, 120.0, 119.9, 118.4, 113.8 ppm.

MS Analysis (low res): LRMS (ESI+) *m/z* (%): 385 (*M*+H, $C_{18}H_{13}N_2O_4S_2$, 90%): (ESI-) *m/z* (%): 383 (*M*-H, $C_{18}H_{11}N_2O_4S_2$, 100%),

MS Analysis (high res): Insert data when obtained. Exact mass calculated for $C_xH_xN_x$ [*M*+H]$^+$, XXX.XXXX. Found XXX.XXXX.

HPLC method details: Column: Zorbax SB-C18 Rapid Resolution HT 2.1x50mm 1.8-Micron; Method: LCMS ISOCRATIC 50%B 0.4MLMIN-1.M filename: KP6078; Peak retention time: 1.812 mins; Area (%): 99.

Procedure: To a microwave vial was added the *N*-(4-oxo-4,5-dihydrothiazol-2-yl)naphthalene-1-sulfonamide (149 mg, 0.49 mmol), 2-furaldehyde (0.05 mL, 0.54 mmol, 1.1 eq), the benzoic acid/piperidine catalyst (3 drops) and ethanol (3 mL). The reaction mixture was treated with microwave irradiation (120°C, 30 min). After cooling a precipitate was collected by vacuum filtration. The precipitate was washed with H_2O (2 mL), cold ethanol (5 mL) and cold diethyl ether (10 mL) to give the desired product as a pale brown solid (103 mg, 55%).

Other analyses, reference papers, previously obtained data, comments, etc:

Analyst	analyst1
Date	Thursday, 4 July 2019 12:32 PM

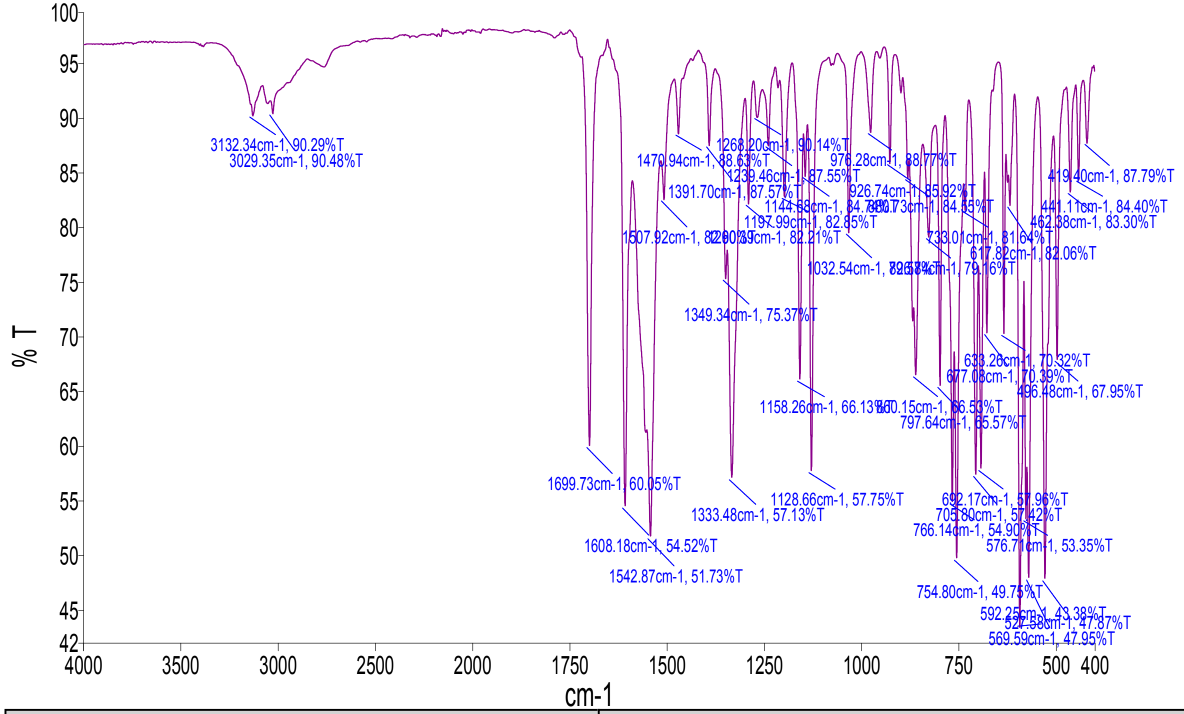

Sample Name	Description	Quality Checks
KP6078	Sample 009 By Analyst1 Date Thursday, July 04 2019	The Quality Checks do not report any warnings for the sample.

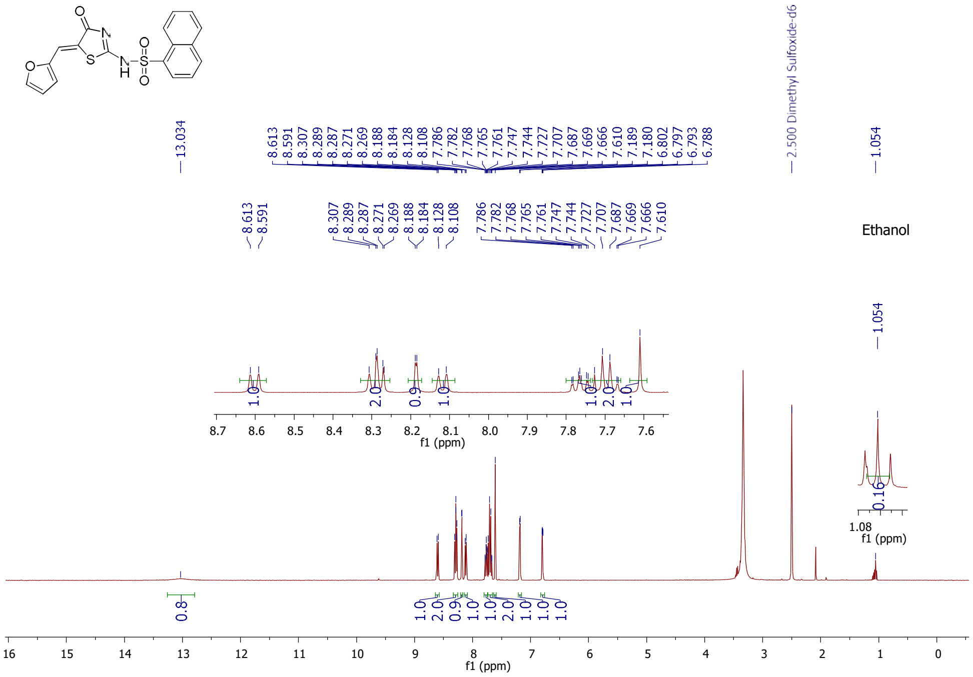

13.034
8.613 8.591 8.307 8.289 8.287 8.271 8.269 8.188 8.184 8.128 8.108 7.786 7.782 7.768 7.765 7.761 7.747 7.744 7.727 7.707 7.687 7.669 7.666 7.610 7.189 7.180 6.802 6.797 6.793 6.788
2.500 Dimethyl Sulfoxide-d6
1.054
Ethanol
f1 (ppm)

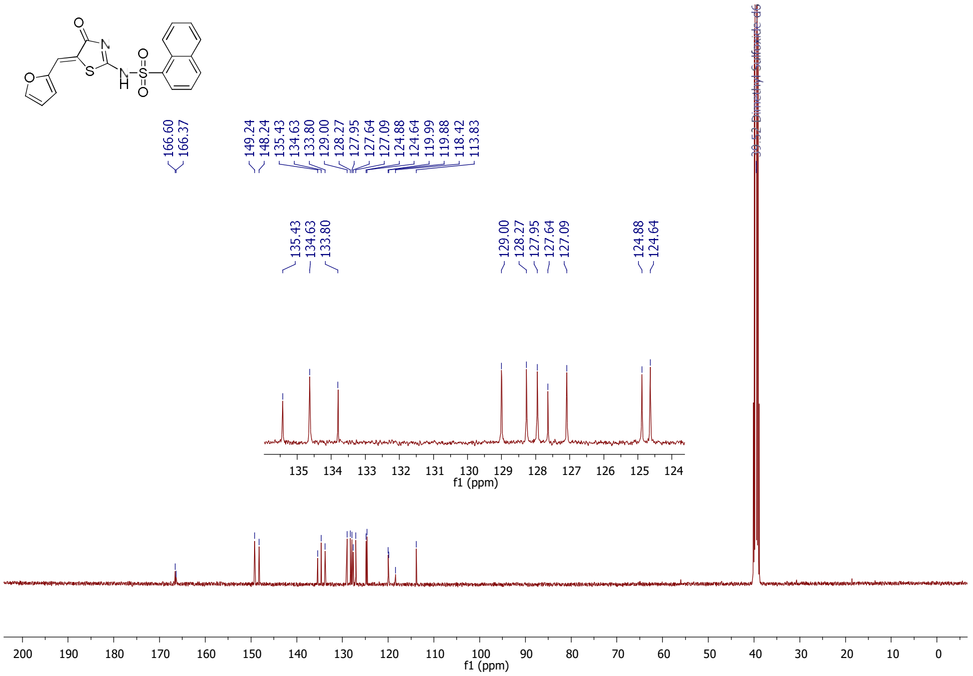

166.60 166.37
149.24 148.24 135.43 134.63 133.80 129.00 128.27 127.95 127.64 127.09 124.88 124.64 119.99 119.88 118.42 113.83
Dimethyl Sulfoxide-d6
f1 (ppm)

LCMS Report

Agilent Technologies

**Data file:**	D:\Chem32\1\Data\KP\KP6076KP6078_dsNEW 2019-10-28 09-42-33\003-46-KP6078.D		
**Sample name:**	KP6078		
**Description:**			
**Sample amount:**	0.000	**Sample type:**	Sample
**Instrument:**	LCMS	**Location:**	46
**Injection date:**	10/28/2019 9:58:29 AM	**Injection:**	1 of 1
**Acq. method:**	LCMS ISOCRATIC 50% B.M_REDUCED FLOW.M	**Injection volume:**	2.000
**Analysis method:**	LCMS ISOCRATIC 50% B.M_REDUCED FLOW.M	**Acq. operator:**	SYSTEM
**Last changed:**	5/15/2019 9:20:00 AM		

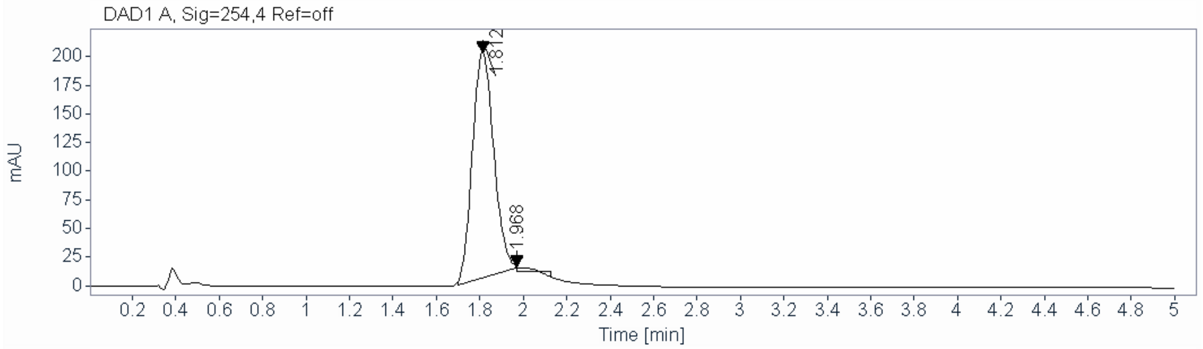

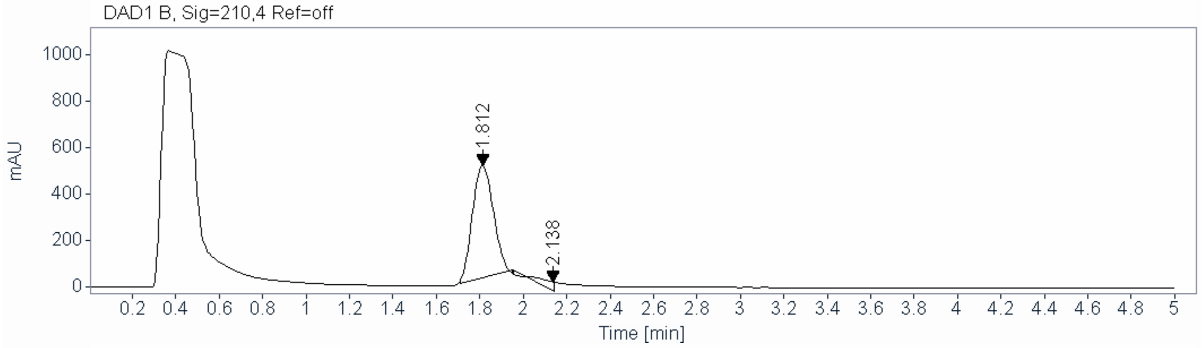

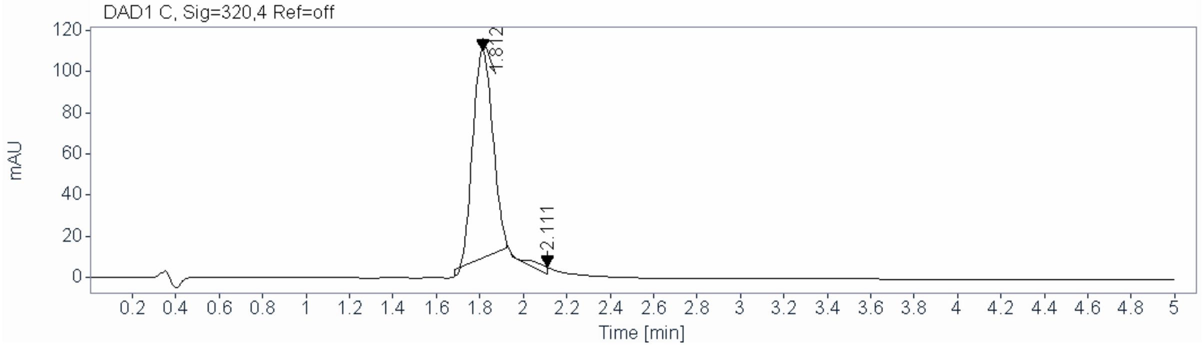

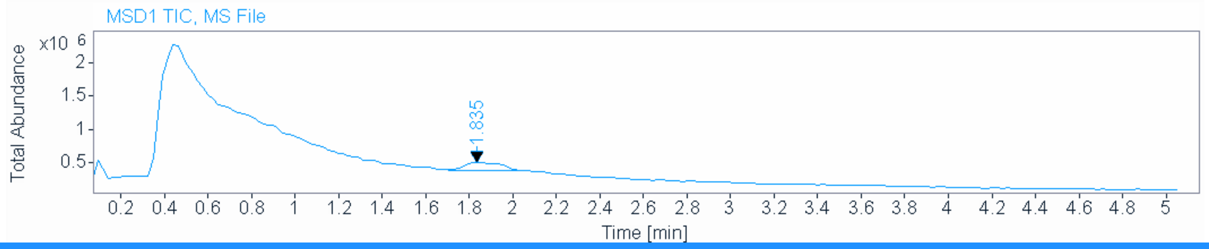

Agilent Technologies

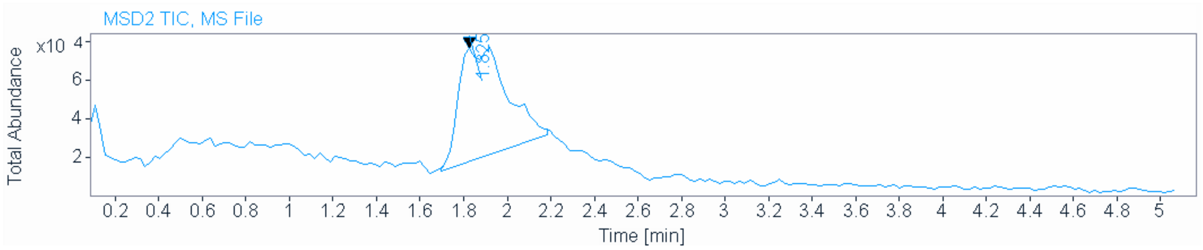

Signal: DAD1 A, Sig=254,4 Ref=off

RT [min]	Type	Width [min]	Area	Height	Area%	Name
1.812	MM	0.1138	1345.9240	197.0372	98.8724	
1.968	MM	0.0500	15.3497	4.4069	1.1276	
		Sum	1361.2736			

Signal: DAD1 B, Sig=210,4 Ref=off

RT [min]	Type	Width [min]	Area	Height	Area%	Name
1.812	MM	0.1113	3233.2178	484.2895	94.4501	
2.138	MM	0.0794	189.9832	39.8919	5.5499	
		Sum	3423.2010			

Signal: DAD1 C, Sig=320,4 Ref=off

RT [min]	Type	Width [min]	Area	Height	Area%	Name
1.812	MM	0.1067	649.2352	101.3890	97.1002	
2.111	MM	0.1030	19.3887	3.1385	2.8998	
		Sum	668.6239			

Signal: MSD1 TIC, MS File

RT [min]	Type	Width [min]	Area	Height	Area%	Name
1.835	MM	0.1973	1515355.7500	128028.8047	100.0000	
		Sum	1515355.750			

Signal: MSD2 TIC, MS File

RT [min]	Type	Width [min]	Area	Height	Area%	Name
1.825	MM	0.2445	869894.8125	59307.3008	100.0000	
		Sum	869894.8125			

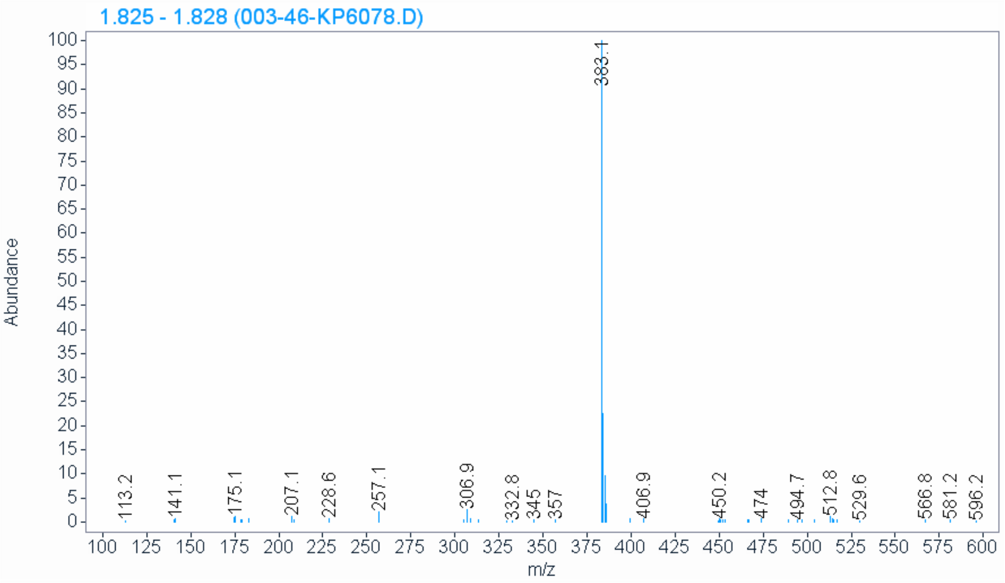
1.825 - 1.828 (003-46-KP6078.D)
Abundance
m/z
383.1
113.2
141.1
175.1
207.1
228.6
257.1
306.9
332.8
345
357
406.9
450.2
474
494.7
512.8
529.6
566.8
581.2
596.2

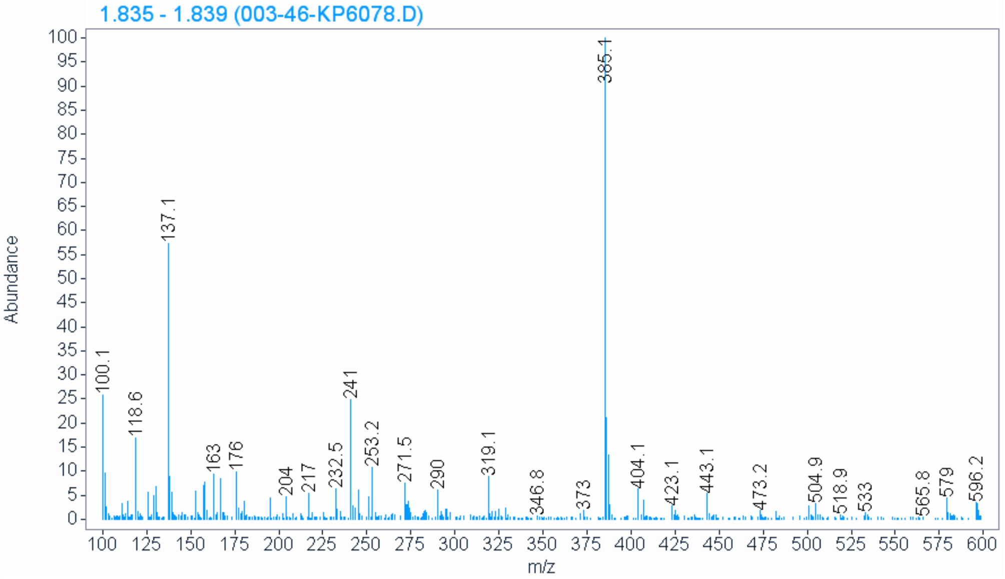
1.835 - 1.839 (003-46-KP6078.D)
Abundance
m/z
385.1
137.1
100.1
118.6
241
163
176
204
217
232.5
253.2
271.5
290
319.1
346.8
373
404.1
423.1
443.1
473.2
504.9
518.9
533
565.8
579
596.2

Compound Name: (Z)-*N*-(5-((5-chlorofuran-2-yl)methylene)-4-oxo-4,5-dihydrothiazol-2-yl)naphthalene-1-sulfonamide

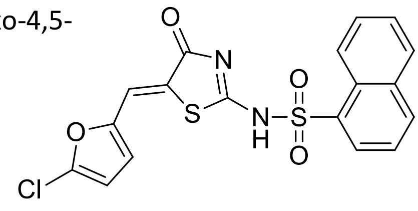

Chemical Formula: $C_{18}H_{11}ClN_2O_4S_2$
Exact Mass: 417.98
Molecular Weight: 418.87

Compound Code: 49 (KP6080)

Obtained Weight & Yield: 139 mg (68%)

Purity (by LCMS and ^{1}H NMR): > 96% by ^{1}H-NMR

Appearance: deep red solid

Solubility: DMSO, slightly soluble in acetone and methanol

Melting Point: < 240 °C (dec.)

TLC Rf (and conditions): 0.48 (10% MeOH in DCM)

IR Analysis (including assignment): IR (neat): ν_{max} = 3137 (N-H), 3034, 2940 (aromatic C-H), 1698 (C=O), 1606 (alkene C=C), 1545 (N-H), 1316 (sulfonamide), 1127 (C-N), 874 (C-Cl) cm^{-1}

^{1}H NMR Analysis: ^{1}H NMR (400 MHz, DMSO) δ 13.08 (s, br, 1H), 8.60 (d, *J* = 8.5 Hz, 1H), 8.31 – 8.28 (m, 2H), 8.12 (d, *J* = 8.1 Hz, 1H), 7.79 – 7.75 (m, 1H), 7.73 – 7.67 (m, 2H), 7.53 (s, 1H), 7.23 (d, *J* = 3.6 Hz, 1H), 6.83 (d, *J* = 3.6 Hz, 1H) ppm.
Ethanol impurity at 1.05 ppm (3.84%)

^{13}C NMR Analysis: ^{13}C NMR (101 MHz, DMSO) δ 166.5, 166.2, 149.2, 140.1, 135.4, 134.7, 133.8, 129.0, 128.3, 128.1, 127.6, 127.1, 124.9, 124.6, 121.6, 119.4, 118.6, 111.2 ppm.

MS Analysis (low res): LRMS (ESI+) *m/z*: 419 (*M*+H, $C_{18}H_{12}{}^{35}ClN_2O_4S_2$, 100%), 421 (*M*+H, $C_{18}H_{12}{}^{37}ClN_2O_4S_2$, 45); (ESI-) *m/z*: 417 (*M*-H, $C_{18}H_{10}{}^{35}ClN_2O_4S_2$, 100%), 419 (*M*-H, $C_{18}H_{10}{}^{37}ClN_2O_4S_2$, 45)

HPLC method details: Column: Zorbax SB-C18 Rapid Resolution HT 2.1x50mm 1.8-Micron; Method: LCMS ISOCRATIC 60%B 0.4MLMIN-1.M filename: KP6080; Peak retention time: 1.38 mins; Area (%): 100.

Procedure: To a microwave vial was added the *N*-(4-oxo-4,5-dihydrothiazol-2-yl)naphthalene-1-sulfonamide (151 mg, 0.49 mmol), 5-chloro-2-furaldehyde (73 mg, 0.56 mmol, 1.1 eq), ethanol (3 mL) and the benzoic acid/piperidine catalyst (3 drops). The reaction mixture was treated with microwave irradiation (120 °C, 30 min). After cooling a precipitate was collected by vacuum filtration. The precipitate was washed with H_2O (2 mL), cold ethanol (5 mL) and cold diethyl ether (10 mL) to give the desired product as a red solid (139 mg, 68%).

Other analyses, reference papers, previously obtained data, comments, etc:

Analyst	analyst1
Date	Thursday, 4 July 2019 12:35 PM

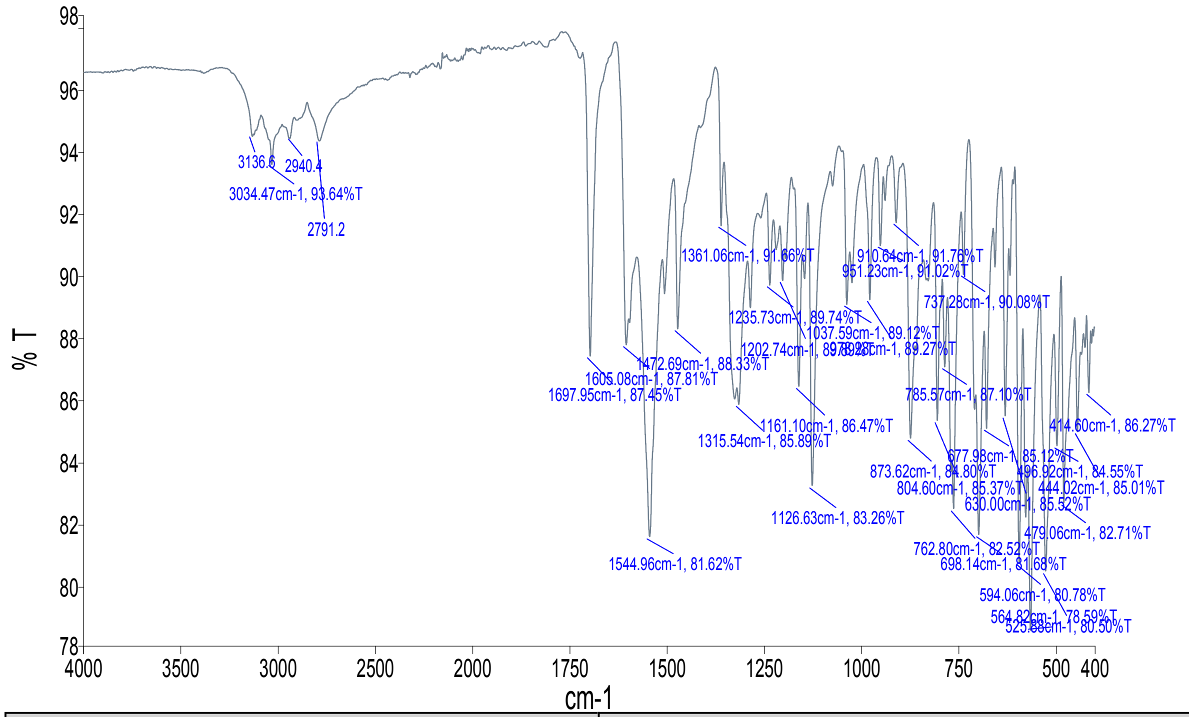

Sample Name	Description	Quality Checks
KP6080	Sample 010 By Analyst1 Date Thursday, July 04 2019	The Quality Checks give rise to a Weak Bands warning for the sample.

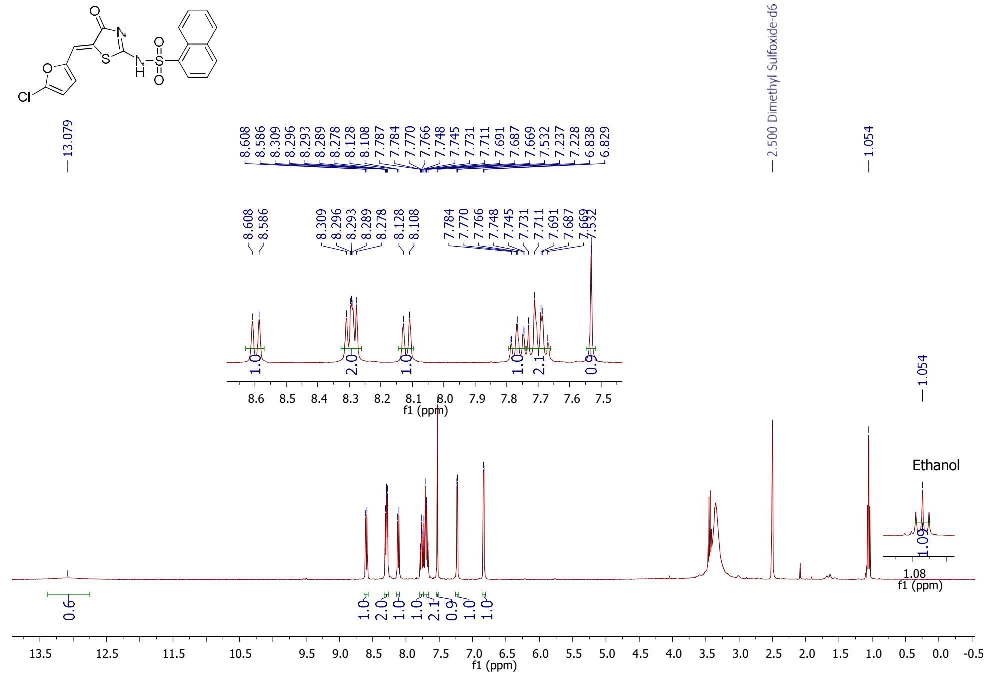
O
N
S
N
H
S
O
O
O
Cl
13.079
8.608 8.586 8.309 8.296 8.293 8.289 8.278 8.128 8.108 7.787 7.784 7.770 7.766 7.748 7.745 7.731 7.711 7.691 7.687 7.669 7.532 7.237 7.228 6.838 6.829
2.500 Dimethyl Sulfoxide-d6
1.054
Ethanol
1.09
1.08
f1 (ppm)
0.6
1.0 2.0 1.0 1.0 2.1 0.9 1.0 1.0
f1 (ppm)

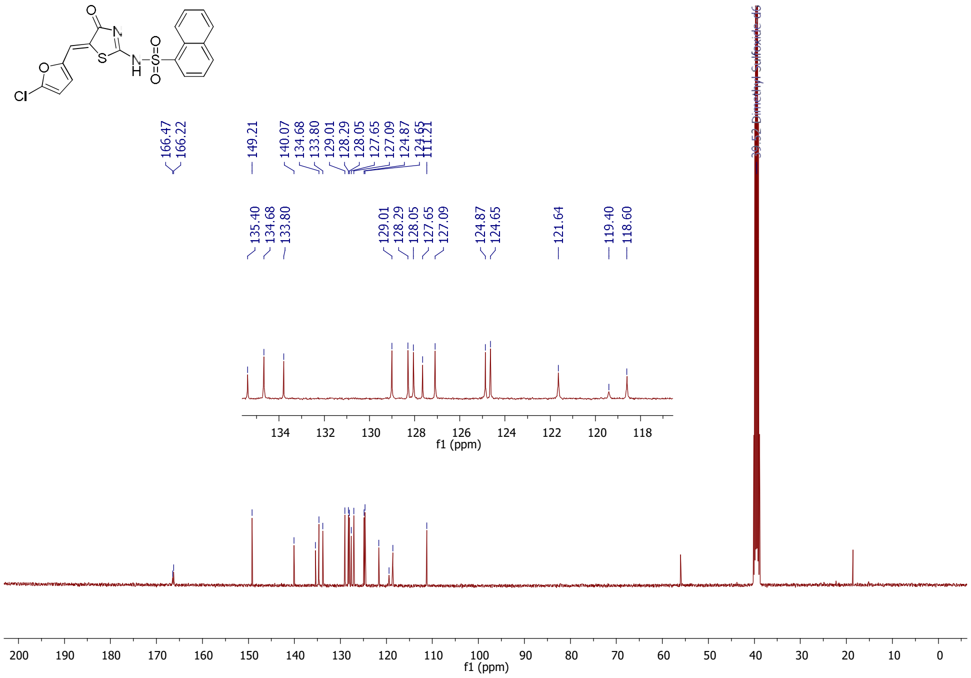
O
N
S
N
H
S
O
O
O
Cl
166.47
166.22
149.21
140.07 134.68 133.80 129.01 128.29 128.05 127.65 127.09 124.87 124.65 111.21
135.40 134.68 133.80
129.01 128.29 128.05 127.65 127.09
124.87 124.65
121.64
119.40 118.60
f1 (ppm)
f1 (ppm)

LCMS Report

Agilent Technologies

Data file: D:\Chem32\1\Data\KP\KP60768082 2019-08-13 12-36-15\003-45-KP6080.D
Sample name: KP6080
Description:
Sample amount: 0.000
Sample type: Sample

Instrument: LCMS
Location: 45
Injection date: 8/13/2019 12:54:26 PM
Injection: 1 of 1
Acq. method: LCMS ISOCRATIC 60% B 0.4MLMIN-1.M
Injection volume: 2.000
Analysis method: LCMS ISOCRATIC 60%B 0.4MLMIN-1.M
Acq. operator: SYSTEM
Last changed: 5/8/2019 8:55:04 AM

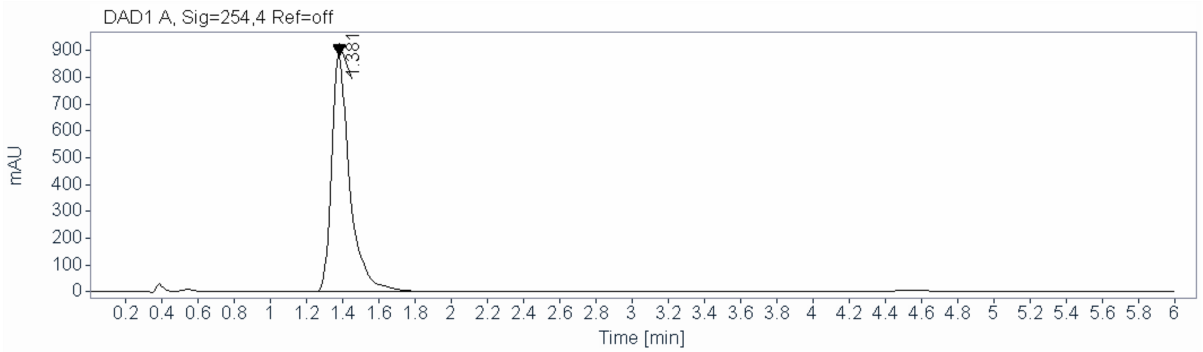

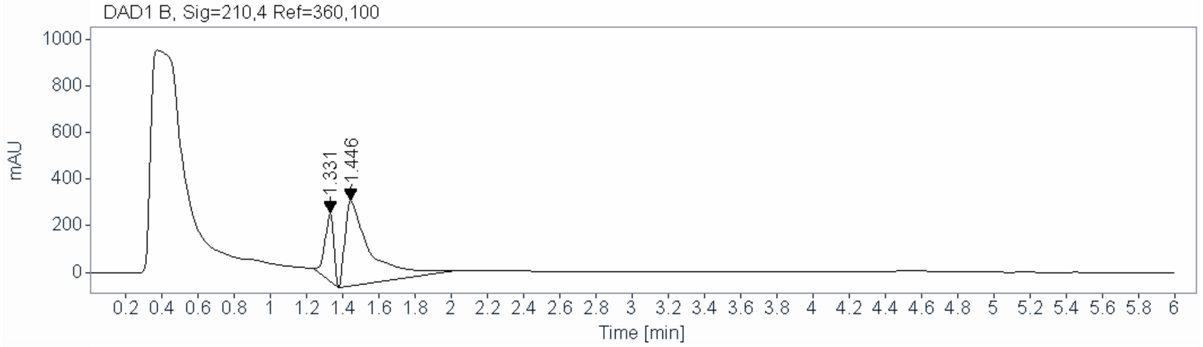

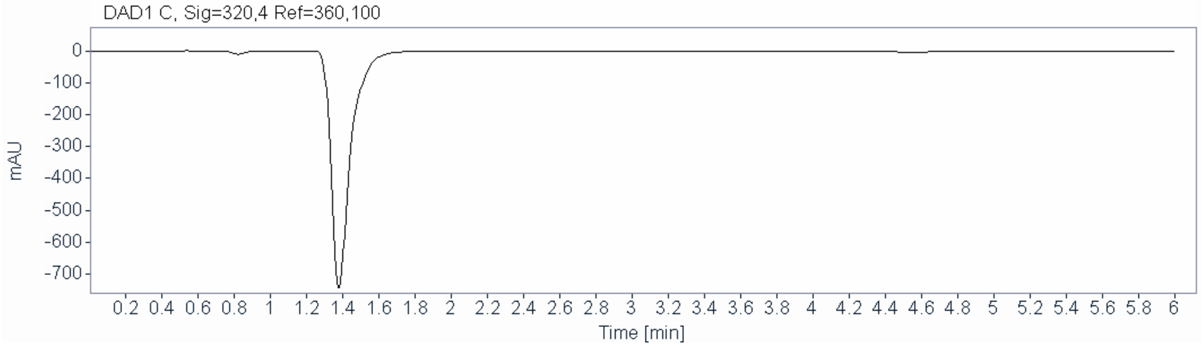

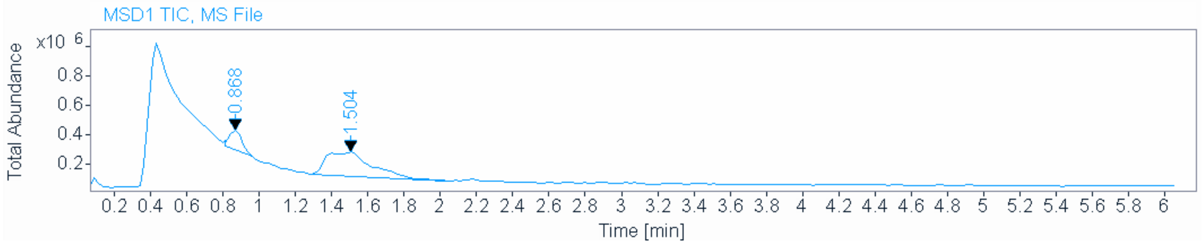

Agilent Technologies

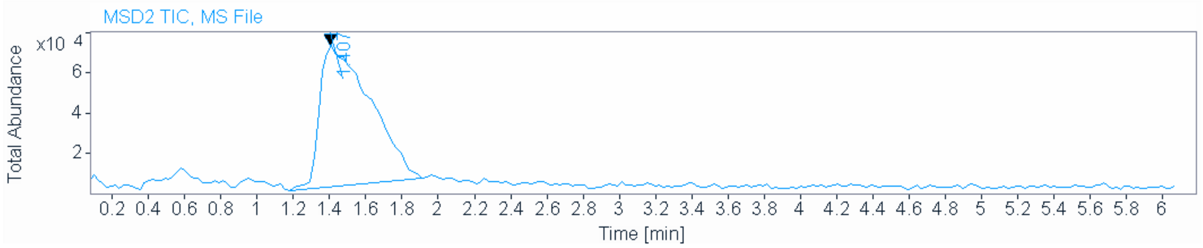

Signal: DAD1 A, Sig=254,4 Ref=off

RT [min]	Type	Width [min]	Area	Height	Area%	Name
1.381	BB	0.1038	6195.9009	883.4984	100.0000	
		Sum	6195.9009			

Signal: DAD1 B, Sig=210,4 Ref=360,100

RT [min]	Type	Width [min]	Area	Height	Area%	Name
1.331	BB	0.0528	975.3879	293.1342	21.4422	
1.446	BB	0.1411	3573.5239	368.7129	78.5578	
		Sum	4548.9119			

Signal: MSD1 TIC, MS File

RT [min]	Type	Width [min]	Area	Height	Area%	Name
0.868	MM	0.0777	607926.1250	130437.0859	17.7517	
1.504	BB	0.2229	2816675.750 0	162436.9688	82.2483	
		Sum	3424601.875			

Signal: MSD2 TIC, MS File

RT [min]	Type	Width [min]	Area	Height	Area%	Name
1.407	BB	0.2534	1358986.125 0	70201.5781	100.0000	
		Sum	1358986.125			

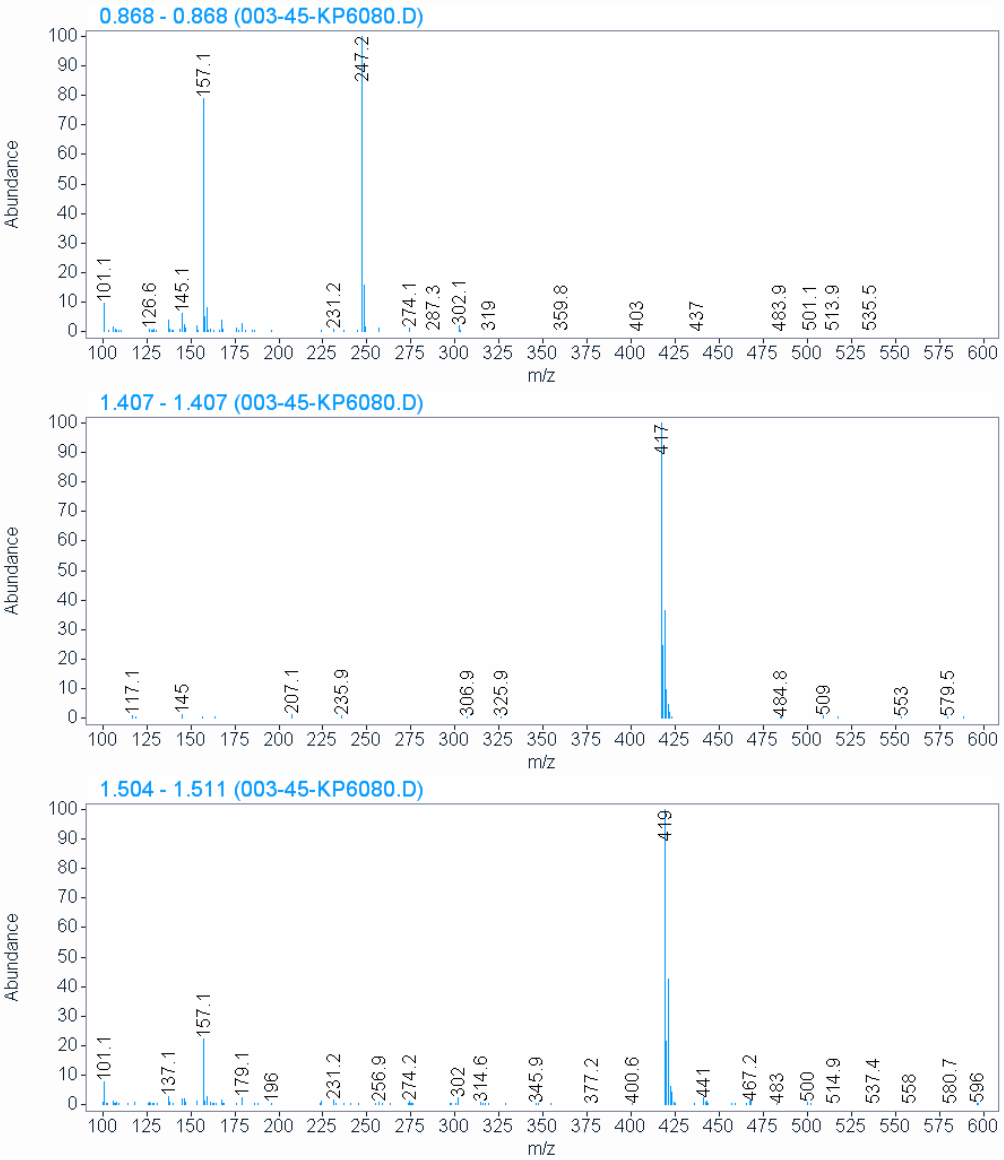
0.868 - 0.868 (003-45-KP6080.D)
Abundance
m/z
101.1
126.6
145.1
157.1
231.2
247.2
274.1
287.3
302.1
319
359.8
403
437
483.9
501.1
513.9
535.5
1.407 - 1.407 (003-45-KP6080.D)
117.1
145
207.1
235.9
306.9
325.9
417
484.8
509
553
579.5
1.504 - 1.511 (003-45-KP6080.D)
101.1
137.1
157.1
179.1
196
231.2
256.9
274.2
302
314.6
345.9
377.2
400.6
419
441
467.2
483
500
514.9
537.4
558
580.7
596

Compound Name: (Z)-*N*-(5-((5-bromofuran-2-yl)methylene)-4-oxo-4,5-dihydrothiazol-2-yl)naphthalene-1-sulfonamide

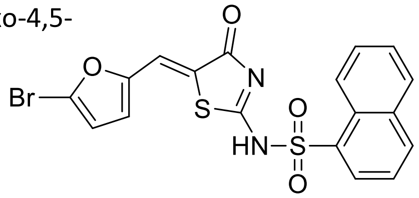

Chemical Formula: $C_{18}H_{11}BrN_2O_4S_2$
Exact Mass: 461.93
Molecular Weight: 463.32

Compound Code: 50 (KP6083)

Obtained Weight & Yield: 183 mg (82%)

Purity (by LCMS and ^{1}H NMR): >99% (^{1}H NMR)

Appearance: Bright red solid

Solubility: DMSO, slightly soluble in acetone and methanol

Melting Point: < 243 °C (dec.)

TLC Rf (and conditions): 0.46 (10% MeOH in DCM)

IR Analysis (including assignment): IR (neat): ν_{max} = 3133, 3034 (aromatic C-H), 1695 (C=O), 1542 (N-H), 1314 (sulfonamide), 1125 (C-N), 594 (C-Br) cm^{-1}

^{1}H NMR Analysis: ^{1}H NMR (400 MHz, DMSO) δ 13.07 (br. s, 1H), 8.59 (d, *J* = 8.6 Hz, 1H), 8.32 – 8.28 (m, 2H), 8.12 (d, *J* = 8.1 Hz, 1H), 7.79 – 7.75 (m, 1H), 7.74 – 7.67 (m, 2H), 7.54 (s, 1H), 7.19 (d, *J* = 3.6 Hz, 1H), 6.93 (d, *J* = 3.6 Hz, 1H) ppm.
Ethanol at 1.05 ppm (0.66%)

^{13}C NMR Analysis: ^{13}C NMR (101 MHz, DMSO) δ 166.3, 166.1, 151.3, 135.3, 134.7, 133.8, 129.0, 128.3, 128.1, 127.9, 127.6, 127.1, 124.8, 124.6, 121.8, 119.3, 118.6, 116.0 ppm.

MS Analysis (low res): LRMS (ESI+) *m/z*: 463 (*M*+H, $C_{18}H_{12}{}^{79}BrN_2O_4S_2$, 90%), 465 (*M*+H, $C_{18}H_{12}{}^{81}BrN_2O_4S_2$, 100%); (ESI-) *m/z*: 461 (*M*-H, $C_{18}H_{10}{}^{79}BrN_2O_4S_2$, 100%), 463 (*M*-H, $C_{18}H_{10}{}^{81}BrN_2O_4S_2$, 100%)

HPLC method details: Column: Zorbax SB-C18 Rapid Resolution HT 2.1x50mm 1.8-Micron; Method LCMS ISOCRATIC 60%B 0.4MLMIN-1.M filename: KP6083; Peak retention time: 1.45 mins; Area (%): 100.

Procedure: To a microwave vial was added the *N*-(4-oxo-4,5-dihydrothiazol-2-yl)naphthalene-1-sulfonamide (147 mg, 0.48 mmol), 5-bromo-2-furaldehyde (105 mg, 0.60 mmol, 1.3 eq), the benzoic acid/piperidine catalyst (3 drops) and ethanol (3 mL). The reaction mixture was treated with microwave irradiation (120°C, 30 min). After cooling a precipitate was collected by vacuum filtration. The precipitate was washed with H_2O (2 mL), cold ethanol (5 mL) and cold diethyl ether (10 mL) to give the desired product as a bright red solid (108 mg, 82%).

Other analyses, reference papers, previously obtained data, comments, etc:

Analyst	analyst1
Date	Thursday, 4 July 2019 12:40 PM

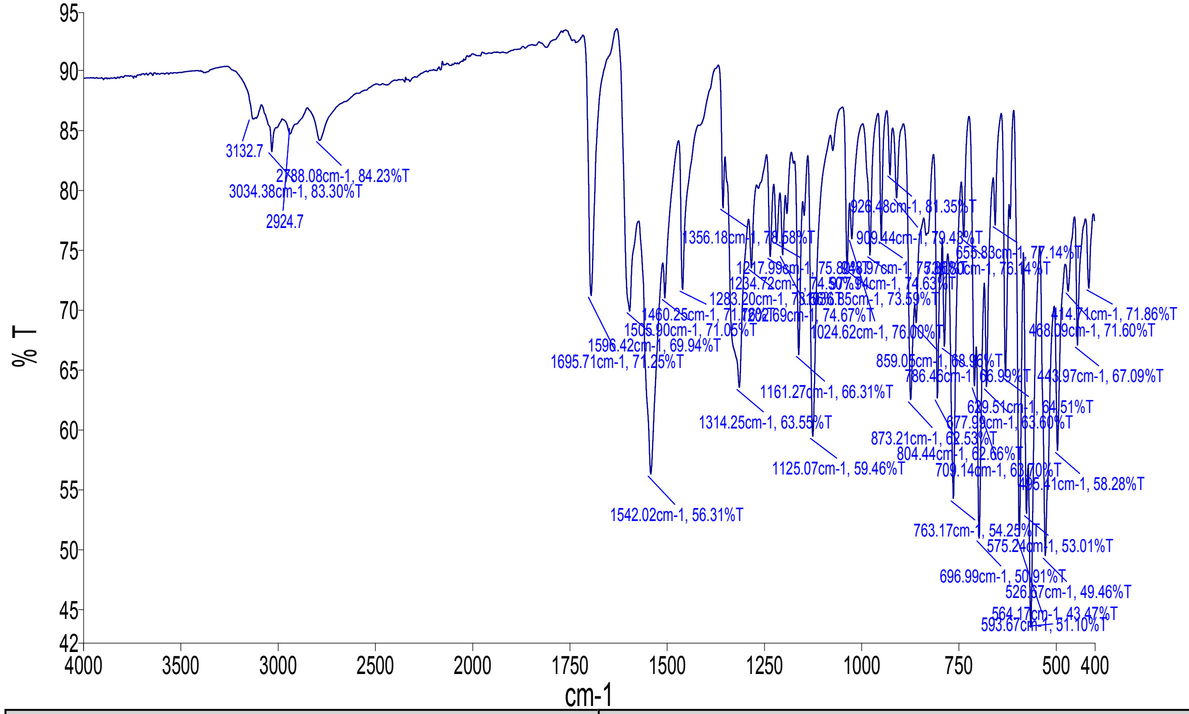

Sample Name	Description	Quality Checks
KP6083	Sample 012 By Analyst1 Date Thursday, July 04 2019	The Quality Checks do not report any warnings for the sample.

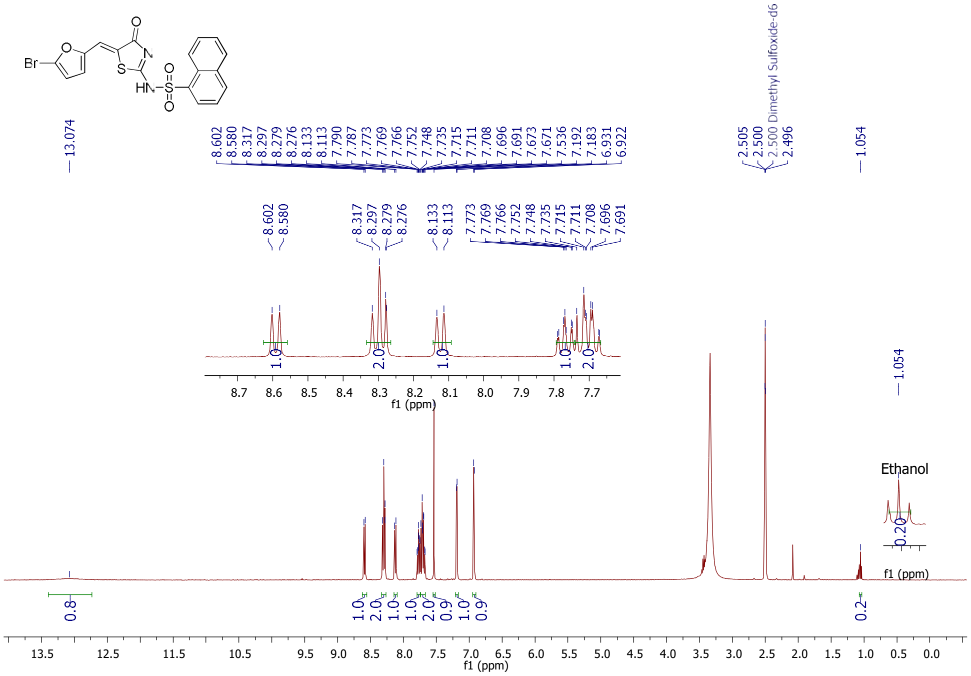
Br
O
S
N
HN
O
Dimethyl Sulfoxide-d6
Ethanol
f1 (ppm)

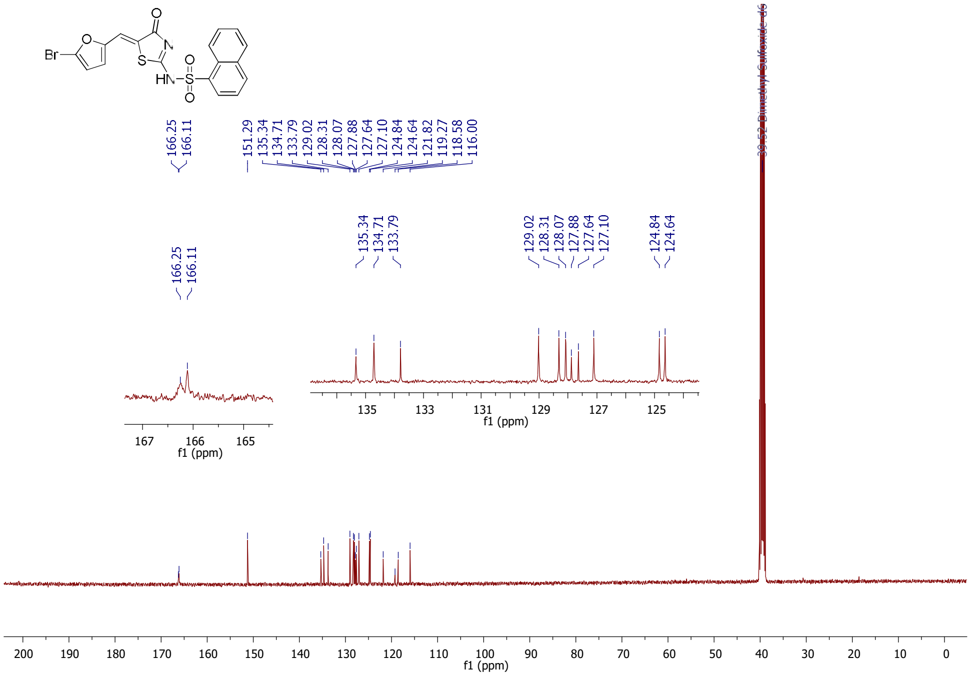
Br
O
S
N
HN
O
Dimethyl Sulfoxide-d6
f1 (ppm)

LCMS Report

Agilent Technologies

Data file: D:\Chem32\1\Data\KP\KP60838485 2019-08-13 14-20-02\002-47-KP6083.D
Sample name: KP6083
Description:
Sample amount: 0.000
Sample type: Sample

Instrument: LCMS
Location: 47
Injection date: 8/13/2019 2:29:17 PM
Injection: 1 of 1
Acq. method: LCMS ISOCRATIC 60% B 0.4MLMIN-1.M
Injection volume: 2.000
Analysis method: LCMS ISOCRATIC 60%B 0.4MLMIN-1.M
Acq. operator: SYSTEM
Last changed: 5/8/2019 8:55:04 AM

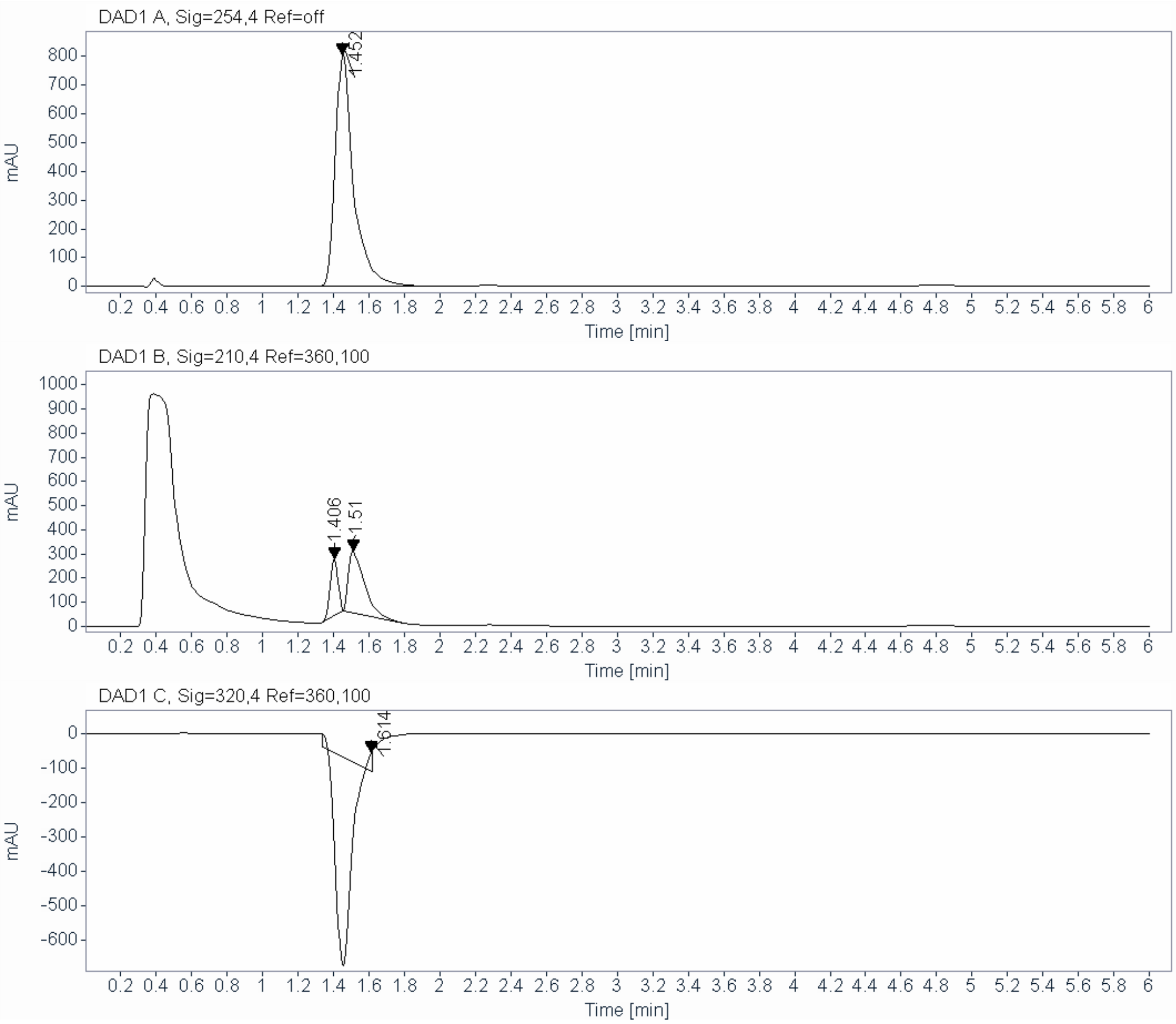

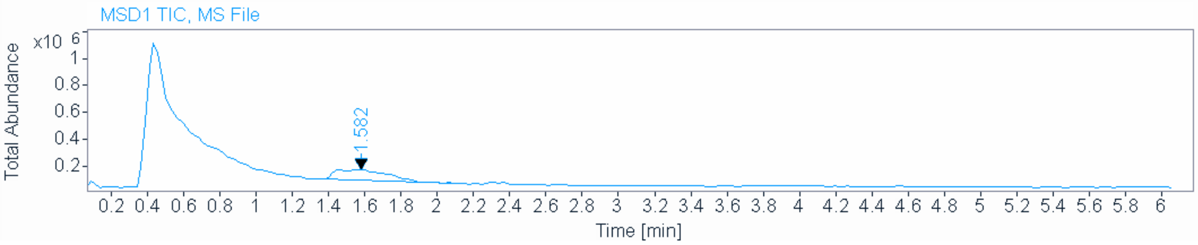

LCMS Report

Agilent Technologies

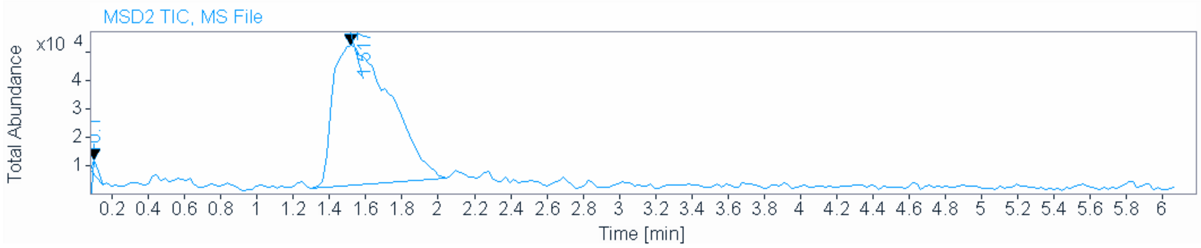

**Signal:**	DAD1 A, Sig=254,4 Ref=off					
**RT [min]**	**Type**	**Width [min]**	**Area**	**Height**	**Area%**	**Name**
1.452	BB	0.1075	5776.9487	807.2343	100.0000	
		Sum	5776.9487			

**Signal:**	DAD1 B, Sig=210,4 Ref=360,100					
**RT [min]**	**Type**	**Width [min]**	**Area**	**Height**	**Area%**	**Name**
1.406	BB	0.0503	735.2757	236.5503	31.0196	
1.510	BB	0.0898	1635.0862	258.6070	68.9804	
		Sum	2370.3619			

**Signal:**	DAD1 C, Sig=320,4 Ref=360,100					
**RT [min]**	**Type**	**Width [min]**	**Area**	**Height**	**Area%**	**Name**
1.614	MM	0.0364	119.6345	54.8529	100.0000	
		Sum	119.6345			

**Signal:**	MSD1 TIC, MS File					
**RT [min]**	**Type**	**Width [min]**	**Area**	**Height**	**Area%**	**Name**
1.582	BB	0.2600	1622714.250 0	81440.5000	100.0000	
		Sum	1622714.250			

**Signal:**	MSD2 TIC, MS File					
**RT [min]**	**Type**	**Width [min]**	**Area**	**Height**	**Area%**	**Name**
0.100	BB	0.0366	11426.3262	5196.6606	1.0410	
1.517	BB	0.3057	1086248.750 0	49208.0391	98.9590	
		Sum	1097675.076			

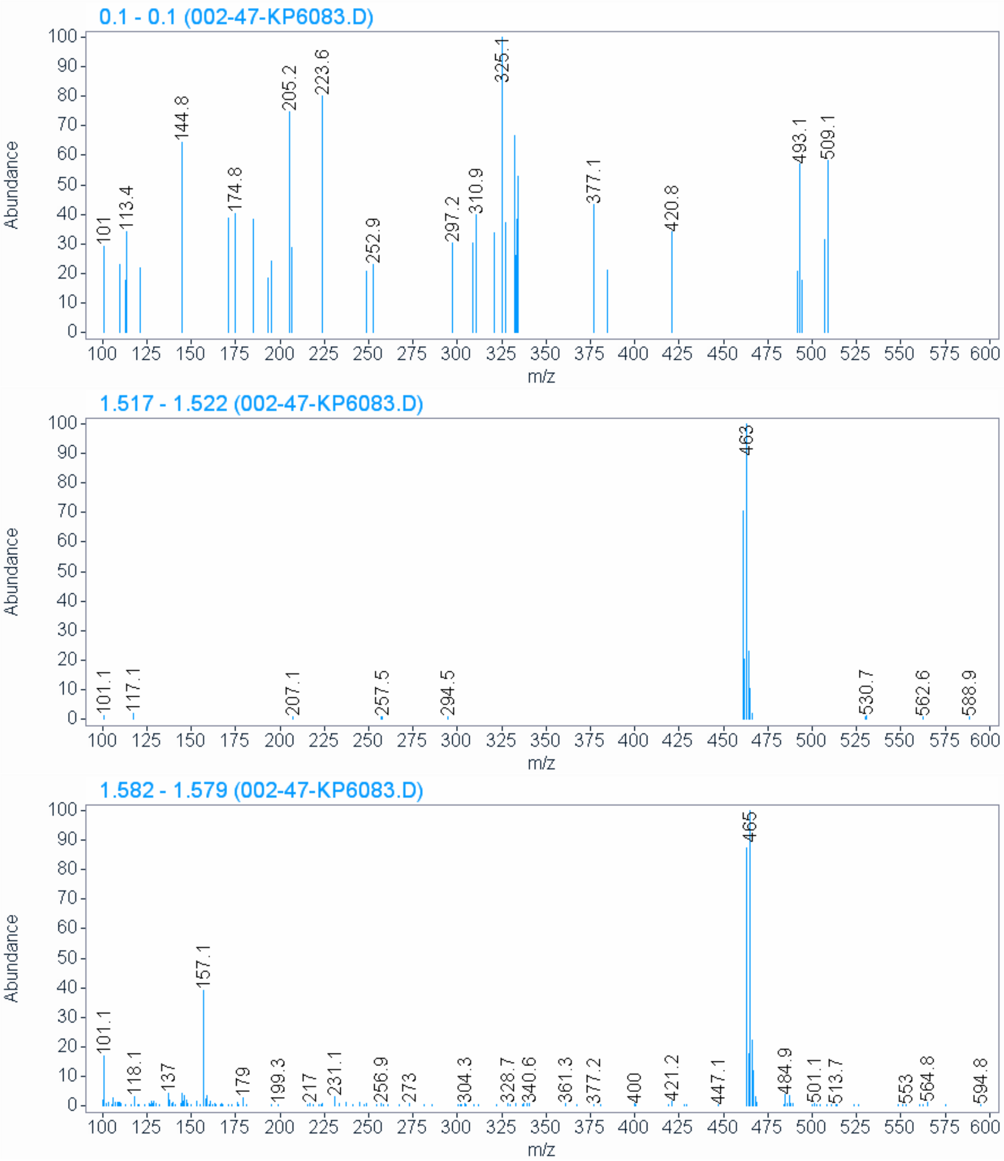
0.1 - 0.1 (002-47-KP6083.D)
Abundance
m/z
101
113.4
144.8
174.8
205.2
223.6
252.9
297.2
310.9
325.1
377.1
420.8
493.1
509.1
1.517 - 1.522 (002-47-KP6083.D)
101.1
117.1
207.1
257.5
294.5
463
530.7
562.6
588.9
1.582 - 1.579 (002-47-KP6083.D)
101.1
118.1
137
157.1
179
199.3
217
231.1
256.9
273
304.3
328.7
340.6
361.3
377.2
400
421.2
447.1
465
484.9
501.1
513.7
553
564.8
594.8

Compound Name: (Z)-*N*-(5-((5-(hydroxymethyl)furan-2-yl)methylene)-4-oxo-4,5-dihydrothiazol-2-yl)naphthalene-1-sulfonamide

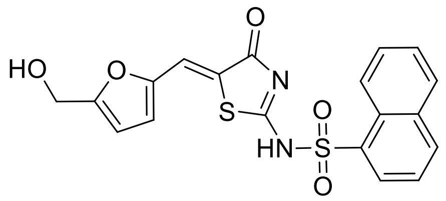

Compound Code: 51 (KP6092)

Obtained Weight & Yield: 65 mg (32%)

Purity (by LCMS and ^{1}H NMR): > 99% by ^{1}H-NMR

Appearance: Orange powder

Solubility: DMSO, slightly soluble in acetone.

Melting Point: > 267 °C (dec.)

TLC Rf (and conditions): 0.36 (10% MeOH in DCM)

IR Analysis (including assignment): IR (neat): ν_{max} = 3504 (OH), 3031 (NH), 2882 (aromatic CH), 2697 (CH), 1702 (C=O), 1556 (aromatic C-C), 1332 (sulfonamide), 1159 (C-O-C), 1126 (C-N) cm^{-1}

^{1}H NMR Analysis: ^{1}H NMR (400 MHz, DMSO) δ 12.99 (br, s, 1H, NH), 8.59 (d, *J* = 8.6 Hz, 1H), 8.30 (d, *J* = 7.7 Hz, 2H), 8.12 (d, *J* = 7.9 Hz, 1H), 7.77 (ddd, *J* = 8.5, 6.9, 1.3 Hz, 1H), 7.73 – 7.67 (m, 2H), 7.57 (s, 1H), 7.13 (d, *J* = 3.5 Hz, 1H), 6.62 (d, *J* = 3.5 Hz, 1H), 5.54 (br. s, 1H, OH), 4.56 (s, 2H) ppm.

^{13}C NMR Analysis: ^{13}C NMR (101 MHz, DMSO) δ 166.6, 166.3, 161.1, 148.5, 135.4, 134.6, 133.8, 129.0, 128.3, 128.1, 127.7, 127.1, 124.9, 124.6, 121.2, 120.0, 117.5, 111.0, 56.0 ppm.

MS Analysis (low res): LRMS (ESI-) *m/z*: 413 (*M*-H, $C_{19}H_{13}N_2O_5S_2$, 100%)

HPLC method details: Column: Zorbax SB-C18 Rapid Resolution HT 2.1x50mm 1.8-Micron; Method: LCMS ISOCRATIC 60%B 0.4MLMIN-1.M; filename: KP6092; Peak retention time: 0.57 mins; Area (%): 100.

Procedure: To a microwave vial was added the *N*-(4-oxo-4,5-dihydrothiazol-2-yl)naphthalene-1-sulfonamide (151 mg, 0.49 mmol), 5-hydroxymethyl-2-furaldehyde (74.7 mg, 0.59 mmol, 1.2 eq), ethanol (3 mL) and the benzoic acid/piperidine catalyst (3 drops). The reaction mixture was treated with microwave irradiation (120°C, 30 min). Addition of H_2O to the reaction mixture resulted in formation of a precipitate which was collected by vacuum filtration and washed with ethanol (5 mL) and diethyl ether (10 mL) to give an orange solid (65 mg, 32%).

Other analyses, reference papers, previously obtained data, comments, etc:

PerkinElmer Spectrum Version 10.4.4
Thursday, 4 July 2019 12:24 PM

Analyst analyst1
Date Thursday, 4 July 2019 12:24 PM

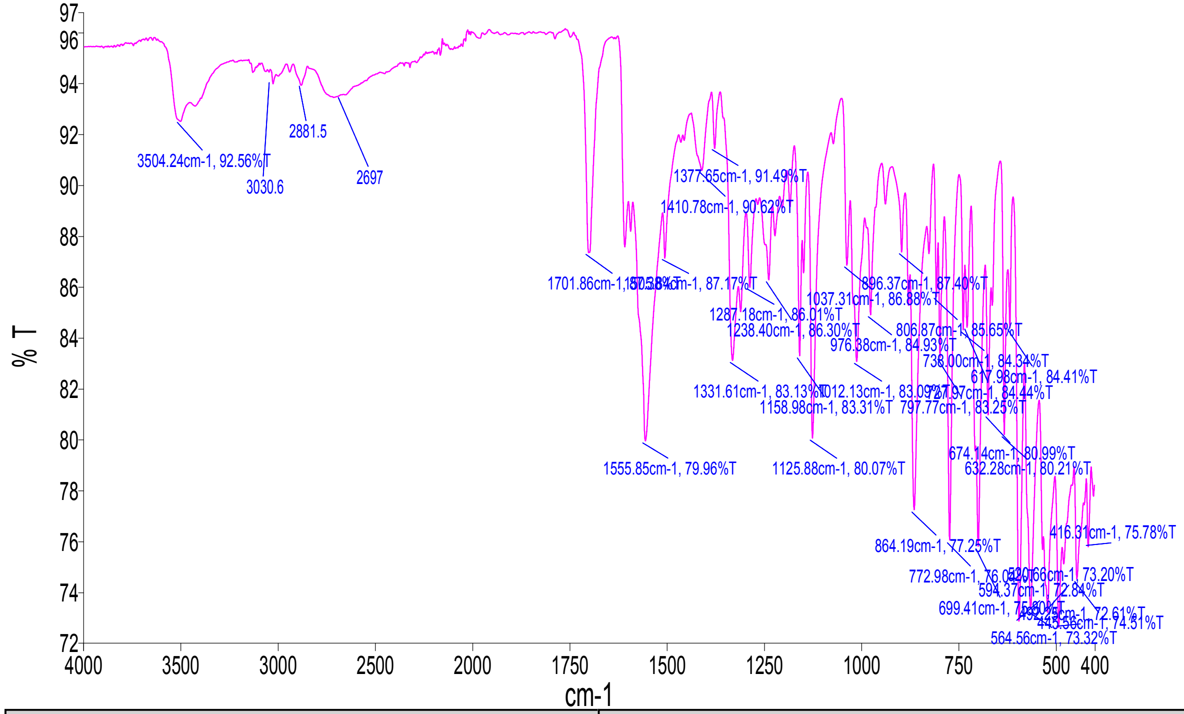

Sample Name	Description	Quality Checks
KP6072	Sample 006 By Analyst1 Date Thursday, July 04 2019	The Quality Checks give rise to a Weak Bands warning for the sample.

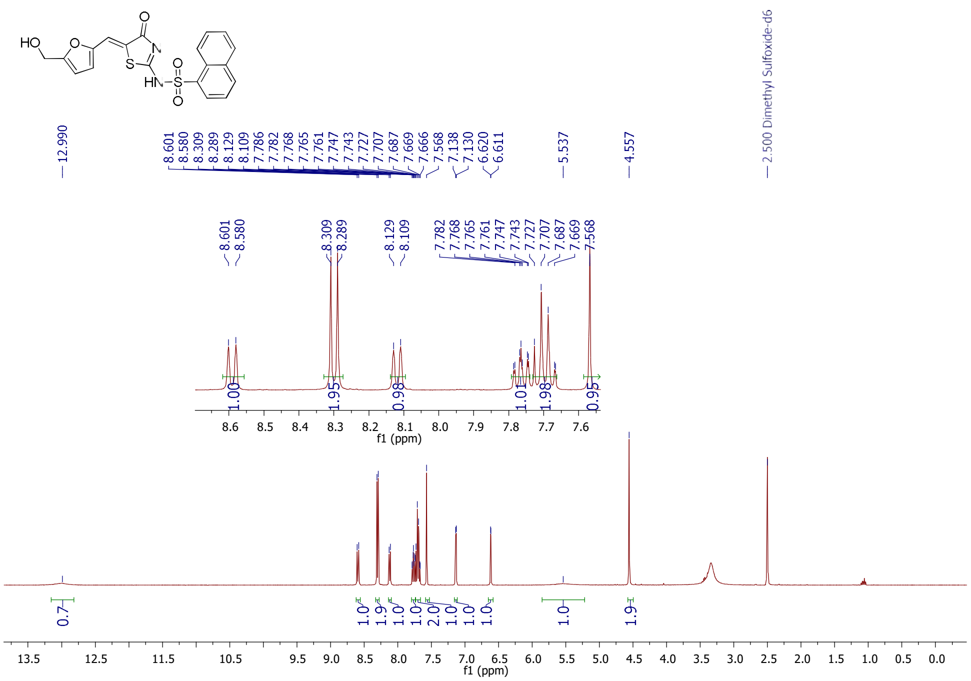

HO
O
S
N
O
HN
S
O
O
12.990
8.601
8.580
8.309
8.289
8.129
8.109
7.786
7.782
7.768
7.765
7.761
7.747
7.743
7.727
7.707
7.687
7.669
7.666
7.568
7.138
7.130
6.620
6.611
5.537
4.557
2.500 Dimethyl Sulfoxide-d6
1.00
1.95
0.98
1.01
1.98
0.95
f1 (ppm)
0.7
1.0
1.9
1.0
1.0
2.0
1.0
1.0
1.0
1.9
f1 (ppm)

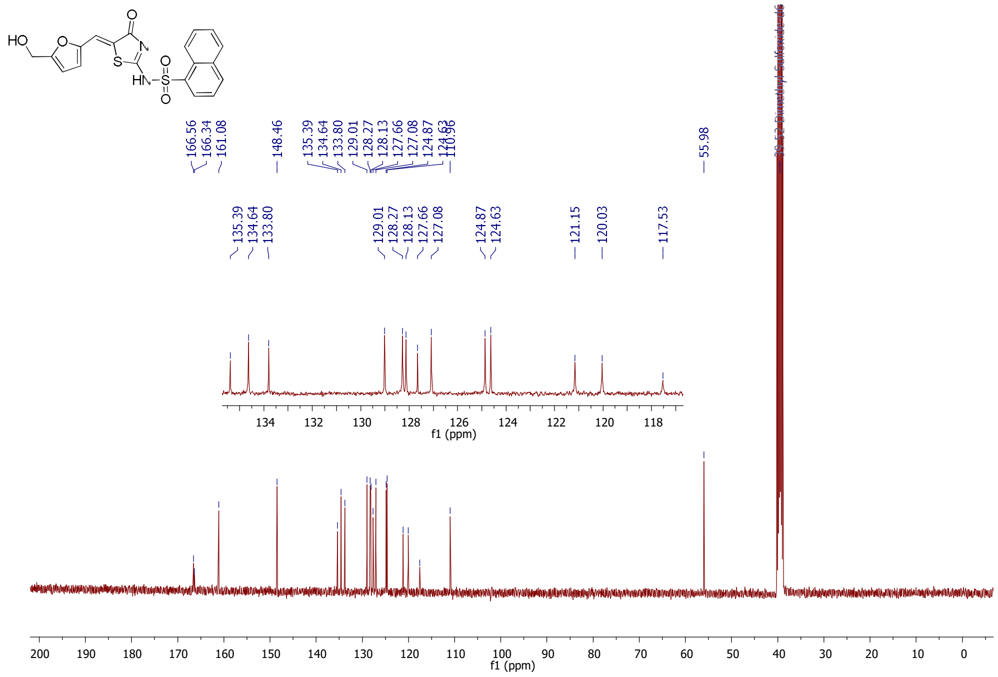

HO
O
S
N
O
HN
S
O
O
166.56
166.34
161.08
148.46
135.39
134.64
133.80
129.01
128.27
128.13
127.66
127.08
124.87
124.63
110.96
55.98
121.15
120.03
117.53
f1 (ppm)
f1 (ppm)

Agilent Technologies

**Data file:**	D:\Chem32\1\Data\KP\KP609192 2019-08-14 15-23-30\003-55-KP6092.D		
**Sample name:**	KP6092		
**Description:**			
**Sample amount:**	0.000	**Sample type:**	Sample
**Instrument:**	LCMS	**Location:**	55
**Injection date:**	8/14/2019 3:40:23 PM	**Injection:**	1 of 1
**Acq. method:**	LCMS ISOCRATIC 60% B 0.4MLMIN-1.M	**Injection volume:**	2.000
**Analysis method:**	LCMS ISOCRATIC 60%B 0.4MLMIN-1.M	**Acq. operator:**	SYSTEM
**Last changed:**	5/8/2019 8:55:04 AM		

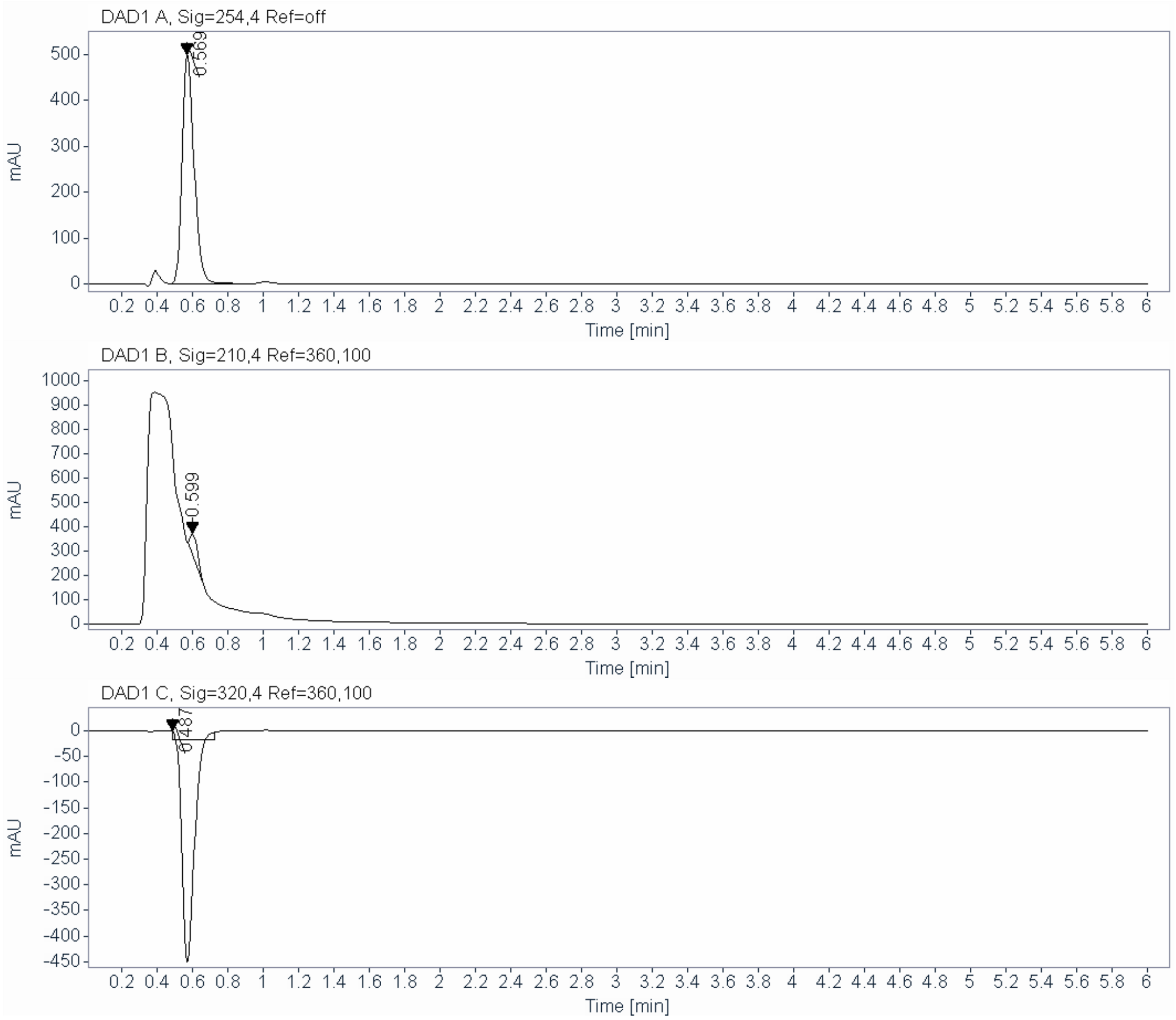

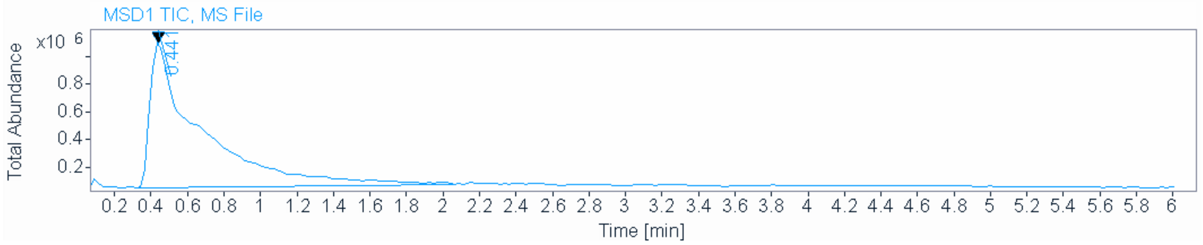

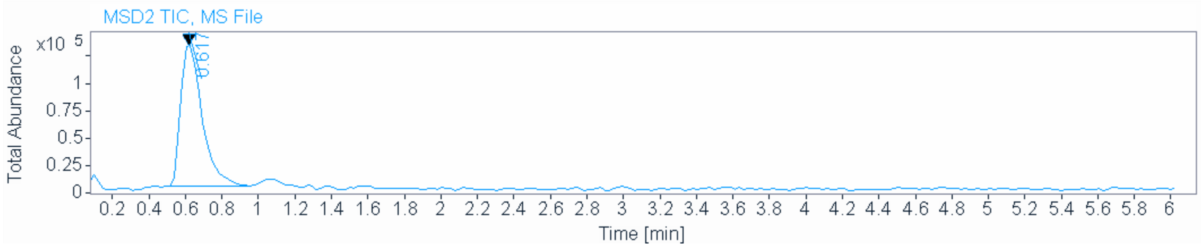

Signal: DAD1 A, Sig=254,4 Ref=off

RT [min]	Type	Width [min]	Area	Height	Area%	Name
0.569	BB	0.0752	2368.9465	500.7723	100.0000	
		Sum	2368.9465			

Signal: DAD1 B, Sig=210,4 Ref=360,100

RT [min]	Type	Width [min]	Area	Height	Area%	Name
0.599	BB	0.0481	261.7215	89.4750	100.0000	
		Sum	261.7215			

Signal: DAD1 C, Sig=320,4 Ref=360,100

RT [min]	Type	Width [min]	Area	Height	Area%	Name
0.487	MM	0.0422	41.1454	16.2676	100.0000	
		Sum	41.1454			

Signal: MSD1 TIC, MS File

RT [min]	Type	Width [min]	Area	Height	Area%	Name
0.441	BB	0.2489	19924658.0000	1050104.5000	100.0000	
		Sum	19924658.00			

Signal: MSD2 TIC, MS File

RT [min]	Type	Width [min]	Area	Height	Area%	Name
0.617	BB	0.1231	1067011.6250	130309.8984	100.0000	
		Sum	1067011.625			

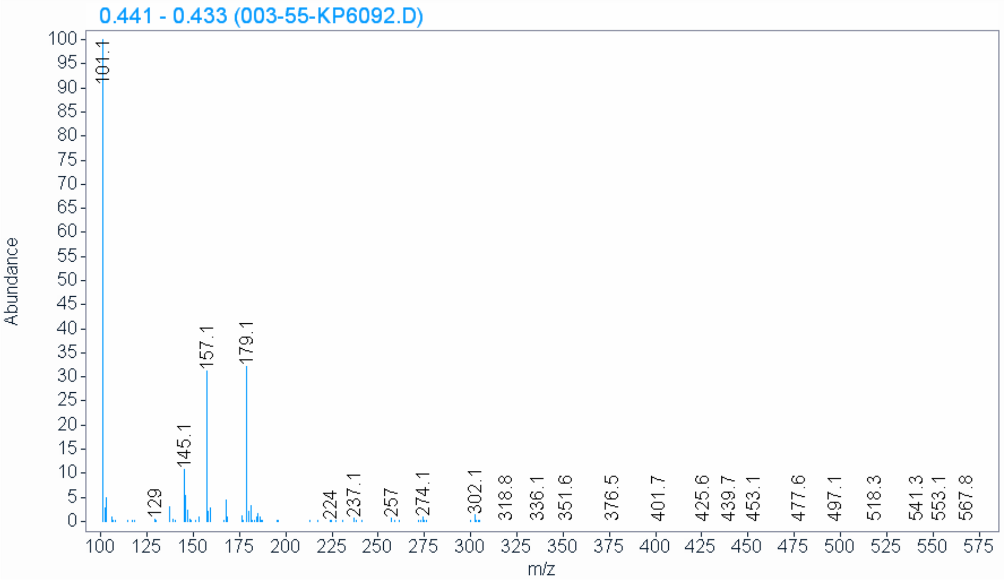
0.441 - 0.433 (003-55-KP6092.D)
Abundance
m/z
101.1
129
145.1
157.1
179.1
224
237.1
257
274.1
302.1
318.8
336.1
351.6
376.5
401.7
425.6
439.7
453.1
477.6
497.1
518.3
541.3
553.1
567.8

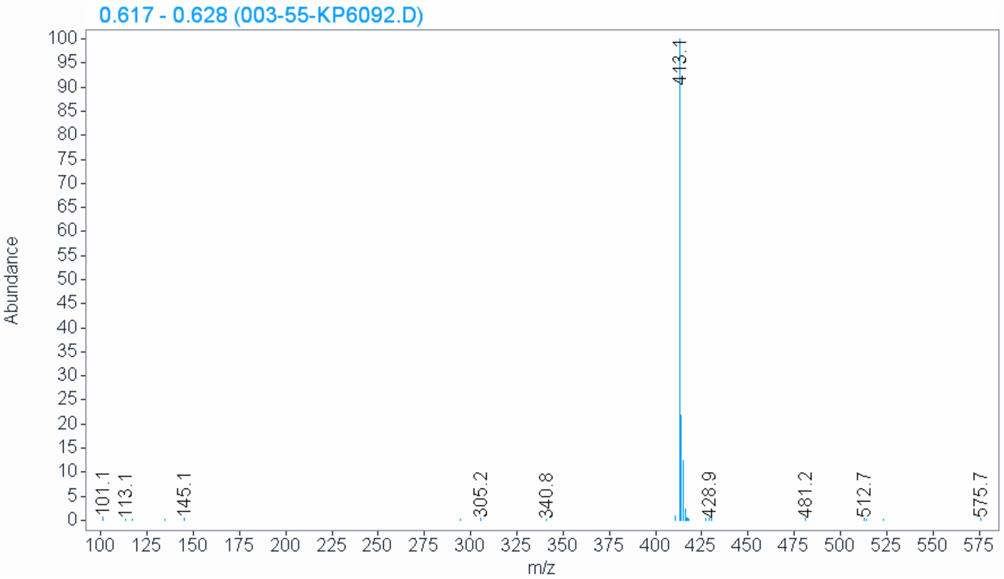
0.617 - 0.628 (003-55-KP6092.D)
Abundance
m/z
413.1
101.1
113.1
145.1
305.2
340.8
428.9
481.2
512.7
575.7

Compound Name: (Z)-*N*-(4-oxo-5-((3-oxo-3,4-dihydro-2H-benzo[b][1,4]oxazin-6-yl)methylene)-4,5-dihydrothiazol-2-yl)naphthalene-1-sulfonamide

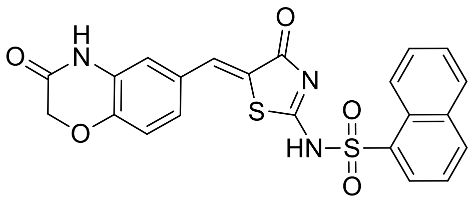

Chemical Formula: $C_{22}H_{15}N_3O_5S_2$
Exact Mass: 465.05
Molecular Weight: 465.50

Compound Code: 52 (KP6040)

Obtained Weight & Yield: 127 mg (83%)

Purity (by LCMS and ^{1}H NMR): > 98% by ^{1}H-NMR

Appearance: Yellow/orange powder

Solubility: DMSO, slightly soluble in acetone, ethanol and methanol.

Melting Point: > 300 °C (dec.)

TLC Rf (and conditions): 0.24 (10% MeOH in DCM)

IR Analysis (including assignment): IR (neat): ν_{max} = 3178, 3036 (C-H aromatic), 1726, 1669 (C=O), 1553 (C=C aromatic), 1324 (sulfonamide), 1126 (C-N) cm^{-1}

^{1}H NMR Analysis: ^{1}H NMR (400 MHz, DMSO) δ 11.12 (s, 1H), 8.61 (d, *J* = 8.7 Hz, 1H), 8.32 – 8.29 (m, 2H), 8.12 (d, *J* = 8.0 Hz, 1H), 7.77 (ddd, *J* = 8.5, 6.9, 1.4 Hz, 1H), 7.73 – 7.67 (m, 2H), 7.66 (s, 1H), 7.29 (dd, *J* = 8.5, 2.1 Hz, 1H), 7.22 (d, *J* = 2.1 Hz, 1H), 7.13 (d, *J* = 8.4 Hz, 1H), 4.70 (s, 2H) ppm. Sulfonamide NH not visible – exchanging. Ethanol at 1.05 ppm (0.46%), aldehyde starting material at 9.84 ppm (0.76%)

^{13}C NMR Analysis: ^{13}C NMR (151 MHz, DMSO) δ 165.9 (br), 164.3, 145.5, 135.4, 134.7, 133.8, 133.3 (br), 129.0, 128.3, 128.10, 128.08, 127.7, 127.2, 127.1, 127.0, 124.9, 124.6, 117.1, 116.1, 66.8 pm. Two carbons not visible.

MS Analysis (low res): LRMS (ESI-) *m/z*: 464 (M-H, $C_{22}H_{14}N_3O_5S_2$, 100%)

HPLC method details: Column: Zorbax SB-C18 Rapid Resolution HT 2.1x50mm 1.8-Micron; Method: LCMS ISOCRATIC 50% B.M_REDUCED FLOW.M filename: KP6040; Peak retention time: 1.24 mins; Area (%): 99

Procedure: To a microwave vial was added the *N*-(4-oxo-4,5-dihydrothiazol-2-yl)naphthalene-1-sulfonamide (102 mg, 0.33 mmol), 3-oxo-3,4-dihydro-2H-benzo[1,4]oxazine-6-carbaldehyde (69 mg, 0.39 mmol, 1.2 eq), ethanol (3 mL) and the benzoic acid/piperidine catalyst (approximately 5 drops). The reaction mixture was heated by microwave irradiation (120 °C) for 30 min after which the reaction was cooled overnight. A yellow precipitate was collected by vacuum filtration and washed with cold ether to give the desired product (127 mg, 83%).

Other analyses, reference papers, previously obtained data, comments, etc:

PerkinElmer Spectrum Version 10.4.00
Monday, 27 May 2019 1:46 PM

Analyst	research
Date	Monday, 27 May 2019 1:46 PM

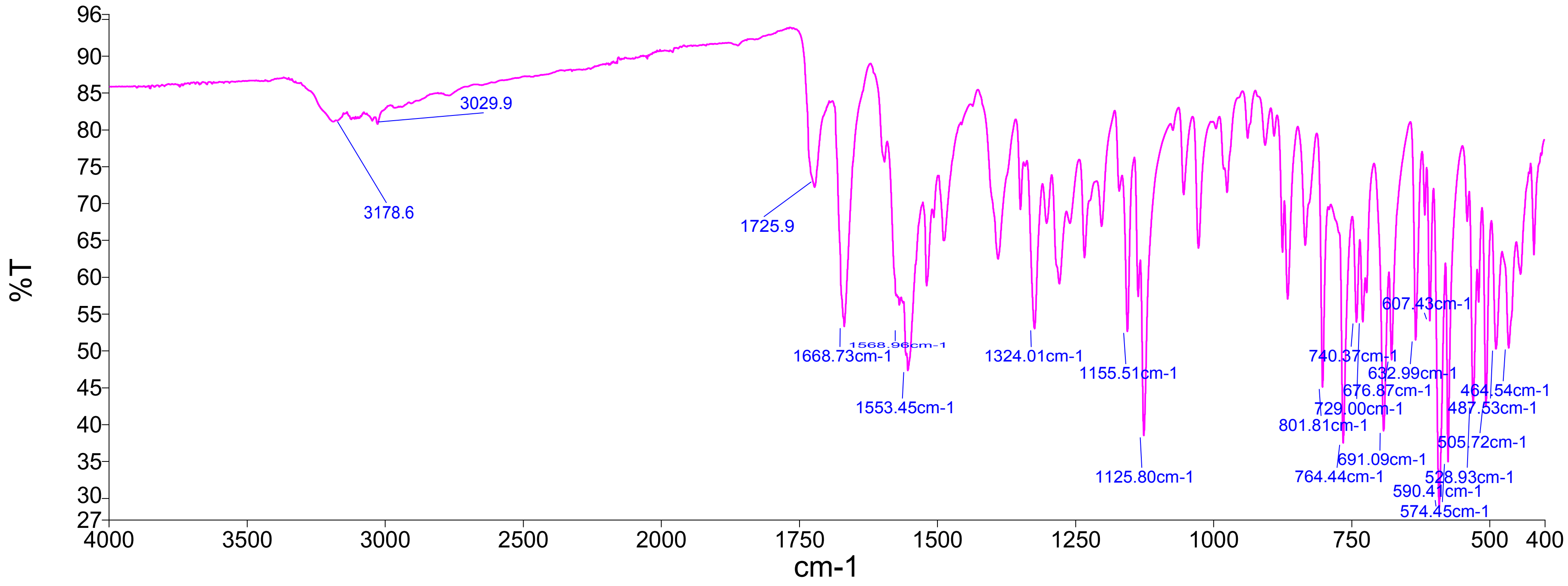

Sample Name	Description	Quality Checks
KP6040	Sample 215 By research Date Monday, May 27 2019	The Quality Checks do not report any warnings for the sample.

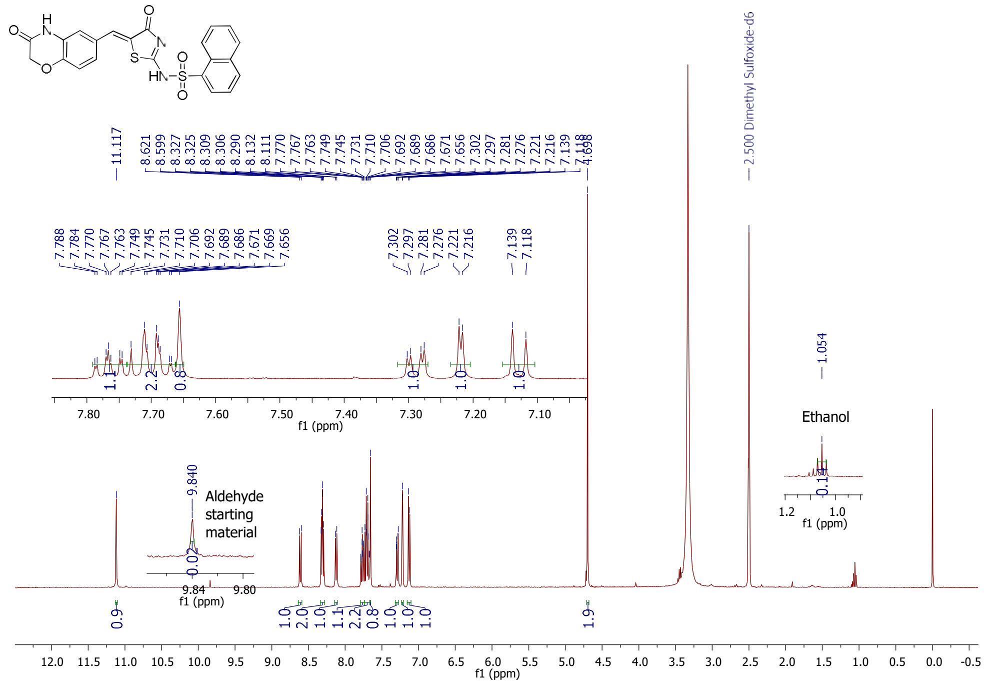

— 11.117
8.621
8.599
8.327
8.325
8.306
8.309
8.290
8.132
8.111
7.770
7.767
7.763
7.749
7.745
7.731
7.710
7.706
7.692
7.689
7.686
7.671
7.656
7.302
7.297
7.281
7.276
7.221
7.216
7.139
7.118
4.698
— 2.500 Dimethyl Sulfoxide-d6
7.788
7.784
7.770
7.767
7.763
7.749
7.745
7.731
7.710
7.706
7.692
7.689
7.686
7.671
7.669
7.656
7.302
7.297
7.281
7.276
7.221
7.216
7.139
7.118
1.1
2.2
0.8
1.0
1.0
1.0
7.80 7.70 7.60 7.50 7.40 7.30 7.20 7.10
f1 (ppm)
— 1.054
Ethanol
1.2 1.0
f1 (ppm)
9.840
Aldehyde
starting
material
0.02
9.84 9.80
f1 (ppm)
0.9
1.0
2.0
1.0
1.1
2.2
0.8
1.0
1.0
1.0
1.9
12.0 11.5 11.0 10.5 10.0 9.5 9.0 8.5 8.0 7.5 7.0 6.5 6.0 5.5 5.0 4.5 4.0 3.5 3.0 2.5 2.0 1.5 1.0 0.5 0.0 -0.5
f1 (ppm)

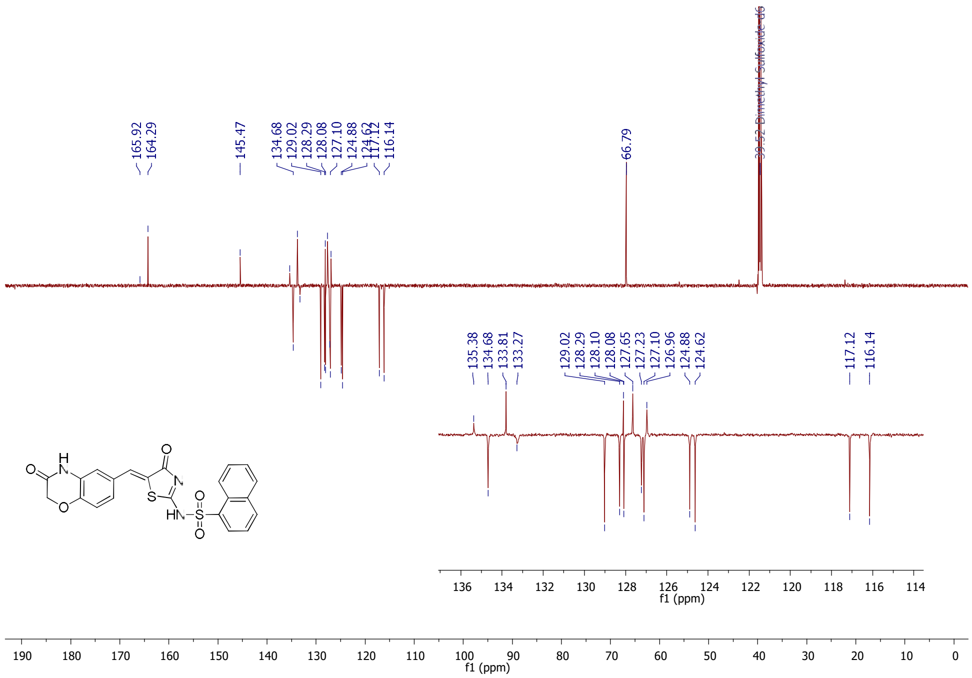

165.92
164.29
— 145.47
134.68
129.02
128.29
128.08
127.10
124.88
124.62
117.12
116.14
— 66.79
Dimethyl Sulfoxide-d6
135.38
134.68
133.81
133.27
129.02
128.29
128.10
128.08
127.65
127.23
127.10
126.96
124.88
124.62
117.12
116.14
136 134 132 130 128 126 124 122 120 118 116 114
f1 (ppm)
190 180 170 160 150 140 130 120 110 100 90 80 70 60 50 40 30 20 10 0
f1 (ppm)

LCMS Report

Agilent Technologies

**Data file:**	D:\Chem32\1\Data\KP\PRE 11-6-19\KP6039-47 2019-05-21 10-32-44\005-36-KP6040.D		
**Sample name:**	KP6040		
**Description:**			
**Sample amount:**	0.000	**Sample type:**	Sample
**Instrument:**	LCMS	**Location:**	36
**Injection date:**	5/21/2019 11:01:04 AM	**Injection:**	1 of 1
**Acq. method:**	LCMS ISOCRATIC 50% B.M_REDUCED FLOW.M	**Injection volume:**	2.000
**Analysis method:**	LCMS ISOCRATIC 50% B.M_REDUCED FLOW.M	**Acq. operator:**	SYSTEM
**Last changed:**	5/15/2019 9:20:00 AM		

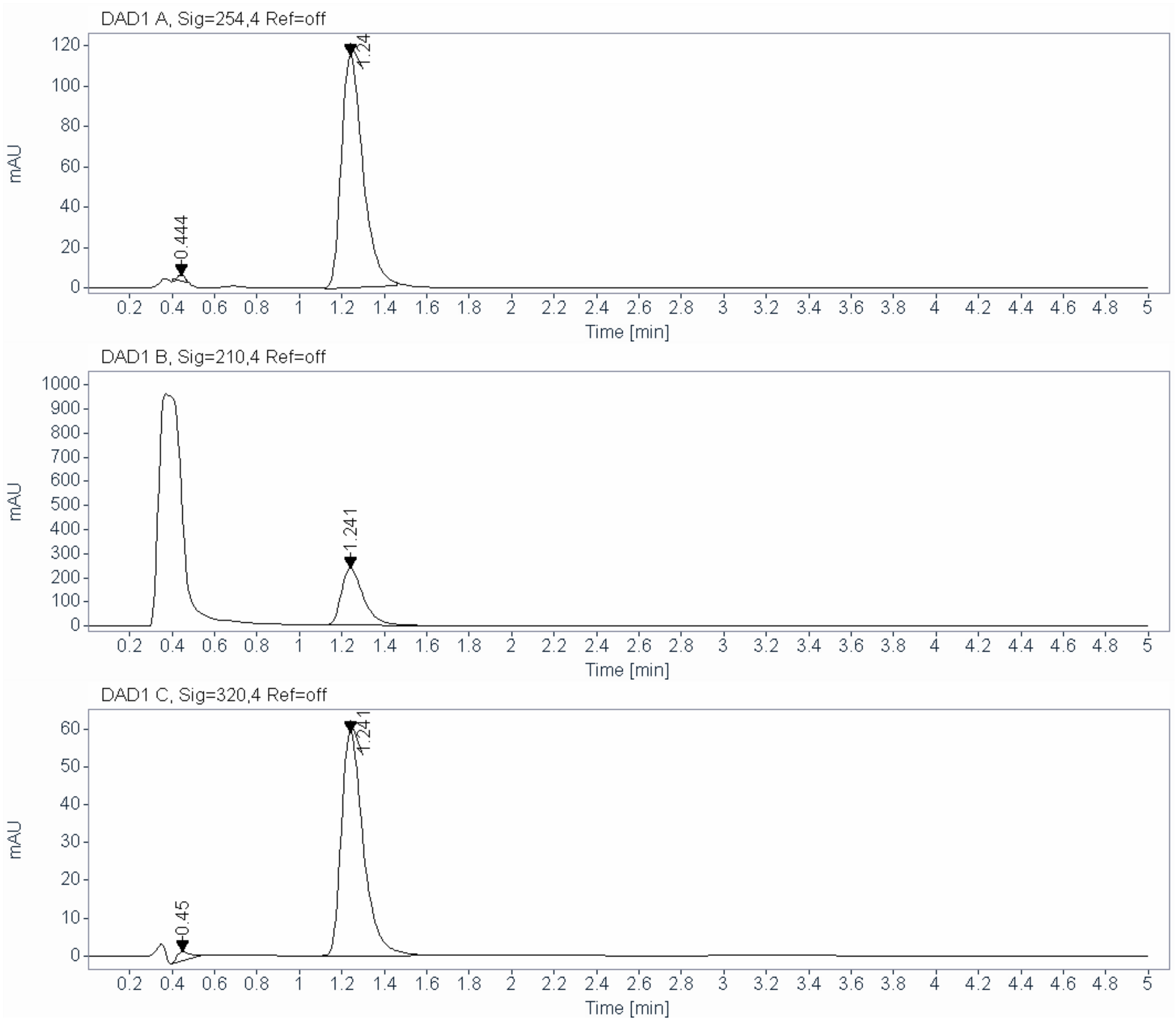

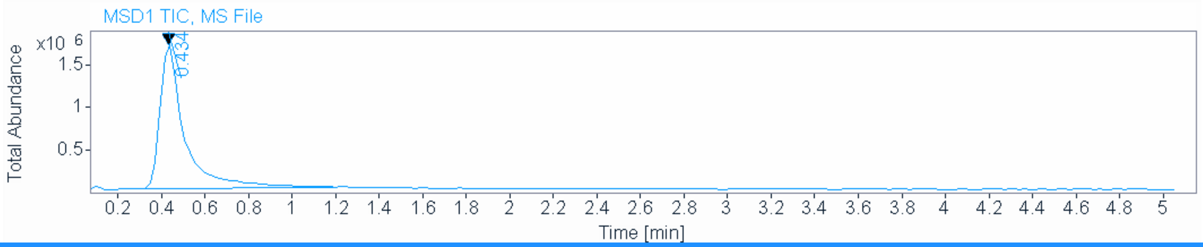

Agilent Technologies

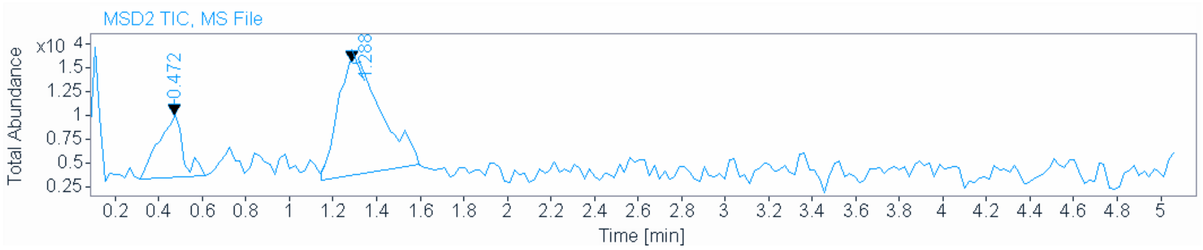

Signal: DAD1 A, Sig=254,4 Ref=off

RT [min]	Type	Width [min]	Area	Height	Area%	Name
0.444	MM	0.0363	5.9548	2.7360	0.7237	
1.240	MM	0.1183	816.8444	115.0760	99.2763	
		Sum	822.7992			

Signal: DAD1 B, Sig=210,4 Ref=off

RT [min]	Type	Width [min]	Area	Height	Area%	Name
1.241	BB	0.1130	1722.7483	236.4216	100.0000	
		Sum	1722.7483			

Signal: DAD1 C, Sig=320,4 Ref=off

RT [min]	Type	Width [min]	Area	Height	Area%	Name
0.450	BB	0.0700	11.1035	2.4904	2.5223	
1.241	BB	0.1126	429.1121	59.1903	97.4777	
		Sum	440.2156			

Signal: MSD1 TIC, MS File

RT [min]	Type	Width [min]	Area	Height	Area%	Name
0.434	BB	0.1118	13329968.0000	1706553.2500	100.0000	
		Sum	13329968.00			

Signal: MSD2 TIC, MS File

RT [min]	Type	Width [min]	Area	Height	Area%	Name
0.472	BB	0.1204	51538.0156	6474.0205	23.6691	
1.288	MM	0.2269	166205.9375	12206.4756	76.3309	
		Sum	217743.9531			

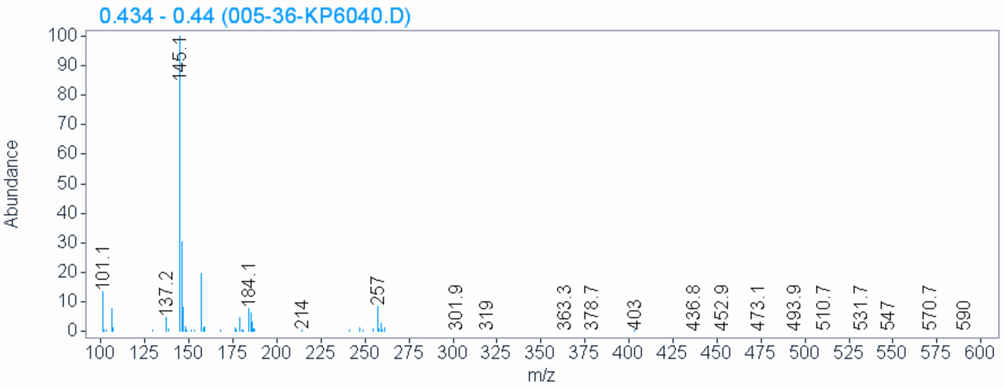
0.434 - 0.44 (005-36-KP6040.D)
Abundance
m/z
101.1
137.2
145.1
184.1
214
257
301.9
319
363.3
378.7
403
436.8
452.9
473.1
493.9
510.7
531.7
547
570.7
590

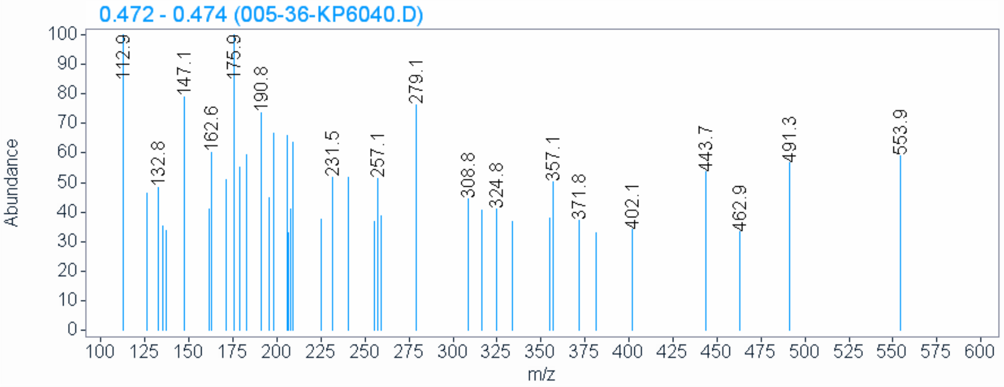
0.472 - 0.474 (005-36-KP6040.D)
Abundance
m/z
112.9
132.8
147.1
162.6
175.9
190.8
231.5
257.1
279.1
308.8
324.8
357.1
371.8
402.1
443.7
462.9
491.3
553.9

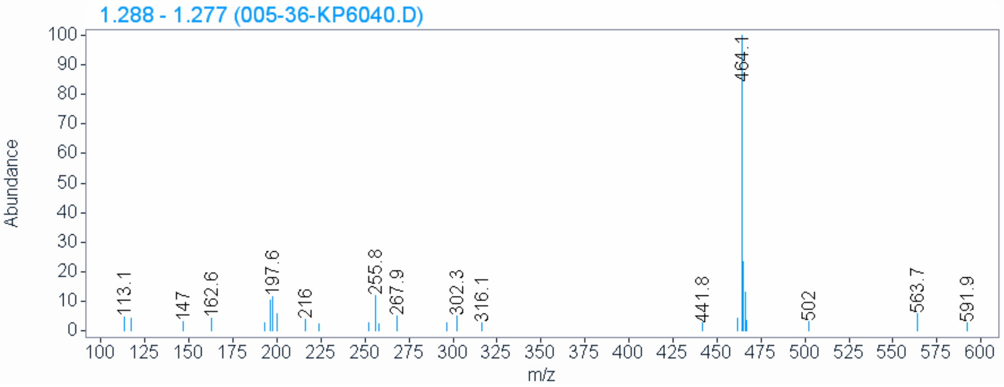
1.288 - 1.277 (005-36-KP6040.D)
Abundance
m/z
113.1
147
162.6
197.6
216
255.8
267.9
302.3
316.1
441.8
464.1
502
563.7
591.9

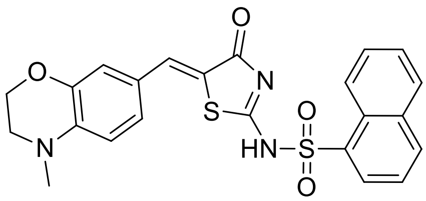

Compound Name: (Z)-*N*-(5-((4-methyl-3,4-dihydro-2H-benzo[b][1,4]oxazin-7-yl)methylene)-4-oxo-4,5-dihydrothiazol-2-yl)naphthalene-1-sulfonamide

Compound Code: 53 (KP6103)

Obtained Weight & Yield: 77 mg, 34%

Purity (by LCMS and ^{1}H NMR): > 97% by ^{1}H-NMR and LCMS

Appearance: red solid

Solubility: DMSO, slightly soluble in methanol and acetone

Melting Point: > 242 °C (dec.)

TLC Rf (and conditions): N/A

IR Analysis (including assignment): IR (neat): ν_{max} = 2926 (aromatic C-H), 2977 (C-H), 1684 (C=O), 1520 (aromatic C-C), 1300 (sulfonamide), 1213 (C-O-C), 1122 (C-N) cm^{-1}

^{1}H NMR Analysis: ^{1}H NMR (400 MHz, DMSO) δ 12.95 (s, 1H), 8.61 (d, *J* = 8.5 Hz, 1H), 8.30 (dd, *J* = 7.8, 4.2 Hz, 2H), 8.11 (d, *J* = 8.1 Hz, 1H), 7.78 – 7.74 (m, 1H), 7.73 – 7.67 (m, 2H), 7.57 (s, 1H), 7.16 (dd, *J* = 8.5, 2.0 Hz, 1H), 6.97 (d, *J* = 2.1 Hz, 1H), 6.82 (d, *J* = 8.6 Hz, 1H), 4.25 – 4.22 (m, 2H), 3.43 – 3.41 (m, 2H), 2.97 (s, 3H) ppm.
Some impurities < 3% total. Ethanol at 4.05 ppm (0.88%) and starting material at 4.04 ppm (1.93%).

^{13}C NMR Analysis: ^{13}C NMR (101 MHz, DMSO) δ 165.8, 143.2, 139.6, 135.6, 134.8, 134.5, 133.8, 129.0, 128.2, 128.0, 127.7, 127.1, 126.5, 124.9, 124.6, 120.8, 116.5, 114.5, 111.8, 63.8, 48.0, 37.7 ppm
One carbon missing (quaternary carbon)

MS Analysis (low res): LRMS (ESI-) *m/z* (%): 464 (*M*-H, $C_{23}H_{18}N_3O_4S_2$, 100%)

MS Analysis (high res): Exact mass calculated for $C_{23}H_{18}N_3O_4S_2$ $[M\text{-H}]^-$, 464.0700. Found 464.0742.

HPLC method details: Column: Zorbax SB-C18 Rapid Resolution HT 2.1x50mm 1.8-Micron; Method: LCMS ISOCRATIC 60%B 0.4MLMIN-1.M filename: KP6102; Peak retention time: 1.458 mins; Area (%): 100.

Procedure: To a 10 mL microwave vial was added *N*-(4-oxo-4,5-dihydrothiazol-2-yl)naphthalene-1-sulfonamide (150 mg, 0.49 mmol), 4-methyl-3,4-dihydro-*2H*-1,4-benzoxazio-7-carboxaldehyde (100 mg, 0.54 mmol, 1.1 eq), ethanol (3 mL) and a catalytic amount of the benzoic acid/piperidine catalyst (approximately 2 drops). The suspension was heated using microwave irradiation (200 W, 120 °C) for 30 min then placed in the freezer over an extended period. The resulting precipitate was collected by vacuum filtration and washed with cold ethanol and cold ether to give the desired product (77 mg, 34%)

Other analyses, reference papers, previously obtained data, comments, etc:

PerkinElmer Spectrum Version 10.4.00
Tuesday, 23 July 2019 11:44 AM

Analyst	research
Date	Tuesday, 23 July 2019 11:44 AM

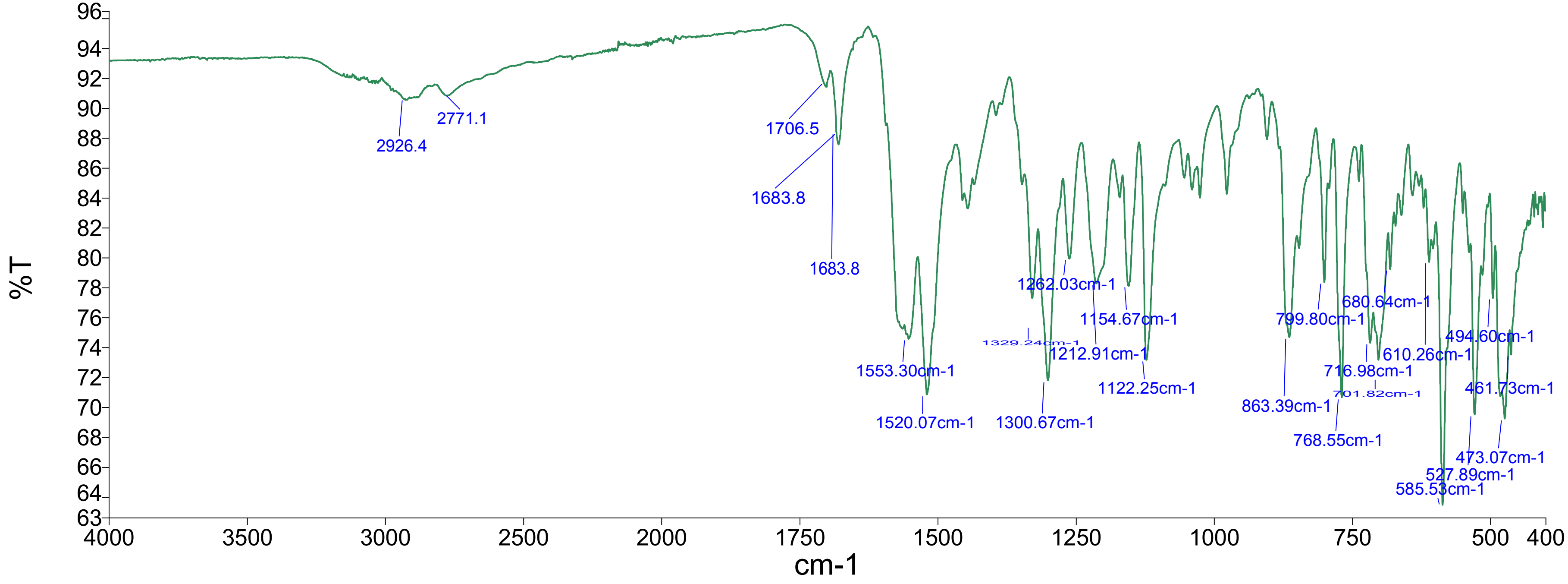

Sample Name	Description	Quality Checks
KP6103	Sample 236 By research Date Tuesday, July 23 2019	The Quality Checks give rise to a Weak Bands warning for the sample.

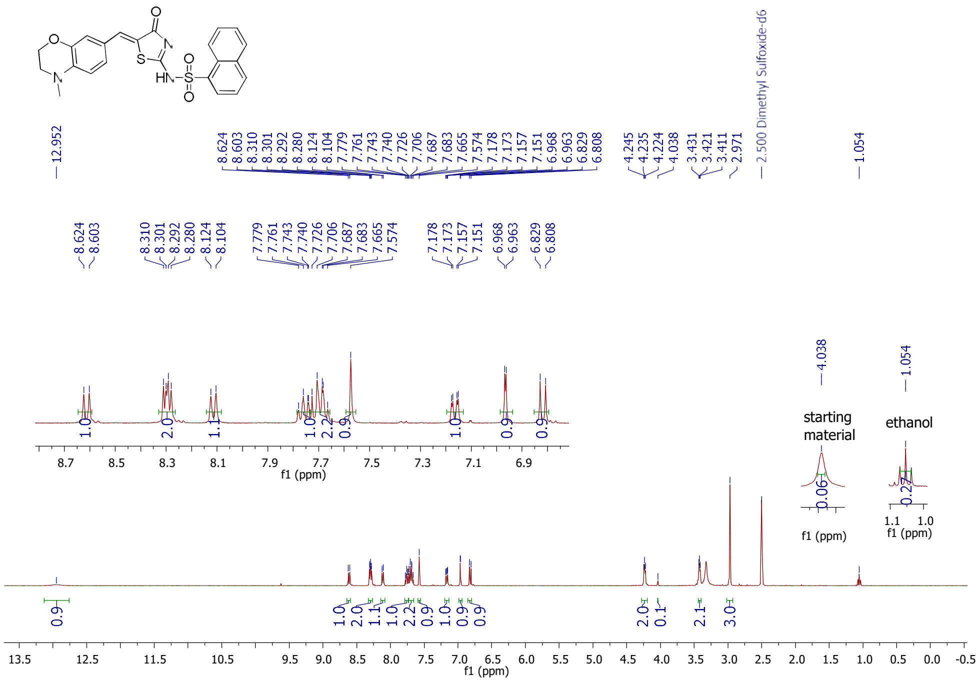
12.952
8.624 8.603 8.310 8.301 8.292 8.280 8.124 8.104 7.779 7.761 7.743 7.740 7.726 7.706 7.687 7.683 7.665 7.574 7.178 7.173 7.157 7.151 6.968 6.963 6.829 6.808
4.245 4.235 4.224 4.038 3.431 3.421 3.411 2.971
2.500 Dimethyl Sulfoxide-d6
1.054
starting material
ethanol
f1 (ppm)

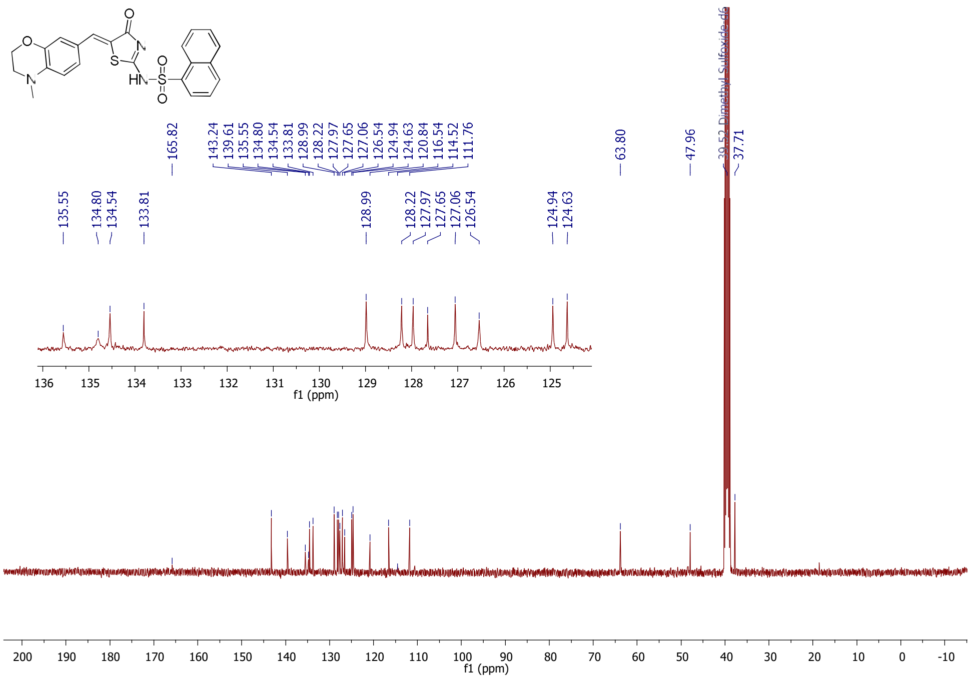
165.82
143.24 139.61 135.55 134.80 134.54 133.81 128.99 128.22 127.97 127.65 127.06 126.54 124.94 124.63 120.84 116.54 114.52 111.76
63.80
47.96
39.52 Dimethyl Sulfoxide-d6
37.71
f1 (ppm)

LCMS Report

Agilent Technologies

**Data file:**	D:\Chem32\1\Data\KP\KP_DS_11NOV 2019-11-11 14-45-23\005-44-KP6103.D		
**Sample name:**	KP6103		
**Description:**			
**Sample amount:**	0.000	**Sample type:**	Sample
**Instrument:**	LCMS	**Location:**	44
**Injection date:**	11/11/2019 3:18:44 PM	**Injection:**	1 of 1
**Acq. method:**	LCMS ISOCRATIC 60% B 0.4MLMIN-1.M	**Injection volume:**	2.000
**Analysis method:**	LCMS ISOCRATIC 60%B 0.4MLMIN-1.M	**Acq. operator:**	SYSTEM
**Last changed:**	5/8/2019 8:55:04 AM		

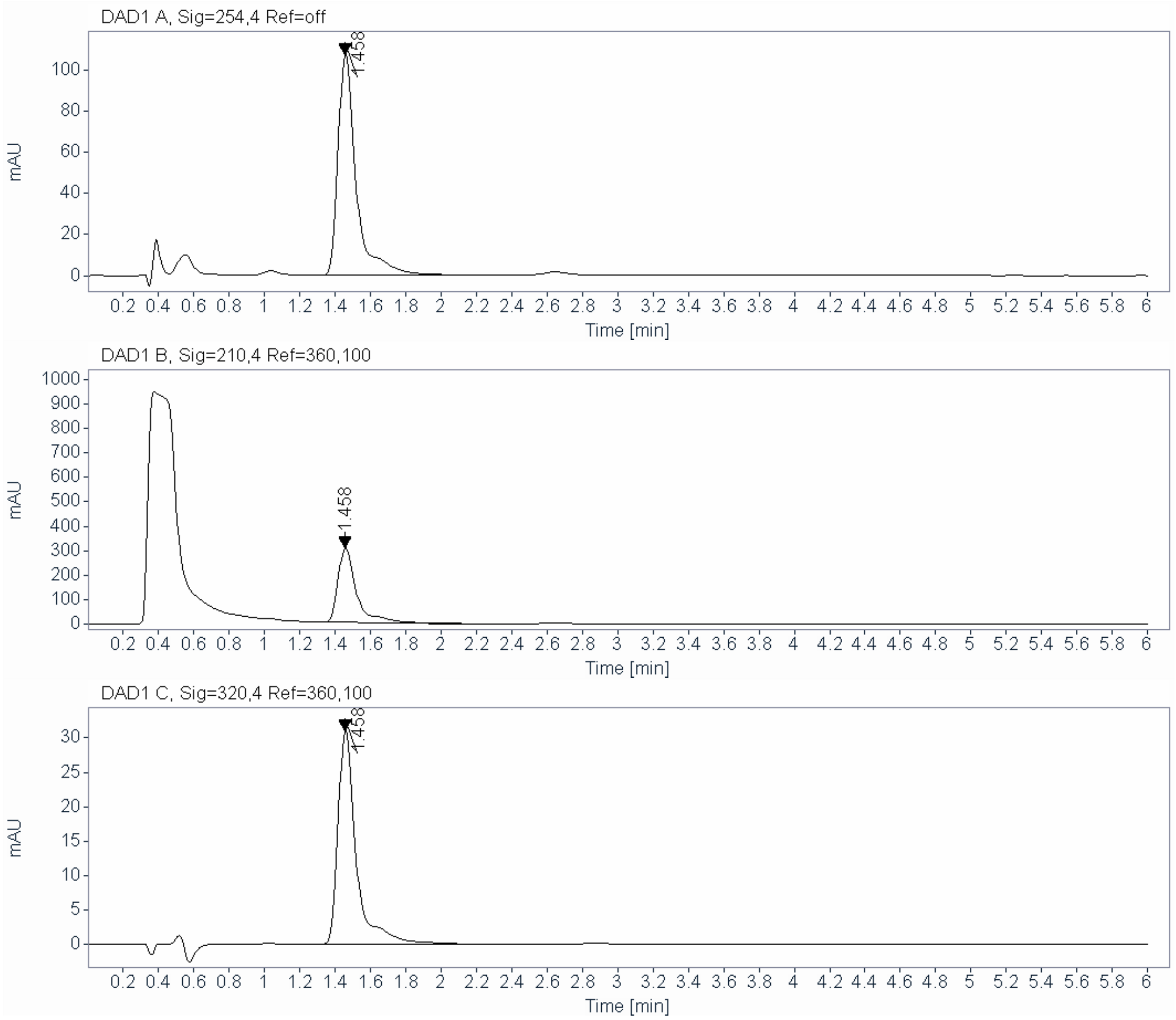

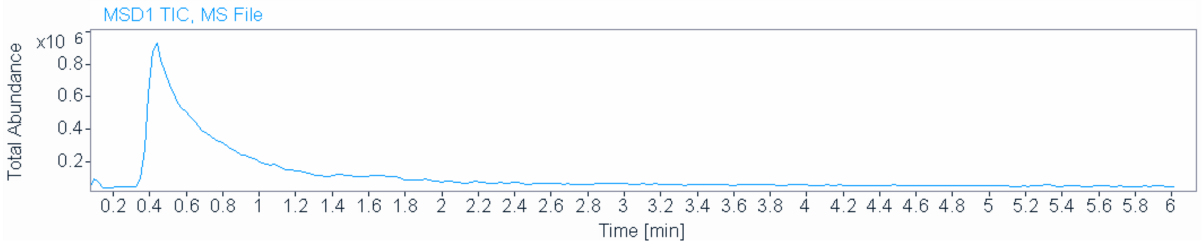

Agilent Technologies

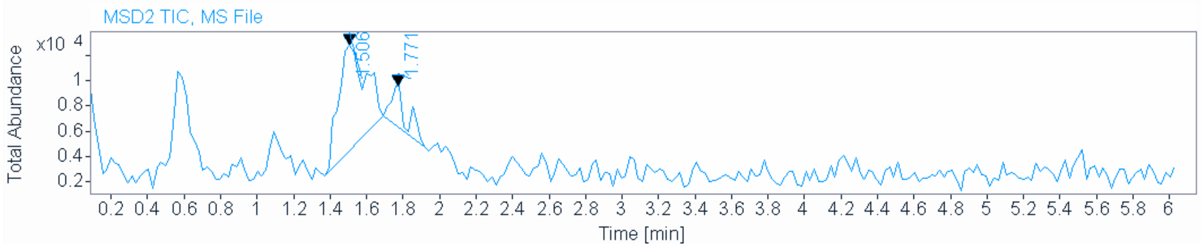

Signal: DAD1 A, Sig=254,4 Ref=off

RT [min]	Type	Width [min]	Area	Height	Area%	Name
1.458	BB	0.1079	773.2750	107.5453	100.0000	
		Sum	773.2750			

Signal: DAD1 B, Sig=210,4 Ref=360,100

RT [min]	Type	Width [min]	Area	Height	Area%	Name
1.458	BB	0.1090	2221.1716	304.7155	100.0000	
		Sum	2221.1716			

Signal: DAD1 C, Sig=320,4 Ref=360,100

RT [min]	Type	Width [min]	Area	Height	Area%	Name
1.458	BB	0.1077	223.6593	31.1881	100.0000	
		Sum	223.6593			

Signal: MSD2 TIC, MS File

RT [min]	Type	Width [min]	Area	Height	Area%	Name
1.506	BB	0.1470	84289.0234	8255.4033	81.3617	
1.771	BB	0.0763	19308.8984	3600.7041	18.6383	
		Sum	103597.9219			

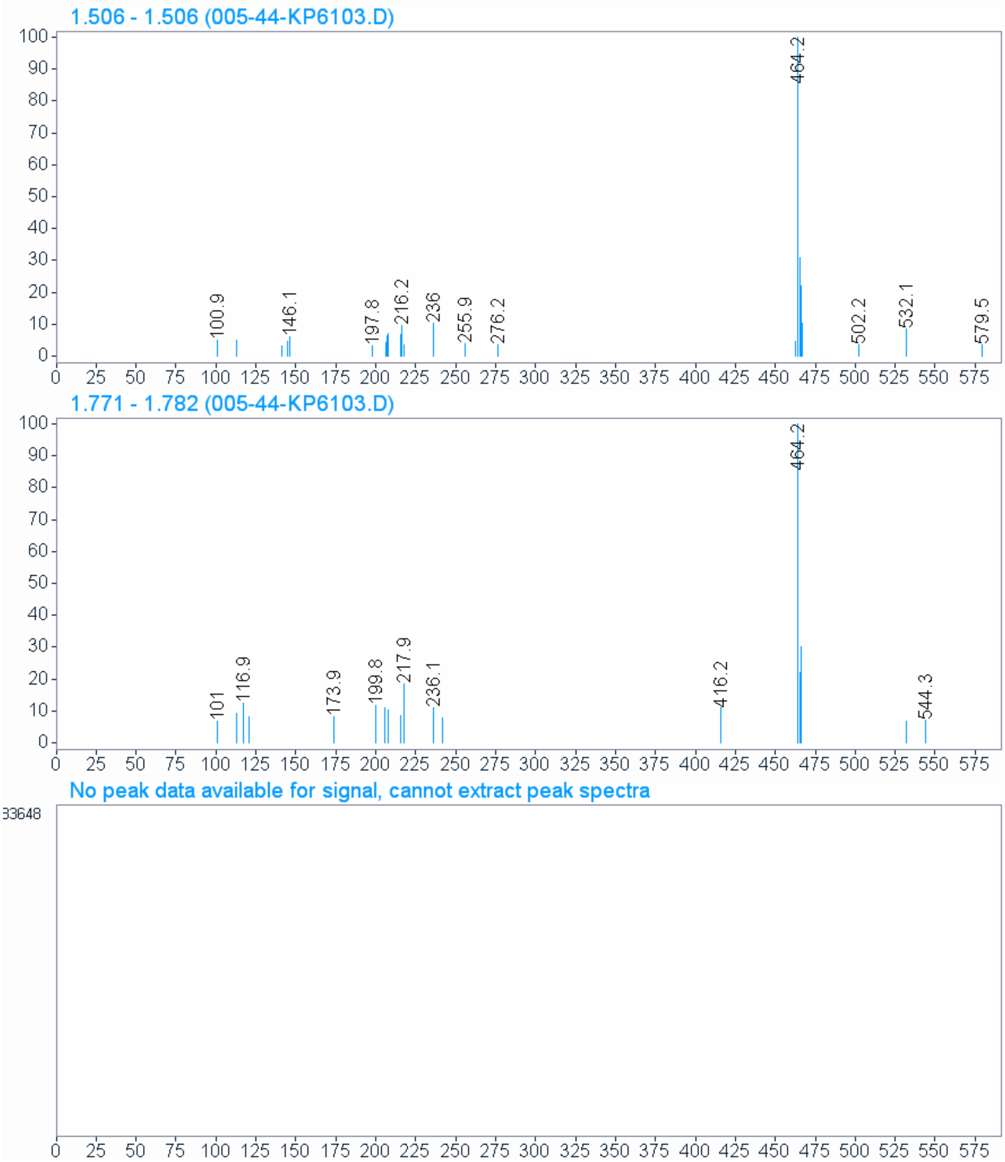
1.506 - 1.506 (005-44-KP6103.D)
464.2
100.9
146.1
197.8
216.2
236
255.9
276.2
502.2
532.1
579.5
1.771 - 1.782 (005-44-KP6103.D)
464.2
101
116.9
173.9
199.8
217.9
236.1
416.2
544.3
No peak data available for signal, cannot extract peak spectra
33648

Compound Name: (*Z*)-*N*-(5-((2,3-dihydrobenzo[b][1,4]dioxin-6-yl)methylene)-4-oxo-4,5-dihydrothiazol-2-yl)naphthalene-1-sulfonamide

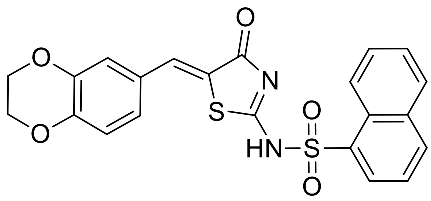

Chemical Formula: $C_{22}H_{16}N_2O_5S_2$
Exact Mass: 452.05
Molecular Weight: 452.50

Compound Code: 54 (KP6052)

Obtained Weight & Yield: 188 mg (64%)

Purity (by LCMS and ^{1}H NMR): > 99% by ^{1}H-NMR

Appearance: Orange/red powder

Solubility: DMSO, slightly soluble in acetone and methanol.

Melting Point: > 244 °C (dec.)

TLC Rf (and conditions): 0.61 (10% MeOH in DCM)

IR Analysis (including assignment): IR (neat): ν_{max} = 3056, 2926, 2771 (C-H aromatic), 1694 (C=O), 1553 (C=C aromatic), 1289 (sulfonamide), 1123 (C-N) cm^{-1}

^{1}H NMR Analysis: ^{1}H NMR (400 MHz, DMSO) δ 8.62 – 8.60 (m, 1H), 8.30 (dt, *J* = 8.3, 2.5 Hz, 2H), 8.12 (d, *J* = 8.0 Hz, 1H), 7.77 (ddd, *J* = 8.5, 6.9, 1.4 Hz, 1H), 7.72 – 7.66 (m, 3H), 7.19 – 7.16 (m, 2H), 7.08 – 7.05 (m, 1H), 4.34 – 4.30 (m, 4H) ppm.
NH exchanging – not observed. Ethanol at 1.05 ppm (0.91%)

^{13}C NMR Analysis: ^{13}C NMR (101 MHz, DMSO) δ 166.6, 165.7, 146.1, 143.8, 135.3, 134.7, 133.8, 133.7, 129.0, 128.3, 128.1, 127.6, 127.1, 126.1, 124.9, 124.7, 124.2, 119.2, 119.1, 118.2, 64.6, 64.0 ppm.

MS Analysis (low res): LRMS (ESI-) *m/z*: 451 (M-H, $C_{22}H_{15}N_2O_5S_2$, 100%)

HPLC method details: Column: Zorbax SB-C18 Rapid Resolution HT 2.1x50mm 1.8-Micron; Method: LCMS ISOCRATIC 60%B 0.4MLMIN- 1.MM filename: KP6052_60; Peak retention time: 1.49 mins; Area (%): 95.

Procedure: To a microwave vial was added the *N*-(4-oxo-4,5-dihydrothiazol-2-yl)naphthalene-1-sulfonamide (197 mg, 0.65 mmol), 2,3-dihydrobenzo[b][1,4]dioxine-6-carbaldehyde (125 mg, 0.76 mmol, 1.2 eq), ethanol (3 mL) and the benzoic acid/piperidine catalyst (approximately 10 drops). The reaction was heated by microwave irradiation (120 °C) for 40 min after cooling overnight the precipitate was collected by vacuum filtration and washed with ether to give the desired product (188 mg, 64%).

Other analyses, reference papers, previously obtained data, comments, etc:

PerkinElmer Spectrum Version 10.4.00
Monday, 27 May 2019 2:06 PM

Analyst	research
Date	Monday, 27 May 2019 2:06 PM

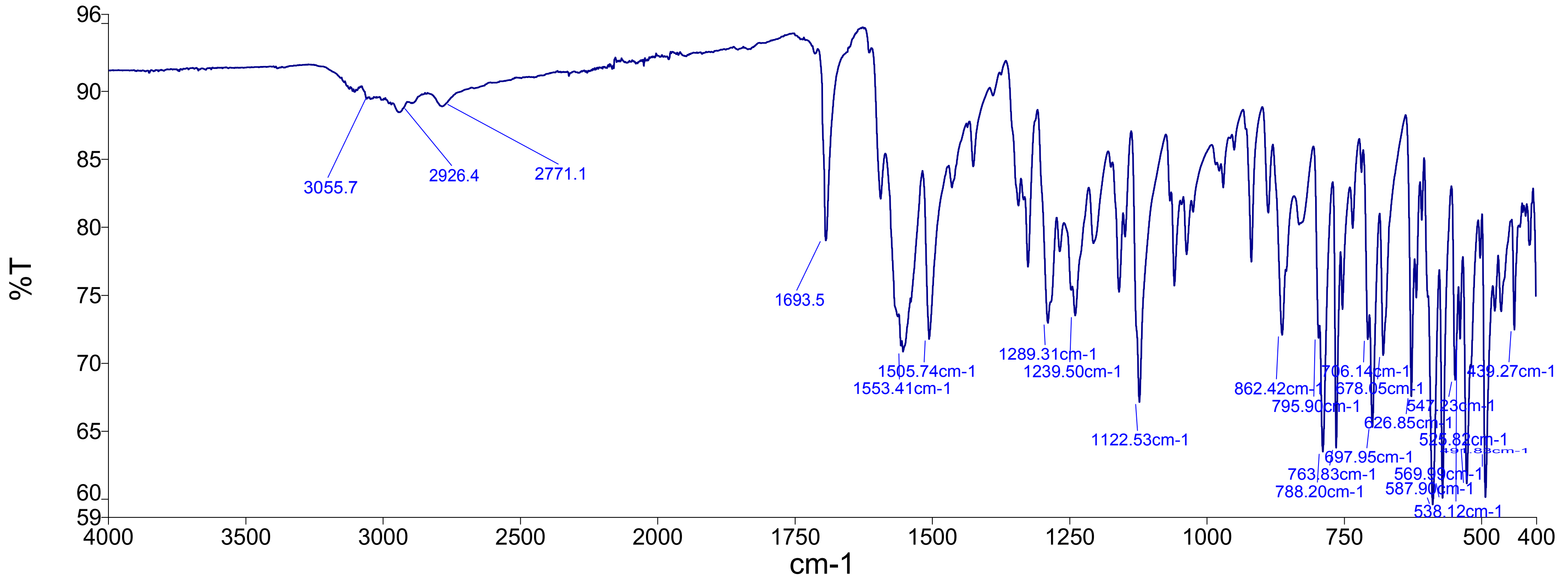

Sample Name	Description	Quality Checks
KP6053	Sample 221 By research Date Monday, May 27 2019	The Quality Checks give rise to a Weak Bands warning for the sample.

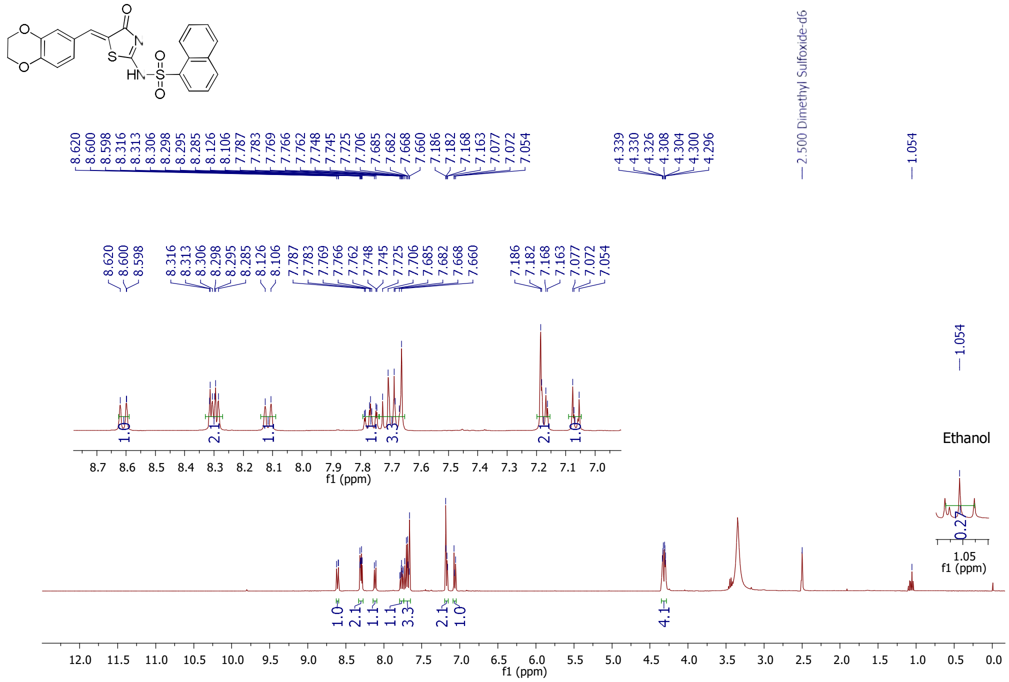

2.500 Dimethyl Sulfoxide-d6
8.620 8.600 8.598 8.316 8.313 8.306 8.298 8.295 8.285 8.126 8.106 7.787 7.783 7.769 7.766 7.762 7.748 7.745 7.725 7.706 7.685 7.682 7.668 7.660 7.186 7.182 7.168 7.163 7.077 7.072 7.054
4.339 4.330 4.326 4.308 4.304 4.300 4.296
1.054
Ethanol
0.27
1.0 2.1 1.1 1.1 3.3 2.1 1.0 4.1
f1 (ppm)

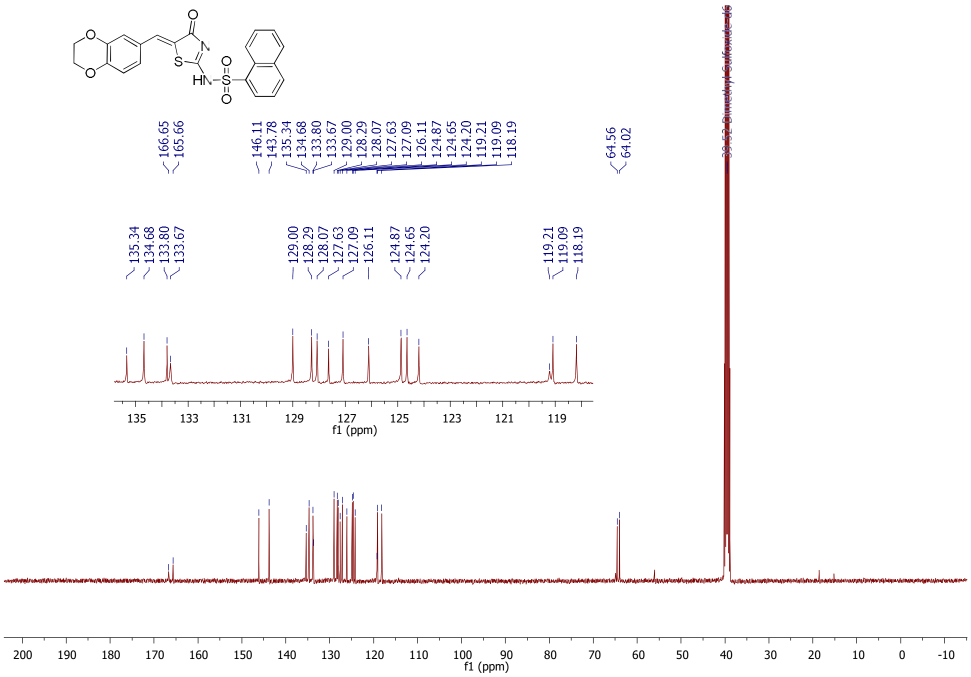

39.52 Dimethyl Sulfoxide-d6
166.65 165.66
146.11 143.78 135.34 134.68 133.80 133.67 129.00 128.29 128.07 127.63 127.09 126.11 124.87 124.65 124.20 119.21 119.09 118.19
64.56 64.02
f1 (ppm)

LCMS Report

Agilent Technologies

**Data file:**	D:\Chem32\1\Data\KP\KP5189-6052_60 2019-07-01 10-04-49\003-23-KP6052_60.D		
**Sample name:**	KP6052_60		
**Description:**			
**Sample amount:**	0.000	**Sample type:**	Sample
**Instrument:**	LCMS	**Location:**	23
**Injection date:**	7/1/2019 10:22:50 AM	**Injection:**	1 of 1
**Acq. method:**	LCMS ISOCRATIC 60% B 0.4MLMIN-1.M	**Injection volume:**	2.000
**Analysis method:**	LCMS ISOCRATIC 60%B 0.4MLMIN-1.M	**Acq. operator:**	SYSTEM
**Last changed:**	5/8/2019 8:55:04 AM		

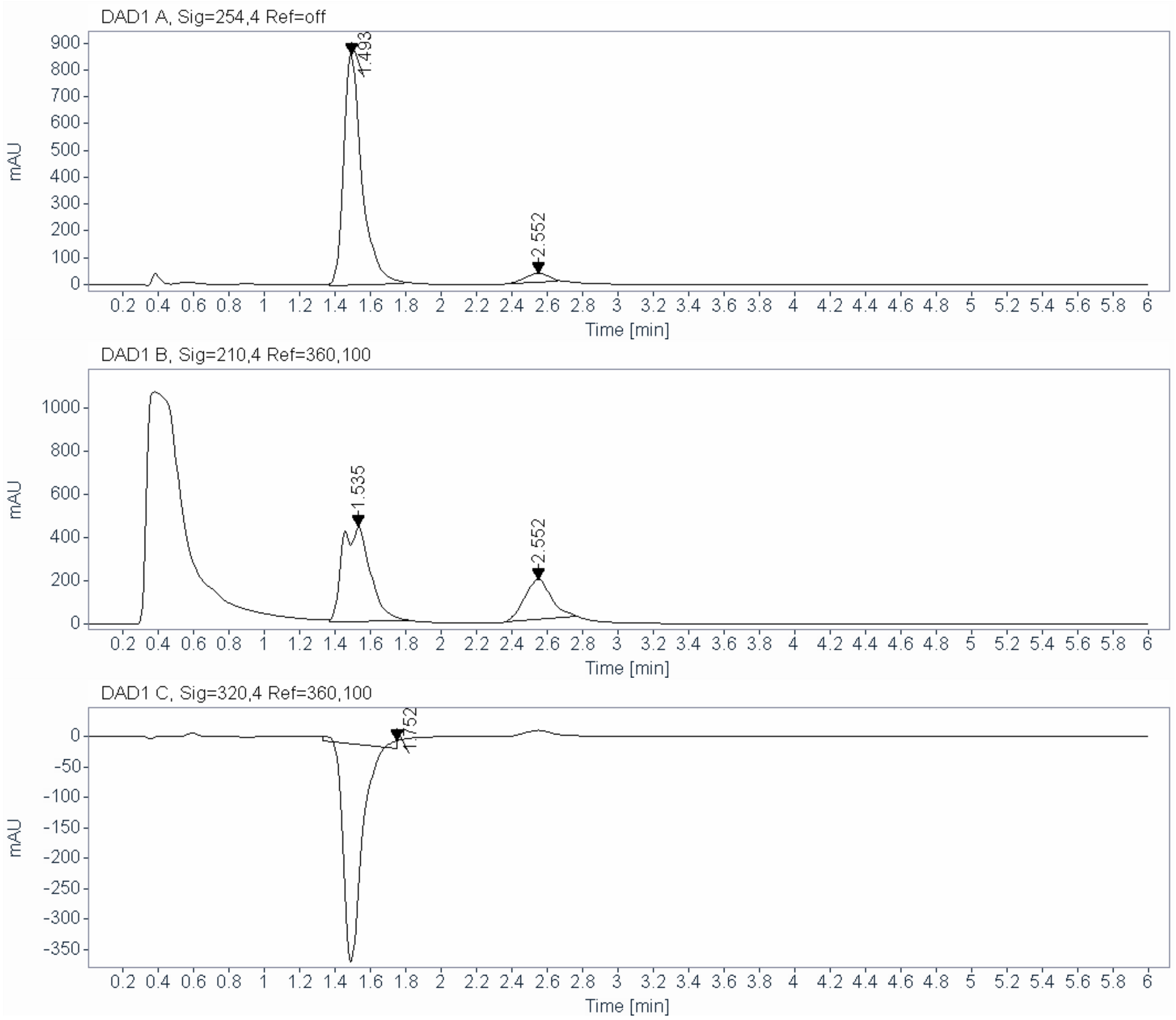

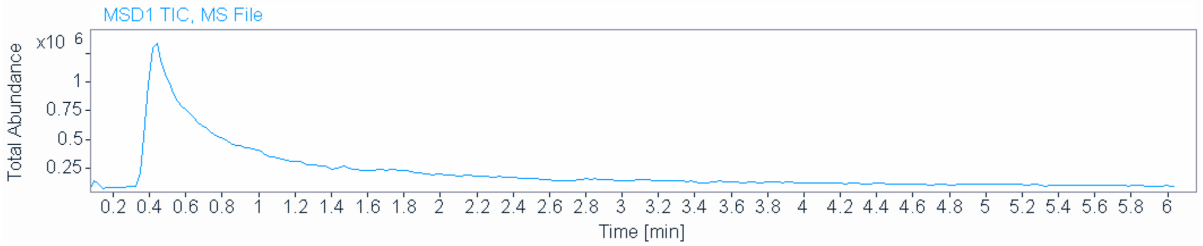

Agilent Technologies

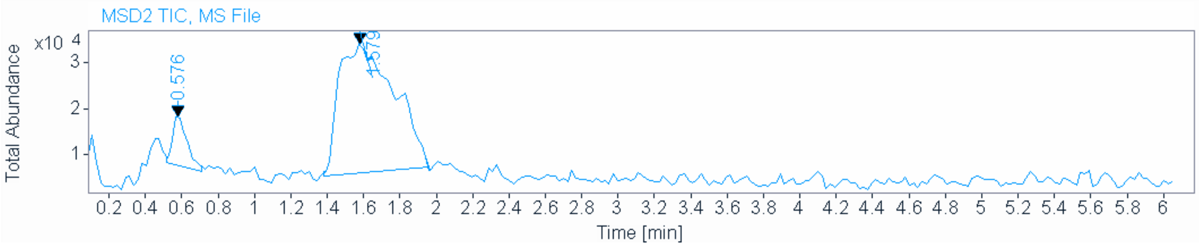

Signal: DAD1 A, Sig=254,4 Ref=off

RT [min]	Type	Width [min]	Area	Height	Area%	Name
1.493	MM	0.1198	6204.1382	862.9229	95.3484	
2.552	MM	0.1490	302.6696	33.8601	4.6516	
		Sum	6506.8078			

Signal: DAD1 B, Sig=210,4 Ref=360,100

RT [min]	Type	Width [min]	Area	Height	Area%	Name
1.535	MM	0.1847	4840.6768	436.9049	71.2407	
2.552	MM	0.1759	1954.1405	185.1774	28.7593	
		Sum	6794.8173			

Signal: DAD1 C, Sig=320,4 Ref=360,100

RT [min]	Type	Width [min]	Area	Height	Area%	Name
1.752	MM	0.0740	54.8554	12.3537	100.0000	
		Sum	54.8554			

Signal: MSD2 TIC, MS File

RT [min]	Type	Width [min]	Area	Height	Area%	Name
0.576	MM	0.0865	59073.0391	11376.0449	9.1180	
1.579	MM	0.3492	588798.8750	28101.5488	90.8820	
		Sum	647871.9141			

Agilent Technologies

0.576 - 0.566 (003-23-KP6052_60.D)

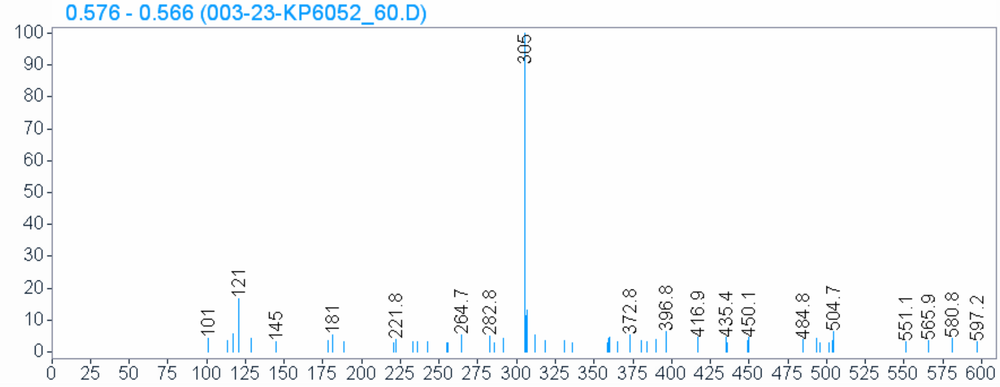

1.579 - 1.575 (003-23-KP6052_60.D)

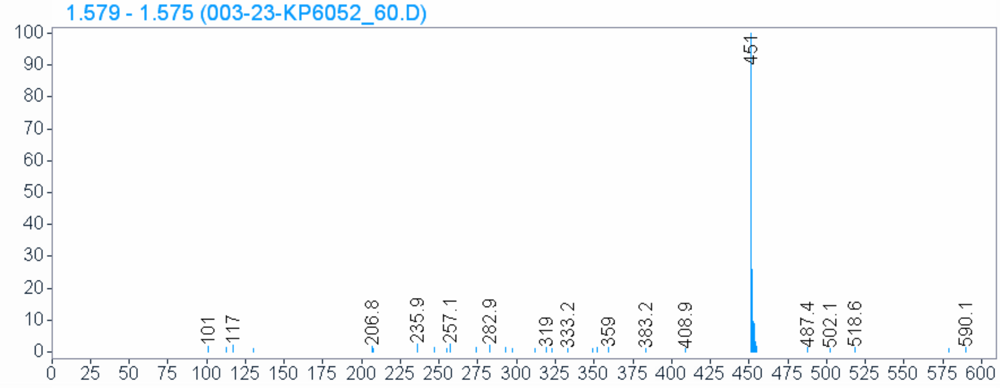

No peak data available for signal, cannot extract peak spectra

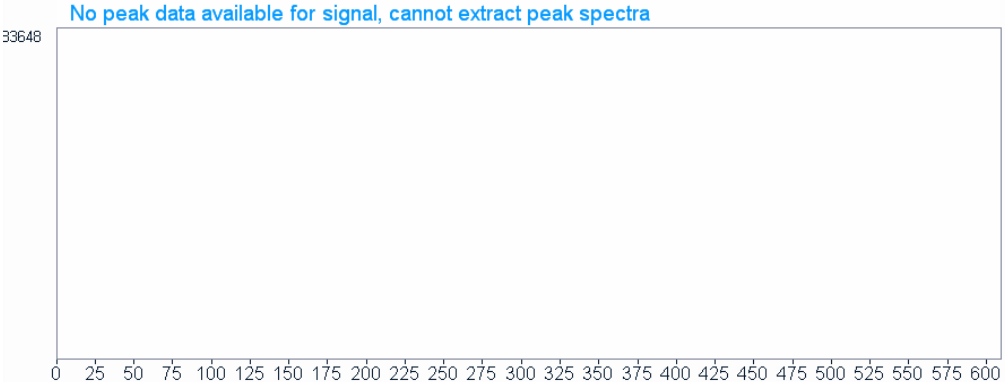

Compound Name: (Z)-*N*-(5-((2H-chromen-3-yl)methylene)-4-oxo-4,5-dihydrothiazol-2-yl)naphthalene-1-sulfonamide

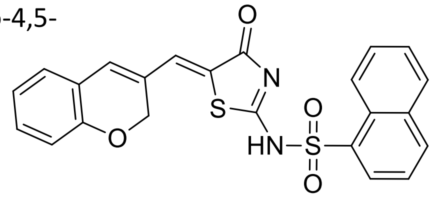

Chemical Formula: $C_{23}H_{16}N_2O_4S_2$
Exact Mass: 448.06
Molecular Weight: 448.51

Compound Code: 55 (KP6105)

Obtained Weight & Yield: 58 mg, 26%

Purity (by LCMS and ^{1}H NMR): 99% by ^{1}H-NMR and LCMS

Appearance: orange/red solid

Solubility: DMSO, slightly soluble in methanol and acetone

Melting Point: > 237 °C (dec.)

TLC Rf (and conditions): N/A

IR Analysis (including assignment): IR (neat): ν_{max} = 3114 (NH), 2939 (aromatic C-H), 2784 (C-H), 1690 (C=O), 1551 (aromatic C-C), 1350 (sulfonamide), 1157 (C-O-C), 1127 (C-N) cm^{-1}

^{1}H NMR Analysis: ^{1}H NMR (400 MHz, DMSO) δ 8.60 (d, *J* = 8.5 Hz, 1H), 8.31 (d, *J* = 7.5 Hz, 2H), 8.12 (d, *J* = 8.0 Hz, 1H), 7.79 – 7.75 (m, 1H), 7.73 – 7.67 (m, 2H), 7.32 (s, 1H), 7.28 – 7.24 (m, 2H), 7.17 (s, 1H), 6.97 (td, *J* = 7.5, 1.0 Hz, 1H), 6.87 – 6.85 (m, 1H), 5.12 (s, 2H) ppm.
NH exchanging (not observed). Ethanol at 1.06 ppm (0.68%)

^{13}C NMR Analysis: ^{13}C DEPTQ NMR (151 MHz, DMSO) δ 166.4, 165.0, 153.6, 135.2, 134.7, 133.8, 133.0, 131.8, 130.9, 129.0, 128.6, 128.3, 128.3, 127.9, 127.6, 127.1, 124.9, 124.7, 122.6, 121.9, 121.0, 115.7, 65.7 ppm.

MS Analysis (low res): LRMS (ESI-) *m/z* (%): 447 (*M*-H, $C_{23}H_{15}N_2O_4S_2$, 100%)

MS Analysis (high res): Exact mass calculated for $C_{23}H_{15}N_2O_4S_2$ [*M*-H]$^-$, 447.0500. Found 447.0478.

HPLC method details: Column: Zorbax SB-C18 Rapid Resolution HT 2.1x50mm 1.8-Micron; Method: LCMS ISOCRATIC 60%B 0.4MLMIN-1.M filename: KP6105; Peak retention time: 2.155 mins; Area (%): 99.

Procedure: To a 10 mL microwave vial was added *N*-(4-oxo-4,5-dihydrothiazol-2-yl)naphthalene-1-sulfonamide (149 mg, 0.49 mmol), 2-H-chromemo-3-carboxaldehyde (87 mg, 0.54 mmol, 1.1 eq), ethanol (3 mL) and a catalytic amount of the benzoic acid/piperidine catalyst (approximately 2 drops). The suspension was heated using microwave irradiation (200 W, 120 °C) for 110 min then allowed to precipitate at room temperature. The resulting precipitate was collected by vacuum filtration and washed with cold ethanol and cold ether to give the desired product (58 mg, 26%)

Other analyses, reference papers, previously obtained data, comments, etc:

PerkinElmer Spectrum Version 10.4.00
Tuesday, 23 July 2019 11:47 AM

Analyst	research
Date	Tuesday, 23 July 2019 11:47 AM

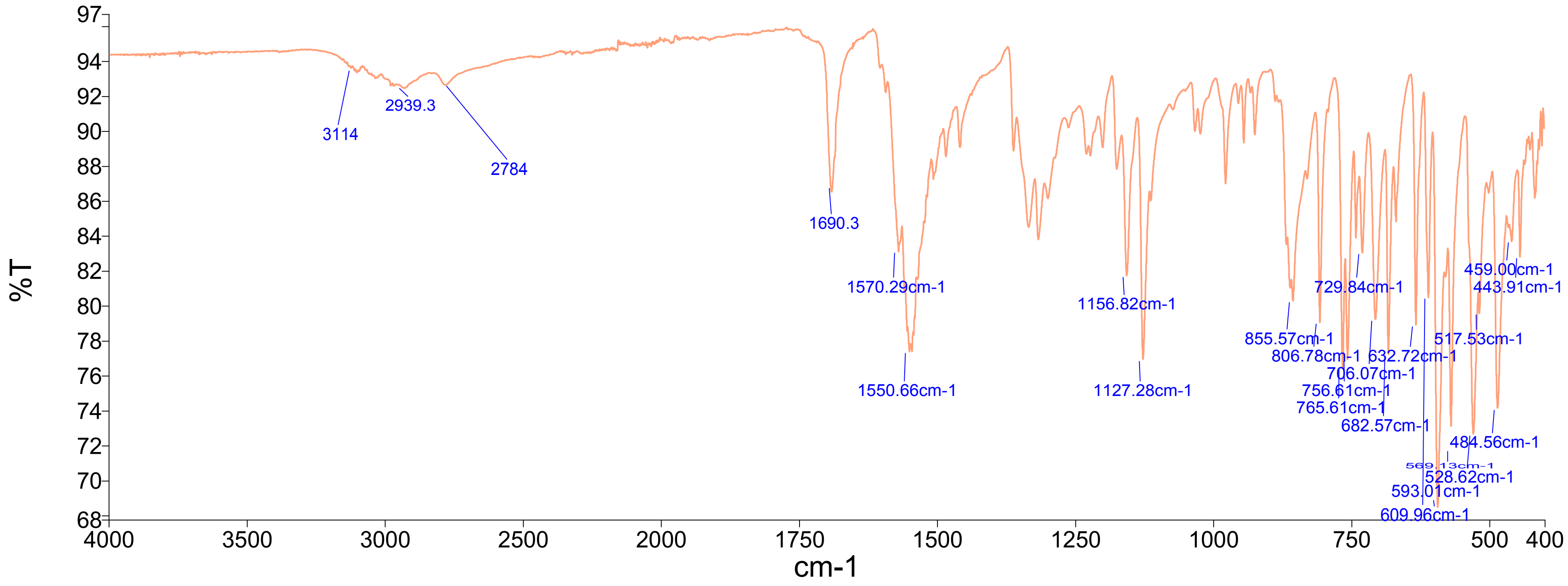

Sample Name	Description	Quality Checks
KP6105	Sample 237 By research Date Tuesday, July 23 2019	The Quality Checks give rise to a Weak Bands warning for the sample.

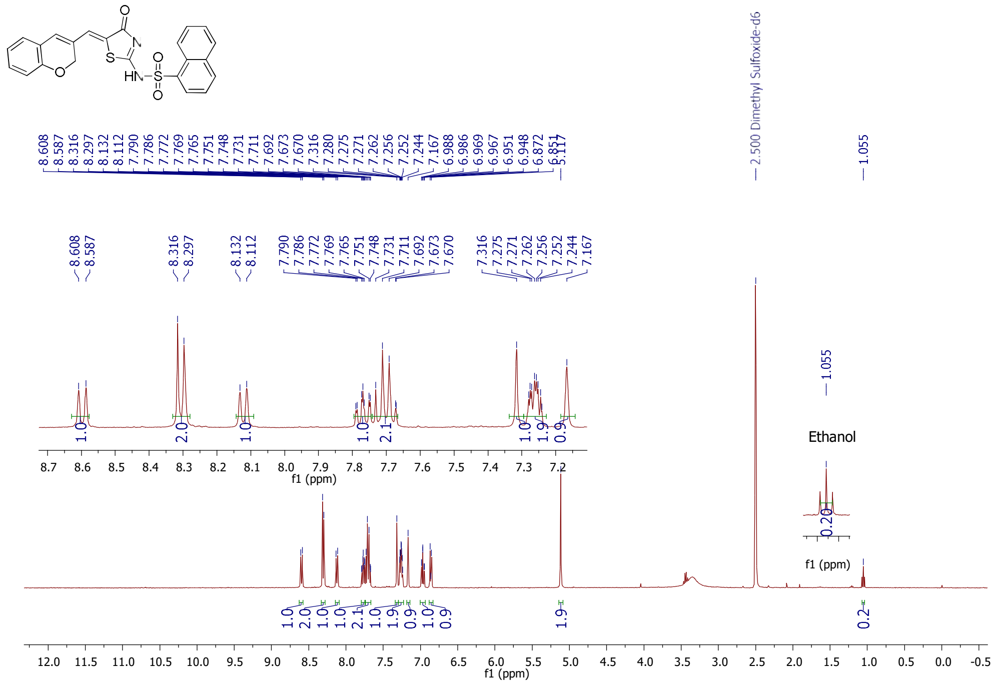

8.608
8.587
8.316
8.297
8.132
8.112
7.790
7.786
7.772
7.769
7.765
7.751
7.748
7.731
7.711
7.692
7.673
7.670
7.316
7.280
7.275
7.271
7.262
7.256
7.252
7.244
7.167
6.988
6.986
6.969
6.967
6.951
6.948
6.872
6.851
5.117
2.500 Dimethyl Sulfoxide-d6
1.055
Ethanol
f1 (ppm)

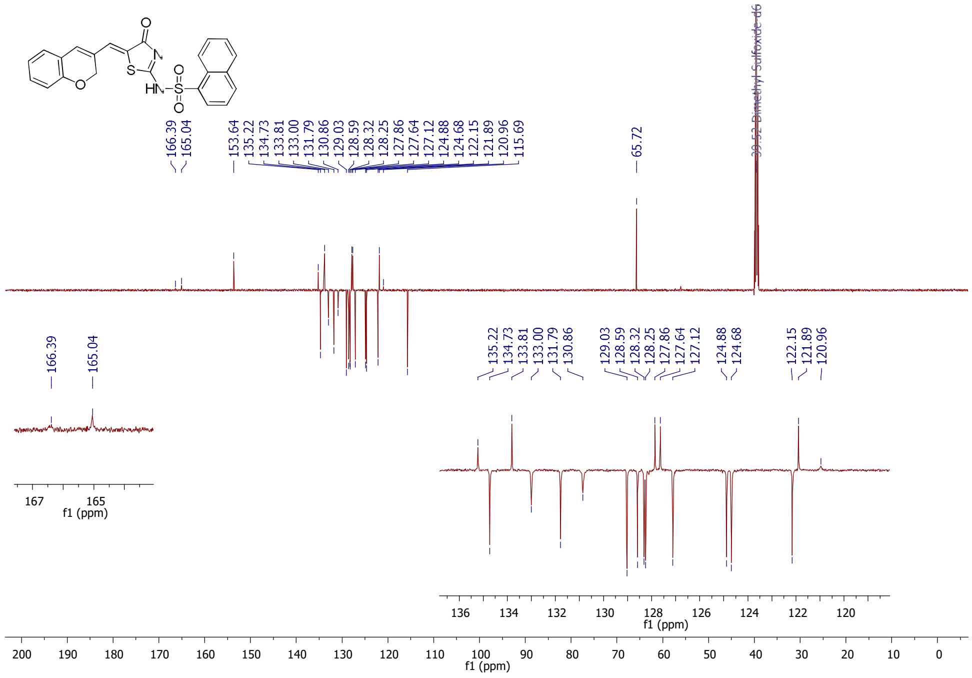

166.39
165.04
153.64
135.22
134.73
133.81
133.00
131.79
130.86
129.03
128.59
128.32
128.25
127.86
127.64
127.12
124.88
124.68
122.15
121.89
120.96
115.69
65.72
39.52 Dimethyl Sulfoxide-d6
f1 (ppm)

LCMS Report

Agilent Technologies

**Data file:**	D:\Chem32\1\Data\KP\KP_DS_11NOV 2019-11-11 14-45-23\006-45-KP6105.D		
**Sample name:**	KP6105		
**Description:**			
**Sample amount:**	0.000	**Sample type:**	Sample
**Instrument:**	LCMS	**Location:**	45
**Injection date:**	11/11/2019 3:26:19 PM	**Injection:**	1 of 1
**Acq. method:**	LCMS ISOCRATIC 60% B 0.4MLMIN-1.M	**Injection volume:**	2.000
**Analysis method:**	LCMS ISOCRATIC 60%B 0.4MLMIN-1.M	**Acq. operator:**	SYSTEM
**Last changed:**	5/8/2019 8:55:04 AM		

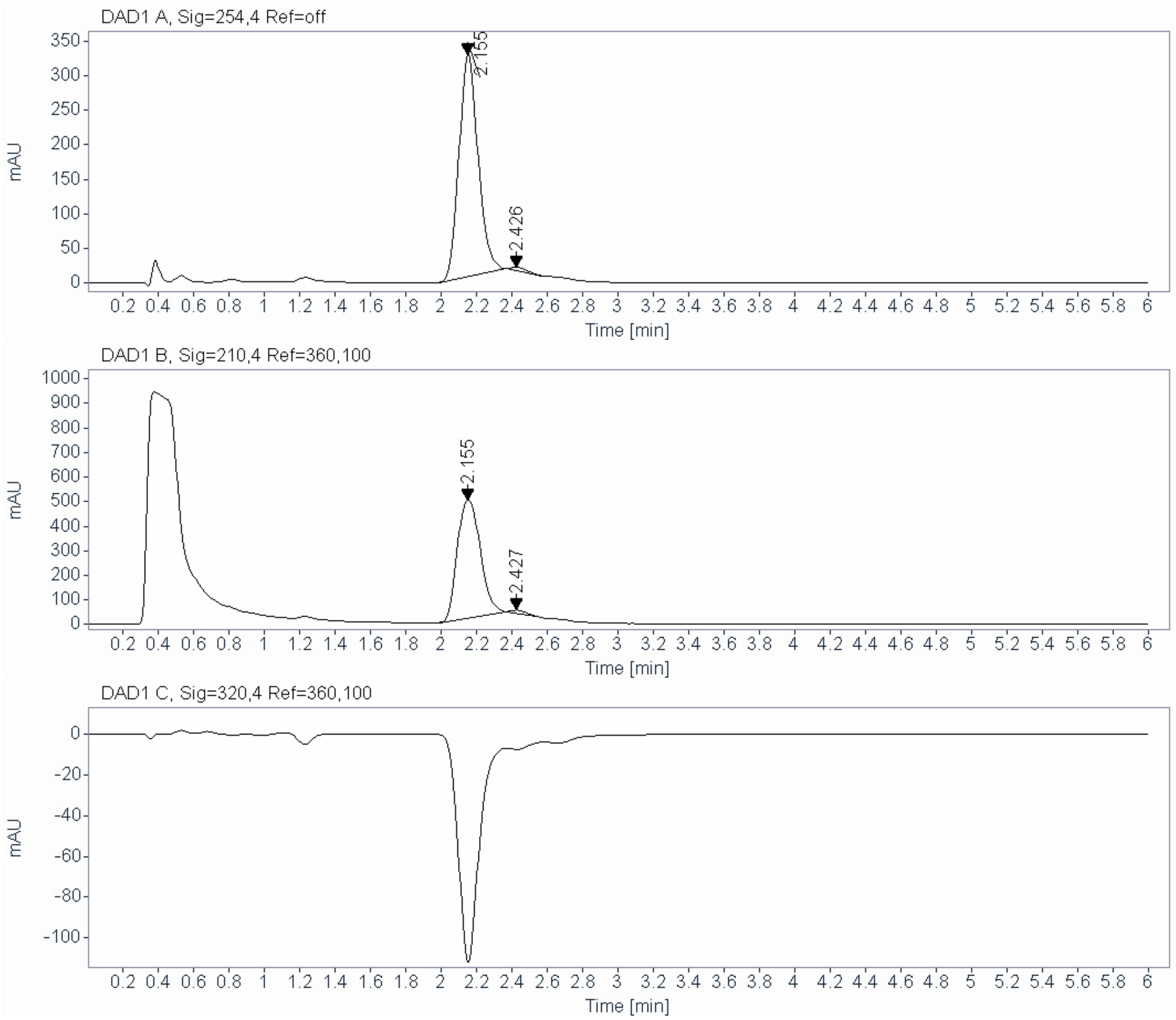

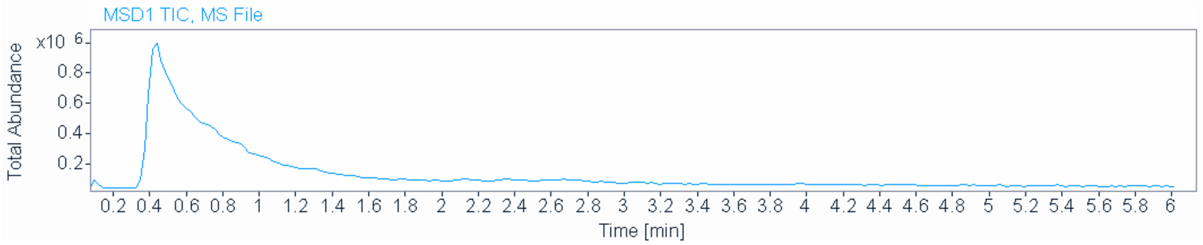

LCMS Report

Agilent Technologies

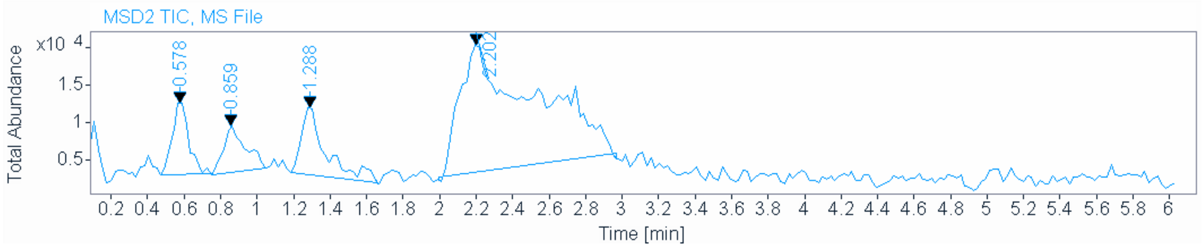

Signal: DAD1 A, Sig=254,4 Ref=off

RT [min]	Type	Width [min]	Area	Height	Area%	Name
2.155	BB	0.1156	2415.7839	321.8735	98.7084	
2.426	BB	0.1039	31.6100	4.8612	1.2916	
		Sum	2447.3939			

Signal: DAD1 B, Sig=210,4 Ref=360,100

RT [min]	Type	Width [min]	Area	Height	Area%	Name
2.155	BB	0.1433	4317.8467	478.4319	98.1395	
2.427	BB	0.1025	81.8550	12.4847	1.8605	
		Sum	4399.7017			

Signal: MSD2 TIC, MS File

RT [min]	Type	Width [min]	Area	Height	Area%	Name
0.578	BB	0.0923	60708.8164	9878.5195	9.0303	
0.859	BB	0.1115	51566.0938	6155.5669	7.6704	
1.288	BB	0.1349	84436.6016	9172.2080	12.5598	
2.202	MM	0.4643	475564.0625	17071.4609	70.7395	
		Sum	672275.5742			

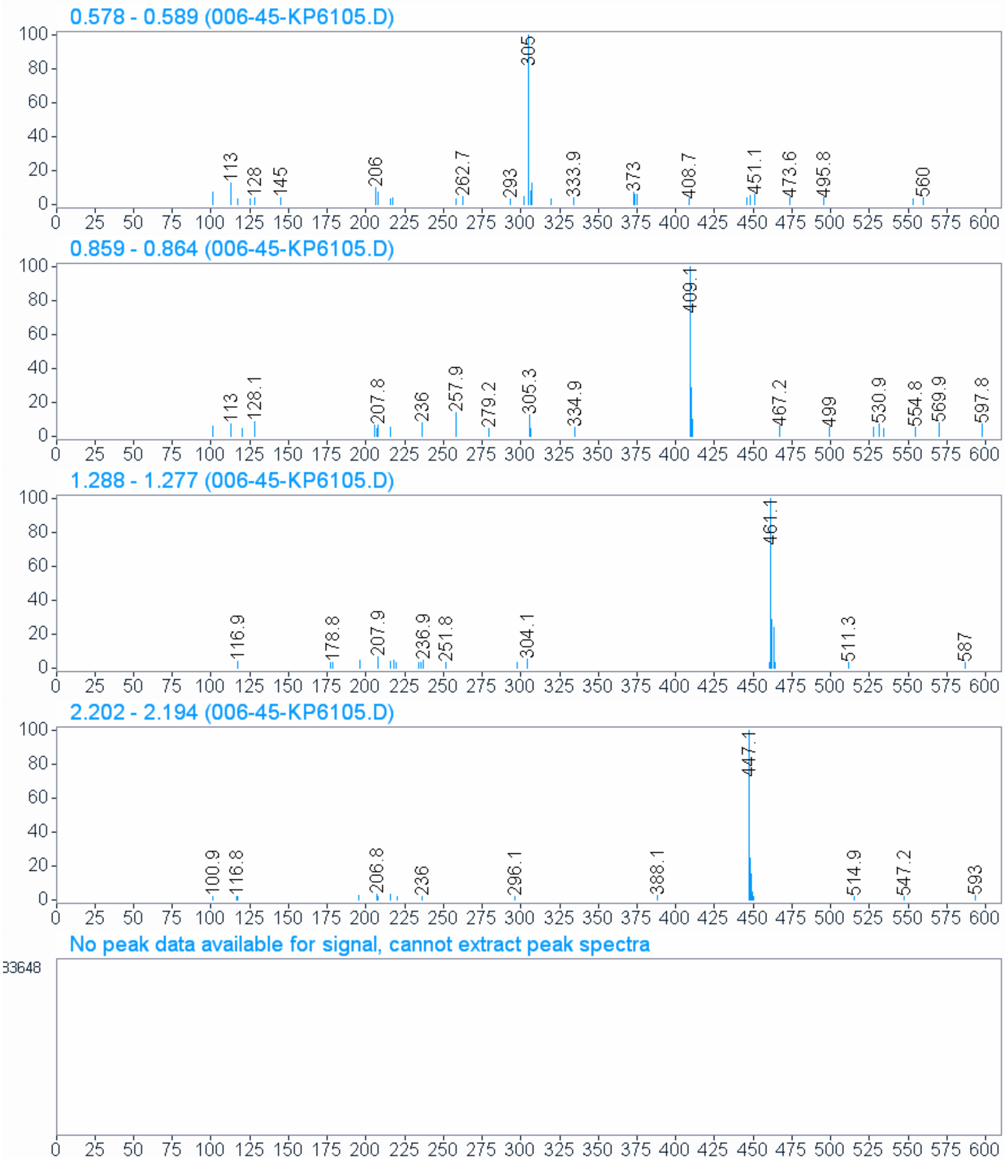
0.578 - 0.589 (006-45-KP6105.D)
0.859 - 0.864 (006-45-KP6105.D)
1.288 - 1.277 (006-45-KP6105.D)
2.202 - 2.194 (006-45-KP6105.D)
No peak data available for signal, cannot extract peak spectra

Compound Name: (Z)-*N*-(5-(benzo[d][1,3]dioxol-5-ylmethylene)-4-oxo-4,5-dihydrothiazol-2-yl)naphthalene-1-sulfonamide

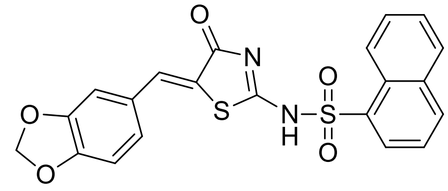

Chemical Formula: $C_{21}H_{14}N_2O_5S_2$
Exact Mass: 438.03
Molecular Weight: 438.48

Compound Code: 56 (KP6095)

Obtained Weight & Yield: 151 mg (70%)

Purity (by LCMS and ^{1}H NMR): > 98% by ^{1}H-NMR

Appearance: yellow powder

Solubility: DMSO, slightly soluble in acetone

Melting Point: > 274 °C (dec.)

TLC Rf (and conditions): 0.46 (10% MeOH in DCM)

IR Analysis (including assignment): IR (neat): ν_{max} = 3066 (NH), 2948 (aromatic C-H), 2791 (C-H), 1708 (C=O), 1562 (aromatic C-C), 1300 (sulfonamide), 1158 (C-O-C), 1123 (C-N) cm^{-1}

^{1}H NMR Analysis: ^{1}H NMR (400 MHz, DMSO) δ 13.16 (br, s, 1H, NH), 8.60 (d, *J* = 8.6 Hz, 1H), 8.31 (d, *J* = 7.7 Hz, 2H), 8.12 (d, *J* = 8.1 Hz, 1H), 7.79 – 7.75 (m, 1H), 7.73 – 7.67 (m, 2H), 7.61 (d, *J* = 1.2 Hz, 1H), 7.10 – 7.03 (m, 3H), 6.18 (d, *J* = 1.0 Hz, 2H) ppm.
Starting material at 4.04 ppm (0.69%) and acetone at 2.09 ppm (1.05%)

^{13}C NMR Analysis: ^{13}C NMR (101 MHz, DMSO) δ 166.4, 165.5, 147.8, 147.3, 135.2, 134.8, 133.8, 129.0, 128.3, 128.2, 127.6, 127.1, 125.6, 124.8, 124.6, 122.83, 122.78, 120.4, 114.8, 110.8, 102.0 ppm.

MS Analysis (low res): LRMS (ESI-) *m/z*: 437 (*M*-H, $C_{21}H_{13}N_2O_5S_2$, 100%)

HPLC method details: Column: Zorbax SB-C18 Rapid Resolution HT 2.1x50mm 1.8-Micron; Method; LCMS ISOCRATIC 60%B 0.4MLMIN-1.M filename: KP6085; Peak retention time: 1.52 mins; Area (%): 100.
NOTE: this sample was entered in under the wrong code (KP6085 instead of KP6095)

Procedure: To a microwave vial was added the sulfonamide intermediate (152 mg, 0.49 mmol), benzo[d][1,3]dioxole-5-carbaldehyde (83 mg, 0.54 mmol, 1.1 eq), ethanol (3 mL) and the benzoic acid/piperidine catalyst (3 drops). The reaction mixture was treated with microwave irradiation (120°C, 30 min). After cooling, a precipitate was collected by vacuum filtration. The precipitate was washed with H_2O (2 mL), cold ethanol (5 mL) and cold diethyl ether (10 mL) to give the desired product as an orange powder (151 mg, 70%).

Other analyses, reference papers, previously obtained data, comments, etc:

Analyst	analyst1
Date	Thursday, 4 July 2019 12:53 PM

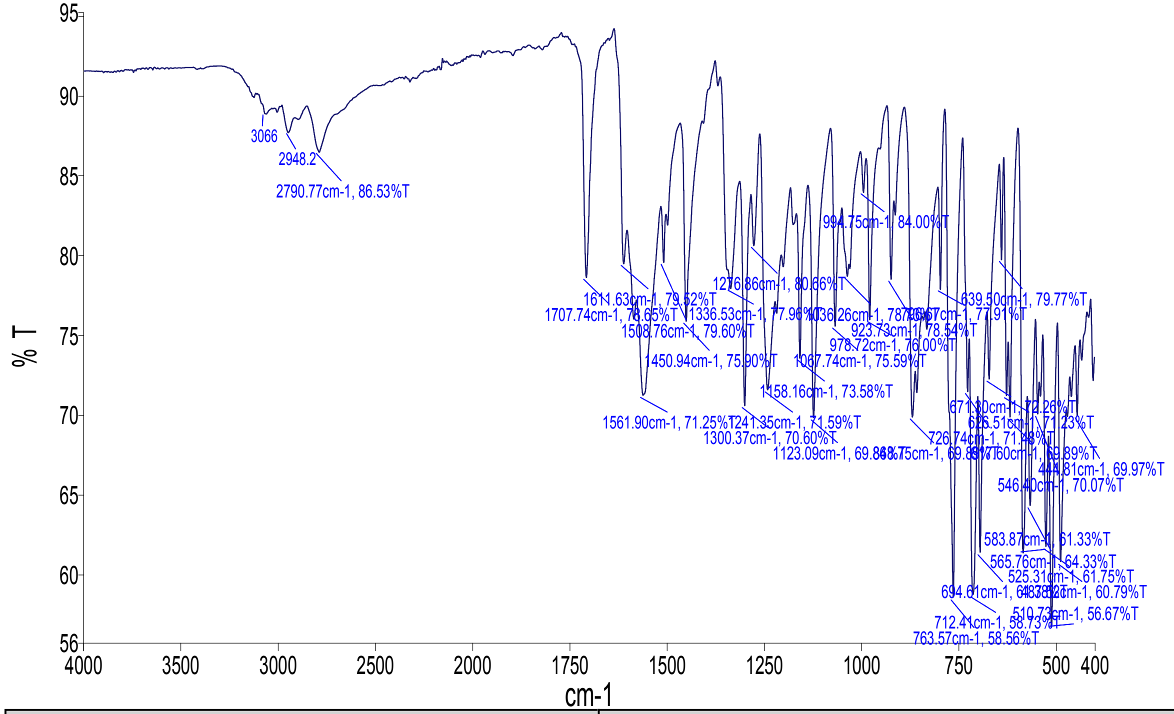

Sample Name	Description	Quality Checks
KP6095	Sample 017 By Analyst1 Date Thursday, July 04 2019	The Quality Checks give rise to a Weak Bands warning for the sample.

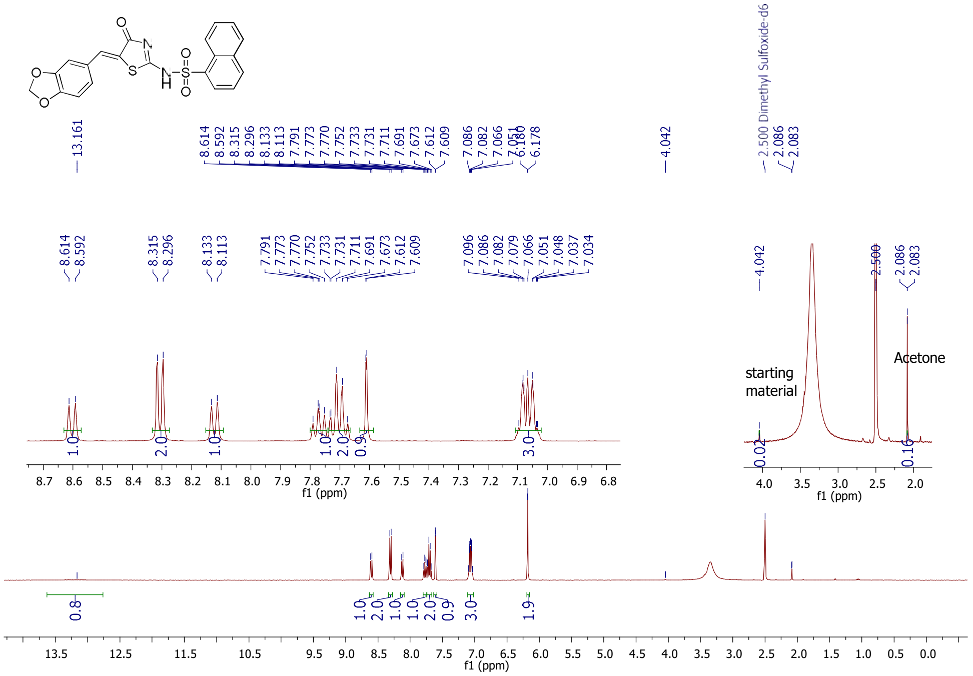

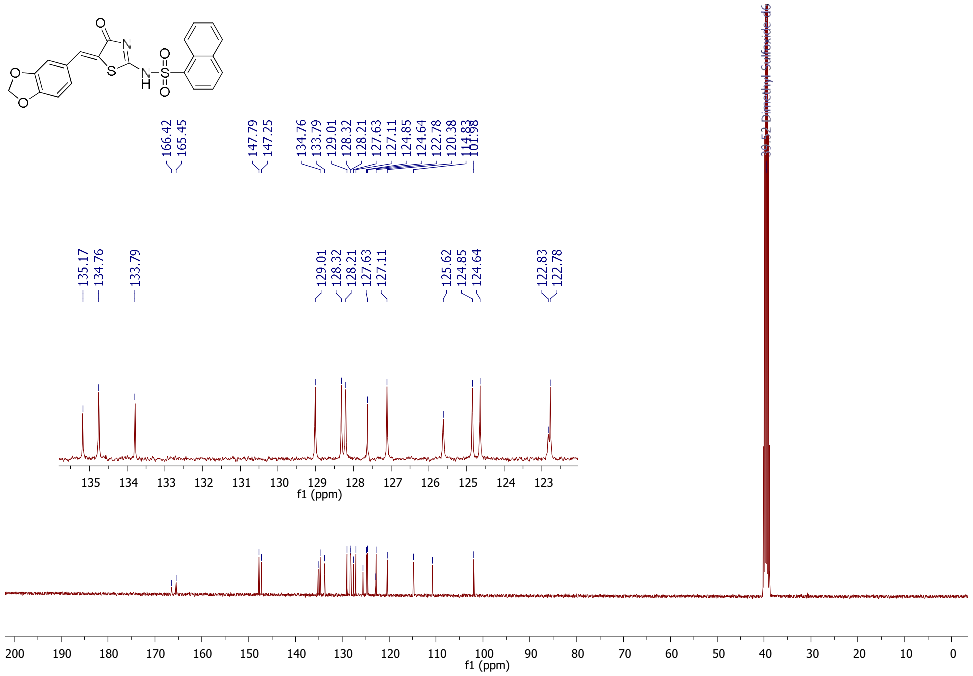

LCMS Report

Agilent Technologies

**Data file:**	D:\Chem32\1\Data\KP\KP60838485 2019-08-13 14-20-02\004-49-KP6085.D		
**Sample name:**	KP6085		
**Description:**			
**Sample amount:**	0.000	**Sample type:**	Sample
**Instrument:**	LCMS	**Location:**	49
**Injection date:**	8/13/2019 2:44:33 PM	**Injection:**	1 of 1
**Acq. method:**	LCMS ISOCRATIC 60% B 0.4MLMIN-1.M	**Injection volume:**	2.000
**Analysis method:**	LCMS ISOCRATIC 60%B 0.4MLMIN-1.M	**Acq. operator:**	SYSTEM
**Last changed:**	5/8/2019 8:55:04 AM		

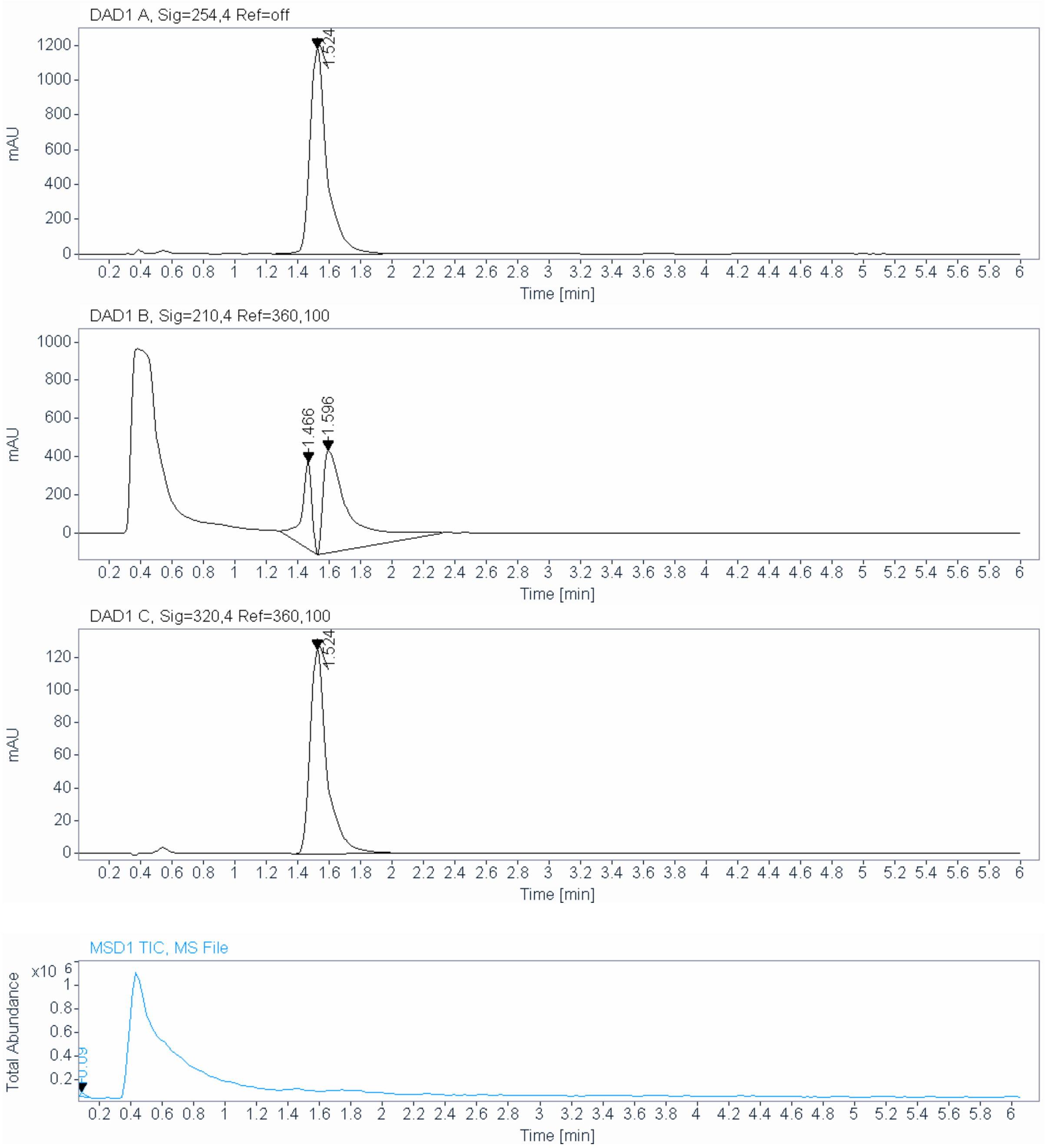

Agilent Technologies

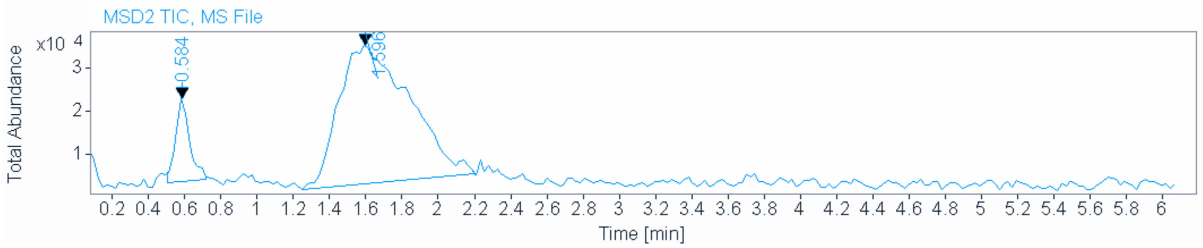

Signal: DAD1 A, Sig=254,4 Ref=off

RT [min]	Type	Width [min]	Area	Height	Area%	Name
1.524	BB	0.1117	8904.1514	1184.9817	100.0000	
		Sum	8904.1514			

Signal: DAD1 B, Sig=210,4 Ref=360,100

RT [min]	Type	Width [min]	Area	Height	Area%	Name
1.466	BB	0.0658	2032.1630	457.3785	25.0245	
1.596	BB	0.1669	6088.5234	534.6782	74.9755	
		Sum	8120.6864			

Signal: DAD1 C, Sig=320,4 Ref=360,100

RT [min]	Type	Width [min]	Area	Height	Area%	Name
1.524	BB	0.1083	927.8543	125.4219	100.0000	
		Sum	927.8543			

Signal: MSD1 TIC, MS File

RT [min]	Type	Width [min]	Area	Height	Area%	Name
0.090	BB	0.0327	79149.9297	40379.2188	100.0000	
		Sum	79149.9297			

Signal: MSD2 TIC, MS File

RT [min]	Type	Width [min]	Area	Height	Area%	Name
0.584	MM	0.0875	102779.2578	19567.3848	10.5785	
1.596	BB	0.3574	868808.7500	32061.6914	89.4215	
		Sum	971588.0078			

Agilent Technologies

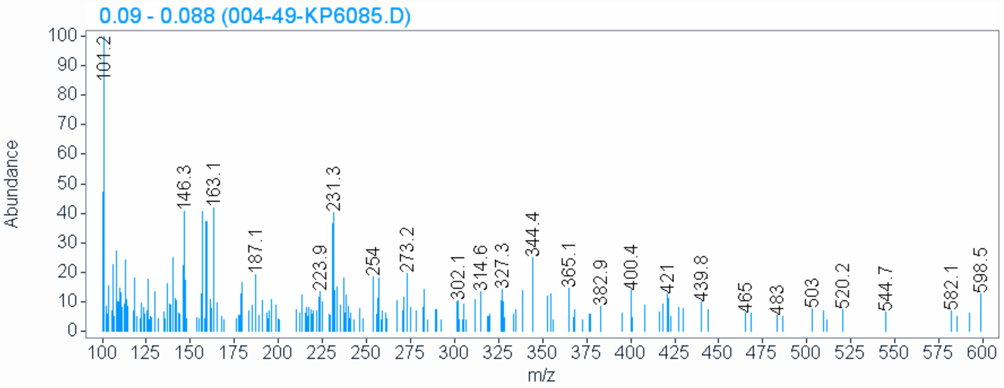

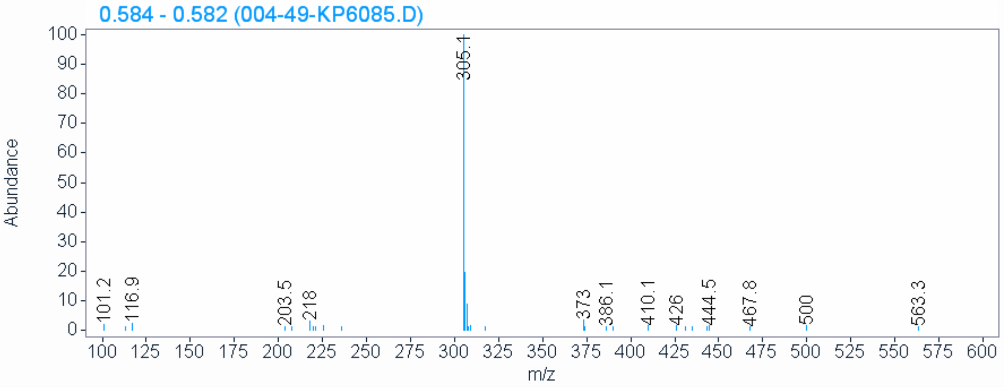

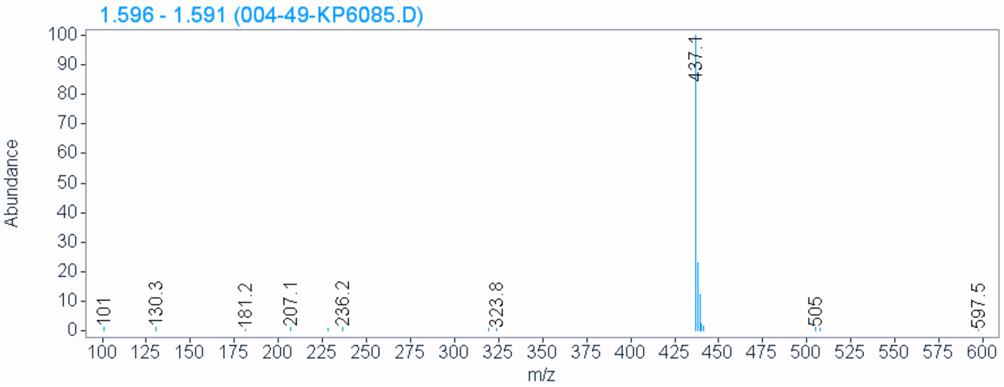

Compound Name: (Z)-*N*-(5-((2,3-dihydrobenzofuran-5-yl)methylene)-4-oxo-4,5-dihydrothiazol-2-yl)naphthalene-1-sulfonamide

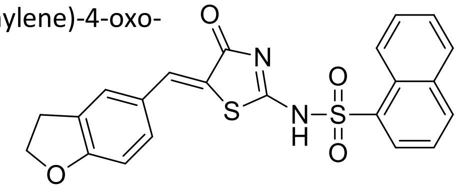

Chemical Formula: $C_{22}H_{16}N_2O_4S_2$
Exact Mass: 436.06
Molecular Weight: 436.50

Compound Code: 57 (KP6041)

Obtained Weight & Yield: 87 mg (61%).

Purity (by LCMS and ^{1}H NMR): > 99% by ^{1}H-NMR

Appearance: Light orange/pink powder

Solubility: DMSO, slightly soluble in ethyl acetate, acetone, ethanol and methanol.

Melting Point: > 259 °C (dec.)

TLC Rf (and conditions): 0.57 (10% MeOH in DCM)

IR Analysis (including assignment): IR (neat): ν_{max} = 3114, 2972, 2778 (C-H aromatic), 1684 (C=O), 1549 (C-C aromatic), 1489 (C-H), 1157 (C-O), 1127 (C-N) cm^{-1}

^{1}H NMR Analysis: ^{1}H NMR (400 MHz, DMSO) δ 8.60 (d, *J* = 8.7 Hz, 1H), 8.31 – 8.28 (m, 2H), 8.12 (d, *J* = 8.1 Hz, 1H), 7.75 (dd, *J* = 8.5, 1.4 Hz, 1H), 7.72 – 7.68 (m, 3H), 7.54 (s, 1H), 7.47 (dd, *J* = 8.4, 1.7 Hz, 1H), 6.99 (d, *J* = 8.4 Hz, 1H), 4.65 (t, *J* = 8.7 Hz, 2H) ppm.
NH exchanging – not visible. 2H at 3.00ppm under water peak – confirmed by 2D NMR.
Ether impurity at 1.09 ppm (1.09%)

^{13}C NMR Analysis: ^{13}C NMR (101 MHz, DMSO) δ 167.7, 162.4, 135.5, 134.6, 134.2, 133.8, 132.2, 129.4, 129.0, 128.2, 128.0, 127.6, 127.3, 127.1, 125.4, 124.9, 124.6, 110.1, 72.1, 28.6 ppm.
2D NMR confirms broad alkene CH at 134.2 ppm
2 quaternary carbons missing

MS Analysis (low res): LRMS (ESI-) *m/z*: 435 (M-H, $C_{22}H_{15}N_2O_4S_2$, 100%), (ESI+) *m/z*: 437 (M+H, $C_{22}H_{17}N_2O_4S_2$, 100%)

HPLC method details: Column: Zorbax SB-C18 Rapid Resolution HT 2.1x50mm 1.8-Micron; Method: LCMS ISOCRATIC 50% B.M_REDUCED FLOW.M filename: KP6041; Peak retention time: 3.38 mins; Area (%): 98.

Procedure: To a 10 mL microwave vial was added the *N*-(4-oxo-4,5-dihydrothiazol-2-yl)naphthalene-1-sulfonamide (100 mg, 0.33 mmol), 2,3-Dihydrobenzo[b]furan-5-carbaldehyde (66 mg, 0.44 mmol, 1.1 eq), the benzoic acid/piperidine catalyst mix (approximately 5 drops) and ethanol (3 mL). The reaction mixture was heated by microwave irradiation (200 W, 120 °C) for 20 min after which a precipitate was observed. The solution was cooled overnight and the solid collected by vacuum filtration, washing with water, cold ethanol and diethyl ether to give the desired product (87 mg, 61%).

Other analyses, reference papers, previously obtained data, comments, etc:

PerkinElmer Spectrum Version 10.4.00
Monday, 27 May 2019 1:49 PM

Analyst	research
Date	Monday, 27 May 2019 1:49 PM

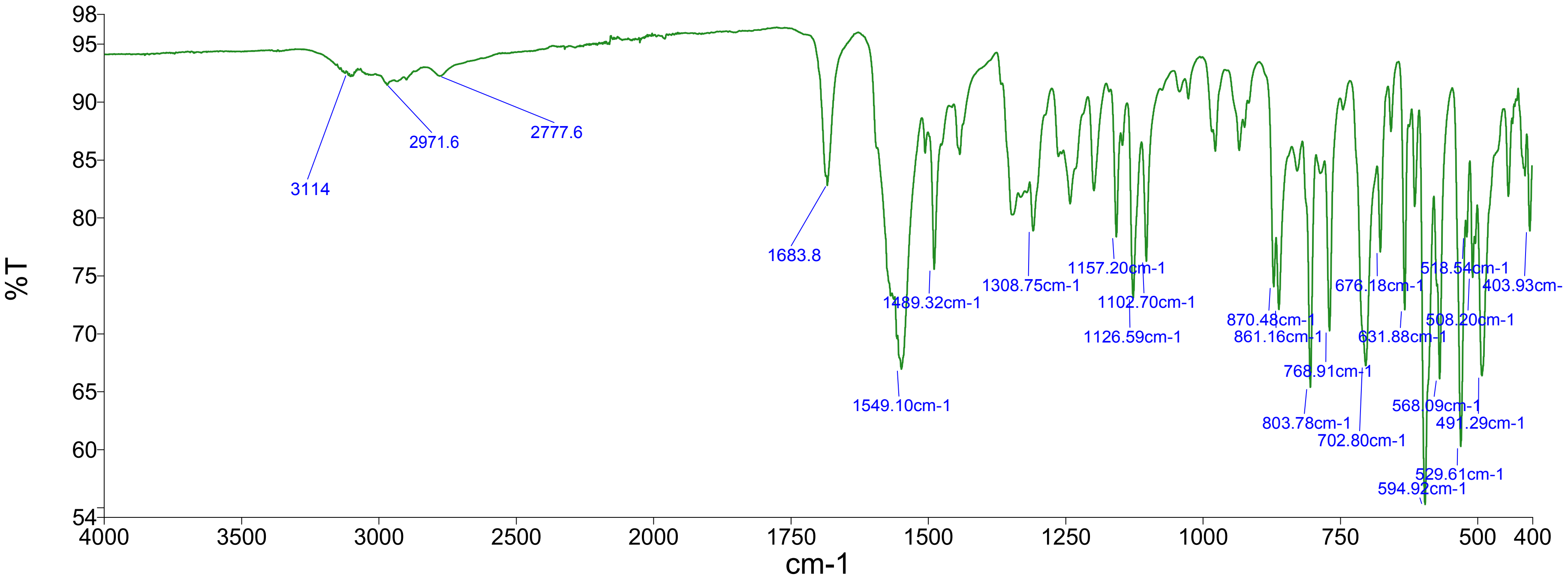

Sample Name	Description	Quality Checks
KP6041	Sample 216 By research Date Monday, May 27 2019	The Quality Checks do not report any warnings for the sample.

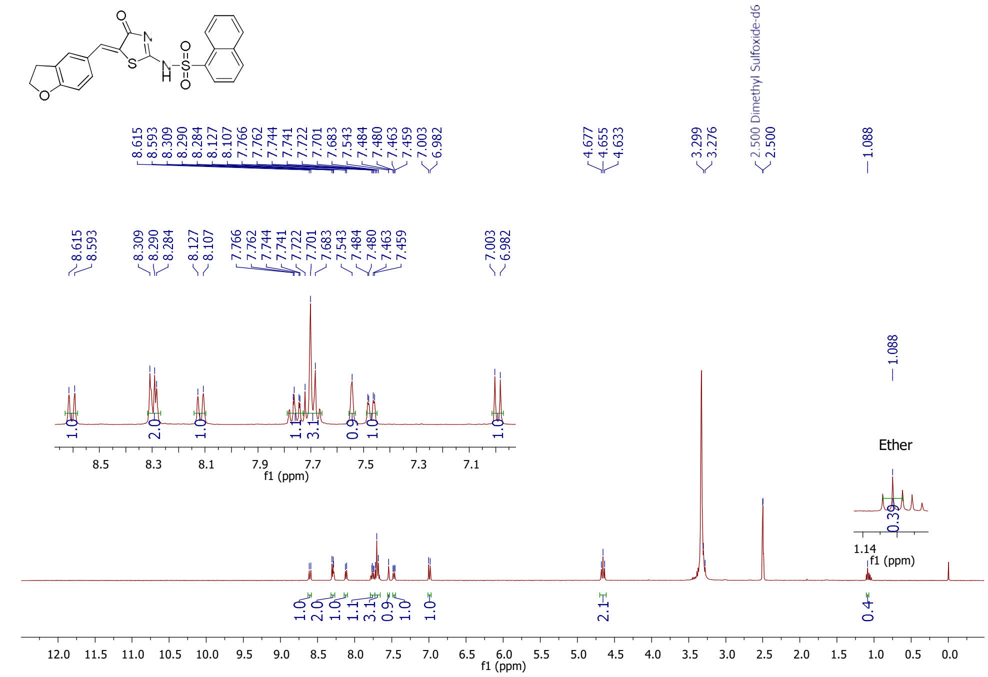

8.615 8.593 8.309 8.290 8.284 8.127 8.107 7.766 7.762 7.744 7.741 7.722 7.701 7.683 7.543 7.484 7.480 7.463 7.459 7.003 6.982
4.677 4.655 4.633
3.299 3.276
2.500 Dimethyl Sulfoxide-d6
2.500
1.088
Ether
f1 (ppm)

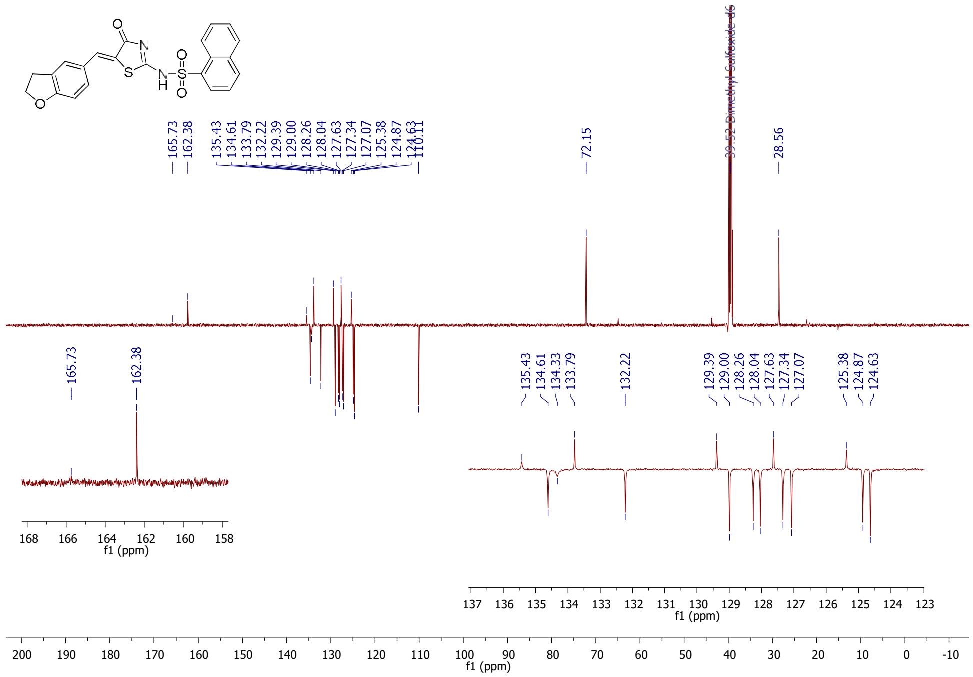

165.73 162.38
135.43 134.61 133.79 132.22 129.39 129.00 128.26 128.04 127.63 127.34 127.07 125.38 124.87 124.63 110.11
72.15
39.52 Dimethyl Sulfoxide-d6
28.56
134.33
f1 (ppm)

LCMS Report

**Data file:**	D:\Chem32\1\Data\KP\PRE 11-6-19\KP6039-47 2019-05-21 10-32-44\006-37-KP6041.D		
**Sample name:**	KP6041		
**Description:**			
**Sample amount:**	0.000	**Sample type:**	Sample
**Instrument:**	LCMS	**Location:**	37
**Injection date:**	5/21/2019 11:07:49 AM	**Injection:**	1 of 1
**Acq. method:**	LCMS ISOCRATIC 50% B.M_REDUCED FLOW.M	**Injection volume:**	2.000
**Analysis method:**	LCMS ISOCRATIC 50% B.M_REDUCED FLOW.M	**Acq. operator:**	SYSTEM
**Last changed:**	5/15/2019 9:20:00 AM		

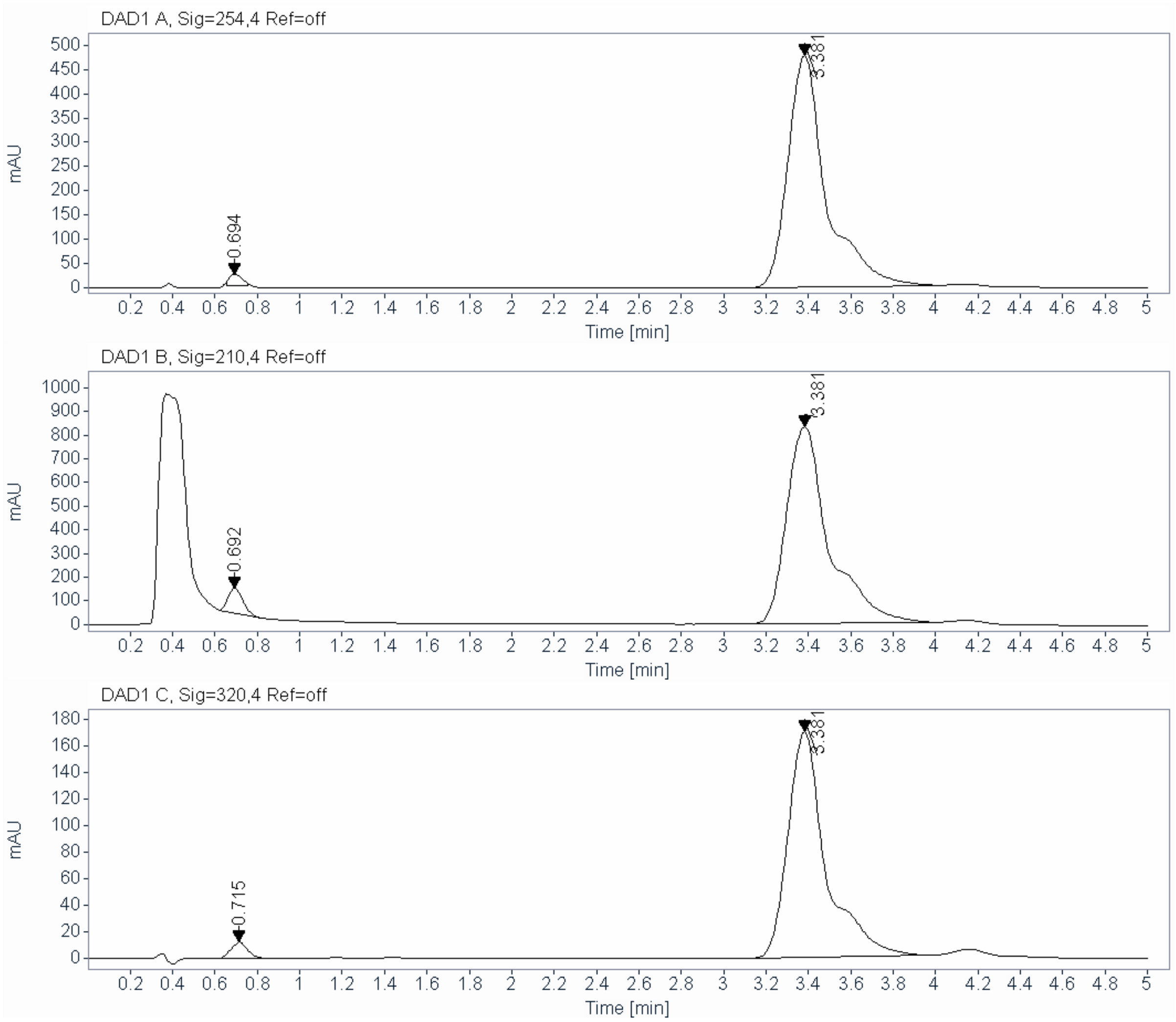

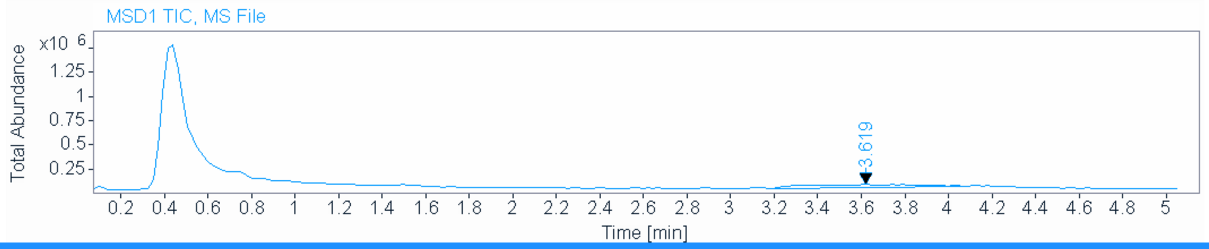

Agilent Technologies

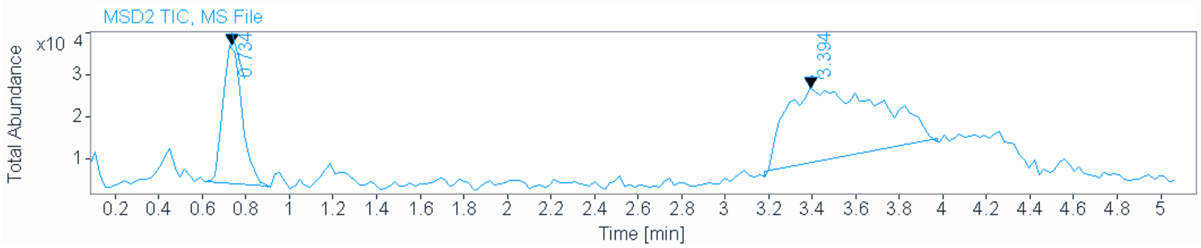

Signal: DAD1 A, Sig=254,4 Ref=off

RT [min]	Type	Width [min]	Area	Height	Area%	Name
0.694	MM	0.0685	96.4832	23.4810	1.5937	
3.381	BB	0.1863	5957.6323	475.1249	98.4063	
		Sum	6054.1155			

Signal: DAD1 B, Sig=210,4 Ref=off

RT [min]	Type	Width [min]	Area	Height	Area%	Name
0.692	BB	0.0798	510.6386	103.0395	4.2613	
3.381	BB	0.2072	11472.5762	830.0742	95.7387	
		Sum	11983.2148			

Signal: DAD1 C, Sig=320,4 Ref=off

RT [min]	Type	Width [min]	Area	Height	Area%	Name
0.715	BB	0.0918	68.6726	12.1698	3.1452	
3.381	BB	0.1857	2114.7249	169.3154	96.8548	
		Sum	2183.3975			

Signal: MSD1 TIC, MS File

RT [min]	Type	Width [min]	Area	Height	Area%	Name
3.619	BB	0.4583	992142.8750	26448.9746	100.0000	
		Sum	992142.8750			

Signal: MSD2 TIC, MS File

RT [min]	Type	Width [min]	Area	Height	Area%	Name
0.734	BB	0.0979	200736.4688	33256.6367	28.3740	
3.394	MM	0.4708	506728.9063	17938.0820	71.6260	
		Sum	707465.3750			

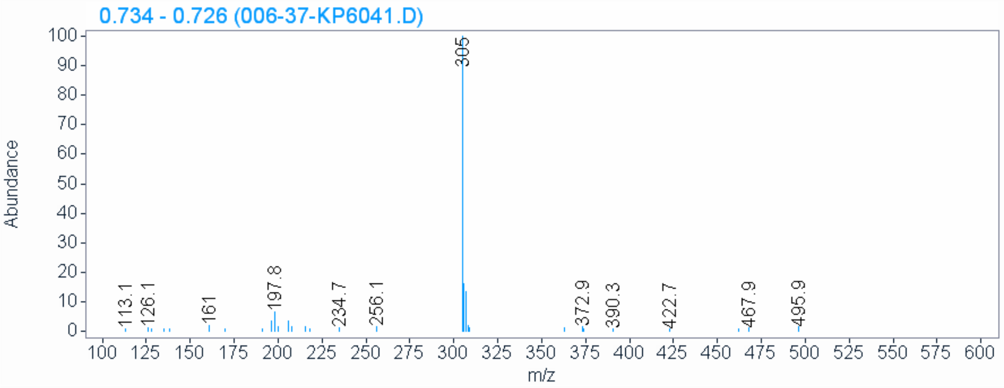
0.734 - 0.726 (006-37-KP6041.D)
Abundance
m/z
113.1
126.1
161
197.8
234.7
256.1
305
372.9
390.3
422.7
467.9
495.9

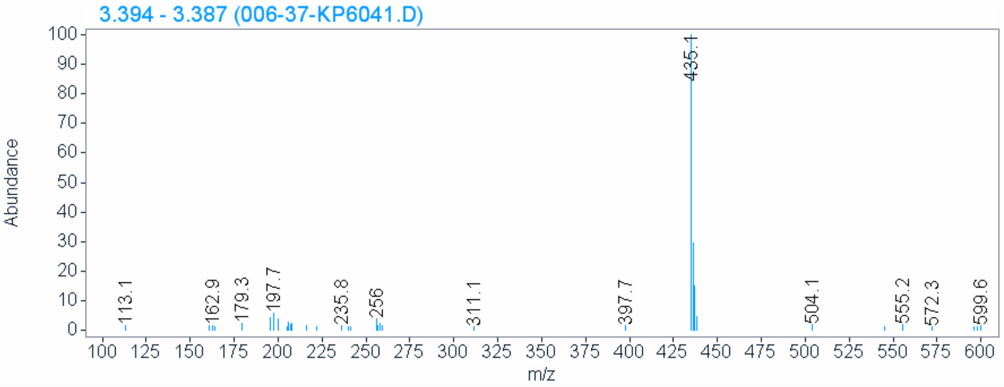
3.394 - 3.387 (006-37-KP6041.D)
Abundance
m/z
113.1
162.9
179.3
197.7
235.8
256
311.1
397.7
435.1
504.1
555.2
572.3
599.6

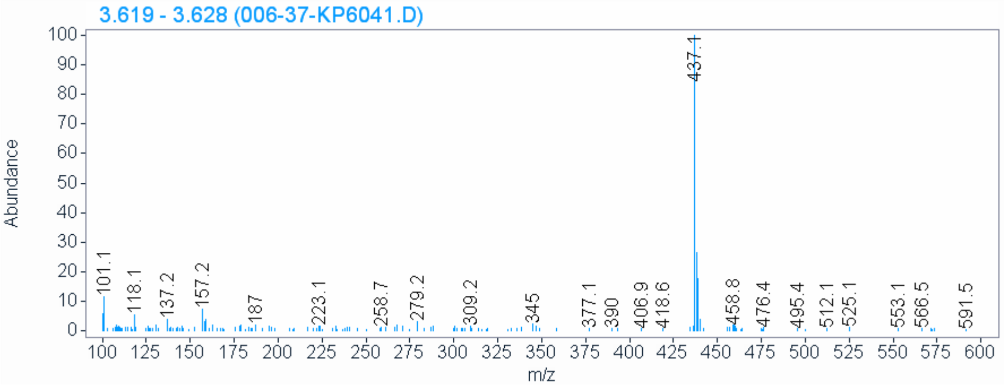
3.619 - 3.628 (006-37-KP6041.D)
Abundance
m/z
101.1
118.1
137.2
157.2
187
223.1
258.7
279.2
309.2
345
377.1
390
406.9
418.6
437.1
458.8
476.4
495.4
512.1
525.1
553.1
566.5
591.5

Compound Name: (Z)-*N*-(5-(benzo[c][1,2,5]thiadiazol-5-ylmethylene)-4-oxo-4,5-dihydrothiazol-2-yl)naphthalene-1-sulfonamide

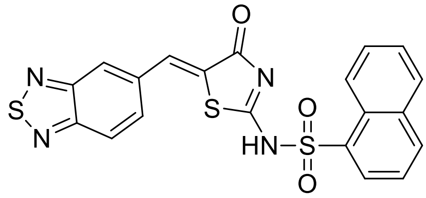

Chemical Formula: $C_{20}H_{12}N_4O_3S_3$
Exact Mass: 452.01
Molecular Weight: 452.53

Compound Code: 58 (KP6045)

Obtained Weight & Yield: 68 mg (31%).

Purity (by LCMS and ^{1}H NMR): > 97% by ^{1}H-NMR

Appearance: Brown powder

Solubility: DMSO, slightly soluble in ethanol and methanol

Melting Point: > 221 °C (dec.)

TLC Rf (and conditions): 0.34 (10% MeOH in DCM)

IR Analysis (including assignment): IR (neat): ν_{max} = 3056, 2939, 2771 (C-H aromatic), 1716 (C=O), 1557 (C-C aromatic), 1325 (sulfonamide), 1124 (C-N) cm^{-1}

^{1}H NMR Analysis: ^{1}H NMR (400 MHz, DMSO) δ 8.63 – 8.61 (m, 1H), 8.42 (s, 1H), 8.34 – 8.28 (m, 3H), 8.12 (d, *J* = 8.0 Hz, 1H), 7.99 (s, 1H), 7.97 (dd, *J* = 9.2, 1.7 Hz, 1H), 7.78 – 7.76 (m, 1H), 7.73 – 7.69 (m, 2H) ppm.
NH exchanging – not observed
Starting material impurity at 4.04ppm (1.99%), DCM at 5.75 ppm (0.65%)

^{13}C NMR Analysis: ^{13}C NMR (101 MHz, DMSO) δ 166.7, 165.3, 154.3, 154.2, 135.2, 134.8, 134.6, 133.8, 132.2, 130.1, 129.0, 128.3, 128.2, 127.6, 127.1, 125.0, 124.8, 124.7, 123.5, 122.3 ppm.

MS Analysis (low res): LRMS (ESI-) *m/z*: 451 (M-H, $C_{20}H_{11}N_4O_3S_3$, 100%)

HPLC method details: Column: Zorbax SB-C18 Rapid Resolution HT 2.1x50mm 1.8-Micron; Method: LCMS ISOCRATIC 60%B 0.4MLMIN-1.M filename: KP6045; Peak retention time: 1.59 mins; Area (%): 79.

Procedure: To a microwave vial was added the *N*-(4-oxo-4,5-dihydrothiazol-2-yl)naphthalene-1-sulfonamide (147 mg, 0.49 mmol), benzothiadiazle-5-carbaldehyde (90 mg, 0.54 mmol, 1.1 eq), the benzoic acid/piperidine catalyst (approximately 10 drops) and ethanol (3 mL). The reaction was heated by microwave irradiation (200 W, 120 °C) for 1 h and cooled overnight. The solid was collected and remaining starting material removed by column chromatography using a gradient solvent up to 10% MeOH in DCM (68 mg, 31%).

Other analyses, reference papers, previously obtained data, comments, etc:

PerkinElmer Spectrum Version 10.4.00
Monday, 27 May 2019 1:54 PM

Analyst research
Date Monday, 27 May 2019 1:54 PM

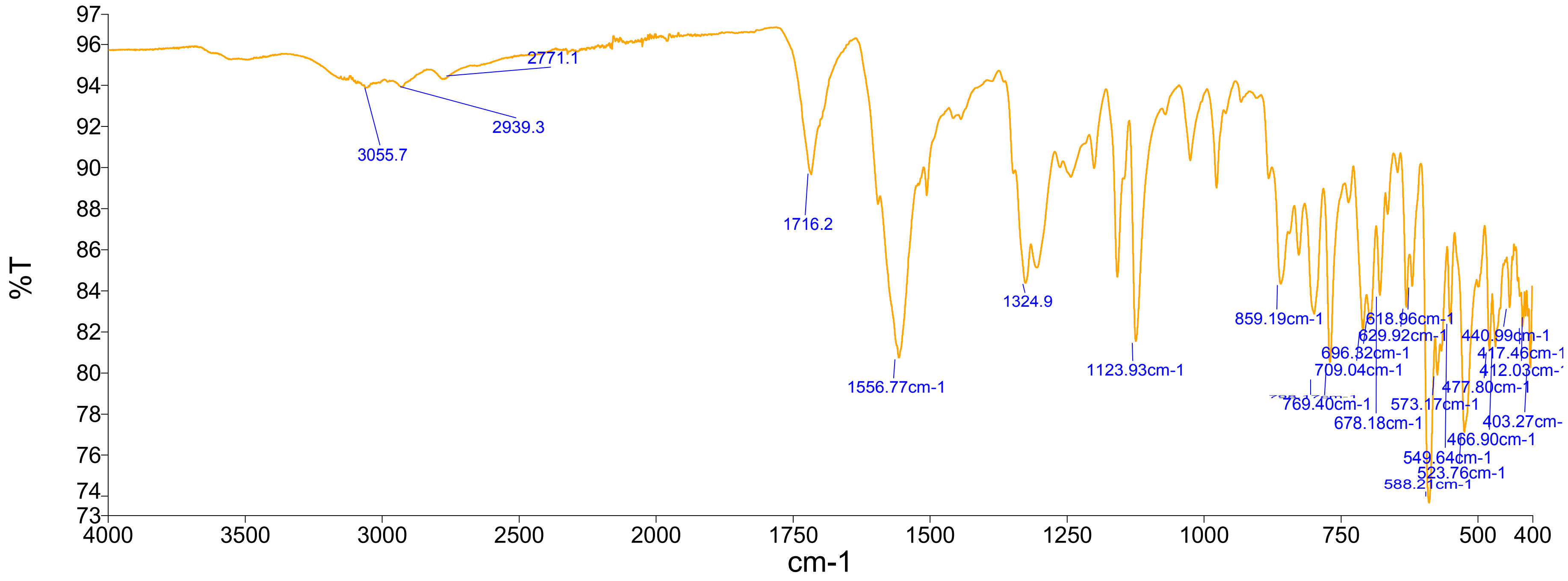

Sample Name	Description	Quality Checks
KP6045	Sample 217 By research Date Monday, May 27 2019	The Quality Checks give rise to a Weak Bands warning for the sample.

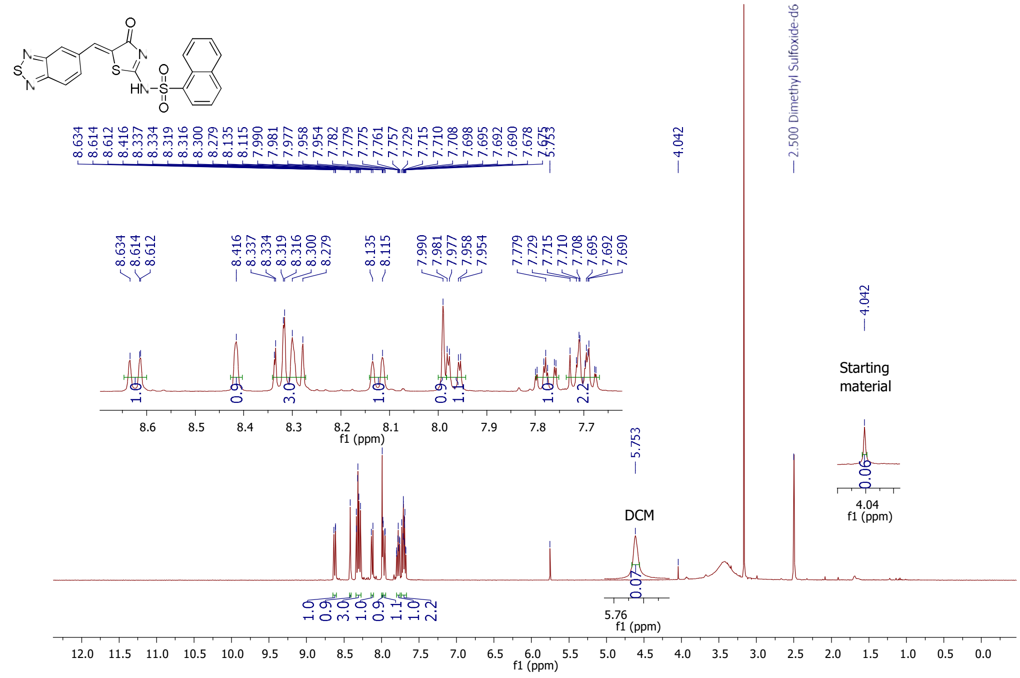

Starting material
DCM
2.500 Dimethyl Sulfoxide-d6
f1 (ppm)

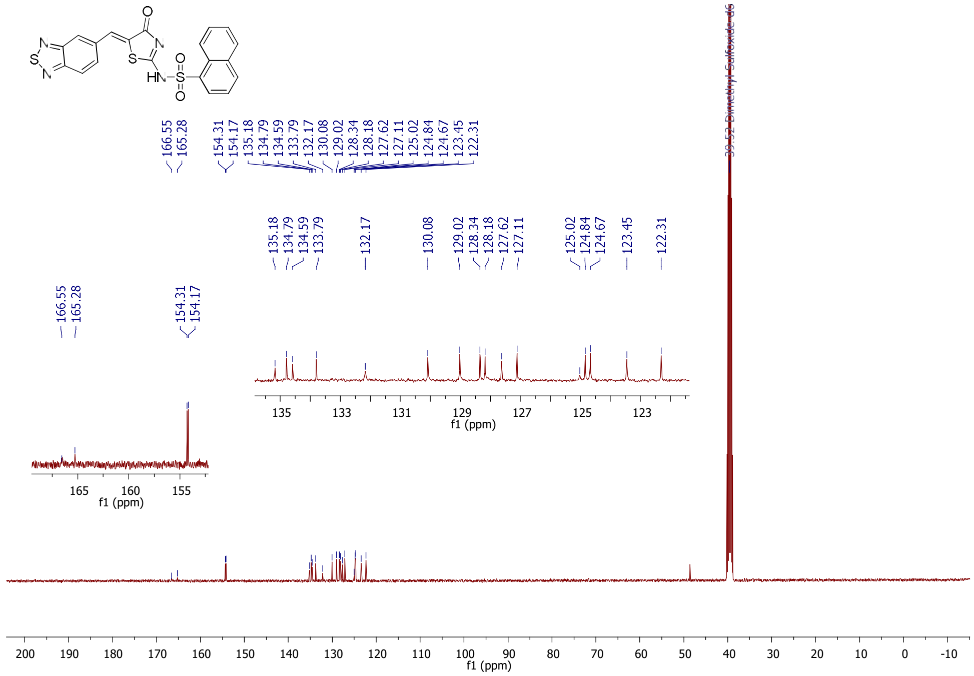

39.52 Dimethyl Sulfoxide-d6
f1 (ppm)

LCMS Report

Agilent Technologies

**Data file:**	D:\Chem32\1\Data\KP\PRE 11-6-19\KP60514519 2019-05-24 14-00-35\003-36-KP6045.D		
**Sample name:**	KP6045		
**Description:**			
**Sample amount:**	0.000	**Sample type:**	Sample
**Instrument:**	LCMS	**Location:**	36
**Injection date:**	5/24/2019 2:17:34 PM	**Injection:**	1 of 1
**Acq. method:**	LCMS ISOCRATIC 60% B 0.4MLMIN-1.M	**Injection volume:**	2.000
**Analysis method:**	LCMS ISOCRATIC 60%B 0.4MLMIN-1.M	**Acq. operator:**	SYSTEM
**Last changed:**	5/8/2019 8:55:04 AM		

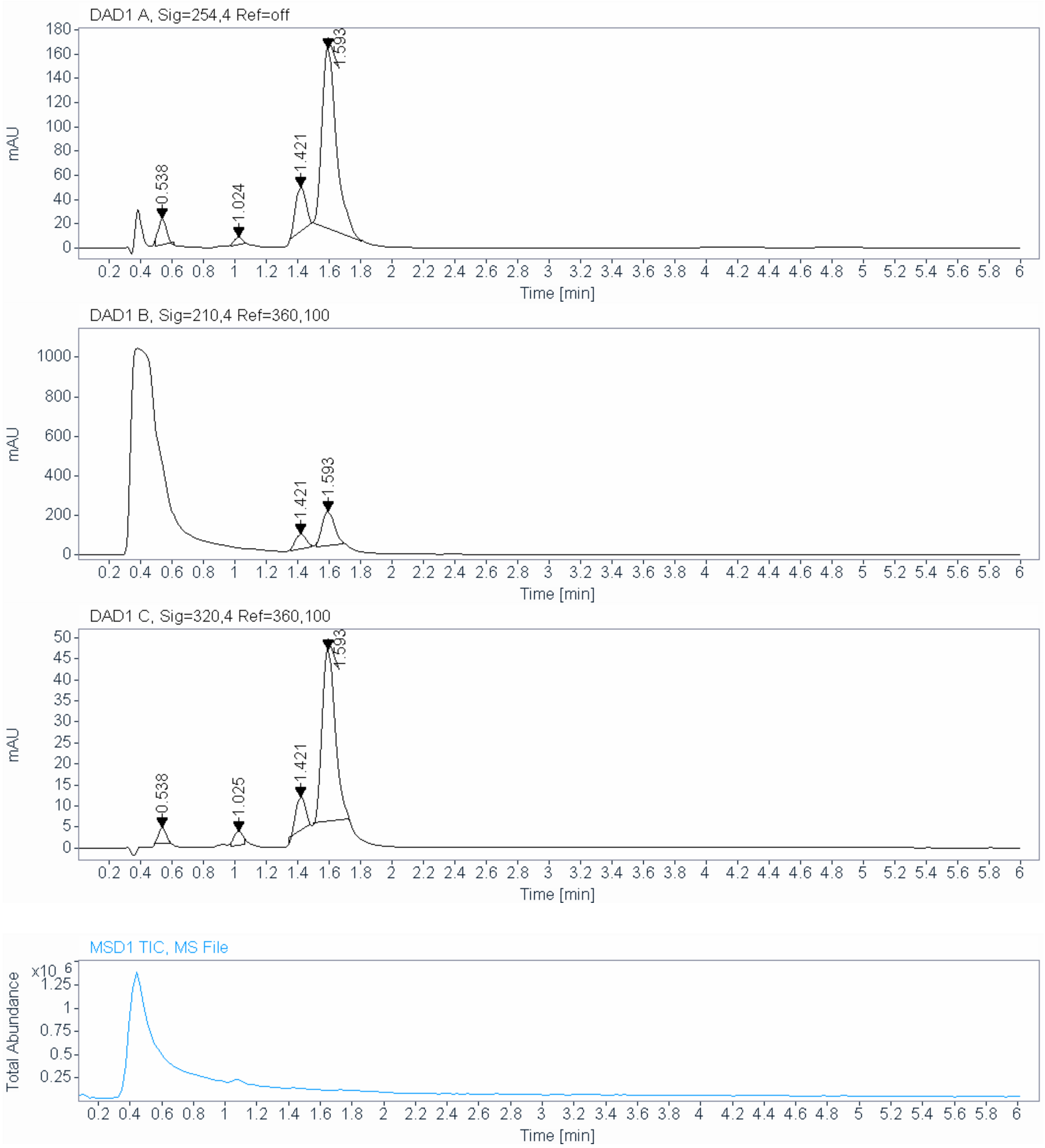

LCMS Report

Agilent Technologies

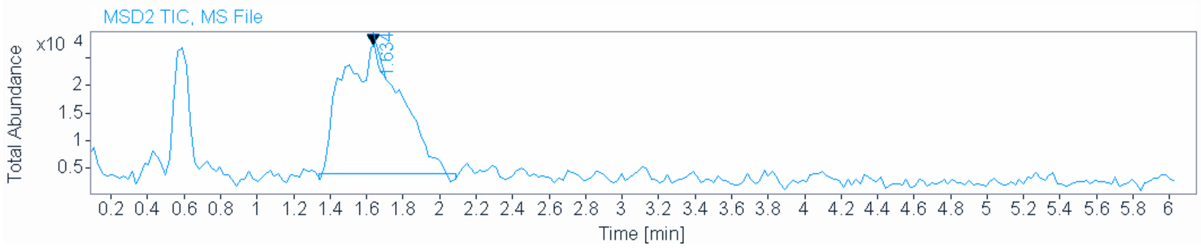

Signal: DAD1 A, Sig=254,4 Ref=off

RT [min]	Type	Width [min]	Area	Height	Area%	Name
0.538	MM	0.0596	75.8147	21.2043	6.1705	
1.024	MM	0.0577	21.3498	6.1716	1.7376	
1.421	MM	0.0754	166.8683	36.8949	13.5812	
1.593	MM	0.1075	964.6348	149.5297	78.5106	
		Sum	1228.6676			

Signal: DAD1 B, Sig=210,4 Ref=360,100

RT [min]	Type	Width [min]	Area	Height	Area%	Name
1.421	MM	0.0808	361.2500	74.5562	27.5684	
1.593	MM	0.0919	949.1288	172.1049	72.4316	
		Sum	1310.3789			

Signal: DAD1 C, Sig=320,4 Ref=360,100

RT [min]	Type	Width [min]	Area	Height	Area%	Name
0.538	MM	0.0576	13.3894	3.8739	4.5016	
1.025	MM	0.0626	12.6991	3.3786	4.2695	
1.421	MM	0.0689	33.2646	8.0480	11.1838	
1.593	MM	0.0964	238.0836	41.1435	80.0451	
		Sum	297.4368			

Signal: MSD2 TIC, MS File

RT [min]	Type	Width [min]	Area	Height	Area%	Name
1.634	MM	0.3660	521485.5625	23750.0371	100.0000	
		Sum	521485.5625			

Agilent Technologies

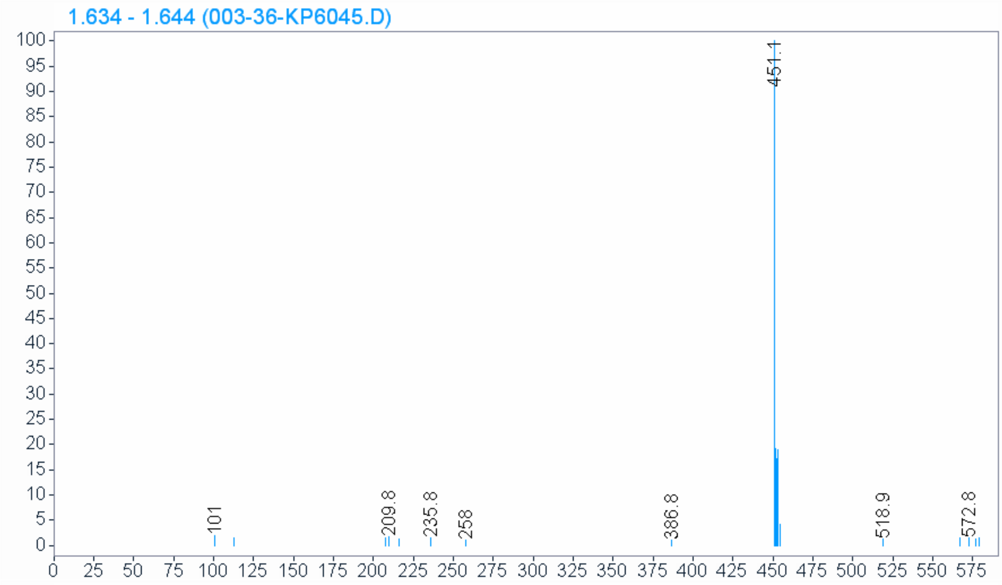

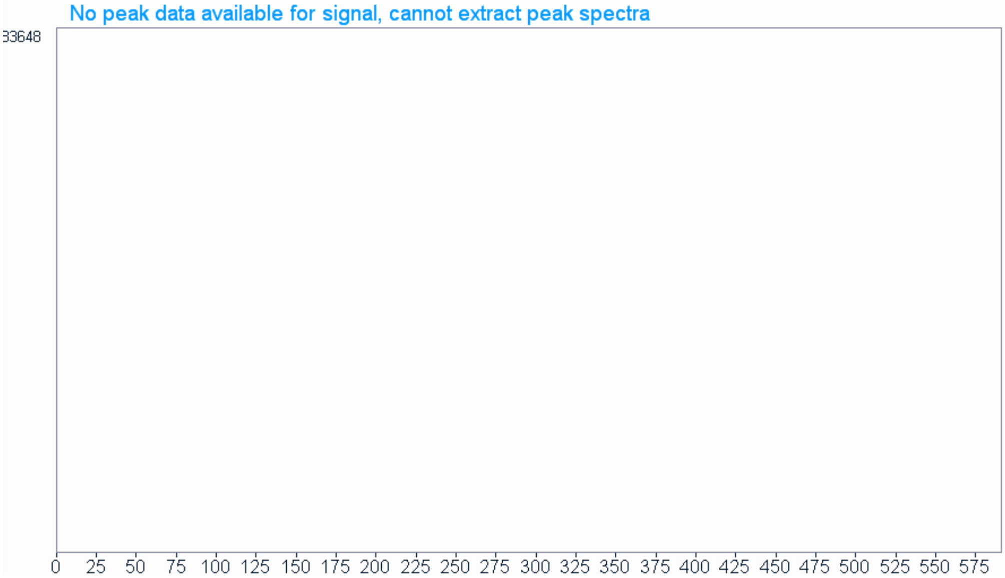

Compound Name: (Z)-*N*-(5-((1H-indol-5-yl)methylene)-4-oxo-4,5-dihydrothiazol-2-yl)naphthalene-1-sulfonamide

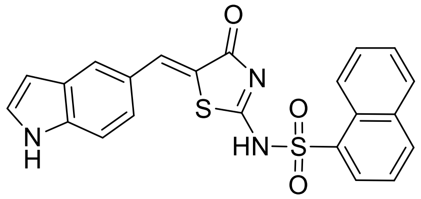

Chemical Formula: $C_{22}H_{15}N_3O_3S_2$
Exact Mass: 433.06
Molecular Weight: 433.50

Compound Code: 59 (KP7059)

Obtained Weight & Yield: 104 mg, 48%

Purity (by LCMS and ^{1}H NMR): > 95% by ^{1}H-NMR and LCMS

Appearance: orange solid

Solubility: DMSO, slightly soluble in acetone and methanol

Melting Point: > 186 °C (dec.)

TLC Rf (and conditions): N/A

IR Analysis (including assignment): IR (neat): 3340 (N-H), 2985 (C-H aromatic), 2784 (C-H), 1687 (C=O), 1559 (aromatic C-C), 1289 (sulfonamide), 1116 (C-N) cm^{-1}

^{1}H NMR Analysis: ^{1}H NMR (400 MHz, DMSO) δ 11.56 (br, s, 1H, NH), 8.62 (d, *J* = 8.5 Hz, 1H), 8.33 – 8.29 (m, 2H), 8.12 (d, *J* = 8.0 Hz, 1H), 7.92 (d, *J* = 15.3 Hz, 1H), 7.88 (s, 1H), 7.77 (t, *J* = 7.27 Hz, 1H), 7.73 – 7.76 (m, 2H), 7.62 (d, *J* = 8.5 Hz, 1H), 7.51 – 7.50 (m, 1H), 7.43 – 7.41 (m, 1H), 6.64 (s, 1H) ppm.
One NH exchanging – not visible
Ethanol at 1.06 ppm (0.95%) and starting material at 4.04 ppm (2.41%)

^{13}C NMR Analysis: ^{13}C NMR (101 MHz, DMSO) δ 166.7, 166.0, 137.2, 136.6, 135.4, 134.6, 133.8, 129.0, 128.29, 128.25 (2C), 128.1, 127.7, 127.1, 124.9, 124.65, 124.5, 123.8, 123.3, 116.8, 112.7, 102.6 ppm.

MS Analysis (low res): LRMS (ESI-) *m/z* (%): 432 (*M*-H, $C_{22}H_{14}N_3O_3S_2$, 100%).

MS Analysis (high res): Exact mass calculated for $C_{22}H_{14}N_3O_3S_2$ [*M*-H]$^-$, 432.0500. Found 432.0481.

HPLC method details: Column: Zorbax SB-C18 Rapid Resolution HT 2.1x50mm 1.8-Micron; Method: LCMS ISOCRATIC 60%B 0.4MLMIN-1.M filename: KP7059; Peak retention time: 0.949 mins; Area (%): 100

Procedure: To a 10 mL microwave vial was added *N*-(4-oxo-4,5-dihydrothiazol-2-yl)naphthalene-1-sulfonamide (146 mg, 0.49 mmol), indole-5-carboxaldehyde (84 mg, 0.54 mmol, 1.1 eq), ethanol (3 mL) and a catalytic amount of the benzoic acid/piperidine catalyst (approximately 5 drops). The suspension was heated using microwave irradiation (200 W, 120 °C) for 50 min then allowed to precipitate at in the freezer. The resulting precipitate was collected by vacuum filtration and washed with cold ethanol and cold ether to give the desired product (104 mg, 48%).

Other analyses, reference papers, previously obtained data, comments, etc:

PerkinElmer Spectrum Version 10.4.00
Thursday, 21 November 2019 11:21 AM

Analyst	research
Date	Thursday, 21 November 2019 11:21 AM

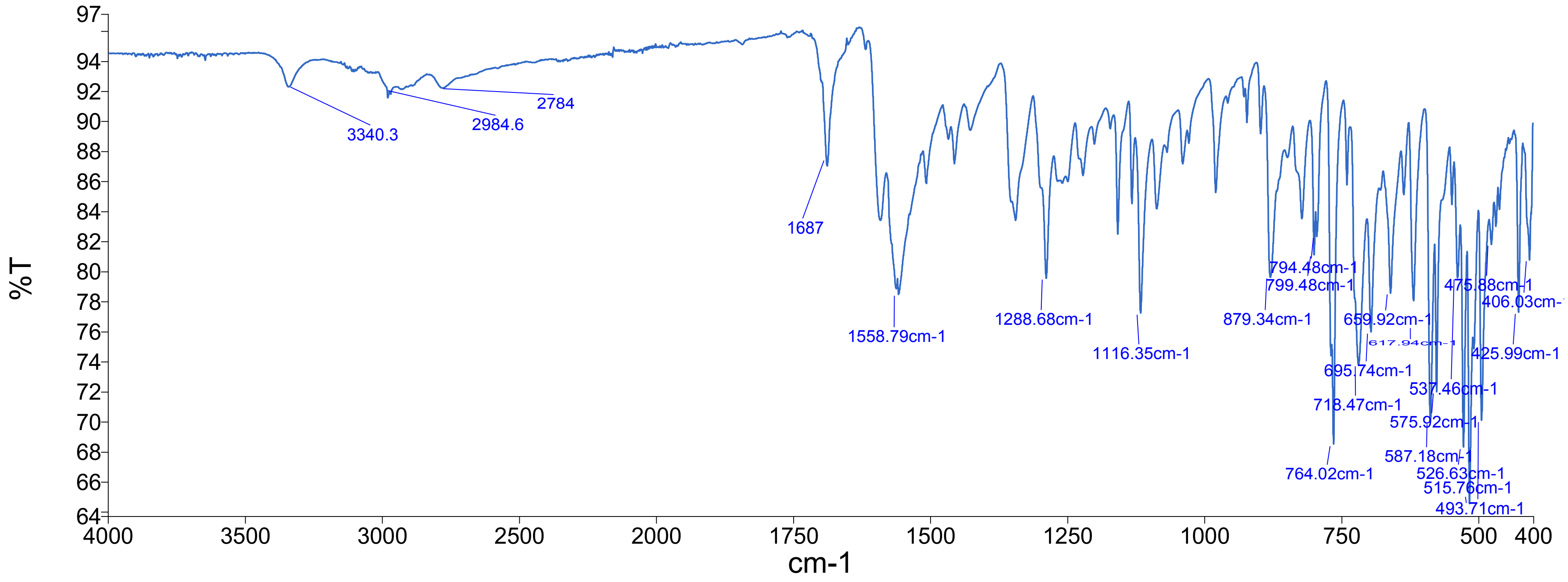

Sample Name	Description	Quality Checks
KP7059	Sample 241 By research Date Thursday, November 21 2019	The Quality Checks give rise to multiple warnings for the sample.

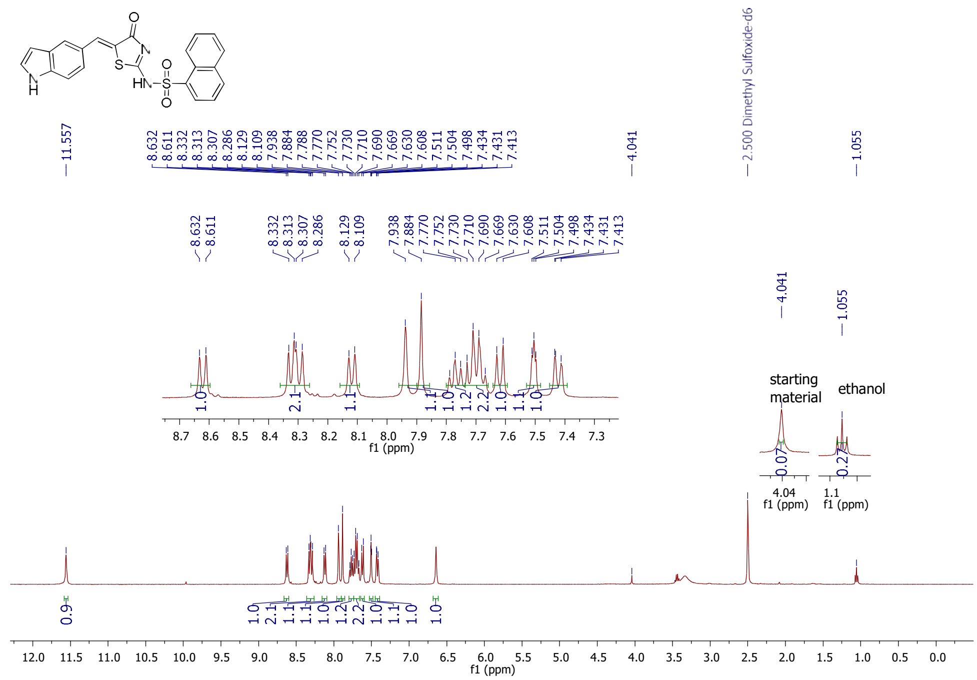

2.500 Dimethyl Sulfoxide-d6
starting material
ethanol
f1 (ppm)

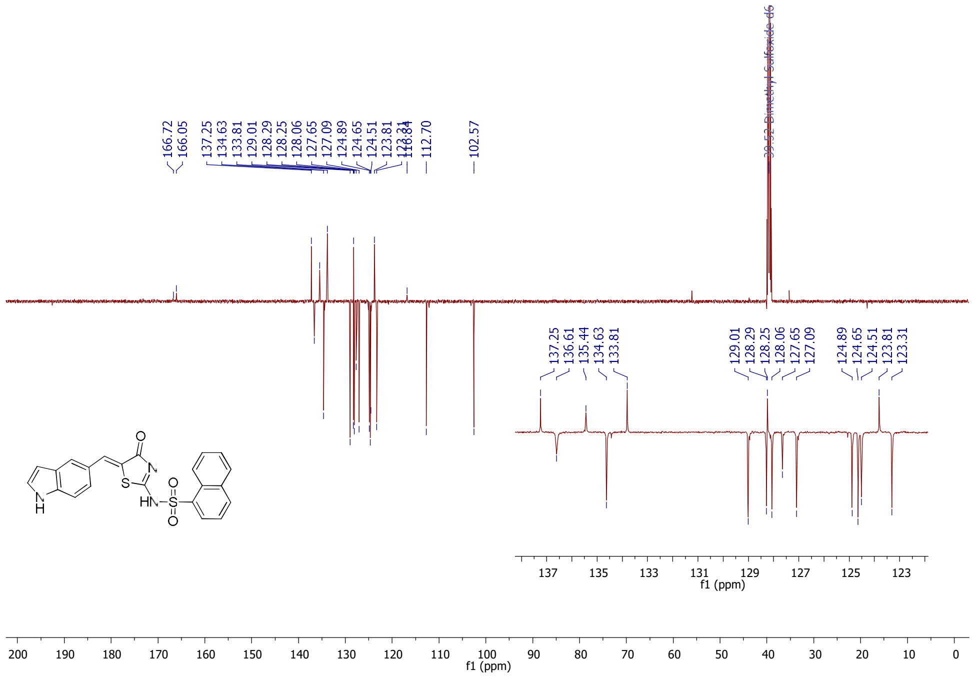

39.52 Dimethyl Sulfoxide-d6
f1 (ppm)

LCMS Report

Agilent Technologies

**Data file:**	D:\Chem32\1\Data\KP\KP_DS_INDOLE1 2019-11-15 10-03-37\003-34-KP6059.D		
**Sample name:**	KP6059		
**Description:**			
**Sample amount:**	0.000	**Sample type:**	Sample
**Instrument:**	LCMS	**Location:**	34
**Injection date:**	11/15/2019 10:20:23 AM	**Injection:**	1 of 1
**Acq. method:**	LCMS ISOCRATIC 60% B 0.4MLMIN-1.M	**Injection volume:**	2.000
**Analysis method:**	LCMS ISOCRATIC 60%B 0.4MLMIN-1.M	**Acq. operator:**	SYSTEM
**Last changed:**	5/8/2019 8:55:04 AM		

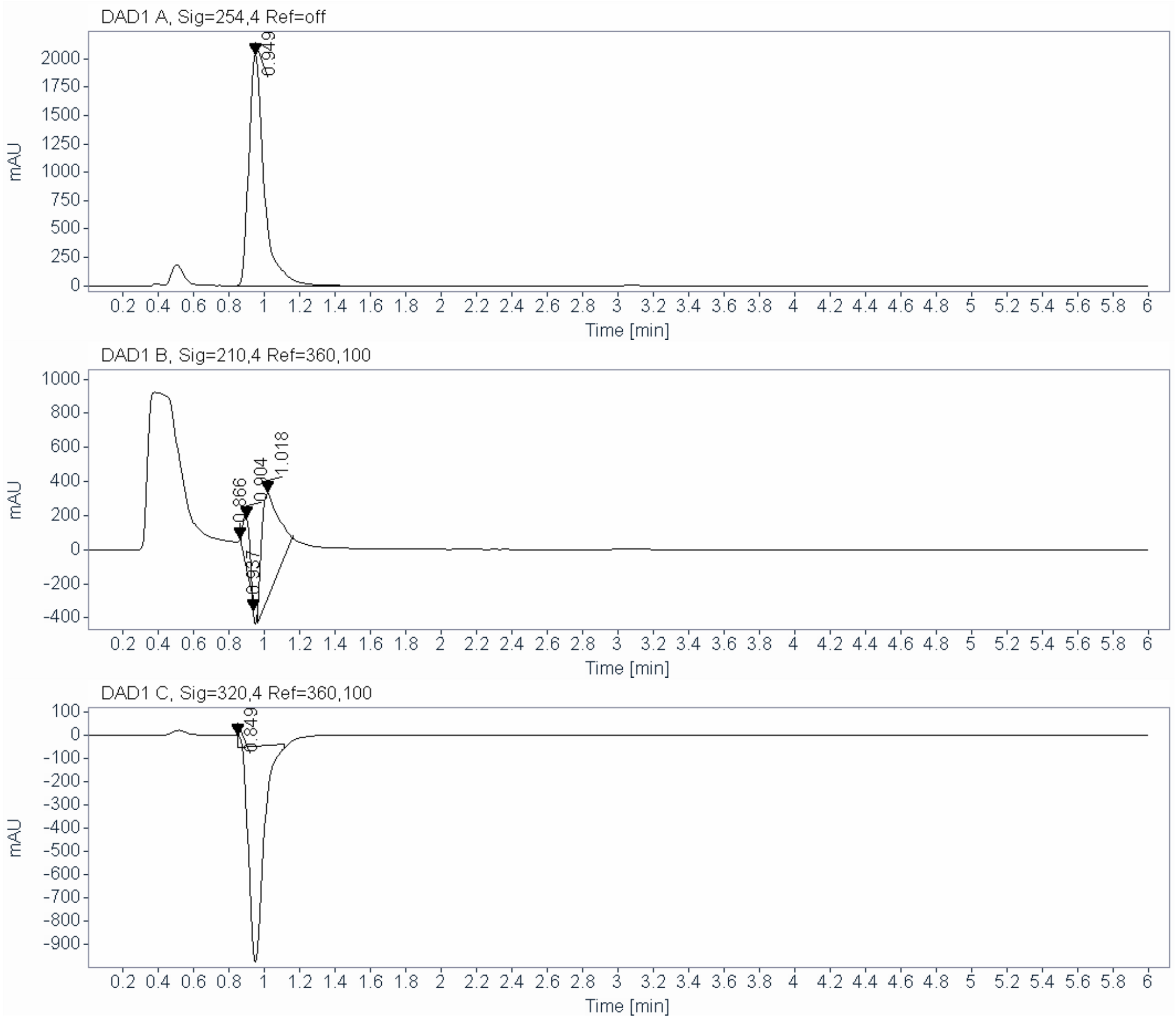

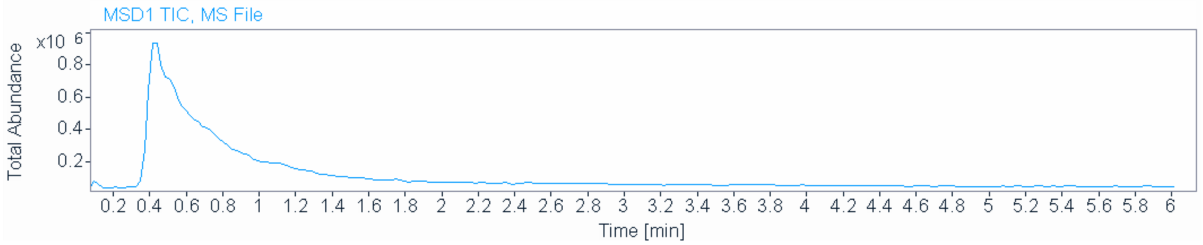

Agilent Technologies

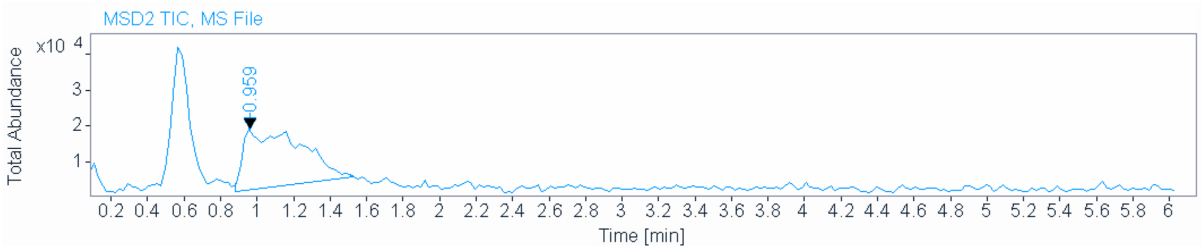

Signal: DAD1 A, Sig=254,4 Ref=off

RT [min]	Type	Width [min]	Area	Height	Area%	Name
0.949	BB	0.0926	12343.9658	2039.0067	100.0000	
		Sum	12343.9658			

Signal: DAD1 B, Sig=210,4 Ref=360,100

RT [min]	Type	Width [min]	Area	Height	Area%	Name
0.866	MP N	0.0000	16.7106	28.6343	0.3570	
0.904	PP	0.0382	727.7986	317.8807	15.5471	
0.937	PM N	0.0118	44.2983	62.4471	0.9463	
1.018	MM	0.1018	3892.4497	637.2025	83.1497	
		Sum	4681.2572			

Signal: DAD1 C, Sig=320,4 Ref=360,100

RT [min]	Type	Width [min]	Area	Height	Area%	Name
0.849	MM	0.0181	59.8466	55.2118	100.0000	
		Sum	59.8466			

Signal: MSD2 TIC, MS File

RT [min]	Type	Width [min]	Area	Height	Area%	Name
0.959	MM	0.3417	356614.9063	17391.6777	100.0000	
		Sum	356614.9063			

Agilent Technologies

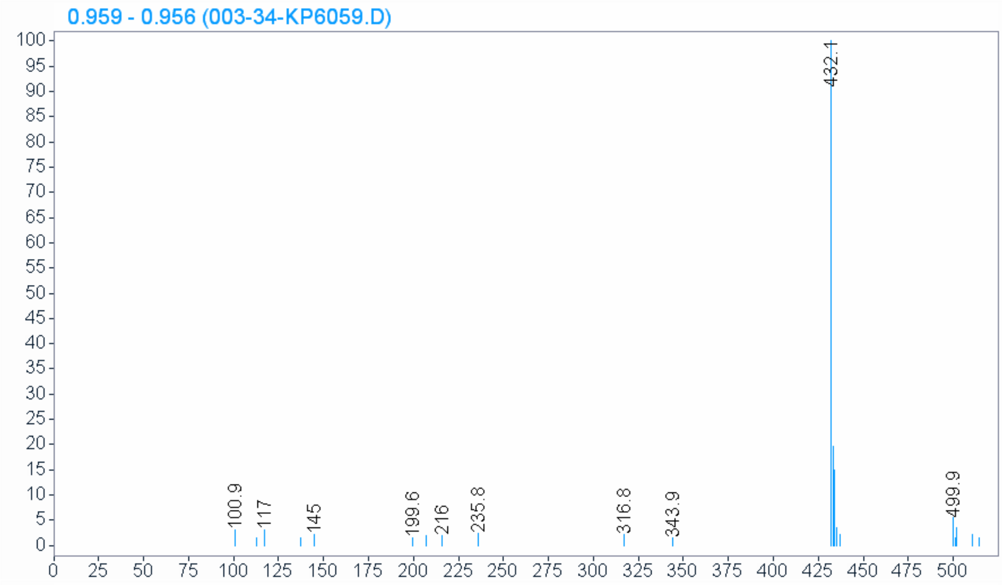

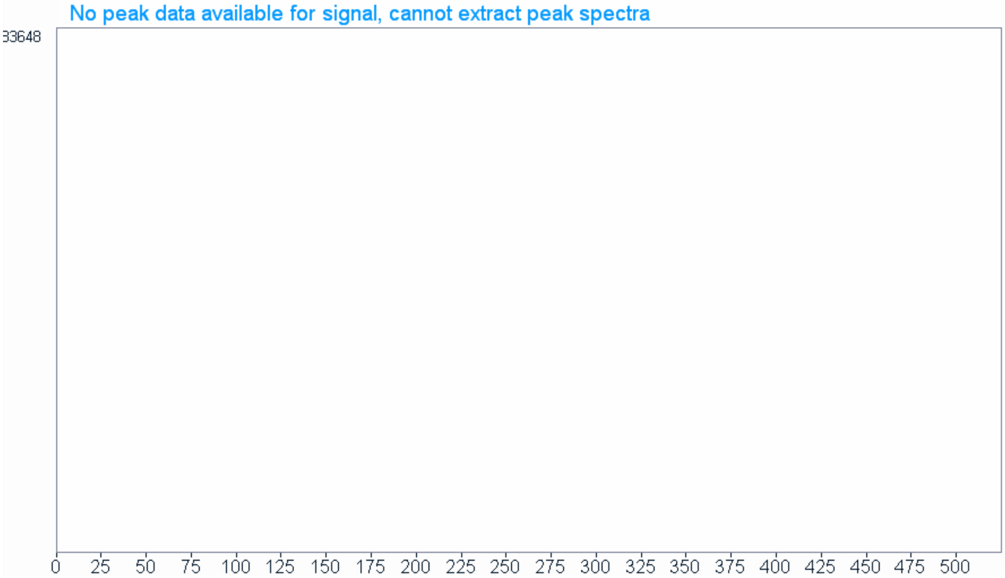

Compound Name: (Z)-*N*-(5-((1H-indol-3-yl)methylene)-4-oxo-4,5-dihydrothiazol-2-yl)naphthalene-1-sulfonamide

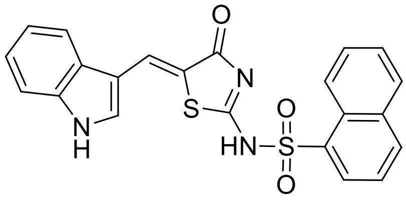

Chemical Formula: $C_{22}H_{15}N_3O_3S_2$
Exact Mass: 433.06
Molecular Weight: 433.50

Compound Code: 60 (KP7058)

Obtained Weight & Yield: 63 mg, 30%

Purity (by LCMS and ^{1}H NMR): >99% by ^{1}H-NMR and LCMS

Appearance: yellow solid

Solubility: DMSO, slightly soluble in acetone and methanol

Melting Point: > 298 °C (dec.)

TLC Rf (and conditions): N/A

IR Analysis (including assignment): IR (neat): 3295 (N-H), 3108, 2926 (C-H aromatic), 2765 (C-H), 1684 (C=O), 1551 (aromatic C-C), 1283 (sulfonamide), 1122 (C-N)

^{1}H NMR Analysis: ^{1}H NMR (400 MHz, DMSO) δ 12.93 (br, s, 1H, NH), 12.23 (br, s, 1H, NH), 8.63 (d, *J* = 8.5 Hz, 1H), 8.31 (dd, *J* = 20.3, 7.7 Hz, 2H), 8.11 (d, *J* = 8.0 Hz, 1H), 8.02 (s, 1H), 7.91 – 7.89 (m, 2H), 7.76 (t, *J* = 7.5 Hz, 1H), 7.72 – 7.66 (m, 2H), 7.55 (d, *J* = 8.0 Hz, 1H), 7.27 (t, *J* = 7.4 Hz, 1H), 7.21 (t, *J* = 7.3 Hz, 1H) ppm.
Ethanol at 1.06 ppm (0.63%)

^{13}C NMR Analysis: ^{13}C NMR (151 MHz, DMSO) δ 166.5, 165.7, 136.4, 135.7, 134.5, 133.8, 129.7, 129.0, 128.2, 128.1, 127.7, 127.0, 126.8, 126.4, 125.0, 124.6, 123.2, 121.3, 118.5, 112.6, 110.4 ppm.
1 carbons missing

MS Analysis (low res): LRMS (ESI-) *m/z* (%): 432 (*M*-H, $C_{22}H_{14}N_3O_3S_2$, 100%)

MS Analysis (high res): Exact mass calculated for $C_{22}H_{14}N_3O_3S_2$ [*M*-H]$^-$, 432.0500. Found 432.0480.

HPLC method details: Column: Zorbax SB-C18 Rapid Resolution HT 2.1x50mm 1.8-Micron; Method: LCMS ISOCRATIC 60%B 0.4MLMIN-1.M filename: KP7058; Peak retention time: 0.972 mins; Area (%): 100

Procedure: To a 10 mL microwave vial was added *N*-(4-oxo-4,5-dihydrothiazol-2-yl)naphthalene-1-sulfonamide (150 mg, 0.49 mmol), indole-3-carboxaldehyde (78 mg, 0.54 mmol, 1.1 eq), ethanol (3 mL) and a catalytic amount of the benzoic acid/piperidine catalyst (approximately 5 drops). The suspension was heated using microwave irradiation (200 W, 120 °C) for 40 min then allowed to precipitate at in the freezer. The resulting precipitate was collected by vacuum filtration and washed with cold ethanol and cold ether to give the desired product (63 mg, 30%).

Other analyses, reference papers, previously obtained data, comments, etc:

PerkinElmer Spectrum Version 10.4.00
Thursday, 21 November 2019 11:21 AM

Analyst	research
Date	Thursday, 21 November 2019 11:21 AM

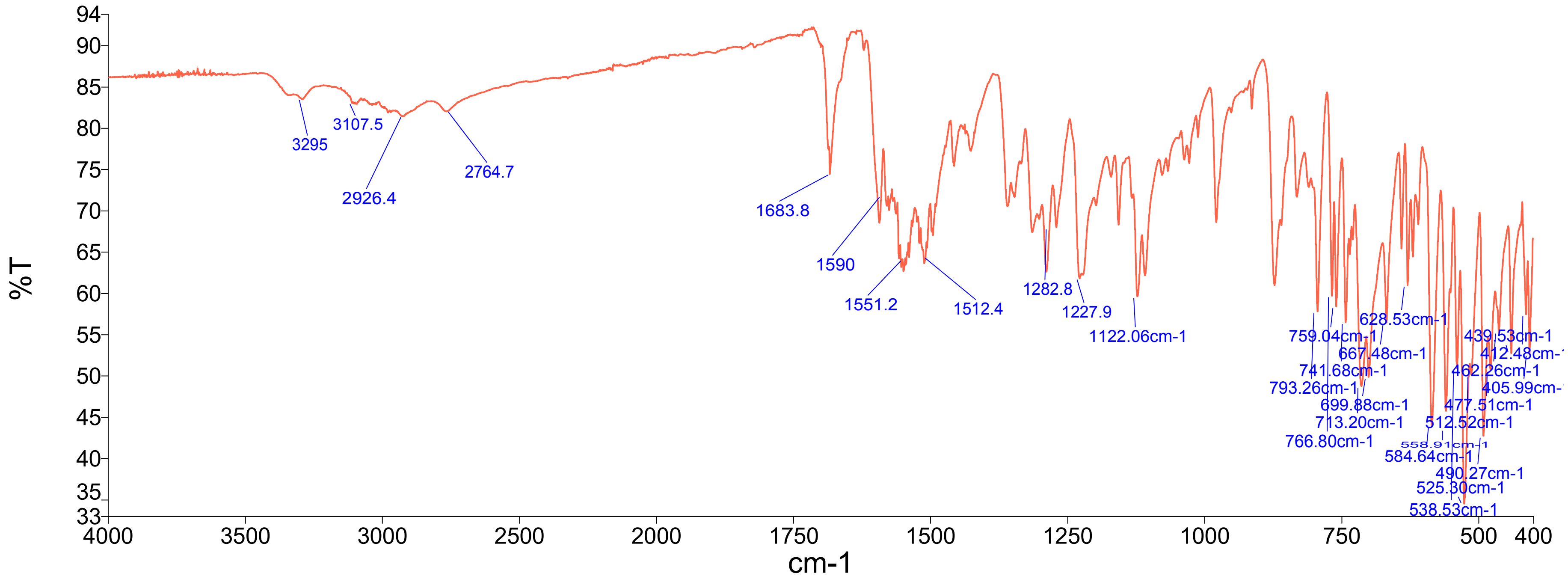

Sample Name	Description	Quality Checks
KP7058	Sample 240 By research Date Thursday, November 21 2019	The Quality Checks give rise to a Negative Bands warning for the sample.

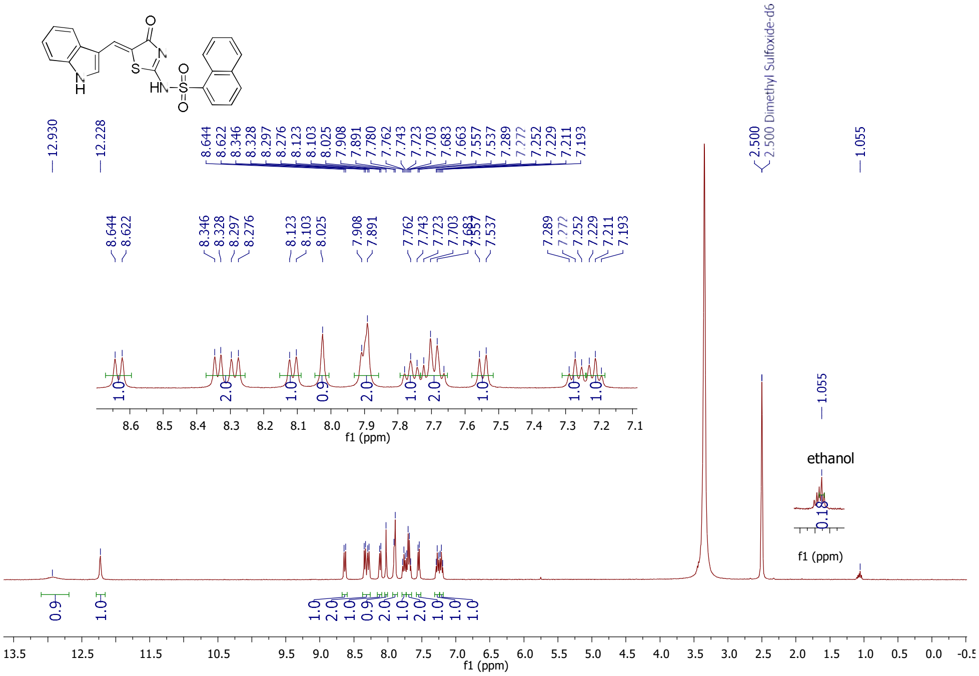
2.500 Dimethyl Sulfoxide-d6
ethanol
f1 (ppm)

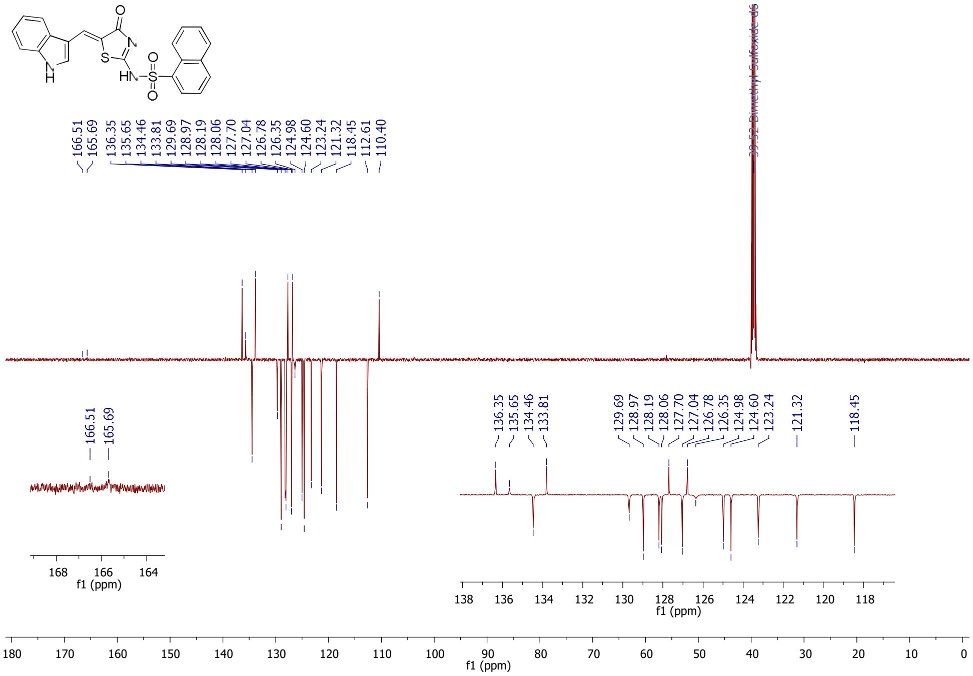
39.52 Dimethyl Sulfoxide-d6
f1 (ppm)

LCMS Report

Data file: D:\Chem32\1\Data\KP\KP_DS_INDOLE1 2019-11-15 10-03-37\002-33-KP6058.D
Sample name: KP6058
Description:
Sample amount: 0.000
Sample type: Sample

Instrument: LCMS
Location: 33
Injection date: 11/15/2019 10:12:50 AM
Injection: 1 of 1
Acq. method: LCMS ISOCRATIC 60% B 0.4MLMIN-1.M
Injection volume: 2.000
Analysis method: LCMS ISOCRATIC 60%B 0.4MLMIN-1.M
Acq. operator: SYSTEM
Last changed: 5/8/2019 8:55:04 AM

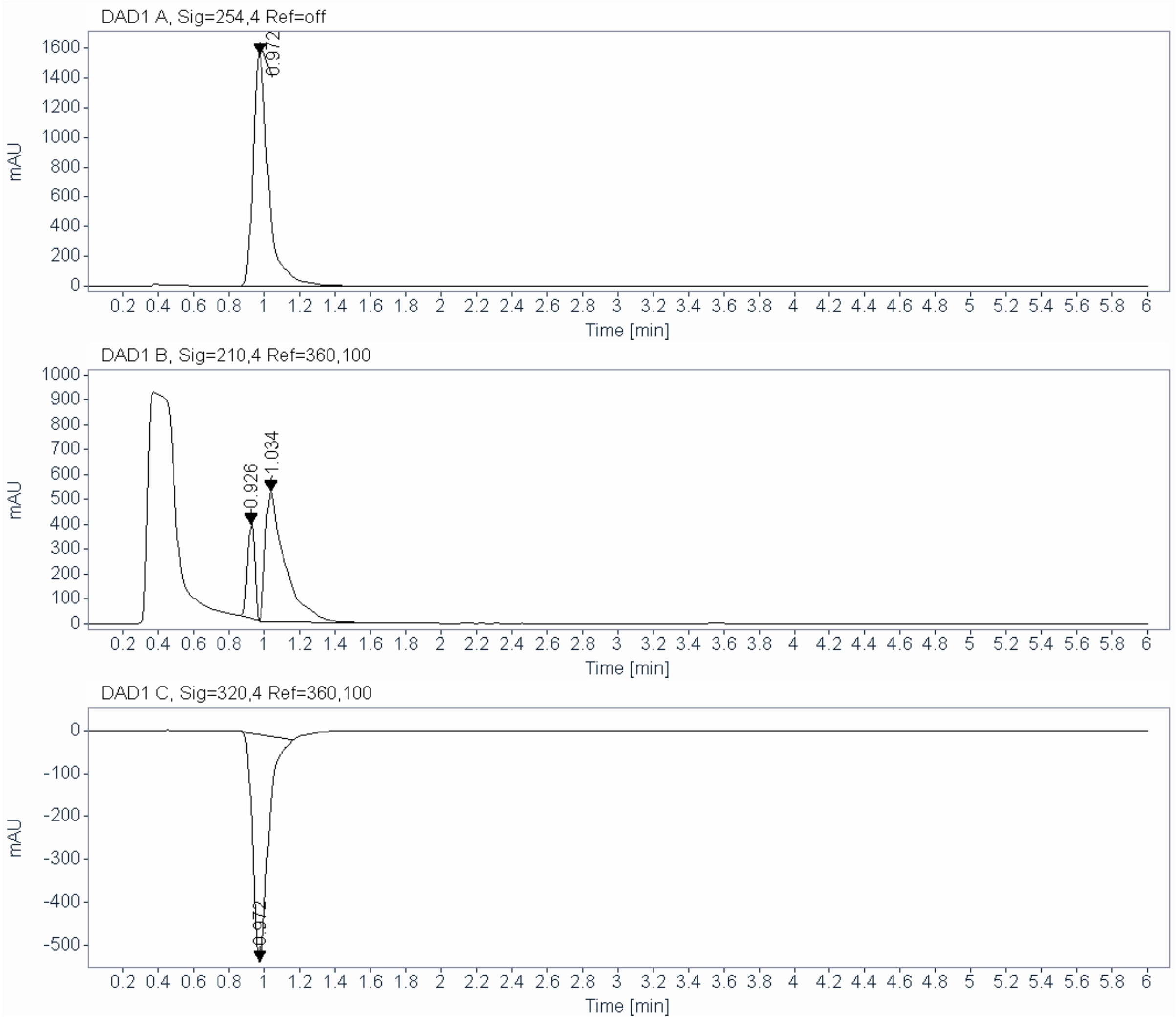

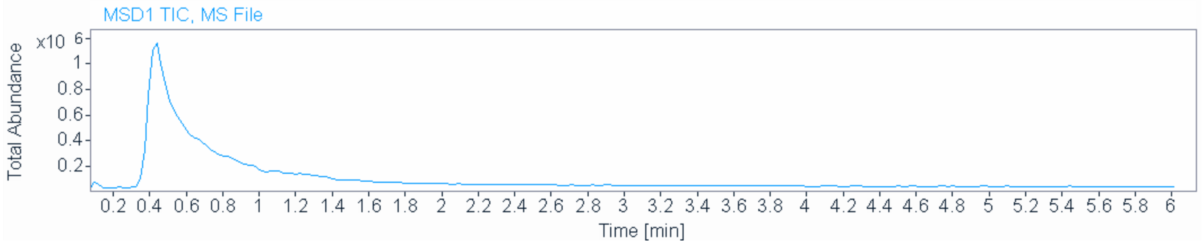

Agilent Technologies

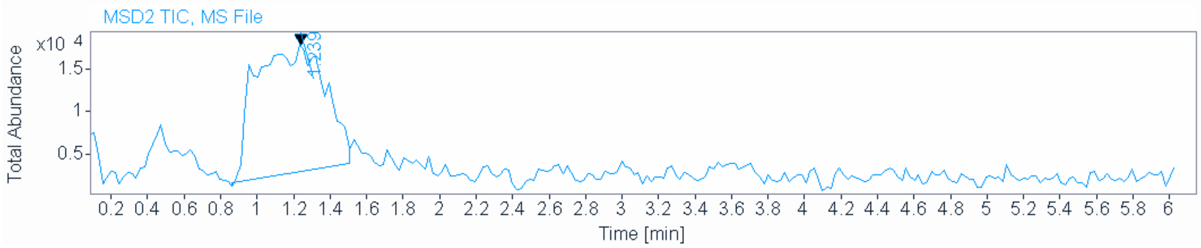

Signal: DAD1 A, Sig=254,4 Ref=off

RT [min]	Type	Width [min]	Area	Height	Area%	Name
0.972	BB	0.0949	9503.3994	1564.4972	100.0000	
		Sum	9503.3994			

Signal: DAD1 B, Sig=210,4 Ref=360,100

RT [min]	Type	Width [min]	Area	Height	Area%	Name
0.926	BB	0.0440	1050.4844	382.6826	20.8142	
1.034	BB	0.1053	3996.4644	522.3287	79.1858	
		Sum	5046.9487			

Signal: DAD1 C, Sig=320,4 Ref=360,100

RT [min]	Type	Width [min]	Area	Height	Area%	Name
0.972	PM N	0.0952	3048.2639	533.5623	100.0000	
		Sum	3048.2639			

Signal: MSD2 TIC, MS File

RT [min]	Type	Width [min]	Area	Height	Area%	Name
1.239	MM	0.4404	396849.5000	15017.3545	100.0000	
		Sum	396849.5000			

Agilent Technologies

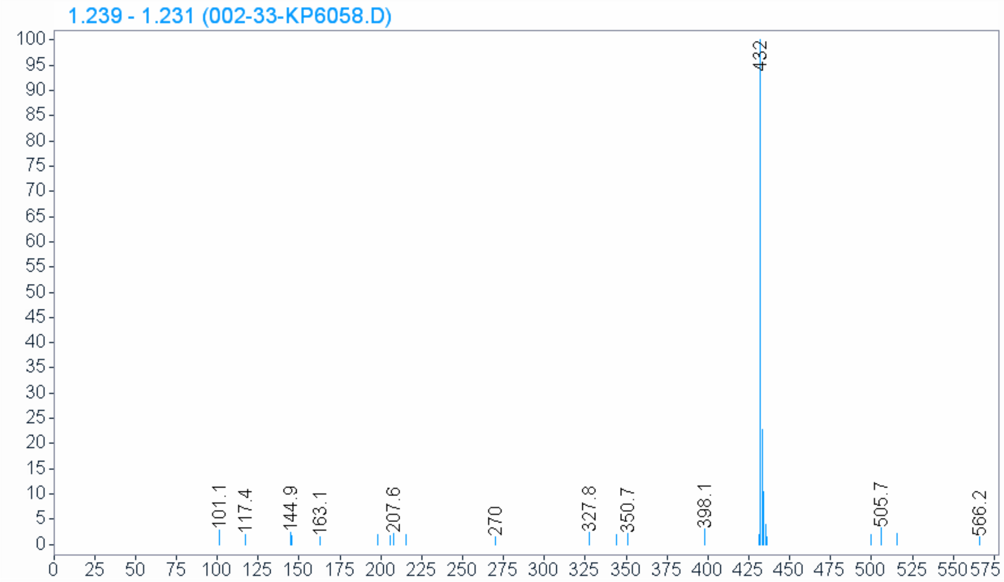

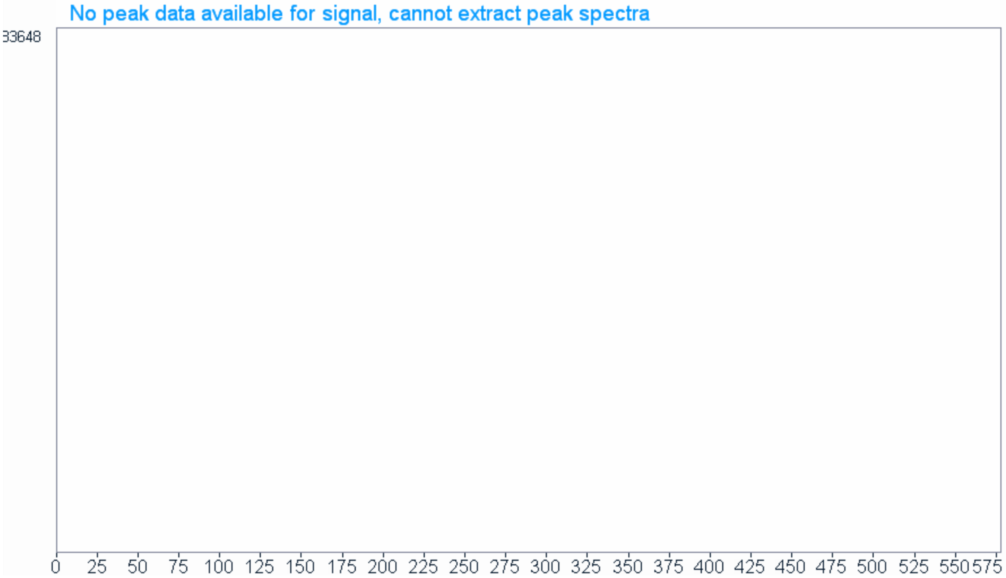

Compound Name: (Z)-*N*-(5-((1-methyl-1H-indol-3-yl)methylene)-4-oxo-4,5-dihydrothiazol-2-yl)naphthalene-1-sulfonamide

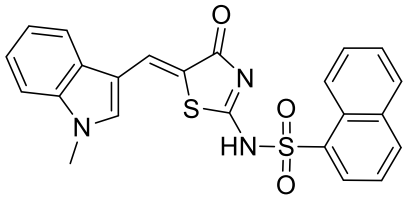

Chemical Formula: $C_{23}H_{17}N_3O_3S_2$
Exact Mass: 447.07
Molecular Weight: 447.53

Compound Code: 61 (KP7071)

Obtained Weight & Yield: 213 mg, 97%

Purity (by LCMS and ^{1}H NMR): > 99% by ^{1}H-NMR, 98% by LCMS

Appearance: yellow solid

Solubility: DMSO, slightly soluble in acetone and methanol

Melting Point: > 289 °C (dec.)

TLC Rf (and conditions): N/A

IR Analysis (including assignment): IR (neat): 2913 (C-H aromatic), 2758 (C-H), 1684 (C=O), 1521 (aromatic C-C), 1299 (sulfonamide), 1122 (C-N) cm^{-1}

^{1}H NMR Analysis: ^{1}H NMR (400 MHz, DMSO) δ 12.92 (br, s, 1H, NH), 8.63 (d, *J* = 8.6 Hz, 1H), 8.33 (dd, *J* = 22.2, 7.7 Hz, 2H), 8.12 (d, *J* = 8.0 Hz, 1H), 8.02 (s, 1H), 8.96 – 7.92 (m, 2H), 7.79 – 7.67 (m, 3H), 7.60 (d, *J* = 8.1 Hz, 1H), 7.34 (t, *J* = 7.5 Hz, 1H), 7.26 (t, *J* = 7.4 Hz, 1H), 4.00 (s, 3H) ppm.
Ethanol at 1.06 ppm (0.38%)

^{13}C NMR Analysis: ^{13}C NMR (101 MHz, DMSO) δ 166.3, 165.3, 137.0, 135.5, 134.6, 133.8, 133.1, 129.0, 128.2, 128.1, 127.7, 127.3, 127.1, 126.1, 124.9, 124.6, 123.3, 121.7, 118.6, 113.6, 111.0, 109.3, 33.4 ppm.

MS Analysis (low res): LRMS (ESI-) *m/z* (%): 447 (*M*-H, $C_{23}H_{16}N_3O_3S_2$, 100%);

MS Analysis (high res): Exact mass calculated for $C_{23}H_{16}N_3O_3S_2$ [*M*-H]$^-$, 446.0600. Found 446.0639.

HPLC method details: Column: Zorbax SB-C18 Rapid Resolution HT 2.1x50mm 1.8-Micron; Method: LCMS ISOCRATIC 60%B 0.4MLMIN-1.M filename: KP7071; Peak retention time: 1.660 mins; Area (%): 98

Procedure: To a 10 mL microwave vial was added *N*-(4-oxo-4,5-dihydrothiazol-2-yl)naphthalene-1-sulfonamide (156 mg, 0.49 mmol), 1-methylindole-3-carboxaldehyde (97 mg, 0.55 mmol, 1.1 eq), ethanol (3 mL) and a catalytic amount of the benzoic acid/piperidine catalyst (approximately 5 drops). The suspension was heated using microwave irradiation (200 W, 120 °C) for 50 min then allowed to precipitate at in the freezer. The resulting precipitate was collected by vacuum filtration and washed with cold ethanol and cold ether to give the desired product (213 mg, 97%).

Other analyses, reference papers, previously obtained data, comments, etc:

PerkinElmer Spectrum Version 10.4.00
Thursday, 21 November 2019 11:24 AM

Analyst	research
Date	Thursday, 21 November 2019 11:24 AM

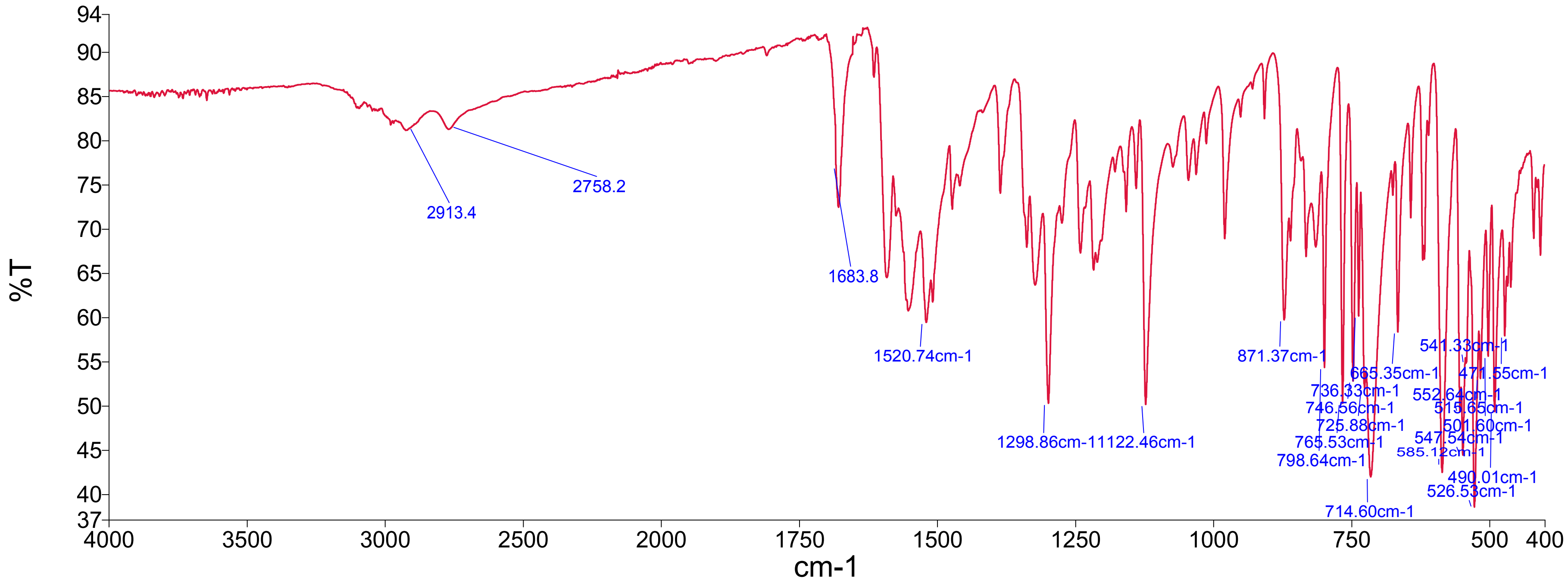

Sample Name	Description	Quality Checks
KP7071	Sample 247 By research Date Thursday, November 21 2019	The Quality Checks do not report any warnings for the sample.

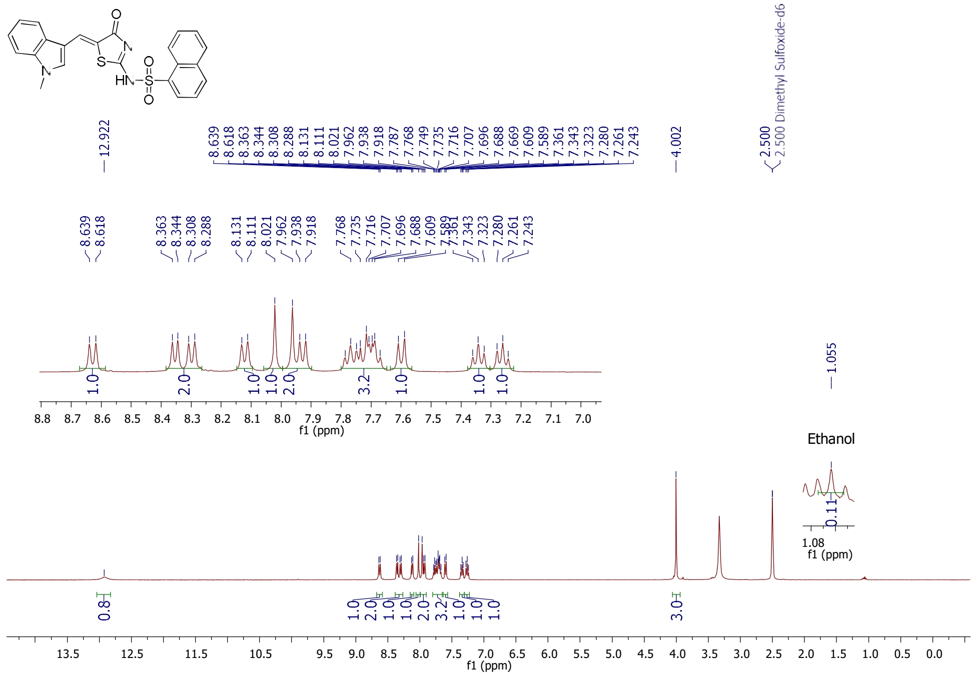

HN
S
O
N
12.922
8.639 8.618 8.363 8.344 8.308 8.288 8.131 8.111 8.021 7.962 7.938 7.918 7.787 7.768 7.749 7.735 7.716 7.707 7.696 7.688 7.669 7.609 7.589 7.361 7.343 7.323 7.280 7.261 7.243
4.002
2.500
2.500 Dimethyl Sulfoxide-d6
1.055
Ethanol
1.08
f1 (ppm)
0.11
0.8
1.0 2.0 1.0 1.0 2.0 3.2 1.0 1.0 1.0
3.0
13.5 12.5 11.5 10.5 9.5 9.0 8.5 8.0 7.5 7.0 6.5 6.0 5.5 5.0 4.5 4.0 3.5 3.0 2.5 2.0 1.5 1.0 0.5 0.0
f1 (ppm)
8.8 8.7 8.6 8.5 8.4 8.3 8.2 8.1 8.0 7.9 7.8 7.7 7.6 7.5 7.4 7.3 7.2 7.1 7.0

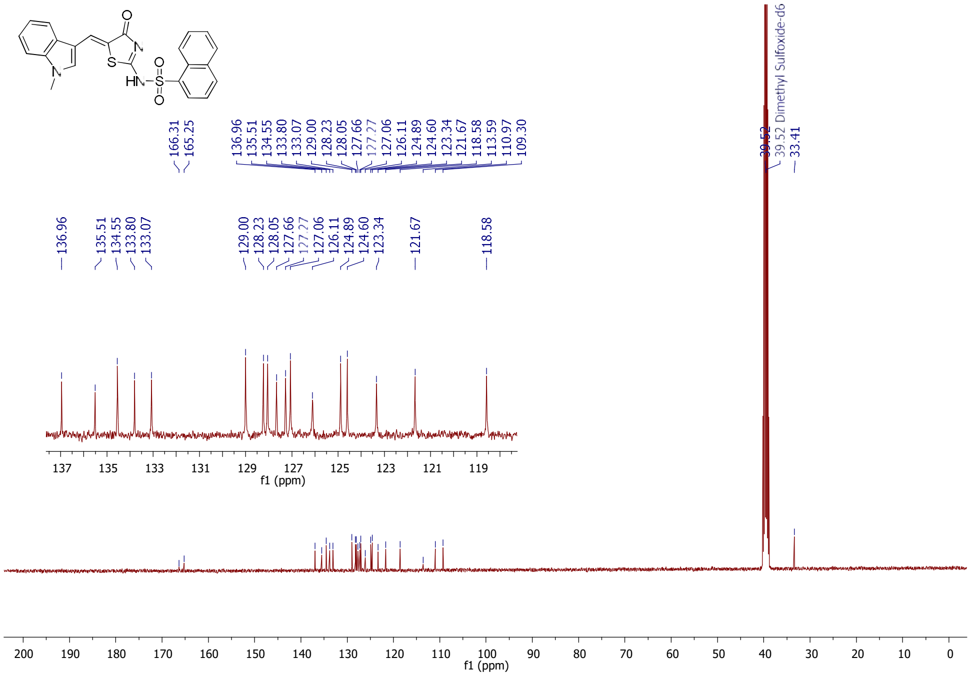

HN
S
O
N
166.31 165.25
136.96 135.51 134.55 133.80 133.07 129.00 128.23 128.05 127.66 127.27 127.06 126.11 124.89 124.60 123.34 121.67 118.58 113.59 110.97 109.30
39.52
39.52 Dimethyl Sulfoxide-d6
33.41
137 135 133 131 129 127 125 123 121 119
f1 (ppm)
200 190 180 170 160 150 140 130 120 110 100 90 80 70 60 50 40 30 20 10 0
f1 (ppm)

LCMS Report

Agilent Technologies

**Data file:**	D:\Chem32\1\Data\KP_DS_IND2 2019-11-15 11-06-04\003-45-KP7071.D
**Sample name:**	KP7071
**Description:**	

**Sample amount:**	0.000	**Sample type:**	Sample
**Instrument:**	LCMS	**Location:**	45
**Injection date:**	11/15/2019 11:23:01 AM	**Injection:**	1 of 1
**Acq. method:**	LCMS ISOCRATIC 60% B 0.4MLMIN-1.M	**Injection volume:**	2.000
**Analysis method:**	LCMS ISOCRATIC 60%B 0.4MLMIN-1.M	**Acq. operator:**	SYSTEM
**Last changed:**	5/8/2019 8:55:04 AM		

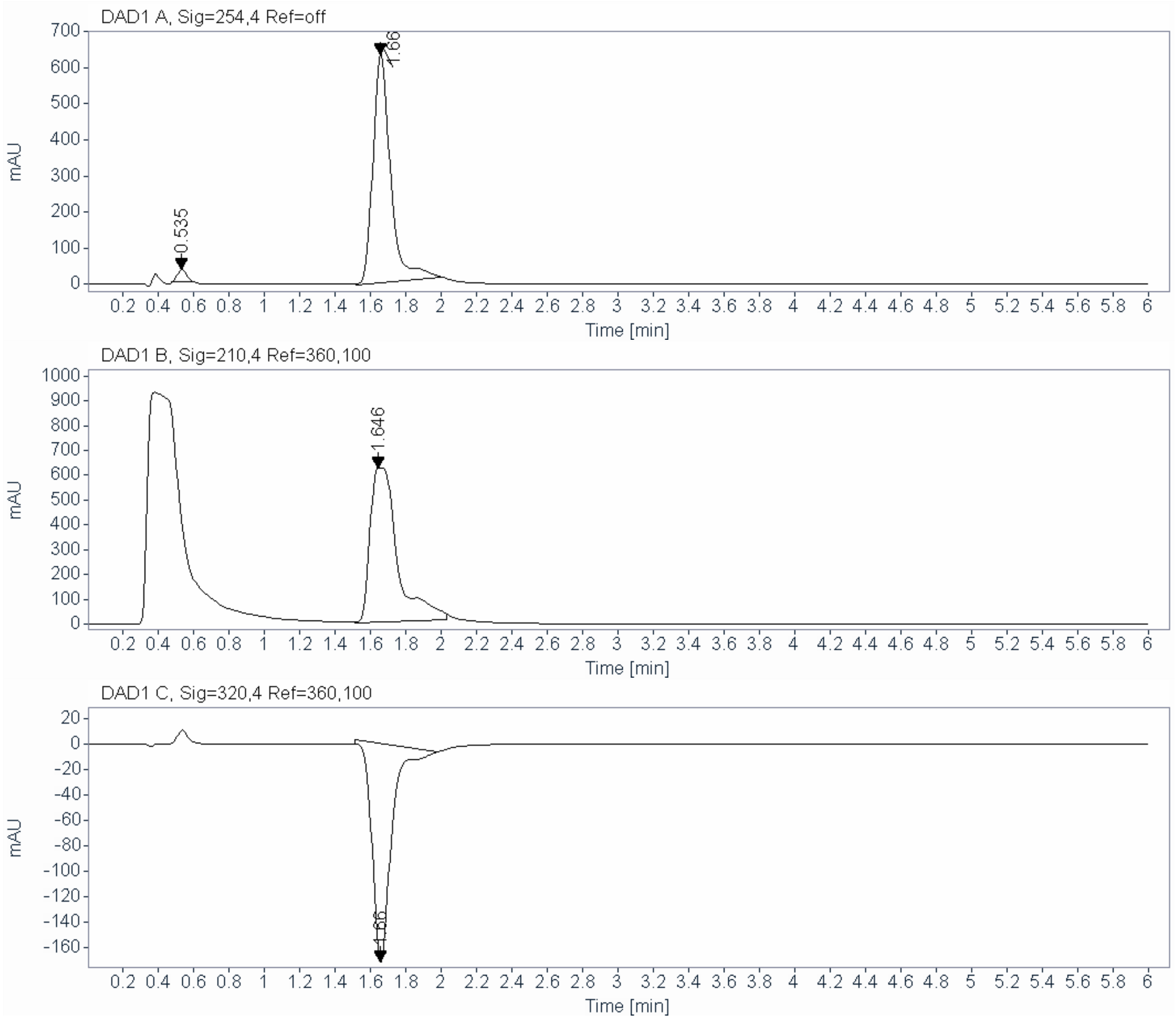

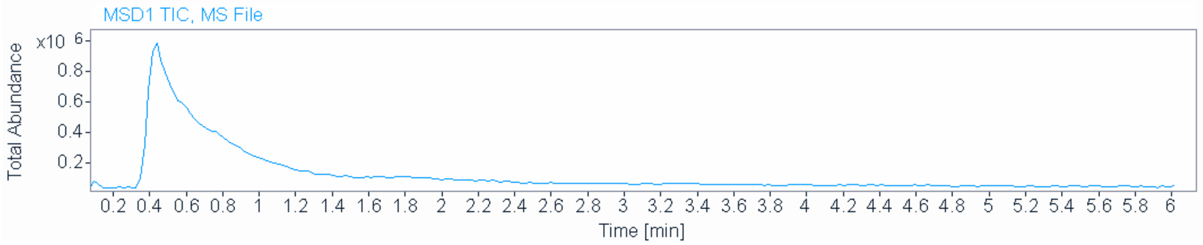

Agilent Technologies

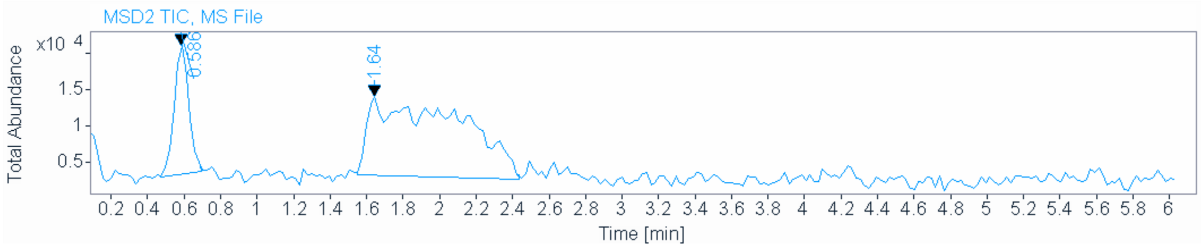

Signal: DAD1 A, Sig=254,4 Ref=off

RT [min]	Type	Width [min]	Area	Height	Area%	Name
0.535	MM	0.0577	113.6670	32.8498	2.4987	
1.660	MM	0.1160	4435.4448	637.1042	97.5013	
		Sum	4549.1118			

Signal: DAD1 B, Sig=210,4 Ref=360,100

RT [min]	Type	Width [min]	Area	Height	Area%	Name
1.646	MM	0.1780	6662.8306	623.7820	100.0000	
		Sum	6662.8306			

Signal: DAD1 C, Sig=320,4 Ref=360,100

RT [min]	Type	Width [min]	Area	Height	Area%	Name
1.660	MP N	0.1175	1218.1979	172.7774	100.0000	
		Sum	1218.1979			

Signal: MSD2 TIC, MS File

RT [min]	Type	Width [min]	Area	Height	Area%	Name
0.586	MM	0.0919	99171.4375	17990.3242	20.8949	
1.640	MM	0.5765	375449.3750	10853.5615	79.1051	
		Sum	474620.8125			

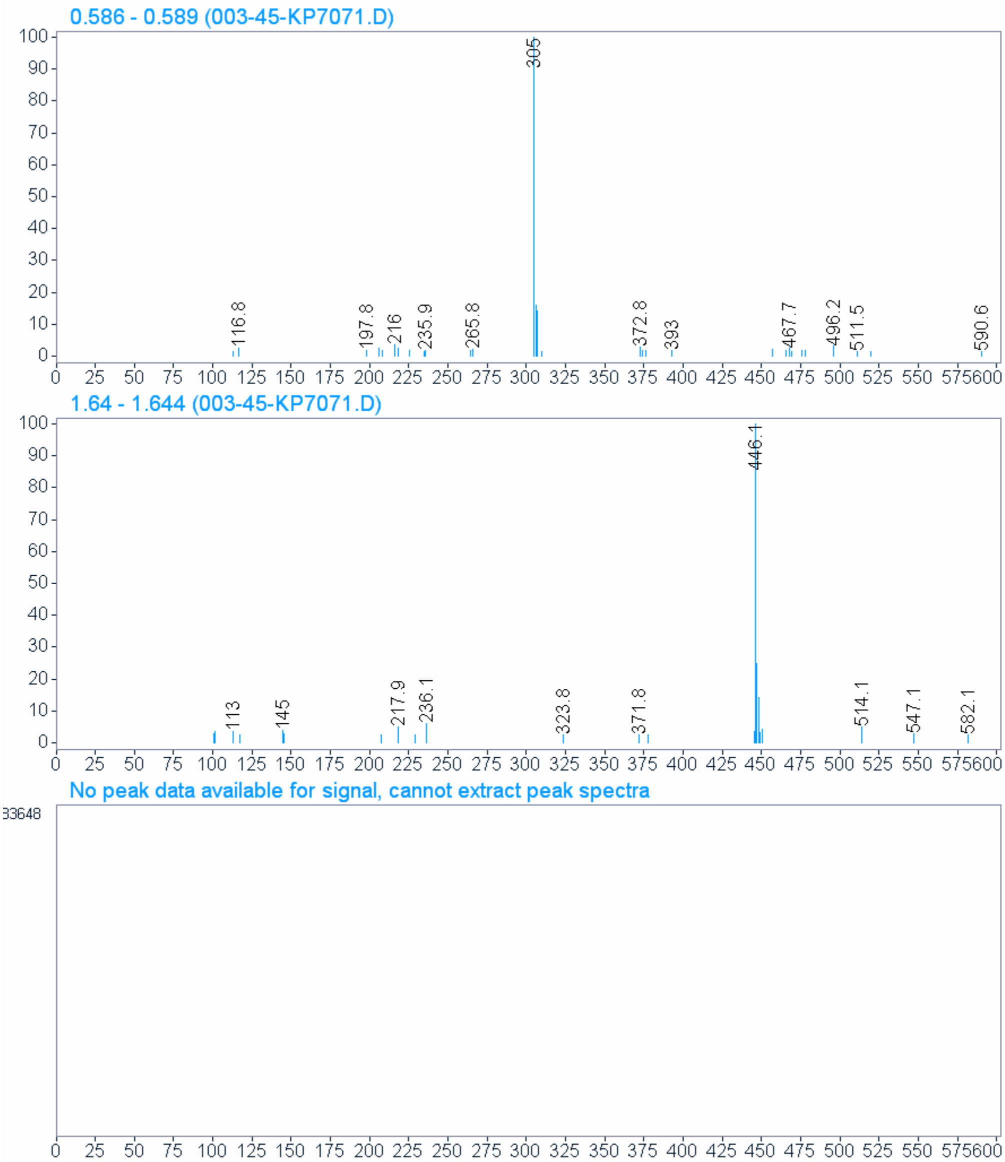
0.586 - 0.589 (003-45-KP7071.D)
305
116.8
197.8
216
235.9
265.8
372.8
393
467.7
496.2
511.5
590.6
1.64 - 1.644 (003-45-KP7071.D)
446.1
113
145
217.9
236.1
323.8
371.8
514.1
547.1
582.1
No peak data available for signal, cannot extract peak spectra
33648

Compound Name: (Z)-*N*-(5-((2-methyl-1H-indol-3-yl)methylene)-4-oxo-4,5-dihydrothiazol-2-yl)naphthalene-1-sulfonamide

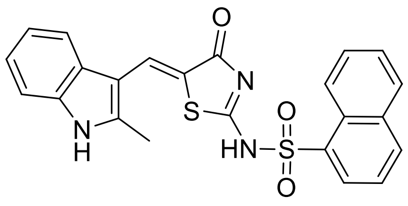

Chemical Formula: $C_{23}H_{17}N_3O_3S_2$
Exact Mass: 447.07
Molecular Weight: 447.53

Compound Code: 62 (KP7064)

Obtained Weight & Yield: 56 mg, 25%

Purity (by LCMS and ^{1}H NMR): > 99% by ^{1}H-NMR and LCMS

Appearance: bright yellow solid

Solubility: DMSO, slightly soluble in acetone and methanol

Melting Point: > 301 °C (dec.)

TLC Rf (and conditions): N/A

IR Analysis (including assignment): IR (neat): 3334 (N-H), 2972 (C-H aromatic), 2881, 2758 (C-H), 1690 (C=O), 1539 (aromatic C-C), 1329 (sulfonamide), 1115 (C-N) cm^{-1}

^{1}H NMR Analysis: ^{1}H NMR (400 MHz, DMSO) δ 12.89 (br, s, 1H, NH), 12.16 (br, s, 1H, NH), 8.60 (d, *J* = 8.4 Hz, 1H), 8.30 – 8.23 (m, 2H), 8.11 (d, *J* = 7.7 Hz, 1H), 7.92 (s, 1H), 7.76 – 7.68 (m, 4H), 7.44 (d, *J* = 6.7 Hz, 1H), 7.24 (s, 2H), 2.53 (s, 3H) ppm.
Ethanol at 1.06 ppm (0.51%).

^{13}C NMR Analysis: ^{13}C NMR (101 MHz, DMSO) δ 166.5, 165.4, 144.4, 136.2, 135.4, 134.5, 133.8, 129.7, 129.0, 128.2 (2C), 127.7, 127.0, 125.0, 124.7, 124.5, 122.5, 121.0, 119.4, 113.0, 112.0, 107.3, 12.4 ppm. 2C determined by 2D NMR

MS Analysis (low res): LRMS (ESI-) *m/z* (%): 446 (*M*-H, $C_{23}H_{16}N_3O_3S_2$, 100%)

MS Analysis (high res): Exact mass calculated for $C_{23}H_{16}N_3O_3S_2$ [*M*-H]$^-$, 446.0600. Found 446.0639.

HPLC method details: Column: Zorbax SB-C18 Rapid Resolution HT 2.1x50mm 1.8-Micron; Method: LCMS ISOCRATIC 60%B 0.4MLMIN-1.M filename: KP7064; Peak retention time: 1.288 mins; Area (%): 100

Procedure: To a 10 mL microwave vial was added *N*-(4-oxo-4,5-dihydrothiazol-2-yl)naphthalene-1-sulfonamide (147 mg, 0.49 mmol), 2-methyl indole-3-carboxaldehyde (88 mg, 0.54 mmol, 1.1 eq), ethanol (3 mL) and a catalytic amount of the benzoic acid/piperidine catalyst (approximately 5 drops). The suspension was heated using microwave irradiation (200 W, 120 °C) for 50 min then allowed to precipitate at in the freezer. The resulting precipitate was collected by vacuum filtration and washed with cold ethanol and cold ether to give the desired product (56 mg, 25%).

Other analyses, reference papers, previously obtained data, comments, etc:

PerkinElmer Spectrum Version 10.4.00
Thursday, 21 November 2019 11:22 AM

Analyst	research
Date	Thursday, 21 November 2019 11:22 AM

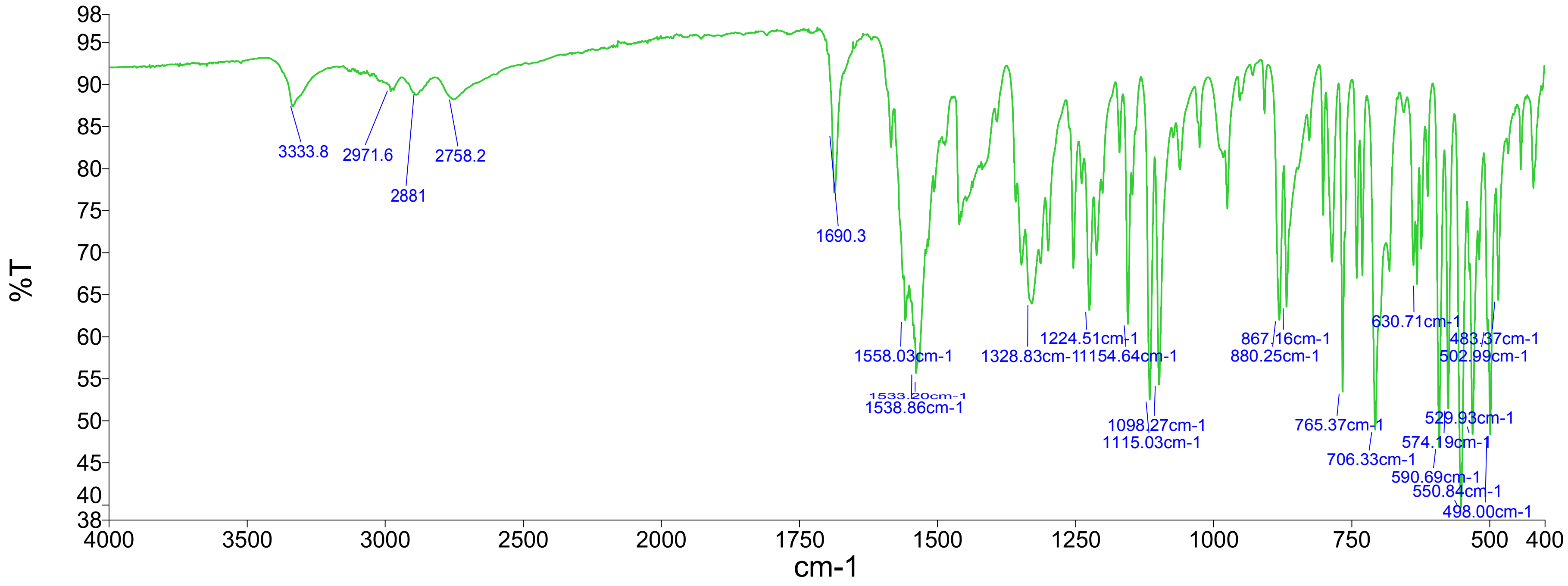

Sample Name	Description	Quality Checks
KP7064	Sample 243 By research Date Thursday, November 21 2019	The Quality Checks do not report any warnings for the sample.

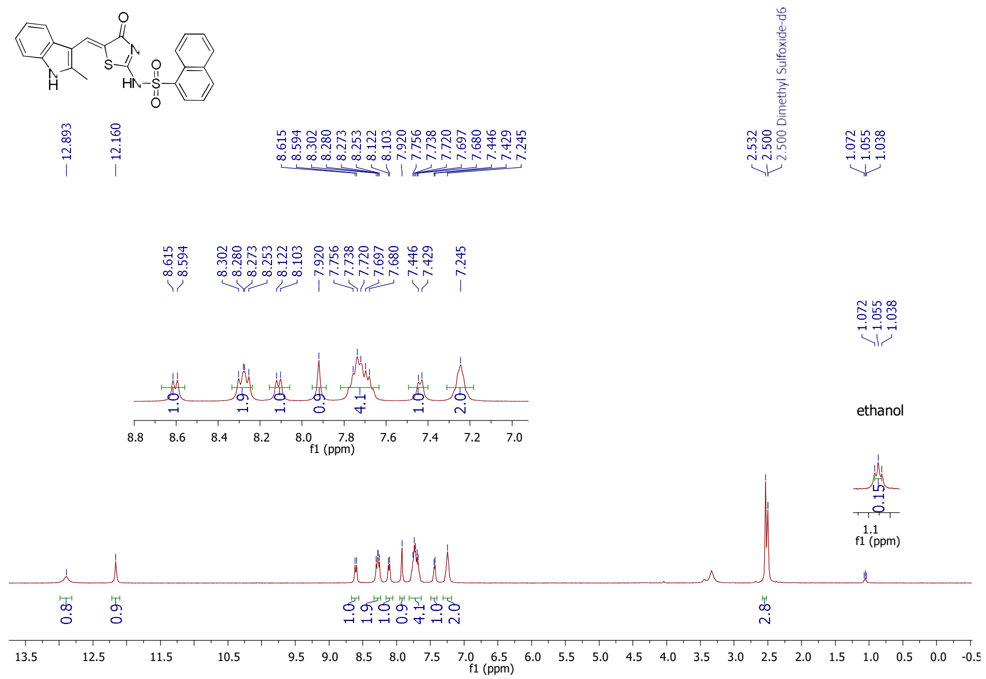

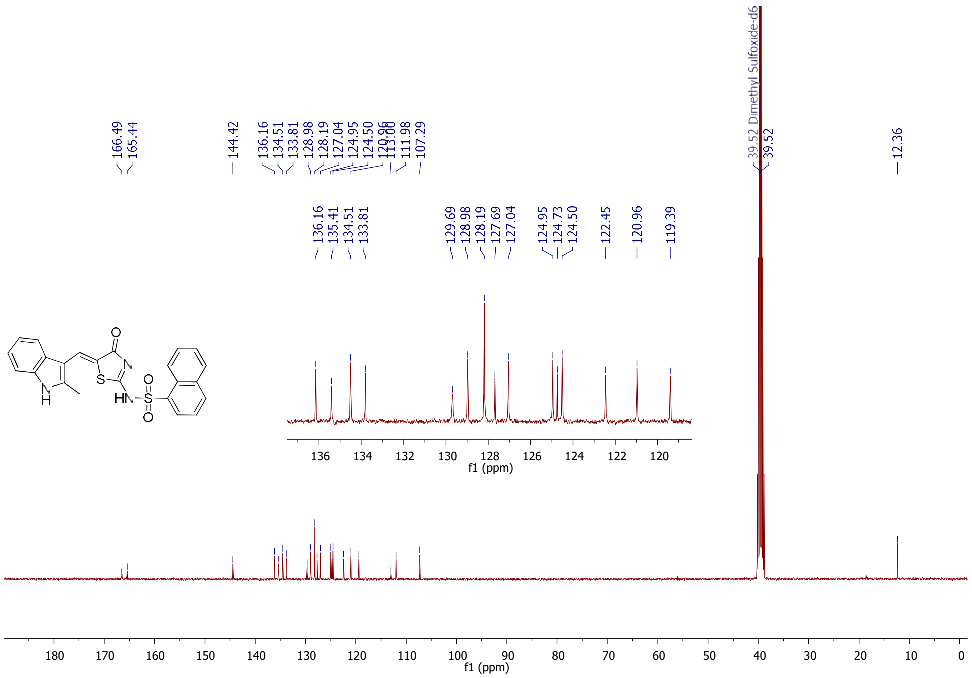

LCMS Report

Agilent Technologies

**Data file:**	D:\Chem32\1\Data\KP\KP_DS_INDOLE1 2019-11-15 10-03-37\004-35-KP6064.D		
**Sample name:**	KP6064		
**Description:**			
**Sample amount:**	0.000	**Sample type:**	Sample
**Instrument:**	LCMS	**Location:**	35
**Injection date:**	11/15/2019 10:28:02 AM	**Injection:**	1 of 1
**Acq. method:**	LCMS ISOCRATIC 60% B 0.4MLMIN-1.M	**Injection volume:**	2.000
**Analysis method:**	LCMS ISOCRATIC 60%B 0.4MLMIN-1.M	**Acq. operator:**	SYSTEM
**Last changed:**	5/8/2019 8:55:04 AM		

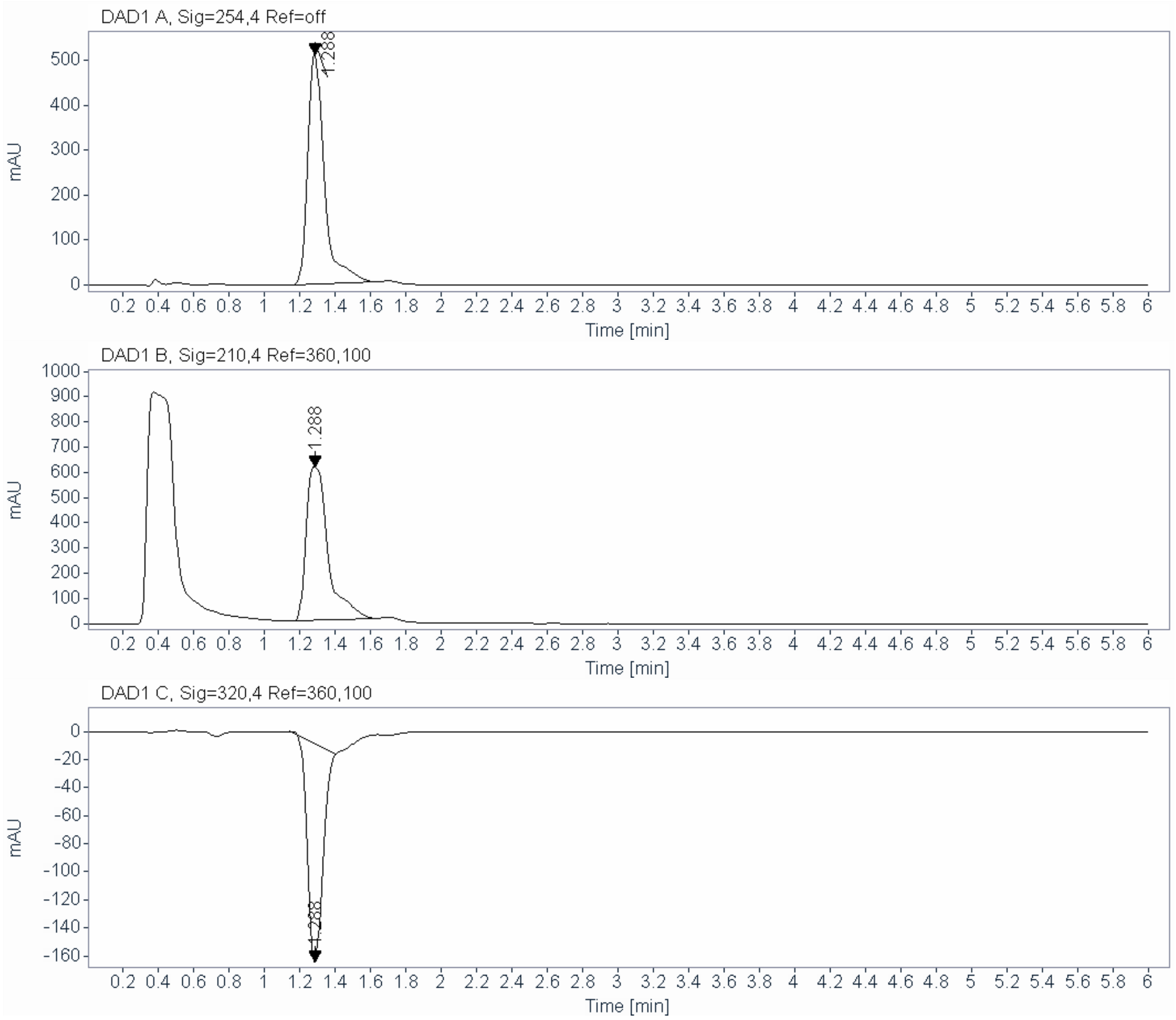

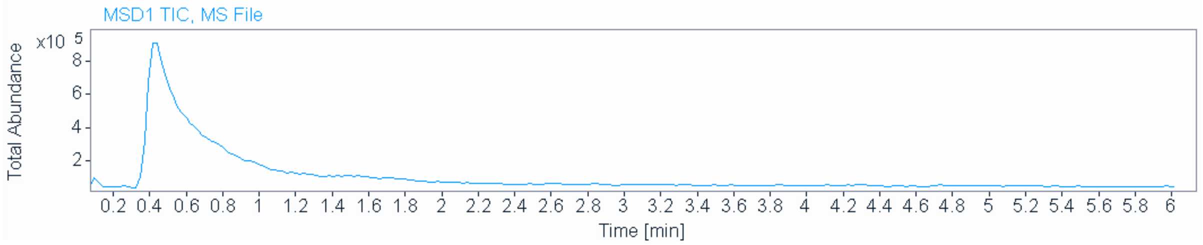

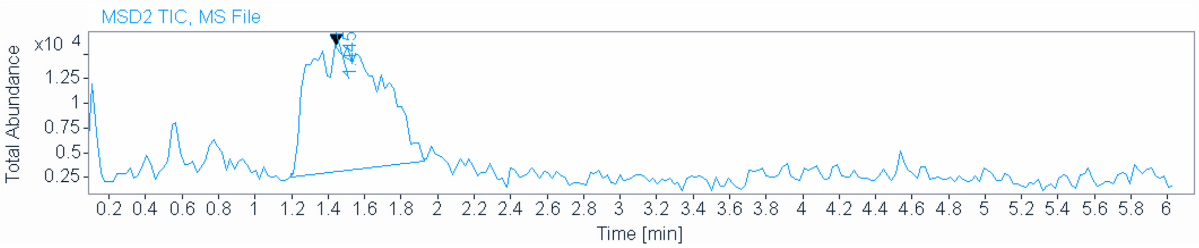

Signal: DAD1 A, Sig=254,4 Ref=off

RT [min]	Type	Width [min]	Area	Height	Area%	Name
1.288	BB	0.1004	3355.4624	512.3193	100.0000	
		Sum	3355.4624			

Signal: DAD1 B, Sig=210,4 Ref=360,100

RT [min]	Type	Width [min]	Area	Height	Area%	Name
1.288	BB	0.1346	5260.5840	610.3260	100.0000	
		Sum	5260.5840			

Signal: DAD1 C, Sig=320,4 Ref=360,100

RT [min]	Type	Width [min]	Area	Height	Area%	Name
1.288	MP N	0.0936	880.4753	156.8554	100.0000	
		Sum	880.4753			

Signal: MSD2 TIC, MS File

RT [min]	Type	Width [min]	Area	Height	Area%	Name
1.445	MM	0.4606	363292.4063	13146.2422	100.0000	
		Sum	363292.4063			

Agilent Technologies

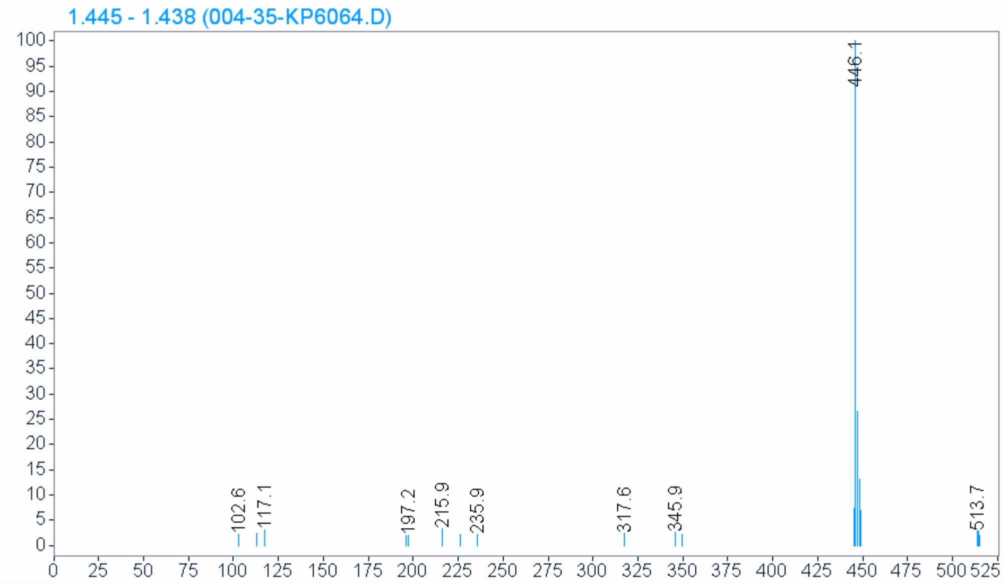

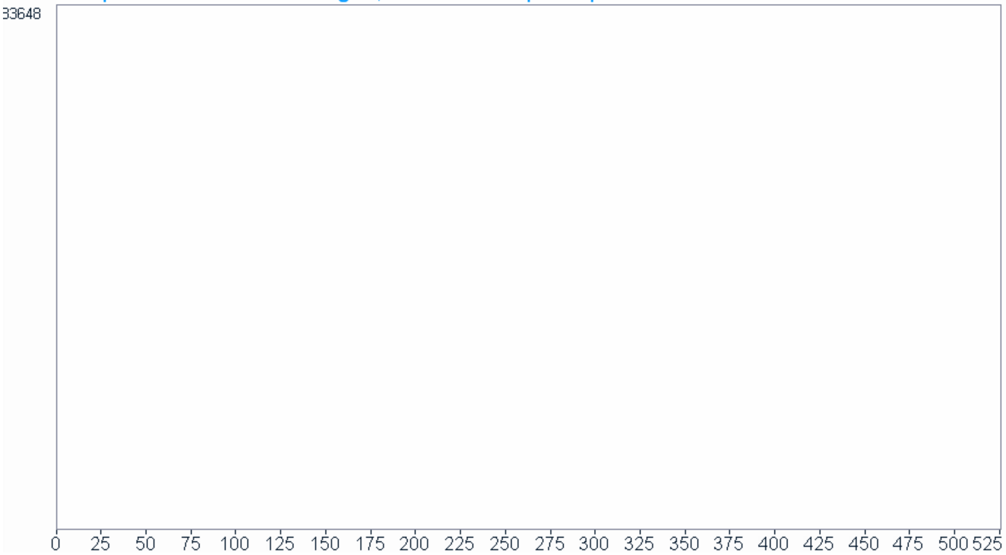

Compound Name: (Z)-*N*-(5-((5-methyl-1H-indol-3-yl)methylene)-4-oxo-4,5-dihydrothiazol-2-yl)naphthalene-1-sulfonamide

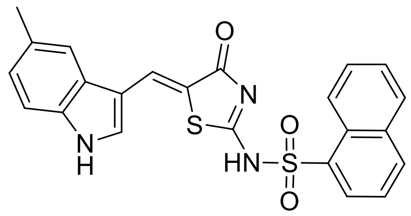

Chemical Formula: $C_{23}H_{17}N_3O_3S_2$
Exact Mass: 447.07
Molecular Weight: 447.53

Compound Code: 63 (KP7063)

Obtained Weight & Yield: 103 mg, 46%

Purity (by LCMS and ^{1}H NMR): > 97% by ^{1}H-NMR and LCMS

Appearance: bright orange solid

Solubility: DMSO, slightly soluble in acetone and methanol

Melting Point: > 278 °C (dec.)

TLC Rf (and conditions): N/A

IR Analysis (including assignment): IR (neat): 3321 (N-H), 2907 (C-H aromatic), 2752 (C-H), 1697 (C=O), 1558 (aromatic C-C), 1322 (sulfonamide), 1126 (C-N) cm^{-1}

^{1}H NMR Analysis: ^{1}H NMR (400 MHz, DMSO) δ 12.91 (br, s, 1H, NH), 12.15 (br, s, 1H, NH), 8.63 (d, *J* = 8.6 Hz, 1H), 8.34 (dd, *J* = 7.35, 1.06 Hz, 1H), 8.29 (d, *J* = 8.29 Hz, 1H), 8.12 (d, *J* = 8.1 Hz, 1H), 8.01 (s, 1H), 7.84 (d, *J* = 3.0 Hz, 1H), 7.79 – 7.75 (m, 1H), 7.72 – 7.66 (m, 3H), 7.43 (d, *J* = 8.3 Hz, 1H), 7.09 (dd, *J* = 8.3, 1.0 Hz, 1H), 2.42 (s, 3H) ppm.
Ether at 1.09 ppm (2.13%)

^{13}C NMR Analysis: ^{13}C NMR (101 MHz, DMSO) δ 166.8, 165.8, 136.0, 135.1, 135.0, 134.3, 130.8, 130.2, 129.5, 128.7, 128.5, 128.1, 127.6, 127.5, 127.2, 125.4, 125.3, 125.1, 118.5, 113.8, 112.8, 110.4, 21.7 ppm.

MS Analysis (low res): LRMS (ESI-) *m/z* (%): 446 (*M*-H, $C_{23}H_{16}N_3O_3S_2$, 100%)

MS Analysis (high res): Exact mass calculated for $C_{23}H_{16}N_3O_3S_2$ [*M*-H]$^-$, 446.0600. Found 446.06381.

HPLC method details: Column: Zorbax SB-C18 Rapid Resolution HT 2.1x50mm 1.8-Micron; Method: LCMS ISOCRATIC 60%B 0.4MLMIN-1.M filename: KP7063; Peak retention time: 0.927 mins; Area (%): 100

Procedure: To a 10 mL microwave vial was added *N*-(4-oxo-4,5-dihydrothiazol-2-yl)naphthalene-1-sulfonamide (147 mg, 0.49 mmol), 5-methylindole-3-carboxaldehyde (90 mg, 0.55 mmol, 1.1 eq), ethanol (3 mL) and a catalytic amount of the benzoic acid/piperidine catalyst (approximately 5 drops). The suspension was heated using microwave irradiation (200 W, 120 °C) for 50 min then allowed to precipitate at in the freezer. The resulting precipitate was collected by vacuum filtration and washed with cold ethanol and cold ether to give the desired product (103 mg, 46%).

Other analyses, reference papers, previously obtained data, comments, etc:

PerkinElmer Spectrum Version 10.4.00
Thursday, 21 November 2019 11:21 AM

Analyst	research
Date	Thursday, 21 November 2019 11:21 AM

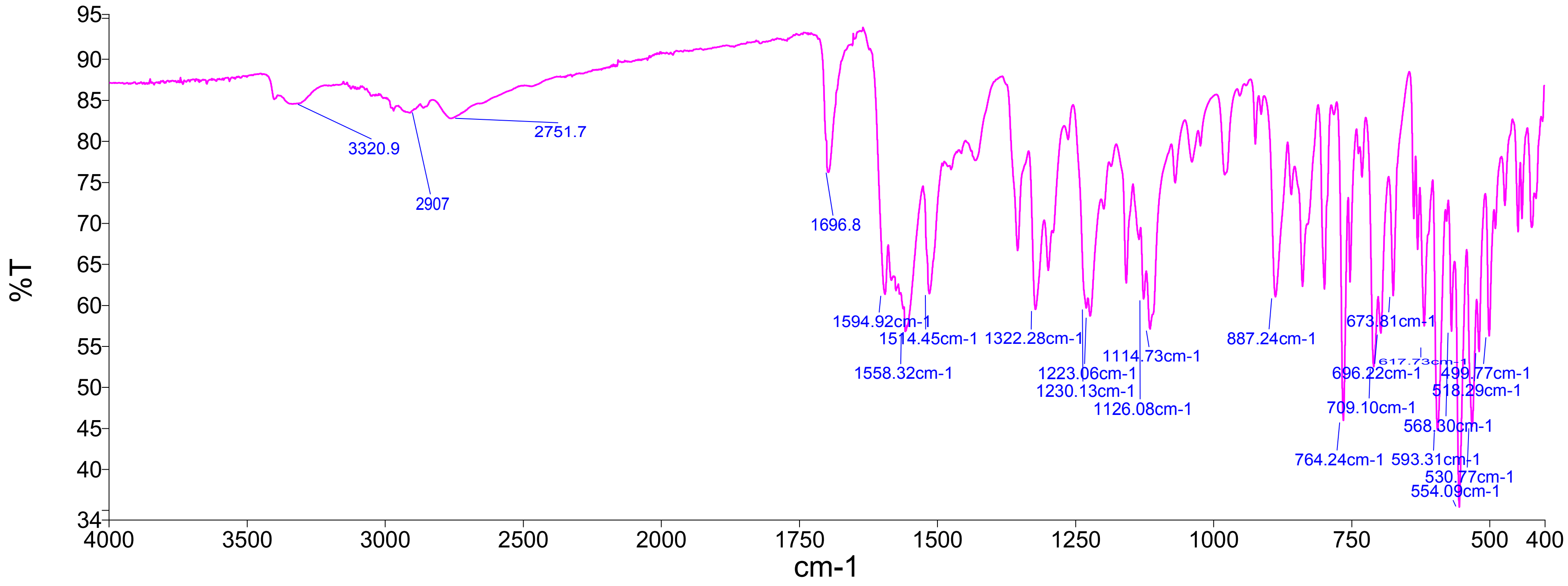

Sample Name	Description	Quality Checks
KP7063	Sample 242 By research Date Thursday, November 21 2019	The Quality Checks do not report any warnings for the sample.

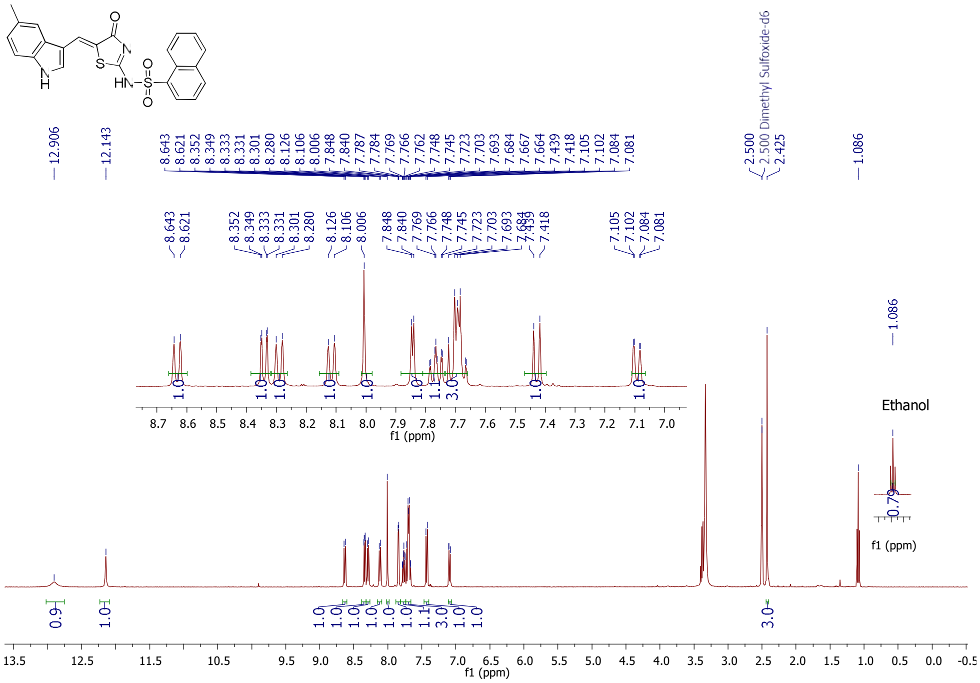

Dimethyl Sulfoxide-d6
Ethanol
f1 (ppm)

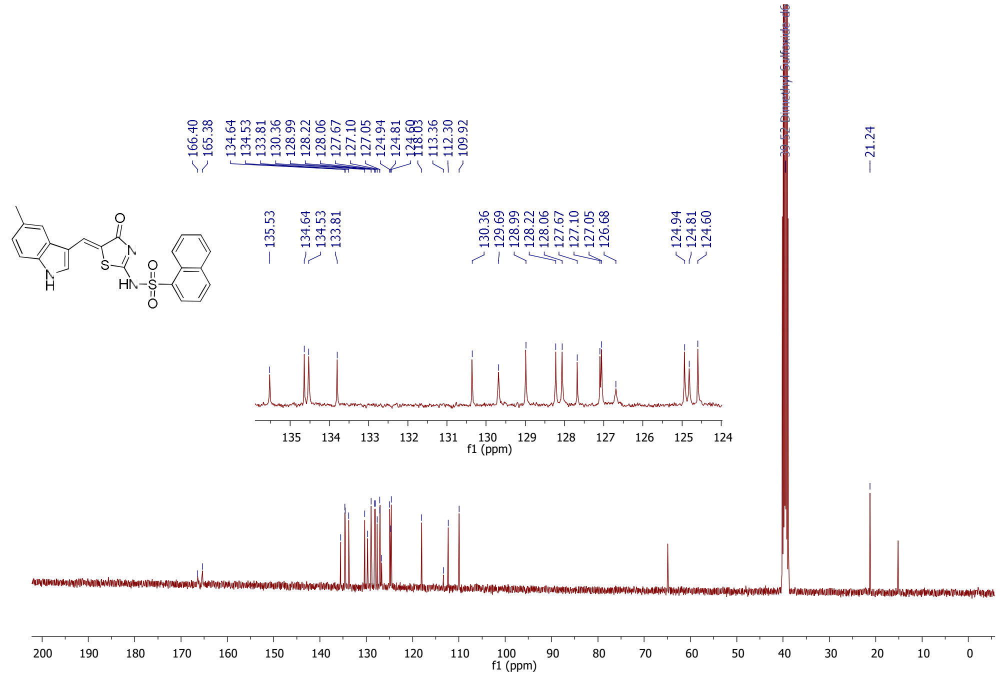

Dimethyl Sulfoxide-d6
f1 (ppm)

LCMS Report

Agilent Technologies

**Data file:**	D:\Chem32\1\Data\KP\KP_DS_INDOLE1 2019-11-15 10-03-37\005-36-KP6063.D		
**Sample name:**	KP6063		
**Description:**			
**Sample amount:**	0.000	**Sample type:**	Sample
**Instrument:**	LCMS	**Location:**	36
**Injection date:**	11/15/2019 10:35:36 AM	**Injection:**	1 of 1
**Acq. method:**	LCMS ISOCRATIC 60% B 0.4MLMIN-1.M	**Injection volume:**	2.000
**Analysis method:**	LCMS ISOCRATIC 60%B 0.4MLMIN-1.M	**Acq. operator:**	SYSTEM
**Last changed:**	5/8/2019 8:55:04 AM		

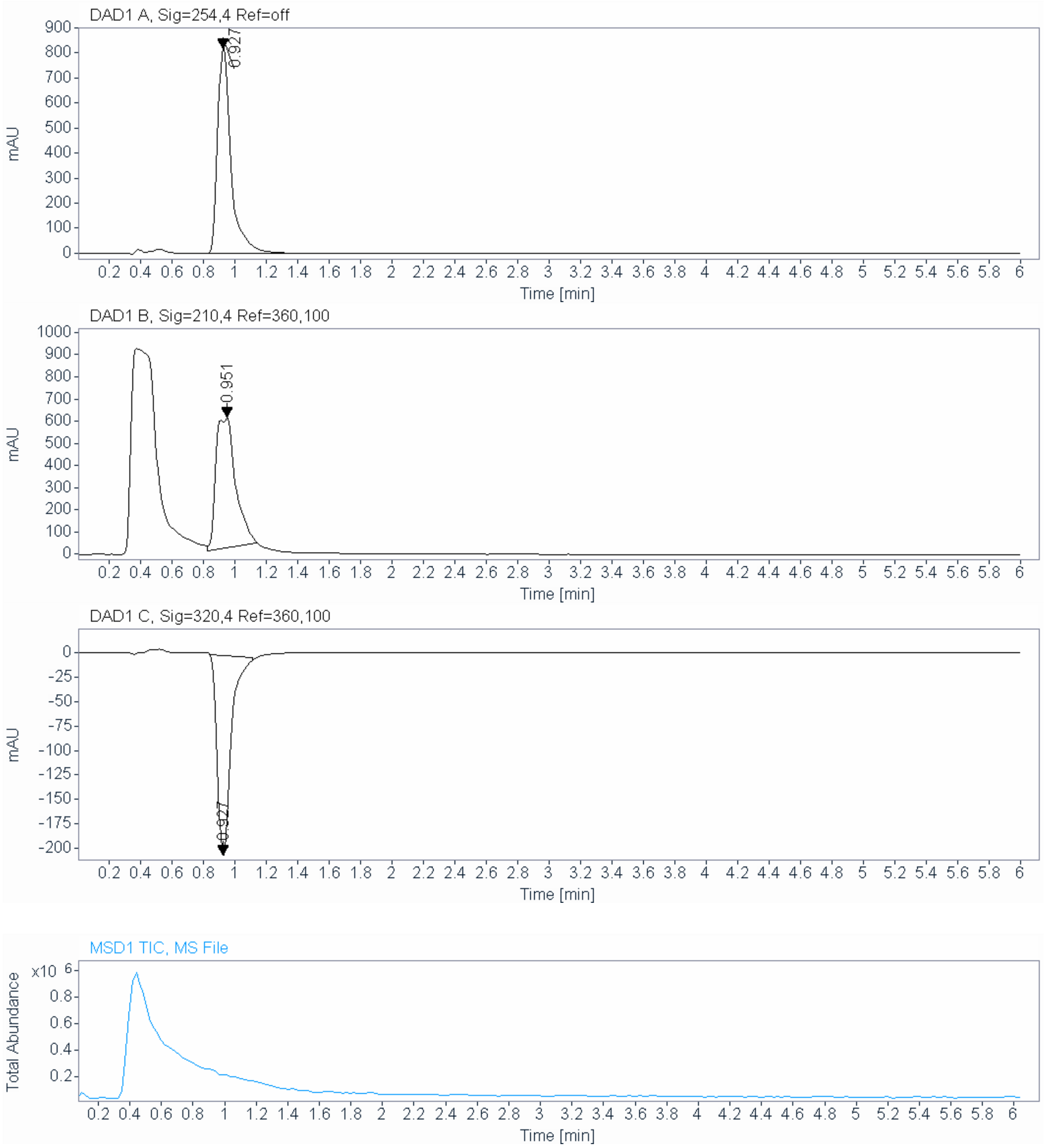

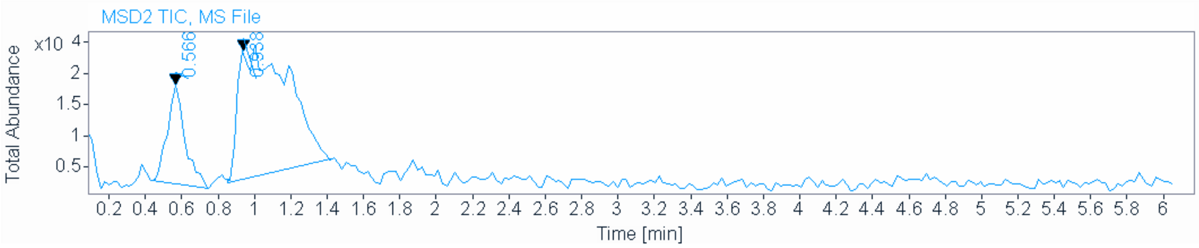

Signal: DAD1 A, Sig=254,4 Ref=off

RT [min]	Type	Width [min]	Area	Height	Area%	Name
0.927	BB	0.0891	4866.3955	821.3731	100.0000	
		Sum	4866.3955			

Signal: DAD1 B, Sig=210,4 Ref=360,100

RT [min]	Type	Width [min]	Area	Height	Area%	Name
0.951	MM	0.1446	5094.6196	587.2233	100.0000	
		Sum	5094.6196			

Signal: DAD1 C, Sig=320,4 Ref=360,100

RT [min]	Type	Width [min]	Area	Height	Area%	Name
0.927	PM N	0.0930	1147.6462	205.7534	100.0000	
		Sum	1147.6462			

Signal: MSD2 TIC, MS File

RT [min]	Type	Width [min]	Area	Height	Area%	Name
0.566	BB	0.0996	106967.6953	15803.9131	21.3228	
0.938	MM	0.3140	394689.9375	20951.6738	78.6772	
		Sum	501657.6328			

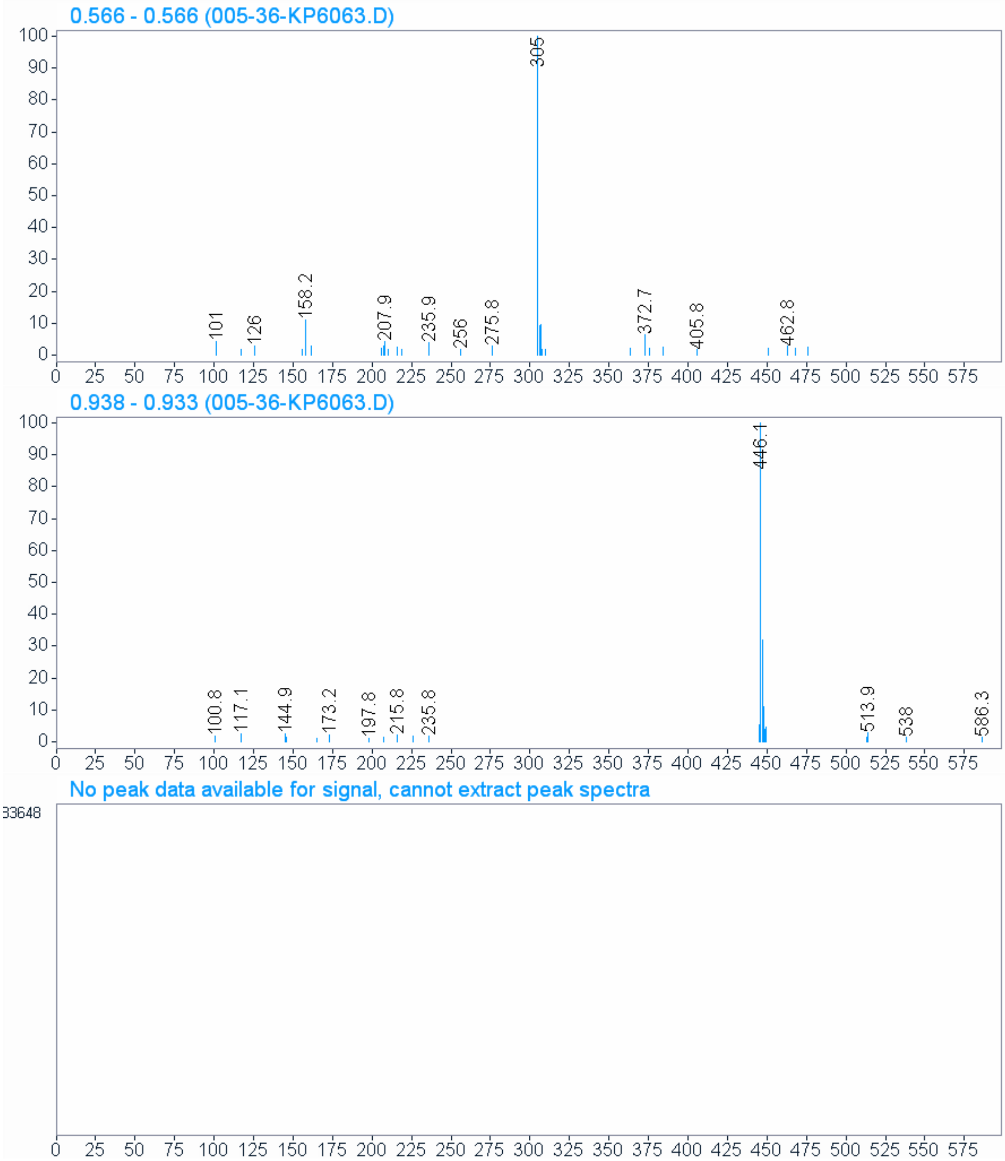
0.566 - 0.566 (005-36-KP6063.D)
305
101
126
158.2
207.9
235.9
256
275.8
372.7
405.8
462.8
0.938 - 0.933 (005-36-KP6063.D)
446.1
100.8
117.1
144.9
173.2
197.8
215.8
235.8
513.9
538
586.3
No peak data available for signal, cannot extract peak spectra
33648

Compound Name: (Z)-*N*-(5-((6-bromo-1H-indol-3-yl)methylene)-4-oxo-4,5-dihydrothiazol-2-yl)naphthalene-1-sulfonamide

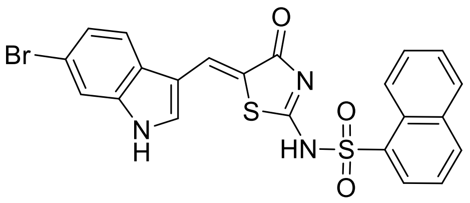

Chemical Formula: $C_{22}H_{14}BrN_3O_3S_2$
Exact Mass: 510.97
Molecular Weight: 512.40

Compound Code: 64 (KP7070)

Obtained Weight & Yield: 114 mg, 45%

Purity (by LCMS and ^{1}H NMR): > 99% by ^{1}H-NMR and LCMS

Appearance: yellow solid

Solubility: DMSO, slightly soluble in acetone and methanol

Melting Point: > 297 °C (dec.)

TLC Rf (and conditions): N/A

IR Analysis (including assignment): IR (neat): 3289 (N-H), 2901 (C-H aromatic), 2745 (C-H), 1687 (C=O), 1549 (aromatic C-C), 1284 (sulfonamide), 1113 (C-N), 709 (C-Br) cm^{-1}

^{1}H NMR Analysis: ^{1}H NMR (400 MHz, DMSO) δ 12.96 (br, s, 1H, NH), 12.27 (br, s, 1H, NH), 8.63 (d, *J* = 8.5 Hz, 1H), 8.32 (dd, *J* = 18.8, 7.7 Hz, 2H), 8.11 (d, *J* = 8.0 Hz, 1H), 8.02 (s, 1H), 7.91 (dd, *J* = 14.4, 5.3 Hz, 2H), 7.79 – 7.67 (m, 4H), 7.32 (d, *J* = 8.2 Hz, 1H) ppm.
Ethanol at 1.05 ppm (0.54%)

^{13}C NMR Analysis: ^{13}C NMR (101 MHz, DMSO) δ 166.2, 165.2, 137.2, 135.4, 134.6, 133.8, 130.0, 129.0, 128.3, 128.1, 127.7, 127.1, 126.1, 125.8, 124.9, 124.6, 124.1, 120.5, 115.8, 115.2, 114.9, 110.4 ppm.

MS Analysis (low res): LRMS (ESI-) *m/z* (%): 512 (*M*-H, $C_{22}H_{13}{}^{81}BrN_3O_3S_2$, 100), 510 (*M*-H, $C_{22}H_{13}{}^{79}BrN_3O_3S_2$, 90); LRMS (ESI+) *m/z* (%): 514 (*M*+H, $C_{22}H_{15}{}^{81}BrN_3O_3S_2$, 40), 512 (*M*+H, $C_{22}H_{15}{}^{79}BrN_3O_3S_2$, 30)

MS Analysis (high res): Exact mass calculated for $C_{22}H_{13}{}^{79}BrN_3O_3S_2$ [*M*-H]$^-$, 509.9583. Found 509.9586. Exact mass calculated for $C_{22}H_{13}{}^{81}BrN_3O_3S_2$ [*M*-H]$^-$, 511.9563. Found 511.9564.

HPLC method details: Column: Zorbax SB-C18 Rapid Resolution HT 2.1x50mm 1.8-Micron; Method: LCMS ISOCRATIC 60%B_3MINS.M filename: KP7070; Peak retention time: 1.813 mins; Area (%): 99

Procedure: To a 10 mL microwave vial was added *N*-(4-oxo-4,5-dihydrothiazol-2-yl)naphthalene-1-sulfonamide (157 mg, 0.49 mmol), 6-bromoindole-3-carboxaldehyde (120 mg, 0.54 mmol, 1.1 eq), ethanol (3 mL) and a catalytic amount of the benzoic acid/piperidine catalyst (approximately 5 drops). The suspension was heated using microwave irradiation (200 W, 120 °C) for 50 min then allowed to precipitate at in the freezer. The resulting precipitate was collected by vacuum filtration and washed with cold ethanol and cold ether to give the desired product (114 mg, 45%).

Other analyses, reference papers, previously obtained data, comments, etc:

PerkinElmer Spectrum Version 10.4.00
Thursday, 21 November 2019 11:23 AM

Analyst research
Date Thursday, 21 November 2019 11:23 AM

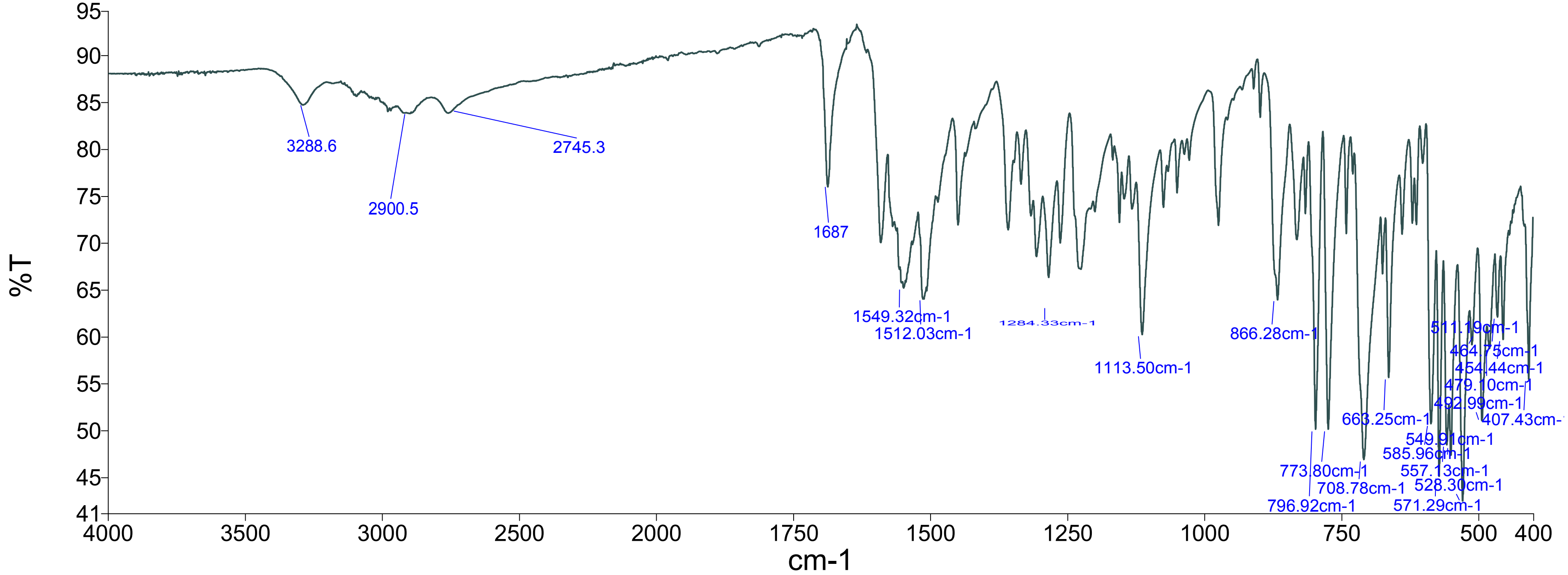

Sample Name	Description	Quality Checks
KP7070	Sample 246 By research Date Thursday, November 21 2019	The Quality Checks give rise to a Negative Bands warning for the sample.

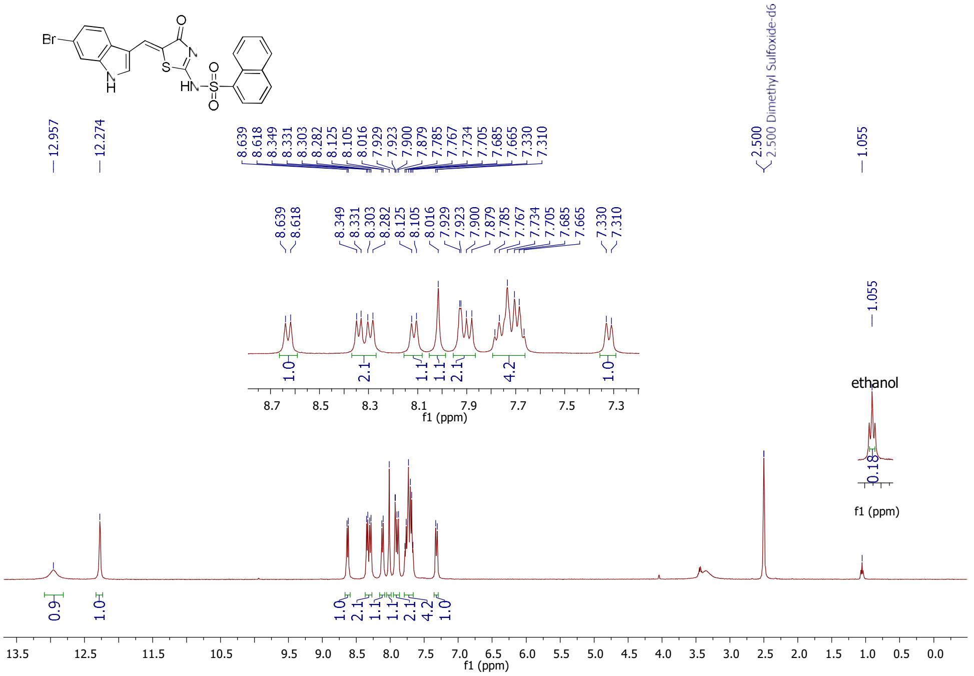
Br
N
H
O
N
S
HN
S
O
O
12.957
12.274
8.639 8.618 8.349 8.331 8.303 8.282 8.125 8.105 8.016 7.929 7.923 7.900 7.879 7.785 7.767 7.734 7.705 7.685 7.665 7.330 7.310
2.500
2.500 Dimethyl Sulfoxide-d6
1.055
ethanol
0.9
1.0
1.0
2.1
1.1
1.1
2.1
4.2
1.0
0.18
f1 (ppm)

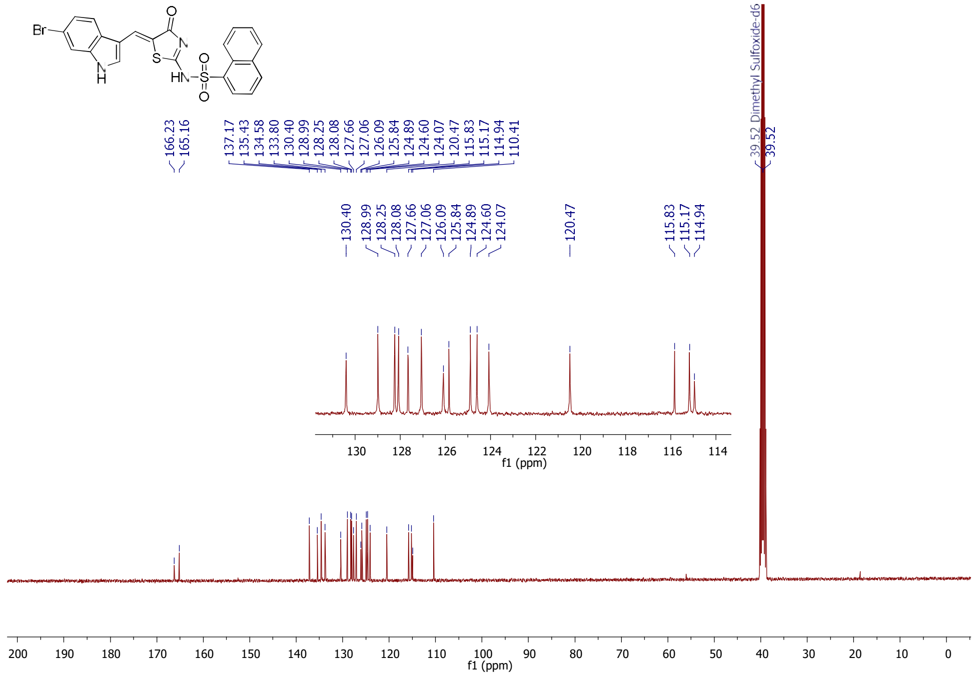
Br
N
H
O
N
S
HN
S
O
O
166.23
165.16
137.17 135.43 134.58 133.80 130.40 128.99 128.25 128.08 127.66 127.06 126.09 125.84 124.89 124.60 124.07 120.47 115.83 115.17 114.94 110.41
39.52 Dimethyl Sulfoxide-d6
39.52
f1 (ppm)

LCMS Report

Agilent Technologies

**Data file:**	D:\Chem32\1\Data\KP\KP_DS_9JULY2020_4 2020-07-09 12-03-22\002-37-KP7070.D		
**Sample name:**	KP7070		
**Description:**			
**Sample amount:**	0.000	**Sample type:**	Sample
**Instrument:**	LCMS	**Location:**	37
**Injection date:**	7/9/2020 12:09:35 PM	**Injection:**	1 of 1
**Acq. method:**	LCMS ISOCRATIC 60% B_3MINS.M	**Injection volume:**	2.000
**Analysis method:**	LCMS ISOCRATIC 60%B_3MINS.M	**Acq. operator:**	SYSTEM
**Last changed:**	5/19/2016 3:52:53 PM		

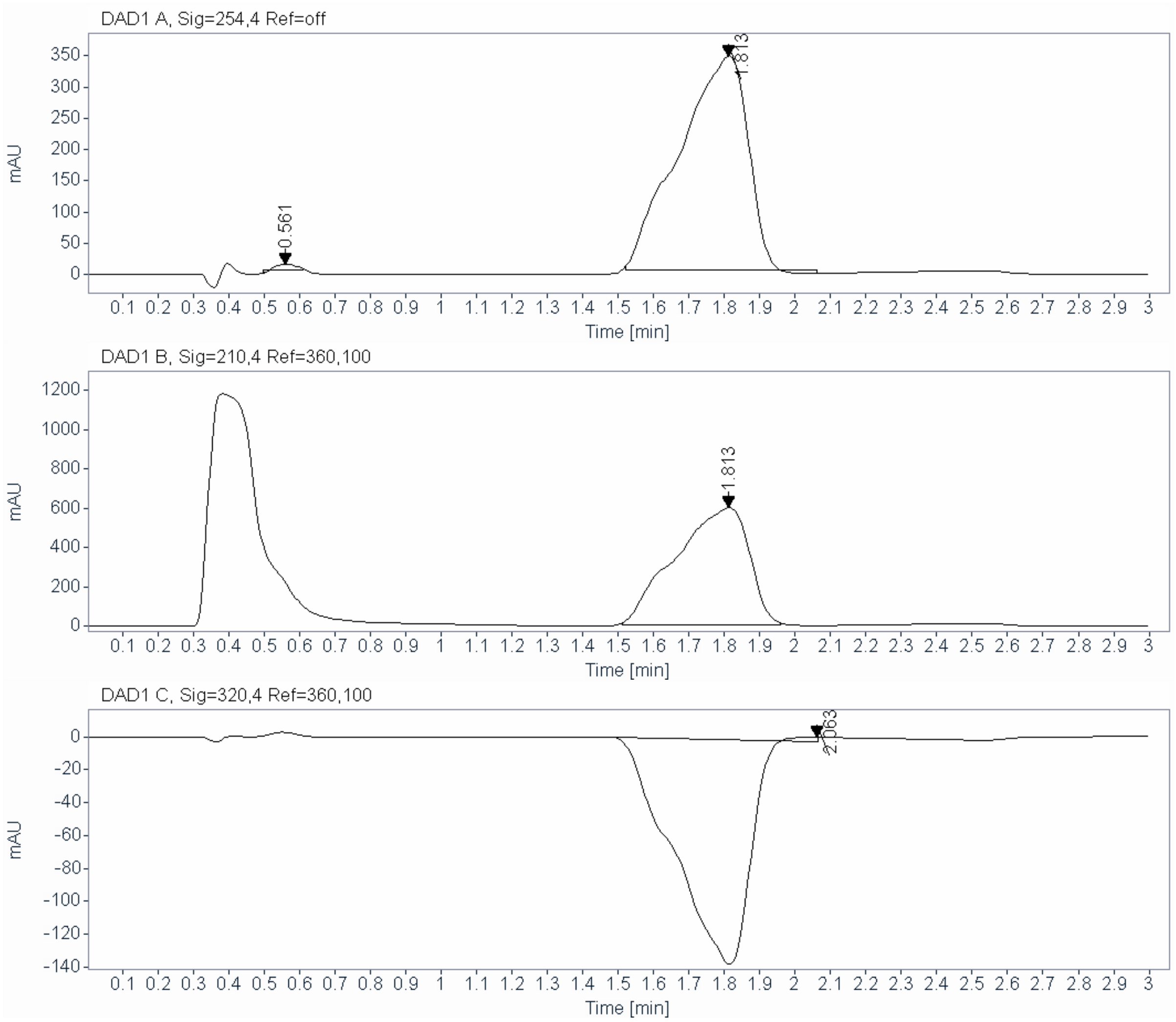

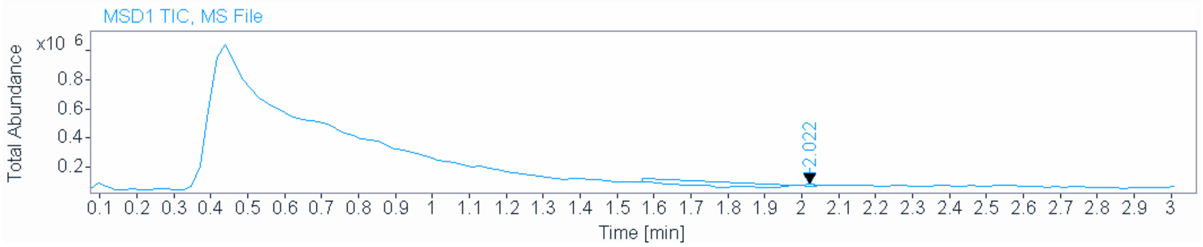

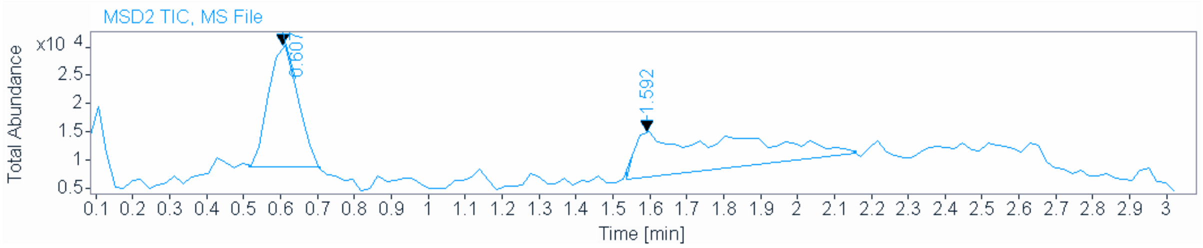

Signal: DAD1 A, Sig=254,4 Ref=off

RT [min]	Type	Width [min]	Area	Height	Area%	Name
0.561	MM	0.0666	39.1551	9.7994	0.8410	
1.813	MM	0.2239	4616.3535	343.6964	99.1590	
		Sum	4655.5087			

Signal: DAD1 B, Sig=210,4 Ref=360,100

RT [min]	Type	Width [min]	Area	Height	Area%	Name
1.813	MM	0.2418	8685.7715	598.6639	100.0000	
		Sum	8685.7715			

Signal: DAD1 C, Sig=320,4 Ref=360,100

RT [min]	Type	Width [min]	Area	Height	Area%	Name
2.063	MM	0.0836	13.0493	2.6002	100.0000	
		Sum	13.0493			

Signal: MSD1 TIC, MS File

RT [min]	Type	Width [min]	Area	Height	Area%	Name
2.022	MM	0.0490	34935.8086	11895.0176	100.0000	
		Sum	34935.8086			

Signal: MSD2 TIC, MS File

RT [min]	Type	Width [min]	Area	Height	Area%	Name
0.607	MM	0.0898	117911.9219	21873.2578	44.4171	
1.592	MM	0.2956	147553.4375	8319.1465	55.5829	
		Sum	265465.3594			

0.607 - 0.612 (002-37-KP7070.D)

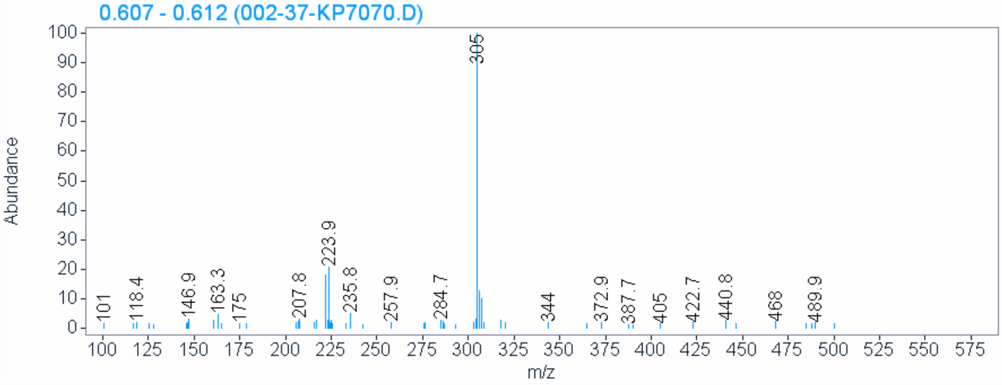

1.592 - 1.598 (002-37-KP7070.D)

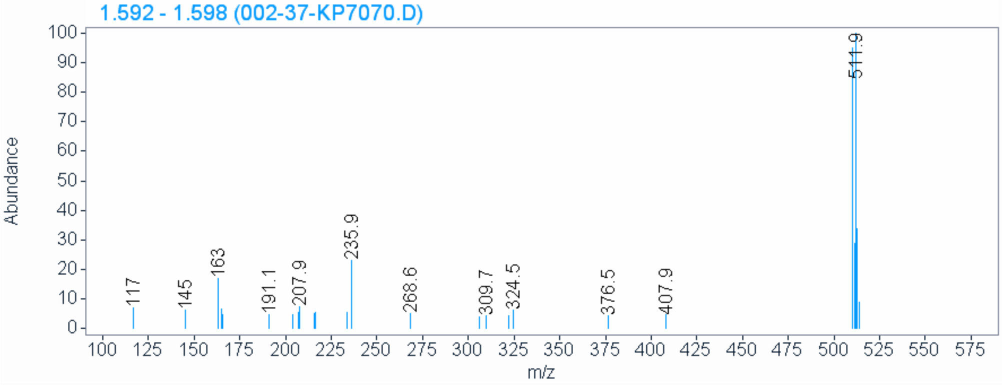

2.022 - 2.022 (002-37-KP7070.D)

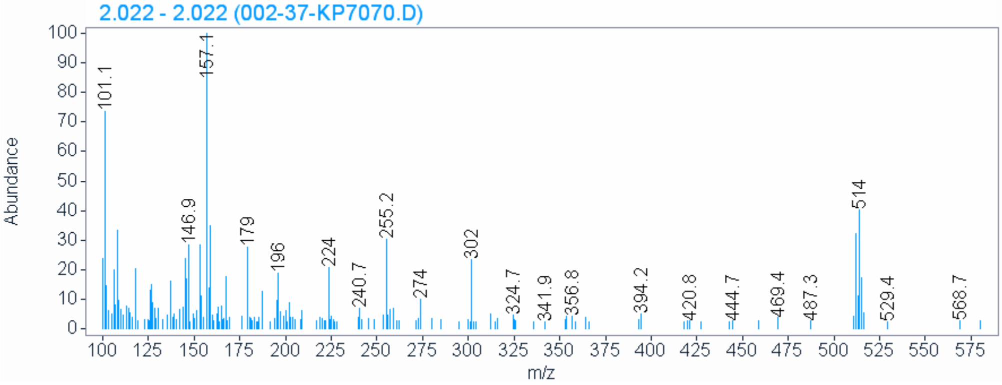

Compound Name: (Z)-*N*-(5-((5-bromo-1H-indol-3-yl)methylene)-4-oxo-4,5-dihydrothiazol-2-yl)naphthalene-1-sulfonamide

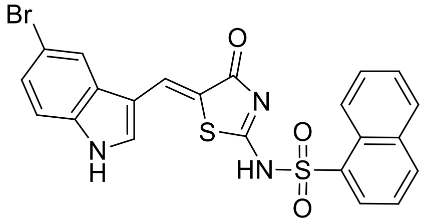

Chemical Formula: $C_{22}H_{14}BrN_3O_3S_2$
Exact Mass: 510.97
Molecular Weight: 512.40

Compound Code: 65 (KP7068)

Obtained Weight & Yield: 105 mg, 42%

Purity (by LCMS and ^{1}H NMR): > 99% by ^{1}H-NMR and LCMS

Appearance: yellow solid

Solubility: DMSO, slightly soluble in acetone and methanol

Melting Point: > 309 °C (dec.)

TLC Rf (and conditions): N/A

IR Analysis (including assignment): IR (neat): 3392 (N-H), 3192 (C-H aromatic), 1700 (C=O), 1558 (aromatic C-C), 1290 (sulfonamide), 11108 (C-N), 565 (C-Br) cm^{-1}

^{1}H NMR Analysis: ^{1}H NMR (400 MHz, DMSO) δ 12.94 (br, s, 1H, NH), 12.35 (br, s, 1H, NH), 8.63 (d, *J* = 8.5 Hz, 1H), 8.34 (d, *J* = 7.32 Hz, 1H), 8.29 (d, *J* = 8.21 Hz, 1H), 8.20 (s, 1H), 8.12 (d, *J* = 8.1 Hz, 1H), 8.07 (s, 1H), 7.94 (d, *J* = 2.7 Hz, 1H), 7.77 (t, *J* = 7.2 Hz, 1H), 7.70 (q, *J* = 7.5 Hz, 2H), 7.51 (d, *J* = 8.6 Hz, 1H), 7.38 (dd, *J* = 8.6, 1.7 Hz, 1H) ppm.

^{13}C NMR Analysis: ^{13}C NMR (101 MHz, DMSO) δ 165.4 (br), 135.6, 135.1, 134.5, 133.8, 130.7, 129.0, 128.6, 128.2, 128.0, 127.7, 127.1, 126.3 (br), 125.8, 124.9, 124.6, 121.3, 114.6, 114.0, 110.1 ppm.
Broad peaks determined by 2D NMR.
2 quaternary carbons missing.

MS Analysis (low res): LRMS (ESI-) *m/z* (%): 510 (*M*-H+H, $C_{22}H_{14}{}^{79}BrN_3O_3S_2$, 80%), 512 (*M*-H+H, $C_{22}H_{14}{}^{81}BrN_3O_3S_2$, 100%)

MS Analysis (high res): Exact mass calculated for $C_{22}H_{13}{}^{79}BrN_3O_3S_2$ [*M*-H]$^-$, 509.9583. Found 509.9585. Exact mass calculated for $C_{22}H_{13}{}^{81}BrN_3O_3S_2$ [*M*-H]$^-$, 511.9563. Found 511.9563.

HPLC method details: Column: Zorbax SB-C18 Rapid Resolution HT 2.1x50mm 1.8-Micron; Method: LCMS ISOCRATIC 60%B 0.4MLMIN-1.M filename: KP7064; Peak retention time: 1.515 mins; Area (%): 100

Procedure: To a 10 mL microwave vial was added *N*-(4-oxo-4,5-dihydrothiazol-2-yl)naphthalene-1-sulfonamide (149 mg, 0.49 mmol), 5-bromoindole-3-carboxaldehyde (127 mg, 0.54 mmol, 1.1 eq), ethanol (3 mL) and a catalytic amount of the benzoic acid/piperidine catalyst (approximately 5 drops). The suspension was heated using microwave irradiation (200 W, 120 °C) for 50 min then allowed to precipitate at in the freezer. The resulting precipitate was collected by vacuum filtration and washed with cold ethanol and cold ether to give the desired product (105 mg, 42%).

Other analyses, reference papers, previously obtained data, comments, etc:

PerkinElmer Spectrum Version 10.4.00
Thursday, 21 November 2019 11:22 AM

Analyst research
Date Thursday, 21 November 2019 11:22 AM

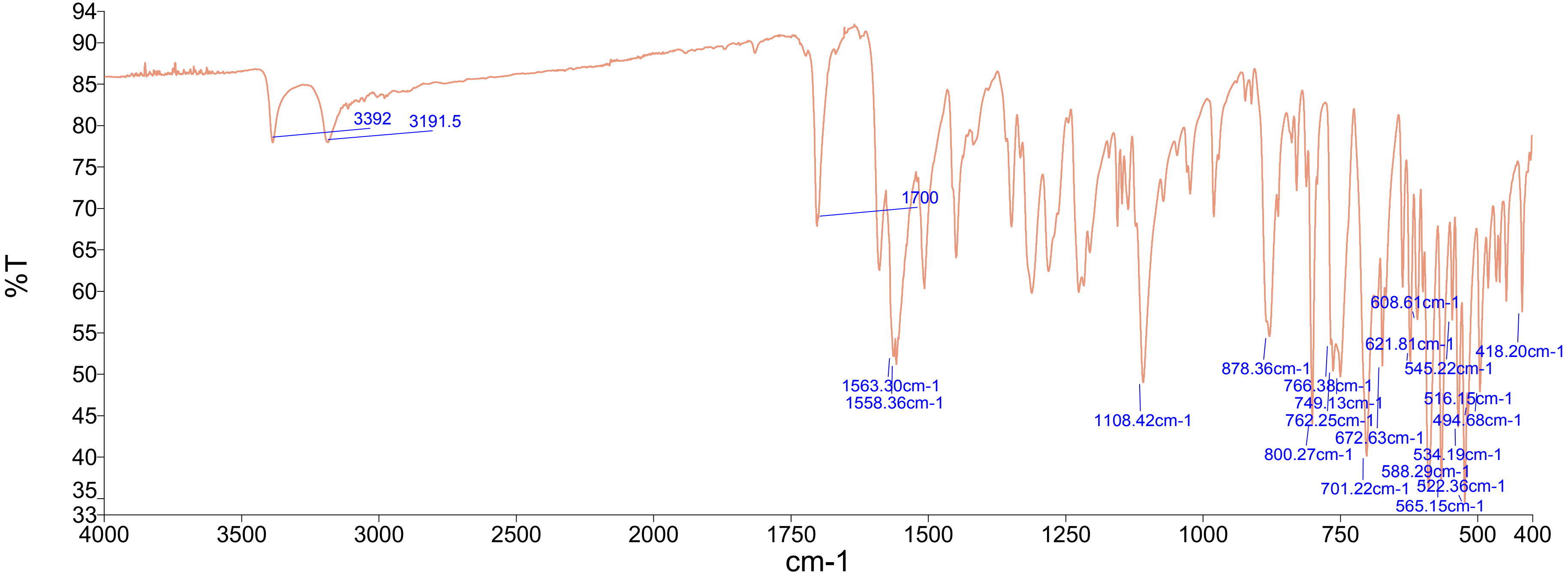

Sample Name	Description	Quality Checks
KP7068	Sample 244 By research Date Thursday, November 21 2019	The Quality Checks do not report any warnings for the sample.

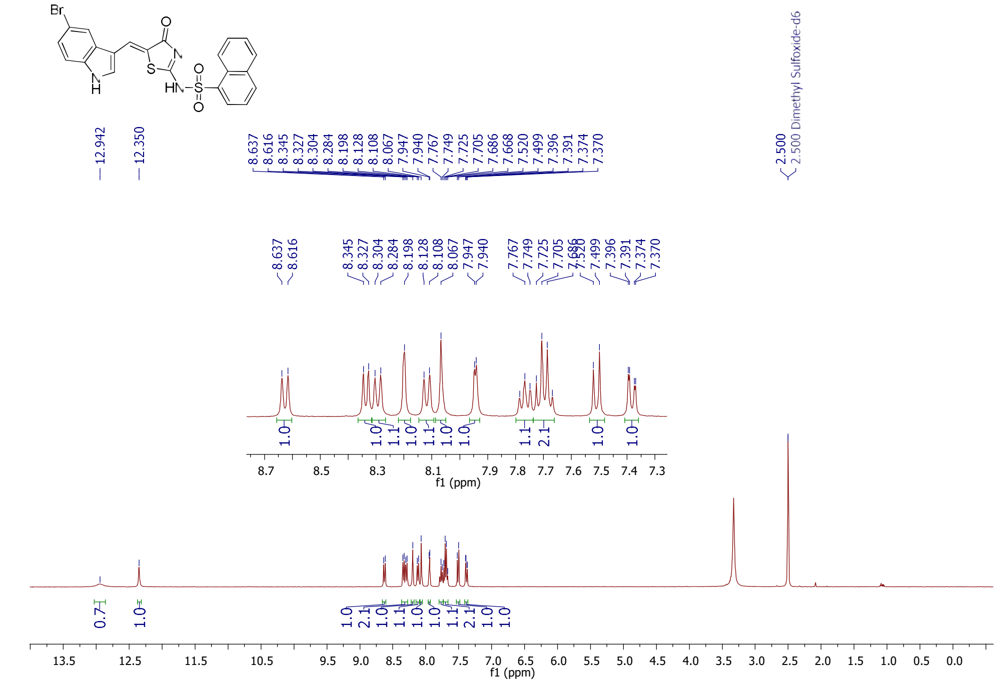
Br
H
N
O
S
HN
12.942
12.350
8.637 8.616 8.345 8.327 8.304 8.284 8.198 8.128 8.108 8.067 7.947 7.940 7.767 7.749 7.725 7.705 7.686 7.668 7.520 7.499 7.396 7.391 7.374 7.370
2.500
2.500 Dimethyl Sulfoxide-d6
f1 (ppm)

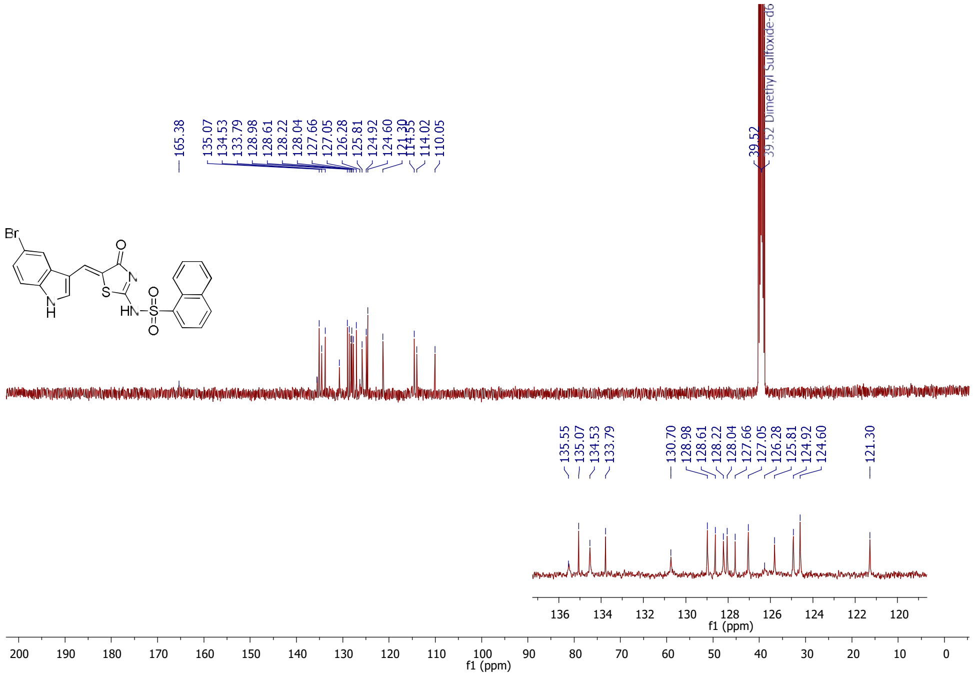
165.38
135.07 134.53 133.79 128.98 128.61 128.22 128.04 127.66 127.05 126.28 125.81 124.92 124.60 121.30 114.55 114.02 110.05
39.52
39.52 Dimethyl Sulfoxide-d6
Br
H
N
O
S
HN
135.55 135.07 134.53 133.79
130.70 128.98 128.61 128.22 128.04 127.66 127.05 126.28 125.81 124.92 124.60
121.30
f1 (ppm)

LCMS Report

Agilent Technologies

**Data file:**	D:\Chem32\1\Data\KP\KP_DS_INDOLE1 2019-11-15 10-03-37\006-37-KP6068.D		
**Sample name:**	KP6068		
**Description:**			
**Sample amount:**	0.000	**Sample type:**	Sample
**Instrument:**	LCMS	**Location:**	37
**Injection date:**	11/15/2019 10:43:13 AM	**Injection:**	1 of 1
**Acq. method:**	LCMS ISOCRATIC 60% B 0.4MLMIN-1.M	**Injection volume:**	2.000
**Analysis method:**	LCMS ISOCRATIC 60%B 0.4MLMIN-1.M	**Acq. operator:**	SYSTEM
**Last changed:**	5/8/2019 8:55:04 AM		

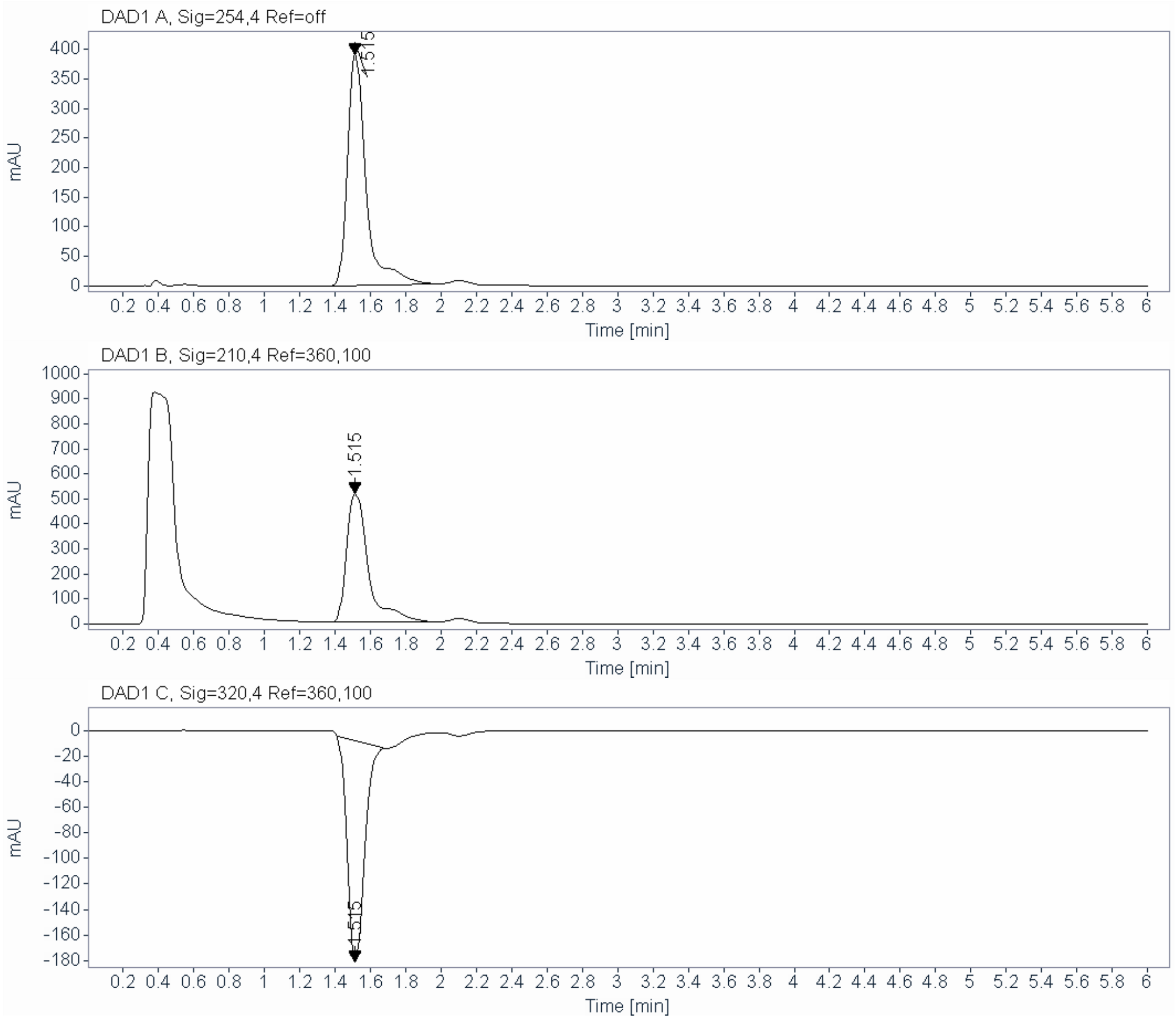

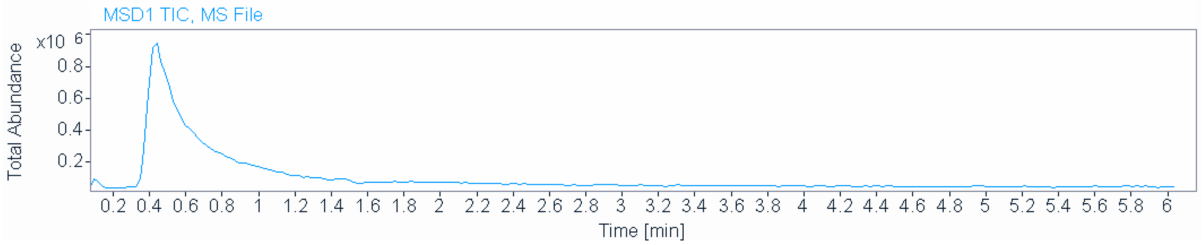

LCMS Report

Agilent Technologies

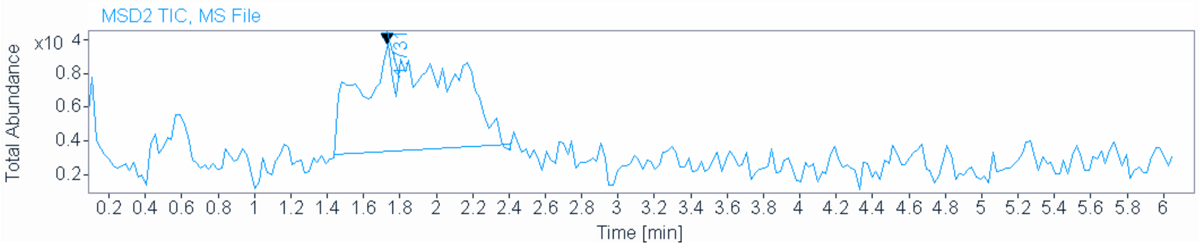

Signal: DAD1 A, Sig=254,4 Ref=off

RT [min]	Type	Width [min]	Area	Height	Area%	Name
1.515	BB	0.1112	2856.8542	391.1441	100.0000	
		Sum	2856.8542			

Signal: DAD1 B, Sig=210,4 Ref=360,100

RT [min]	Type	Width [min]	Area	Height	Area%	Name
1.515	BB	0.1326	4434.2207	514.3192	100.0000	
		Sum	4434.2207			

Signal: DAD1 C, Sig=320,4 Ref=360,100

RT [min]	Type	Width [min]	Area	Height	Area%	Name
1.515	PM N	0.1040	1083.3744	173.6945	100.0000	
		Sum	1083.3744			

Signal: MSD2 TIC, MS File

RT [min]	Type	Width [min]	Area	Height	Area%	Name
1.731	MM	0.5324	209823.4063	6569.0635	100.0000	
		Sum	209823.4063			

Agilent Technologies

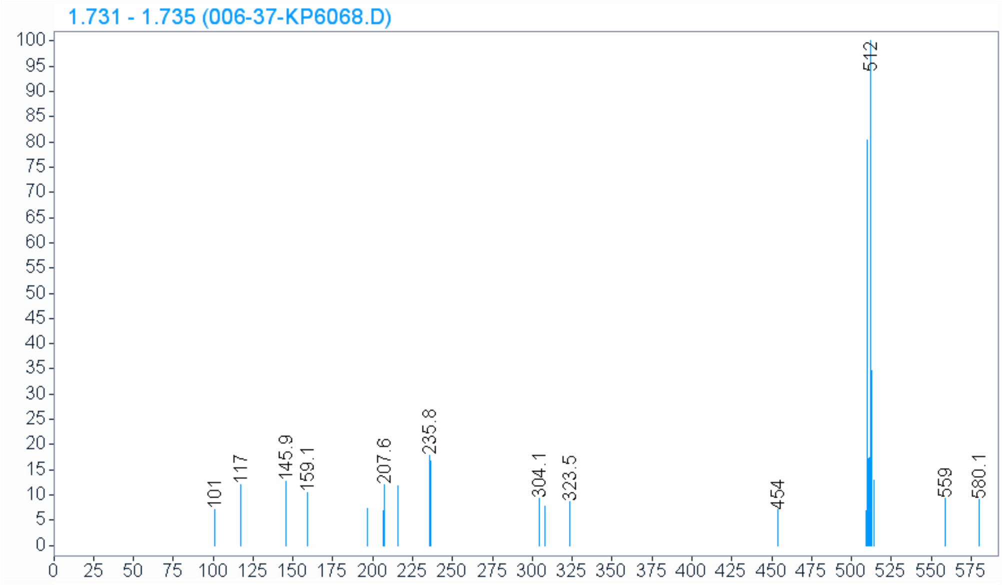

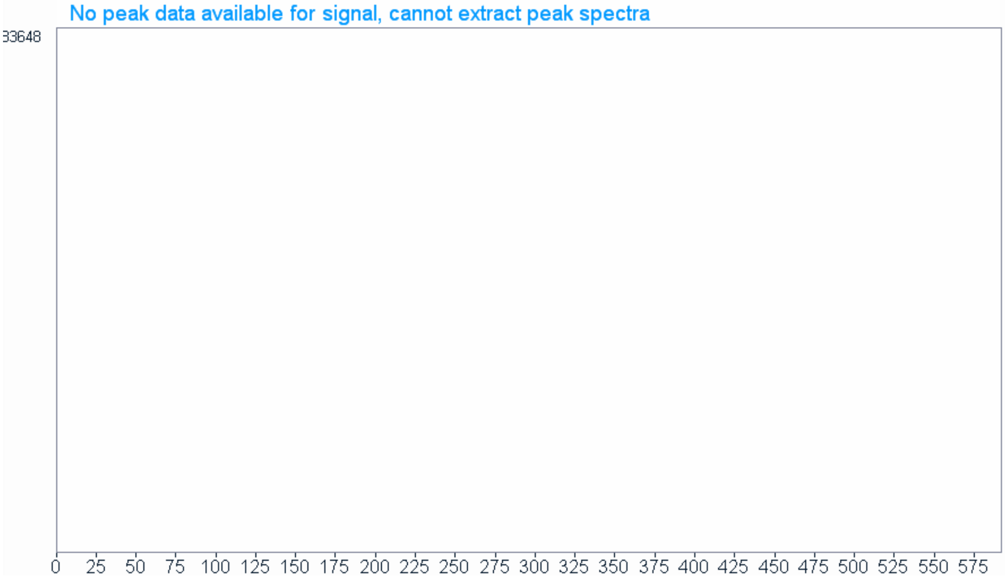

Compound Name: (Z)-*N*-(5-((5-chloro-1H-indol-3-yl)methylene)-4-oxo-4,5-dihydrothiazol-2-yl)naphthalene-1-sulfonamide

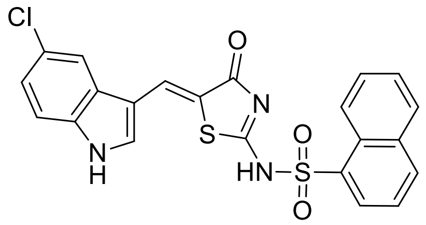

Chemical Formula: $C_{22}H_{14}ClN_3O_3S_2$
Exact Mass: 467.02
Molecular Weight: 467.95

Compound Code: 66 (KP7069)

Obtained Weight & Yield: 66 mg, 29%

Purity (by LCMS and ^{1}H NMR): > 99% by ^{1}H-NMR and LCMS

Appearance: yellow solid

Solubility: DMSO, slightly soluble in acetone and methanol

Melting Point: > 306 °C (dec.)

TLC Rf (and conditions): N/A

IR Analysis (including assignment): IR (neat): 3386 (N-H), 3185 (C-H aromatic), 1703 (C=O), 1558 (aromatic C-C), 1312 (sulfonamide), 11108 (C-N), 702 (C-Cl) cm^{-1}

^{1}H NMR Analysis: ^{1}H NMR (400 MHz, DMSO) δ 12.95 (br, s, 1H, NH), 12.35 (br, s, 1H, NH), 8.63 (d, *J* = 8.5 Hz, 1H), 8.35 – 8.29 (m, 2H), 8.12 (d, *J* = 8.1 Hz, 1H), 8.07 – 8.06 (m, 2H), 7.96 (d, *J* = 2.9 Hz, 1H), 7.79 – 7.75 (m, 1H), 7.73 – 7.67 (m, 2H), 7.56 (d, *J* = 8.7 Hz, 1H), 7.27 (dd, *J* = 8.7, 1.9 Hz, 1H) ppm.

^{13}C NMR Analysis: ^{13}C NMR (101 MHz, DMSO) δ 166.2, 165.2, 135.5, 134.8, 134.6, 133.8, 130.9, 129.0, 128.3, 128.1, 128.0, 127.7, 127.1, 126.4, 126.1, 124.9, 124.6, 123.3, 118.3, 114.7, 114.2, 110.1 ppm.

MS Analysis (low res): LRMS (ESI-) *m/z* (%): 466 (*M*-H, $C_{22}H_{13}{}^{35}ClN_3O_3S_2$, 100%), 468 (*M*-H, $C_{22}H_{13}{}^{37}ClN_3O_3S_2$, 35%)

MS Analysis (high res): Exact mass calculated for $C_{22}H_{13}ClN_3O_3S_2$ [*M*-H]$^-$, 466.0100. Found 466.0091.

HPLC method details: Column: Zorbax SB-C18 Rapid Resolution HT 2.1x50mm 1.8-Micron; Method: LCMS ISOCRATIC 60%B 0.4MLMIN-1.M filename: KP7069; Peak retention time: 1.385 mins; Area (%): 100

Procedure: To a 10 mL microwave vial was added *N*-(4-oxo-4,5-dihydrothiazol-2-yl)naphthalene-1-sulfonamide (159 mg, 0.49 mmol), 5-chloroindole-3-carboxaldehyde (109 mg, 0.54 mmol, 1.1 eq), ethanol (3 mL) and a catalytic amount of the benzoic acid/piperidine catalyst (approximately 5 drops). The suspension was heated using microwave irradiation (200 W, 120 °C) for 50 min then allowed to precipitate at in the freezer. The resulting precipitate was collected by vacuum filtration and washed with cold ethanol and cold ether to give the desired product (66 mg, 29%).

Other analyses, reference papers, previously obtained data, comments, etc:

PerkinElmer Spectrum Version 10.4.00
Thursday, 21 November 2019 11:23 AM

Analyst research

Date Thursday, 21 November 2019 11:23 AM

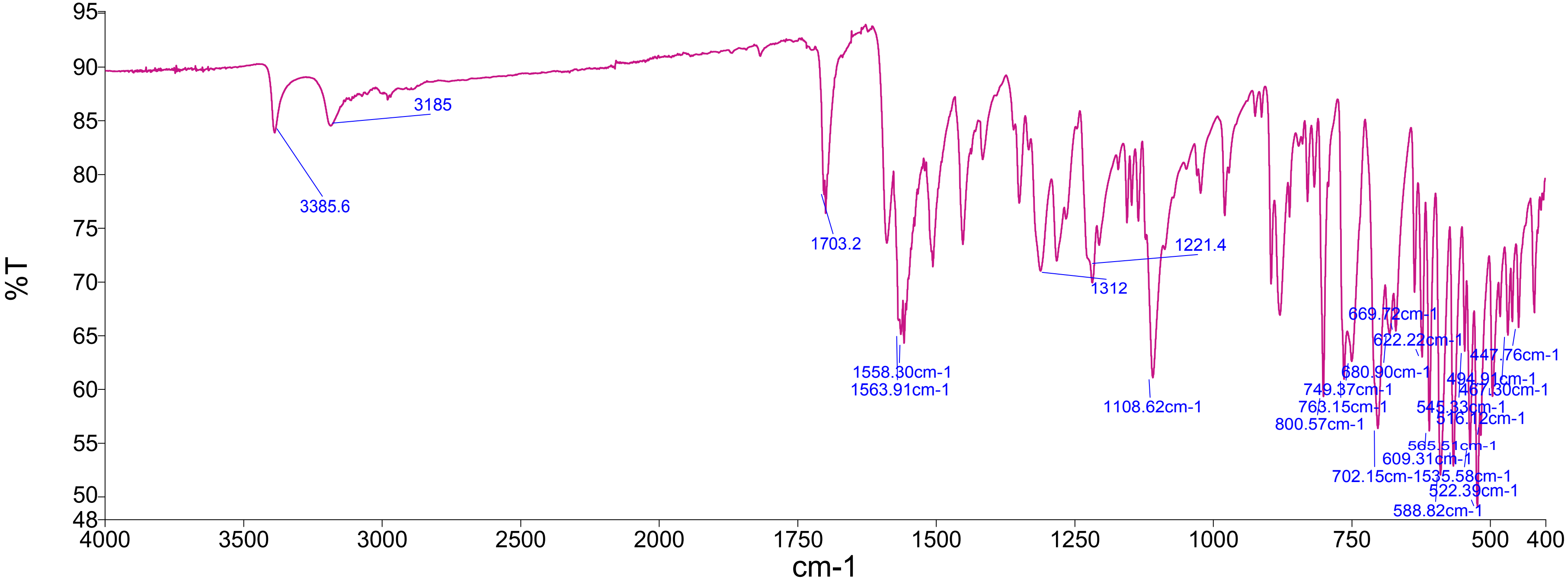

Sample Name	Description	Quality Checks
KP7069	Sample 245 By research Date Thursday, November 21 2019	The Quality Checks do not report any warnings for the sample.

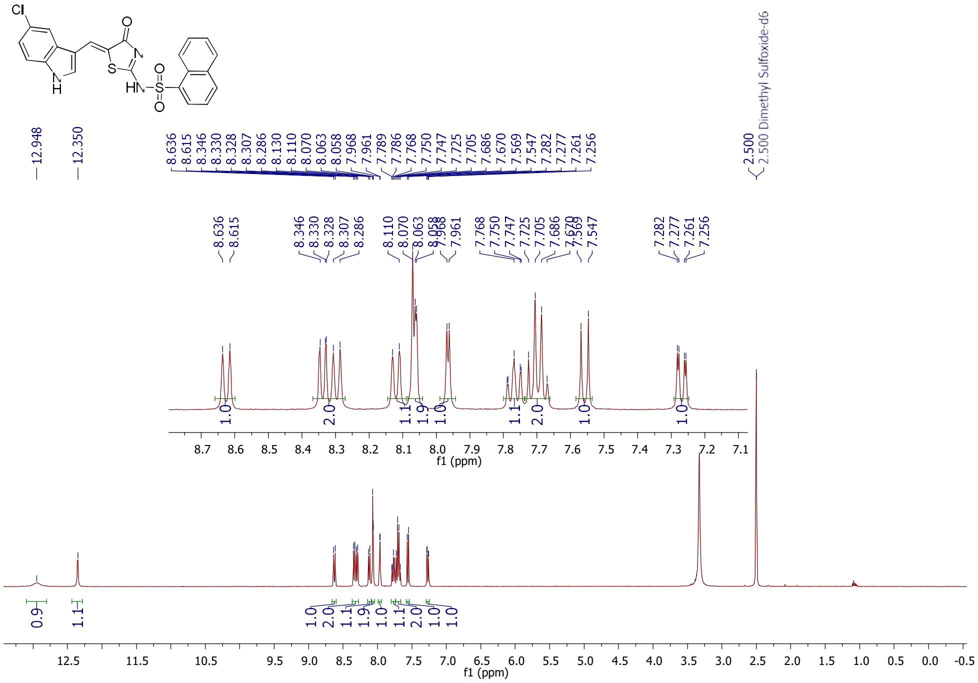
Cl
O
N
S
HN
S
O
O
N
H
12.948
12.350
8.636 8.615 8.346 8.330 8.328 8.307 8.286 8.130 8.110 8.070 8.063 8.058 7.968 7.961 7.789 7.786 7.768 7.750 7.747 7.725 7.705 7.686 7.670 7.569 7.547 7.282 7.277 7.261 7.256
2.500
2.500 Dimethyl Sulfoxide-d6
f1 (ppm)

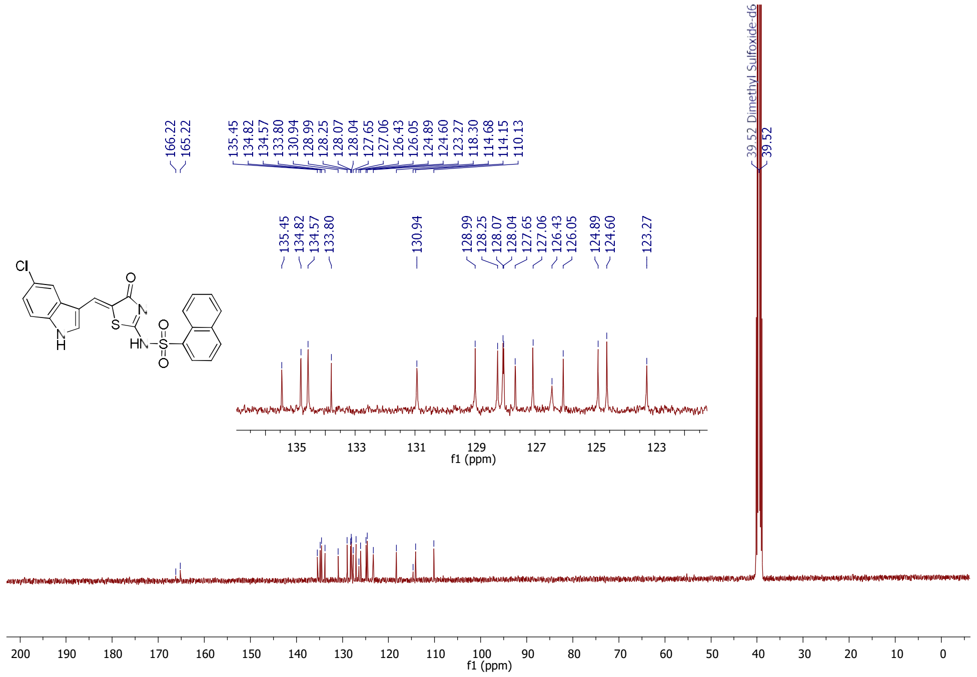
166.22
165.22
135.45 134.82 134.57 133.80 130.94 128.99 128.25 128.07 128.04 127.65 127.06 126.43 126.05 124.89 124.60 123.27 118.30 114.68 114.15 110.13
39.52 Dimethyl Sulfoxide-d6
39.52
Cl
O
N
S
HN
S
O
O
N
H
f1 (ppm)

LCMS Report

Agilent Technologies

**Data file:**	D:\Chem32\1\Data\KP_DS_IND2 2019-11-15 11-06-04\001-43-KP7069.D		
**Sample name:**	KP7069		
**Description:**			
**Sample amount:**	0.000	**Sample type:**	Sample
**Instrument:**	LCMS	**Location:**	43
**Injection date:**	11/15/2019 11:07:45 AM	**Injection:**	1 of 1
**Acq. method:**	LCMS ISOCRATIC 60% B 0.4MLMIN-1.M	**Injection volume:**	2.000
**Analysis method:**	LCMS ISOCRATIC 60%B 0.4MLMIN-1.M	**Acq. operator:**	SYSTEM
**Last changed:**	5/8/2019 8:55:04 AM		

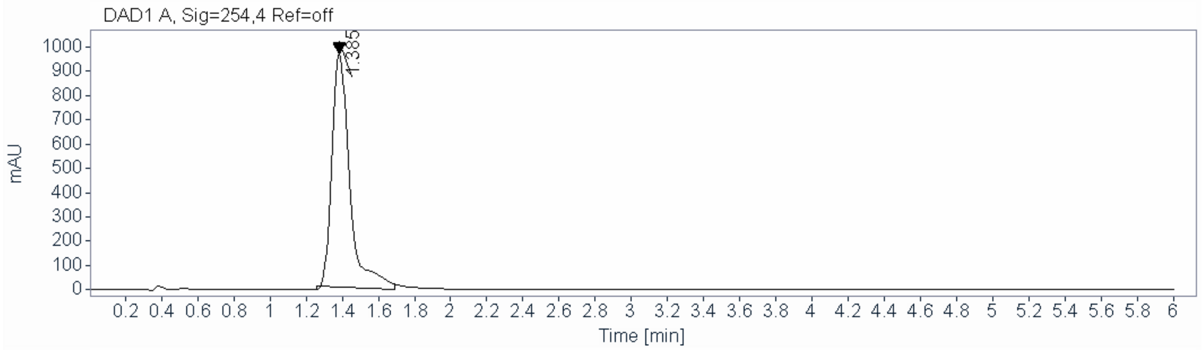

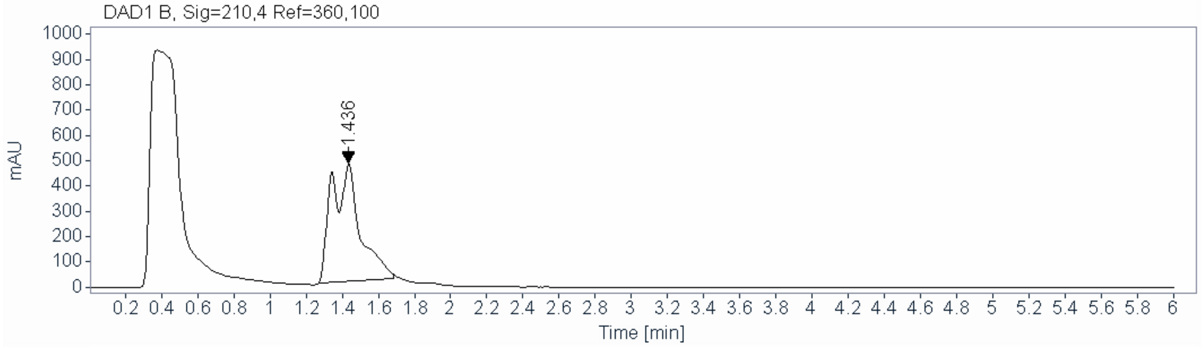

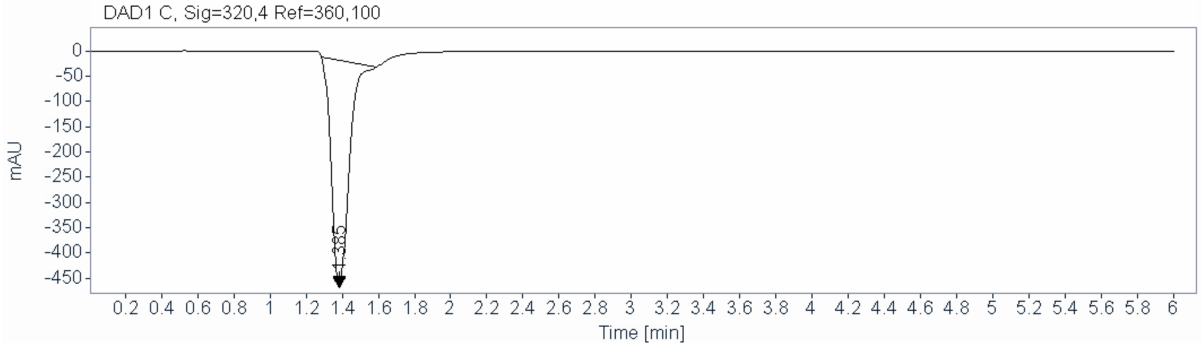

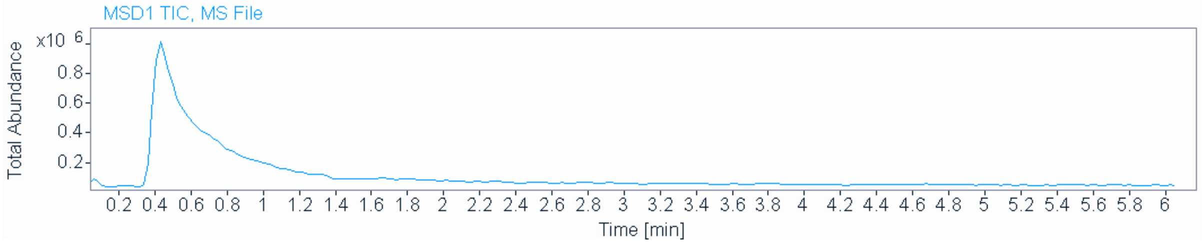

Agilent Technologies

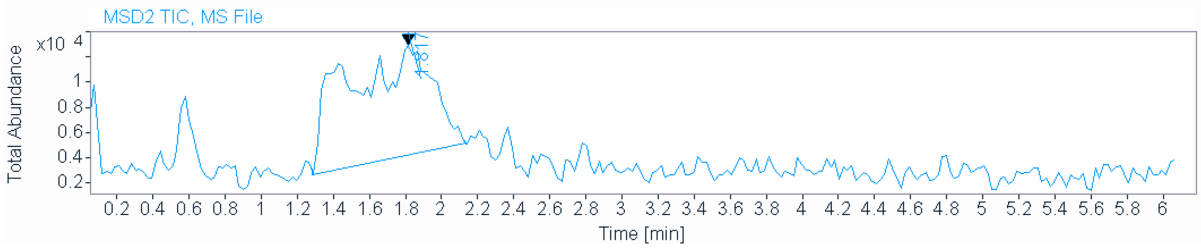

Signal: DAD1 A, Sig=254,4 Ref=off

RT [min]	Type	Width [min]	Area	Height	Area%	Name
1.385	MM	0.1150	6656.5171	964.6016	100.0000	
		Sum	6656.5171			

Signal: DAD1 B, Sig=210,4 Ref=360,100

RT [min]	Type	Width [min]	Area	Height	Area%	Name
1.436	MM	0.1814	5085.3188	467.2106	100.0000	
		Sum	5085.3188			

Signal: DAD1 C, Sig=320,4 Ref=360,100

RT [min]	Type	Width [min]	Area	Height	Area%	Name
1.385	PP N	0.1027	2787.3276	452.3380	100.0000	
		Sum	2787.3276			

Signal: MSD2 TIC, MS File

RT [min]	Type	Width [min]	Area	Height	Area%	Name
1.817	MM	0.5198	286472.3438	8653.0830	100.0000	
		Sum	286472.3438			

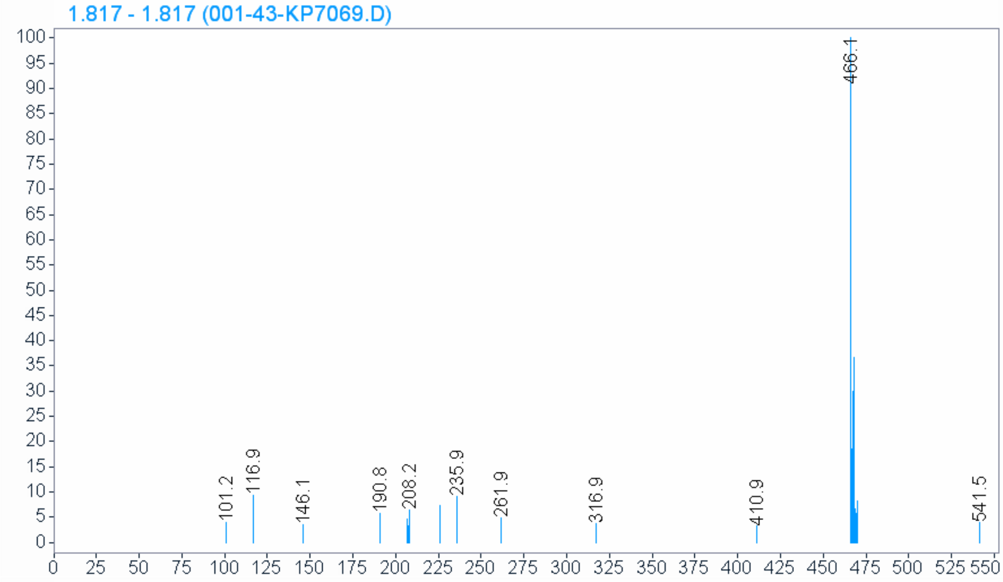

No peak data available for signal, cannot extract peak spectra

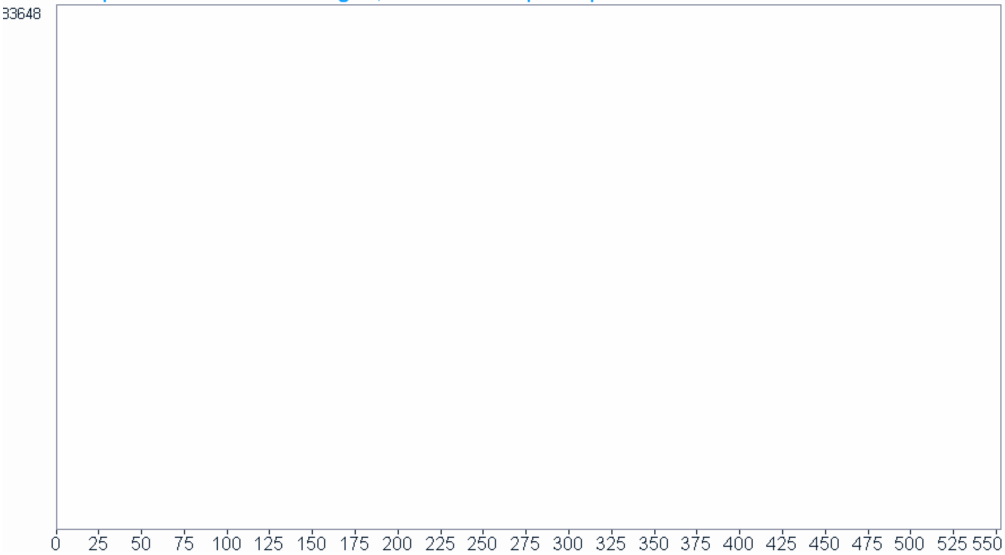

Compound Name: (Z)-*N*-(5-(benzofuran-3-ylmethylene)-4-oxo-4,5-dihydrothiazol-2-yl)naphthalene-1-sulfonamide

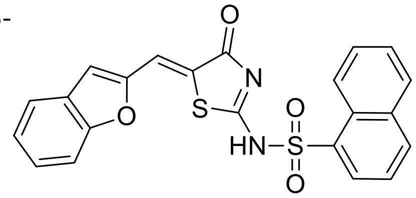

Chemical Formula: $C_{22}H_{14}N_2O_4S_2$
Exact Mass: 434.04
Molecular Weight: 434.49

Compound Code: 67 (KP6071)

Obtained Weight & Yield: 213 mg (75%)

Purity (by LCMS and ^{1}H NMR): > 99% by ^{1}H-NMR and LCMS

Appearance: bright yellow powder

Solubility: DMSO

Melting Point: < 279 °C (dec.)

TLC Rf (and conditions): 0.25 (10% MeOH in DCM)

IR Analysis (including assignment): IR (neat): ν_{max} = 3117, 2943 (aromatic CH), 1690 (C=O), 1557 (N-H), 1335 (sulfonamide), 1125 (C-N) cm^{-1}

^{1}H NMR Analysis: ^{1}H NMR (400 MHz, DMSO) δ 13.16 (broad, 1H, NH), 8.62 (d, *J* = 8.7 Hz, 1H), 8.34 (dd, *J* = 7.4, 1.1 Hz, 1H), 8.31 (d, *J* = 8.3 Hz, 1H), 8.12 (d, *J* = 8.0 Hz, 1H), 7.81 – 7.67 (m, 6H), 7.59 (s, 1H), 7.52 – 7.48 (m, 1H), 7.38 – 7.34 (m, 1H) ppm.
Ethanol Impurity at 1.05 ppm (0.60%)

^{13}C NMR Analysis: ^{13}C NMR (101 MHz, DMSO) δ 166.5, 166.3, 155.5, 150.8, 135.4, 134.7, 133.8, 129.0, 128.3, 128.1, 127.8, 127.7, 127.6, 127.1, 124.9, 124.7, 124.1, 122.7, 122.1, 120.0, 115.4, 111.6 ppm

MS Analysis (low res): LRMS (ESI-) *m/z* (%): 433 (*M*-H, $C_{22}H_{13}N_2O_4S_2$, 100%)

HPLC method details: Column: Zorbax SB-C18 Rapid Resolution HT 2.1x50mm 1.8-Micron; Method: LCMS ISOCRATIC 60%B 0.4MLMIN-1.M filename: KP6074; Peak retention time: 2.03 mins; Area (%): 100.
NOTE: this sample was entered in under the wrong name (KP6071 entered as KP6074)

Procedure: To a microwave vial was added the *N*-(4-oxo-4,5-dihydrothiazol-2-yl)naphthalene-1-sulfonamide (200 mg, 0.65 mmol), 2-benzofuran carboxaldehyde (0.1 mL, 0.72 mmol, 1.1 eq), ethanol (3 mL) and the benzoic acid/piperidine catalyst (10 drops). The reaction was treated with microwave irradiation (120°C, 30 min). A yellow precipitate was observed and the reaction mixture was cooled overnight. The precipitate was collected using vacuum filtration and was washed with H_2O (5 mL), ethanol (5 mL) and ether (10 mL) to give the desired product as a bright yellow powder (213 mg, 75%).

Other analyses, reference papers, previously obtained data, comments, etc:

Analyst	analyst1
Date	Thursday, 4 July 2019 12:21 PM

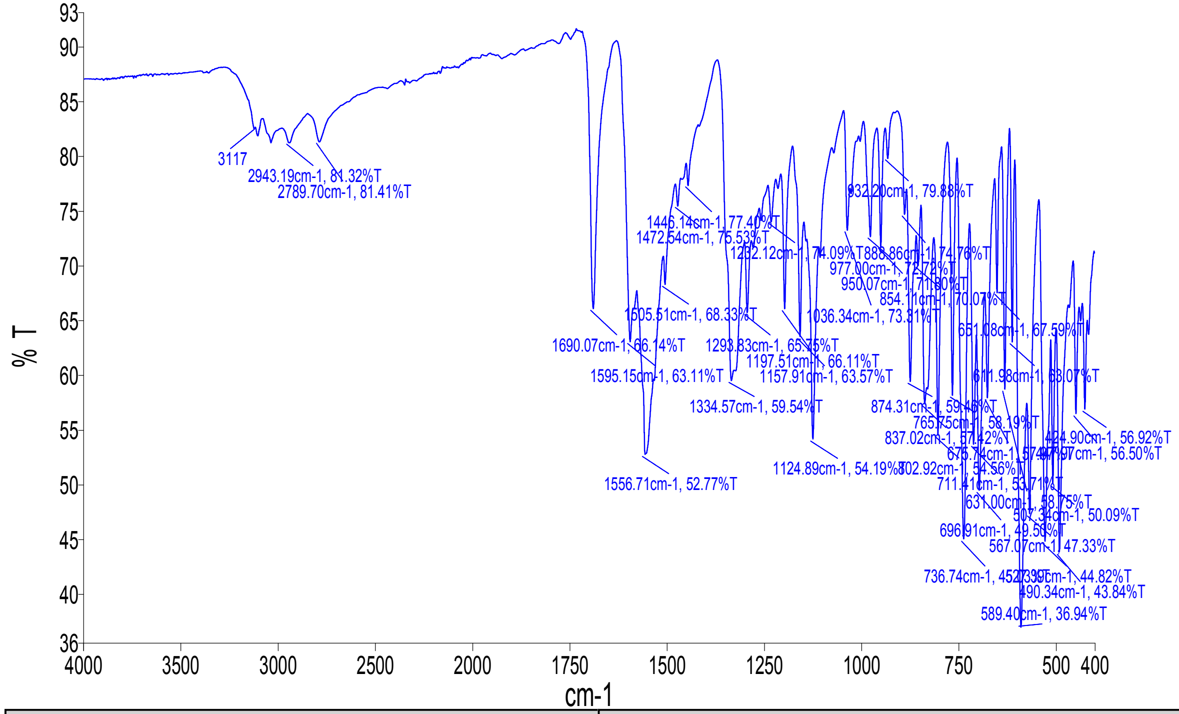

Sample Name	Description	Quality Checks
KP6071	Sample 005 By Analyst1 Date Thursday, July 04 2019	The Quality Checks do not report any warnings for the sample.

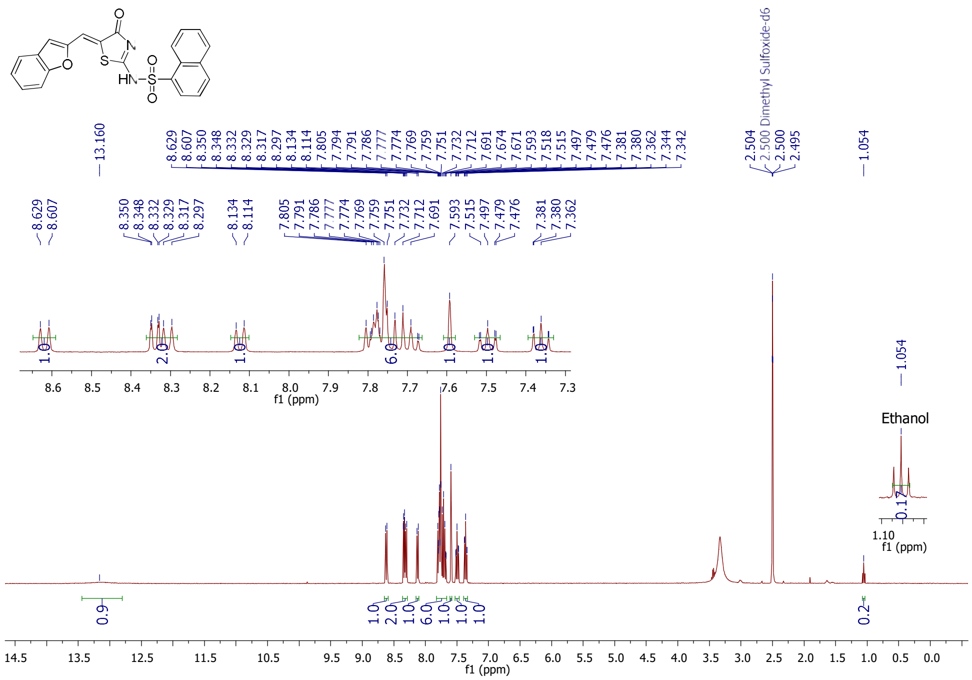

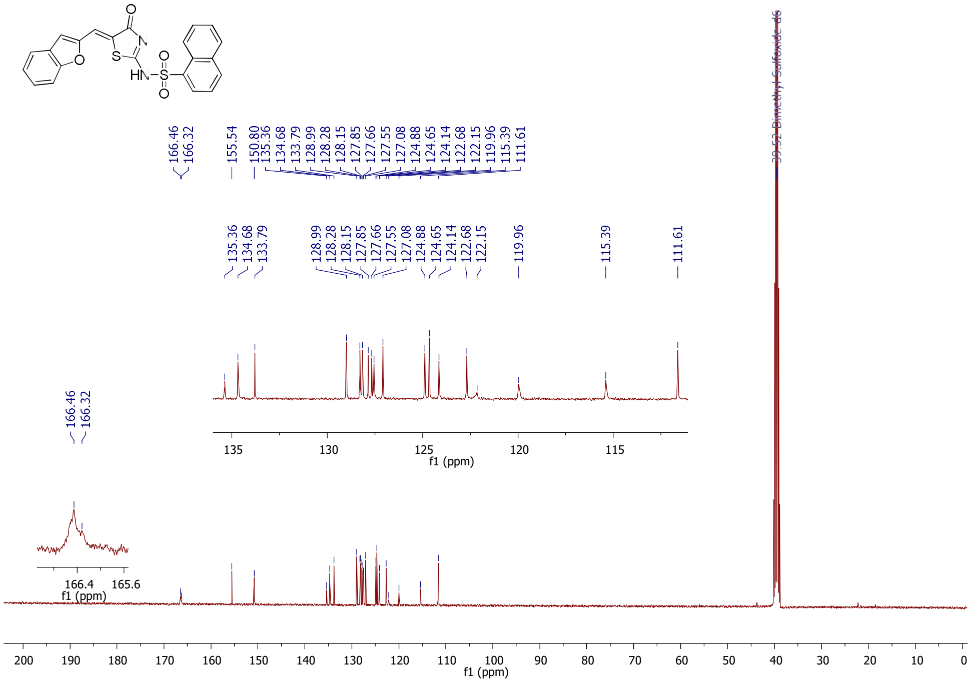

LCMS Report

Agilent Technologies

**Data file:**	D:\Chem32\1\Data\KP\KP60647174 2019-08-13 10-55-55\004-43-KP6074.D		
**Sample name:**	KP6074		
**Description:**			
**Sample amount:**	0.000	**Sample type:**	Sample
**Instrument:**	LCMS	**Location:**	43
**Injection date:**	8/13/2019 11:20:21 AM	**Injection:**	1 of 1
**Acq. method:**	LCMS ISOCRATIC 60% B 0.4MLMIN-1.M	**Injection volume:**	2.000
**Analysis method:**	LCMS ISOCRATIC 60%B 0.4MLMIN-1.M	**Acq. operator:**	SYSTEM
**Last changed:**	5/8/2019 8:55:04 AM		

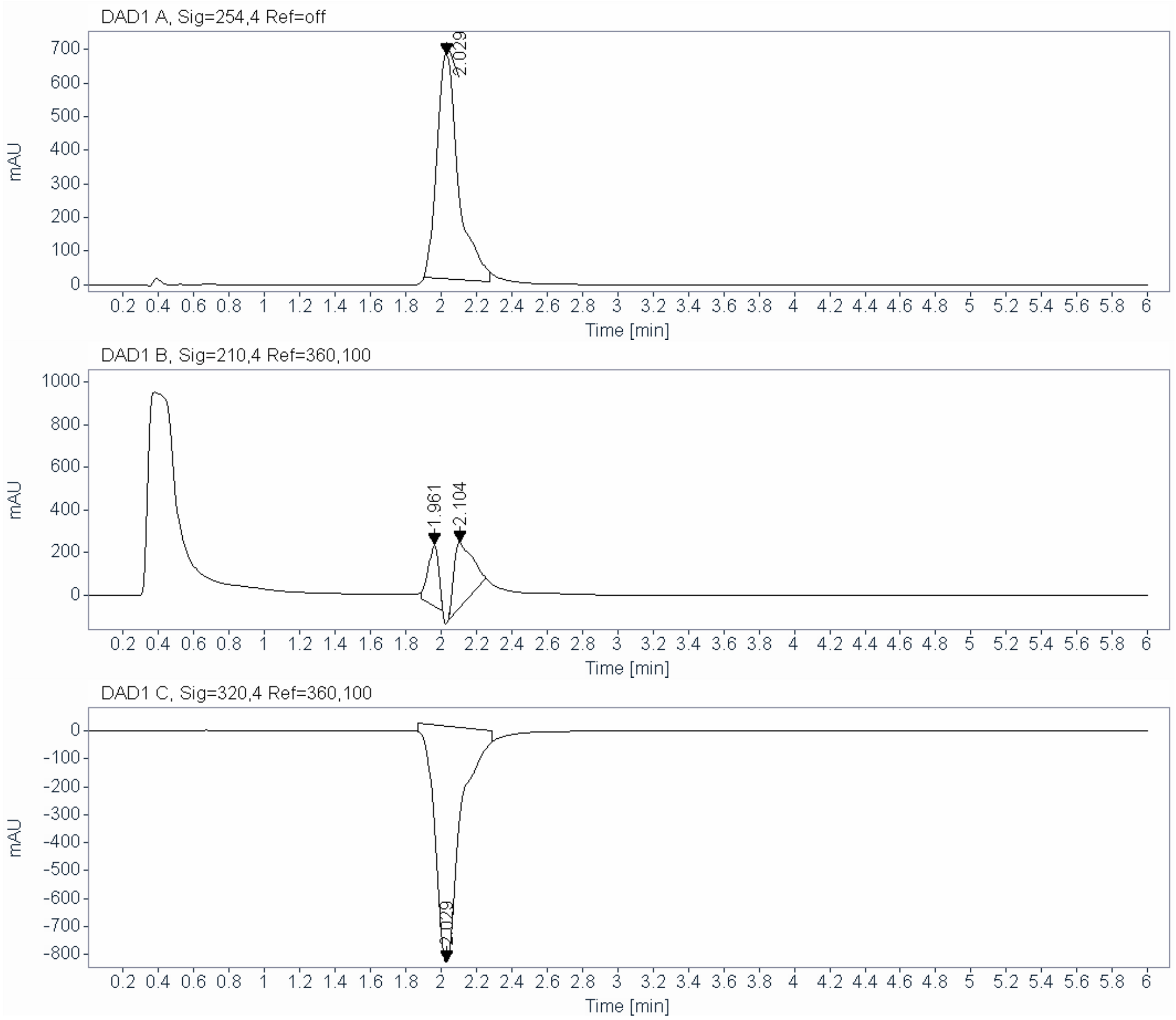

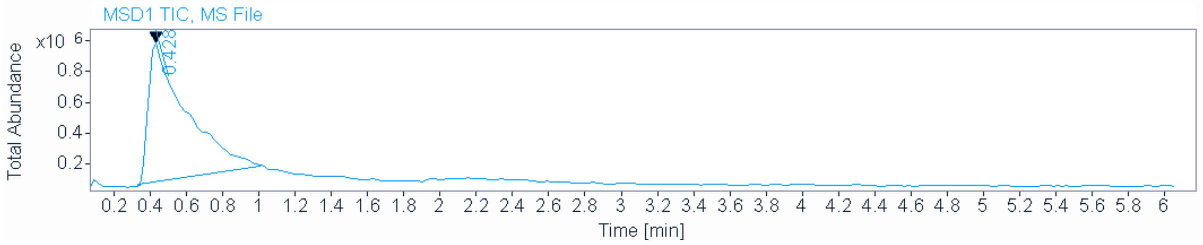

Agilent Technologies

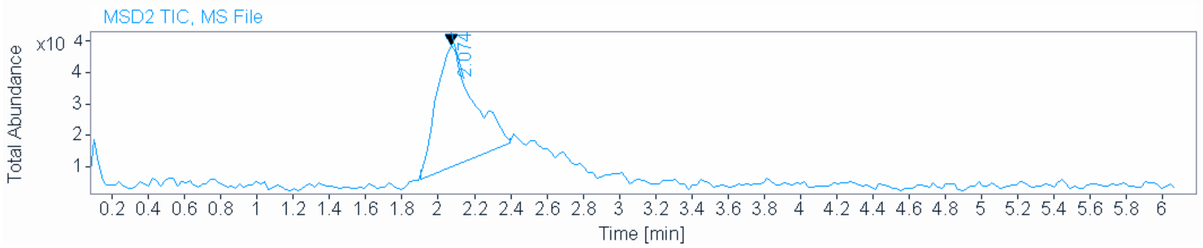

Signal: DAD1 A, Sig=254,4 Ref=off

RT [min]	Type	Width [min]	Area	Height	Area%	Name
2.029	PM	0.1409	5655.8486	668.8738	100.0000	
		Sum	5655.8486			

Signal: DAD1 B, Sig=210,4 Ref=360,100

RT [min]	Type	Width [min]	Area	Height	Area%	Name
1.961	MM	0.0683	1173.8868	286.2641	35.7271	
2.104	MP	0.1139	2111.8162	309.0606	64.2729	
		Sum	3285.7030			

Signal: DAD1 C, Sig=320,4 Ref=360,100

RT [min]	Type	Width [min]	Area	Height	Area%	Name
2.029	MM N	0.1531	7779.3340	847.0196	100.0000	
		Sum	7779.3340			

Signal: MSD1 TIC, MS File

RT [min]	Type	Width [min]	Area	Height	Area%	Name
0.428	MM	0.2397	13085020.0000	909641.3125	100.0000	
		Sum	13085020.00			

Signal: MSD2 TIC, MS File

RT [min]	Type	Width [min]	Area	Height	Area%	Name
2.074	MM	0.2326	542571.9375	38873.6016	100.0000	
		Sum	542571.9375			

Agilent Technologies

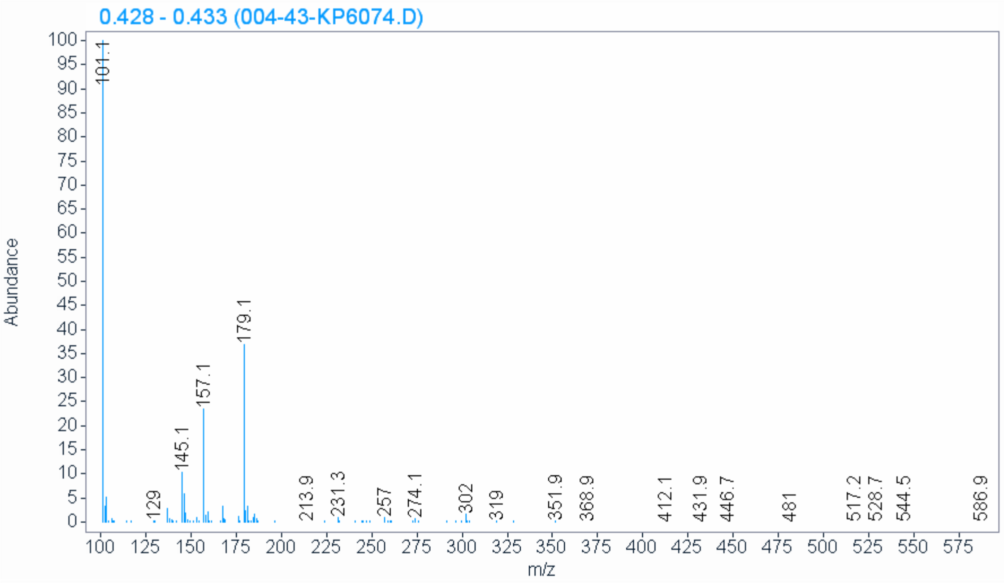

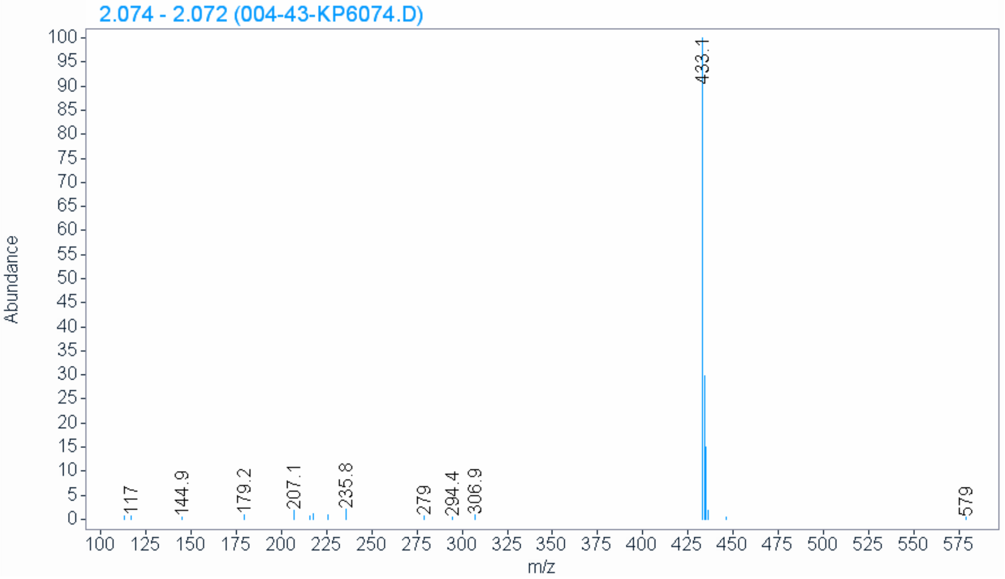

Compound Name: (Z)-*N*-(4-oxo-5-((1-(phenylsulfonyl)-1H-indol-3-yl)methylene)-4,5-dihydrothiazol-2-yl)naphthalene-1-sulfonamide

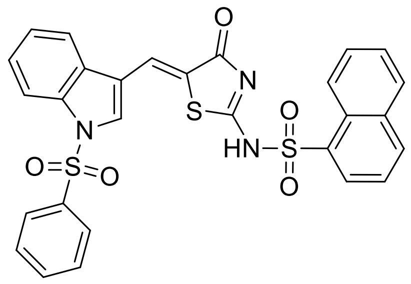

Chemical Formula: $C_{28}H_{19}N_3O_5S_3$
Exact Mass: 573.05
Molecular Weight: 573.66

Compound Code: 68 (KP6123)

Obtained Weight & Yield: 210 mg, 75%

Purity (by LCMS and ^{1}H NMR): 99% by ^{1}H-NMR and LCMS

Appearance: bright yellow solid

Solubility: DMSO, slightly soluble in methanol and acetone

Melting Point: > 300 °C (dec.)

TLC Rf (and conditions): N/A

IR Analysis (including assignment): IR (neat): ν_{max} = 3120 (NH), 3049, 2965 (aromatic C-H), 2784 (C-H), 1603 (C=O), 1551 (aromatic C-C), 1363, 1306 (sulfonamide), 1124 (C-N) cm^{-1}

^{1}H NMR Analysis: ^{1}H NMR (400 MHz, DMSO) δ 13.17 (br, s, 1H, NH), 8.63 (d, *J* = 8.5 Hz, 1H), 8.34 (t, *J* = 7.0 Hz, 2H), 8.15 (t, *J* = 7.9 Hz, 3H), 8.08 (s, 1H), 7.98 (dd, *J* = 14.7, 8.1 Hz, 2H), 7.92 (s, 1H), 7.80 – 7.70 (m, 4H), 7.68 – 7.63 (m, 2H), 7.48 (t, *J* = 7.5 Hz, 1H), 7.39 (t, *J* = 7.5 Hz, 1H) ppm.
Ethanol at 1.05 ppm (0.27%)

^{13}C NMR Analysis: ^{13}C NMR (101 MHz, DMSO) δ 166.0, 164.6, 136.2, 135.6, 135.2, 134.8, 133.81, 133.79, 130.1 (2C), 129.0, 128.7, 128.4, 128.2, 127.6, 127.2 (2C), 127.1 (2C), 126.4, 124.8, 124.7, 124.5, 122.9, 122.8, 120.27, 116.6, 113.3 ppm.

MS Analysis (low res): LRMS (ESI-) *m/z* (%): 572 (*M*-H, $C_{28}H_{18}N_3O_5S_2$, 100%)

MS Analysis (high res): Exact mass calculated for $C_{28}H_{18}N_3O_5S_2$ [*M*-H]$^-$, 572.0400. Found 572.0414.

HPLC method details: Column: Zorbax SB-C18 Rapid Resolution HT 2.1x50mm 1.8-Micron; Method: LCMS ISOCRATIC 60%B 0.4MLMIN-1.M filename: KP6123; Peak retention time: 3.641 mins; Area (%): 98.

Procedure: To a 10 mL microwave vial was added *N*-(4-oxo-4,5-dihydrothiazol-2-yl)naphthalene-1-sulfonamide (150 mg, 0.49 mmol), 1-phenylsulfonyl-3-indole carboxaldehyde (156 mg, 0.54 mmol, 1.1 eq), ethanol (3 mL) and a catalytic amount of the benzoic acid/piperidine catalyst (approximately 2 drops). The suspension was heated using microwave irradiation (200 W, 120 °C) for 10 min then allowed to precipitate at in the freezer. The resulting precipitate was collected by vacuum filtration and washed with cold ethanol and cold ether to give the desired product (210 mg, 75%).

Other analyses, reference papers, previously obtained data, comments, etc:

PerkinElmer Spectrum Version 10.4.00
Tuesday, 23 July 2019 11:54 AM

Analyst research
Date Tuesday, 23 July 2019 11:54 AM

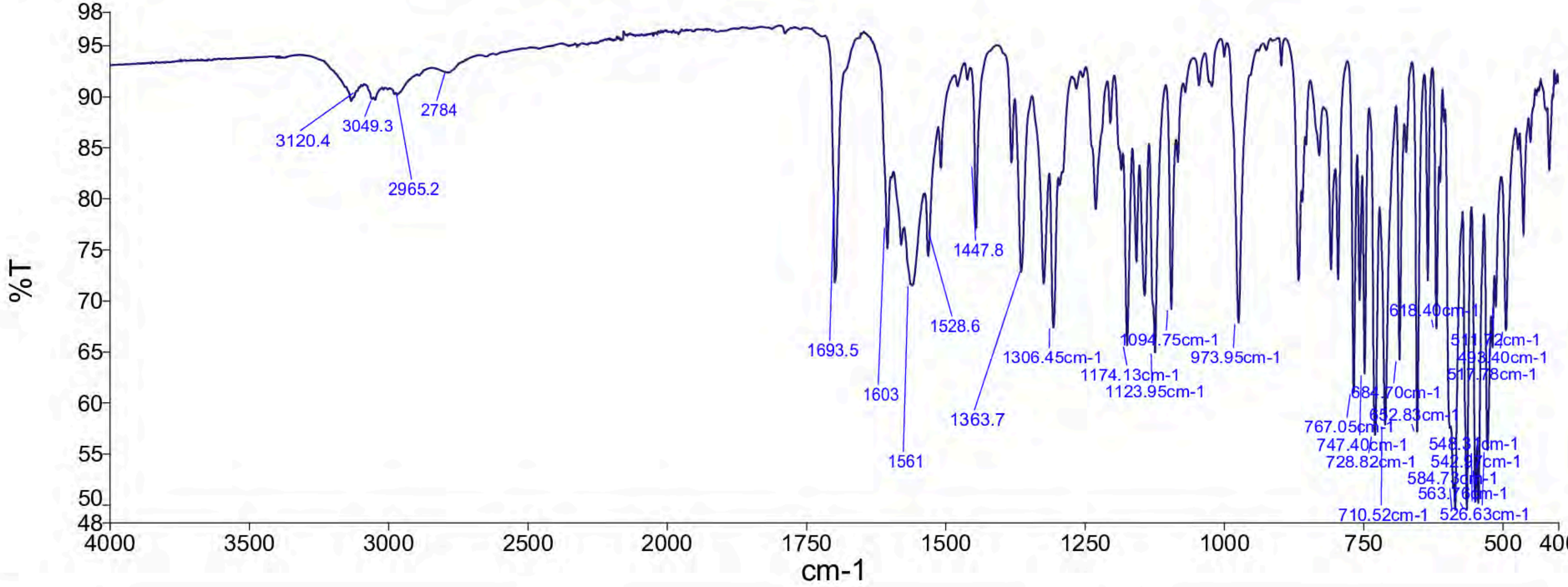

Sample Name	Description	Quality Checks
KP6123	Sample 239 By research Date Tuesday, July 23 2019	The Quality Checks do not report any warnings for the sample.

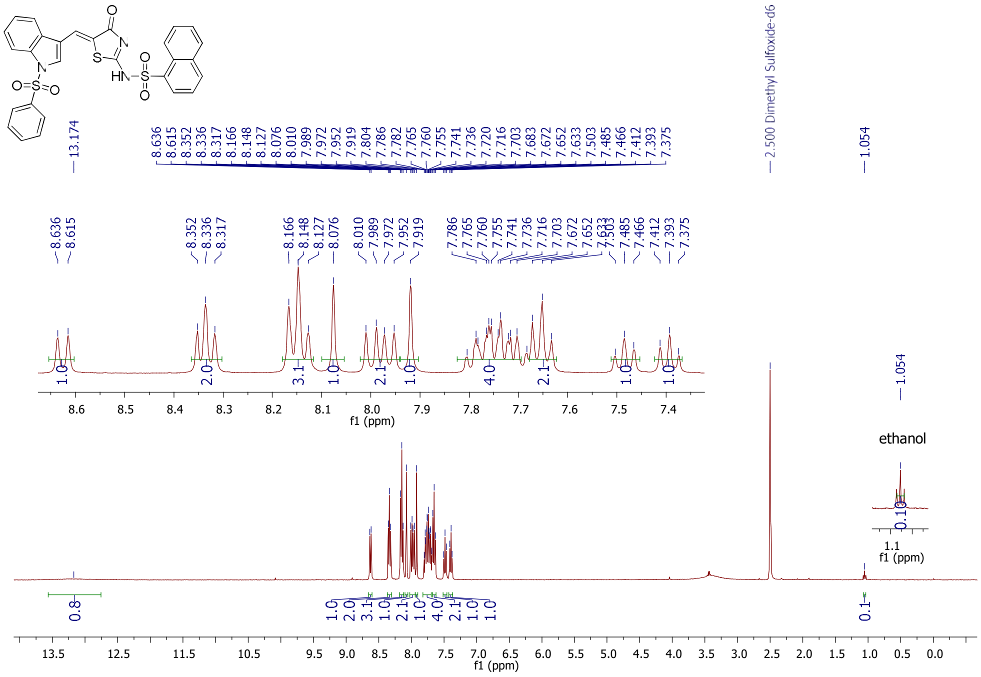
13.174
2.500 Dimethyl Sulfoxide-d6
1.054
ethanol
f1 (ppm)

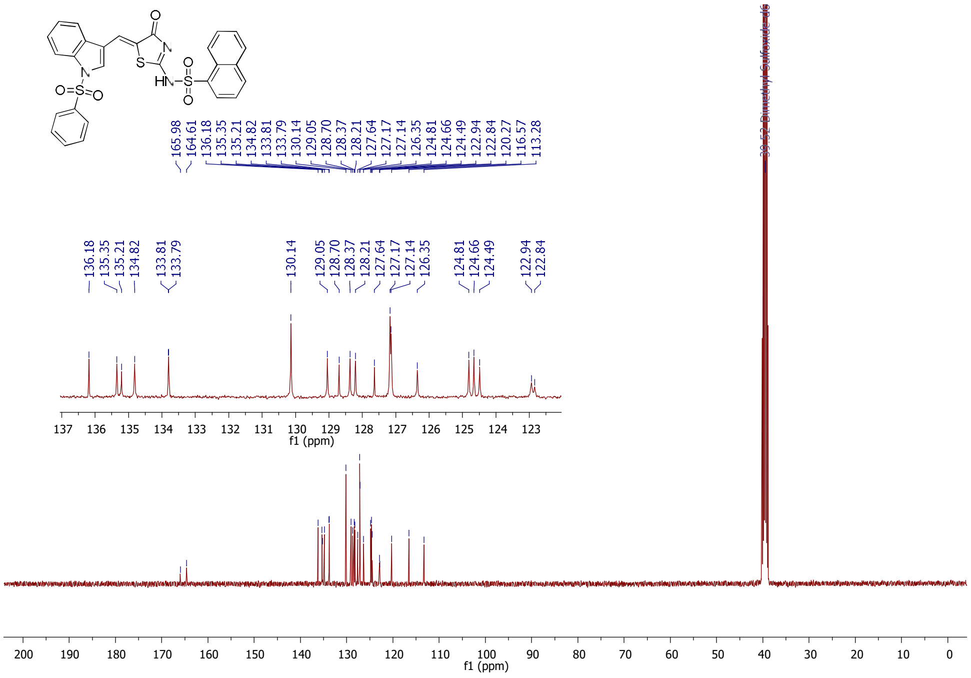
165.98
164.61
136.18
135.35
135.21
134.82
133.81
133.79
130.14
129.05
128.70
128.37
128.21
127.64
127.17
127.14
126.35
124.81
124.66
124.49
122.94
122.84
120.27
116.57
113.28
f1 (ppm)

LCMS Report

Data file: D:\Chem32\1\Data\KP\KP_DS_11NOV 2019-11-11 14-45-23\007-46-KP6123.D
Sample name: KP6123
Description:
Sample amount: 0.000
Sample type: Sample

Instrument: LCMS
Location: 46
Injection date: 11/11/2019 3:33:53 PM
Injection: 1 of 1
Acq. method: LCMS ISOCRATIC 60% B 0.4MLMIN-1.M
Injection volume: 2.000
Analysis method: LCMS ISOCRATIC 60%B 0.4MLMIN-1.M
Acq. operator: SYSTEM
Last changed: 5/8/2019 8:55:04 AM

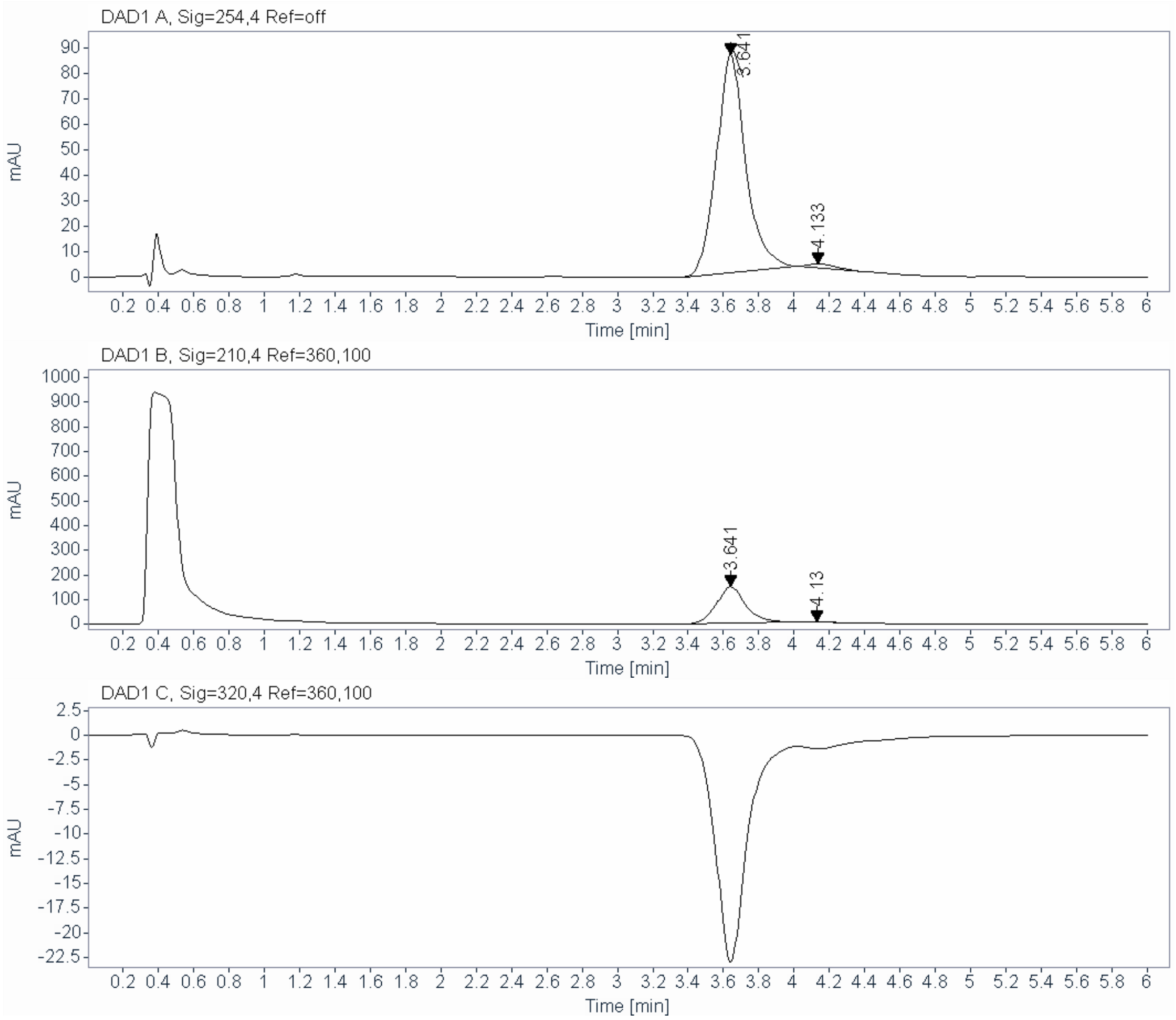

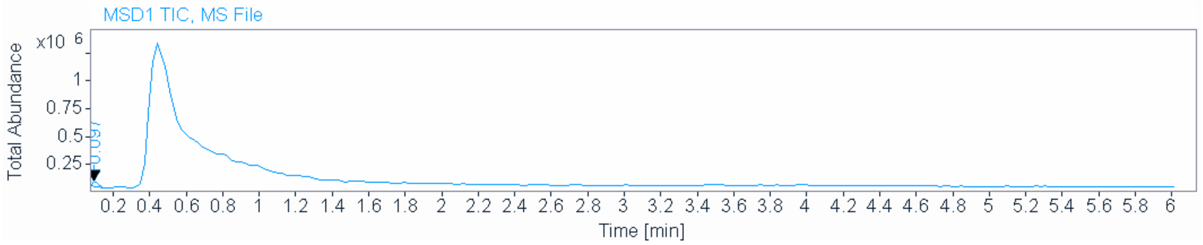

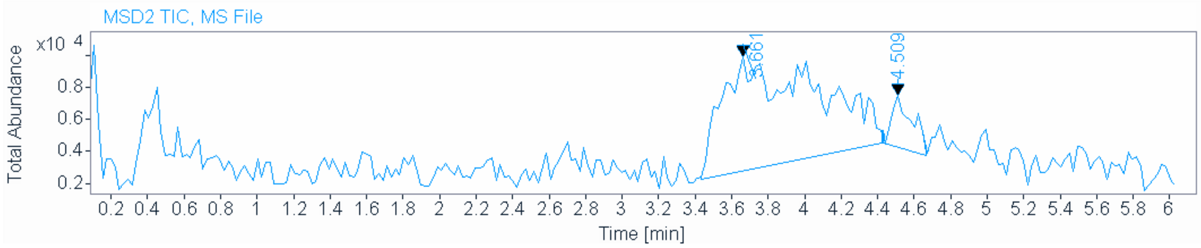

Signal: DAD1 A, Sig=254,4 Ref=off

RT [min]	Type	Width [min]	Area	Height	Area%	Name
3.641	BB	0.1710	991.2344	85.6856	98.1274	
4.133	BB	0.1743	18.9156	1.6938	1.8726	
		Sum	1010.1500			

Signal: DAD1 B, Sig=210,4 Ref=360,100

RT [min]	Type	Width [min]	Area	Height	Area%	Name
3.641	BB	0.1737	1745.4261	147.8627	97.6944	
4.130	BB	0.1560	41.1921	3.4826	2.3056	
		Sum	1786.6183			

Signal: MSD1 TIC, MS File

RT [min]	Type	Width [min]	Area	Height	Area%	Name
0.097	BB	0.0328	82586.1406	41936.1406	100.0000	
		Sum	82586.1406			

Signal: MSD2 TIC, MS File

RT [min]	Type	Width [min]	Area	Height	Area%	Name
3.661	MM	0.5579	245469.3281	7332.5444	90.4466	
4.509	MM	0.1314	25927.7051	3288.3113	9.5534	
		Sum	271397.0332			

Agilent Technologies

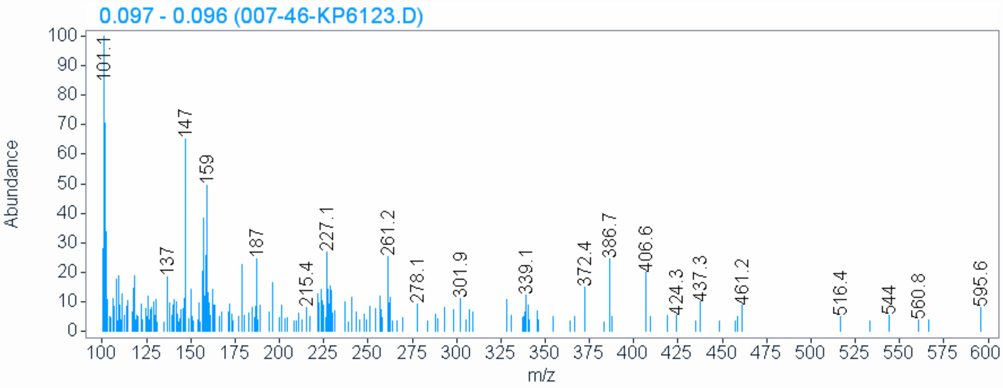

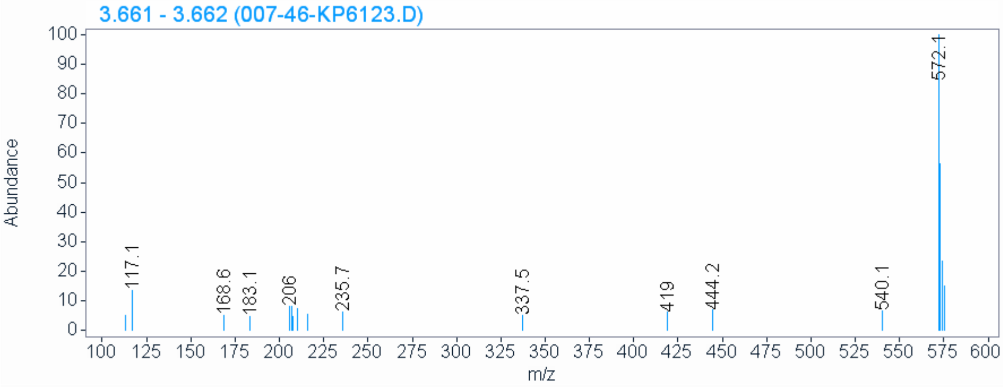

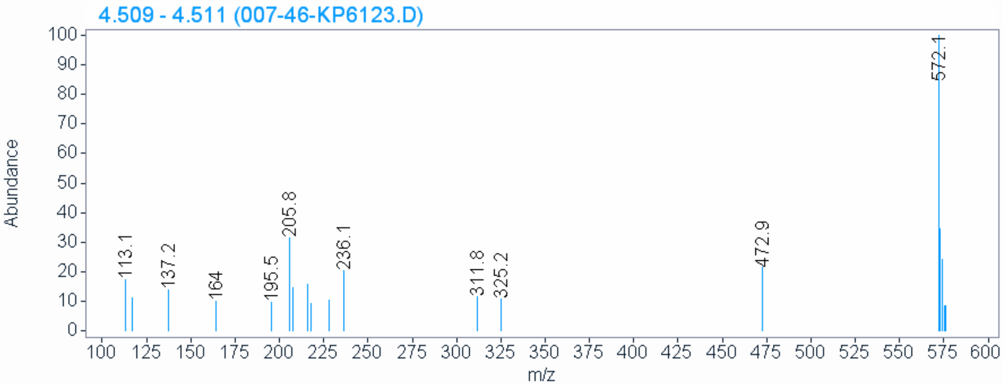

Supplement: Supplementary file 1 — Supplementary Material [file CMDC-20-e202500321-s001.pdf]
